# Adamantyl-BOX Ligand Enabling Stereoselective Synthesis of Axially Chiral Aldehydes Bearing Vicinal Stereogenic Centers

**DOI:** 10.1021/acscentsci.6c01081

**Published:** 2026-09-09

**Authors:** Er-Jun Hao, Xiao-Bo Ding, Jing-Ran Shan, Zhixian Wu, Xin Wang, Renxu Cao, Fangyi Jin, K. N. Houk, Lei Shi

**Affiliations:** † State Key Laboratory of Antiviral Drugs, Pingyuan Laboratory, 66519 School of Pharmaceutical Sciences, Henan Normal University, Xinxiang, Henan 453007, China; ‡ Department of Chemistry and Biochemistry, 8783 University of California Los Angeles, Los Angeles, California 90095 United States; § 12399 Cancer Hospital of Dalian University of Technology, School of Chemistry, Dalian University of Technology, Dalian 116024, China

## Abstract

Biaryl frameworks combining axial and multiple central chiralities are highly valuable in synthesis and drug discovery. However, simultaneous construction of axial and vicinal central chiralities faces severe matching/mismatching limitations. Herein, we describe a dual photoredox/nickel-catalytic system for desymmetrization of biaryl dialdehydes. By employing a bisoxazoline ligand bearing bulky adamantylphenyl side arms, its chiral cavity and distal substituent effects work synergistically to achieve high stereoselectivity, furnishing homoallylic alcohols with up to >20:1 dr and 99% ee. DFT calculations demonstrate that stereoselectivity is predominantly governed by a complex network of noncovalent interactions between the ligand and substrate.

## Graphical abstract

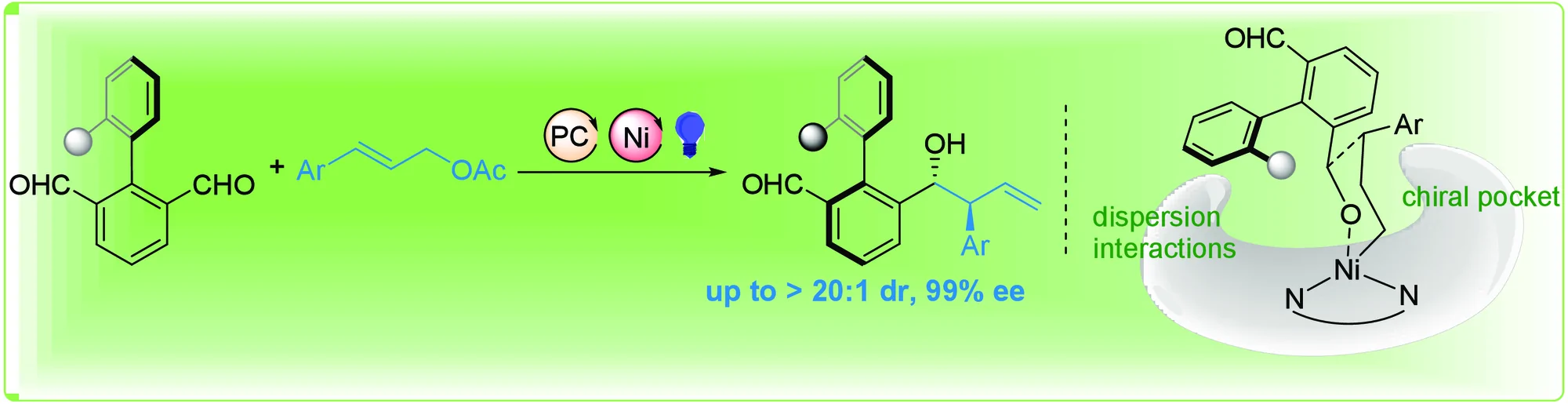

Axially chiral biaryl motifs are widely found in bioactive natural products, pharmaceuticals, and chiral catalysts/ligands (Figure 1a).^1–16^ Biaryl atropisomers bearing additional contiguous stereogenic centers are particularly appealing, as their complex three-dimensional architectures can enhance molecular binding affinity and stereocontrol performance in asymmetric catalysis.^17–22^ Nevertheless, the one-step concurrent construction of axial and multiple central chiralities remains an enduring synthetic challenge, largely due to inherent match/mismatch effects among stereogenic elements, which often lead to unsatisfactory stereoselectivity.

**Figure 1:**
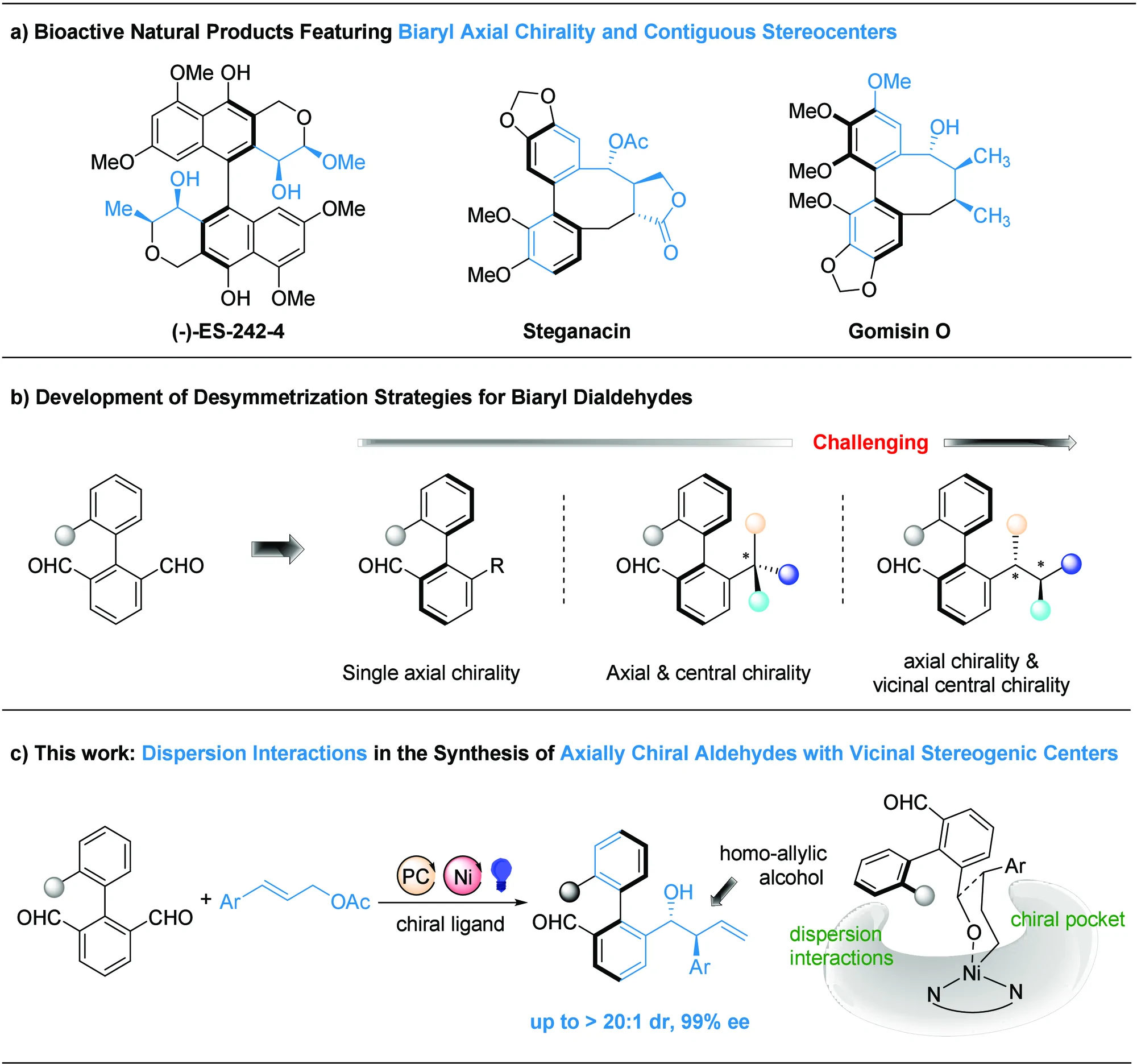
a). Representative natural products containing both axial and chiral alcohol moieties; b) Strategy for desymmetrization of biaryl dialdehydes; c) Synthesis of axially chiral aldehydes through carbonyl allylation reaction.

Aldehyde groups serve as versatile synthetic building blocks. Accordingly, desymmetrization of biaryl dialdehydes has emerged as a powerful route to axially chiral biaryl aldehydes, and a range of related desymmetrization protocols have been developed recently (Figure 1b).^23–38^ For instance, Xiao’s group and Meng’s team separately developed cobalt-catalyzed coupling strategies to synthesize axially chiral aldehydes via biaryl dialdehyde desymmetrization.^31,32^ However, most one-step methods for constructing molecules with both axial and multiple central chiralities afford only modest stereoselectivity, rendering their simultaneous precise stereocontrol a significant challenge.^35–40^

Asymmetric carbonyl allylation provides direct access to homoallylic alcohols, a privileged scaffold in synthesis and drug discovery.^41–60^ Nickel-catalyzed variants are underexplored relative to established precious metal and chromium systems, despite nickel’s advantages in cost, toxicity, and abundance,^61,62^ yet suitable chiral ligands for this multistereocenter regulation remain scarce.^63,64^ Herein, we report an adamantyl-BOX ligand for dual photoredox/nickel-catalyzed asymmetric desymmetrization of biaryl dialdehydes (Figure 1c). This protocol delivers axially chiral aldehydes bearing two vicinal stereocenters with up to 20:1 dr and 99% ee. The origin of this outstanding performance lies in the unique second-layer chiral cavity assembled by flexible adamantylphenyl side arms, which exerts precise stereocontrol via the synergy between cavity confinement and distal substituent effects, independent of direct steric contact with the active site. This work provides a valuable design framework for remote stereocontrol, supported by combined crystallographic and computational studies.

## Results and Discussion

Following our initial ligand screening (detailed below), we identified the bisoxazoline (BOX) ligand **L9**—functionalized with sterically bulky adamantylphenyl side arms—as a promising candidate for constructing an efficient catalytic system that enables precise stereocontrol. With **L9** as the chiral ligand, we chose the coupling between biaryl dialdehyde **1a** and 4-methoxycinnamyl acetate **2a** as the model reaction, followed by systematic optimization (Table 1). Under optimized conditions, the desired product **3a** was obtained in 78% yield with 12:1 dr and 93% ee, using 4CzIPN as the photocatalyst, NiCl_2_ as the metal catalyst, Hantzsch ester (HE) as the reductant, DIPEA as the base, DMF as the solvent, and irradiation with a 5 W 450 nm LED at room temperature for 12 h (entry 1). The screening of nickel sources proved to be critical for the reaction efficiency. While replacing NiCl_2_ with NiBr_2_ maintained high enantioselectivity and diastereoselectivity, a slight decrease in yield was observed (entry 2). In contrast, the use of Ni­(acac)_2_ resulted in a substantial deterioration in both yield and stereoselectivity (entry 3). Further screening of photocatalysts revealed that although Ir­(ppy)_2_(dtbbpy)­PF_6_ (**Ir-1**), Ir­[dF­(CF_3_)­ppy]_2_(dtbbpy)­PF_6_ (**Ir-2**), and Ru­(bpy)_3_(PF_6_)_2_ **Ru-1** were capable of initiating the reaction, the desired product **3a** was formed in trace quantities (entries 4–6). Gratifyingly, the substitution of DMF with THF further enhanced both the yield and stereoselectivity, delivering the product in 83% yield with improved stereocontrol (15:1 dr, 95% ee) (entry 7). Despite yielding comparable stereoselectivity (12:1 dr, 95% ee), replacing DIPEA with triethylamine led to a substantial decrease in yield (50%, entry 8). A series of control experiments—omitting the photocatalyst, NiCl_2_, HE, DIPEA, or light—all failed to yield any product **3a**, proving each component is indispensable (entries 9–10).

**Table 1:**
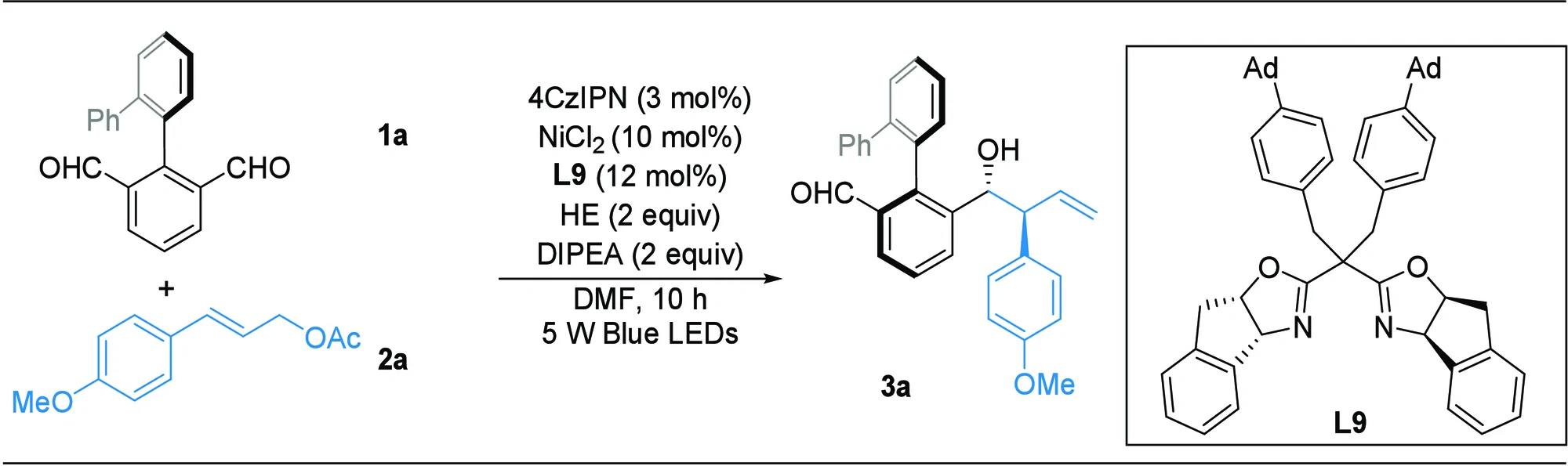
Reaction Optimization^a^

Entry	Variation from standard conditions	**3a** (%)^b^	dr^d^	Ee (%)^e^
1	As shown	78	12:1	93
2	NiBr_2_ instead of NiCl_2_	69	12:1	93
3	Ni(acac)_2_ instead of NiCl_2_	34	4:1	59
4	**Ir-1** instead of 4CzIPN	Trace	-	-
5	**Ir-2** instead of 4CzIPN	Trace	-	-
6	**Ru-1** instead of 4CzIPN	Trace	-	-
7	THF instead of DMF	85(83)^c^	15:1	95
8	Et_3_N instead of DIPEA	50	12:1	95
9	No 4CzIPN, or no NiCl_2_	0	-	-
10	No HE, no DIPEA, or no light	0	-	-

a General reaction conditions: **1a** (0.2 mmol, 57.3 mg), **2a** (0.4 mmol, 82.5 mg), reaction time: 12 h reaction temperature: 10 °C.

b Determined by ^1^H NMR analysis of the reaction mixture using 1,3,5-trimethoxybenzene as an internal standard.

c Isolated yield.

d Determined by ^1^H NMR spectroscopy.

e Determined by chiral HPLC analysis. (See the Supporting Information for the assignment of side products regarding dr.)

To elucidate the ligand structure–activity relationship and the origin of the superior performance of **L9**, we synthesized a series of BOX ligands bearing varied side arm substituents (**L1**–**L9**) and evaluated their performance in the model reaction under optimized conditions (Figure 2a). The simple methyl-substituted ligand **L1** afforded product **3a** with only moderate stereoselectivity (4:1 dr, 66% ee), while cyclopropyl (**L2**) or benzyl (**L3**) substituents gave marginally improved enantioselectivity (62% ee for **L2**, 70% ee for **L3**); however, excessively bulky naphthyl (**L4**) and anthracenyl (**L5**) substituents completely suppressed reactivity, defining a steric threshold for productive catalysis. Gratifyingly, increasing the steric bulk of the benzyl side arm markedly boosted stereoselectivity: para-*tert*-butylbenzyl-substituted **L6** delivered **3a** with 10:1 dr and 76% ee, while 3,5-di-*tert*-butylbenzyl-substituted **L7** further raised enantioselectivity to 80% **ee**. In contrast, the electron-withdrawing trifluoromethyl group in **L8** strongly inhibited reactivity, underscoring the critical impact of side arm electronic properties on catalysis. Consistent with the condition optimization results, ligand **L9** delivered the best performance, furnishing **3a** in 83% yield with excellent stereoselectivity (12:1 dr, 95% ee).

**Figure 2:**
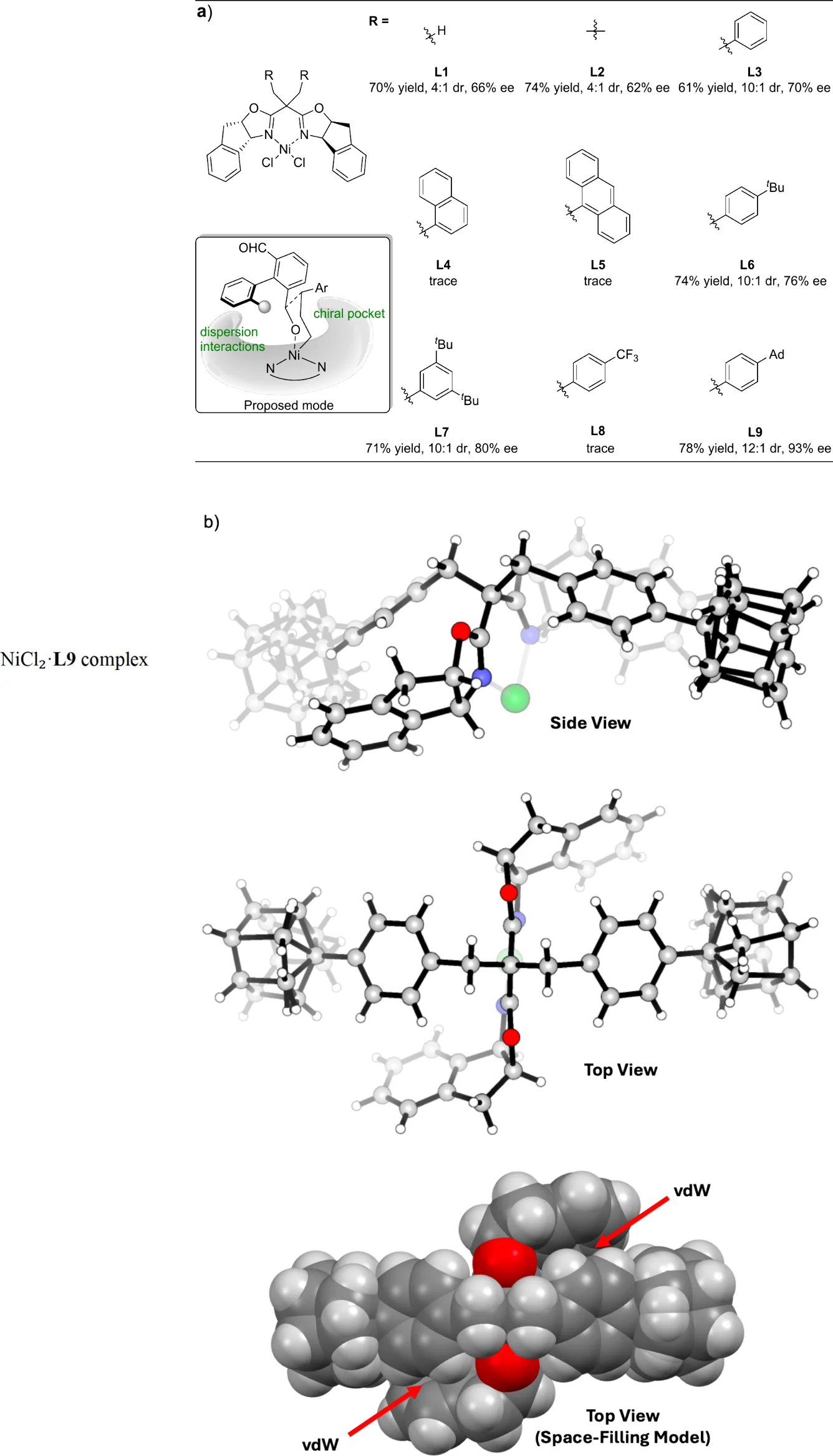
a) Evaluation of ligand effect. b) Single crystal structure of the NiCl_2_·**L9** complex showing encapsulation of the nickel center. Solvent molecules, counterions, and water molecules are omitted for clarity. The adamantane groups are disordered in the single crystal structure. The complete crystal structure is available in the ESI.

To elucidate the coordination mode of optimal ligand **L9** and its stereocontrol origin, we grew single crystals of the NiCl_2_·**L9** complex for X-ray diffraction analysis (Figure 2b). The crystal structure reveals that upon nickel coordination, the two bulky adamantylbenzyl side arms of **L9** fold inward and assemble a well-defined, sterically confined chiral pocket with the Indane-derived backbone. Notably, the adamantyl groups lie at the distal end of the side arms (far from the Ni coordination sphere); their steric bulk drives side arm folding and defines the cavity boundary, constituting the structural origin of the distal substituent effect. This folded conformation encapsulates the metal center, constrains substrate binding orientation, and enforces precise stereocontrol. Weak intramolecular interactions between side arm benzylic moieties and the Indane backbone further rigidify the complex and reinforce the active-site chiral environment. Collectively, these crystallographic data confirm that the synergy between the confined chiral cavity and distal substituent effects is the dominant stereocontrol factor. This second-shell chiral cavity design effectively mitigates inherent match/mismatch effects, delivering exceptional stereoselectivity for multistereocenter construction that is not readily achievable with conventional catalytic systems.

Under the optimized conditions, we began by investigating the substrate scope of allylic acetates. As shown in Table 2, a variety of para-substituted electron-donating groups, such as acetoxyl (**3b**), diphenylamino (**3c**), and *tert*-butyl (**3d**), were compatible with the reaction protocol. In nearly all cases, these substrates were efficiently transformed into the desired products with good to high yields (82–85%) and nice stereocontrol (>20:1 dr, 92–95% ee). Furthermore, the reaction proved robust toward allylic substrates bearing benzyl alcohol moieties (**3e**), furnishing the desired axially chiral compounds smoothly. Notably, the protocol proved compatible with a range of halogen substituents (**3f**-**3i**). Including even the strongly electron-withdrawing fluorine and the highly reactive iodine, all were converted into the corresponding products. Moreover, allylic acetates bearing para-substituted electron-withdrawing groups (such as ester (**3j**), trifluoromethoxy (**3k**), and ketone (**3l**)) were well-tolerated in the desymmetrization, furnishing the corresponding asymmetric products with enantiomeric excess (ee) values ranging from 92% to 95%. Allylic precursors bearing cyano groups at the para-, meta-, or ortho-positions of the benzene ring also served as suitable coupling partners, affording the corresponding homoallylic alcohol products (**3m**-**3o**) in good yields (72%–83%) with nice enantioselectivity (87% ee-99% ee). The 2-phenoxy-substituted allylic acetates also proved to be a suitable substrate, affording the chiral aldehyde **3p** in 90% yield with excellent enantioselectivity. Substrates bearing thioether (**3q**) and mesylate (**3r**) substituents were also viable under the standard conditions, undergoing smooth conversion to the target molecules in good efficiency. This result underscores the broad functional group tolerance of this catalytic system. Delightfully, the large steric hindrance of the multisubstituted allylic precursor performed effectively in the coupling, furnishing the desired product **3s** in 85% yield with excellent stereocontrol (>20:1 dr, 98% ee). Next, high enantioselectivity (90–99% ee) was consistently achieved for substrates featuring naphthalene (**3t**), 1,4-benzodioxane (**3u**), and piperonyl (**3v**) rings, underscoring the generality of the asymmetric protocol. The electron-deficient pyridine-derived precursor (**3w**) was converted with excellent stereocontrol (>20:1 dr, 99% ee), albeit in a moderate 51% yield. Meanwhile, we determined the absolute configuration of product **3b** by X-ray crystallographic analysis, and those of the remaining products were assigned by analogy.

**Table 2:**
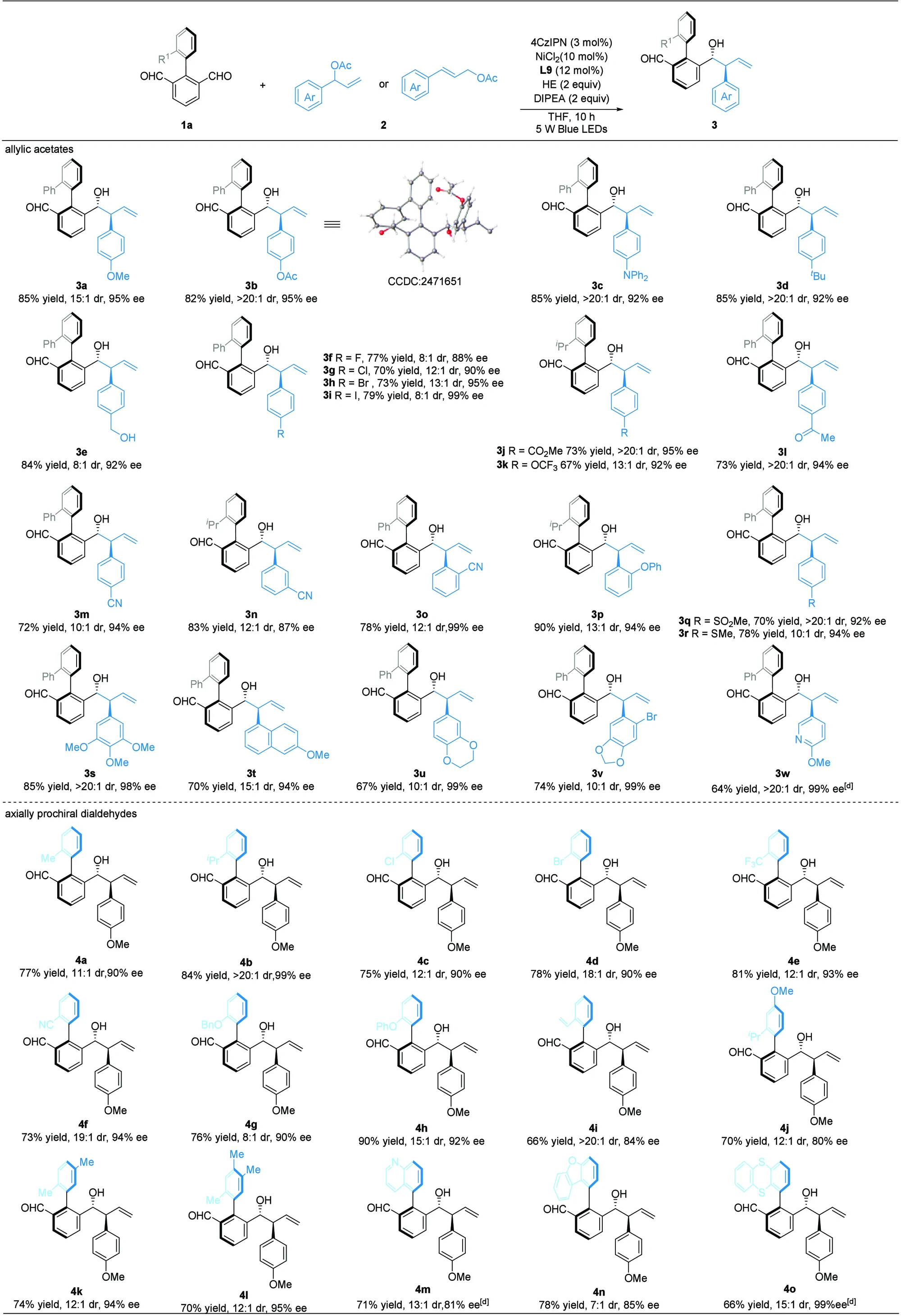
Scope of Reactions Using **L9**-**Ni** as Catalyst^a^ ^,^ ^b^ ^,^ ^c^

a General reaction condition: aldehydes **1** (0.2 mmol), **2** (0.4 mmol), 4CzIPN (0.006 mmol, 4.7 mg), NiCl_2_ (0.02 mmol, 2.6 mg), **L9** (0.024 mmol, 18.7 mg), HE (0.4 mmol, 101.3 mg), DIPEA (0.4 mmol, 51.7 mg),THF (2.0 mL), 450 nm LEDs, 10 °C, 10 h.

b Isolated yield.

c dr was determined by ^1^H NMR analysis, *ee* was determined by chiral HPLC analysis. [d] HE (0.3 mmol, 76.0 mg), 5 °C, 7 h.

Next, we investigated the scope of different substituted biphenyl bialdehydes for the desymmetrization reaction. The replacement of the phenyl group with a methyl (**4a**) or isopropyl (**4b**) substituent at the 2-position proceeded effectively, affording the respective products in 77% yield (11:1 dr, 90% ee) and 84% yield (>20:1 dr, 99% ee); this notable enhancement in stereoselectivity with increased steric bulk underscores the crucial role of steric hindrance at the 2-position. The efficient coupling of 2-chloro- and 2-bromo-substituted dialdehydes with **2a** proceeded to deliver the axially chiral products (**4c**, **4d)** with good to excellent diastereoselectivity (12:1 and 18:1 dr, respectively), despite the relatively limited steric bulk of the halogen substituents. Good yields were also obtained for substrates featuring strong electron-withdrawing groups (trifluoromethyl and cyano) at the 2-position, as the system proved highly compatible with these substituents and smoothly delivered the expected products **4e**–**4f**.

Substitution of the phenyl ring with benzyloxy, phenoxy, or vinyl groups at the 2-position proceeded effectively, delivering products **4g**–**4i** in good yields. Polysubstituted biaryl dialdehydes likewise served as substrates, exhibiting excellent performance in the catalytic system to afford products **4j**–**4l** without compromise to either yield or stereoselectivity (≥12:1 dr, up to 96% ee). Surprisingly, several (hetero)­aryl-substituted derivatives were fully compatible, such as quinoline, thianthrene, and dibenzofuran, yielding **4m**–**4o** in reasonable yields with high stereoselectivities, which are less explored in previous studies.

Furthermore, a gram-scale reaction of **1c** with **2a** under the standard conditions afforded the desired products in good yield with excellent diastereo- and enantioselectivity (Figure 3a). To further demonstrate the synthetic utility of this axially chiral scaffold, we performed a series of functional group transformations using **4b** as a model substrate (Figure 3b). Reduction of the aldehyde group in **4b** using sodium borohydride proceeded smoothly to furnish axially chiral benzyl alcohol **5a** in 93% yield with the complete retention of enantiomeric purity. Oxidation of the hydroxyl group in **4b** with Dess-Martin periodinane (DMP) provided ketone **5b** in 86% yield with maintained enantiocontrol (>20:1 dr, 97% ee). Furthermore, a Knoevenagel condensation between **4b** and diethyl malonate delivered product **5c** in a 76% yield with an excellent enantiomeric excess (99% ee). Notably, subjecting **4b** to Seyferth-Gilbert homologation conditions using dimethyl (1-diazo-2-oxopropyl)­phosphonate generated the alkyne **5d** in 81% yield with 99% ee, demonstrating the robustness of the chiral axis under these transformation conditions.

**Figure 3:**
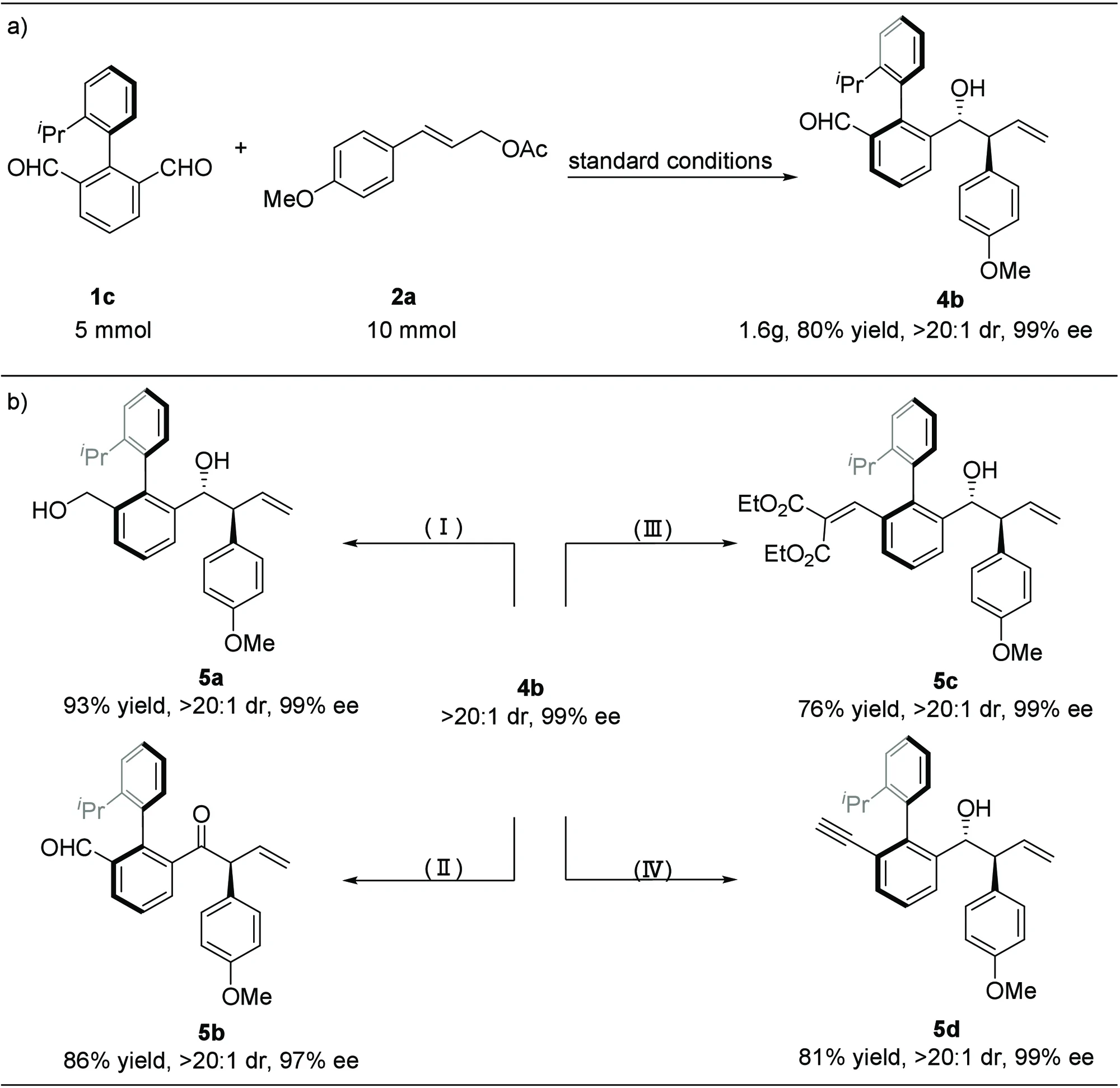
a) Gram-scale experiment and follow-up chemistry. b) Reaction conditions: (i) NaBH_4_ and THF/MeOH, 0 °C. (ii) DMP and THF, rt. (iii) Methylamine hydrochloride, diethyl malonate, and MeOH, rt. (iv) P-(1-diazo-2-oxopropyl)-di-Methylester, K_2_CO_3_, MeOH, rt. (see the Supporting Information for detailed procedures).

To elucidate the mechanism of this transformation, we conducted a series of experiments. First, a radical inhibition experiment was performed by adding 3.0 equiv of TEMPO (2,2,6,6-tetramethylpiperidin-1-oxyl) under the standard conditions. The allylation product was still obtained in 56% yield, and no TEMPO-adduct was detected (Figure 4a). Furthermore, considering that Ni^0^ species can potentially undergo oxidative addition with the substrate **2a** to form the allyl nickel­(II) complex,^65–67^ a control experiment employing Ni^0^ as the catalyst was carried out (Figure 4b and 4c). This reaction delivered the desired product in 44% isolated yield, supporting the involvement of such a catalytic cycle. Furthermore, Stern–Volmer fluorescence quenching experiments revealed that the emission intensity of excited 4CzIPN was significantly quenched in the presence of HE, consistent with rapid single electron transfer between excited 4CzIPN and HE in the photoredox catalytic cycle. (For details see Supporting Information Figure S1.) To understand the stereochemical control of this reaction, we first performed a dynamic resolution experiment (Figure 4d). Under the standard conditions, the racemic product **4e** was recovered unchanged, and no overallylated product was detected. To rule out accidental results, we further selected rac-**4b** and **4m** for validation. In both cases, the products were recovered completely, maintaining their racemic configurations, and no over-reaction products were observed (see Supporting Information). This observation indicates that the enantioselectivity arises solely from desymmetrization. Subsequently, we investigated the nonlinear effect, a general method for probing the structural components of catalysts in asymmetric catalysis. In this study, a linear correlation was observed between the enantiomeric excess of the chiral BOX ligand and that of the allylation product **3a** (see Supporting Information Figure S2).

**Figure 4:**
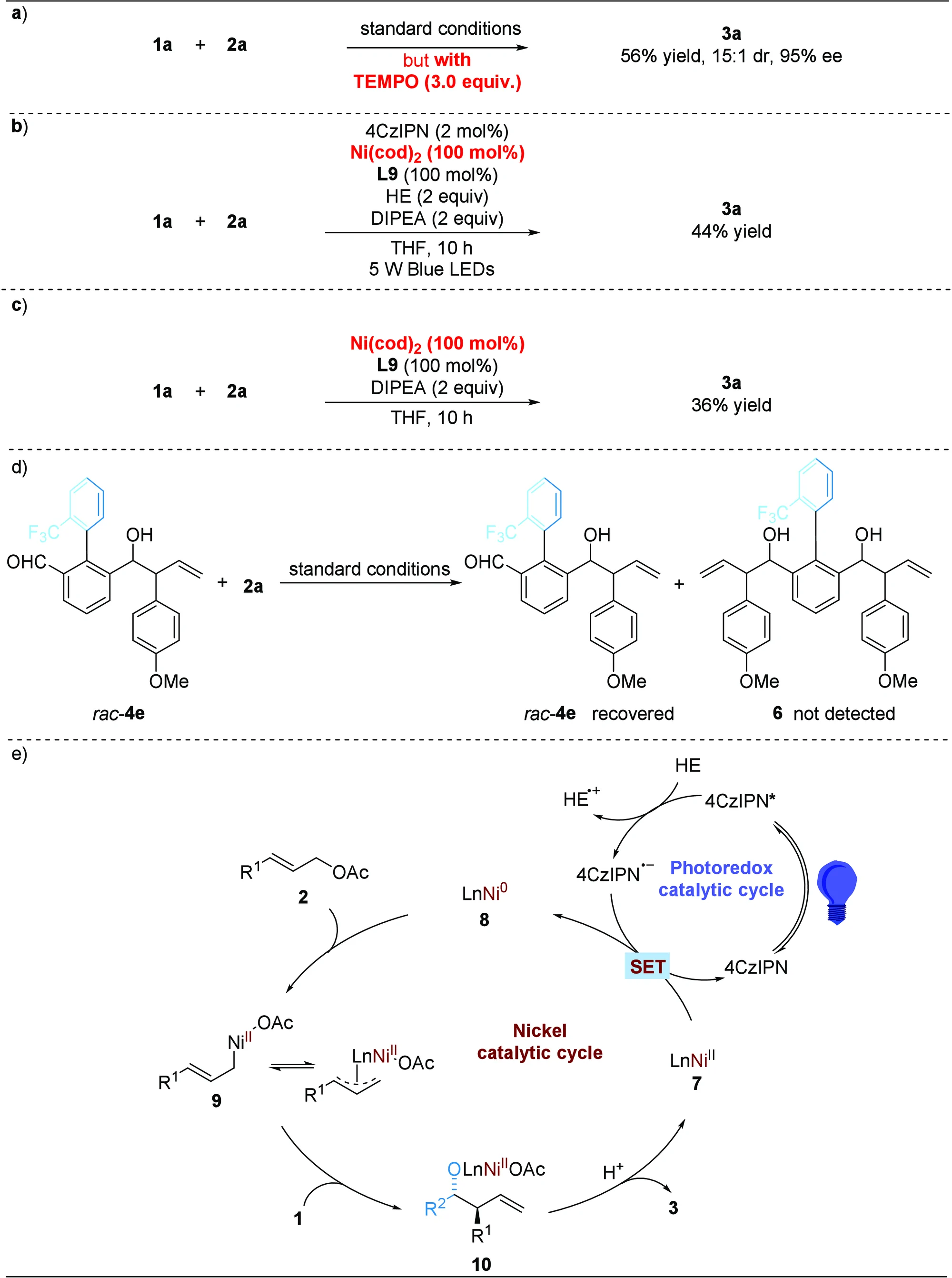
a) Reaction with TEMPO as inhibitor; b) Catalysis with Ni­(cod)_2_ under photoredox conditions; c) Ni­(cod)_2_ as catalyst; d) Kinetic Resolution experiment; e) Proposed photoredox and nickel catalytic cycles.

Guided by the above experimental results and relevant literature precedents,^70–75^ a plausible catalytic mechanism involving both nickel and photoredox cycles is proposed in Figure 4e. Under irradiation with blue LEDs, the photoexcited 4CzIPN is reduced by HE [E_1/2_^ox^ (HE/HE^•+^) = 0.51 V vs Fc+/Fc in MeCN][Bibr ref68] via a single-electron transfer (SET) to generate 4CzIPN^•–^ [E_1/2_^red^ (4CzIPN/4CzIPN^•–^) = −1.21 V vs SCE in MeCN].^69^ This radical anion subsequently reduces the Ni^II^ precatalyst to a Ni^0^ species through two sequential SET events.^62,70–77^ The Ni^0^ species undergoes oxidative addition with allylic acetates **2** to generate the nucleophilic allyl-Ni^II^ intermediate (**9**). Intermediate (**9**) is poised to attack the aldehyde, yielding a nickel alkoxide species (**10**). Finally, protonation of this intermediate liberates the product **3** and regenerates the Ni^II^ species, thereby completing the catalytic cycle. However, the involvement of a nucleophilic allyl-Ni^I^ intermediate that adds to the aldehyde cannot be excluded.

To investigate the role of the chiral BOX ligands in the stereochemical control during the reaction, density functional theory (DFT) calculations were performed on four stereodetermining transition states (Figure 5). Given the potential complexity of photoredox/nickel dual catalysis, including the multiple accessible oxidation and coordination states of the nickel center, we focused our attention here on one plausible mechanistic scenario. In this calculation, **1a** and **2a** were used as the model substrates. For the chiral ligand, we simplified the ligand by replacing the adamantane-substituted benzyl group with a methyl group, corresponding to **L1**. While ligand L1 was less effective than **L9**, it still afforded the product with appreciable enantioselectivity and diastereoselectivity under the optimized conditions (4:1 dr, 66% ee, Figure 2a).

**Figure 5:**
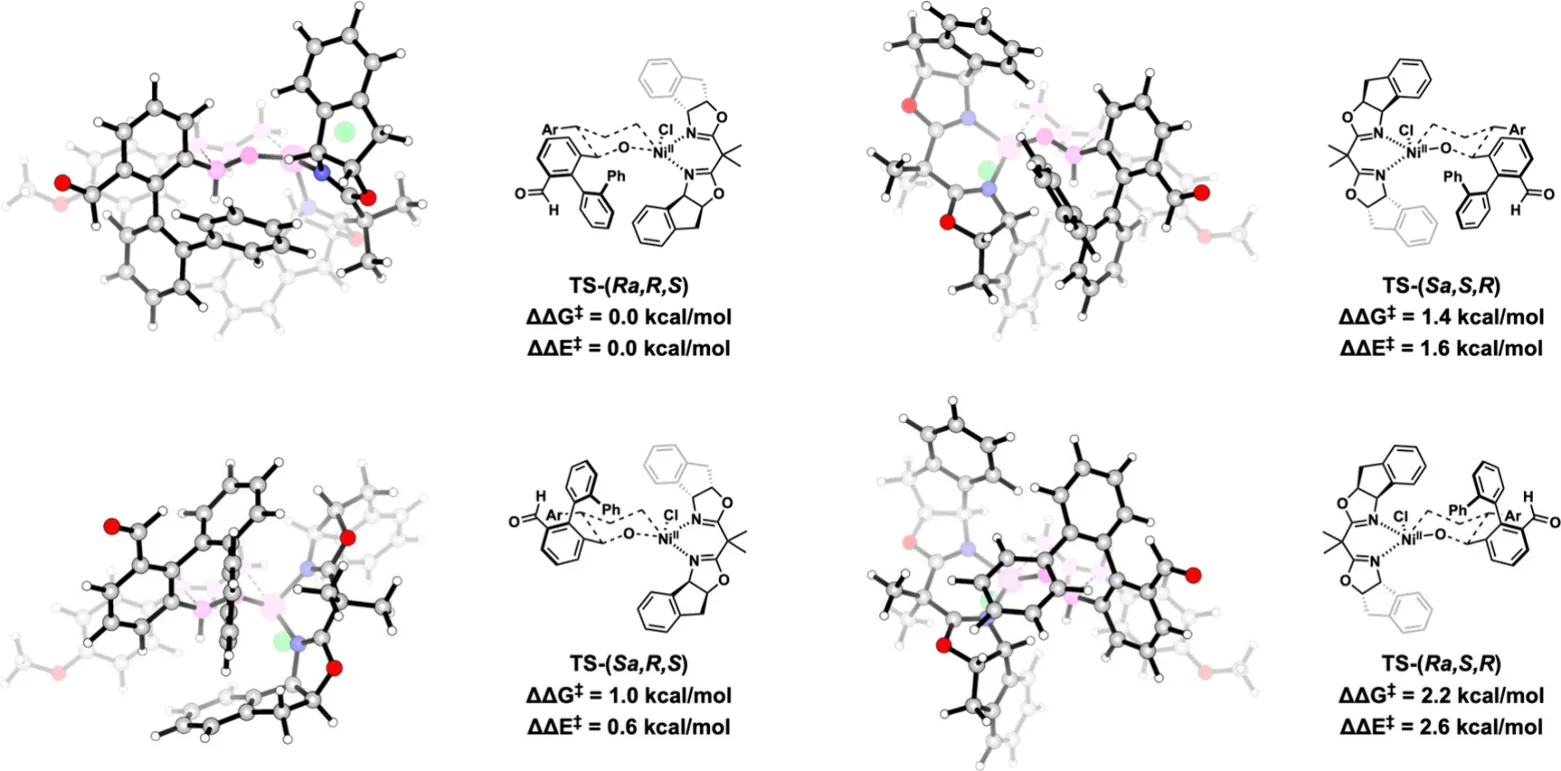
Structures of the four stereodetermining transition states. Relative Gibbs free energies (ΔΔG^‡^) and electronic energies (ΔΔE^‡^) were calculated at the PWPB95-D4/def2-TZVPP/SMD­(DMF)//PBE-D3­(BJ)/def2-SVP level of theory at 298.15 K. Color code for 3D structures: H, white; C, gray; O, red; N, blue; Cl, green. Atoms on the chairlike six-membered ring in the Zimmerman-Traxler transition states are highlighted in pink for clarity.

We first performed geometry optimization and a conformational search of the transition state **TS-(** ***Ra,R,S*** **)** at the PBE-D3­(BJ)/def2-SVP level of theory, which leads to the major product confirmed by the single-crystal structure.^78–83^ High-accuracy single-point energies were subsequently calculated using the double-hybrid functional (with D4 dispersion correction) PWPB95-D4 with the def2-TZVPP basis set and SMD solvation model in DMF.^84–87^ As shown in Figure 5 (top left), **TS-(** ***Ra,R,S*** **)** follows a typical chairlike six-membered Zimmerman-Traxler transition state model, in which both the o-terphenyl group on the aldehyde moiety and the aryl group on the allyl moiety occupy equatorial positions. To minimize steric repulsion, the biphenyl unit in the ortho-terphenyl group is oriented outward from the six-membered ring. The nickel center adopts a slightly distorted square-pyramidal coordination geometry.

Noncovalent interaction (NCI) analysis reveals multiple weak interactions between the chiral ligand and the substrate in **TS-(** ***Ra,R,S*** **)**. Specifically, C–H bonds on the indene and oxazoline moieties of the chiral ligand engage in van der Waals (vdW) or C–H···π interactions with the aryl group of the allyl moiety and the o-terphenyl moiety of the substrate. C–H···O interactions are also observed between the ligand and the aldehyde oxygen (see Supporting Information Figure S3 for a visualization of these interactions).^88–90^ In contrast, for the competing transition state **TS-(** ***Sa,S,R*** **)** that leads to the enantiomeric product, although a notable π–π interaction is observed between the indene moiety of the ligand and the biphenyl group of the substrate, other weak interactions are largely absent (top right, Figure S3). This may partially account for its higher energy (ΔΔG‡ = 1.4 kcal/mol, ca. 83% ee), which is qualitatively consistent with the experimentally observed ee of 66% (ca. ΔΔG‡ = 0.9 kcal/mol).

We then inverted the axial chirality of **TS-(** ***Ra,R,S*** **)** to obtain transition state **TS-(** ***Sa,R,S*** **)**, which leads to the diastereomeric product (bottom left, Figure 5). This transition state was found to be higher in energy by 1.0 kcal/mol (ca. 5:1 dr). Conformational analysis reveals that in this stable conformer of **TS-(** ***Sa,R,S*** **)**, the biphenyl unit of the o-terphenyl group also flips upward above the six-membered ring after the inversion of axial chirality while still being oriented outward from the ring. This observation is consistent with our expectations: if the biphenyl unit were to point inward upon axial inversion, then it would experience significant steric repulsion with the allyl moiety of the substrate. Finally, the fourth transition state **TS-(** ***Ra,S,R*** **)** was located by inverting the axial chirality of **TS-(** ***Sa,S,R*** **)**. This transition state exhibited a significantly higher energy by 2.2 kcal/mol, which is likely due to the lack of stabilizing noncovalent interactions between the ligand and substrate (bottom right, Figure S3).

## Conclusion

In summary, we have developed a dual photoredox/nickel-catalyzed asymmetric desymmetrization of biaryl dialdehydes, filling a critical synthetic gap: one-step construction of one axial chirality and two contiguous central chiralities. A rationally designed adamantylphenyl-substituted BOX ligand, which assembles a confined second-shell chiral cavity upon coordination, enables excellent stereocontrol via the synergy of cavity confinement and distal-substituent effects. The protocol affords homoallylic alcohol products with up to >20:1 dr and 99% ee; gram-scale synthesis and diverse derivatizations validate its synthetic utility. Density functional theory calculations corroborate that a network of noncovalent interactions within the chiral pocket underpins the outstanding stereoselectivity. This work provides both an efficient route to multistereogenic axially chiral biaryls and a second-shell cavity engineering strategy for ligand design in asymmetric catalysis.

## Supplementary Material



## Notes

Experimental details, characterization data of compounds, NMR spectra. Deposition number 2471651 (for **3b**) and 2471652 (for **L9**-NiCl_2_) (PDF)

## Notes

Deposition numbers 2471651 and 2471652 contain the supplementary crystallographic data for this paper. These data can be obtained free of charge via the joint Cambridge Crystallographic Data Centre (CCDC) and Fachinformationszentrum Karlsruhe Access Structures service.
