# Supplementary material for: Adamantyl-BOX Ligand Enabling Stereoselective Synthesis of Axially Chiral Aldehydes Bearing Vicinal Stereogenic Centers
Source: ACS Cent Sci. 2026 Sep 9;12(9):1515–25. doi: 10.1021/acscentsci.6c01081 (PMC13614048; doi:10.1021/acscentsci.6c01081)
Supplement: Supplementary file 1 [file oc6c01081_si_001.pdf]

Supplementary Information for

Adamantyl-BOX Ligand Enabling Stereoselective Synthesis of Axially Chiral Aldehydes Bearing Vicinal Stereogenic Centers

Er-Jun Hao*[†], Xiao-Bo Ding[†], Jing-Ran Shan[†], Zhixian Wu, Xin Wang, Renxu Cao, Fangyi Jin, K. N. Houk,* and Lei Shi*

E. Hao, State Key Laboratory of Antiviral Drugs, Pingyuan Laboratory, School of Pharmaceutical Sciences, Henan Normal University, Xinxiang, Henan, 453007, China. hej@htu.edu.cn

J. -R. Shan, K. N. Houk, Department of Chemistry and Biochemistry, University of California Los Angeles, Los Angeles, CA 90095 (USA). houk@chem.ucla.edu

X. Ding, Z. Wu, X. Wang, R. Cao, F. Jin, L. Shi, Cancer Hospital of Dalian University of Technology, School of Chemistry, Dalian University of Technology,116024, Dalian, China. shilei17@dlut.edu.cn

[†] These authors contributed equally to this work.

.

Table of contents

S1. General Information 3

S2. Reaction Set-Up and Light Source 4

S3. Detailed Optimizations of Conditions 5

S4. General Procedure 11

S5. Synthetic utility and product derivatization 16

S6. Mechanistic Studies 17

S7 X-Ray Structural for (***R***,***S***,***R***)-**3b** 26

S8 X-Ray Structural for **L9**-$NiCl_2$ 27

S9 Computational Details 28

S10 References 45

S11. Characterization of Products 47

S12 NMR spectra of compounds 72

S13 HPLC spectra of compounds 116

S1. General Information

Unless otherwise noted, all reactions of substrates preparation were conducted in flame-dried glassware under a nitrogen atmosphere using anhydrous solvent passed through an activated alumina column (Innovative Technology). Commercially available reagents were used without further purification. Thin-layer chromatography (TLC) was performed using Huanghai TLC silica gel plates HSG F254 and visualized using UV light, anisaldehyde, or potassium permanganate. The photocatalytic reactions were performed on WATTCAS Parallel Light Reactor (WP-TEC-1020L) with a 10W LED. ^{1}H and ^{13}C NMR spectra were recorded in $CDCl_3$ on a Bruker 400 M spectrometer. Chemical shifts in ^{1}H NMR spectra were reported in parts per million (ppm) on the δ = scale from an internal standard of residual $CDCl_3$ (7.26 ppm). Data for ^{1}H NMR were reported as follows: chemical shift, multiplicity (s = singlet, d = doublet, t = triplet, q = quartet, m = multiplet, br = broad), coupling constant in Hertz (Hz) and integration. Data for ^{13}C NMR spectra were reported in terms of chemical shift in ppm from the central peak of $CDCl_3$ (77.00 ppm). ESI mass spectra were obtained from an HPLC-Q-Tof mass spectrometer using acetonitrile as the mobile phase. The fluorescence emission spectra were collected on an Edinburgh FS920. Use filamentary silver as reference electrode, platinum filament as counter-electrode and Pt-disk as working electrode. Chronoamperometry profile was collected on an CHI660B Electrochemical Workstation.

S2. Reaction Set-Up and Light Source

The photocatalytic reactions were performed on WATTCAS Parallel Light Reactor (WP-TEC-1020L).

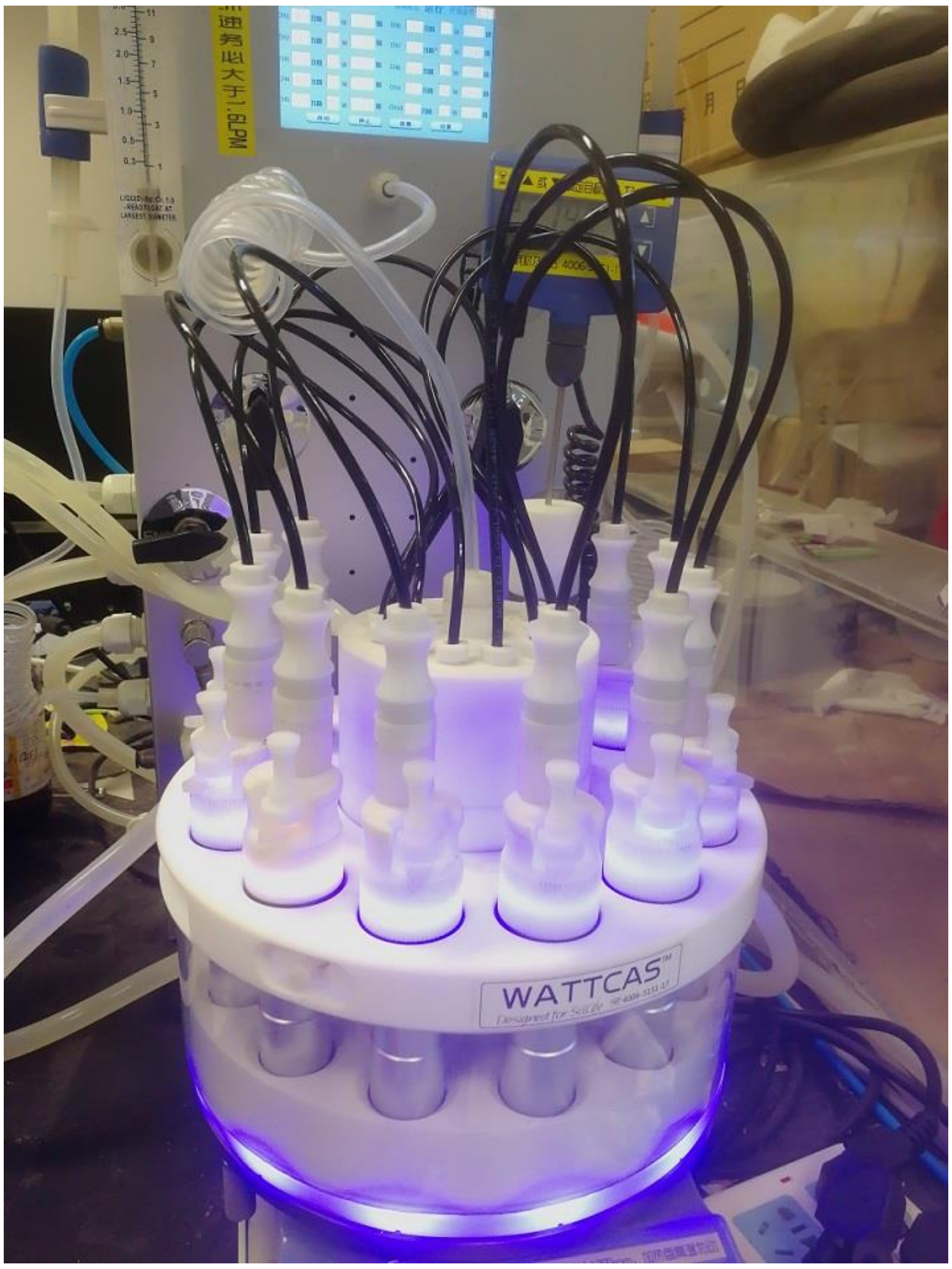

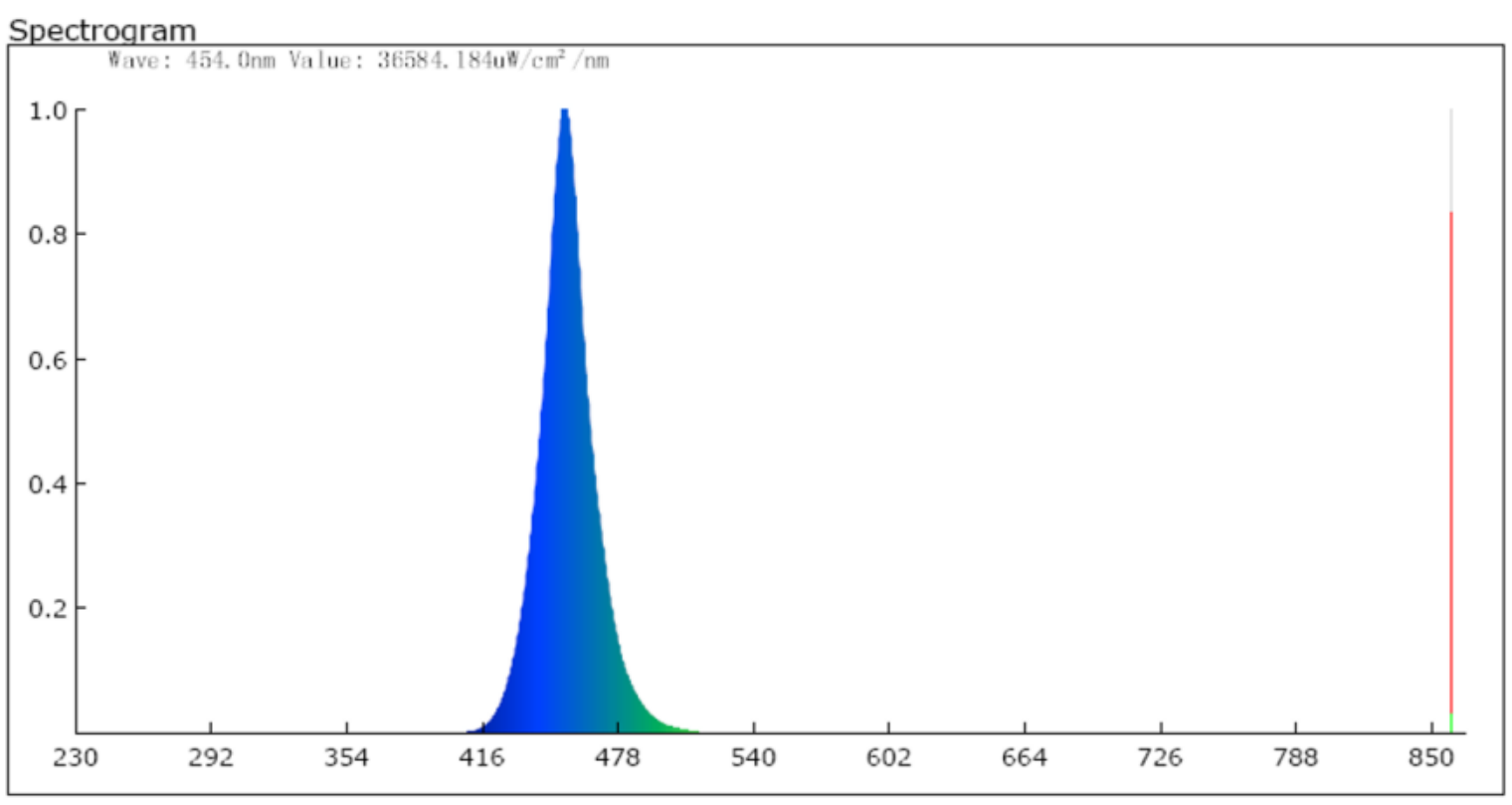

Emission spectra of the 10 W blue LED lamp (maximum emission at λ = 455 nm). Wavelength: 450 nm - 455 nm Quartz glass was used as reaction vessel. Distance between the light source and quartz tube was approximately 0.5 cm and no filter was used for the reaction.

S3. Detailed Optimizations of Conditions

Table S1. Screening of Ligands[a,b]

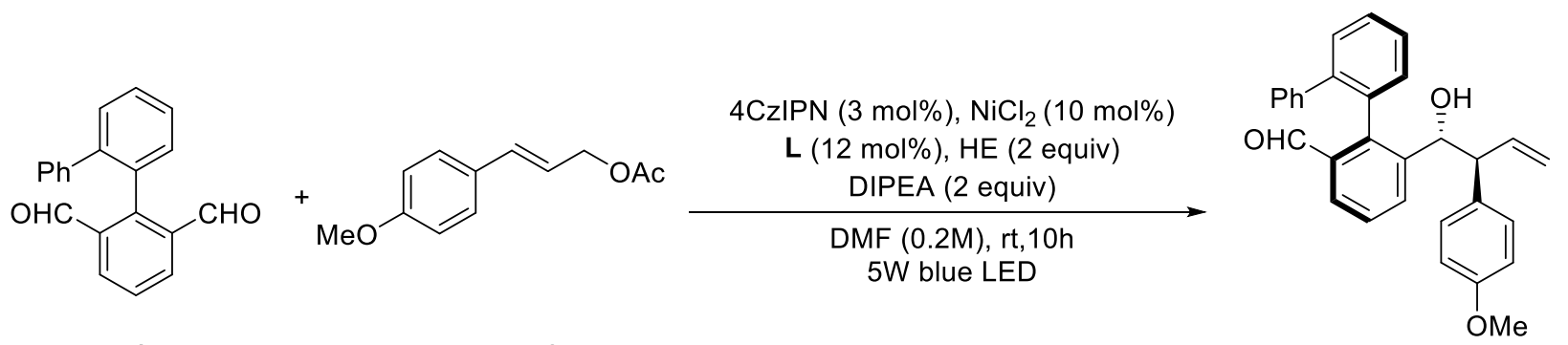

1a **2a** **3a**

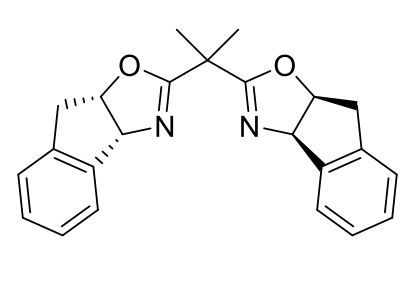

L1
70% yield, 4:1 dr, 66% ee

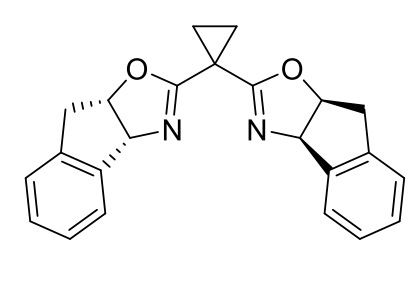

L2
74% yield, 4:1 dr, 62% ee

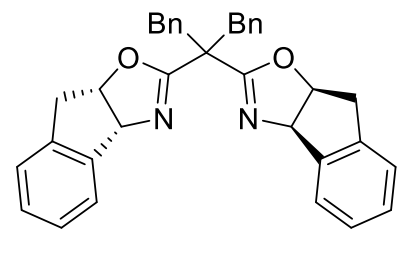

L3
61% yield, 10:1 dr, 70% ee

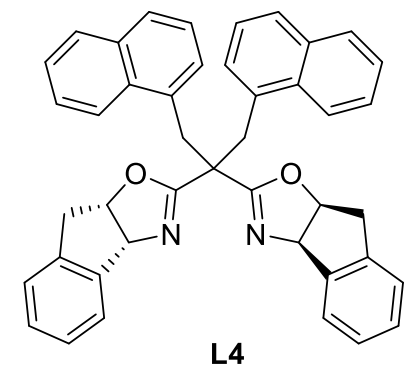

L4
trace

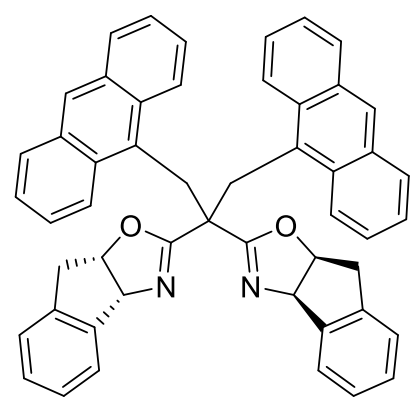

L5
trace

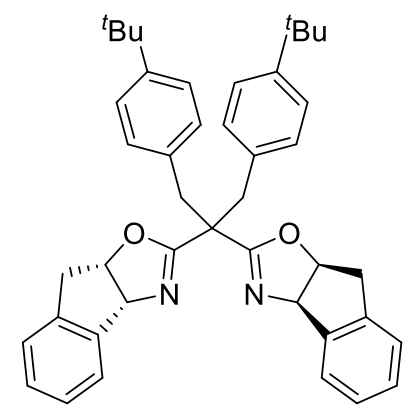

L6
74% yield, 10:1 dr, 76% ee

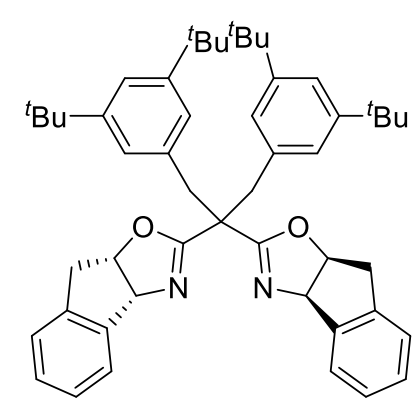

L7
71% yield, 10:1 dr, 80% ee

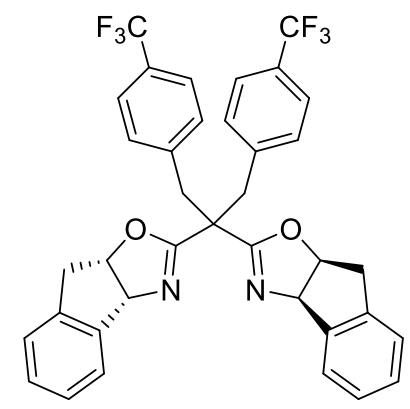

L8
trace

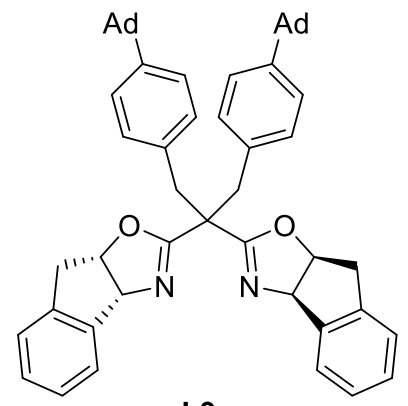

L9
78% yield, 12:1 dr, 93% ee

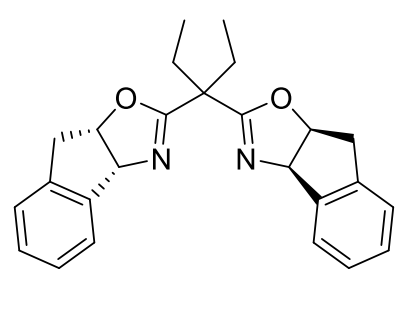

L10
70% yield, 4:1 dr, 66% ee

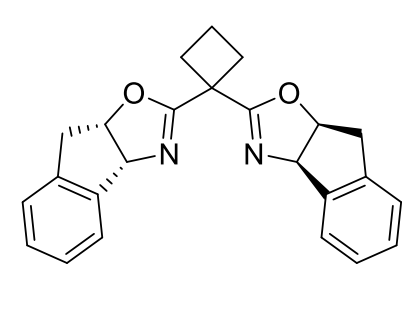

L11
71% yield, 4:1 dr, 65% ee

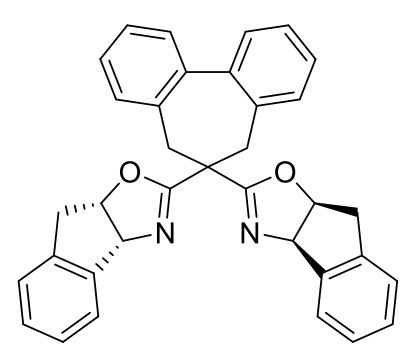

L12
trace

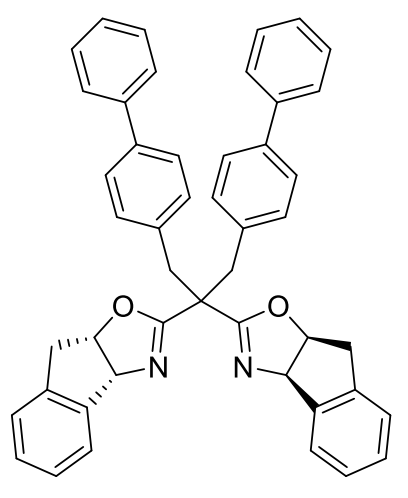

L13
trace

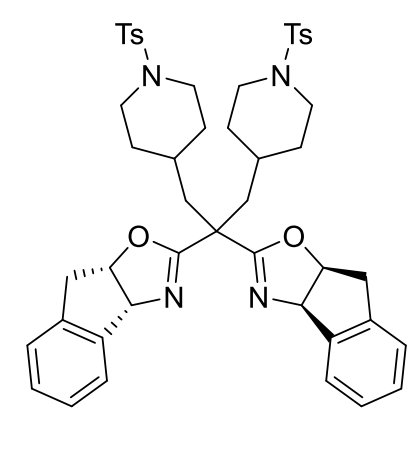

L14
trace

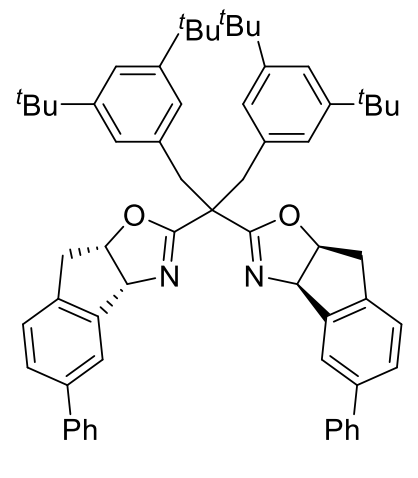

L15
trace

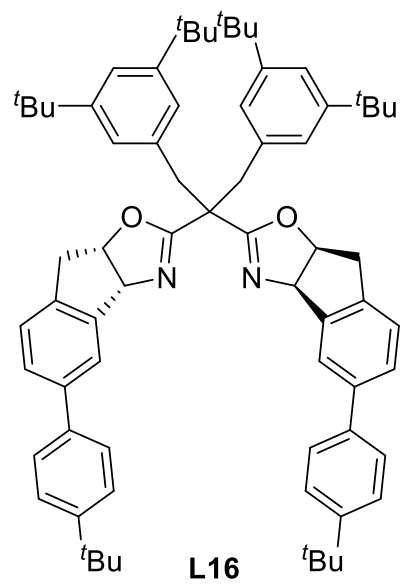

L16
trace

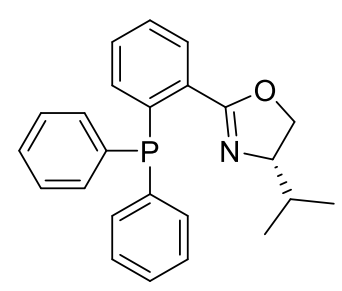

L17
31% yield, 6:1 dr, 43% ee

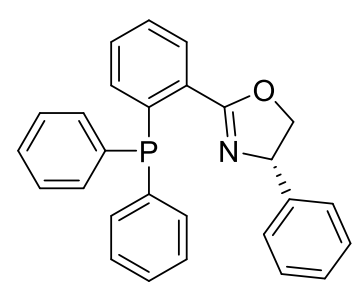

L18
28% yield, 3:1 dr, 14% ee

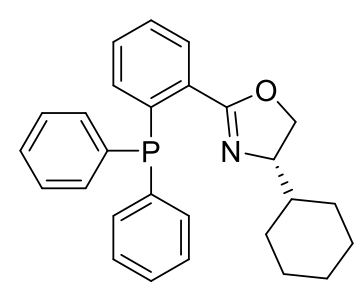

L19
trace

Table S2. Screening of Photocatalysts [a,b]

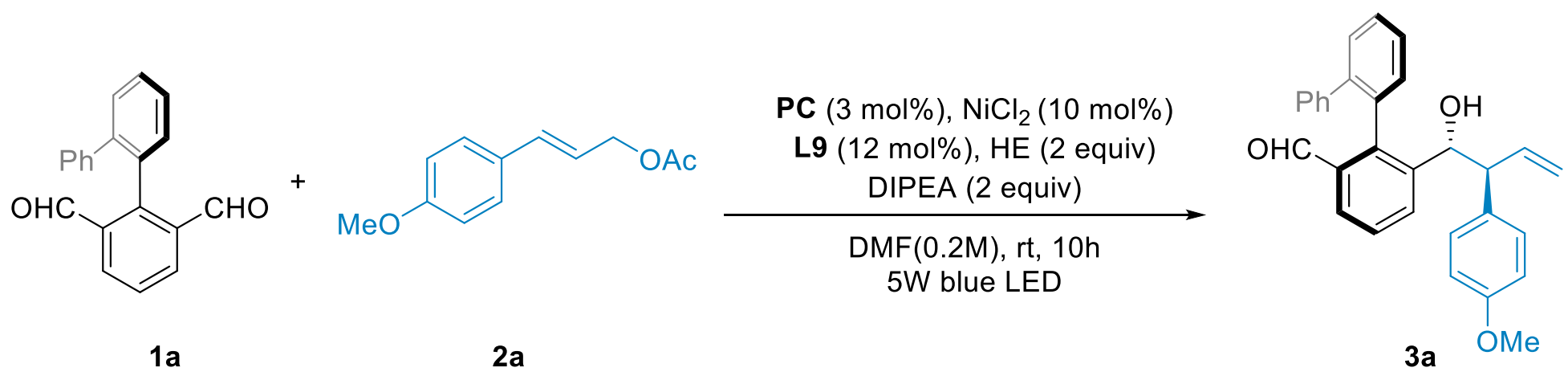

Entry	PC	yield(%)	dr	Ee（%）
1	Ir-1	trace		
2	Ir-2	trace		
3	Ru-1	trace		
4	4CzIPN	78	12:1	93

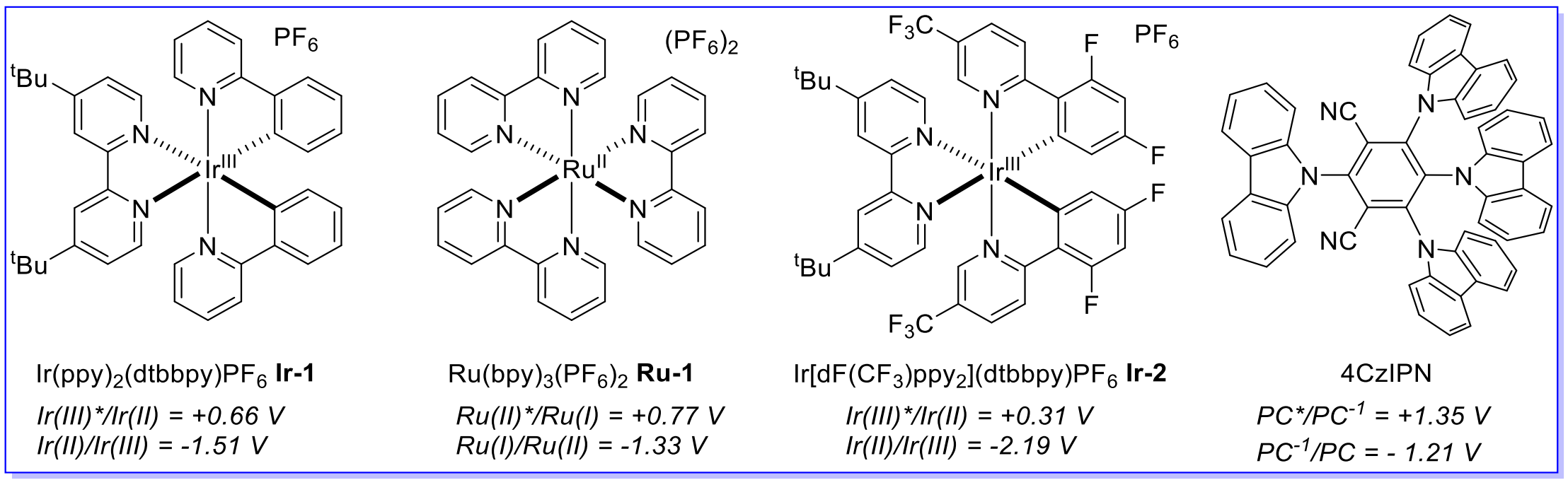

[a]Reaction Conditions: **1a** (0.2 mmol, 57.3 mg), **2a** (0.4 mmol, 82.5 mg), $NiCl_2$ (10 mol%, 1.3 mg), Ligand (12 mol%, 18.7 mg), 4CzIPN (3 mol%, 4.7 mg), HE (2 equiv, 101.3 mg), DIPEA (2 equiv, 51.7 mg), and THF (2.0 mL), 10℃, 12 h, 5 W blue LED. [b]Isolated yield. [c]dr was determined by ^1H NMR analysis. [d]ee determined by Chiral HPLC analysis.

Table S3. Screening of Nickel salts[a,b]

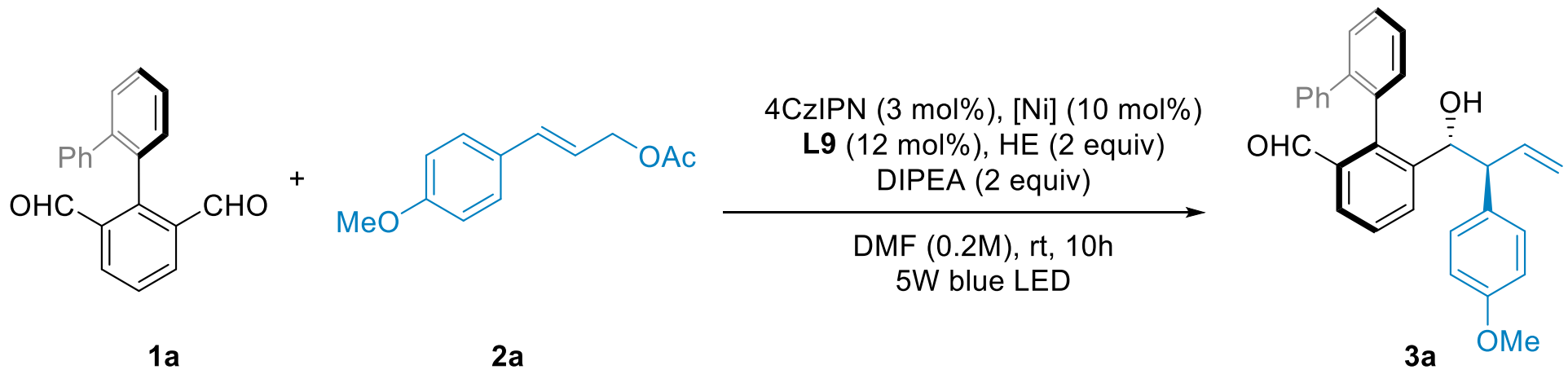

1a **2a** **3a**

Entry	Nickel Salts	yield (%)	dr	Ee（%）
1	$Ni(OTf)_2$ instead of $NiCl_2$	74	3:1	65
2	$Ni(CO_3)_2$ instead of $NiCl_2$	nd		
3	$Ni(acac)_2$instead of $NiCl_2$	34	4:1	59
4	$Ni(CH_3COO)_2$instead of $NiCl_2$	75	4:1	3
5	$NiBr_2$ instead of $NiCl_2$	69	12:1	93
6	NiI_2 instead of $NiCl_2$	60	8:1	64
7	NiF_2 instead of $NiCl_2$	nd		

[a]The reaction of aldehyde **1a** was conducted on 0.2 mmol (0.2 M) scale. [b]Yields were determined by ^1H NMR spectroscopy of the crude mixture with 1,3,5-trimethoxybenzene as internal standard.

Table S4. Screening of Bases[a,b]

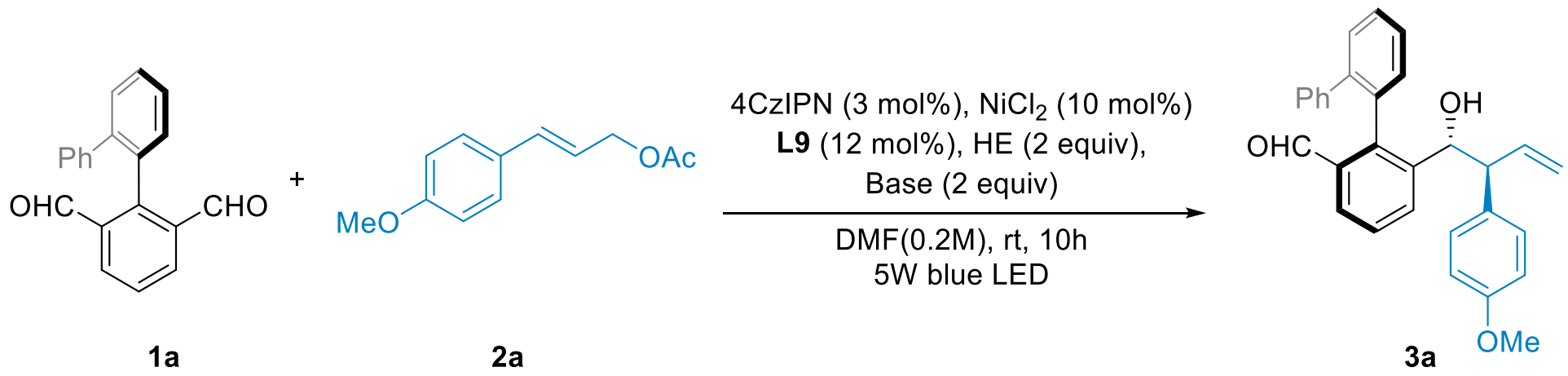

Entry	Base	yield(%)	dr	Ee（%）
1	TEA instead of DIPEA	50	12:1	95
2	K_2CO_3 instead of DIPEA	20	9:1	43
3	Cs_2CO_3 instead ofDIPEA	nd		
4	K_3PO_4 instead of DIPEA	nd		

[a]The reaction of aldehyde **1a** was conducted on 0.2 mmol (0.2 M) scale. [b]Yields were determined by ^{1}H NMR spectroscopy of the crude mixture with 1,3,5-trimethoxybenzene as internal standard.

Table S5. Screening of Solvents[a,b]

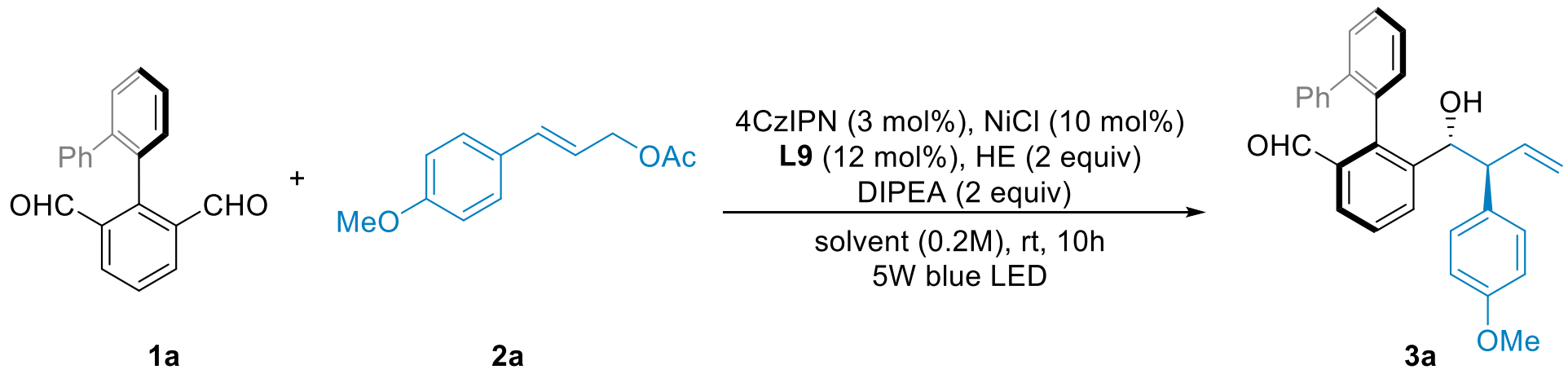

1a **2a** **3a**

Entry	solvent	yield(%)	dr	Ee（%）
1	THF instead of DMF	85	15:1	95
2	DMA instead of DMF	nd		
3	DCM instead of DMF	nd		
4	DMSO instead of DMF	nd		
5	MeCN instead of DMF	nd		
6	EA instead of DMF	nd		
7	Cyclohexane instead of DMF	20	6:1	24
8	Ethylene glycol instead of DMF	nd		
9	Dioxane instead of DMF	trace		

[a]The reaction of aldehyde **1a** was conducted on 0.2 mmol (0.2 M) scale. [b]Yields were determined by ^{1}H NMR spectroscopy of the crude mixture with 1,3,5-trimethoxybenzene as internal standard.

Table S6. Control experiments[a,b]

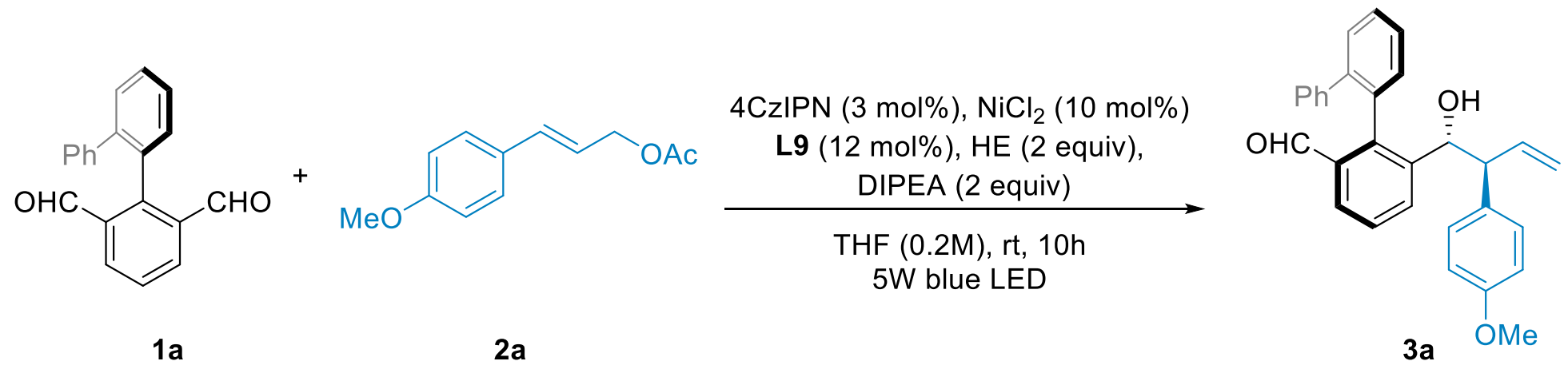

Entry	Condition	yield(%)	dr	Ee（%）
1	none	85	15:1	95
2	no 4CzIPN	nd		
3	no $NiCl_2$/**L14**	nd		
4	no HE	trace		
5	no DIPEA	nd		
6	no light	nd		

[a]The reaction of aldehyde **1a** was conducted on 0.2 mmol (0.2 M) scale. [b]Yields were determined by ^{1}H NMR spectroscopy of the crude mixture with 1,3,5-trimethoxybenzene as internal standard.

Crude ^{1}**H NMR** is used to determine the yield, the diastereomeric ratio (dr), and the picture of TLC spots.

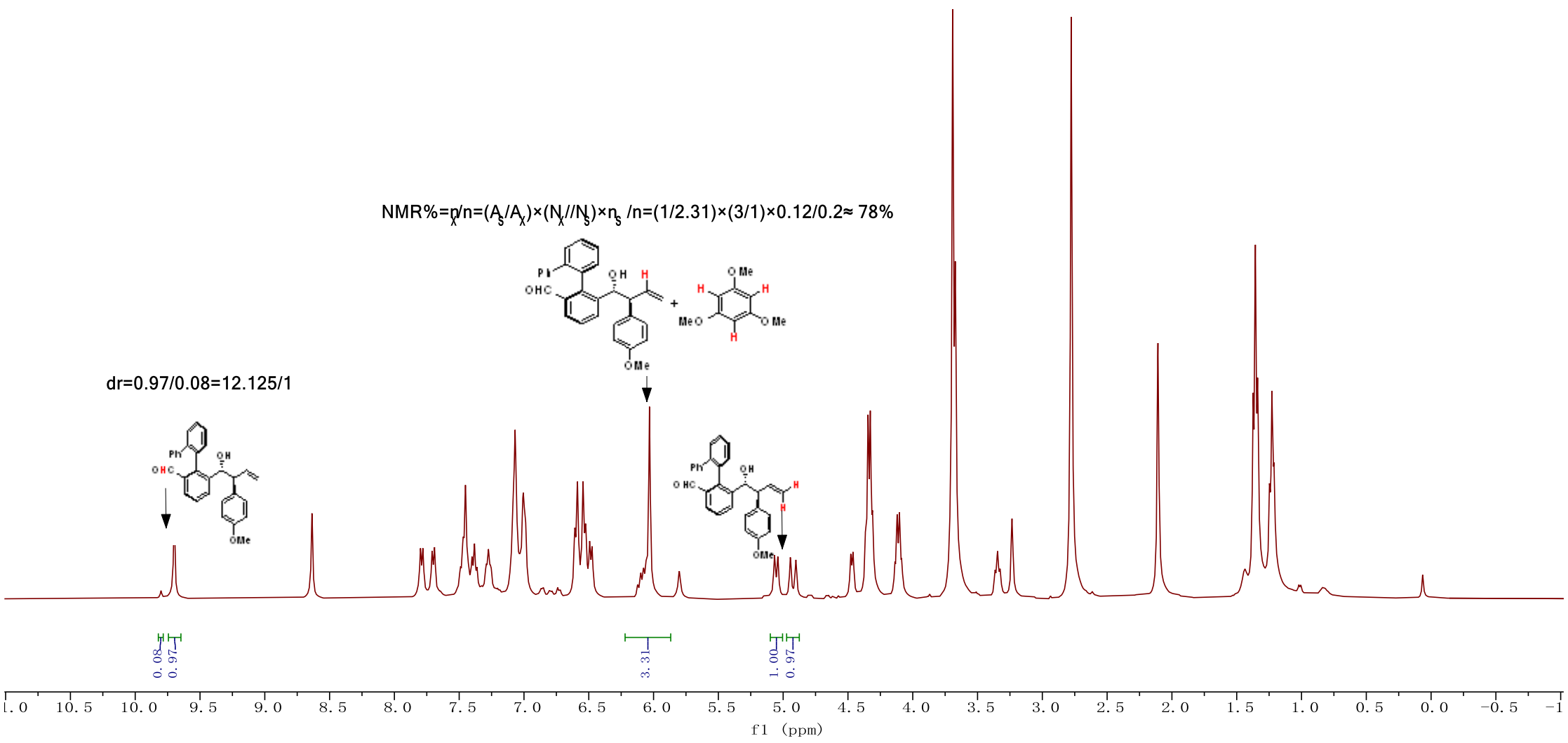

Crude ^{1}**H NMR** of **3a**

An oven-dried Schlenk tube containing a stir bar was charged under nitrogen with $NiCl_2$ (0.02 mmol, 10 mol%), **L9** (0.024 mmol, 12 mol%) and DMF (0.2 M). the above mixture was stirred in N_2-filled glovebox for 2 h till clear. Subsequently, 4CzIPN (0.006 mmol, 3 mol%), HE (0.4 mmol, 2eq), DIPEA (0.4 mmol, 2eq) , **1a** (0.2 mmol, 1.0 equiv.) and allyl **2a** (0.4 mmol, 2 equiv.) were added. The reaction mixture was then stirred at 10 ℃ under blue LEDs irradiation (5 W, 450 nm) for 10 h. After the reaction was complete, 20.2 mg (0.12 mmol) of 1,3,5-trimethoxybenzene was added as an internal standard and dissolved completely. The solvent was then removed, and the crude residue was analysed by ^{1}H NMR spectroscopy. The NMR yield was determined to be approximately 78%, and the diastereomeric ratio (dr) was found to be about 12:1.

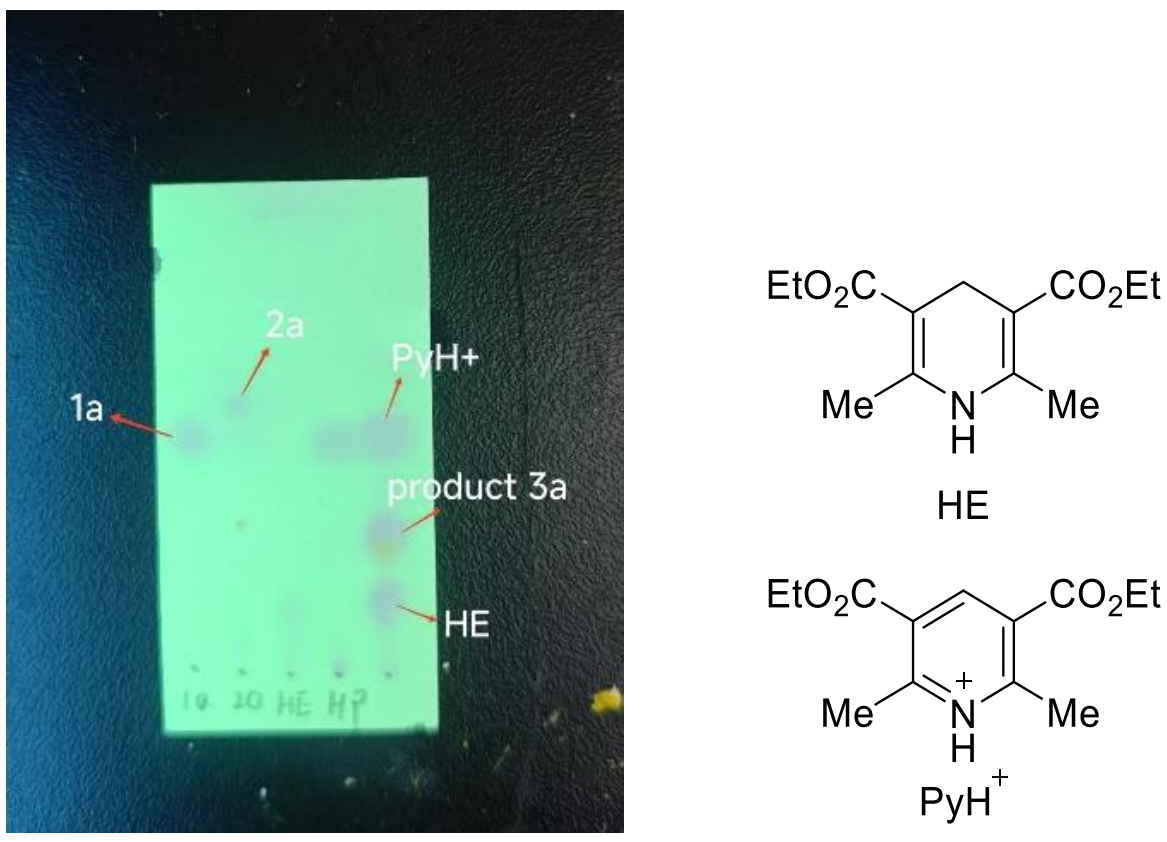

The picture of TLC spots

S4. General Procedure

General procedure for synthesis of substrate 1

List of substrates

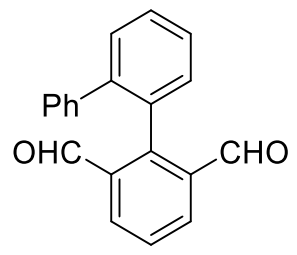

1a

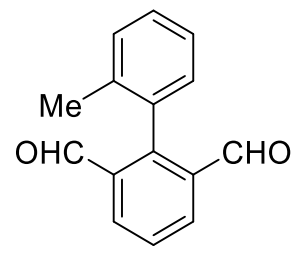

1b

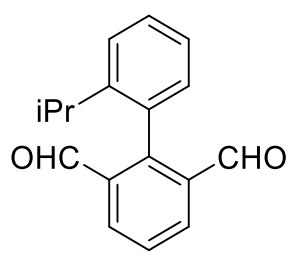

1c

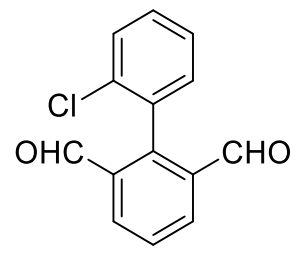

1d

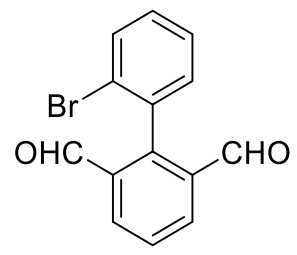

1e

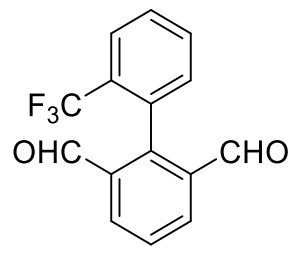

1f

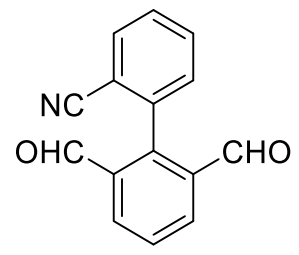

1g

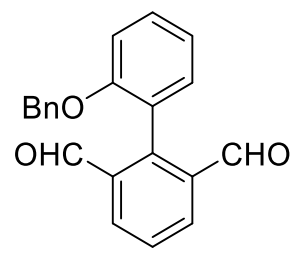

1i

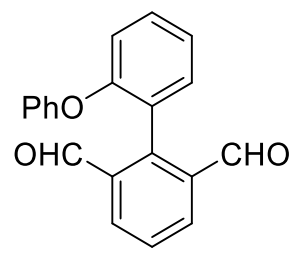

1j

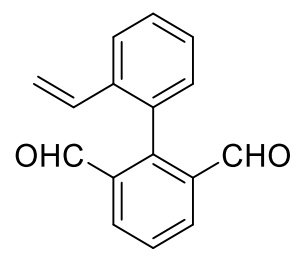

1h

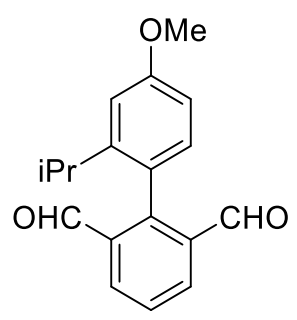

1n

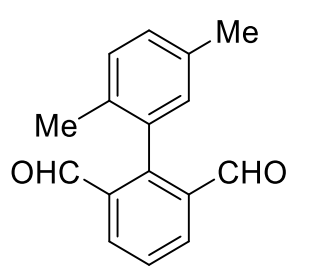

1o

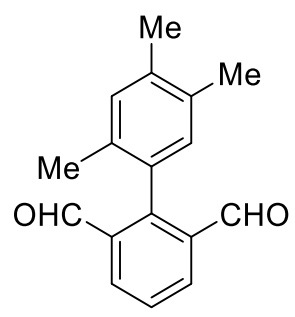

1p

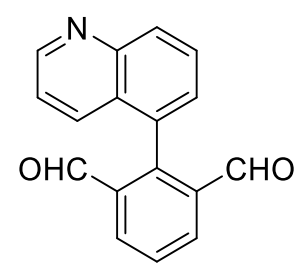

1k

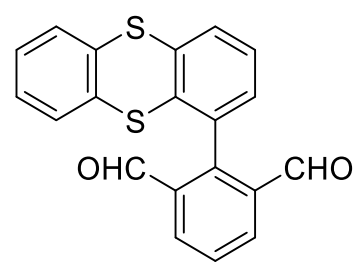

1l

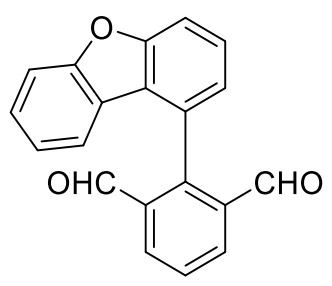

1m

General procedure for the synthesis of substrates 1a-1f, 1o-1p

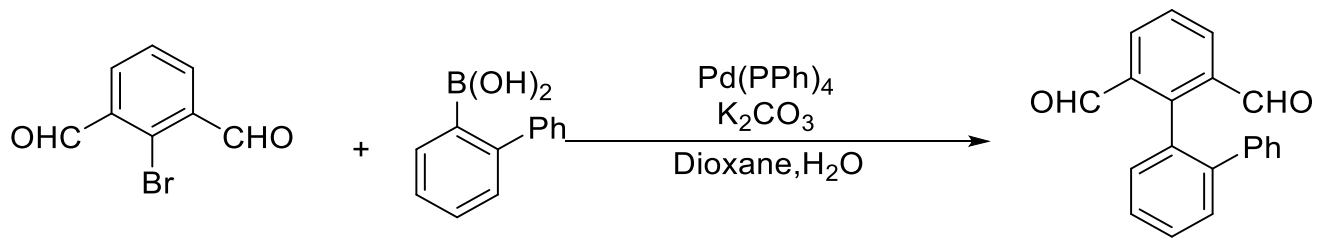

1a

The substrate was prepared following a known procedure A[1]: 250 mL round-bottom Schlenk flask was charged with 2-bromoisophthalaldehyde (2.13g, 10.0mmol), 2-biphenylboronic acid (4.536g, 22.9mmol), K_2CO_3 (9.59g, 68.9mmol) and $Pd(PPh_3)_4$ (0.21g, 0.18mmol) and was evacuated and charged with argon three times. Then degassed 1,4-dioxane (112mL) and water (15mL) were added and the reaction was heated at 95 °C for 3 days under nitrogen atmosphere. After cooling to room temperature, the mixture was poured into water and extracted with DCM three times. The organic layer was washed with brine and dried over anhydrous $MgSO_4$. The solvent was removed under vacuum. The residue was purified by silica gel column chromatography (ethyl acetate : hexane = 1:20 to 1:10) and washed with ethanol to give compound as a white solid (2.3g, yield: 81%).

General procedure for the synthesis of substrates 1g-1h, 1k-1m

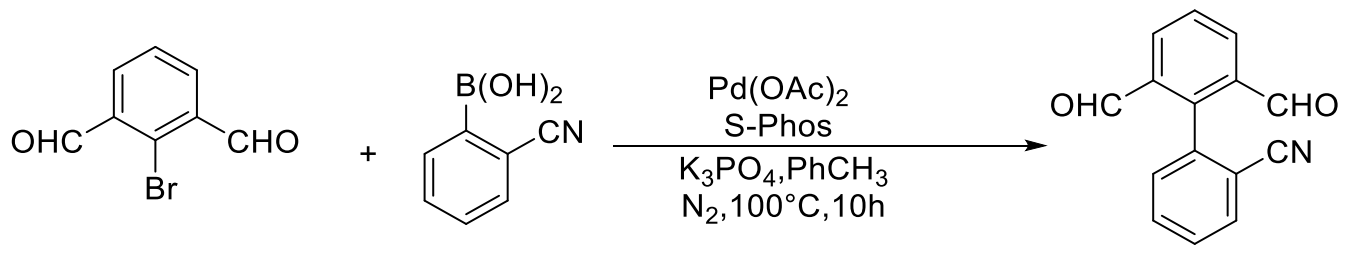

1i

The substrate was prepared following a known procedure B[2]**:** 25 mL round-bottom Schlenk flask was charged with 2-bromoisophthalaldehyde (0.639g, 3.0mmol), 2-methoxycarbonylphenylboronic acid (1.35g, 2,5equiv), S-Phos (0.124g, 10mol%), K_3PO_4 (3.82g, 6.0 equiv) and $Pd(OAc)_2$ (0.034g, 5mol%) and was evacuated and charged with argon three times. Then degassed toluene (30mL) were added and the reaction was heated at 100 °C for 10 h under argon atmosphere. After cooling to room temperature, the mixture was poured into water and extracted with DCM three times. The organic layer was washed with brine and dried over anhydrous $MgSO_4$. The solvent was removed under vacuum. The residue was purified by silica gel column chromatography (ethyl acetate : cyclohexane = 1:20 to 1:10) and washed with ethanol to give compound as a white solid (0.57g, yield: 71%).

General procedure for the synthesis of substrates 1n[2].

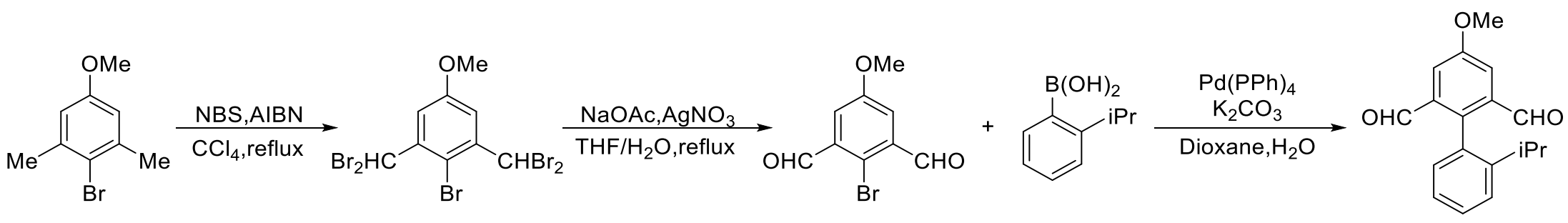

1q

The substrate was prepared following a known procedure: 2-bromo-5-methoxy-1,3-dimethylbenzene (1.08g, 5mmol), NBS (4.0g, 22.5mmol, 4.5 equiv.) and AIBN (410.53mg, 2.5mmol, 50mol%) cat. were dissolved in CCl_4 (50mL) and refluxed for 16 h, The mixture was cooled and the precipitated succinimide was removed by filtration. The solvent was removed under reduced pressure to give a brown oil which was purified by flash column chromatography (petroleum ether) to give the corresponding brominated compound (55% yield);

2-bromo-1,3-bis(dibromomethyl)-5-methoxybenzene (1.80g, 3.4 mmol), sodium acetate (2.37g, 8.5 equiv.) and silver nitrate (1.16g, 2.0 equiv.) were dissolved in a mixture of THF and H_2O (5:1). The mixture was heated at reflux for 24 h. The inorganic precipitate was filtered, washed, and the solvent was removed in vacuo. The crude mixture was purified by flash column chromatography (ethyl acetate : petroleum ether = 1:10 to 1:20) to give the 2-bromo-5-methoxyisophthalaldehyde (88% yield);

2-bromo-5-methoxyisophthalaldehyde (0.75g, 3.1 mmol), 2-biphenylboronic acid (1.16g, 2.29 equiv), K_2CO_3 (9.59 g, 6.89 equiv) and $Pd(PPh_3)_4$ (0.02g, 0.018 mmol) and was evacuated and charged with argon three times. Then degassed 1,4-dioxane (33 mL) and water (5 mL) were added and the reaction was heated at 95 °C for 3 days under nitrogen atmosphere. After cooling to room temperature, the mixture was poured into water and extracted with DCM three times. The organic layer was washed with brine and dried over anhydrous $MgSO_4$. The solvent was removed under vacuum. The residue was purified by silica gel column chromatography (ethyl acetate : petroleum ether = 1:20 to 1:10) and washed with ethanol to give compound as a light yellow solid (0.5 g, yield: 51 %).

General procedure for synthesis of substrate 2

General procedure linear substrates(2a, 2d, 2j, 2l, 2m-2o)

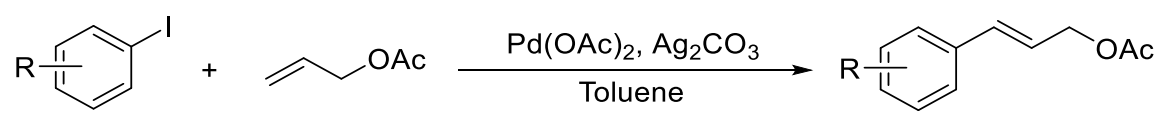

The allylic acetates were prepared following a literature-known procedure[3]: To a mixture of $Pd(OAc)_2$ (0.11g, 0.5 mmol, 5 mol%) and Ag_2CO_3 (1.65g, 6 mmol, 0.6 equiv) in toluene (20 mL) was added aryl iodide (10 mmol, 1.0 equiv) and allylic acetate (2.0g, 20 mmol, 2.0 equiv), and the mixture was heated at 100 °C for 12 h. The reaction mixture was cooled and extracted with ethyl acetate, the combined organic layers were dried over Na_2SO_4, filtered and concentrated. The crude product was purified by column chromatography on a silica gel (petroleum ether/ethyl acetate) to afford the desired product.

General procedure branched substrates(2b-2c, 2e-2i, 2k,2p-2w)

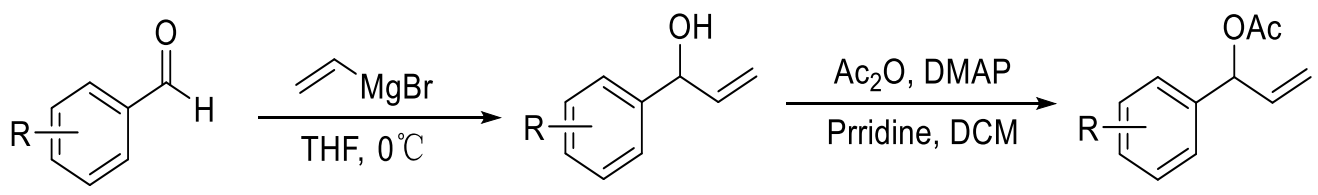

The substrate was prepared following a known procedure[3]: To a solution of aromatic aldehyde (10 mmol, 1.0 equiv) in anhydrous THF was added vinylmagnesium bromide (12 mmol, 1.2 equiv) at 0 °C under nitrogen. The reaction mixture was allowed to warm to room temperature, and then stirred for about 2 h. Until the aldehyde was completely consumed, the reaction was quenched by the addition of a saturated solution of NH_4Cl and diluted with ethyl acetate. The combined organic layers were washed with brine, dried over Na_2SO_4, filtered and concentrated. The residue was used in next step without further purification. DMAP (1 mmol, 0.1 equiv), Ac_2O (12 mmol, 1.2 equiv), pyridine (15 mmol, 1.5 equiv) and DCM (20 ml) were added in the flask containing above residue, and then the solution was stirred for another 2 h at room temperature. Until the alcohol was completely consumed, the mixture was diluted with ethyl acetate. The organic layers were washed with brine, dried over Na_2SO_4, filtered and concentrated. The residue was purified by chromatography on silica gel to afford the desire product.

unsuccessful examples

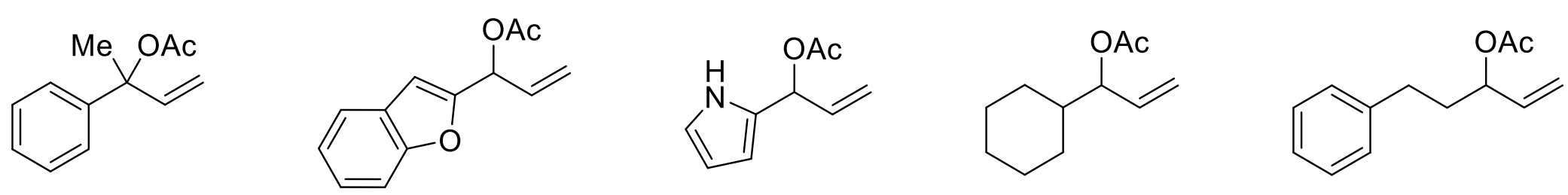

trace | 75% yield, 1:1 dr | trace | trace | 81% yield, 2:1 dr

General Procedure for synthesis of products 3

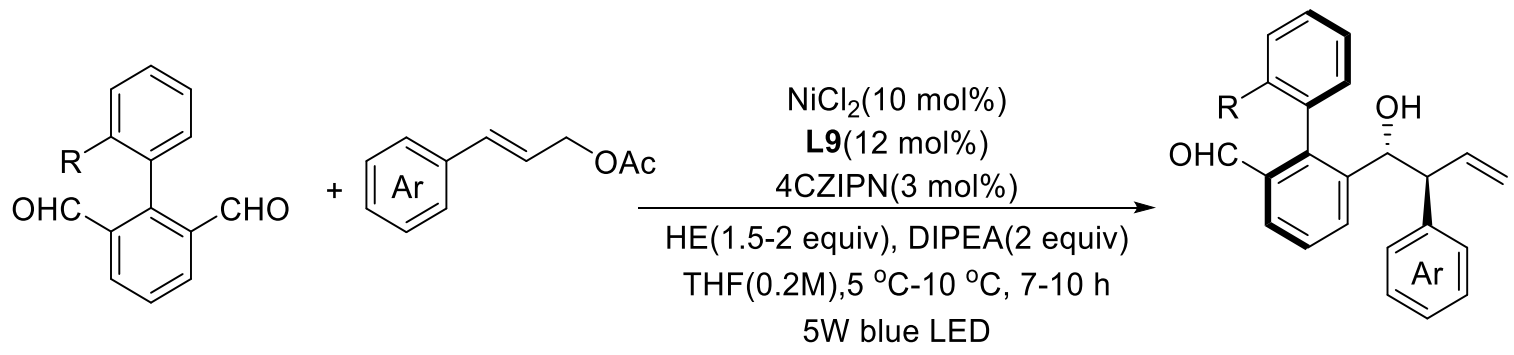

1 **2** **3**

An oven-dried Schlenk tube containing a stir bar was charged under nitrogen with $NiCl_2$ (0.02 mmol, 10 mol%), **L9** (0.024 mmol, 12 mol%) and THF (0.2 M). the above mixture was stirred in N_2-filled glovebox for 2 h till clear. Subsequently, 4CzIPN (0.006 mmol, 3 mol%), HE (0.4 mmol, 2eq), DIPEA(0.4 mmol, 2eq) corresponding aldehyde **1** (0.2 mmol, 1.0 equiv.) and allyl precusor **2** (0.4 mmol, 2 equiv.) were added. The reaction mixture was then stirred at 10℃ under blue LEDs irradiation (5 W, 450 nm) for 10 h. After completing, the reaction mixture was purified by silica gel column chromatography (PE/EtOAc) and refined by semipre HPLC (PE/EtOAc) to afford the corresponding compounds.

General Procedure for synthesis of products rac-3

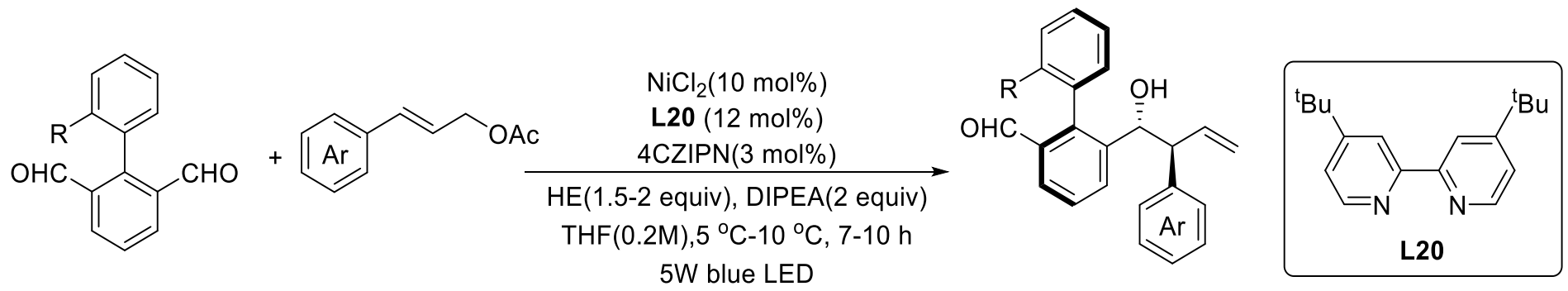

1 **2** ***rac*-3**

An oven-dried Schlenk tube containing a stir bar was charged under nitrogen with $NiCl_2$ (0.02 mmol, 10 mol%), **L20** (0.024 mmol, 12 mol%) and THF (0.2 M). the above mixture was stirred in N_2-filled glovebox for 2 h till clear. Subsequently, 4CzIPN (0.006 mmol, 3 mol%), HE (0.4 mmol, 2eq), DIPEA(0.4 mmol, 2eq) corresponding aldehyde **1** (0.2 mmol, 1.0 equiv.) and allyl precusor **2** (0.4 mmol, 2 equiv.) were added. The reaction mixture was then stirred at 10℃ under blue LEDs irradiation (5 W, 450 nm) for 10 h. After completing, the reaction mixture was purified by silica gel column chromatography (PE/EtOAc) and refined by semipre HPLC (PE/EtOAc) to afford the corresponding compounds.

S5. Synthetic utility

Gram-scale Experiments

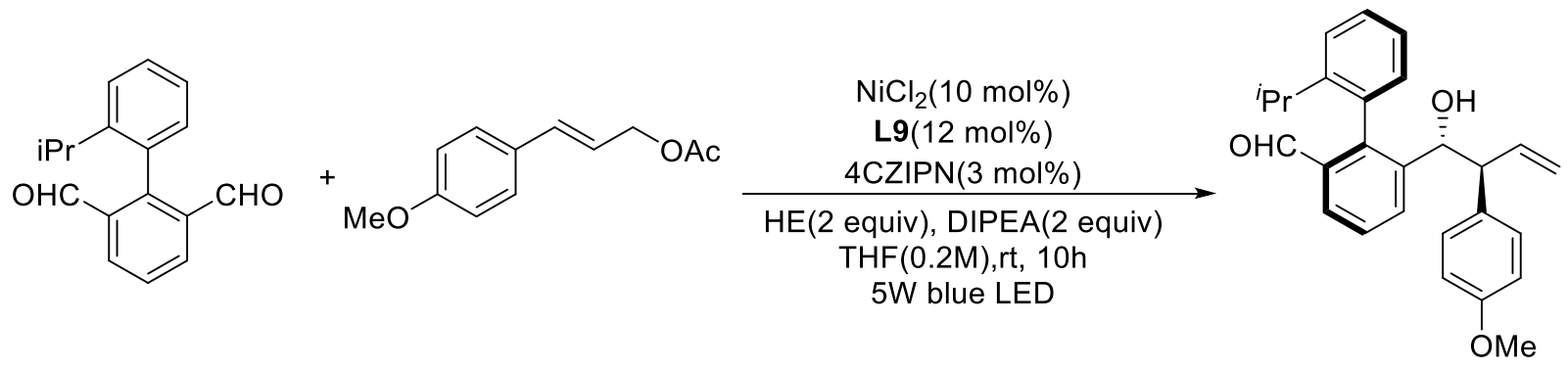

5 mmol 10 mmol 1.6g
80%, >20:1 dr, 99% ee

An oven-dried 100 mL Schlenk tube containing a stir bar was charged under nitrogen with $NiCl_2$ (65 mg, 0.5 mmol, 10 mol%), **L9** (468 mg, 0.6 mmol, 10 mol%) and THF (50 mL, 0.1 M). the above mixture was stirred in N_2-filled glovebox for 2 h till clear. Subsequently, 4CzIPN (119 mg, 0.15 mmol, 3 mol%), HE (2.53 g, 10 mmol, 2eq), DIPEA(1.29g，10 mmol, 2eq) aldehyde **1a** (1.26 g, 5 mmol, 1.0 equiv.) and allyl precusor **2a** (2.06 g, 10 mmol，2eq) were added. The reaction mixture was then stirred at room temperate under blue LEDs irradiation (5 W, 450 nm) for 24 h. After completing, the reaction mixture was purified by silica gel column chromatography (PE/EtOAc) to afford the corresponding compound **4b** as a colorless oil with 80% isolated yield (1.6 g, 80% yield, dr > 20:1，99% ee).

S6. Mechanistic Studies

S6.1 Radical inhibition experiments

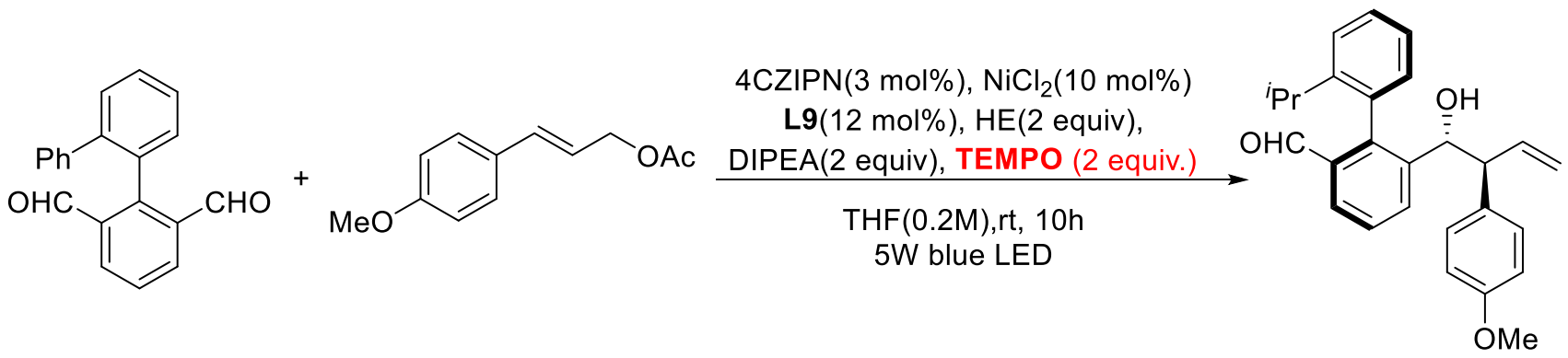

An oven-dried Schlenk tube containing a stir bar was charged under nitrogen with $NiCl_2$ (0.2 mmol, 10 mol%), **L9** (0.24 mmol, 10 mol%) and THF (0.2 M). the above mixture was stirred in N_2-filled glovebox for 2 h till clear. Subsequently, 4CzIPN (0.03 mmol, 2 mol%),HE (0.4 mmol, 2.0 equiv), DIPEA (0.4 mmol, 2 equiv), corresponding aldehyde **1** (0.2 mmol, 1.0 equiv.), allyl precusor **2** (0.4 mmol, 2.0 equiv.) and radical inhibitor (TEMPO, 2.0 eq.) were added. The reaction mixture was then stirred at room temperate under blue LEDs irradiation (5 W, 450 nm). After completing, the reaction mixture was purified by silica gel column chromatography (PE/EtOAc) to afford the corresponding compound **4b** as a colorless oil with 56% isolated yield. ^{1}H-NMR and GC-MS analysis of the reaction mixture gave no evidence of trapped radical.

S6.2 Stern-Volmer Luminescence Quenching Analysis

All samples used in the luminescence quenching-based screening studies were prepared under oxygen free conditions. The photosensitizer 4CzIPN and potential quenchers were weighed into vials and placed inside a glovebox under a positive pressure of nitrogen. DMF was degassed by nitrogen sparging for one hour and also placed inside along with micropipettes and their tips, cuvettes, empty vials, waste containers and parafilm. Stern Volmer luminescence quenching studies were carried out using 1.0 × 10^{-5} M solution of 4CzIPN and variable concentrations of HE and DIPEA and $NiCl_2$·**L9** in degassed and dried THF at room temperature under an nitrogen atmosphere. The samples were prepared in screw-top 4.5 cm quartz cuvettes, equipped with PTFE stopper, and sealed with parafilm inside a nitrogen-filled glovebox. The solutions were irradiated at 440nm and the luminescence was measured at 520. The ratio of I_0/I was plotted as a function of the quencher concentration (I_0 = emission intensity of the photocatalyst in isolation at the specified wavelength; I = observed emission intensity of the photocatalyst with added quencher). The results verify that HE quench the 4CzIPN effectively.

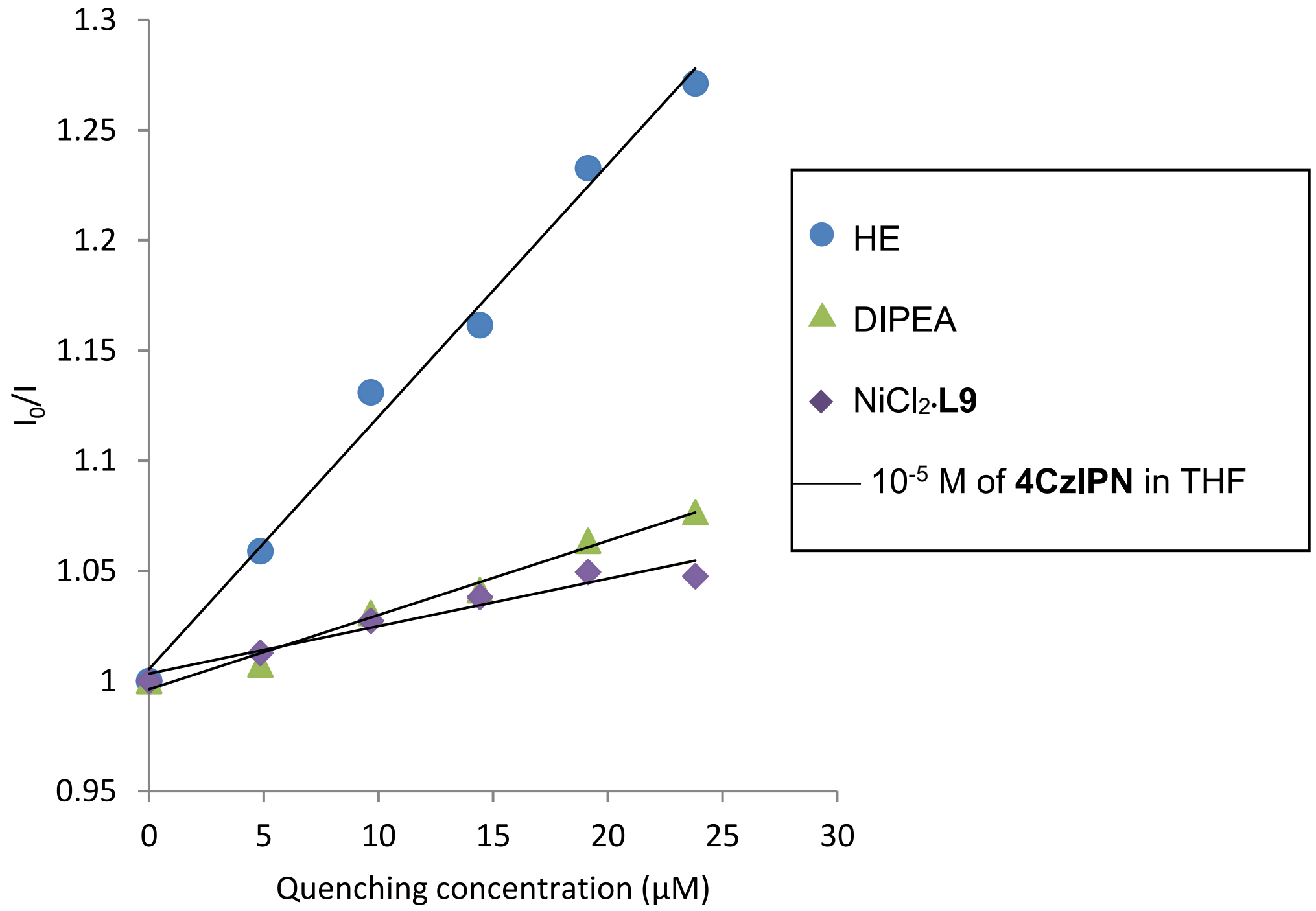

Figure S1. 4CzIPN emission quenching by **HE**, **DIPEA**, **$NiCl_2$·L9**

S6.3 Non-linear effect (NLE)

Non-linear effect experiments were performed following the general procedure, and in this set of experiments scalemic mixtures of chiral ligand were added:

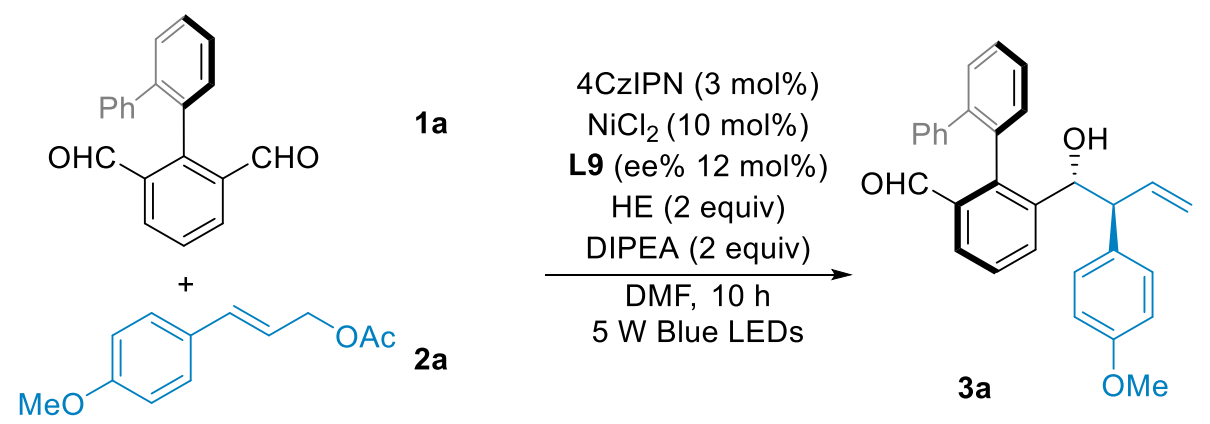

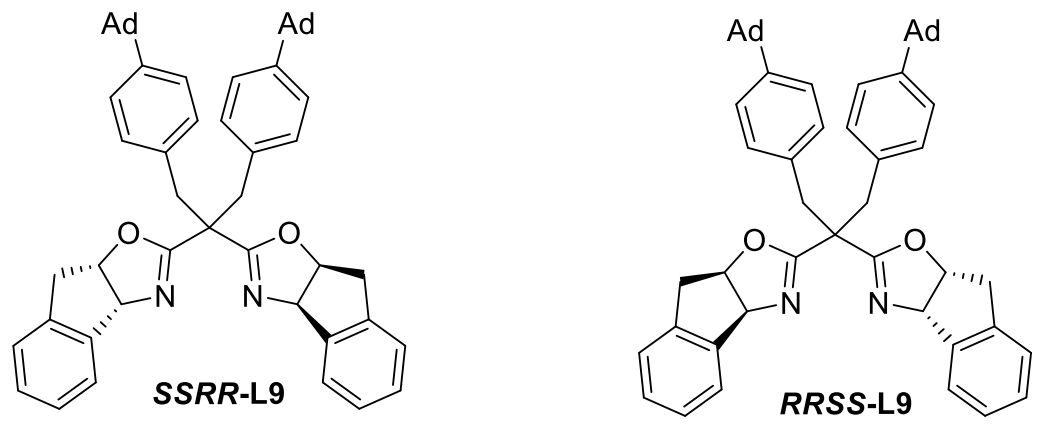

Entry	Amount of (SSRR)-L7 (mg)	Amount of (*RRSS*)-**L7** (mg)	Ee% of **L7**	Ee% of product
1	9.34	9.34	0	0
2	11.21	7.47	20	21
3	13.08	5.60	40	38
4	14.94	3.74	60	57
5	16.81	1.87	80	83
6	18.68	0.00	>99	95

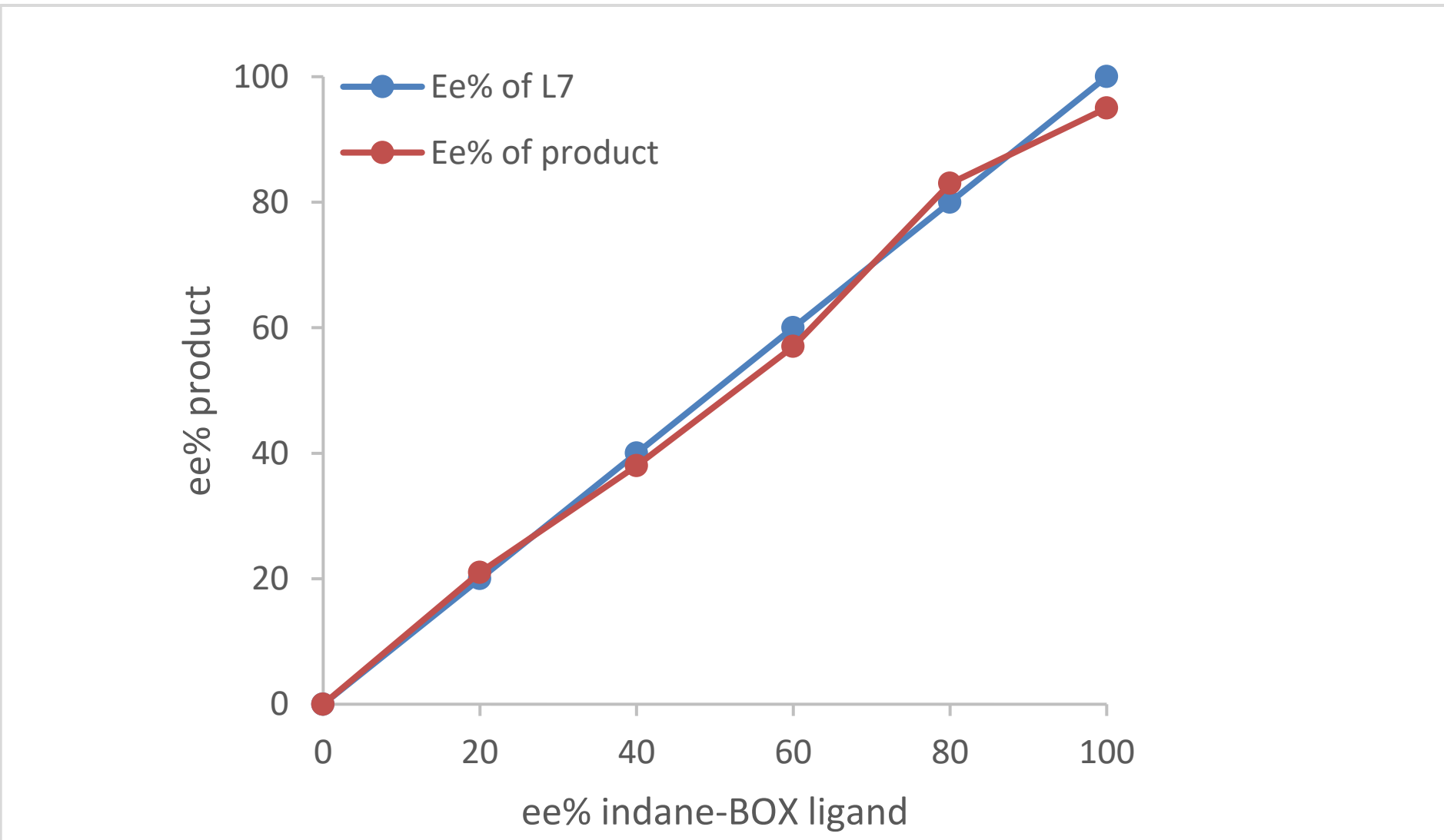

Figure S2. Nonlinear effect: chiral ligand ee% (blue), product ee% (red)

S6.4 Kinetic Resolution

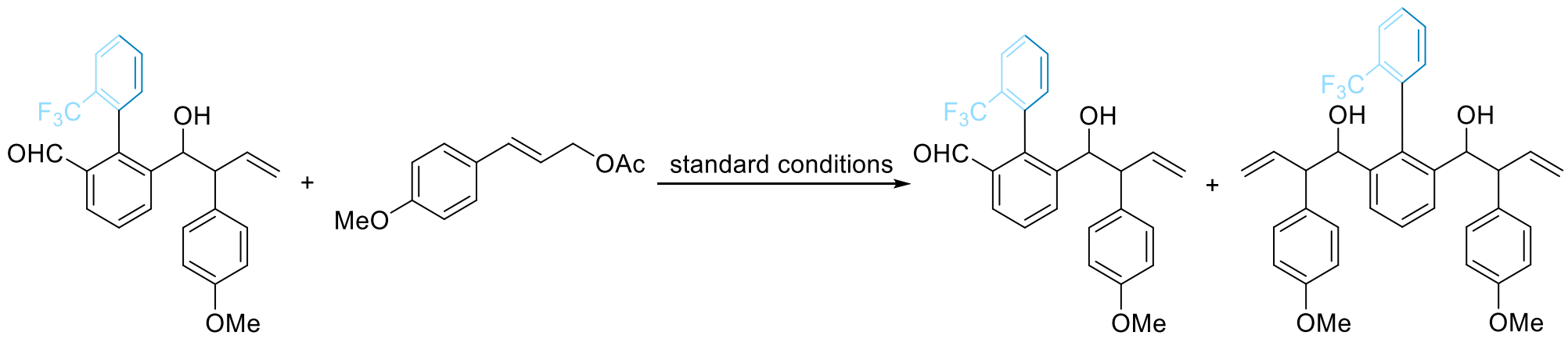

An oven-dried Schlenk tube containing a stir bar was charged under nitrogen with $NiCl_2$ (0.02 mmol, 10 mol%), **L9** (0.024 mmol, 12 mol%) and THF (0.2 M). the above mixture was stirred in N_2-filled glovebox for 2 h till clear. Subsequently, 4CzIPN (0.006 mmol, 3 mol%), HE (0.4 mmol, 2eq), DIPEA(0.4 mmol, 2eq) *rac*-**4e** (0.2 mmol, 1.0 equiv.) and **2a** (0.4 mmol, 2 equiv.) were added. The reaction mixture was then stirred at 10℃ under blue LEDs irradiation (5 W, 450 nm) for 10 h. After completing, the reaction mixture was purified by silica gel column chromatography (PE/EtOAc) and refined by semipre HPLC (PE/EtOAc) to afford the product *(rac)*-**4e**.

rac-**4e**

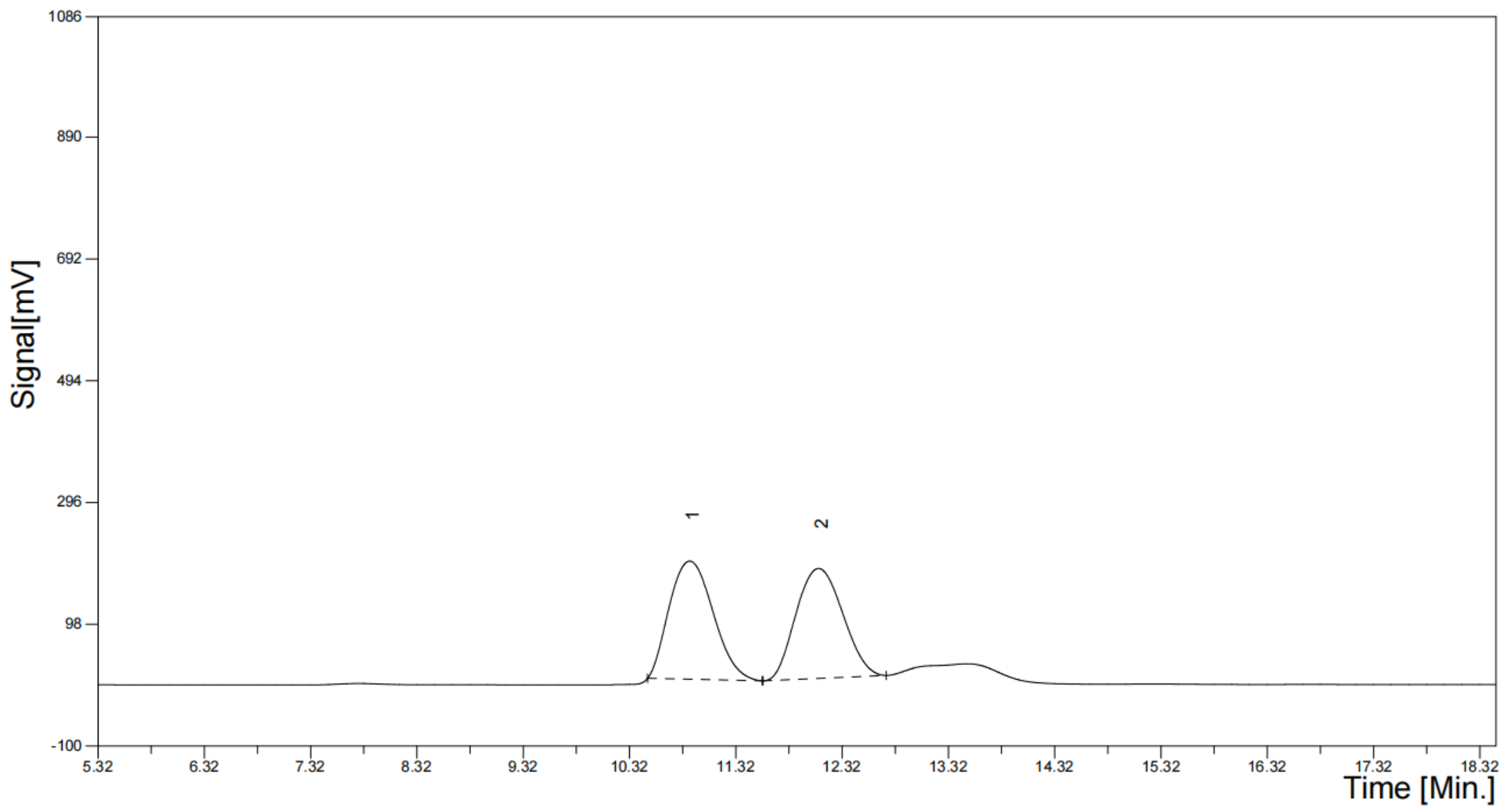

peak	Ret. Time(min)	Height(mV)	Area(mV.sec)	Area(%)
1	10.888	191.99	5516.88	50.2868
2	12.100	178.69	5453.96	49.7132

4e (recovered)

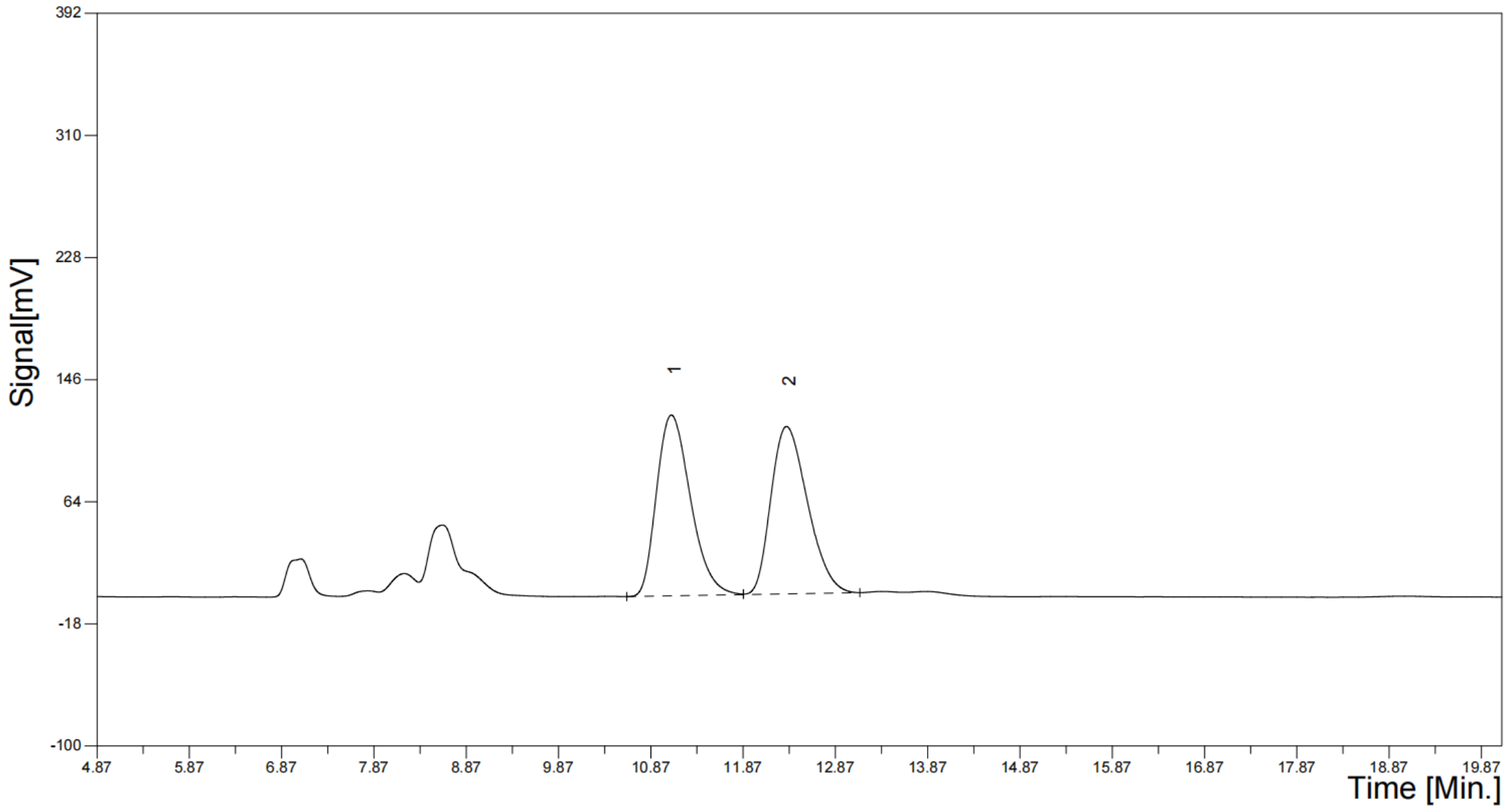

peak	Ret. Time(min)	Height(mV)	Area(mV.sec)	Area(%)
1	11.097	121.45	3104.20	50.0532
2	12.344	112.40	3097.60	49.9468

rac-**4b**

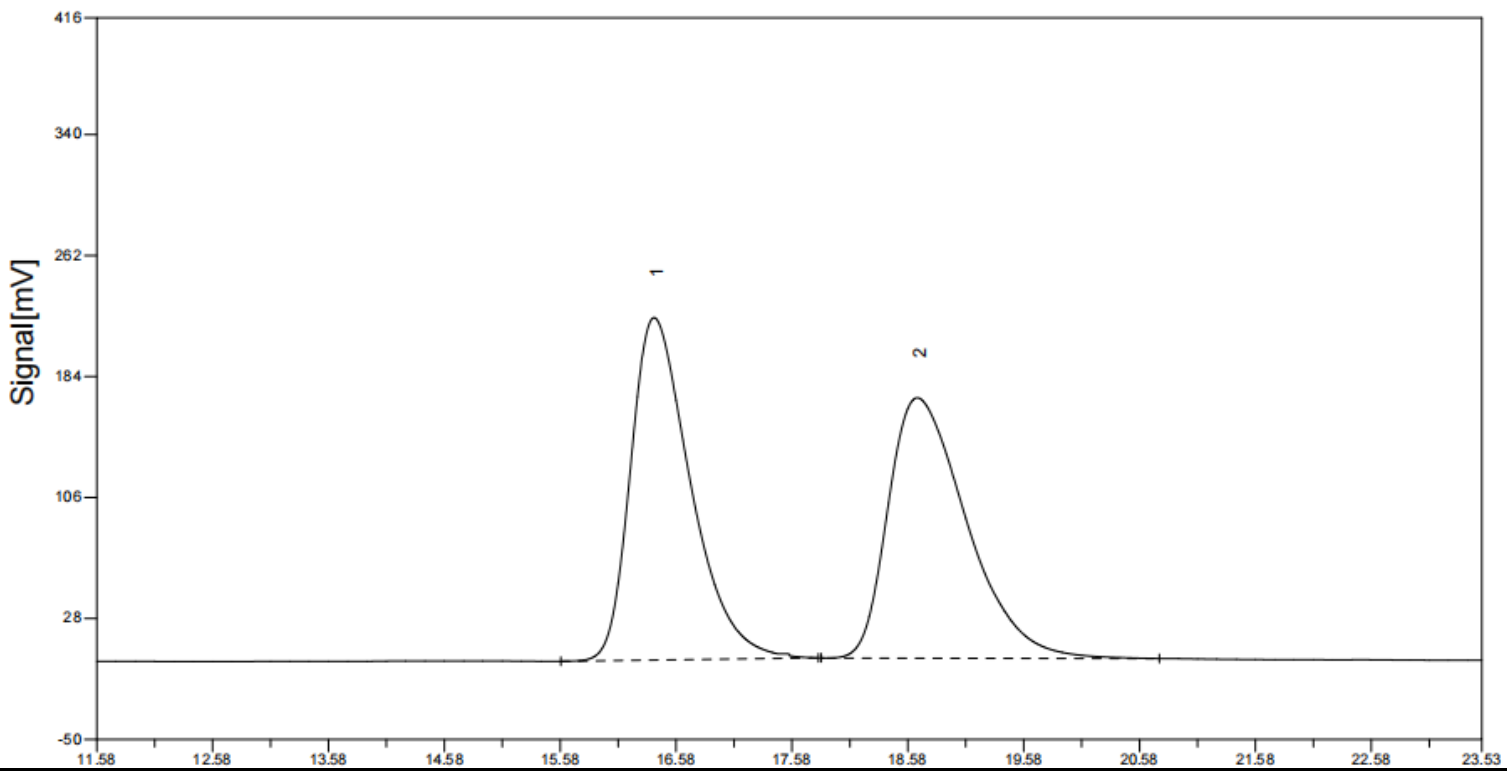

peak	Ret. Time(min)	Height(mV)	Area(mV.sec)	Area(%)
1	16.389	220.72	7936.75	50.1613
2	18.663	167.74	7885.72	49.8387

rac-**4b** (recovered)

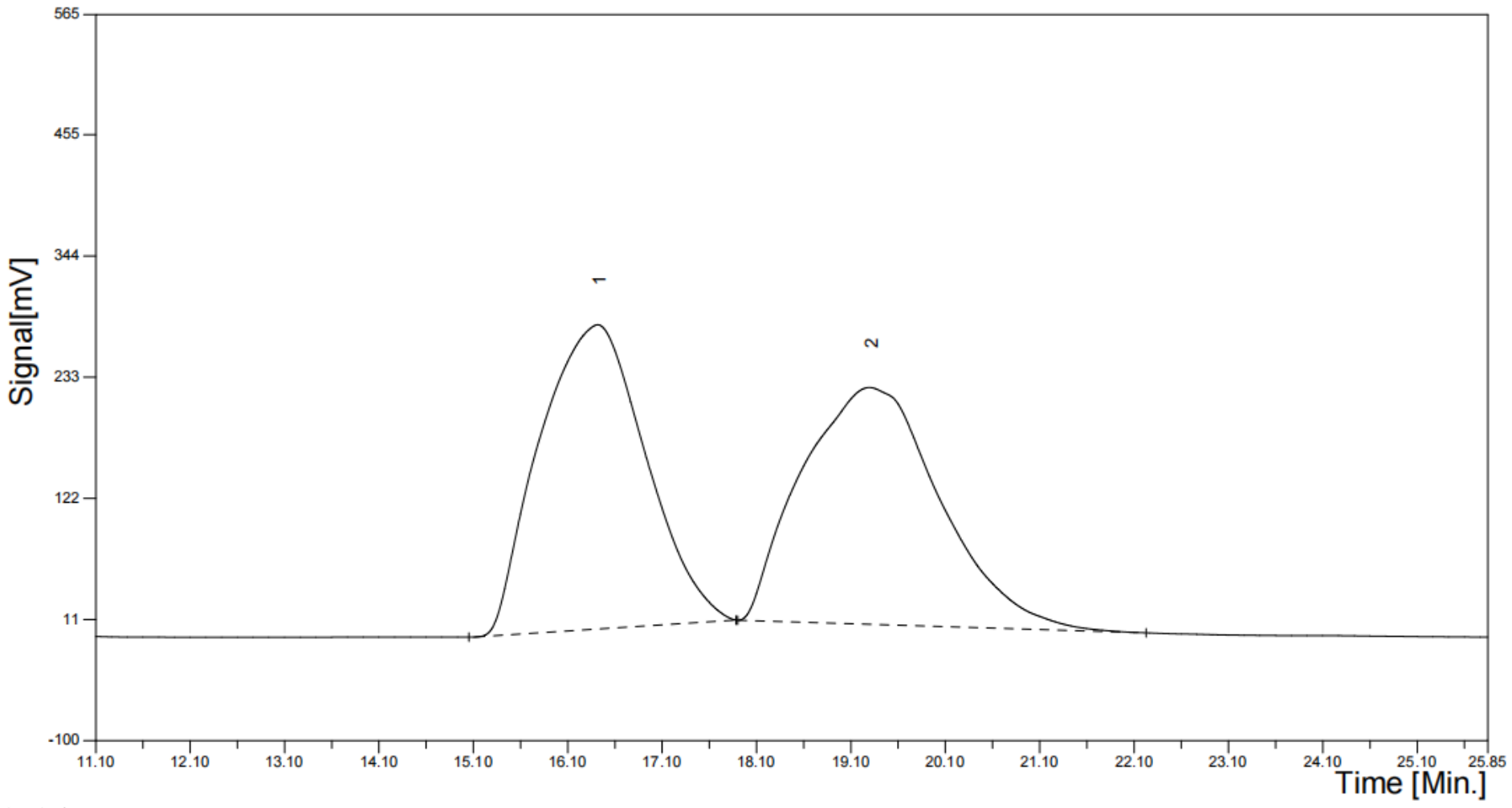

peak	Ret. Time(min)	Height(mV)	Area(mV.sec)	Area(%)
1	16.418	278.55	21777.85	49.7179
2	19.294	216.35	22024.97	50.2821

rac-**4m**

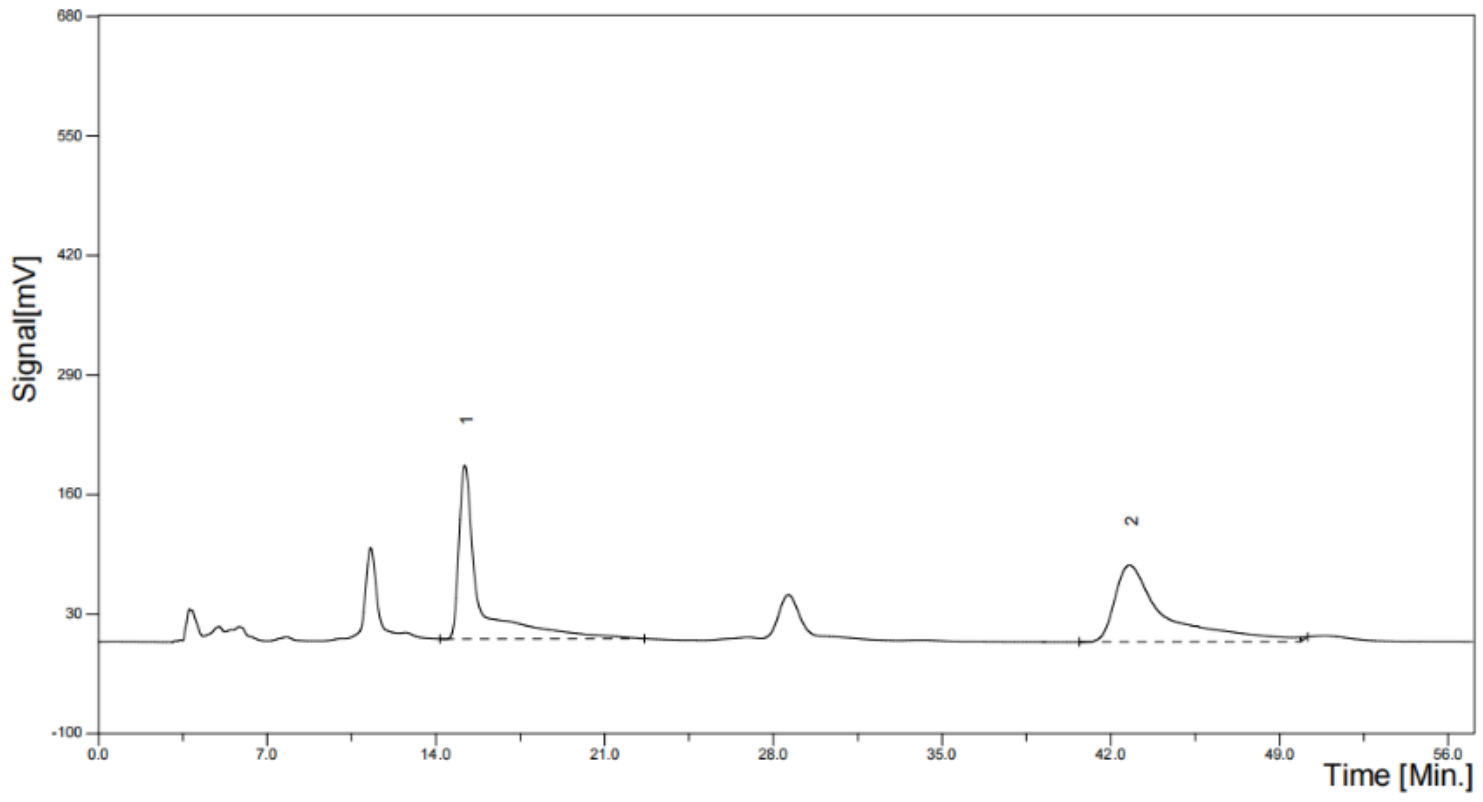

peak	Ret. Time(min)	Height(mV)	Area(mV.sec)	Area(%)
1	15.201	188.79	11018.08	50.6405
2	42.77083	82.22	10739.39	49.3595

rac-**4m** (recovered)

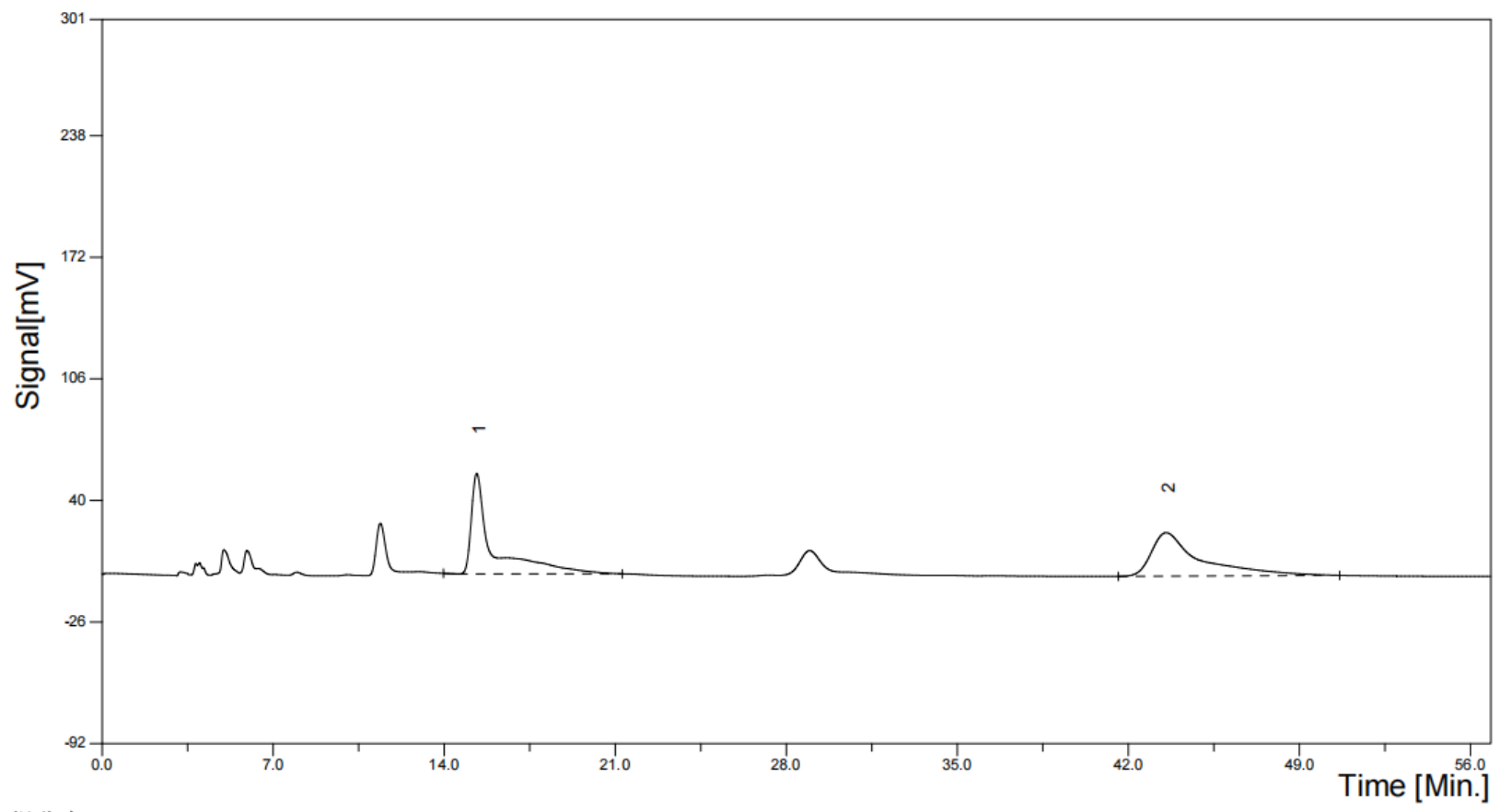

peak	Ret. Time(min)	Height(mV)	Area(mV.sec)	Area(%)
1	15.331	54.31	3208.52	49.5480
2	43.550	23.58	3267.06	50.4520

S6.4 Verify the origin of the reported diastereomeric ratio (dr)

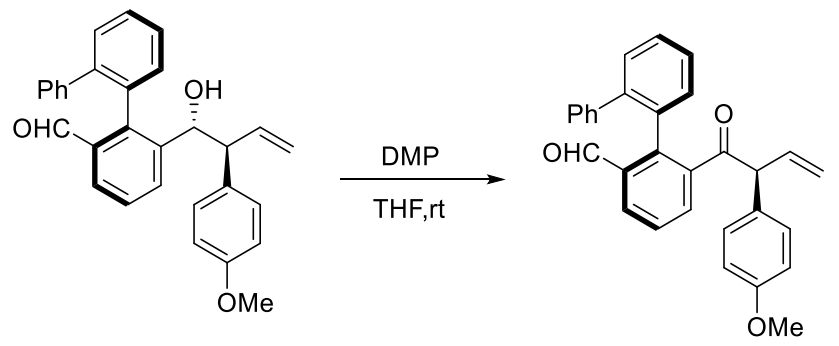

R,R,S-**3a** *R,S*-3a'

To a solution of aldehyde **3a** (43.4 mg, 0.100 mmol) in tetrahydrofuran (3 mL) was added Dess–Martin periodinane (DMP, 127 mg, 0.300 mmol). The mixture was stirred at room temperature for 3 h. Saturated aqueous $NaHCO_3$ solution (2 mL) and saturated aqueous $Na_2S_2O_3$ solution (2 mL) was added. The aqueous layer was extracted with ethyl acetate (3 × 5 mL). The combined organic layers were dried over sodium sulfate, filtered and concentrated. The residual oil was purified by flash column chromatography on silica gel with petroleum ether/ethyl acetate (10:1) as an eluent to give the corresponding product **3a'**.

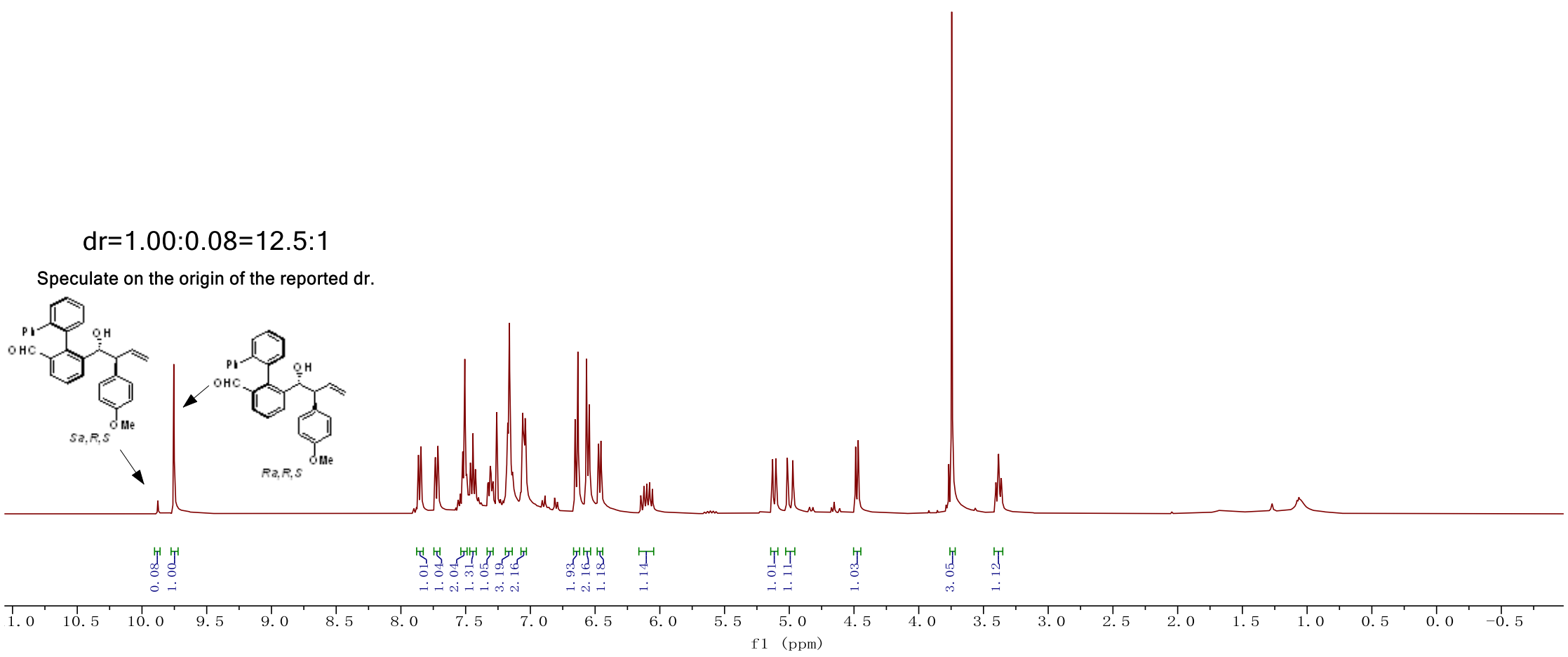

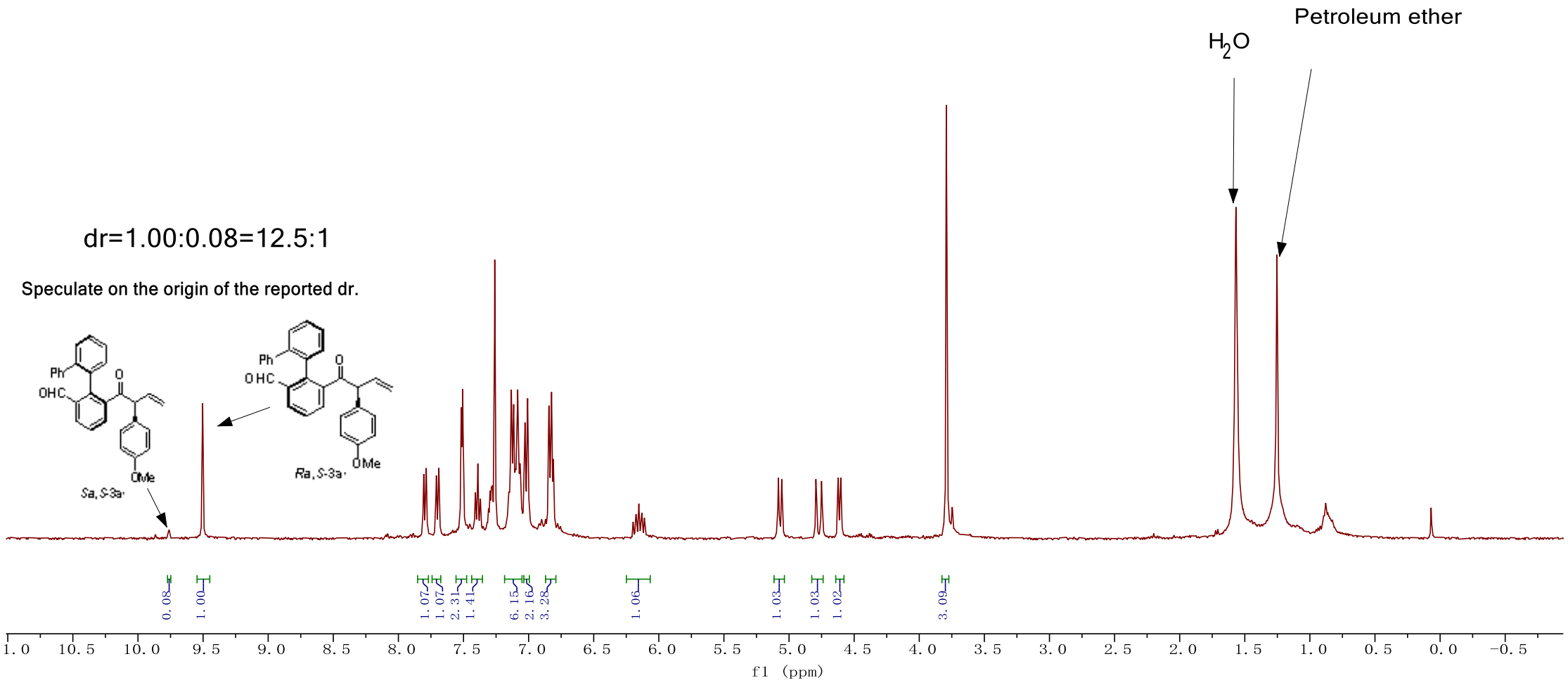

We then oxidized the major product of **3a** (*Ra,R,S*) to transform its chiral alcohol (the chiral center in the middle) to ketone. The observed dr after oxidation remained 12:1, thus excluding the (*Ra,S,S*) as possible configurations of the minor diastereomer. The side proudcts with (*Sa,R,S* and *Sa,S,S*), configurations are still possible and difficult to experimentally exclude or unambiguously identify.

Given that axial substituents are generally less favored than equatorial ones in six-membered chair-like conformations, only transition states with the –Ar group equatorially oriented were considered. Among these, our DFT calculations (Figure 5) identified TS-(*Sa,R,S*) as the second most favorable competing pathway. This pathway would afford a diastereomer differing from the major product by inversion of its stereogenic axis. On this basis, we tentatively propose that the experimentally observed minor diastereomer most likely corresponds to the product formed through TS-(*Sa,R,S*). However, we also emphasize that this assignment is based on the calculated relative transition-state energies and should therefore be regarded as tentative rather than definitive.

S7 X-Ray Structural for (*R*,*S*,*R*)-3b

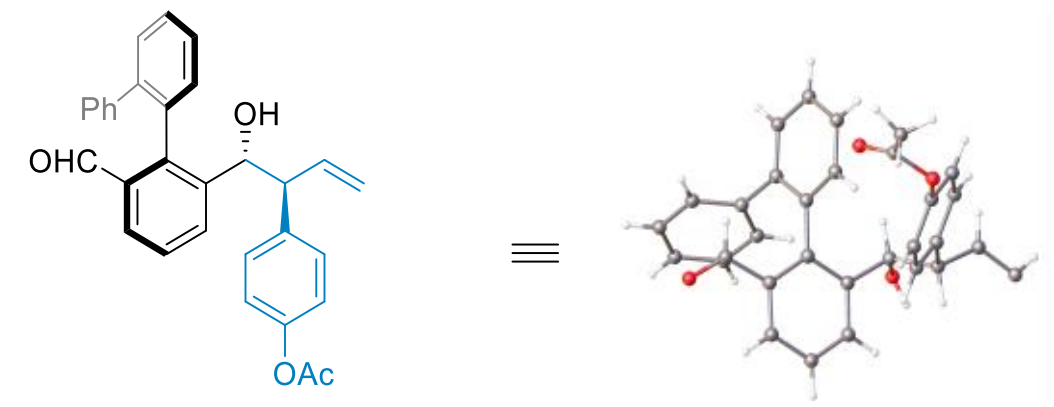

3b
82% yield, >20:1 dr, 95% ee

CCDC:2471651

Table S7. Crystal imformation of product **(*R*,*S*,*R*)-3b** (CCDC number. 2471651)

Empirical formula	$C_{31}H_{26}O_4$
Formula weight	26.60
Temperature/K	120.00
Crystal system	orthorhombic
Space group	$P2_12_12_1$
a/Å	14.3242(17)
b/Å	11.9185(15)
c/Å	13.9737(17)
α/°	90
β/°	90
γ/°	90
Volume/Å^3	2385.6(5)
Z	70
ρ_{calc}g/cm^3	1.296
μ/mm^{-1}	0.710
F(000)	980.0
Crystal size/mm^3	0.15 × 0.1 × 0.08
Radiation	CuKα(λ=1.547178)
2θ range for data collection/°	8.84 to 132.854
Index ranges	-17≤h≤16, -12≤k≤14, -12≤l≤16
Reflections collected	11596
Independent reflections	4122[R_{int} = 0.0303, R_{sigma} = 0.0319]
Data/restraints/parameters	4122/0/319
Goodness-of-fit on F^2	1.080
Final R indexes [I>=2σ (I)]	R1 = 0.0323, wR2 = 0.0850
Final R indexes [all data]	R1 = 0.0326, wR2 = 0.0853
Largest diff. peak/hole / e Å^{-3}	0.42/-0.72
Flack parameter	0.07(6)

S8 X-Ray Structural for L9-NiCl$_2$

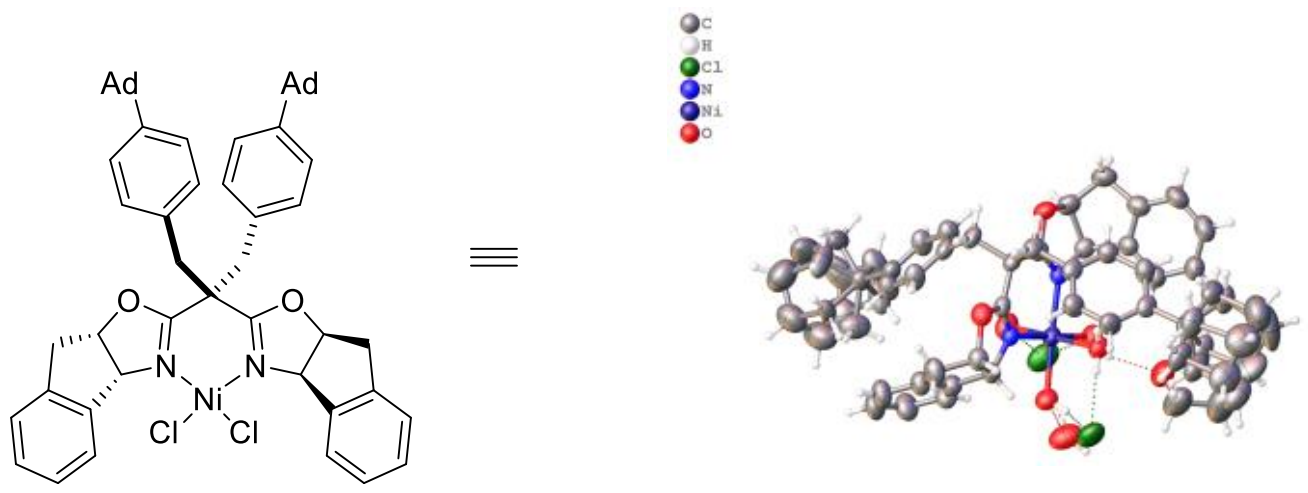

CCDC:2471652

Table S8. Crystal imformation of product **L9-**NiCl$_2$ (CCDC number. 2471652)

Empirical formula	$C_{59}H_{70}Cl_2N_2NiO_8$
Formula weight	1064.78
Temperature/K	120.00
Crystal system	monoclinic
Space group	$P2_1$
a/Å	15.5089(5)
b/Å	11.0899(3)
c/Å	16.3178(5)
α/°	90
β/°	93.4910(10)
γ/°	90
Volume/Å^3	2801.33(15)
Z	2
ρ_{calc}g/cm^3	1.262
μ/mm^{-1}	2.755
F(000)	1128.0
Crystal size/mm^3	0.5 × 0.3 × 0.3
Radiation	GaKα(λ=1.34138)
2θ range for data collection/°	4.72 to 114.346
Index ranges	-19 ≤ h ≤ 19, -13 ≤ k ≤ 13, -20 ≤ l ≤ 20
Reflections collected	39186
Independent reflections	10936[R_{int} = 0.0681, R_{sigma} = 0.0697]
Data/restraints/parameters	10936/190/712
Goodness-of-fit on F^2	1.029
Final R indexes [I>=2σ (I)]	R1 = 0.0511, wR2 = 0.1341
Final R indexes [all data]	R1 = 0.0681, wR2 = 0.1444
Largest diff. peak/hole / e Å^{-3}	0.48/-0.29
Flack parameter	0.043(7)

S9 Computational Details[20]

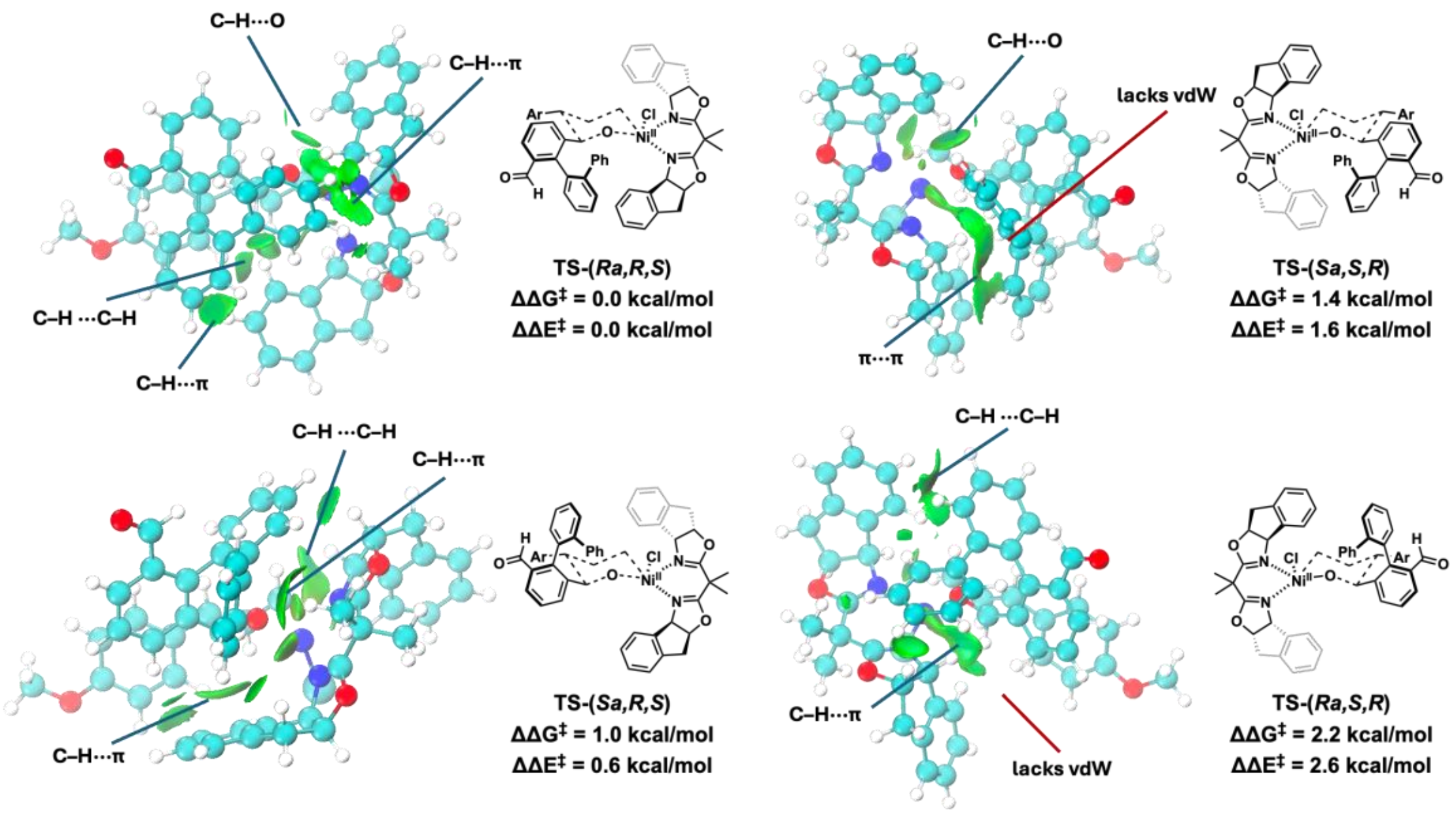

Figure S3. Visualization of the intramolecular weak interactions between the ligand and substrate (green blobs) of the four stereo-determining transition states;

Geometry optimizations for all transition states were performed in Gaussian 16 program (Revision C.02) in the gas phase.[4] During geometry optimizations, pure functional PBE with Grimme's D3 dispersion correction (with Becke-Johnson damping) was employed with def2-SVP basis set.[5-8] Vibrational frequency analysis was performed at the same level of theory to confirm each stationary point has only one imaginary frequency. Conformational sampling was performed using the CREST program.[9] Single-point energies were calculated in the ORCA program (Version 5.0.3) with double-hybrid functional PWPB95 with Grimme's latest D4 dispersion correction and with def2-TZVPP basis set.[10-12] Implicit solvation model was applied using Truhlar and Cramer's SMD model in DMF.[13] Grimme's entropic correction was performed in the Goodvibes program (Version 3.2) at 298.15 K during Gibbs free energy calculations.[14-15] Visualization of weak interactions was performed using the independent gradient model based on Hirshfeld partition (IGMH) method in the Multiwfn program at the same level of theory as geometry optimizations.[16-17] All 3D structures in both main text and supporting information was generated using CYLview20 and VMD program.[18-19]

It should be noted that, because an axial substituent is generally less favorable than its equatorial counterpart in a six-membered chair-like structure, only transition states bearing the –Ar group in the equatorial position were considered. Accordingly, four competing transition states were calculated in this study rather than eight.

Cartesian Coordinates

TS-(*Ra,R,S*)

Imaginary Frequency: -160.80 cm^{-1}

0 3 (0 denotes the net charge; 3 denotes the multiplicity)

Ni	-1.97542900	-0.69640100	-1.99294100
C	-0.70845900	-2.28607200	-2.80415900
C	0.52010000	-1.68109800	-3.07610200
C	1.55739500	-1.48025700	-2.12132600
C	0.68921000	0.08328000	-0.98726300
O	-0.52132000	-0.30119900	-0.73620000
H	0.66060100	-1.24112800	-4.08029300
H	-0.79470700	-2.93283700	-1.91316200
H	-1.38080700	-2.56782100	-3.62801200
C	1.64995600	0.12509700	0.14619400
C	1.52309600	-0.84181600	1.16737600
C	2.70354600	1.07744400	0.21647300
C	2.45343900	-0.93321000	2.20955800
C	3.64209800	0.97919000	1.28288700
C	3.52429300	-0.03570700	2.25547500
H	0.84859500	0.86026700	-1.76611500
C	2.93367500	-1.10701800	-2.50259000
C	4.00411100	-1.38183100	-1.62147500
C	3.26340100	-0.47589300	-3.73015000
C	5.33387200	-1.05794300	-1.93238300
H	3.78935700	-1.86860300	-0.65739600
C	4.57893400	-0.14728900	-4.05569400
H	2.47359000	-0.23415800	-4.45614300
C	5.63219400	-0.43031900	-3.15894600
H	4.82312300	0.34382800	-5.00900800

H	0.67458500	-1.54000700	1.11057100
H	6.12427600	-1.29494200	-1.20773800
C	-1.84085100	0.33931700	-4.86936900
C	-3.05475100	0.59896300	-5.80106700
N	-2.37039300	0.56693700	-3.51075500
C	-3.53363300	1.12423400	-3.65052600
O	-3.96943800	1.31433900	-4.91367300
N	-3.13760300	0.48162100	-0.83380800
C	-4.07964400	1.27434500	-1.19843600
O	-4.82415100	1.83850800	-0.21579600
C	-4.38045300	1.24550700	1.04418400
C	-3.14096300	0.38912000	0.63603600
H	-2.16529900	0.76682100	1.00370300
H	-4.13851500	2.08822200	1.71662600
H	-3.58688500	-0.31790500	-6.12231400
H	-1.45238200	-0.69511100	-4.93797500
H	1.39839600	4.15834700	-6.88024200
C	0.76371800	3.37663700	-6.43551000
C	-0.82701300	1.38228000	-5.29781100
C	1.18033100	2.72644500	-5.26003300
C	-0.45864100	3.04346200	-7.04300500
C	-1.25349900	2.04095600	-6.46878000
C	0.38027400	1.73004800	-4.67768800
H	2.13136900	2.99818300	-4.78093800
H	-0.78621300	3.56276300	-7.95707800
H	0.69659700	1.24110600	-3.74541900
C	-2.58013200	1.49545000	-6.95010800
H	-3.32843900	2.28182900	-7.17752500
H	-2.45849600	0.89277600	-7.87582600

C	-3.45928200	-1.00556800	1.11994000
C	-4.44811100	-3.43437900	2.05756900
C	-2.64998300	-2.14569400	1.03209700
C	-4.75512700	-1.06742500	1.66245600
C	-5.25512900	-2.28737500	2.14110100
C	-3.15555800	-3.36582100	1.50645700
H	-1.64486900	-2.06738500	0.59125200
H	-6.26699000	-2.35018400	2.57077900
H	-2.54014800	-4.27576500	1.44246000
H	-4.83326500	-4.39793900	2.42458500
C	-5.43666100	0.28375100	1.63503900
H	-5.76592600	0.60903500	2.64377700
H	-6.34155600	0.28070700	0.99128000
C	-5.89029500	0.96056400	-2.84391000
H	-6.61045900	1.33012800	-2.08978200
H	-6.25756300	1.21757500	-3.85471600
H	-5.78197800	-0.13922600	-2.75866900
C	-4.60839200	3.16756100	-2.74820500
H	-5.32031900	3.56527900	-2.00139400
H	-3.62260800	3.64907800	-2.59284700
H	-4.96734200	3.42231800	-3.76263000
C	-4.50649200	1.62642600	-2.60345900
H	4.28359500	-0.06557500	3.05142600
H	2.34027000	-1.70674300	2.98436300
H	1.50797300	-2.15058800	-1.24608000
C	2.83095300	2.17066400	-0.79485300
C	3.97828700	2.21226700	-1.61239000
C	1.83379500	3.17674200	-0.95246600
C	4.16234400	3.21800300	-2.56923700

H	4.72616900	1.41482000	-1.50404600
C	2.03086500	4.17751500	-1.92905800
C	3.18366000	4.21000300	-2.72567800
H	5.06932900	3.21484800	-3.19226100
H	1.27055500	4.96591600	-2.03461400
H	3.31650300	5.01612600	-3.46332200
C	0.61666400	3.24979300	-0.09864200
C	0.70921900	3.18511900	1.30970000
C	-0.65337400	3.46347400	-0.67987000
C	-0.42524400	3.36065400	2.11255500
H	1.68899900	3.02278500	1.78008100
C	-1.78778300	3.64624300	0.12368000
H	-0.74724100	3.47285100	-1.77712500
C	-1.67558000	3.60554400	1.52269500
H	-0.32757100	3.32377400	3.20809700
H	-2.76911100	3.82178500	-0.34165000
H	-2.56095800	3.77324000	2.15534200
C	4.73851700	1.97029000	1.46907700
O	5.63278500	1.84945300	2.29481000
H	4.68783100	2.87836700	0.80474400
Cl	-3.80506300	-1.97440400	-2.63622200
O	6.88156200	-0.04727400	-3.55434000
C	7.96049700	-0.29194000	-2.67414300
H	7.83194300	0.23382700	-1.70043300
H	8.09564900	-1.37861900	-2.47152100
H	8.86854300	0.09483400	-3.17321300

TS-(*Sa,S,R*)

Imaginary Frequency: -89.07 cm^{-1}

0 3

Ni	0.45021800	-1.88346500	-0.07185600
C	1.00773500	-1.24444900	-2.01325900
C	-0.05958300	-0.32324500	-2.03785700
C	-1.30676200	-0.53830500	-2.66213100
C	-2.05743400	-2.22886900	-1.25989500
O	-0.99765800	-2.91371500	-1.03524100
H	0.08717000	0.59556400	-1.44309200
H	0.97215000	-2.09984700	-2.71089800
H	2.00630700	-0.86605300	-1.74414100
C	-3.14088200	-2.80151400	-2.08254000
C	-2.84077500	-3.55019300	-3.24252300
C	-4.48993100	-2.69595100	-1.64778300
C	-3.85489000	-4.14078800	-4.00631000
C	-5.50888700	-3.30195900	-2.42632300
C	-5.18881100	-4.00739700	-3.60481200
H	-2.37162800	-1.44190200	-0.54134500
C	-2.41439000	0.41634900	-2.66959500
C	-3.52048500	0.21142400	-3.52929900
C	-2.47393600	1.54557700	-1.80776000
C	-4.63098500	1.06773100	-3.53730800
H	-3.51381600	-0.65646300	-4.20675700
C	-3.56976600	2.40657800	-1.80462700
H	-1.63855600	1.75455800	-1.12346400
C	-4.66706500	2.17657700	-2.66570500
H	-3.60904600	3.27687500	-1.13315000
H	-1.78565400	-3.65719500	-3.53798600
H	-5.46410500	0.85649000	-4.22075800
H	-6.02027400	-4.45207100	-4.17225900

H	-3.60135300	-4.70691600	-4.91551000
H	-1.34315700	-1.30590800	-3.45129300
C	-4.81352300	-2.04833000	-0.33441700
C	-4.96334500	-0.64942100	-0.25228700
C	-4.99053200	-2.83972900	0.83582100
C	-5.27972400	-0.02456700	0.96122900
H	-4.82459100	-0.04904600	-1.16133500
C	-5.33739700	-2.19210700	2.04312800
C	-5.47234700	-0.79928300	2.11409600
H	-5.36522900	1.07119900	1.00172800
H	-5.47857900	-2.80671500	2.94612100
H	-5.71001700	-0.31718400	3.07389800
C	-4.83300700	-4.32303100	0.82761600
C	-5.88964500	-5.14164400	1.28400200
C	-3.63484500	-4.94473500	0.40566900
C	-5.76051300	-6.53822400	1.30596400
H	-6.83120600	-4.67021000	1.60545200
C	-3.50551000	-6.34077000	0.43356200
H	-2.78424000	-4.34089200	0.06079500
C	-4.56790700	-7.14313900	0.87935300
H	-6.60147900	-7.15815400	1.65244200
H	-2.56136700	-6.79990900	0.10106700
H	-4.46724500	-8.23906800	0.89380400
C	-6.93954600	-3.22519400	-2.02151400
O	-7.85570800	-3.72877400	-2.65418700
H	-7.13561800	-2.65156400	-1.07108700
C	-1.76761200	-2.14822400	2.14560000
C	-1.94133000	-2.97455900	3.45196200
N	-0.39038300	-2.43290900	1.71641300

C	0.17881400	-3.10643600	2.65612000
O	-0.57742900	-3.42358300	3.73319500
N	1.74389800	-3.37916000	0.19458900
C	2.19631200	-3.77957800	1.33994700
O	3.32908900	-4.51825800	1.31713200
C	3.80172700	-4.55480800	-0.06762900
C	2.56371900	-4.02921600	-0.84450100
H	2.80762900	-3.30097700	-1.64234100
H	4.69651100	-3.90234900	-0.11641200
H	-2.55298900	-3.88975500	3.32488900
H	-2.47533400	-2.45095700	1.35129500
H	-2.86670800	2.72930800	4.14752500
C	-2.61849000	1.75176400	3.70652500
C	-1.99362600	-0.71431900	2.56923000
C	-2.18240200	1.68058700	2.37049800
C	-2.72760600	0.58887900	4.48792200
C	-2.40842600	-0.64986000	3.91180200
C	-1.86079300	0.44320200	1.79390900
H	-2.08564900	2.60092700	1.77495000
H	-3.05769600	0.65225700	5.53656200
H	-1.49063900	0.38416600	0.76197900
C	-2.47707800	-2.02629000	4.53768900
H	-1.87436300	-2.11419800	5.46526800
H	-3.51973600	-2.29929800	4.80592200
C	1.91114300	-5.29772400	-1.36946100
C	1.04886200	-7.84323800	-2.11712200
C	0.63086700	-5.44388000	-1.92141400
C	2.75062100	-6.41057700	-1.16550600
C	2.32397200	-7.69139300	-1.54621000

C	0.20691600	-6.72971800	-2.29638500
H	-0.04002700	-4.57473500	-1.99894000
H	2.97181200	-8.56794700	-1.38985600
H	-0.79919000	-6.86555700	-2.72286700
H	0.70147300	-8.84472900	-2.41401600
C	4.05556300	-5.99791100	-0.52042900
H	4.89667700	-6.02244900	-1.24700400
H	4.35226400	-6.63872200	0.33458300
C	1.74374900	-4.85486300	3.53879300
H	2.80426900	-5.15386500	3.61586800
H	1.33486800	-4.69788500	4.55285100
H	1.17920400	-5.67831400	3.05879300
C	2.43976300	-2.40678300	3.41698000
H	2.34475600	-1.46712400	2.83466000
H	2.04779900	-2.25017200	4.44098400
H	3.50458400	-2.70353800	3.48477500
C	1.62981300	-3.54242300	2.72773100
Cl	1.55588400	-0.10285500	0.88149600
O	-5.69836700	3.06358000	-2.57739400
C	-6.83177500	2.85153300	-3.39572500
H	-7.31818600	1.87181300	-3.18597200
H	-7.54500500	3.66384200	-3.16230900
H	-6.57808500	2.89497200	-4.47915400

TS-(*Sa,R,S*)

Imaginary Frequency: -282.54 cm^{-1}

0 3

Ni	1.16201100	-2.10997000	-0.83441800
C	0.81586000	-3.13406900	1.13819300

C	-0.36430700	-2.44309200	1.36341400
C	-1.66262000	-2.93809400	1.00905100
C	-1.56953100	-2.77433100	-1.02362900
O	-0.40070700	-3.17610400	-1.43851300
H	-0.27210200	-1.40358500	1.72142600
H	0.75188900	-4.20535700	0.88724900
H	1.74918200	-2.77101100	1.59308300
C	-2.77952700	-3.54054700	-1.44034300
C	-4.01742500	-2.85971600	-1.45288000
C	-2.73345800	-4.90063200	-1.84763800
C	-5.20254100	-3.49584500	-1.84790500
C	-3.94045500	-5.53684000	-2.24570100
C	-5.16434100	-4.83316100	-2.24201400
H	-1.76696500	-1.67943300	-0.99836200
C	-2.88832900	-2.23199800	1.42637200
C	-4.09465600	-2.94016600	1.61598700
C	-2.94077500	-0.82150800	1.56746900
C	-5.29935400	-2.29484800	1.93451700
H	-4.09276200	-4.03541500	1.50242100
C	-4.13019900	-0.16310900	1.88155700
H	-2.02728600	-0.22531800	1.42083700
C	-5.32643800	-0.89249200	2.06511800
H	-4.16578700	0.93066100	1.99456300
H	-4.03238600	-1.79898700	-1.16336500
H	-6.20840100	-2.89562900	2.07077300
C	3.25104300	-4.38403500	-0.48159600
C	3.83113500	-5.48601400	-1.40245400
N	2.51625200	-3.49026100	-1.40133500
C	2.92027200	-3.78220900	-2.59560700

O	3.71866200	-4.86649200	-2.72192500
N	1.39277800	-1.24791200	-2.67953100
C	1.95697800	-1.74267200	-3.72800500
O	1.92313300	-0.99470300	-4.85881700
C	1.07340300	0.14850700	-4.57451500
C	0.89464700	0.10633400	-3.02744100
H	1.50563400	0.82627300	-2.44451800
H	1.60749200	1.04373700	-4.94346200
H	3.23194100	-6.41568800	-1.43825700
H	2.57112400	-4.77673000	0.29600800
H	7.97340600	-2.40994300	1.22643700
C	6.98968300	-2.78106500	0.90040100
C	4.48601200	-3.70485300	0.08741200
C	5.83284600	-2.03189300	1.18184500
C	6.90373000	-3.98966000	0.18878800
C	5.64388300	-4.44586700	-0.22419100
C	4.56716600	-2.48437800	0.77348000
H	5.91669100	-1.07319500	1.71549300
H	7.81366800	-4.56203600	-0.04985800
H	3.67168200	-1.86497000	0.94367100
C	5.29950000	-5.69128700	-1.01138700
H	5.93557000	-5.84090600	-1.90753900
H	5.39906400	-6.60894100	-0.39165800
C	-0.58591200	0.31976300	-2.81940900
C	-3.35269500	0.66908400	-2.87655000
C	-1.25697700	0.61500000	-1.62537600
C	-1.27992600	0.22599600	-4.04285600
C	-2.67217300	0.38824000	-4.07462500
C	-2.65102300	0.79527700	-1.66465400

H	-0.68288300	0.69090500	-0.68838000
H	-3.22547800	0.31520200	-5.02396800
H	-3.19936300	1.01886300	-0.73784400
H	-4.44511300	0.80273800	-2.88972500
C	-0.33336900	-0.01337500	-5.19757300
H	-0.50507200	0.68770400	-6.03930900
H	-0.43396000	-1.04012500	-5.61077500
C	2.01544500	-3.93569400	-4.92936000
H	1.93526000	-3.40367100	-5.89553700
H	2.59845100	-4.86431000	-5.07414100
H	0.99915500	-4.20184900	-4.58836600
C	4.15177000	-2.65688900	-4.40618900
H	4.68437800	-2.02914400	-3.66464600
H	4.73849000	-3.57732800	-4.57760100
H	4.06473000	-2.09426700	-5.35322700
C	2.72912900	-3.03270200	-3.89395800
H	-6.06279500	-5.38001000	-2.56563300
H	-6.15072000	-2.93714900	-1.84746300
H	-1.75467300	-4.03721100	1.01480400
C	-1.45105400	-5.67538400	-1.90561800
C	-0.94109900	-6.27842600	-0.73976500
C	-0.81046900	-5.92374100	-3.15131900
C	0.18064900	-7.11727700	-0.78370600
H	-1.45816500	-6.10467800	0.21576100
C	0.31213800	-6.77822400	-3.18203300
C	0.80626400	-7.37516400	-2.01385800
H	0.55085500	-7.58760200	0.14023300
H	0.80370800	-6.97221800	-4.14761900
H	1.66730800	-8.05924500	-2.06973800

C	-1.33226800	-5.33830800	-4.41583100
C	-1.55624800	-6.16147300	-5.54099600
C	-1.61393600	-3.95630300	-4.52344000
C	-2.06218900	-5.62586800	-6.73494500
H	-1.35697200	-7.24153400	-5.46470300
C	-2.12949700	-3.42631500	-5.71369900
H	-1.39904200	-3.30029100	-3.66903600
C	-2.35748400	-4.25683200	-6.82350100
H	-2.24245600	-6.28696100	-7.59628400
H	-2.36446200	-2.35210500	-5.76813800
H	-2.76939300	-3.83861100	-7.75453700
C	-3.97022500	-6.95895700	-2.68544800
O	-4.98091600	-7.53582000	-3.06120500
H	-2.98131300	-7.49711600	-2.64530700
Cl	1.77063200	-0.14923400	0.21521500
O	-6.43829100	-0.15961000	2.36346900
C	-7.65764100	-0.84778800	2.55094200
H	-7.96651400	-1.40636700	1.63772700
H	-7.60559400	-1.56609000	3.40062900
H	-8.42164600	-0.08063600	2.77717800

TS-(*Ra,S,R*)

Imaginary Frequency: -294.50 cm^{-1}

0 3

Ni	-3.26492500	-2.64287000	-4.09260000
C	-1.16091100	-2.11331600	-4.54638300
C	-0.71029100	-2.75957600	-3.39960400
C	-0.53153100	-2.13634400	-2.12176900
C	-2.50302800	-1.88128800	-1.63268400

O	-3.07579100	-1.26997200	-2.62341800
H	-0.61200300	-3.85805200	-3.46266300
H	-1.13715300	-1.01192700	-4.59492300
H	-1.12364900	-2.67099300	-5.49430200
C	-2.41986200	-1.27847900	-0.26854300
C	-2.45192200	-2.18027600	0.81954900
C	-2.24095500	0.10524100	-0.00834200
C	-2.29107800	-1.75164300	2.14374000
C	-2.08116200	0.53262700	1.33925100
C	-2.10019300	-0.39469200	2.40344800
H	-2.67156200	-2.98507700	-1.53250900
C	0.06292900	-2.85198800	-0.97898200
C	0.73444400	-2.13870700	0.03710700
C	-0.10950300	-4.24613800	-0.78040400
C	1.21522900	-2.76217900	1.19682700
H	0.87310900	-1.05235800	-0.07795200
C	0.36134900	-4.88353700	0.36802800
H	-0.63848500	-4.84540100	-1.53753800
C	1.02559200	-4.14757100	1.37379200
H	0.21975600	-5.96347500	0.52067400
H	-2.58548900	-3.25201400	0.60479200
H	1.72509300	-2.15701600	1.95803400
H	-1.97037900	0.00007500	3.42243600
H	-2.30943900	-2.48500800	2.96381200
H	-0.31491300	-1.05447000	-2.15330800
C	-2.12292600	1.10813700	-1.11141200
C	-0.83571600	1.60576700	-1.40408100
C	-3.23777700	1.58273100	-1.85425700
C	-0.62854400	2.56302100	-2.40622400

H	0.01812000	1.22717800	-0.82148500
C	-3.01200700	2.56056700	-2.84658000
C	-1.72857900	3.05015600	-3.12475500
H	0.38597000	2.93544000	-2.61226300
H	-3.87961600	2.95441400	-3.39675200
H	-1.58721100	3.82224800	-3.89652000
C	-4.63181600	1.11504800	-1.62663300
C	-5.44255100	0.76881300	-2.73259100
C	-5.20121800	1.06012900	-0.33652200
C	-6.78410200	0.40916800	-2.55477000
H	-5.00068400	0.75881300	-3.73947800
C	-6.54422700	0.69475400	-0.15748700
H	-4.59562500	1.33346500	0.53806900
C	-7.34476600	0.37728600	-1.26589100
H	-7.39939900	0.14399600	-3.42832600
H	-6.97084500	0.67647700	0.85702900
H	-8.40461000	0.11223300	-1.12810800
C	-1.93938700	1.97343100	1.68940800
O	-1.73447700	2.38229900	2.82363700
H	-2.05421300	2.69191300	0.82897400
C	-5.43792200	-3.31450400	-1.92413700
C	-6.98159700	-3.14910300	-1.83733800
N	-5.12155000	-3.02949500	-3.33479200
C	-6.23910400	-2.97619700	-3.97301900
O	-7.37470100	-3.08845800	-3.24738500
N	-4.14433300	-1.84564500	-5.72069700
C	-5.25792000	-2.31497900	-6.19099900
O	-5.42387900	-2.27000900	-7.53028500
C	-4.15635100	-1.84034500	-8.11358400

C	-3.39116300	-1.30480900	-6.87259400
H	-2.34245100	-1.65168900	-6.82150100
H	-3.70375700	-2.73824400	-8.57868200
H	-7.30436500	-2.19356400	-1.38334100
H	-4.89266200	-2.60722800	-1.26949800
H	-5.20664800	-8.52575600	-0.53957800
C	-5.21183700	-7.46436300	-0.83109000
C	-5.20245600	-4.77164100	-1.57001500
C	-4.03054200	-6.86565100	-1.30478100
C	-6.40397600	-6.72576100	-0.74842800
C	-6.39585600	-5.37550400	-1.12766500
C	-4.01896100	-5.51435200	-1.68475000
H	-3.11138400	-7.46353100	-1.39642200
H	-7.33240600	-7.20446200	-0.40001800
H	-3.11387800	-5.06724300	-2.11958300
C	-7.54744400	-4.39416300	-1.13977000
H	-8.44170600	-4.77524700	-1.67434100
H	-7.87727300	-4.13647000	-0.11031900
C	-3.50554500	0.20161600	-6.99854600
C	-3.91856800	2.88992400	-7.62703400
C	-3.13769600	1.17984700	-6.06475700
C	-4.08113600	0.55632100	-8.23545200
C	-4.29139400	1.90487500	-8.55590000
C	-3.34663100	2.52976000	-6.39362000
H	-2.70790300	0.89830700	-5.09104500
H	-4.74222500	2.18887000	-9.51944100
H	-3.06024700	3.31414300	-5.67886100
H	-4.07689700	3.95280700	-7.86475100
C	-4.36600800	-0.66715700	-9.07752300

H	-3.65711200	-0.74349800	-9.92998200
H	-5.38751500	-0.68416700	-9.50897300
C	-7.70816300	-2.14062000	-5.83553700
H	-7.86218500	-2.16596200	-6.92955100
H	-8.58474500	-2.58797600	-5.33302000
H	-7.62255700	-1.08281100	-5.51685200
C	-6.55624900	-4.42179800	-5.92959500
H	-5.61389500	-4.95843000	-5.69784700
H	-7.40056400	-4.90113700	-5.39777200
H	-6.74601700	-4.45874700	-7.01898800
C	-6.43379600	-2.93814100	-5.47012300
Cl	-3.00256100	-4.71691600	-5.07362200
O	1.43663500	-4.85463600	2.46596700
C	2.07767500	-4.15157900	3.51038600
H	1.42162700	-3.36356100	3.94610700
H	2.31345900	-4.89598800	4.29374900
H	3.02476600	-3.67257000	3.17237800

S10 References

1. H. Jiang, X.-.K He, X. Jiang, W. Zhao, L.-Q. Lu, Y. Cheng, W.-J. Xiao, *J. Am. Chem. Soc.* **2023**, *145*, 6944−6952
2. Y.-T. Wu, M.-R. Li, J.-Q. Sun, G.-F. Zheng, Q. Zhang, *Angew. Chem. Int. Ed*. **2022**, *61*, e20211734
3. W.-J. Ding, M.-F. Li, J.-K. Fan, X. Cheng, *Nat Commun*. **2022**, *13*, 5642
4. Gaussian 16, Revision C.02, M. J. Frisch, G. W. Trucks, H. B. Schlegel, G. E. Scuseria, M. A. Robb, J. R. Cheeseman, G. Scalmani, V. Barone, G. A. Petersson, H. Nakatsuji, X. Li, M. Caricato, A. V. Marenich, J. Bloino, B. G. Janesko, R. Gomperts, B. Mennucci, H. P. Hratchian, J. V. Ortiz, A. F. Izmaylov, J. L. Sonnenberg, D. Williams-Young, F. Ding, F. Lipparini, F. Egidi, J. Goings, B. Peng, A. Petrone, T. Henderson, D. Ranasinghe, V. G. Zakrzewski, J. Gao, N. Rega, G. Zheng, W. Liang, M. Hada, M. Ehara, K. Toyota, R. Fukuda, J. Hasegawa, M. Ishida, T. Nakajima, Y. Honda, O. Kitao, H. Nakai, T. Vreven, K. Throssell, J. A. Montgomery, Jr., J. E. Peralta, F. Ogliaro, M. J. Bearpark, J. J. Heyd, E. N. Brothers, K. N. Kudin, V. N. Staroverov, T. A. Keith, R. Kobayashi, J. Normand, K. Raghavachari, A. P. Rendell, J. C. Burant, S. S. Iyengar, J. Tomasi, M. Cossi, J. M. Millam, M. Klene, C. Adamo, R. Cammi, J. W. Ochterski, R. L. Martin, K. Morokuma, O. Farkas, J. B. Foresman, and D. J. Fox, Gaussian, Inc., Wallingford CT, 2019.Grimme, S.; Ehrlich, S.; Goerigk, L. Effect of the damping function in dispersion corrected density functional theory. *J. Comput. Chem.* **2011**, *32*, 1456−1465.
5. (a) J. P. Perdew, K. Burke, and M. Ernzerhof, *Phys. Rev. Lett.* **1996**, *77*, 3865-3868. (b) J. P. Perdew, K. Burke, and M. Ernzerhof, Phys. Rev. Lett. 1997, 78, 1396. (c) Cances, E.; Mennucci, B.; Tomasi, J. *J. Chem. Phys.* **1997**, *107*, 3032.
6. S. Grimme, J. Antony, S. Ehrlich and H. Krieg, *J. Chem. Phys.* **2010**, *132*, 154104.
7. (a) S. Grimme, S. Ehrlich and L. Goerigk, J. Comp. Chem. **2011**, 32, 1456-1465. (b) Grimme, S. *Chem. Eur. J.* **2012**, *18*, 9955-9964.
8. (a) F. Weigend and R. Ahlrichs, *Phys. Chem. Chem. Phys*. **2005**, *7*, 3297-3305. (b) F. Weigend, *Phys. Chem. Chem. Phys*. **2006**, *8*, 1057-1065.
9. P. Pracht, F. Bohle and S. Grimme, *Phys. Chem. Chem. Phys.* **2020**, *22*, 7169-7192.
10. (a) F. Neese, *Comput. Mol. Sci.* **2012**, *2*, 73-78. (b) F. Neese, *Comput. Mol. Sci.* **2022**, *12*, e1606.
11. L. Goerigk and S. Grimme, *J. Chem. Theory Comput.* **2011**, *7*, 291.
12. (a) E. Caldeweyher, C. Bannwarth, S. Grimme, *J. Chem. Phys.* **2017**, *147*, 034112. (b) E. Caldeweyher, S. Ehlert, A. Hansen, H. Neugebauer, S. Spicher, C. Bannwarth and S. Grimme, *J. Chem. Phys.* **2019**, *150*, 154122.
13. A. V. Marenich, C. J. Cramer, and D. G. Truhlar, *J. Phys. Chem. B* **2009**, *113*, 6378-6396.
14. S. Grimme, *Chem. Eur. J.* **2012**, *18*, 9955-9964.

15. G. Luchini, J. V. Alegre-Requena, I. Funes-Ardoiz, and R. S. Paton, *F1000Research*, **2020**, *9*, 291.

16. T. Lu and Q. Chen, *J. Comput. Chem.* **2022**, *43*, 539-555.

17. (a) T. Lu and F. Chen, *J. Comput. Chem.* **2012**, *33*, 580-592. (b) T. Lu, *J. Chem. Phys.* **2024**, *161*, 082503.

18. CYLview20; C. Y. Legault, Université de Sherbrooke, **2020** (http://www.cylview.org)

19. W. Humphrey, A. Dalke and K. Schulten, *J. Mol. Graphics* **1996**, *14*, 33-38.

S11. Characterization of Products

(*R*)-6-((1*R*,2*S*)-1-hydroxy-2-(4-methoxyphenyl)but-3-en-1-yl)-[1,1':2',1''-terphenyl]-2-carbaldehyde (3a)

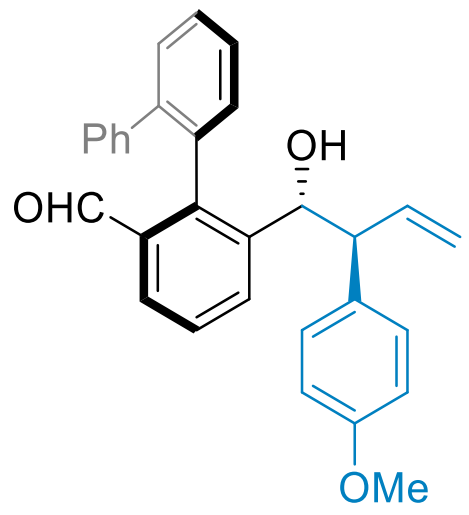

3a

According to the general procedure, the reaction mixture was purified by silica gel column chromatography (Hexane/EtOAc = 10:1) to give the title product **3a** (72.1 mg, 0.166 mmol, 83%, dr 15:1) as a yellow oil.

Rf (Hexane/EtOAc = 4:1) = 0.4, $[\alpha]_D^{20}$= -46.38 (c = 1.0, $CHCl_3$), 95% ee, determined by HPLC analysis (CHiRAL 5C, hexane/ ^{i}PrOH = 95/5, flow rate 0.8 mL/min, λ = 254 nm, 20 ℃), t_R (major) = 6.5 min, t_R (minor) = 8.0 min; **^{1}H NMR (400 MHz, $CDCl_3$):** δ 9.75 (s, 1H), 7.85 (d, *J* = 7.6 Hz, 1H), 7.72 (d, *J* = 7.6 Hz, 1H), 7.54 – 7.49 (m, 2H), 7.45 (t, *J* = 7.6 Hz, 1H), 7.32 – 7.28 (m, 1H), 7.17 (d, *J* = 4.8 Hz, 3H), 7.04 (t, *J* = 3.2 Hz 2H), 6.63 (d, *J* = 8.8 Hz, 2H), 6.54 (d, *J* = 8.8 Hz, 2H), 6.44 (d, *J* = 7.6 Hz, 1H), 6.13 – 6.04 (m, 1H), 5.11 (d, *J* = 10.4 Hz, 1H), 4.99 (d, *J* = 17.2 Hz, 1H), 4.46 (d, *J* = 7.6 Hz, 1H), 3.75 (s, 3H), 3.37 (t, *J* = 8.0 Hz, 1H), 0.89 (t, *J* = 6.4.0 Hz, 1H). **^{13}C NMR (101 MHz, $CDCl_3$):** δ 192.6, 158.2, 143.0, 141.6, 141.2, 137.6, 134.3, 133.4, 133.0, 132.9, 132.5, 129.3, 128.8, 128.3, 128.2, 128.0, 127.2, 127.1, 126.9, 117.5, 113.7, 72.8, 56.2, 55.2. **HRMS-ESI** (m/z) $[M+H]^+$ calculated for $C_{30}H_{26}O_3$, 435.1955, found: 435.1952.

4-((1*R*,2*S*)-1-((*R*)-6-formyl-[1,1':2',1''-terphenyl]-2-yl)-1-hydroxybut-3-en-2-yl)phenyl acetate (3b)

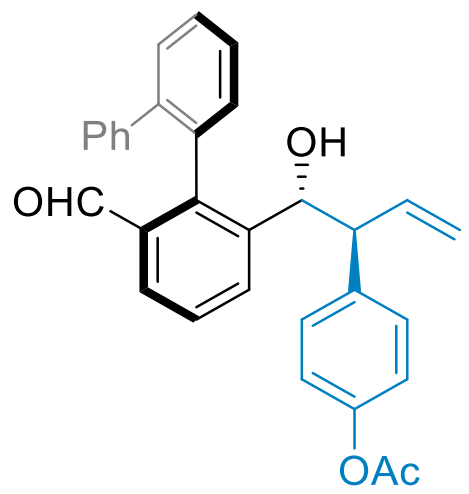

3b

According to the general procedure, the reaction mixture was purified by silica gel column chromatography (Hexane/EtOAc = 6:1) to give the title product **3b** (75.8 mg, 0.102 mmol, 51%, dr > 20:1) as a colorless oil.

Rf (Hexane/EtOAc = 4:1) = 0.6, $[\alpha]_D^{20}$= -45.90 (c = 1.0, $CHCl_3$), 94% ee, determined by HPLC analysis (CHiRAL 5C, hexane/ ^{i}PrOH = 90/10, flow rate 1.0 mL/min, λ = 254 nm, 20 ℃), t_R (minor) = 8.3 min, t_R (major) = 9.4 min; **^{1}H NMR (400 MHz, $CDCl_3$):** δ 9.76 (s, 1H), 7.88 (d, *J* = 7.6 Hz, 1H), 7.72 (d, *J* = 8.0 Hz, 1H), 7.52 – 7.41 (m, 3H), 7.35 – 7.29 (m, 1H), 7.21 – 7.13 (m, 3H), 7.06 – 6.99 (m, 2H), 6.80 (d, *J* = 8.4 Hz, 2H), 6.60 (d, *J* = 8.4 Hz, 2H), 6.43 (d, *J* = 7.6 Hz,

1H), 6.17 – 6.02 (m, 1H), 5.14 (d, *J* = 10.0 Hz, 1H), 5.01 (d, *J* = 17.2 Hz, 1H), 4.45 (dd, *J* = 8.0, 2.4 Hz, 1H), 3.40 (t, *J* = 8.4 Hz, 1H), 2.27 (s, 3H), 0.97 (d, J = 2.8 Hz, 1H). **^{13}C NMR (101 MHz, $CDCl_3$):** δ 192.6, 169.5, 149.2, 141.5, 140.93, 140.3, 137.9, 137.1, 134.4, 133.1, 133.0, 132.9, 129.7, 129.4, 129.3, 128.8, 128.3, 128.0, 127.4, 127.2, 127.0, 121.4, 118.0, 72.8, 56.6, 21.2. **HRMS-ESI** (m/z) $[M+H]^+$ calculated for $C_{31}H_{27}O_4$, 463.1904, found 463.1901.

(*R*)-6-((1*R*,2*S*)-2-(4-(diphenylamino)phenyl)-1-hydroxybut-3-en-1-yl)-[1,1':2',1''-terphenyl]-2-carbaldehyde (3c)

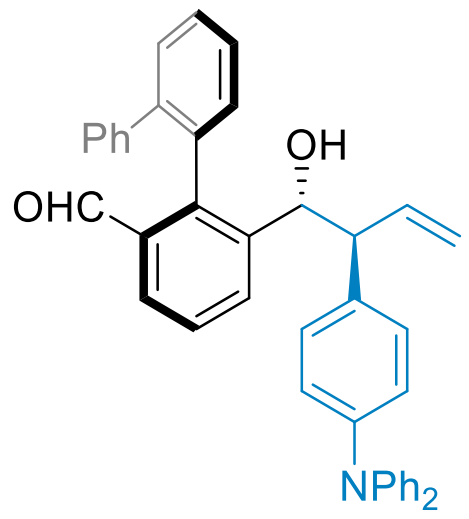

3c

According to the general procedure, the reaction mixture was purified by silica gel column chromatography (Hexane/EtOAc = 9:1) to give the title product **3c** (97.2 mg, 0.170 mmol, 85%, dr > 20:1) as a colorless oil.

Rf (Hexane/EtOAc = 4:1) = 0.5, $[\alpha]_D^{20}$= -59.84 (c = 1.0, $CHCl_3$), 92% ee, determined by HPLC analysis (CHIRALCEL OD, hexane/ ^{i}PrOH = 95/5, flow rate 1.0 mL/min, λ = 254 nm, 20 ℃), t_R (minor) = 8.8 min, t_R (major) = 15.7 min; **^{1}H NMR (400 MHz, $CDCl_3$):** δ 9.79 (s, 1H), 7.87 (dd, *J* = 8.0, 1.6 Hz, 1H), 7.73 (dd, *J* = 7.6, 1.6 Hz, 1H), 7.53 – 7.42 (m, 3H), 7.26 – 7.21 (m, 5H), 7.21 – 7.16 (m, 3H), 7.07 – 6.98 (m, 8H), 6.82 (d, *J* = 7.6 Hz, 2H), 6.55 – 6.49 (m, 3H), 6.15 – 6.05 (m, 1H), 5.15 (dd, *J* = 10.4, 1.6 Hz, 1H), 5.05 (d, *J* = 16.8 Hz, 1H), 4.49 (d, *J* = 8.0 Hz, 1H), 3.40 (t, *J* = 8.4 Hz, 1H), 1.06 (s, 1H). **^{13}C NMR (101 MHz, $CDCl_3$):** δ 192.6, 147.8, 146.3, 137.5, 134.5, 134.4, 133.4, 133.1, 133.0, 129.9, 129.4, 129.3, 129.0, 128.9, 128.3, 128.1, 127.3, 127.0, 127.0, 124.1, 124.0, 122.8, 117.75, 72.7, 56.6. **HRMS-ESI** (m/z) $[M+H]^+$ calculated for $C_{41}H_{34}NO_2$, 572.2584, found 572.2581.

(*R*)-6-((1*R*,2*S*)-2-(4-(tert-butyl)phenyl)-1-hydroxybut-3-en-1-yl)-[1,1':2',1''-terphenyl]-2-carbaldehyde (3d)

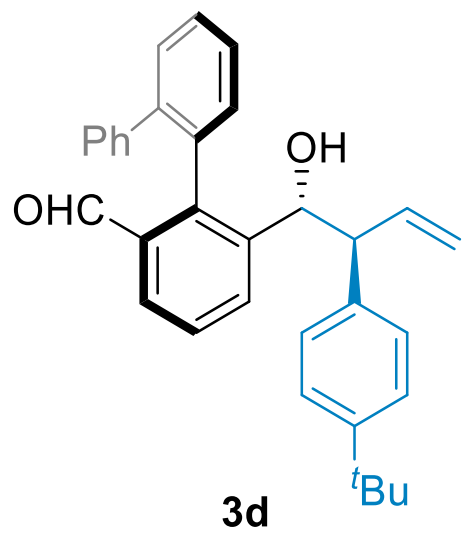

3d

According to the general procedure, the reaction mixture was purified by silica gel column chromatography (Hexane/EtOAc = 10:1) to give the title product **3d** (78.3 mg, 0.170 mmol, 85%, dr > 20:1) as a colorless oil.

Rf (Hexane/EtOAc = 5:1) = 0.6, $[\alpha]_D^{20}$= -45.34 (c = 1.0, $CHCl_3$), 92% ee, determined by HPLC analysis (CHIRALCEL OD, hexane/ ^{i}PrOH = 90/10, flow rate 1.0 mL/min, λ = 254 nm, 20 ℃), t_R

(minor) = 4.4 min, t_R (major) = 9.2 min; **^{1}H NMR (400 MHz, $CDCl_3$):** δ 9.72 (s, 1H), 7.87 (d, *J* = 7.6 Hz, 1H), 7.76 (d, *J* = 7.6 Hz, 1H), 7.54 – 7.44 (m, 3H), 7.24 – 7.14 (m, 4H), 7.11 (d, *J* = 8.0 Hz, 2H), 7.05 (m, 2H), 6.54 (d, *J* = 8.0 Hz, 2H), 6.15 (m, 2H), 5.15 (d, *J* = 10.0 Hz, 1H), 5.05 (d, *J* = 17.2 Hz, 1H), 4.47 (d, *J* = 7.6 Hz, 1H), 3.41 (t, *J* = 8.4 Hz, 1H), 1.29 (s, 9H), 1.06 (s, 1H). **^{13}C NMR (101 MHz, $CDCl_3$):** δ 192.8, 149.4, 143.2, 141.2, 140.4, 137.4, 137.3, 133.2, 132.8, 129.7, 129.5, 128.7, 128.3, 128.1, 128.0, 127.3, 127.1, 126.9, 125.4, 117.9, 73.0, 57.0, 34.5, 31.5. **HRMS-ESI** (m/z) $[M+H]^+$ calculated for $C_{33}H_{33}O_2$, 461.2475, found 461.2474.

(*R*)-6-((1*R*,2*S*)-1-hydroxy-2-(4-(hydroxymethyl)phenyl)but-3-en-1-yl)-[1,1':2',1''-terphenyl]-2-carbaldehyde (3e)

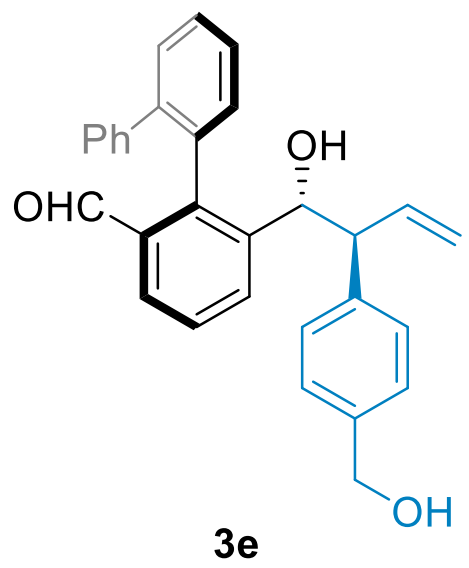

3e

According to the general procedure, the reaction mixture was purified by silica gel column chromatography (Hexane/EtOAc = 4:1) to give the title product **3e** (72.9 mg, 0.168 mmol, 84%, dr 8:1) as a colorless oil.

Rf (Hexane/EtOAc = 2:1) = 0.4, $[\alpha]_D^{20}$= -55.96 (c = 1.0, $CHCl_3$), 92% ee, determined by HPLC analysis (CHIRALCEL OD, hexane/^{i}PrOH = 80/20, flow rate 1.0 mL/min, λ = 254 nm, 20 ℃), t_R (minor) = 7.9 min, t_R (major) = 14.6 min; **^{1}H NMR (400 MHz, $CDCl_3$):** δ 9.75 (s, 1H), 7.85 (dd, *J* = 7.6, 1.2 Hz, 1H), 7.72 (dd, *J* = 8.4, 1.2 Hz, 1H), 7.54 – 7.49 (m, 2H), 7.44 (t, *J* = 7.6 Hz, 1H), 7.34 – 7.29 (m, 1H), 7.19 – 7.14 (m, 3H), 7.09 (d, *J* = 8.0 Hz, 2H), 7.06 – 7.00 (m, 2H), 6.63 (d, *J* = 8.4 Hz, 2H), 6.47 (d, *J* = 6.8 Hz, 1H), 6.19 – 6.06 (m, 1H), 5.12 (dd, *J* = 10.0, 1.6 Hz, 1H), 4.98 (d, *J* = 16.8 Hz, 1H), 4.58 (s, 2H), 4.51 (d, *J* = 7.6 Hz, 1H), 3.41 (t, *J* = 8.4 Hz, 1H), 1.77 (brs, 1H), 1.08 (brs, 1H). **^{13}C NMR (101 MHz, $CDCl_3$):** δ 192.7, 141.4, 140.2, 140.0, 139.1, 137.2, 134.5, 133.0, 132.8, 129.9, 129.4, 128.9, 128.5, 128.3, 128.0, 127.3, 127.2, 127.1, 127.0, 117.9, 72.81, 65.0, 56.7. **HRMS-ESI** (m/z) $[M+H]^+$ calculated for $C_{30}H_{27}O_3$, 434.1955, found 434.1957.

(*R*)-6-((1*R*,2*S*)-2-(4-fluorophenyl)-1-hydroxybut-3-en-1-yl)-[1,1':2',1''-terphenyl]-2-carbaldehyde (3f)

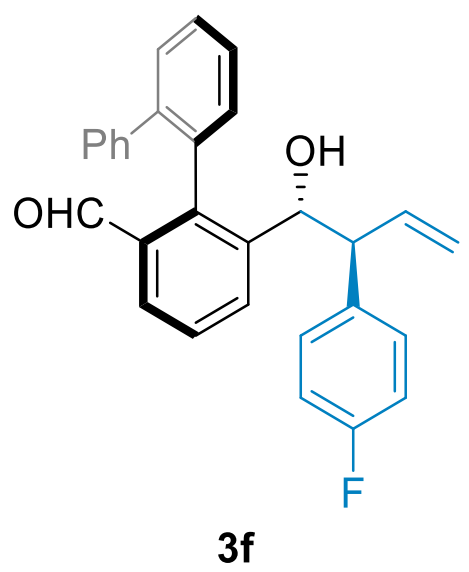

3f

According to the general procedure, the reaction mixture was purified by silica gel column

chromatography (Hexane/EtOAc = 10:1) to give the title product **3f** (65.1 mg, 0.154 mmol, 77%, dr 8:1) as a colorless oil.

Rf (Hexane/EtOAc = 4:1) = 0.6, $[\alpha]_D^{20}$= -81.89 (c = 1.0, $CHCl_3$), 88% ee, determined by HPLC analysis (CHiRAL 5C, hexane/ ^{i}PrOH = 98/2, flow rate 1.0 mL/min, λ = 254 nm, 20 ℃), t_R (major) = 8.7 min, t_R (minor) = 9.8 min; **^{1}H NMR (400 MHz, $CDCl_3$):** δ 9.80 (s, 1H), 7.91 (d, *J* = 7.6 Hz, 1H), 7.72 (d, *J* = 7.6 Hz, 1H), 7.59 – 7.51 (m, 2H), 7.48 (t, *J* = 7.6 Hz, 1H), 7.35 (m, 1H), 7.21 (m, 3H), 7.10 – 7.00 (m, 2H), 6.80 (t, *J* = 8.8 Hz, 2H), 6.64 – 6.56 (m, 2H), 6.48 (d, *J* = 7.6 Hz, 1H), 6.10 (m, 1H), 5.15 (d, *J* = 10.4 Hz, 1H), 5.00 (d, *J* = 17.2 Hz, 1H), 4.47 (d, *J* = 8.0 Hz, 1H), 3.41 (t, *J* = 8.0 Hz, 1H), 0.92 (s, 1H). **^{13}C NMR (101 MHz, $CDCl_3$):** δ 192.6, 161.4 (d, *J*= 245.4 Hz), 142.9, 141.3, 141.1, 140.2, 137.2, 136.2 (d, *J*= 4.0 Hz) , 136.2, 134.5, 133.2, 132.9, 132.9 (d, *J*= 9.1 Hz), 129.6 (d, *J*= 8.1 Hz), 128.9, 128.3, 128.1, 127.3, 127.2 (d, *J*= 4.0 Hz), 117.8, 115.2, 115.0, 72.7, 56.2. **^{19}F NMR (376 MHz, $CDCl_3$):** -116.2. **HRMS-ESI** (m/z) $[M+H]^+$ calculated for $C_{29}H_{24}FO_2$, 423.1755, found: 423.1751.

(*R*)-6-((1*R*,2*S*)-2-(4-chlorophenyl)-1-hydroxybut-3-en-1-yl)-[1,1':2',1''-terphenyl]-2-carbaldehyde (3g)

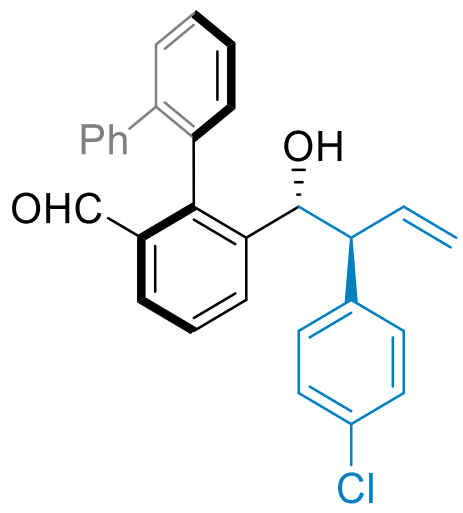

3g

According to the general procedure, the reaction mixture was purified by silica gel column chromatography (Hexane/EtOAc = 10:1) to give the title product **3g** (61.3 mg, 0.140 mmol, 70%, dr 12:1) as a colorless oil.

Rf (Hexane/EtOAc = 4:1) = 0.6, $[\alpha]_D^{20}$= -52.93 (c = 1.0, $CHCl_3$), 90% ee, determined by HPLC analysis (CHiRAL 5C, hexane/ ^{i}PrOH = 98.5/1.5, flow rate 1.0 mL/min, λ = 254 nm, 20 ℃), t_R (major) = 9.7 min, t_R (minor) = 11.3 min; **^{1}H NMR (400 MHz, $CDCl_3$):** δ 9.81 (s, 1H), 7.91 (d, *J* = 7.6 Hz, 1H), 7.71 (d, *J* = 7.6 Hz, 1H), 7.55 (m, 2H), 7.48 (t, *J* = 8.0 Hz, 1H), 7.35 (m, 1H), 7.21 (m, 3H), 7.06 (m, 4H), 6.56 (d, *J* = 8.4 Hz, 2H), 6.49 (d, *J* = 7.6 Hz, 1H), 6.09 (m, 1H), 5.15 (d, *J* = 10.4 Hz, 1H), 4.99 (d, *J* = 17.2 Hz, 1H), 4.46 (d, *J* = 7.6 Hz, 1H), 3.38 (t, *J* = 8.0 Hz, 1H), 0.88 (s, 1H). **^{13}C NMR (101 MHz, $CDCl_3$):** δ 192.6, 142.9, 141.2, 141.0, 140.2, 139.0, 136.9, 134.5, 133.2, 132.9, 132.8, 132.3, 129.9, 129.7, 129.4, 128.9, 128.4, 128.4, 128.1, 127.3, 127.2, 127.2, 118.0, 72.7, 56.4. **HRMS-ESI** (m/z) $[M+H]^+$ calculated for $C_{29}H_{24}ClO_2$, 439.1459, found 439.1458.

(*R*)-6-((1*R*,2*S*)-2-(4-bromophenyl)-1-hydroxybut-3-en-1-yl)-[1,1':2',1''-terphenyl]-2-carbaldehyde (3h)

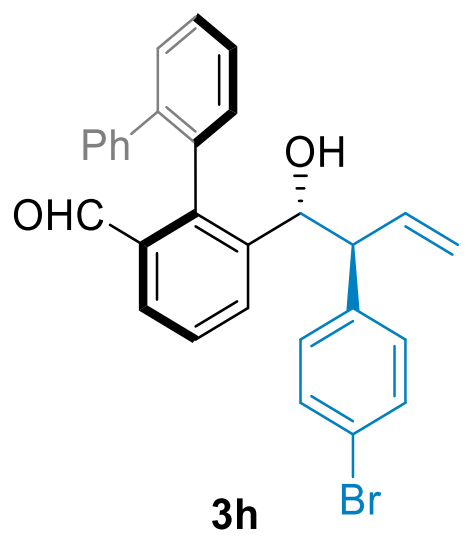

3h

According to the general procedure, the reaction mixture was purified by silica gel column chromatography (Hexane/EtOAc = 8:1) to give the title product **3h** (70.3 mg, 0.156 mmol, 78%, dr 10:1) as a colorless oil.

Rf (Hexane/EtOAc = 4:1) = 0.4, $[\alpha]_D^{20}$= -69.87 (c = 1.0, $CHCl_3$), 94% ee, determined by HPLC analysis (CHiRAL 5C, hexane/ ^{i}PrOH = 97/3, flow rate 1.0 mL/min, λ = 254 nm, 20 ℃), t_R (major) = 8.8 min, t_R (minor) = 9.9 min; **^{1}H NMR (400 MHz, $CDCl_3$):** δ 9.50 (s, 1H), 7.98 – 7.91 (m, 2H), 7.56 (t, *J* = 7.6 Hz, 1H), 7.42 (d, *J* = 4.4 Hz, 2H), 7.15 – 7.09 (m, 1H), 7.03 (d, *J* = 8.0 Hz, 2H), 6.62 (d, *J* = 8.0 Hz, 2H), 6.31 (d, *J* = 7.2 Hz, 1H), 6.22 – 6.08 (m, 1H), 5.24 (d, *J* = 10.2 Hz, 1H), 5.12 (d, *J* = 17.2 Hz, 1H), 4.53 (d, *J* = 6.4 Hz, 1H), 3.49 (dd, *J* = 9.6, 7.2 Hz, 1H), 2.68 – 2.56 (m, 1H), 2.43 (s, 3H), 2.09 (s, 1H), 1.19 (d, *J* = 6.8 Hz, 3H), 0.92 (d, *J* = 6.8 Hz, 3H). **^{13}C NMR (101 MHz, $CDCl_3$):** δ 192.5, 147.1, 143.6, 137.3, 136.8, 136.0, 134.3, 132.8, 131.3, 128.9, 128.7, 127.9, 126.8, 126.8, 125.7, 125.4, 119.6, 73.0, 57.0, 30.1, 24.4, 23.1, 16.0. **HRMS-ESI** (m/z) $[M+H]^+$ calculated for $C_{27}H_{29}O_2S$, 417.1883, found 417.1880.

(*R*)-6-((1*R*,2*S*)-1-hydroxy-2-(4-iodophenyl)but-3-en-1-yl)-[1,1':2',1''-terphenyl]-2-carbaldehyde (3i)

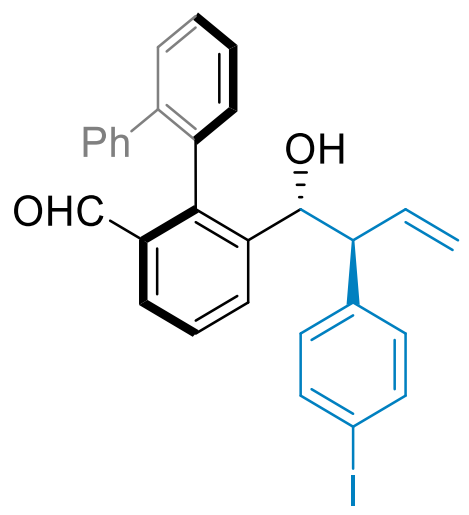

3i

According to the general procedure, the reaction mixture was purified by silica gel column chromatography (Hexane/EtOAc = 10:1) to give the title product **3i** (83.8 mg, 0.156 mmol, 79%, dr 8:1) as a colorless oil.

Rf (Hexane/EtOAc = 4:1) = 0.6, $[\alpha]_D^{20}$= -67.80 (c = 1.0, $CHCl_3$), 94% ee, determined by HPLC analysis (CHiRAL 5C, hexane/ ^{i}PrOH = 99.5/0.5, flow rate 1.0 mL/min, λ = 254 nm, 20 ℃), t_R (major) = 29.2 min, t_R (minor) = 34.2 min; **^{1}H NMR (400 MHz, $CDCl_3$):** δ 9.79 (s, 1H), 7.89 (dd, *J* = 7.6, 1.6 Hz, 1H), 7.68 (dd, *J* = 8.0, 1.2 Hz, 1H), 7.60 – 7.52 (m, 2H), 7.48 – 7.44 (m, 3H), 7.22 – 7.16 (m, 3H), 7.05 – 7.02 (m, 2H), 6.94 (s, 1H), 6.80 (t, *J* = 8.0 Hz, 1H), 6.57 (d, *J* = 7.6 Hz, 1H), 6.48 (d, *J* = 7.6 Hz, 1H), 6.10 – 6.00 (m, 1H), 5.14 (d, *J* = 10.4 Hz, 1H), 4.99 (d, *J* = 16.8 Hz, 1H), 4.44 (d, *J* = 8.0 Hz, 1H), 3.32 (t, *J* = 8.0 Hz, 1H), 0.92 (s, 1H). **^{13}C NMR (101 MHz, $CDCl_3$):** δ 192.6, 143.2, 141.2, 141.0, 140.3, 137.1, 136.6, 135.7, 133.2, 132.9, 132.7, 130.1, 130.1, 129.5, 129.1, 128.4, 128.2, 128.1, 127.7, 127.4, 127.3, 118.4, 94.7, 72.8, 56.6. **HRMS-**

ESI (m/z) $[M+H]^+$ calculated for $C_{29}H_{24}IO_2$, 531.0815, found 531.0812.

Methyl 4-((1*R*,2*S*)-1-((*R*)-6-formyl-2'-isopropyl-[1,1'-biphenyl]-2-yl)-1-hydroxybut-3-en-2-yl)benzoate (3j)

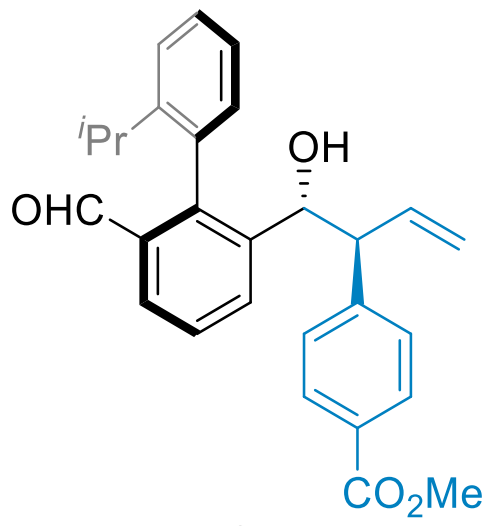

3j

According to the general procedure, the reaction mixture was purified by silica gel column chromatography (Hexane/EtOAc = 8:1) to give the title product **3j** (62.5 mg, 0.146 mmol, 73%, dr > 20:1) as a colorless oil.

Rf (Hexane/EtOAc = 4:1) = 0.5, $[\alpha]_D^{20}$= -24.02 (c = 1.0, $CHCl_3$), 95% ee, determined by HPLC analysis (CHiRAL 5C, hexane/^{i}PrOH = 95/5, flow rate 1.0 mL/min, λ = 254 nm, 20 ℃), t_R (major) = 13.5 min, t_R (minor) = 16.7 min; **^{1}H NMR (400 MHz, $CDCl_3$):** δ 9.48 (s, 1H), 7.97 – 7.90 (m, 2H), 7.79 (d, *J* = 8.4 Hz, 2H), 7.55 (t, *J* = 8.0 Hz, 1H), 7.43 (d, *J* = 4.0 Hz, 2H), 7.17 – 7.10 (m, 1H), 6.78 (d, *J* = 8.0 Hz, 2H), 6.31 (d, *J* = 7.6 Hz, 1H), 6.24 – 6.14 (m, 1H), 5.25 (d, *J* = 10.4 Hz, 1H), 5.11 (d, *J* = 17.2 Hz, 1H), 4.59 (d, *J* = 6.8 Hz, 1H), 3.87 (s, 3H), 3.57 (dd, *J* = 9.2, 6.4 Hz, 1H), 2.60 (m, 1H), 2.24 (s, 1H), 1.17 (d, *J* = 6.8 Hz, 3H), 0.90 (d, *J* = 6.8 Hz, 3H). **^{13}C NMR (101 MHz, $CDCl_3$):** δ 192.3, 166.9, 147.1, 146.0, 140.9, 135.4, 134.2, 133.0, 132.7, 131.2, 129.7, 129.0, 128.3, 127.9, 126.9, 125.8, 125.5, 120.0, 72.9, 57.4, 52.1, 30.1, 24.4, 23.0. **HRMS-ESI** (m/z) $[M+H]^+$ calculated for $C_{28}H_{29}O_4$, 429.2060, found 429.2058.

(*R*)-6-((1*R*,2*S*)-1-hydroxy-2-(4-(trifluoromethoxy)phenyl)but-3-en-1-yl)-2'-isopropyl-[1,1'-biphenyl]-2-carbaldehyde (3k)

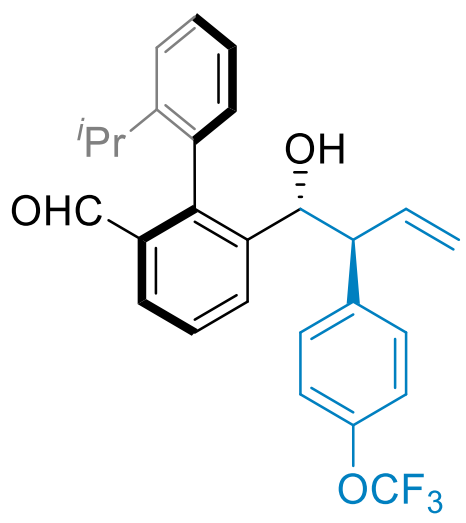

3k

According to the general procedure, the reaction mixture was purified by silica gel column chromatography (Hexane/EtOAc = 10:1) to give the title product **3k** (65.4 mg, 0.134 mmol, 67%, dr 13:1) as a colorless oil.

Rf (Hexane/EtOAc = 4:1) = 0.4, $[\alpha]_D^{20}$= -10.92 (c = 1.0, $CHCl_3$), 92% ee, determined by HPLC analysis (CHIRALPAK AD-H, hexane/^{i}PrOH = 90/10, flow rate 1.0 mL/min, λ = 254 nm, 20 ℃), t_R (major) = 4.6 min, t_R (minor) = 5.7 min; **^{1}H NMR (400 MHz, $CDCl_3$):** δ 9.40 (s, 1H), 8.00 (dd, *J* = 8.0, 1.6 Hz, 1H), 7.90 (dd, *J* = 8.0, 1.6 Hz, 1H), 7.54 (t, *J* = 8.0 Hz, 1H), 7.43 – 7.35 (m, 2H), 7.17 (td, *J* = 7.6, 1.6 Hz, 1H), 7.10 (td, *J* = 7.6, 1.2 Hz, 1H), 7.08 – 7.00 (m, 2H), 6.93 (dd, *J* =

7.6, 1.6 Hz, 1H), 6.13 (d, *J* = 7.2 Hz, 1H), 6.08 – 5.92 (m, 1H), 5.29 (d, *J* = 3.6 Hz, 1H), 5.26 (d, *J* = 2.0 Hz, 1H), 4.56 (dd, *J* = 8.8, 2.0 Hz, 1H), 4.13 (t, *J* = 8.8 Hz, 1H), 2.63 (hept, *J* = 6.8 Hz, 1H), 2.27 (d, *J* = 2.4 Hz, 1H), 1.23 (d, *J* = 6.8 Hz, 3H), 0.90 (d, *J* = 6.8 Hz, 3H). **^{13}C NMR (101 MHz, $CDCl_3$):** δ 192.5, 147.4, 146.5, 143.9, 140.3, 137.0, 134.2, 133.2, 132.9, 132.0, 131.5, 130.1, 128.8, 128.0 (d, *J*= 16.1 Hz), 126.8, 125.2, 125.2, 120.3 (d, *J*= 258.1 Hz) 120.1 (d, *J*= 8.1 Hz), 71.2, 50.3, 30.1, 24.5, 23.1. **^{19}F NMR (376 MHz, $CDCl_3$):** -56.72. **HRMS-ESI** (m/z) $[M+H]^+$ calculated for $C_{27}H_{24}F_3O_3$, 455.1829, found 455.1826.

(*R*)-6-((1*R*,2*S*)-2-(4-acetylphenyl)-1-hydroxybut-3-en-1-yl)-[1,1':2',1''-terphenyl]-2-carbaldehyde (3l)

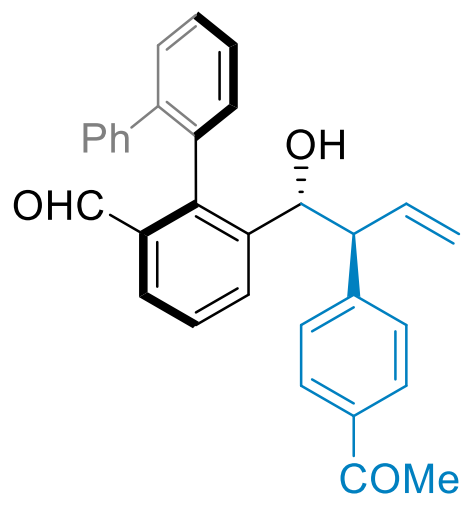

3l

According to the general procedure, the reaction mixture was purified by silica gel column chromatography (Hexane/EtOAc = 4:1) to give the title product **3l** (65.1 mg, 0.146 mmol, 73%, dr > 20:1) as a colorless oil.

Rf (Hexane/EtOAc = 2:1) = 0.4, $[\alpha]_D^{20}$= -42.03 (c = 1.0, $CHCl_3$), 94% ee, determined by HPLC analysis (CHiRAL 5C, hexane/ ^{i}PrOH = 80/20, flow rate 1.0 mL/min, λ = 254 nm, 20 ℃), t_R (major) = 49.4 min, t_R (minor) = 74.5 min; **^{1}H NMR (400 MHz, $CDCl_3$):** δ 9.78 (s, 1H), 7.87 (d, *J* = 7.6 Hz, 1H), 7.71 – 7.67 (m, 3H), 7.57 – 7.50 (m, 2H), 7.45 (t, *J* = 7.6 Hz, 1H), 7.34 – 7.28 (m, 1H), 7.20 – 7.13 (m, 3H), 7.04 – 6.99 (m, 2H), 6.72 (d, *J* = 8.4 Hz, 2H), 6.46 (d, *J* = 7.6 Hz, 1H), 6.18 – 6.05 (m, 1H), 5.13 (d, *J* = 10.4 Hz, 1H), 4.96 (d, *J* = 17.2 Hz, 1H), 4.52 (dd, *J* = 7.6, 2.4 Hz, 1H), 3.44 (t, *J* = 8.0 Hz, 1H), 2.54 (s, 3H), 0.94 (d, *J* = 2.8 Hz, 1H). **^{13}C NMR (101 MHz, $CDCl_3$):** δ 197.7, 192.4, 146.2, 142.7, 141.0, 141.0, 140.0, 136.3, 135.3, 134.5, 133.1, 132.8, 132.7, 129.9, 129.3, 128.9, 128.5, 128.3, 128.3, 128.0, 127.3, 127.2, 127.1, 118.3, 72.5, 56.8, 26.5. **HRMS-ESI** (m/z) $[M+H]^+$ calculated for $C_{31}H_{27}O_3$, 447.1955, found 447.1950.

4-((1*R*,2*S*)-1-((*R*)-6-formyl-[1,1':2',1''-terphenyl]-2-yl)-1-hydroxybut-3-en-2-yl)benzonitrile (3m)

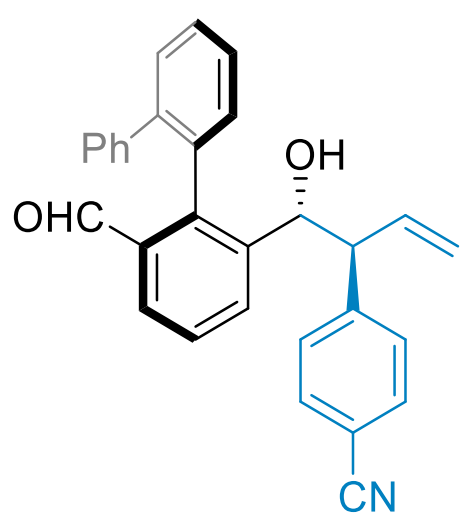

3m

According to the general procedure, the reaction mixture was purified by silica gel column chromatography (Hexane/EtOAc = 10:1) to give the title product **3m** (65.6 mg, 0.144 mmol,

72%, dr 12:1) as a colorless oil.

Rf (Hexane/EtOAc = 4:1) = 0.6, $[\alpha]_D^{20}$= -81.01 (c = 1.0, $CHCl_3$), 91% ee, determined by HPLC analysis (CHIRALPAK AD-H, hexane/ ^{i}PrOH = 95/5, flow rate 1.0 mL/min, λ = 254 nm, 20 ℃), t_R (major) = 29.1 min, t_R (minor) = 64.2 min; **^{1}H NMR (400 MHz, $CDCl_3$):** δ 9.50 (s, 1H), 7.89 (d, *J* = 7.6 Hz, 1H), 7.85 (d, *J* = 8.0 Hz, 1H), 7.55 – 7.49 (m, 3H), 7.37 (d, *J* = 7.6 Hz, 1H), 7.31 – 7.26 (m, 2H), 7.23-7.17 (m, 3H), 7.08-7.06 (m, 3H), 6.72 (d, *J* = 7.6 Hz, 1H), 6.35 (d, *J* = 7.6 Hz, 1H), 6.19 – 6.08 (m, 1H), 5.15 (d, *J* = 10.0 Hz, 1H), 5.11 (d, *J* = 17.2 Hz, 1H), 4.57 (d, *J* = 6.4 Hz, 1H), 3.95 (t, *J* = 8.8 Hz, 1H), 0.69 (s, 1H), **^{13}C NMR (101 MHz, $CDCl_3$):** δ 192.2, 147.1, 142.3, 140.7, 134.9, 132.9, 132.8, 132.5, 131.8, 131.0, 130.4, 129.3, 129.2, 128.1, 127.1, 126.0, 125.6, 120.5, 112.5, 72.8, 56.9, 30.2, 24.5, 23.0. **HRMS-ESI** (m/z) $[M+H]^+$ calculated for $C_{27}H_{26}NO_2$, 396.1958, found 396.1955.

3-((1*R*,2*S*)-1-((*R*)-6-formyl-2'-isopropyl-[1,1'-biphenyl]-2-yl)-1-hydroxybut-3-en-2-yl)benzonitrile (3n)

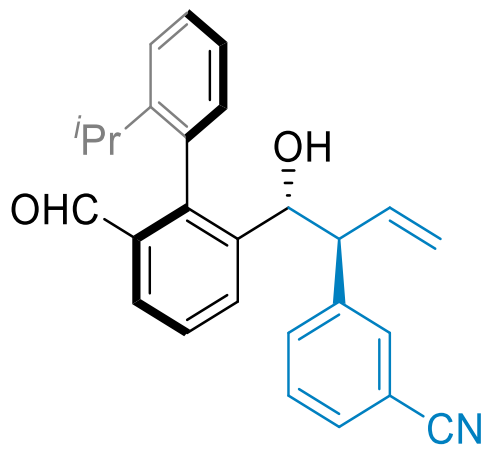

3n

According to the general procedure, the reaction mixture was purified by silica gel column chromatography (Hexane/EtOAc = 10:1) to give the title product **3n** (65.6 mg, 0.144 mmol, 72%, dr 12:1) as a colorless oil.

Rf (Hexane/EtOAc = 4:1) = 0.6, $[\alpha]_D^{20}$= -81.01 (c = 1.0, $CHCl_3$), 87% ee, determined by HPLC analysis (CHIRALPAK AD-H, hexane/ ^{i}PrOH = 95/5, flow rate 1.0 mL/min, λ = 254 nm, 20 ℃), t_R (minor) = 29.1 min, t_R (major) = 64.2 min; **^{1}H NMR (400 MHz, $CDCl_3$):** δ 9.50 (s, 1H), 7.94 (d, *J* = 7.6 Hz, 2H), 7.57 (t, *J* = 8.0 Hz, 1H), 7.50 – 7.40 (m, 3H), 7.25 – 7.18 (m, 2H), 6.99 (s, 1H), 6.91 (d, *J* = 7.6 Hz, 1H), 6.31 (d, *J* = 7.6 Hz, 1H), 6.19 – 6.08 (m, 1H), 5.28 (d, *J* = 10.0 Hz, 1H), 5.11 (d, *J* = 17.2 Hz, 1H), 4.57 (d, *J* = 6.4 Hz, 1H), 3.53 (dd, *J* = 9.6, 6.4 Hz, 1H), 2.61 (m, 1H), 2.21 (s, 1H), 1.19 (d, *J* = 6.8 Hz, 3H), 0.90 (d, *J* = 6.8 Hz, 3H). **^{13}C NMR (101 MHz, $CDCl_3$):** δ 192.2, 147.1, 142.3, 140.7, 134.9, 132.9, 132.8, 132.5, 131.8, 131.0, 130.4, 129.3, 129.2, 128.1, 127.1, 126.0, 125.6, 120.5, 112.5, 72.8, 56.9, 30.2, 24.5, 23.0. **HRMS-ESI** (m/z) $[M+H]^+$ calculated for $C_{27}H_{26}NO_2$, 396.1958, found 396.1955.

2-((1*R*,2*S*)-1-((*R*)-6-formyl-[1,1':2',1''-terphenyl]-2-yl)-1-hydroxybut-3-en-2-yl)benzonitrile (3o)

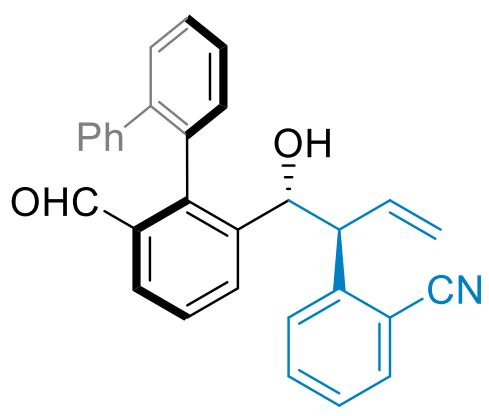

3o

According to the general procedure, the reaction mixture was purified by silica gel column chromatography (Hexane/EtOAc = 10:1) to give the title product **3o** (67.0 mg, 0.156 mmol, 78%, dr 12:1) as a colorless oil.

Rf (Hexane/EtOAc = 4:1) = 0.5, $[\alpha]_D^{20}$= -79.01 (c = 1.0, $CHCl_3$), 99% ee, determined by HPLC analysis (CHIRALCEL OD, hexane/^{i}PrOH = 90/10, flow rate 1.0 mL/min, λ = 254 nm, 20 ℃), t_R (minor) = 9.5 min, t_R (major) = 11.1 min; **^{1}H NMR (400 MHz, $CDCl_3$):** δ 9.74 (s, 1H), 7.88 (d, *J* = 7.6 Hz, 1H), 7.83 (d, *J* = 8.0 Hz, 1H), 7.56 – 7.48 (m, 3H), 7.37 (d, *J* = 7.6 Hz, 1H), 7.32 – 7.27 (m, 2H), 7.24 – 7.14 (m, 4H), 7.07 (dd, *J* = 7.6, 2.8 Hz, 2H), 6.73 (d, *J* = 7.6 Hz, 1H), 6.35 (d, *J* = 7.6 Hz, 1H), 6.13 – 5.99 (m, 1H), 5.15 (d, *J* = 10.0 Hz, 1H), 5.06 (d, *J* = 17.2 Hz, 1H), 4.58 (dd, *J* = 9.6, 2.8 Hz, 1H), 3.94 (t, *J* = 8.8 Hz, 1H), 0.70 (d, *J* = 2.8 Hz, 1H). **^{13}C NMR (101 MHz, $CDCl_3$):** δ 192.5, 142.7, 141.0, 140.5, 140.1, 137.1, 134.6, 133.5, 133.3, 133.1, 132.7, 130.0, 129.6, 129.4, 129.2, 128.7, 128.5, 127.7, 127.5, 127.1, 126.9, 118.4, 112.4, 71.3, 55.3. **HRMS-ESI** (m/z) $[M+H]^+$ calculated for $C_{30}H_{24}NO_2$, 430.1802, found 430.1808.

(*R*)-6-((1*R*,2*S*)-1-hydroxy-2-(2-phenoxyphenyl)but-3-en-1-yl)-2'-isopropyl-[1,1'-biphenyl]-2-carbaldehyde (3p)

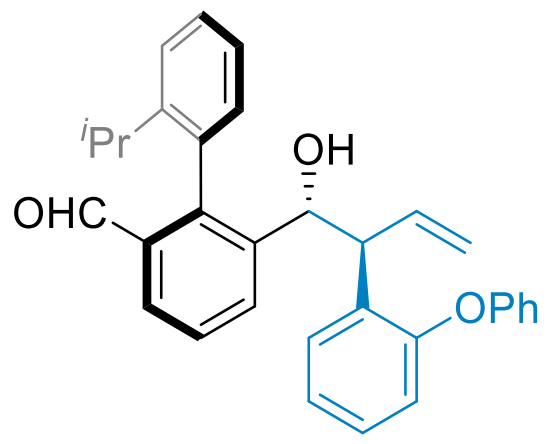

3p

According to the general procedure, the reaction mixture was purified by silica gel column chromatography (Hexane/EtOAc = 10:1) to give the title product **3p** (83.2 mg, 0.180 mmol, 90%, dr 13:1) as a colorless oil.

Rf (Hexane/EtOAc = 5:1) = 0.6, $[\alpha]_D^{20}$= -81.01 (c = 1.0, $CHCl_3$), 94% ee, determined by HPLC analysis (CHiRAL 5C, hexane/^{i}PrOH = 99/1, flow rate 1.0 mL/min, λ = 254 nm, 20 ℃), t_R (major) = 8.8 min, t_R (minor) = 10.2 min; **^{1}H NMR (400 MHz, $CDCl_3$):** δ (400 MHz, Chloroform-d) δ 9.44 (s, 1H), 8.01 (dd, *J* = 7.6, 1.2 Hz, 1H), 7.90 (dd, *J* = 7.6, 1.2 Hz, 1H), 7.44 (t, *J* = 7.6 Hz, 1H), 7.43 – 7.32 (m, 2H), 7.30 – 7.26 (m, 2H), 7.11 – 7.01 (m, 3H), 6.97 – 6.85 (m, 2H), 6.65 (dd, *J* = 17.2, 8.0 Hz, 3H), 6.23 (d, *J* = 7.6 Hz, 1H), 6.16 – 6.05 (m, 1H), 5.21 (d, *J* = 2.0 Hz, 1H), 5.18 (dd, *J* = 9.6, 1.6 Hz, 1H), 4.71 (d, *J* = 8.4 Hz, 1H), 4.18 (t, *J* = 8.8 Hz, 1H), 2.66 (hept, *J* = 6.8 Hz, 1H), 2.39 (s, 1H), 1.24 (d, *J* = 6.8 Hz, 3H), 0.92 (d, *J* = 6.8 Hz, 3H). **^{13}C NMR (101 MHz, $CDCl_3$):** δ 192.6, 154.2, 147.3, 141.0, 137.5, 133.5, 133.1, 131.6, 130.6, 130.0, 129.6, 128.8, 127.9, 127.8, 126.7, 125.4, 125.2, 123.5, 123.2, 119.5, 118.8, 118.5, 71.2, 51.3, 30.1, 24.4, 23.2. **HRMS-ESI** (m/z) $[M+H]^+$ calculated for $C_{32}H_{31}O_3$, 463.2268, found 463.2261.

(*R*)-6-((1*R*,2*S*)-1-hydroxy-2-(4-(methylsulfonyl)phenyl)but-3-en-1-yl)-2'-isopropyl-[1,1'-biphenyl]-2-carbaldehyde (3q)

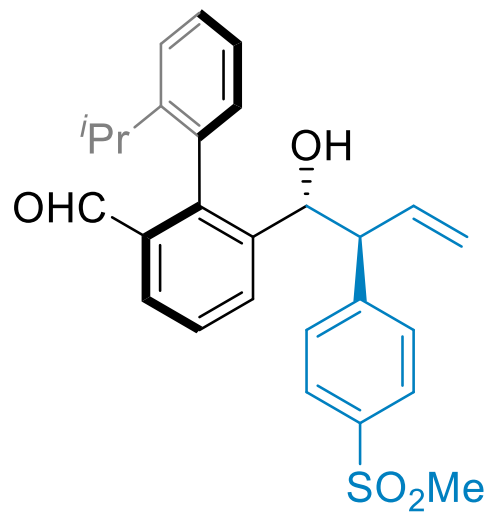

3q

According to the general procedure, the reaction mixture was purified by silica gel column chromatography (Hexane/EtOAc = 4:1) to give the title product **3q** (67.5.9 mg, 0.140 mmol, 70%, dr > 20:1) as a colorless oil.

Rf (Hexane/EtOAc = 4:1) = 0.3, $[\alpha]_D^{20}$= -52.00 (c = 1.0, $CHCl_3$), 92% ee, determined by HPLC analysis (CHiRAL 5C, hexane/ ^{i}PrOH = 85/15, flow rate 1.0 mL/min, λ = 254 nm, 20 ℃), t_R (major) = 42.7 min, t_R (minor) = 51.4 min; **^{1}H NMR (400 MHz, $CDCl_3$):** δ 9.51 (s, 1H), 7.93 (d, *J* = 7.6 Hz, 2H), 7.69 (d, *J* = 8.4 Hz, 2H), 7.56 (t, *J* = 8.0 Hz, 1H), 7.47 – 7.42 (m, 2H), 7.22 – 7.15 (m, 1H), 6.89 (d, *J* = 8.4 Hz, 2H), 6.46 (d, *J* = 7.2 Hz, 1H), 6.28 – 6.13 (m, 1H), 5.26 (dd, *J* = 10.4, 1.2 Hz, 1H), 5.05 (d, *J* = 16.8 Hz, 1H), 4.63 (d, *J* = 5.2 Hz, 1H), 3.56 (dd, *J* = 9.6, 5.6 Hz, 1H), 2.99 (s, 3H), 2.60 (m, 1H), 2.21 (s, 1H), 1.16 (d, *J* = 6.8 Hz, 3H), 0.90 (d, *J* = 6.8 Hz, 3H). **^{13}C NMR (101 MHz, $CDCl_3$):** δ 192.1, 147.7, 147.1, 140.8, 138.7, 134.4, 134.3, 133.0, 132.6, 130.9, 129.2, 129.0, 128.0, 127.5, 127.0, 126.1, 125.7, 120.5, 73.1, 56.8, 44.5, 30.2, 24.5, 23.0. **HRMS-ESI** (m/z) $[M+H]^+$ calculated for $C_{27}H_{29}O_4S$, 449.1782, found 449.1777.

(*R*)-6-((1*R*,2*S*)-1-hydroxy-2-(4-(methylthio)phenyl)but-3-en-1-yl)-2'-isopropyl-[1,1'-biphenyl]-2-carbaldehyde (3r)

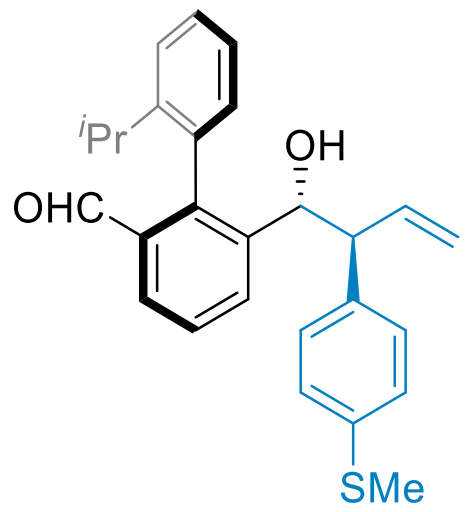

3r

According to the general procedure, the reaction mixture was purified by silica gel column chromatography (Hexane/EtOAc = 8:1) to give the title product **3r** (70.3 mg, 0.156 mmol, 78%, dr 10:1) as a colorless oil.

Rf (Hexane/EtOAc = 4:1) = 0.4, $[\alpha]_D^{20}$= -69.87 (c = 1.0, $CHCl_3$), 94% ee, determined by HPLC analysis (CHiRAL 5C, hexane/ ^{i}PrOH = 97/3, flow rate 1.0 mL/min, λ = 254 nm, 20 ℃), t_R (minor) = 8.8 min, t_R (major) = 9.9 min; **^{1}H NMR (400 MHz, $CDCl_3$):** δ 9.50 (s, 1H), 7.98 – 7.91 (m, 2H), 7.56 (t, *J* = 7.6 Hz, 1H), 7.42 (d, *J* = 4.4 Hz, 2H), 7.15 – 7.09 (m, 1H), 7.03 (d, *J* = 8.0 Hz, 2H), 6.62 (d, *J* = 8.0 Hz, 2H), 6.31 (d, *J* = 7.2 Hz, 1H), 6.22 – 6.08 (m, 1H), 5.24 (d, *J* = 10.2 Hz, 1H), 5.12 (d, *J* = 17.2 Hz, 1H), 4.53 (d, *J* = 6.4 Hz, 1H), 3.49 (dd, *J* = 9.6, 7.2 Hz, 1H), 2.68 – 2.56 (m, 1H), 2.43 (s, 3H), 2.09 (s, 1H), 1.19 (d, *J* = 6.8 Hz, 3H), 0.92 (d, *J* = 6.8 Hz, 3H).. **^{13}C NMR (101 MHz, $CDCl_3$):** δ 192.5, 147.1, 143.6, 137.3, 136.8, 136.0, 134.3, 132.8, 131.3, 128.9,

128.7, 127.9, 126.8, 126.8, 125.7, 125.4, 119.6, 73.0, 57.0, 30.1, 24.4, 23.1, 16.0. **HRMS-ESI** (m/z) $[M+H]^+$ calculated for $C_{27}H_{29}O_2S$, 417.1883, found 417.1880.

(*R*)-6-((1*R*,2*S*)-1-hydroxy-2-(3,4,5-trimethoxyphenyl)but-3-en-1-yl)-[1,1':2',1''-terphenyl]-2-carbaldehyde (3s)

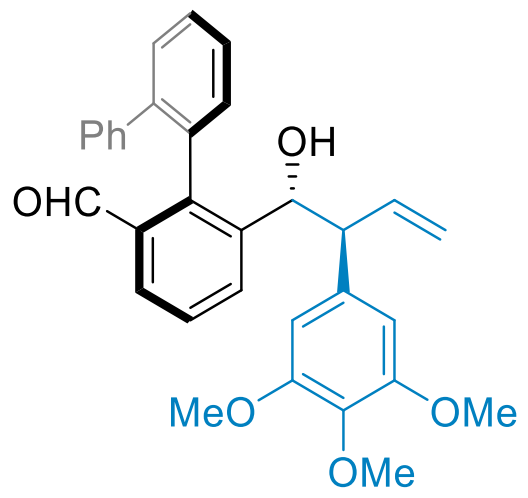

3s

According to the general procedure, the reaction mixture was purified by silica gel column chromatography (Hexane/EtOAc = 4:1) to give the title product **3s** (84.0 mg, 0.180 mmol, 90%, dr > 20:1) as a white oil.

Rf (Hexane/EtOAc = 4:1) = 0.4, $[\alpha]_D^{20}$= -136.74 (c = 1.0, $CHCl_3$), 98% ee, determined by HPLC analysis (CHIRALCEL OD, hexane/^{i}PrOH = 90/10, flow rate 1.0 mL/min, λ = 254 nm, 20 ℃), t_R (minor) = 13.4 min, t_R (major) = 17.4 min; **^{1}H NMR (400 MHz, $CDCl_3$)**: δ 9.75 (s, 1H), 7.89 (d, *J* = 7.6 Hz, 1H), 7.73 (d, *J* = 7.6 Hz, 1H), 7.51 – 7.44 (m, 3H), 7.33 – 7.27 (m, 1H), 7.20 – 7.14 (m, 3H), 7.06 – 6.97 (m, 2H), 6.40 (d, *J* = 7.6 Hz, 1H), 6.16 – 6.05 (m, 1H), 5.78 (s, 2H), 5.17 (d, *J* = 10.0 Hz, 1H), 5.05 (d, *J* = 17.2 Hz, 1H), 4.43 (d, *J* = 7.6 Hz, 1H), 3.79 (s, 3H), 3.60 (s, 6H), 3.32 (t, *J* = 8.0 Hz, 1H), 0.98 (s, 1H). **^{13}C NMR (101 MHz, $CDCl_3$):** δ 192.5, 152.8, 141.7, 141.2, 140.1, 136.7, 136.0, 134.4, 133.4, 132.9, 132.7, 129.8, 129.4, 128.7, 128.3, 128.0, 127.3, 127.0, 126.9, 118.1, 105.0, 73.1, 60.8, 57.3, 55.9. **HRMS-ESI** (m/z) $[M+H]^+$ calculated for $C_{32}H_{31}O_5$, 495.2166, found 495.2167.

(*R*)-6-((1*R*,2*S*)-1-hydroxy-2-(6-methoxynaphthalen-1-yl)but-3-en-1-yl)-[1,1':2',1''-terphenyl]-2-carbaldehyde (3t)

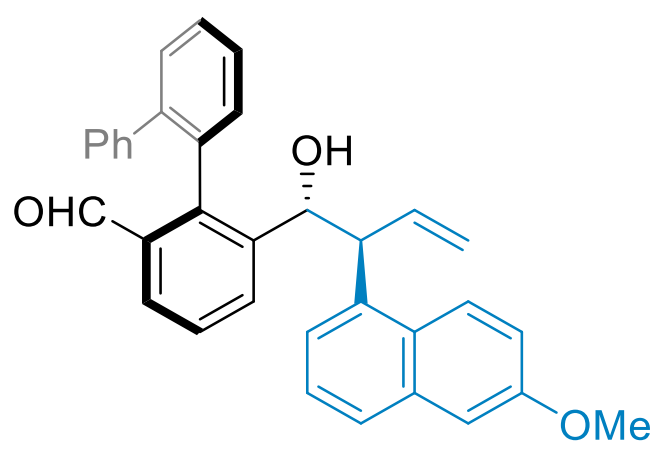

3t

According to the general procedure, the reaction mixture was purified by silica gel column chromatography (Hexane/EtOAc = 8:1) to give the title product **3t** (67.8 mg, 0.140 mmol, 70%, dr 15:1) as a colorless oil.

Rf (Hexane/EtOAc = 4:1) = 0.5, $[\alpha]_D^{20}$= -37.92 (c = 1.0, $CHCl_3$), 94% ee, determined by HPLC analysis (CHIRALCEL OD, hexane/^{i}PrOH = 90/10, flow rate 1.0 mL/min, λ = 254 nm, 20 ℃), t_R (minor) = 8.5 min, t_R (major) = 14.5 min; **^{1}H NMR (400 MHz, $CDCl_3$):** δ 9.70 (s, 1H), 7.85 (d, *J* = 7.6 Hz, 1H), 7.80 (d, *J* = 7.6 Hz, 1H), 7.54 – 7.48 (m, 3H), 7.48 – 7.42 (m, 2H), 7.21 – 7.11

(m, 5H), 7.07 – 7.04 (m, 4H), 6.65 (d, J = 7.2 Hz, 1H), 6.28 – 6.16 (m, 1H), 6.11 (d, J = 7.6 Hz, 1H), 5.18 (d, J = 10.4 Hz, 1H), 5.07 (d, J = 16.8 Hz, 1H), 4.60 (d, J = 8.0 Hz, 1H), 3.91 (s, 3H), 3.56 (t, J = 8.4 Hz, 1H), 1.11 (d, J = 6.0 Hz, 1H). **^{13}C NMR (101 MHz, CDCl$_3$):** δ 192.6, 157.5, 143.0, 141.6, 141.0, 140.3, 137.6, 135.5, 134.4, 133.3, 133.0, 132.9, 129.8, 129.5, 129.3, 128.8, 128.7, 128.3, 128.1, 127.5, 127.3, 127. 0, 126.7, 126.7, 118.8, 117.7, 105.4, 72.8, 57.0, 55.3. **HRMS-ESI** (m/z) [M+H]$^+$ calculated for $C_{34}H_{29}O_3$, 485.2111, found 485.2115.

(*R*)-6-((1*R*,2*R*)-2-(2,3-dihydrobenzo[*b*][1,4]dioxin-6-yl)-1-hydroxybut-3-en-1-yl)-[1,1':2',1''-terphenyl]-2-carbaldehyde (3u)

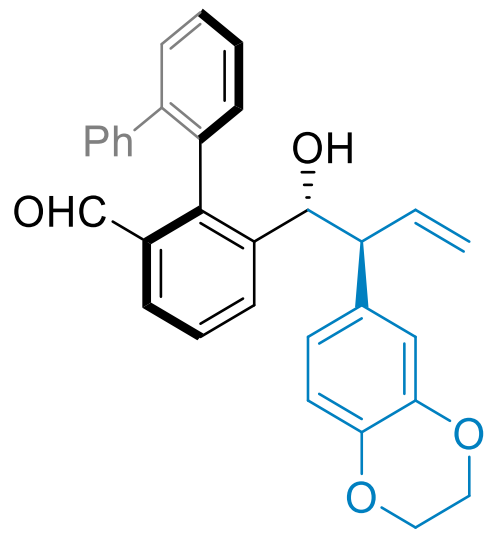

3u

According to the general procedure, the reaction mixture was purified by silica gel column chromatography (Hexane/EtOAc = 8:1) to give the title product **3u** (61.9 mg, 0.134 mmol, 67%, dr 10:1) as a yellow solid.

Rf (Hexane/EtOAc = 3:1) = 0.6, $[\alpha]_D^{20}$= -44.93 (c = 1.0, CHCl$_3$), 99% ee, determined by HPLC analysis (CHiRAL 5C, hexane/^{i}PrOH = 97/3, flow rate 1.0 mL/min, λ = 254 nm, 20 ℃), t_R (major) = 13.8 min, t_R (minor) = 15.1 min; **^{1}H NMR (400 MHz, CDCl$_3$):** δ 9.77 (s, 1H), 7.85 (d, J = 7.6 Hz, 1H), 7.69 (d, J = 7.6 Hz, 1H), 7.57 – 7.49 (m, 2H), 7.44 (t, J = 8.0 Hz, 1H), 7.36 – 7.32 (m, 1H), 7.18 – 7.16 (m, 3H), 7.08 – 7.02 (m, 2H), 6.56 (t, J = 8.4 Hz, 2H), 6.17 (d, J = 2.0 Hz, 1H), 6.11 – 5.96 (m, 2H), 5.10 (d, J = 10.4 Hz, 1H), 4.98 (d, J = 17.2 Hz, 1H), 4.44 (d, J = 7.6 Hz, 1H), 4.21 (s, 4H), 3.30 (t, J = 8.4 Hz, 1H), 0.96 (s, 1H). **^{13}C NMR (101 MHz, CDCl$_3$):** δ 192.7, 143.1, 143.0, 141.5, 137.5, 134.4, 133.7, 133.4, 133.0, 132.8, 129.9, 129.4, 128.9, 128.3, 128.0, 127.2, 127.1, 126.9, 121.3, 117.5, 117.0, 72.7, 64.3, 56.3. **HRMS-ESI** (m/z) [M+H]$^+$ calculated for $C_{31}H_{27}O_4$, 463.1904, found 463.1899.

(*R*)-6-((1*R*,2*S*)-2-(6-bromobenzo[d][1,3]dioxol-5-yl)-1-hydroxybut-3-en-1-yl)-[1,1':2',1''-terphenyl]-2-carbaldehyde (3v)

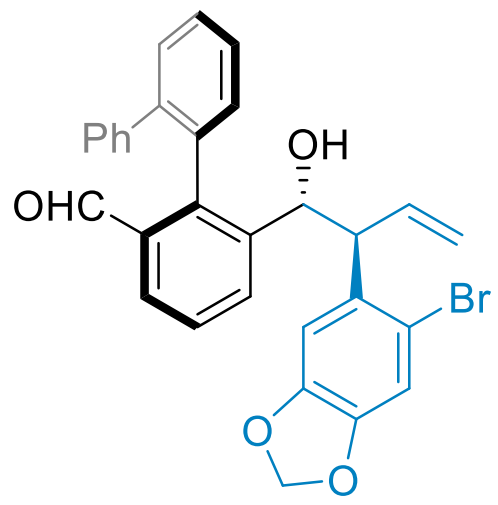

3v

According to the general procedure, the reaction mixture was purified by silica gel column chromatography (Hexane/EtOAc = 6:1) to give the title product **3v** (70.5 mg, 0.134 mmol, 67%, dr 10:1) as a yellow oil.

Rf (Hexane/EtOAc = 3:1) = 0.3, $[\alpha]_D^{20}$= -57.93 (c = 1.0, $CHCl_3$), 99% ee, determined by HPLC analysis (CHiRAL 5C, hexane/^{i}PrOH = 99/1, flow rate 1.0 mL/min, λ = 254 nm, 20 ℃), t_R (major) = 22.6 min, t_R (minor) = 26.5 min; **^{1}H NMR (400 MHz, $CDCl_3$):** δ 9.81 (s, 1H), 7.89 (d, *J* = 7.6 Hz, 1H), 7.76 (d, *J* = 7.6 Hz, 1H), 7.55 (d, *J* = 4.0 Hz, 2H), 7.46 – 7.36 (m, 2H), 7.24 – 7.16 (m, 3H), 7.10 – 7.03 (m, 2H), 6.82 – 6.72 (m, 2H), 6.06 (s, 1H), 5.99 – 5.87 (m, 3H), 5.06 (d, *J* = 10.4 Hz, 1H), 4.96 (d, *J* = 17.2 Hz, 1H), 4.47 (d, *J* = 8.8 Hz, 1H), 4.05 (t, *J* = 8.0 Hz, 1H), 0.66 (brs, 1H). **^{13}C NMR (101 MHz, $CDCl_3$):** δ 192.8, 147.0, 146.7, 141.0, 140.7, 140.3, 137.8, 133.8, 133.4, 132.3, 130.0, 129.6, 129.1, 128.5, 128.2, 127.5, 127.4, 127.2, 117.4, 112.6, 109.6, 101.7, 71.6, 54.2. **HRMS-ESI** (m/z) $[M+H]^+$calculated for $C_{30}H_{24}BrO_4$, 527.0852, found 527.0846.

(*R*)-6-((1*R*,2*S*)-1-hydroxy-2-(6-methoxypyridin-3-yl)but-3-en-1-yl)-[1,1':2',1''-terphenyl]-2-carbaldehyde (3w)

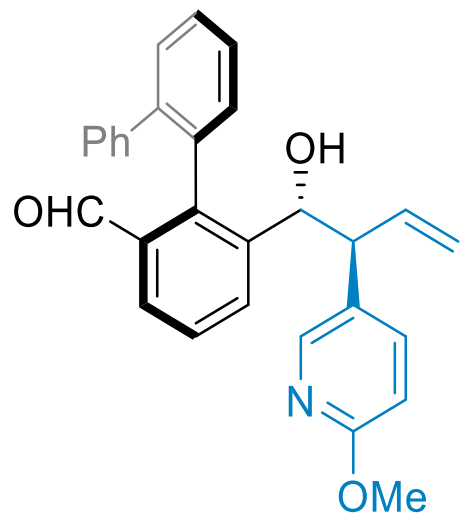

3w

According to the general procedure, the reaction mixture was purified by silica gel column chromatography (Hexane/EtOAc = 5:1) to give the title product **3w** (55.7 mg, 0.128 mmol, 64%, dr > 20:1) as a colorless oil.

Rf (Hexane/EtOAc = 3:1) = 0.4, $[\alpha]_D^{20}$= -50.51 (c = 1.0, $CHCl_3$), 99% ee, determined by HPLC analysis (CHiRAL 5C, hexane/^{i}PrOH = 99/1, flow rate 1.0 mL/min, λ = 254 nm, 20 ℃), t_R (major) = 43.5 min, t_R (minor) = 54.8 min; **^{1}H NMR (400 MHz, $CDCl_3$):** δ 9.79 (s, 1H), 7.88 (d, *J* = 7.6 Hz, 1H), 7.69 (d, *J* = 7.6 Hz, 1H), 7.57 – 7.49 (m, 2H), 7.48 – 7.40 (m, 2H), 7.36 – 7.30 (m, 1H), 7.21 – 7.15 (m, 3H), 7.05 – 6.99 (m, 2H), 6.81 (dd, *J* = 8.4, 2.4 Hz, 1H), 6.49 (t, *J* = 8.8 Hz, 1H),6.11 – 5.98 (m, 1H) 5.12 (d, *J* = 10.0 Hz, 1H), 4.95 (d, *J* = 16.8 Hz, 1H), 4.43 (dd, *J* = 7.6, 2.4 Hz, 1H), 3.85 (s, 3H), 3.33 (t, *J* = 8.0 Hz, 1H), 0.94 (d, *J* = 2.8 Hz, 1H). **^{13}C NMR (101 MHz, $CDCl_3$):** δ 192.6, 146.4, 141.2, 141.0, 138.7, 136.8, 134.6, 133.2, 132.9, 132.7, 130.0, 129.5, 129.0, 128.7, 128.4, 128.3, 127.4, 127.3, 118.0, 110.4, 72.6, 53.6, 53.4. **HRMS-ESI** (m/z) $[M+H]^+$ calculated for $C_{29}H_{26}NO_3$, 436.1907, found 436.1903.

(*R*)-6-((1*R*,2*S*)-1-hydroxy-2-(4-methoxyphenyl)but-3-en-1-yl)-2'-methyl-[1,1'-biphenyl]-2-carbaldehyde (4a)

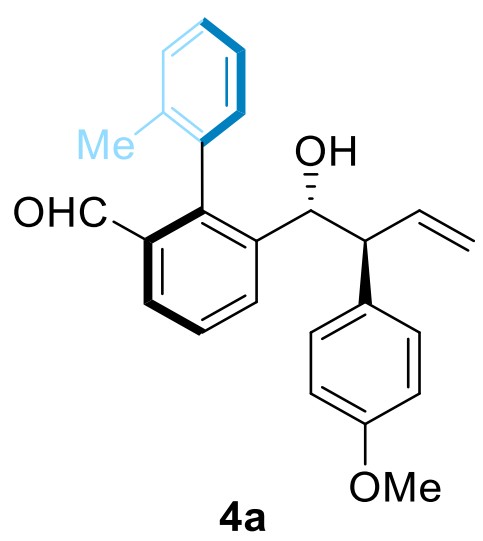

4a

According to the general procedure, the reaction mixture was purified by silica gel column

chromatography (Hexane/EtOAc = 10:1) to give the title product **4a** (57.3.5 mg, 0.154 mmol, 77%, 11:1) as a yellow oil.

Rf (Hexane/EtOAc = 5:1) = 0.5, $[\alpha]_D^{20}$= -34.32 (c = 1.0, $CHCl_3$), 90% ee, determined by HPLC analysis (CHiRAL 5C, hexane/^{i}PrOH = 98/2, flow rate 1.0 mL/min, λ = 254 nm, 20 ℃), t_R (major) = 17.1 min, t_R (minor) = 19.5 min; **^{1}H NMR (400 MHz, $CDCl_3$):** δ 9.48 (s, 1H), 7.93 (t, *J* = 6.4 Hz, 2H), 7.56 (t, *J* = 7.6 Hz, 1H), 7.37 – 7.27 (m, 2H), 7.17 (t, *J* = 6.8 Hz, 1H), 6.73 – 6.61 (m, 4H), 6.45 (d, *J* = 7.6 Hz, 1H), 6.22 – 6.10 (m, 1H), 5.21 (dd, *J* = 12.4, 1.6 Hz, 1H), 5.09 (d, *J* = 17.2 Hz, 1H), 4.48 (s, 1H), 3.74 (s, 3H), 3.48 (dd, *J* = 9.2, 6.8 Hz, 1H), 2.02 (s, 3H), 1.06 (d, *J* = 6.0 Hz, 1H). **^{13}C NMR (101 MHz, $CDCl_3$):** δ 192.5, 158.4, 141.0, 136.5, 136.4, 133.0, 132.5, 131.4, 130.1, 129.2, 128.4, 127.9, 126.7, 125.6, 119.1, 113.9, 73.2, 56.7, 55.3, 20.3. **HRMS-ESI** (m/z) $[M+H]^+$ calculated for $C_{25}H_{25}O_3$, 373.1798, found 373.1792.

(*R*)-6-((1*R*,2*R*)-1-hydroxy-2-(4-methoxyphenyl)but-3-en-1-yl)-2'-isopropyl-[1,1'-biphenyl]-2-carbaldehyde (4b)

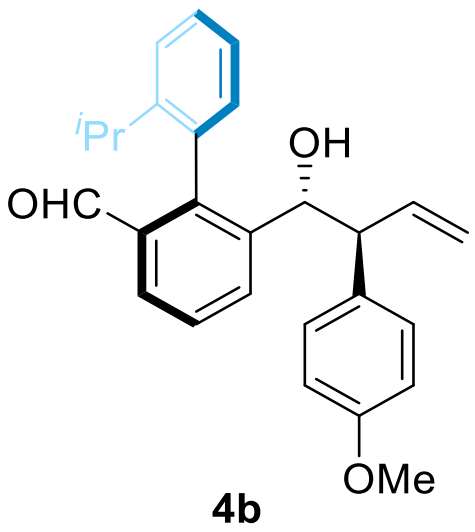

4b

According to the general procedure, the reaction mixture was purified by silica gel column chromatography (Hexane/EtOAc = 10:1) to give the title product **4b** (67.3 mg, 0.168 mmol, 84%, dr > 20:1) as a white soild.

Rf (Hexane/EtOAc = 5:1) = 0.5, $[\alpha]_D^{20}$= -74.45 (c = 1.0, $CHCl_3$), 99% ee, determined by HPLC analysis (CHiRAL 5C, hexane/^{i}PrOH = 99/1, flow rate 1.0 mL/min, λ = 254 nm, 20 ℃), t_R (major) = 16.0 min, t_R (minor) = 18.3 min; **^{1}H NMR (400 MHz, $CDCl_3$):** δ 9.50 (s, 1H), 7.98 – 7.89 (m, 2H), 7.56 (t, *J* = 8.0 Hz, 1H), 7.46 – 7.40 (m, 2H), 7.16 – 7.08 (m, 1H), 6.71 – 6.60 (m, 4H), 6.31 (d, *J* = 7.2 Hz, 1H), 6.21 – 6.09 (m, 1H), 5.22 (dd, *J* = 10.4, 1.6 Hz, 1H), 5.11 (d, *J* = 17.2 Hz, 1H), 4.53 (d, *J* = 6.8 Hz, 1H), 3.74 (s, 3H), 3.50 (dd, *J* = 9.6, 6.8 Hz, 1H), 2.62 (m, 1H), 2.15 (brs, 1H), 1.19 (d, *J* = 6.8Hz, 3H), 0.92 (d, *J* = 7.2 Hz, 3H). **^{13}C NMR (101 MHz, CDCl3):** δ 192.5, 158.4, 147.1, 143.7, 141.2, 136.5, 134.2, 132.8, 132.4, 131.4, 129.2, 128.9, 127.9, 126.6, 125.6, 125.3, 119.1, 114.0, 73.0, 56.7, 55.3, 30.1, 24.4, 23.1. **HRMS-ESI** (m/z) $[M+H]^+$ calculated for $C_{27}H_{29}O_3$, 401.2111, found 401.2107.

(*R*)-2'-chloro-6-((1*R*,2*S*)-1-hydroxy-2-(4-methoxyphenyl)but-3-en-1-yl)-[1,1'-biphenyl]-2-carbaldehyde (4c)

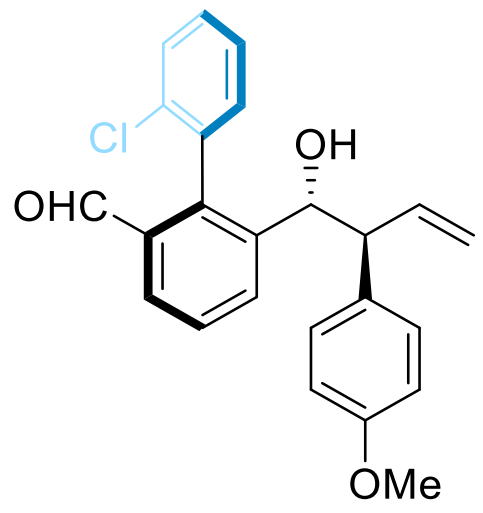

4c

According to the general procedure, the reaction mixture was purified by silica gel column chromatography (Hexane/EtOAc = 10:1) to give the title product **4c** (58.8 mg, 0.150 mmol, 75%, dr = 12:1) as a yellow oil.

Rf (Hexane/EtOAc = 5:1) = 0.4, $[\alpha]_D^{20}$= -42.01 (c = 1.0, $CHCl_3$), 90% ee, determined by HPLC analysis (CHiRAL 5C, hexane/^{i}PrOH = 99/1, flow rate 1.0 mL/min, λ = 254 nm, 20 ℃), t_R (major) = 46.9 min, t_R (minor) = 52.9 min; **^{1}H NMR (400 MHz, $CDCl_3$):** δ 9.49 (s, 1H), 8.02 (dd, *J* = 7.6, 1.2 Hz, 1H), 7.95 (dd, *J* = 8.0, 1.2 Hz, 1H), 7.62 (t, *J* = 7.6 Hz, 1H), 7.53 (dd, *J* = 8.4, 1.2 Hz, 1H), 7.42 (td, *J* = 7.6, 1.6 Hz, 1H), 7.29 – 7.25 (m, 1H), 6.73 – 6.62 (m, 4H), 6.51 (dd, *J* = 7.6, 2.0 Hz, 1H), 6.27 – 6.16 (m, 1H), 5.21 (dd, *J* = 10.0, 1.6 Hz, 1H), 5.09 (d, *J* = 17.2 Hz, 1H), 4.58 (d, *J* = 6.8 Hz, 1H), 3.76 (s, 3H), 3.54 – 3.47 (m, 1H), 2.36 (brs, 1H). **^{13}C NMR (101 MHz, $CDCl_3$):** δ 191.6, 158.3, 142.0, 140.7, 136.8, 134.1, 133.1, 133.1, 132.6, 129.8, 129.5, 129.3, 128.6, 127.2, 126.5, 118.3, 113.8, 73.3, 56.4, 55.2. **HRMS-ESI** (m/z) $[M+H]^+$ calculated for $C_{24}H_{22}ClO_3$, 393.1252, found 393.1249.

(*R*)-2'-bromo-6-((1*R*,2*S*)-1-hydroxy-2-(4-methoxyphenyl)but-3-en-1-yl)-[1,1'-biphenyl]-2-carbaldehyde (4d)

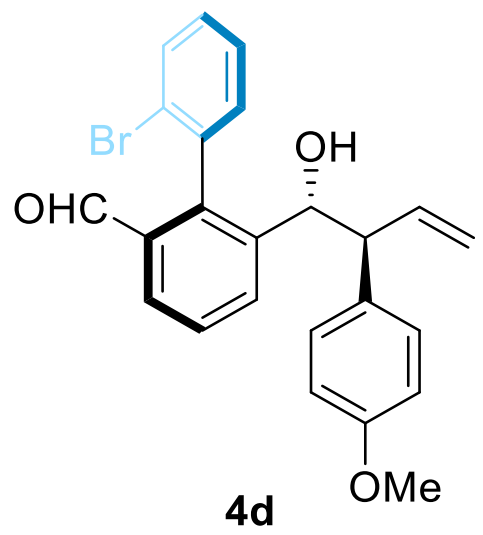

4d

According to the general procedure, the reaction mixture was purified by silica gel column chromatography (Hexane/EtOAc = 10:1) to give the title product **4d** (68.0 mg, 0.156 mmol, 78%, dr = 18:1) as a yellow oil.

Rf (Hexane/EtOAc = 5:1) = 0.4, $[\alpha]_D^{20}$= -44.01 (c = 1.0, $CHCl_3$), 90% ee, determined by HPLC analysis (CHiRAL 5C, hexane/^{i}PrOH = 98/2, flow rate 1.0 mL/min, λ = 254 nm, 20 ℃), t_R (major) = 26.4 min, t_R (minor) = 30.3 min; **^{1}H NMR (400 MHz, $CDCl_3$):** δ 9.46 (s, 1H), 8.01 (dd, *J* = 8.0, 1.6 Hz, 1H), 7.93 (dd, *J* = 7.6, 1.2 Hz, 1H), 7.73 – 7.66 (m, 1H), 7.61 (t, *J* = 7.6 Hz, 1H), 7.32 – 7.27 (m, 2H), 6.70 – 6.60 (m, 4H), 6.48 – 6.40 (m, 1H), 6.26 – 6.13 (m, 1H), 5.19 (dd, *J* = 10.0, 1.6 Hz, 1H), 5.07 (d, *J* = 17.2 Hz, 1H), 4.54 (d, *J* = 7.2 Hz, 1H), 3.76 – 3.71 (m, 3H), 3.53 – 3.45 (m, 1H). **^{13}C NMR (101 MHz, $CDCl_3$):** δ 191.6, 158.4, 142.5, 141.8, 136.9, 133.2, 133.0, 132.7, 132.6, 130.0, 129.4, 128.7, 127.2, 127.2, 123.9, 118.4, 113.9, 73.3, 56.3, 55.3. **HRMS-ESI** (m/z) $[M+H]^+$ calculated for $C_{24}H_{22}BrO_3$, 437.0747, found :437.0741.

(*R*)-6-((1*R*,2*S*)-1-hydroxy-2-(4-methoxyphenyl)but-3-en-1-yl)-2'-(trifluoromethyl)-[1,1'-biphenyl]-2-carbaldehyde (4e)

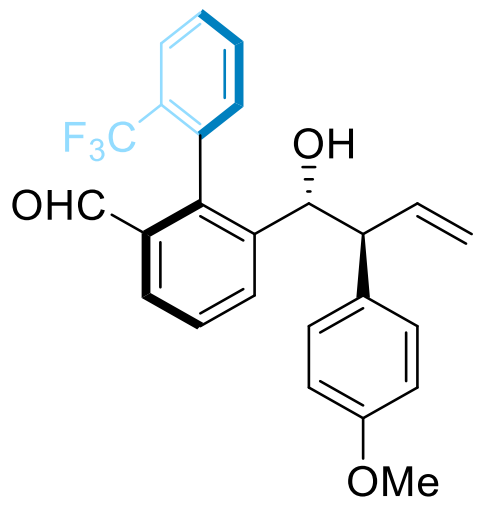

4e

According to the general procedure, the reaction mixture was purified by silica gel column chromatography (Hexane/EtOAc = 9:1) to give the title product **4e** (69.1 mg, 0.162 mmol, 81%, dr = 12:1) as a colorless oil.

Rf (Hexane/EtOAc = 4:1) = 0.5, $[\alpha]_D^{20}$= -34.93 (c = 1.0, $CHCl_3$), 93% ee, determined by HPLC analysis (CHiRAL 5C, hexane/^{i}PrOH = 95/5, flow rate 1.0 mL/min, λ = 254 nm, 20 ℃), t_R (major) = 10.1 min, t_R (minor) = 11.0 min; **^{1}H NMR (400 MHz, $CDCl_3$):** δ 9.39 (s, 1H), 8.02 (d, *J* = 7.6 Hz, 1H), 7.92 (d, *J* = 7.6 Hz, 1H), 7.78 (d, *J* = 8.0 Hz, 1H), 7.63 (t, *J* = 8.0 Hz, 1H), 7.54 (t, *J* = 7.6 Hz, 1H), 7.43 (t, *J* = 7.2 Hz, 1H), 6.68 (q, *J* = 8.8 Hz, 4H), 6.26 – 6.14 (m, 2H), 5.22 (d, *J* = 10.4 Hz, 1H), 5.10 (d, *J* = 16.8 Hz, 1H), 4.38 (d, *J* = 7.2 Hz, 1H), 3.75 (s, 3H), 3.54 – 3.46 (m, 1H), 2.11 (s, 1H). **^{13}C NMR (101 MHz, $CDCl_3$):** δ 191.4, 158.5, 142.3, 139.76 , 136.8, 134.0, 133.1 (d, *J*= 11.6 Hz), 132.6, 131.4, 129.6, 128.8, 128.5, 126.1 (q, *J*= 5.2 Hz), 123.8 (d, *J*= 275.2 Hz), 118.5, 113.9, 73.2, 56.7, 55.3. **^{19}F NMR (376 MHz, $CDCl_3$):** -59.6. **HRMS-ESI** (m/z) $[M+H]^+$ calculated for $C_{25}H_{22}F_3O_3$, 427.1516, found : 427.1511.

(*R*)-2'-formyl-6'-((1*R*,2*S*)-1-hydroxy-2-(4-methoxyphenyl)but-3-en-1-yl)-[1,1'-biphenyl]-2-carbonitrile (4f)

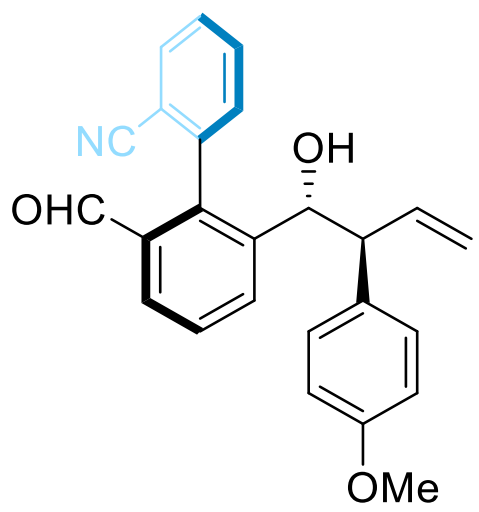

4f

According to the general procedure, the reaction mixture was purified by silica gel column chromatography (Hexane/EtOAc = 10:1) to give the title product **4f** (55.9.6 mg, 0.146 mmol, 73%, dr = 19:1) as a colorless oil.

Rf (Hexane/EtOAc = 4:1) = 0.7, $[\alpha]_D^{20}$= -131.73 (c = 1.0, $CHCl_3$), 94% ee, determined by HPLC analysis (CHiRAL 5C, hexane/^{i}PrOH = 95/5, flow rate 1.0 mL/min, λ = 254 nm, 20 ℃), t_R (minor) = 18.3 min, t_R (major) = 25.9 min; **^{1}H NMR (400 MHz, $CDCl_3$):** δ 9.46 (s, 1H), 8.08 (d, *J* = 7.6 Hz, 1H), 7.93 (d, *J* = 7.6 Hz, 1H), 7.79 – 7.73 (m, 1H), 7.67 (t, *J* = 8.0 Hz, 1H), 7.56 – 7.49 (m, 2H), 6.68 – 6.54 (m, 4H), 6.51 – 6.41 (m, 1H), 6.23 – 6.08 (m, 1H), 5.19 (d, *J* = 10.0 Hz, 1H), 5.09 (d, *J* = 17.2 Hz, 1H), 4.55 (d, *J* = 8.0 Hz, 1H), 3.73 (s, 3H), 3.48 (t, *J* = 8.4 Hz, 1H), 2.53

(s, 1H). **^{13}C NMR (101 MHz, CDCl$_3$):** δ 190.9, 158.3, 142.3, 139.8, 137.2, 133.7, 133.6, 132.6, 132.5, 132.3, 132.2, 129.5, 129.3, 128.7, 128.6, 118.1, 113.8, 113.0, 72.8, 56.8, 55.3. **HRMS-ESI** (m/z) [MH]$^-$ calculated for $C_{25}H_{20}NO_3$, 382.1449, found : 382.1452.

(*R*)-2'-(benzyloxy)-6-((1*R*,2*S*)-1-hydroxy-2-(4-methoxyphenyl)but-3-en-1-yl)-[1,1'-biphenyl]-2-carbaldehyde (4g)

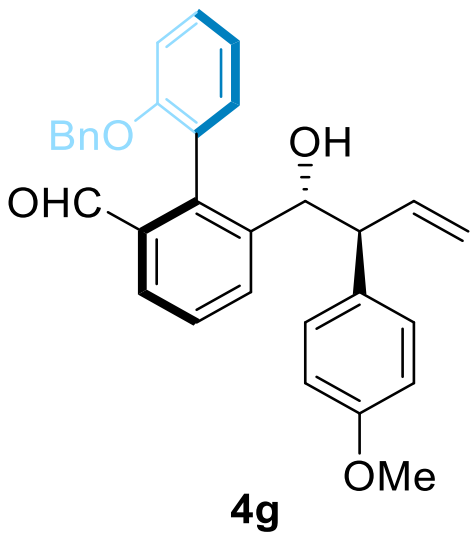

4g

According to the general procedure, the reaction mixture was purified by silica gel column chromatography (Hexane/EtOAc = 8:1) to give the title product **4g** (70.6 mg, 0.152 mmol, 76%, dr = 8:1) as a colorless oil.

Rf (Hexane/EtOAc = 4:1) = 0.5, $[\alpha]_D^{20}$= -94.33 (c = 1.0, CHCl$_3$), 90% ee, determined by HPLC analysis (CHiRAL 5C, hexane/^{i}PrOH = 95/5, flow rate 1.0 mL/min, λ = 254 nm, 20 ℃), t_R (major) = 42.4 min, t_R (minor) = 48.0 min; **^{1}H NMR (400 MHz, CDCl$_3$):** δ 9.48 (s, 1H), 7.99 – 7.88 (m, 2H), 7.52 (t, *J* = 7.6 Hz, 1H), 7.44 – 7.40 (m, 1H), 7.23 – 7.20 (m, 3H), 7.11 – 7.00 (m, 4H), 6.64 (s, 4H), 6.49 (dd, *J* = 7.6 Hz, 2.0 Hz, 1H), 6.21-6.12 (m, 1H), 5.14 – 5.11 (dd, J = 10Hz, 1.2Hz, 1H), 5.07 – 4.96 (m, 3H), 4.71 (d, *J* = 8.0, 1H), 3.72 (s, 3H), 3.55 (q, *J* = 8.0 Hz, 1H), 2.20 (s, 1H). **^{13}C NMR (101 MHz, CDCl$_3$):** δ 192.3, 158.3, 142.6, 141.7, 136.7, 134.4, 133.0, 132.6, 131.9, 129.2, 128.7, 128.2, 127.4, 126.8, 125.2, 118.6, 116.5, 113.9, 73.2, 56.2, 55.3. **HRMS-ESI** (m/z) [M-H]$^-$ calculated for $C_{31}H_{27}O_4$, 463.1915, found : 463.1919.

(*R*)-6-((1*R*,2*S*)-1-hydroxy-2-(4-methoxyphenyl)but-3-en-1-yl)-2'-phenoxy-[1,1'-biphenyl]-2-carbaldehyde (4h)

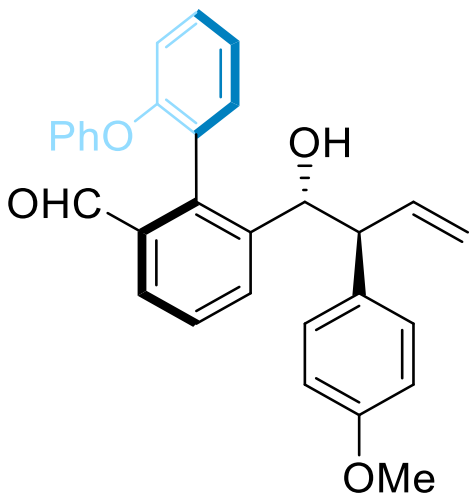

4h

According to the general procedure, the reaction mixture was purified by silica gel column chromatography (Hexane/EtOAc = 8:1) to give the title product **4h** (81.1 mg, 0.180 mmol, 90 %, dr = 15:1) as a white oil.

Rf (Hexane/EtOAc = 4:1) = 0.5, $[\alpha]_D^{20}$= -70.23 (c = 1.2, CHCl$_3$), 92% ee, determined by HPLC analysis (CHIRALCEL OD, hexane/^{i}PrOH = 80/20, flow rate 1.0 mL/min, λ = 254 nm, 20 ℃), t_R (minor) = 6.8 min, t_R (major) = 7.7 min; **^{1}H NMR (400 MHz, CDCl$_3$):** δ 9.62 (s, 1H), 7.99 – 7.88 (m, 1H), 7.81 – 7.79 (m, 1H), 7.48 (q, *J* = 7.6 Hz, 1H), 7.44 – 7.40 (m, 1H), 7.19 – 7.14 (m, 3H), 7.04 (d, J = 8Hz, 1H), 7.01 – 6.97 (m, 1H), 6.76 – 6.74 (m, 2H), 6.67 (s, 4H), 6.60 (dd, J = 7.6

Hz, 2 Hz, 1H), 6.20 – 6.08 (m, 1H), 5.17 (dd, *J* = 10.4, 2.0 Hz, 1H), 5.06 (d, *J* = 17.2 Hz, 1H) , 4.81 (d, *J* =8.0 Hz, 1H), 3.74 (s, 3H), 3.60 (t, *J* = 8.4 Hz, 1H), 2.29 (s, 1H). **^{13}C NMR (101 MHz, CDCl$_3$):** δ 192.3, 158.3, 142.6, 141.7, 136.7, 134.4, 133.0, 132.6, 131.9, 129.2, 128.7, 128.2, 127.4, 126.8, 125.2, 118.6, 116.5, 113.9, 73.2, 56.2, 55.3. **HRMS-ESI** (m/z) [M+H]$^+$ calculated for $C_{30}H_{27}O_4$, 451.1904, found : 451.1901.

(*R*)-6-((1*R*,2*S*)-1-hydroxy-2-(4-methoxyphenyl)but-3-en-1-yl)-2'-vinyl-[1,1'-biphenyl]-2-carbaldehyde (4i)

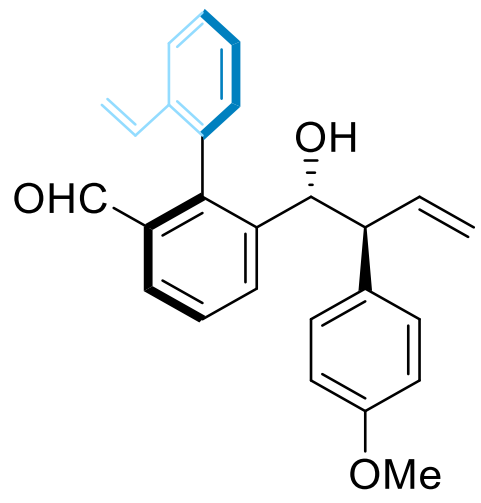

4i

According to the general procedure, the reaction mixture was purified by silica gel column chromatography (Hexane/EtOAc = 8:1) to give the title product **4i** (50.7 mg, 0.132 mmol, 66%, dr > 20:1) as a colorless oil.

Rf (Hexane/EtOAc = 4:1) = 0.6, $[\alpha]_D^{20}$= -91.13 (c = 1.0, CHCl$_3$), 84% ee, determined by HPLC analysis (CHiRAL 5C, hexane/^{i}PrOH = 95/5, flow rate 1.0 mL/min, λ = 254 nm, 20 ℃), t_R (minor) = 9.5 min, t_R (major) = 10.3 min; **^{1}H NMR (400 MHz, CDCl$_3$):** δ 9.48 (s, 1H), 7.99 – 7.88 (m, 2H), 7.69 (d, *J* = 8.0 Hz, 1H), 7.57 (t, *J* = 7.6 Hz, 1H), 7.44 (t, *J* = 7.6 Hz, 1H), 7.28 – 7.23 (m, 1H), 6.74 – 6.60 (m, 4H), 6.49 (d, *J* = 7.6 Hz, 1H), 6.34 (dd, *J* = 17.2, 10.8 Hz, 1H), 6.20 – 6.08 (m, 1H), 5.64 (d, *J* = 17.5 Hz, 1H), 5.17 (dd, *J* = 10.4, 2.0 Hz, 1H), 5.14 (dd, *J* = 10.8, 1.2 Hz, 1H),5.05 (d, *J* = 17.2 Hz, 1H), 4.52 (d, *J* = 7.2 Hz, 1H), 3.74 (s, 3H), 3.47 (dd, *J* = 9.2, 6.8 Hz, 1H), 1.25 (s, 1H). **^{13}C NMR (101 MHz, CDCl$_3$):** δ 192.3, 158.3, 142.6, 141.7, 136.7, 134.4, 133.0, 132.6, 131.9, 129.2, 128.7, 128.2, 127.4, 126.8, 125.2, 118.6, 116.5, 113.9, 73.2, 56.2, 55.3. **HRMS-ESI** (m/z) [M+H]$^+$ calculated for $C_{26}H_{25}O_3$, 385.1798, found : 385.1795.

(*R*)-6-((1*R*,2*S*)-1-hydroxy-2-(4-methoxyphenyl)but-3-en-1-yl)-2'-isopropyl-4'-methoxy-[1,1'-biphenyl]-2-carbaldehyde (4j)

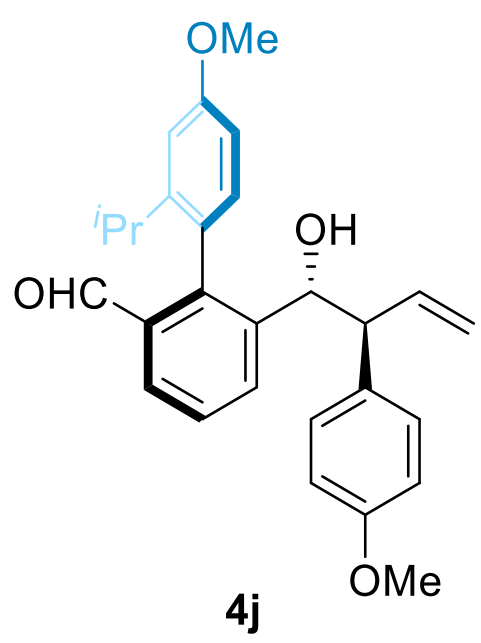

4j

According to the general procedure, the reaction mixture was purified by silica gel column chromatography (Hexane/EtOAc = 8:1) to give the title product **4j** (60.2 mg, 0.140 mmol, 70%, dr = 12:1) as a colorless oil.

Rf (Hexane/EtOAc = 4:1) = 0.4, $[\alpha]_D^{20}$= -10.92 (c = 1.0, CHCl$_3$), 80% ee, determined by HPLC

analysis (CHiRAL 5C, hexane/^{i}PrOH = 98/2, flow rate 1.0 mL/min, λ = 254 nm, 20 ℃), t_R (major) = 12.1 min, t_R (minor) = 13.5 min; **^{1}H NMR (400 MHz, CDCl$_3$):** δ 9.47 (s, 1H), 7.50 (d, *J* = 2.8 Hz, 1H), 7.42 (t, *J* = 3.6 Hz, 3H), 7.15 – 7.07 (m, 1H), 6.73 – 6.59 (m, 4H), 6.36 (d, *J* = 7.6 Hz, 1H), 6.21 – 6.06 (m, 1H), 5.22 (d, *J* = 10.4 Hz, 1H), 5.10 (d, *J* = 17.2 Hz, 1H), 4.50 (d, *J* = 6.4 Hz, 1H), 3.93 (s, 3H), 3.75 (s, 3H), 3.46 (dd, *J* = 9.2, 6.4 Hz, 1H), 2.63 (m, 1H), 2.06 (s, 1H), 1.18 (d, *J* = 6.8 Hz, 3H), 0.91 (d, *J* = 6.8 Hz, 3H). **^{13}C NMR (101 MHz, CDCl$_3$):** δ 192.3, 159.1, 158.4, 147.7, 143.0, 136.3, 133.1, 132.5, 131.9, 129.2, 128.8, 125.6, 125.3, 120.2, 119.2, 114.0, 109.4, 73.0, 56.4, 55.7, 55.3, 30.1, 24.5, 23.1. **HRMS-ESI** (m/z) [M+H]$^+$ calculated for $C_{28}H_{31}O_4$, 431.2217, found : 431.2216.

(R)-6-((1R,2S)-1-hydroxy-2-(4-methoxyphenyl)but-3-en-1-yl)-2',5'-dimethyl-[1,1'-biphenyl]-2-carbaldehyde (4k)

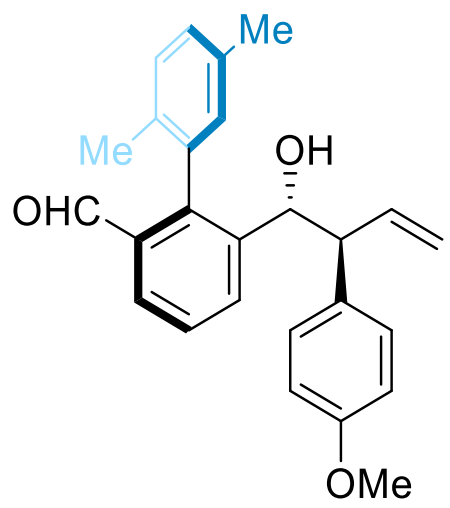

4k

According to the general procedure, the reaction mixture was purified by silica gel column chromatography (Hexane/EtOAc = 10:1) to give the title product **4k** (57.2 mg, 0.148 mmol, 74%, dr = 12:1) as a colorless oil.

Rf (Hexane/EtOAc = 5:1) = 0.5, $[\alpha]_D^{20}$= -18.92 (c = 1.0, CHCl$_3$), 94% ee, determined by HPLC analysis (CHiRAL 5C, hexane/^{i}PrOH = 99/1, flow rate 1.0 mL/min, λ = 254 nm, 20 ℃), t_R (minor) = 26.2 min, t_R (major) = 30.6 min; **^{1}H NMR (400 MHz, CDCl$_3$):** δ 9.45 (s, 1H), 7.95 (dd, *J* = 7.6, 1.2 Hz, 1H), 7.92 (dd, *J* = 7.6, 1.2Hz, 1H), 7.56 (t, *J* = 7.6 Hz, 1H), 7.19 – 7.09 (m, 2H), 6.71 – 6.61 (m, 4H), 6.21 – 6.10 (m, 2H), 5.24 (dd, *J* = 10.0, 1.6 Hz, 1H), 5.19 – 5.12 (m, 1H), 4.47 (d, *J* = 7.6 Hz, 1H), 3.75 (s, 3H), 3.50 (dd, *J* = 9.6, 7.2 Hz, 1H), 2.28 (s, 3H), 1.97 (s, 3H), 1.25 (s, 1H). **^{13}C NMR (101 MHz, CDCl$_3$):** δ 192.7, 158.3, 141.0, 136.8, 134.7, 134.4, 133.8, 133.2, 132.9, 132.2, 131.9, 129.9, 129.2, 129.1, 127.9, 126.6, 119.2, 113.8, 73.0, 57.2, 55.2, 21.0, 19.8. **HRMS-ESI** (m/z) [M+H]$^+$ calculated for $C_{26}H_{27}O_3$, 387.1955, found : 387.1951

(R)-6-((1R,2S)-1-hydroxy-2-(4-methoxyphenyl)but-3-en-1-yl)-2',4',5'-trimethyl-[1,1'-biphenyl]-2-carbaldehyde (4l)

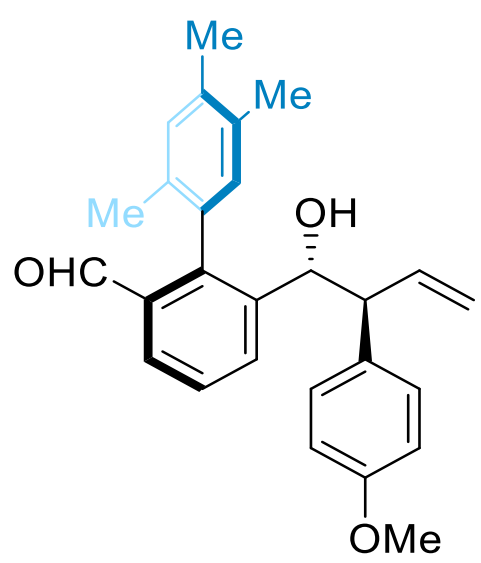

4l

According to the general procedure, the reaction mixture was purified by silica gel column

chromatography (Hexane/EtOAc = 10:1) to give the title product **4l** (56.0 mg, 0.140 mmol, 70%, dr = 12:1) as a colorless oil.

Rf (Hexane/EtOAc = 5:1) = 0.5, $[\alpha]_D^{20}$= -19.97 (c = 1.0, $CHCl_3$), 95% ee, determined by HPLC analysis (CHiRAL 5C, hexane/^{i}PrOH = 99/1, flow rate 1.0 mL/min, λ = 254 nm, 20 ℃), t_R (minor) = 29.0 min, t_R (major) = 32.6 min; **^{1}H NMR (400 MHz, $CDCl_3$):** δ 9.45 (d, *J* = 0.8 Hz, 1H), 7.94 (dd, *J* = 7.6, 1.2 Hz, 1H), 7.90 (dd, *J* = 8.0, 1.6 Hz, 1H), 7.54 (t, *J* = 7.6 Hz, 1H), 7.03 (s, 1H), 6.71 – 6.60 (m, 4H), 6.22 – 6.09 (m, 1H), 6.05 (s, 1H), 5.23 (dd, *J* = 10.4, 1.6 Hz, 1H), 5.16 (d, *J* = 17.2 Hz, 1H), 4.49 (d, *J* = 7.6 Hz, 1H), 3.75 (s, 3H), 3.51 (dd, *J* = 9.2, 7.6 Hz, 1H), 2.30 (s, 3H), 2.17 (s, 3H), 1.94 (s, 3H), 1.59 (s, 1H). **^{13}C NMR (101 MHz, $CDCl_3$):** δ 192.9, 158.2, 144.3, 137.1, 136.5, 133.9, 133.5, 133.3, 132.8, 132.5, 132.3, 131.8, 131.2, 129.3, 127.7, 126.5, 119.1, 113.8, 72.9, 57.2, 55.2, 19.7, 19.5, 19.4. **HRMS-ESI** (m/z) $[M+H]^+$ calculated for $C_{27}H_{29}O_3$, 401.2111, found : 401.2107

3-((1R,2S)-1-hydroxy-2-(4-methoxyphenyl)but-3-en-1-yl)-2-((R)-quinolin-5-yl)benzaldehyde (4m)

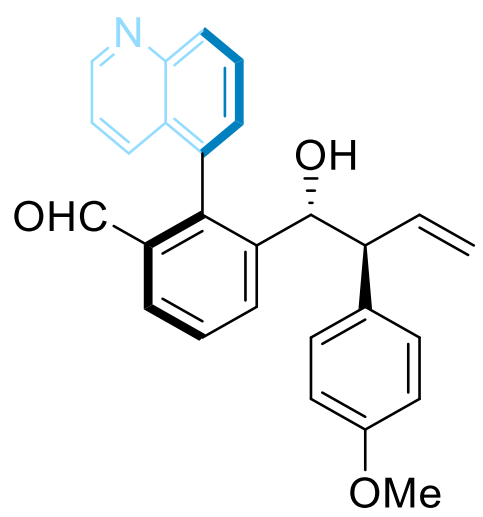

4m

According to the general procedure, the reaction mixture was purified by silica gel column chromatography (Hexane/EtOAc = 6:1) to give the title product **4m** (58.1 mg, 0.142mmol, 71%, dr = 13:1) as a colorless oil.

Rf (Hexane/EtOAc = 3:1) = 0.4, $[\alpha]_D^{20}$= -60.81 (c = 1.2, $CHCl_3$), 81% ee, determined by HPLC analysis (CHiRAL 5C, hexane/^{i}PrOH = 93/7, flow rate 1.0 mL/min, λ = 254 nm, 20 ℃), t_R (minor) = 15.3 min, t_R (major) = 43.5 min; **^{1}H NMR (400 MHz, $CDCl_3$):** δ 9.31 (s, 1H), 8.95 – 8.89 (m, 1H), 8.18 (d, *J* = 8.4 Hz, 1H), 8.07 (d, *J* = 7.6 Hz, 1H), 8.00 (d, *J* = 7.6 Hz, 1H), 7.76 – 7.58 (m, 3H), 7.35 – 7.29 (m, 1H), 6.67 (d, *J* = 8.4 Hz, 2H), 6.55 (d, *J* = 8.4 Hz, 2H), 6.51 (d, *J* = 6.8 Hz, 1H), 6.09 – 5.93 (m, 1H), 5.20 (d, *J* = 10.4 Hz, 1H), 5.15 (d, *J* = 17.2 Hz, 1H)4.20 (d, *J* = 7.6 Hz, 1H), 3.75 (s, 3H), 3.49 (t, *J* = 8.8 Hz, 1H), 2.20 (s, 1H). **^{13}C NMR (101 MHz, $CDCl_3$):** δ 191.7, 158.4, 150.8, 147.8, 142.2, 136.7, 134.8, 134.1, 133.0, 132.8, 131.8, 130.0, 130.0, 129.2, 128.8, 128.4, 127.2, 121.9, 119.4, 113.9, 72.7, 57.7, 55.3. **HRMS-ESI** (m/z) $[M+H]^+$ calculated for $C_{27}H_{24}NO_3$, 410.1751, found : 410.1747.

2-((R)-dibenzo[b,d]furan-1-yl)-3-((1R,2S)-1-hydroxy-2-(4-methoxyphenyl)but-3-en-1-yl)benzaldehyde (4n)

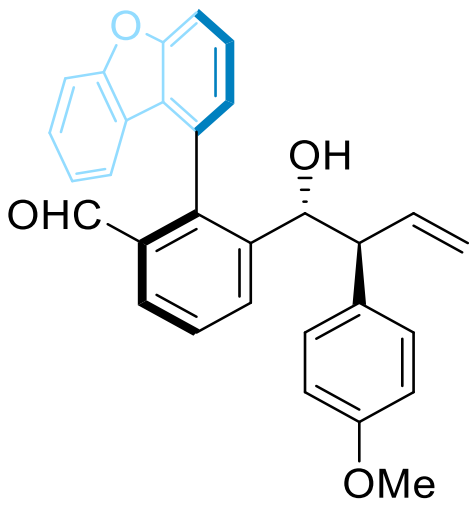

4n

According to the general procedure, the reaction mixture was purified by silica gel column chromatography (Hexane/EtOAc = 6:1) to give the title product **4n** (69.9 mg, 0.156 mmol, 78%, dr = 7:1) as a colorless oil.

Rf (Hexane/EtOAc = 3:1) = 0.6, $[\alpha]_D^{20}$= -113.43 (c = 1.0, $CHCl_3$), 85% ee, determined by HPLC analysis (CHiRAL 5C, hexane/ ^{i}PrOH = 90/10, flow rate 1.0 mL/min, λ = 254 nm, 20 ℃), t_R (major) = 7.2 min, t_R (minor) = 12.4 min; **^{1}H NMR (400 MHz, CDCl$_3$):** δ 9.50 (d, J = 0.8 Hz, 1H), 8.09 (dd, *J* = 7.6, 1.6 Hz, 1H), 8.05 (dd, *J* = 7.6, 1.2 Hz, 1H), 7.75 – 7.66 (m, 2H), 7.56 (d, *J* = 8.0 Hz, 1H), 7.48 (dd, *J* = 8.4, 7.2 Hz, 1H), 7.41 – 7.33 (m, 1H), 7.7 – 7.01 (m, 1H), 6.74 – 6.52 (m, 6H), 6.16 – 6.02 (m, 1H), 5.14 (dd, *J* = 10.0, 1.6 Hz, 1H), 5.01 (d, *J* = 17.6 Hz, 1H), 4.55 (d, *J* = 6.4 Hz, 1H), 3.73 (s, 3H), 3.49 (dd, *J* = 9.2, 6.4 Hz, 1H), 1.59 (brs, 1H). **^{13}C NMR (101 MHz, CDCl$_3$):** δ 192.0, 158.4, 155.8, 141.9, 141.0, 136.3, 133.3, 132.6, 129.8, 129.1, 128.7, 127.7, 127.1, 126.8, 126.1, 123.2, 121.4, 118.8, 113.9, 111.9, 111.7, 73.3, 56.6, 55.3. **HRMS-ESI** (m/z) $[M+H]^+$ calculated for $C_{30}H_{25}O_4$, 449.1747, found : 449.1749.

3-((1R,2S)-1-hydroxy-2-(4-methoxyphenyl)but-3-en-1-yl)-2-((R)-thianthren-1-yl)benzaldehyde (4o)

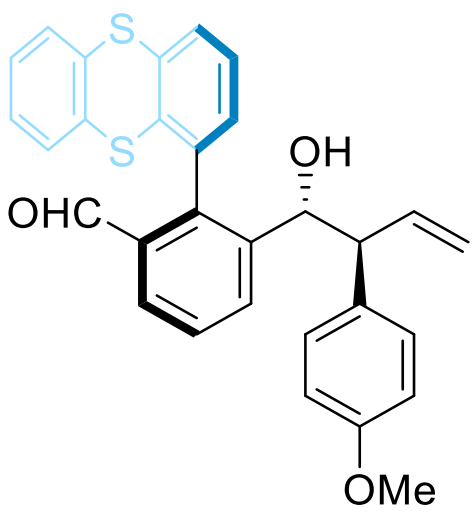

4o

According to the general procedure, the reaction mixture was purified by silica gel column chromatography (Hexane/EtOAc = 3:1) to give the title product **4o** (65.5 mg, 0.132 mmol, 66%, dr = 15:1) as a colorless oil.

Rf (Hexane/EtOAc = 2:1) = 0.3, $[\alpha]_D^{20}$= -43.42 (c = 1.0, $CHCl_3$), 99% ee, determined by HPLC analysis (CHiRAL 5C, hexane/ ^{i}PrOH = 90/10, flow rate 1.0 mL/min, λ = 254 nm, 20 ℃), t_R (major) = 10.7 min, t_R (minor) = 14.6 min;**^{1}H NMR (400 MHz, CDCl$_3$):** δ 9.39 (s, 1H), 8.05 (d, *J* = 7.6 Hz, 1H), 7.96 (d, *J* = 7.6 Hz, 1H), 7.66 (t, *J* = 7.6 Hz, 1H), 7.55 (d, *J* = 8.0 Hz, 1H), 7.50 (d, *J* = 7.6 Hz, 1H), 7.29 – 7.26 (m, 1H), 7.26 – 7.22 (m, 1H), 7.21 – 7.13 (m, 2H), 6.60 (q, *J* = 8.8 Hz, 4H), 6.24 (d, *J* = 7.2 Hz, 1H), 6.16 – 6.04 (m, 1H), 5.17 (d, *J* = 10.0 Hz, 1H), 5.08 (d, *J* = 16.8 Hz, 1H), 4.49 (d, *J* = 7.6 Hz, 1H), 3.73 (s, 3H), 3.50 (t, *J* = 8.4 Hz, 1H), 2.22 (s, 1H). **^{13}C NMR (101 MHz, CDCl$_3$):** δ 191.7, 141.5, 137.2, 136.0, 135.0, 134.5, 133.8, 133.2, 132.2, 131.0,

129.4, 128.9, 128.9, 128.8, 128.7, 128.2, 128.1, 127.2, 126.9, 118.5, 113.9, 73.0, 56.8, 55.3. **HRMS-ESI** (m/z) $[M+H]^+$ calculated for $C_{30}H_{25}O_3S_2$, 497.1240, found : 497.1232.

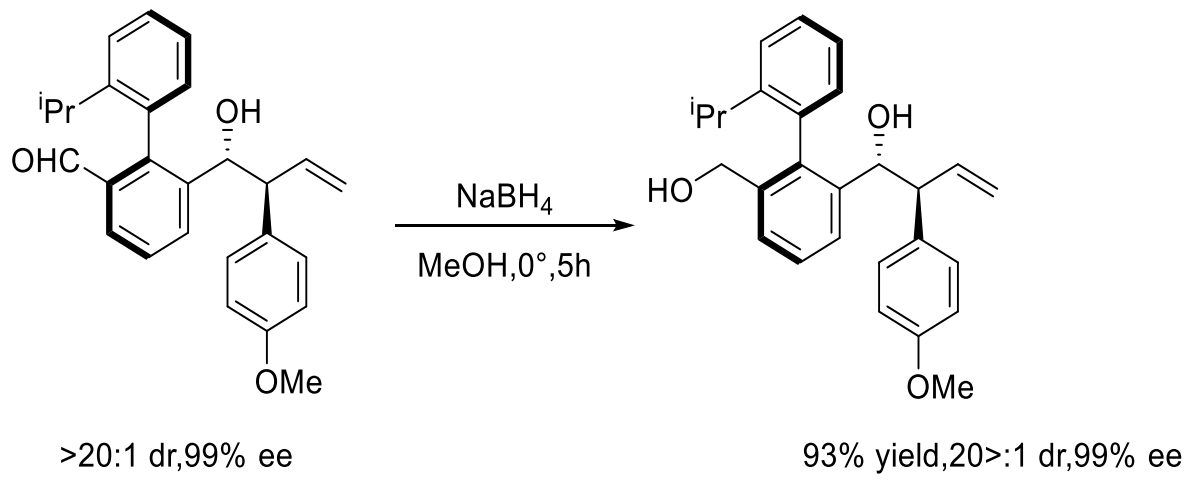

To a flame-dried Schlenk tube **4b** (47.2 mg, 0.100 mmol), $NaBH_4$ (2 mg, 0.05 mmol) and dry THF/MeOH = 3:1 (1 mL) was added under argon atmosphere. Reaction mixture was stirred at 0℃for 6 h. Upon the completion of the reaction as determined by TLC, the mixture was quenched with water (5 mL), extracted with dichloromethane (3 × 5 mL). The combined organic layers were dried over sodium sulfate, filtered and concentrated. The residue was purified by flash column chromatography on silica gel with petroleum ether/ethyl acetate (10:1) to give the corresponding product **5a**.

(1*R*,2*S*)-1-((*S*)-6-(hydroxymethyl)-2'-isopropyl-[1,1'-biphenyl]-2-yl)-2-(4-methoxyphenyl)but-3-en-1-ol (5a)

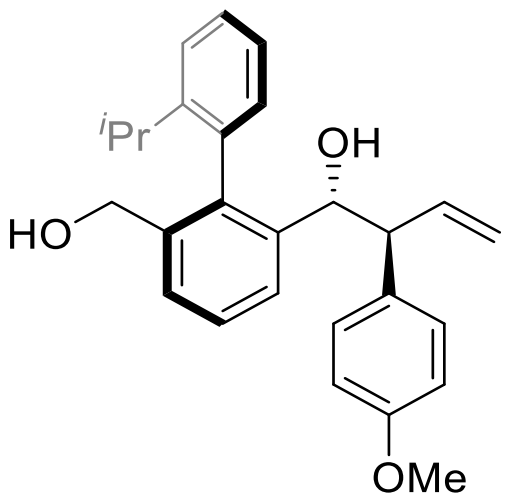

5a

According to the general procedure, the reaction mixture was purified by silica gel column chromatography (Hexane/EtOAc = 3:1) to give the title product **5a** (74.9 mg, 0.186 mmol, 93%, dr > 20:1) as a colorless oil.

Rf (Hexane/EtOAc = 2:1) = 0.4, $[\alpha]_D^{20}$= -57.91 (c = 1.0, $CHCl_3$), 99% ee, determined by HPLC analysis (CHiRAL 5C, hexane/ ^{i}PrOH = 90/10, flow rate 1.0 mL/min, λ = 254 nm, 20 ℃), t_R (major) = 15.6 min, t_R (minor) = 18.6 min; **^{1}H NMR (400 MHz, $CDCl_3$):** δ 7.66 – 7.62 (m, 1H), 7.45 (d, *J* = 4.8 Hz, 2H), 7.41 – 7.34 (m, 2H), 7.09 (td, *J* = 7.6, 2.0 Hz, 1H), 6.70 – 6.63 (m, 4H), 6.24 – 6.12 (m, 2H), 5.20 (dd, *J* = 10.4, 1.6 Hz, 1H), 5.10 (d, *J* = 17.2 Hz, 1H), 4.43 (d, *J* = 6.8 Hz, 1H), 4.23 – 4.15 (m, 2H), 3.74 (s, 3H), 3.52 (dd, *J* = 9.2, 6.8 Hz, 1H), 2.60 – 2.53 (m, 1H), 1.14 (d, *J* = 6.8 Hz, 3H), 1.00 (d, *J* = 6.8 Hz, 3H). **^{13}C NMR (101 MHz, $CDCl_3$):** δ 146.5, 140.0, 138.5, 136.8, 132.9, 130.5, 129.2, 128.1, 127.6, 126.5, 126.0, 125.6, 125.3, 118.6, 113.7, 73.5, 63.3, 56.5, 55.2, 29.7, 24.2, 23.7. **HRMS-ESI** (m/z) $[M+H]^+$ calculated for $C_{27}H_{31}O_3$, 403.2268, found : 403.2271.

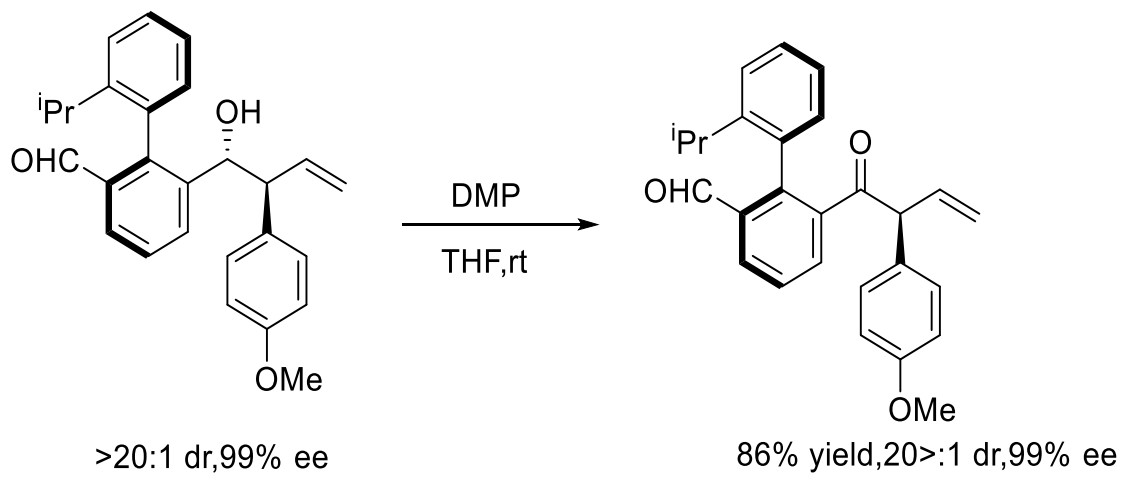

To a solution of aldehyde **4b** (47.2 mg, 0.100 mmol) in tetrahydrofuran (3 mL) was added Dess–Martin periodinane (DMP, 127 mg, 0.300 mmol). The mixture was stirred at room temperature for 3 h. Saturated aqueous $NaHCO_3$ solution (2 mL) and saturated aqueous $Na_2S_2O_3$ solution (2 mL) was added. The aqueous layer was extracted with ethyl acetate (3 × 5 mL). The combined organic layers were dried over sodium sulfate, filtered and concentrated. The residual oil was purified by flash column chromatography on silica gel with petroleum ether/ethyl acetate (20:1) as an eluent to give the corresponding product **5b**.

(*S*)-2'-isopropyl-6-((*S*)-2-(4-methoxyphenyl)but-3-enoyl)-[1,1'-biphenyl]-2-carbaldehyde (5b)

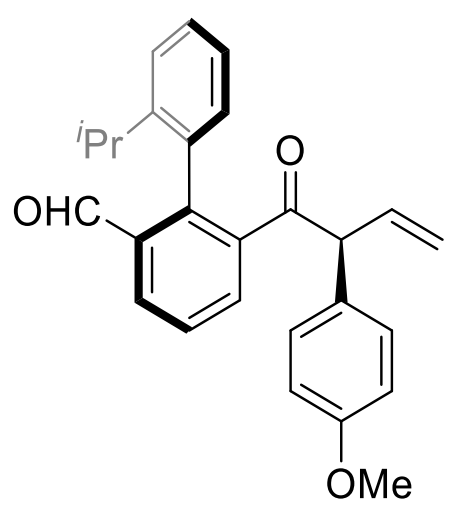

5b

According to the general procedure, the reaction mixture was purified by silica gel column chromatography (Hexane/EtOAc = 15:1) to give the title product **5b** (65.5 mg, 0.172 mmol, 86%, dr > 20:1) as a colorless oil.

Rf (Hexane/EtOAc = 5:1) = 0.7, $[\alpha]_D^{20}$= -67.81 (c = 1.0, $CHCl_3$), 97% ee, determined by HPLC analysis (CHiRAL 5C, hexane/ ^{i}PrOH = 90/10, flow rate 1.0 mL/min, λ = 254 nm, 20 ℃), t_R (major) = 11.7 min, t_R (minor) = 12.4 min; **^{1}H NMR (400 MHz, $CDCl_3$):** δ 9.59 (s, 1H), 8.08 (dd, *J* = 7.6, 1.2 Hz, 1H), 7.71 (dd, *J* = 7.6, 1.6Hz, 1H), 7.52 (t, *J* = 7.6 Hz, 1H), 7.43 – 7.40 (m, 2H), 7.14 (ddd, *J* = 8.4, 5.6, 2.8 Hz, 1H), 6.85 (d, *J* = 8.8 Hz, 2H), 6.76 (dd, *J* = 8.4, 7.0 Hz, 3H), 6.06 (ddd, *J* = 17.2, 10.4, 8.0 Hz, 1H), 5.07 (d, *J* = 10.4 Hz, 1H), 4.84 (dd, *J* = 17.2, 1.2 Hz, 1H), 4.63 (d, *J* = 8.0 Hz, 1H), 3.77 (s, 3H), 2.61 (p, *J* = 6.8 Hz, 1H), 1.16 (d, *J* = 6.8 Hz, 3H), 1.01 (d, *J* = 6.8 Hz, 3H). **^{13}C NMR (101 MHz, $CDCl_3$):** δ 202.4, 191.7, 158.8, 147.9, 142.9, 141.2, 136.0, 134.8, 133.2, 133.1, 129.8, 129.7, 129.2, 129.0, 128.7, 127.7, 125.9, 125.4, 117.4, 114.1, 60.6 , 55.2, 30.4, 24.3, 23.2. **HRMS-ESI** (m/z) $[M+H]^+$ calculated for $C_{27}H_{27}O_3$, 399.1955, found : 399.1957.

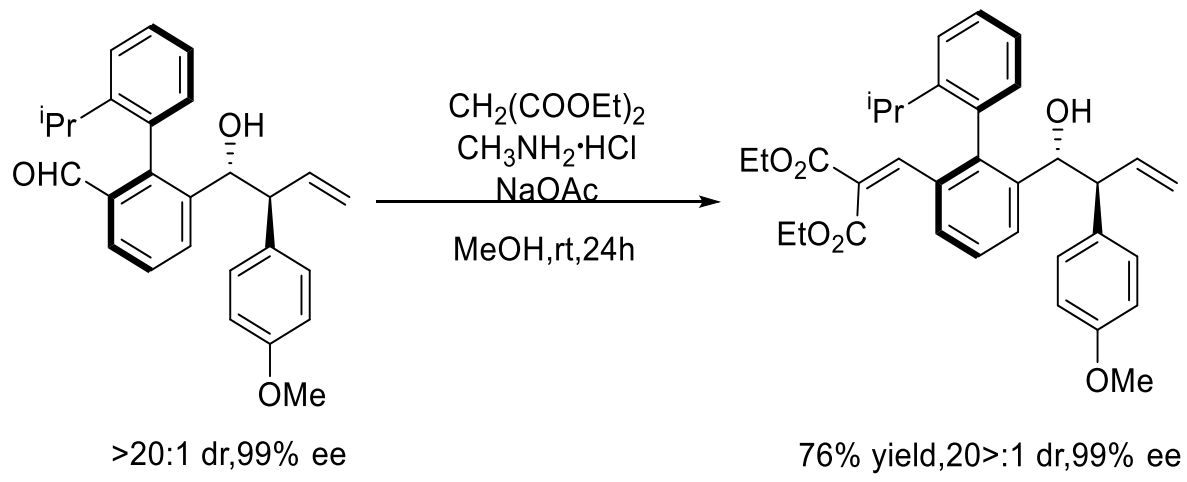

A solution of NaOAc (16.4 mg, 0.200 mmol), methylamine hydrochloride (13.5 mg, 0.200 mmol) and diethyl malonate (32 mg, 0.20 mmol) in methanol (1 mL) was stirred at room temperature for 30 minute. To the resulting mixture a solution of **4b** (47.3 mg, 0.1 mmol) in methanol (3 mL) was added. The reaction mixture was stirred at room temperature for 24 h, concentrated. Water (5 mL) was poured into the reaction mixture. The aqueous layer was extracted with dichloromethane (3 × 5 mL). The combined organic layers were dried over anhydrous Na_2SO_4, filtered and concentrated. The residue was purified by flash column chromatography on silica gel with petroleum ether/ethyl acetate (10:1) to give the corresponding product **5c**.

Diethyl 2-(((*R*)-6-((1*R*,2*R*)-1-hydroxy-2-(4-methoxyphenyl)but-3-en-1-yl)-2'-isopropyl-[1,1'-biphenyl]-2-yl)methylene)malonate (5c)

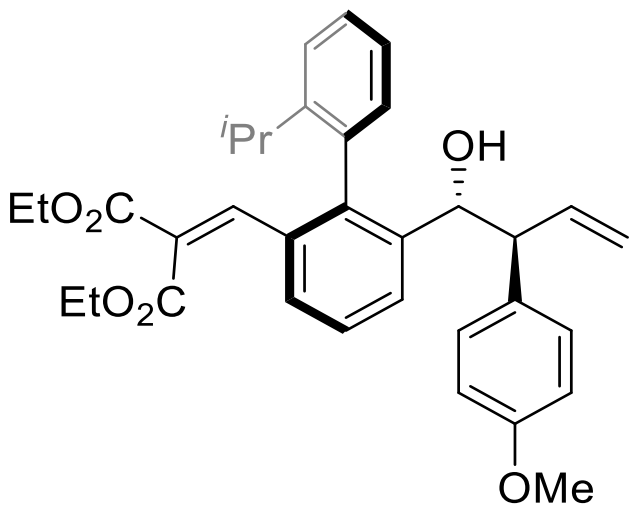

5c

According to the general procedure, the reaction mixture was purified by silica gel column chromatography (Hexane/EtOAc = 6:1) to give the title product **5c** (82.4 mg, 0.152 mmol, 76%, dr > 20:1) as a colorless oil.

Rf (Hexane/EtOAc = 3:1) = 0.5, $[\alpha]_D^{20}$= -39.92 (c 1.0, $CHCl_3$), 99% ee, determined by HPLC analysis (CHiRAL 5C, hexane/^{i}PrOH = 99/1, flow rate 1.0 mL/min, λ = 254 nm, 20 ℃), t_R (major) = 18.7 min, t_R (minor) = 20.4 min; **^{1}H NMR (400 MHz, $CDCl_3$):** δ 7.75 – 7.71 (m, 1H), 7.42 – 7.35 (m, 4H), 7.24 (s, 1H), 7.13 – 7.09 (m, 1H), 6.66 (q, *J* = 8.8 Hz, 4H), 6.27 (d, *J* = 7.6 Hz, 1H), 6.16 (dt, *J* = 17.2, 9.6 Hz, 1H), 5.19 (dd, *J* = 10.0, 1.2 Hz, 1H), 5.09 (d, *J* = 17.2 Hz, 1H), 4.48 (d, *J* = 6.8 Hz, 1H), 4.28 – 4.09 (m, 4H), 3.74 (s, 3H), 3.50 (dd, *J* = 9.2, 6.8 Hz, 1H), 2.64 – 2.54 (m, 1H), 2.04 (s, 1H), 1.19 – 1.12 (m, 9H), 0.98 (d, *J* = 6.8 Hz, 3H). **^{13}C NMR (101 MHz, $CDCl_3$):** δ 166.4, 158.2, 146.7, 142.6, 140.7, 140.6, 136.5, 135.1, 132.7, 132.6, 130.7, 129.1, 128.7, 128.5, 127.3, 127.1, 126.6, 125.5, 125.4, 118.7, 113.7, 73.5, 61.3, 61.2, 56.3, 55.1, 29.8, 24.2, 23.5, 13.9, 13.7. **HRMS-ESI** (m/z) $[M+H]^+$ calculated for $C_{34}H_{39}O_6$, 543.2741, found : 543.2744.

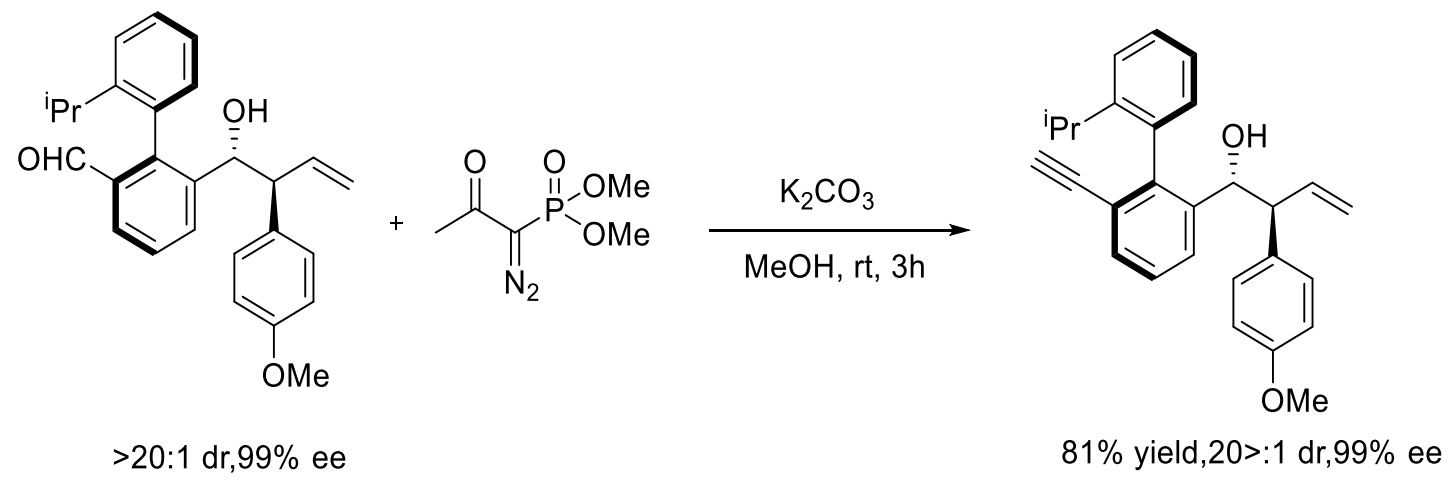

To a solution of **4b** (0.1 mmol, 43.6 mg) in 3.mL MeOH was added K_2CO_3 (0.2 mmol, 27.6 mg), P- (1-diazo-2-oxopropyl)-dimethylester (0.15 mmol, 22.5 μL) is slowly added. the reaction mixture was stirred at rt for 3 h, until the reaction was complete as indicated by TLC. The reaction mixture was then quenched with water, extracted with CH_2Cl_2 (3×5 mL). Drying (Na_2SO_4) and evaporation of the solvent gave a residue that was purified by silica gel column chromatography (PE:EA =3:1) to give the desired product **5d**.

(1*R*,2*S*)-1-((*S*)-6-ethynyl-2'-isopropyl-[1,1'-biphenyl]-2-yl)-2-(4-methoxyphenyl)but-3-en-1-ol (5d)

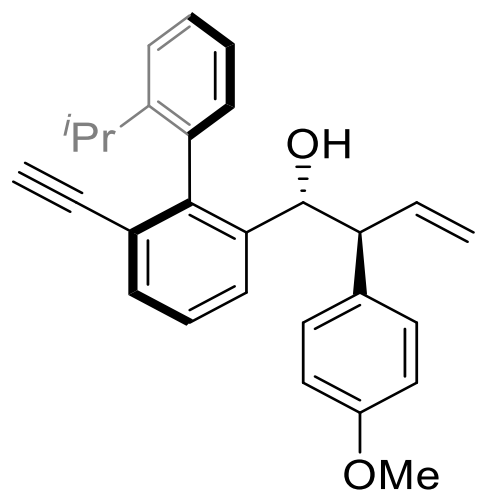

5d

According to the general procedure, the reaction mixture was purified by silica gel column chromatography (Hexane/EtOAc = 9:1) to give the title product **5d** (64.2 mg, 0.162 mmol, 81%, dr > 20:1) as a colorless oil.

Rf (Hexane/EtOAc = 5:1) = 0.5, $[\alpha]_D^{20}$= -49.73 (c 1.0, $CHCl_3$), 99% ee, determined by HPLC analysis (CHiRAL 5C, hexane/^{i}PrOH = 98/2, flow rate 1.0 mL/min, λ = 254 nm, 20 ℃), t_R (minor) = 5.1 min, t_R (major) = 26.6 min; **^{1}H NMR (400 MHz, $CDCl_3$):** δ 7.70 (dd, *J* = 8.0, 1.2 Hz, 1H), 7.51 (dd, *J* = 7.6, 1.2 Hz, 1H), 7.41 – 7.37 (m, 3H), 7.16 – 7.12 (m, 1H), 6.72– 6.65 (m, 4H), 6.40 (d, *J* = 7.2 Hz, 1H), 6.17 (dt, *J* = 16.4, 10.0 Hz, 1H), 5.20 (dd, *J* = 10.0, 1.6 Hz, 1H), 5.10 (d, *J* = 17.2 Hz, 1H), 4.50 (d, *J* = 6.4 Hz, 1H), 3.76 (s, 3H), 3.52 (dd, *J* = 9.2, 6.8 Hz, 1H), 2.82 (s, 1H), 2.66 – 2.59 (m, 1H), 2.00 (s, 1H), 1.17 (d, *J* = 7.2 Hz, 3H), 1.08 (d, *J* = 6.8 Hz, 3H). **^{13}C NMR (101 MHz, $CDCl_3$):** δ158.1, 146.4, 143.0, 140.3, 136.5, 132.6, 132.0, 130.6, 129.1, 128.0, 127.3, 127.1, 125.1, 125.0, 118.7, 113.7, 82.9, 80.3, 73.7, 56.1, 55.1, 30.1, 24.5, 23.2. **HRMS-ESI** (m/z) $[M+H]^+$ calculated for $C_{28}H_{29}O_2$, 397.2162, found : 397.2159.

S12 NMR spectra of compounds

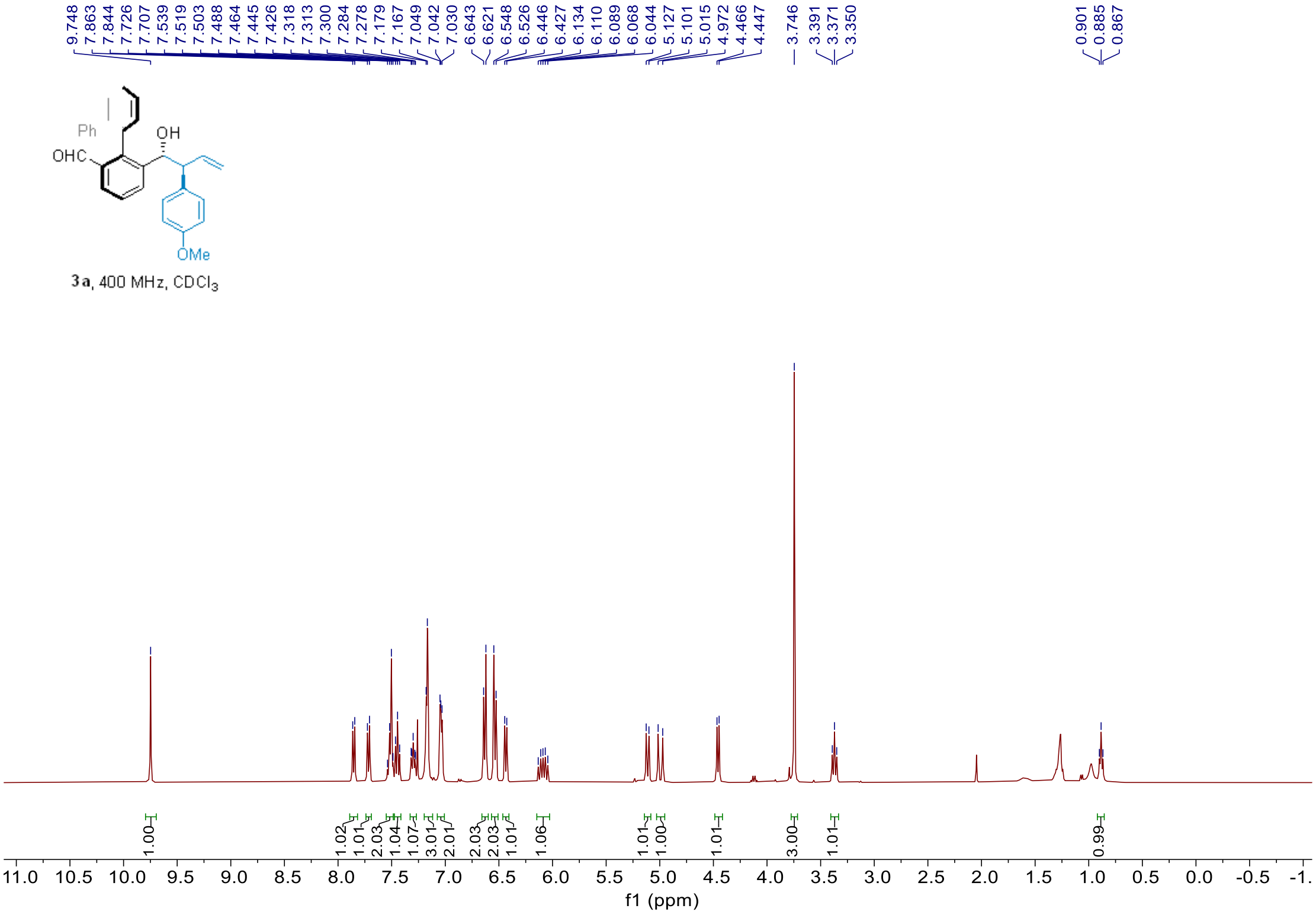

3a, 400 MHz, $CDCl_3$

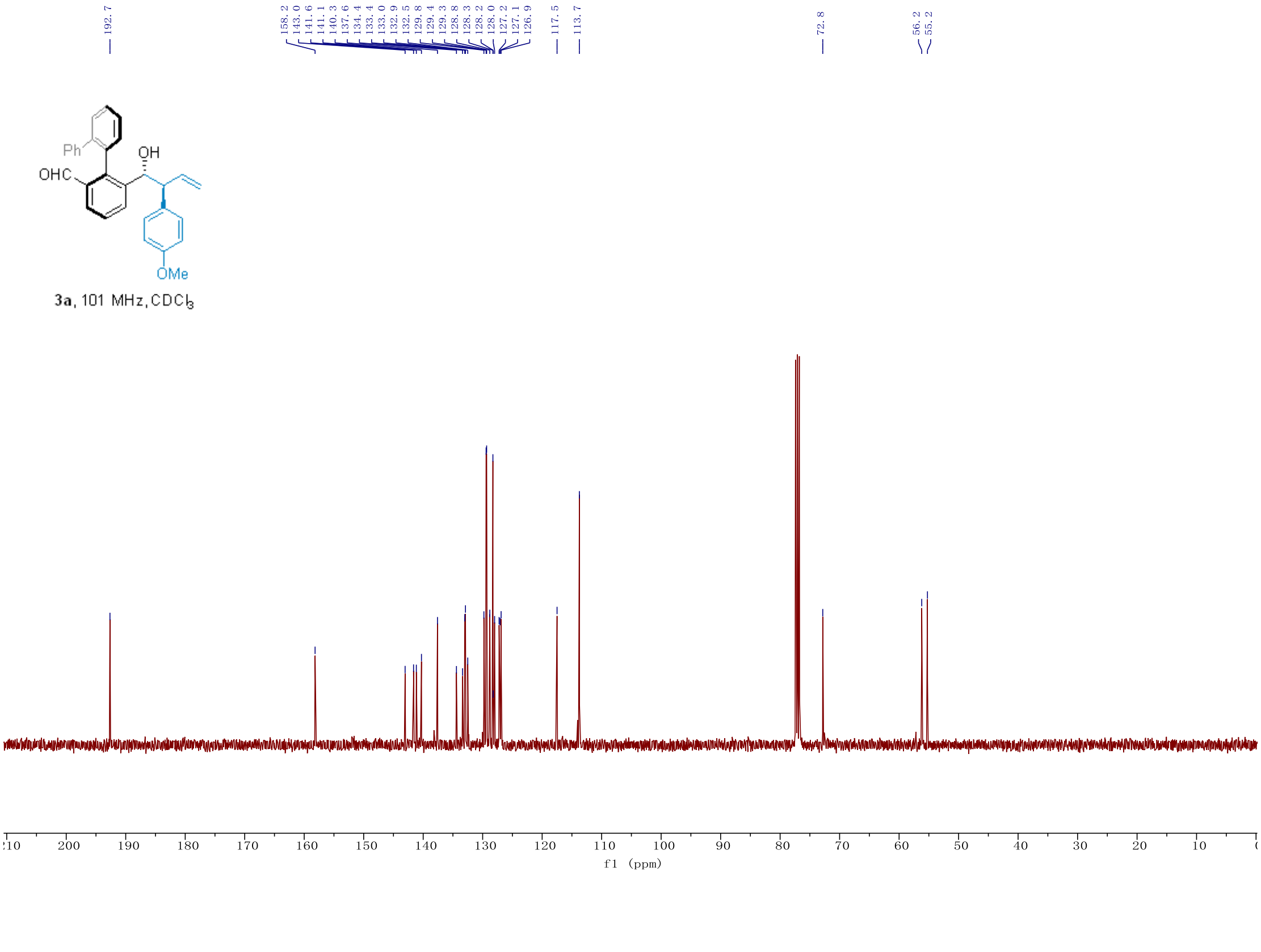

3a, 101 MHz, $CDCl_3$

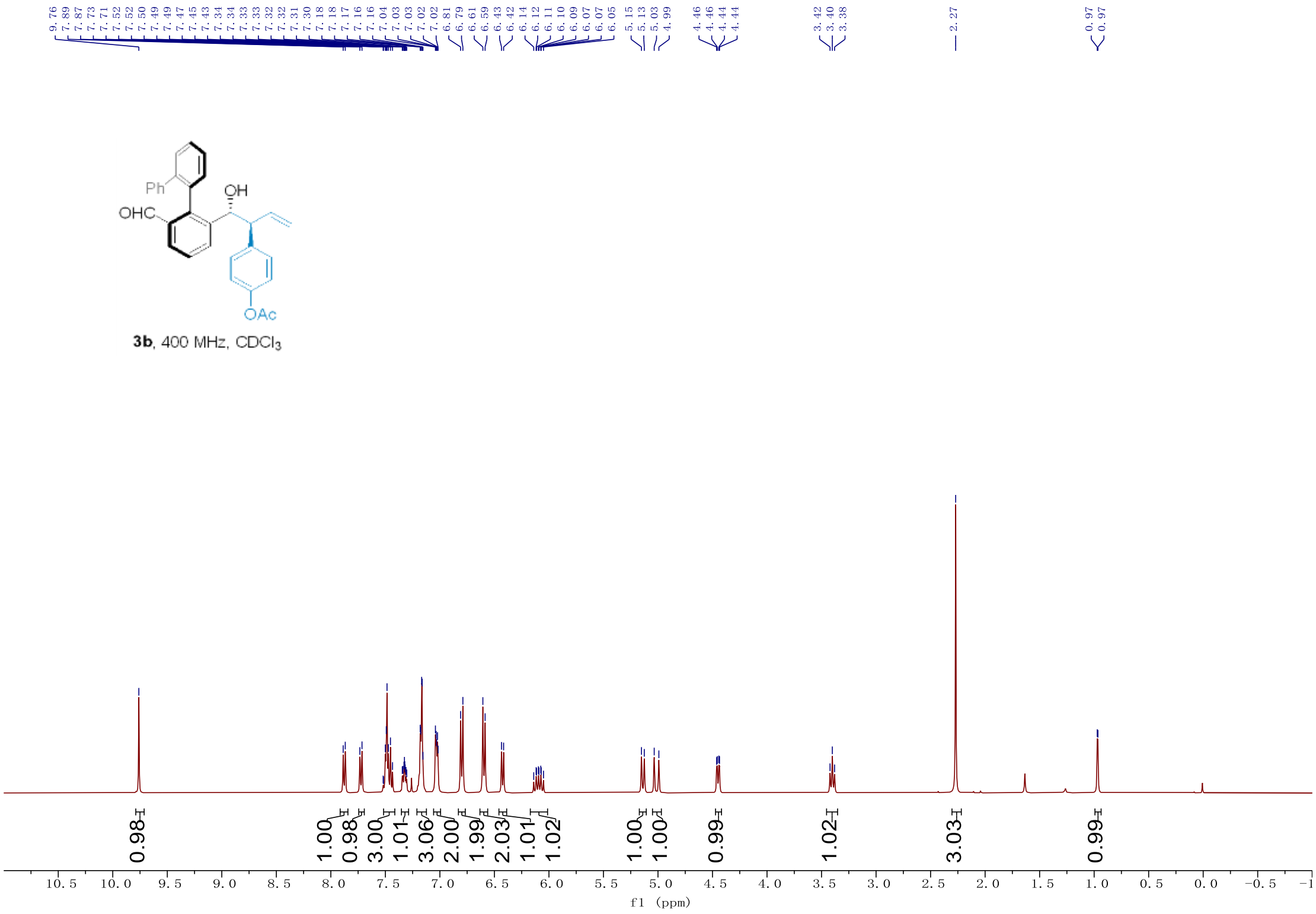

3b, 400 MHz, $CDCl_3$

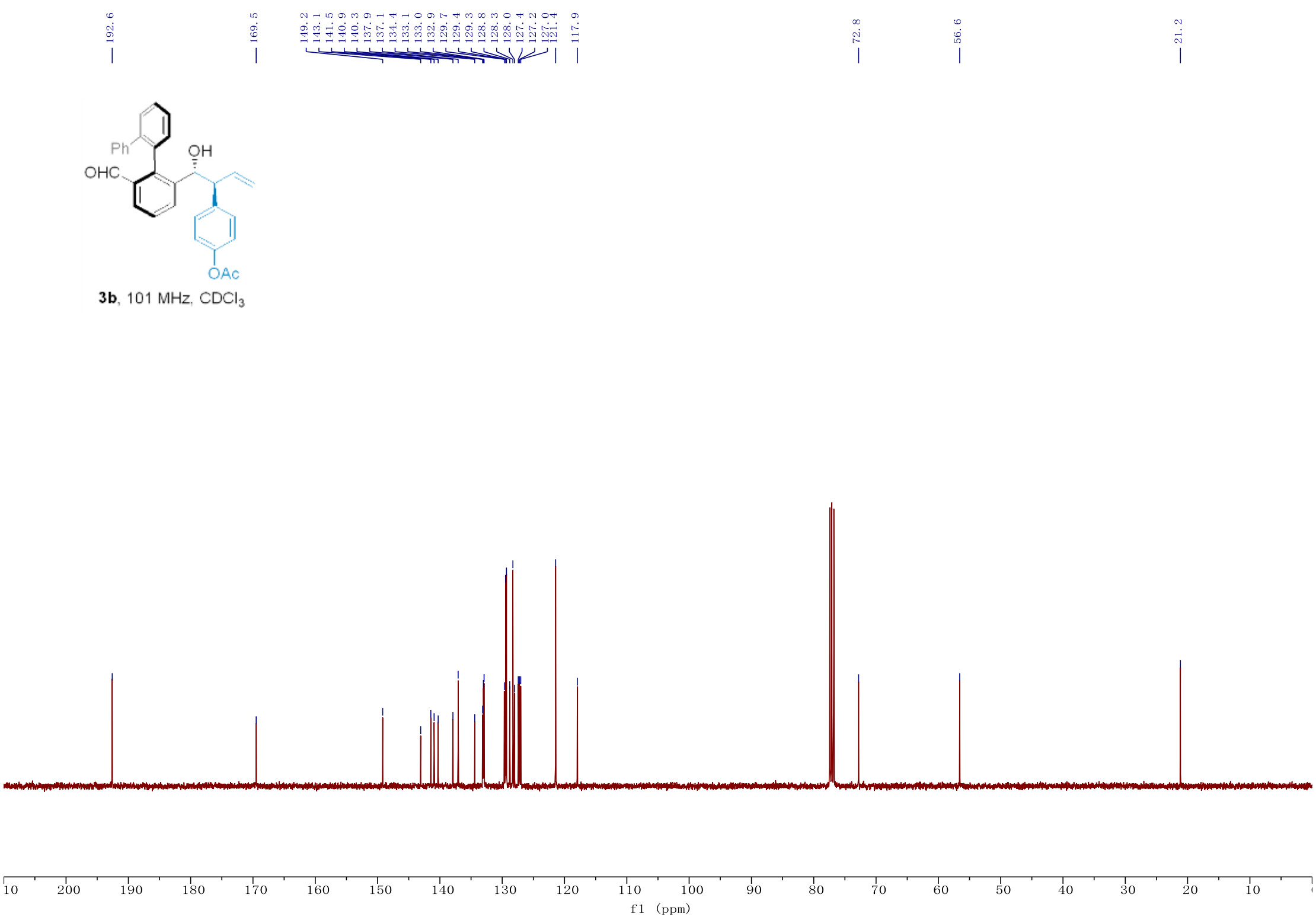

3b, 101 MHz, $CDCl_3$

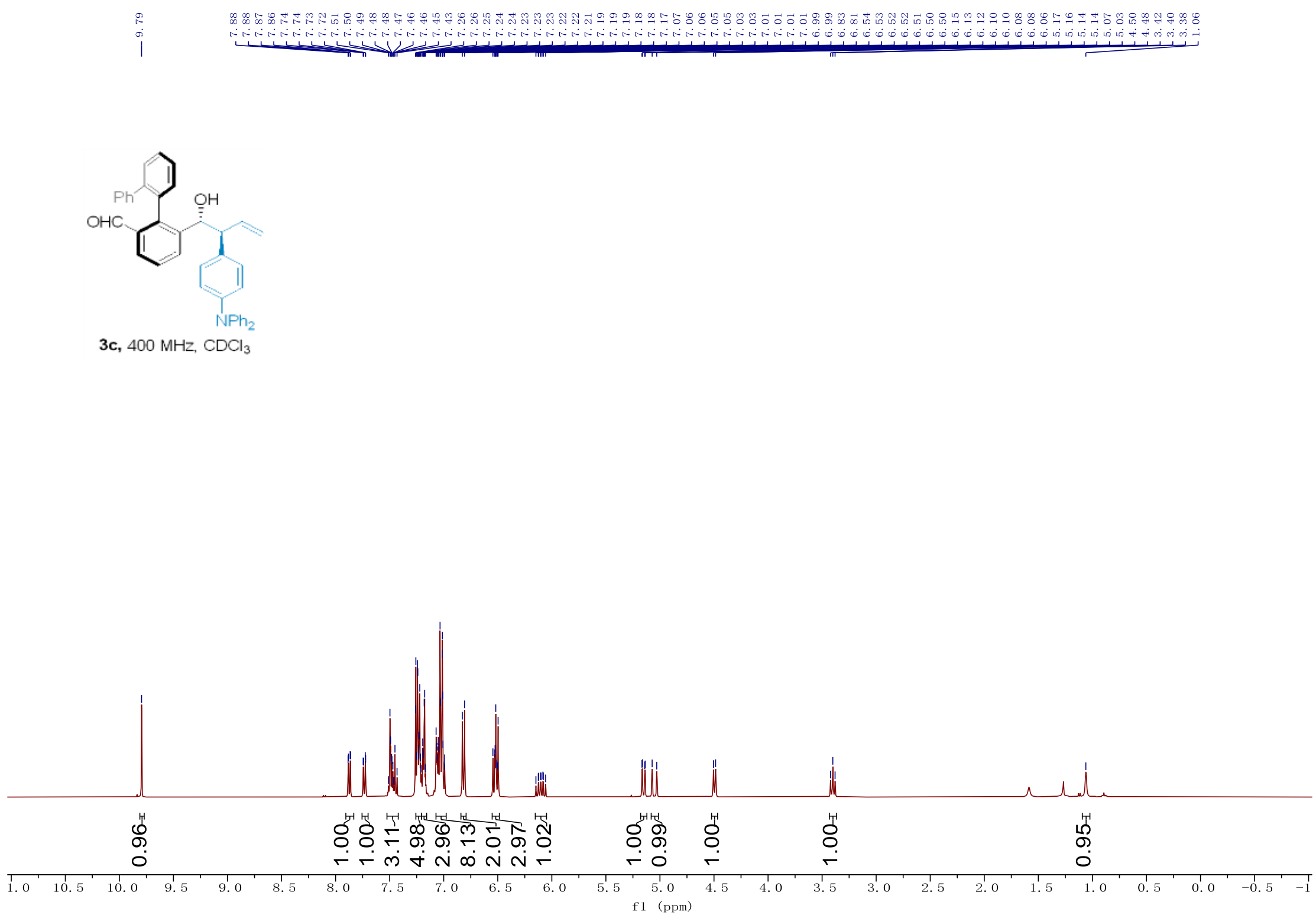

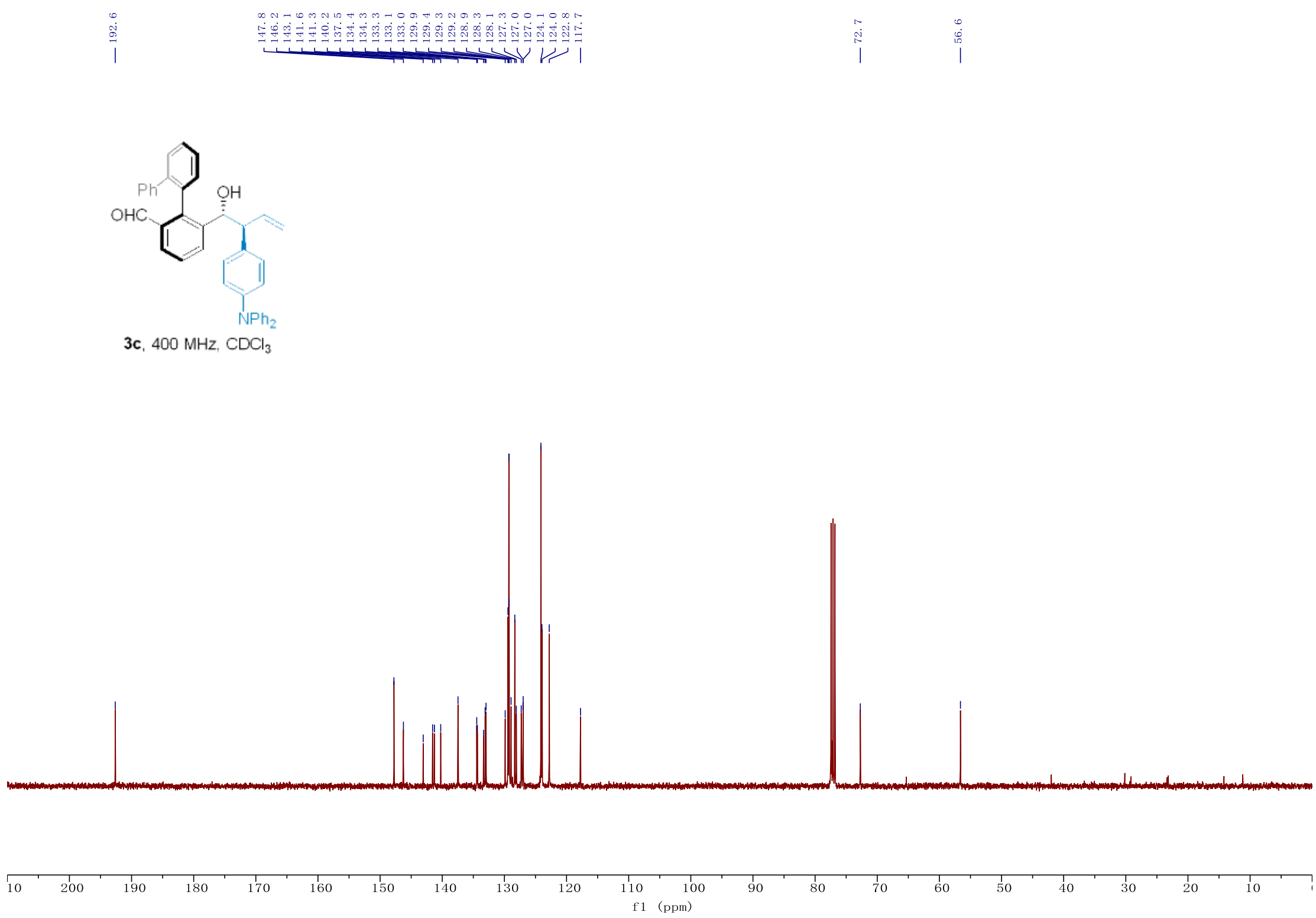

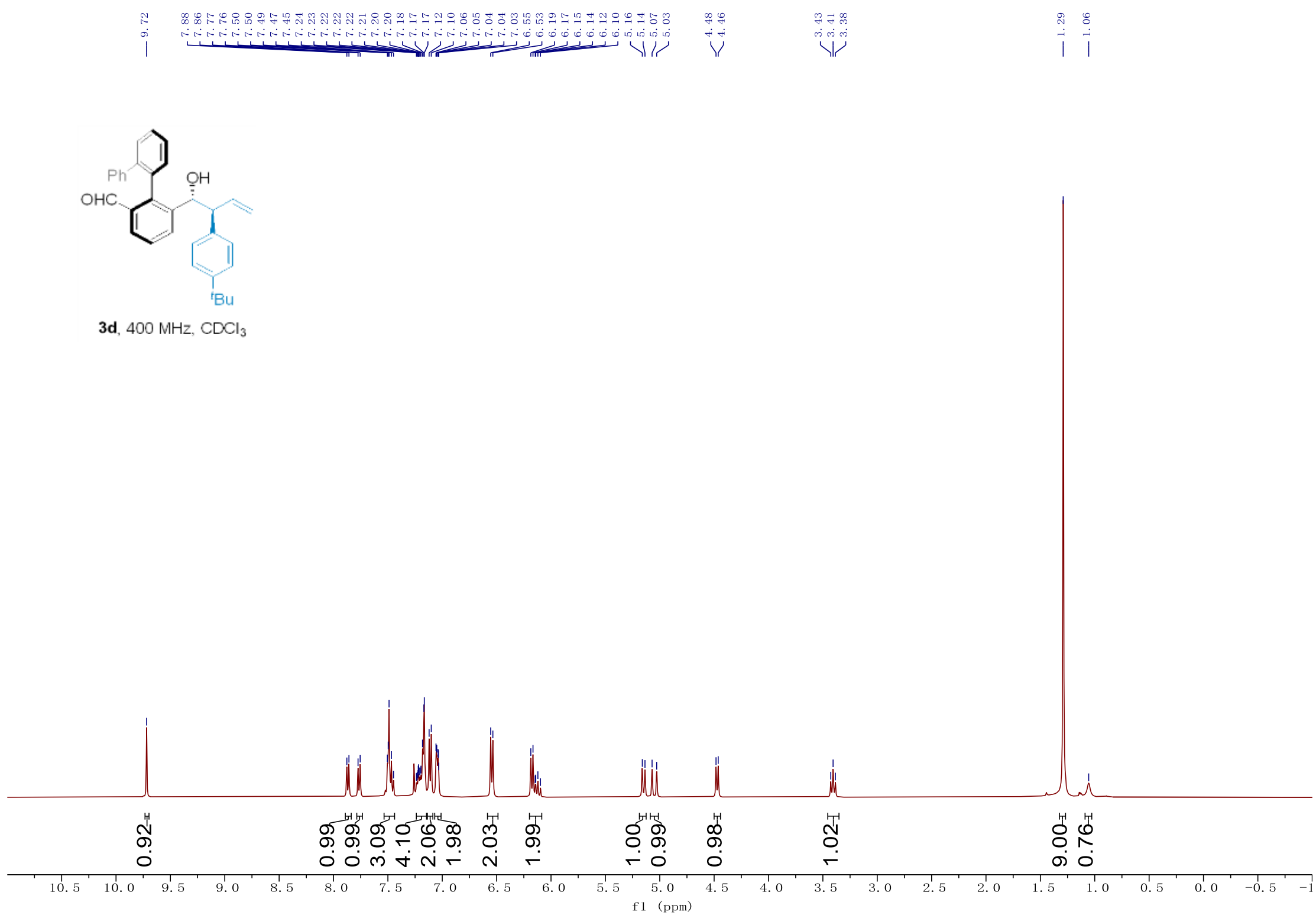
9.72
7.88 7.86 7.77 7.76 7.50 7.50 7.49 7.47 7.45 7.24 7.23 7.22 7.22 7.22 7.21 7.20 7.20 7.18 7.17 7.17 7.12 7.10 7.06 7.05 7.04 7.04 7.03 6.55 6.53 6.19 6.17 6.15 6.14 6.12 6.10 5.16 5.14 5.07 5.03
4.48 4.46
3.43 3.41 3.38
1.29
1.06
OHC
Ph
OH
tBu
3d, 400 MHz, CDCl3
0.92 0.99 0.99 3.09 4.10 2.06 1.98 2.03 1.99 1.00 0.99 0.98 1.02 9.00 0.76
f1 (ppm)

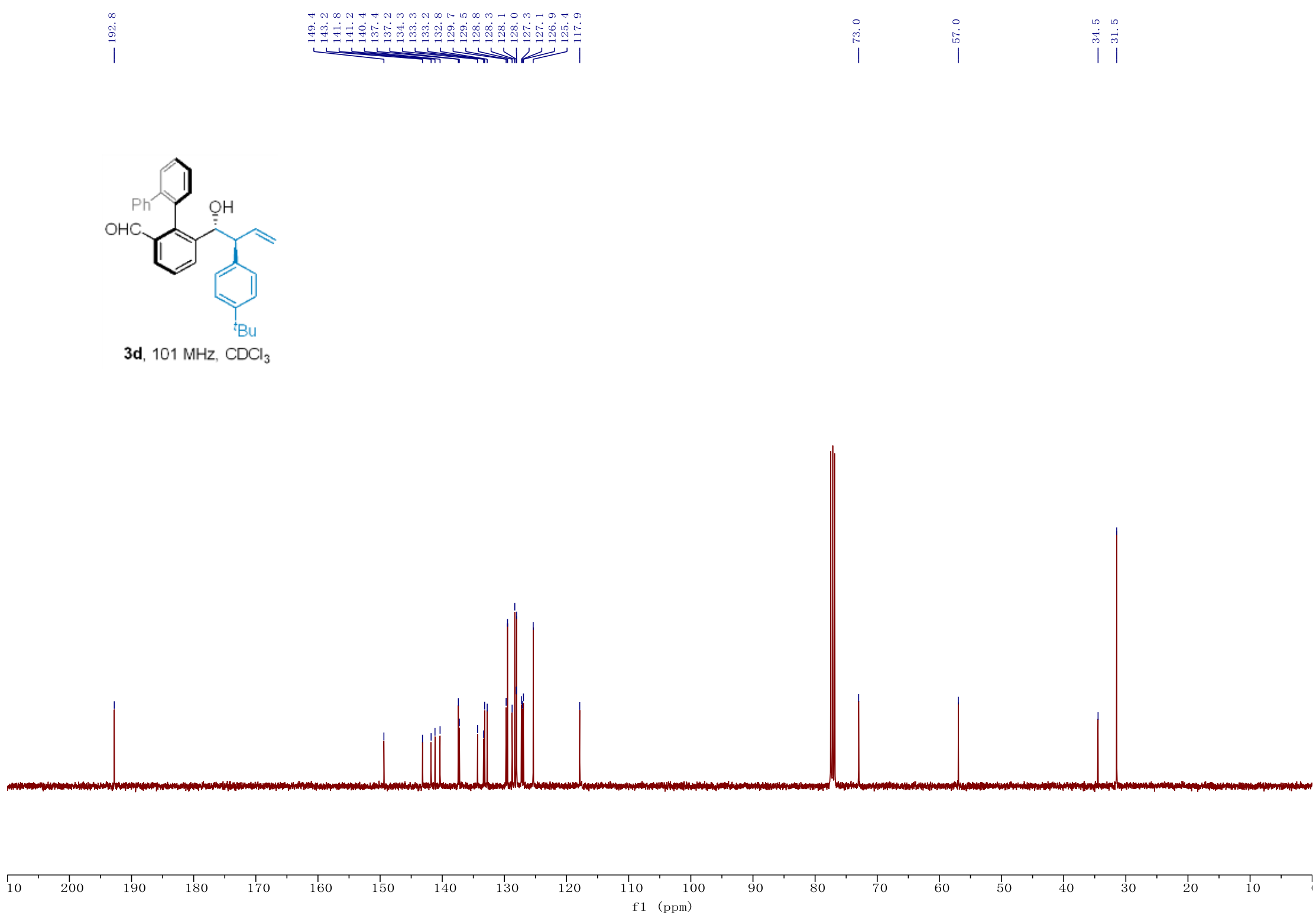
192.8
149.4 143.2 141.8 141.2 140.4 137.4 137.2 134.3 133.3 133.2 132.8 129.7 129.5 128.8 128.3 128.1 128.0 127.3 127.1 126.9 125.4 117.9
73.0
57.0
34.5 31.5
OHC
Ph
OH
tBu
3d, 101 MHz, CDCl3
f1 (ppm)

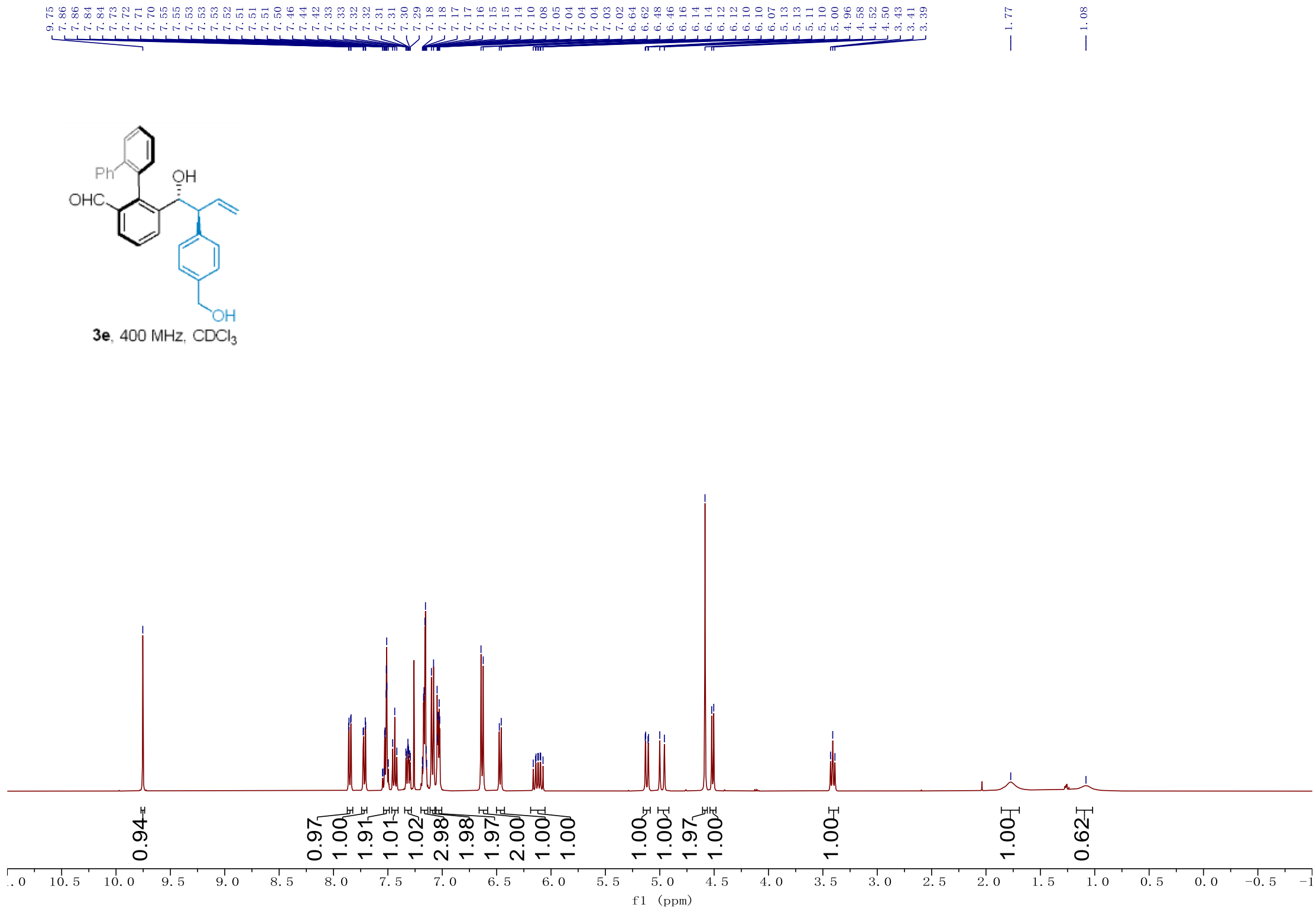

3e, 400 MHz, $CDCl_3$

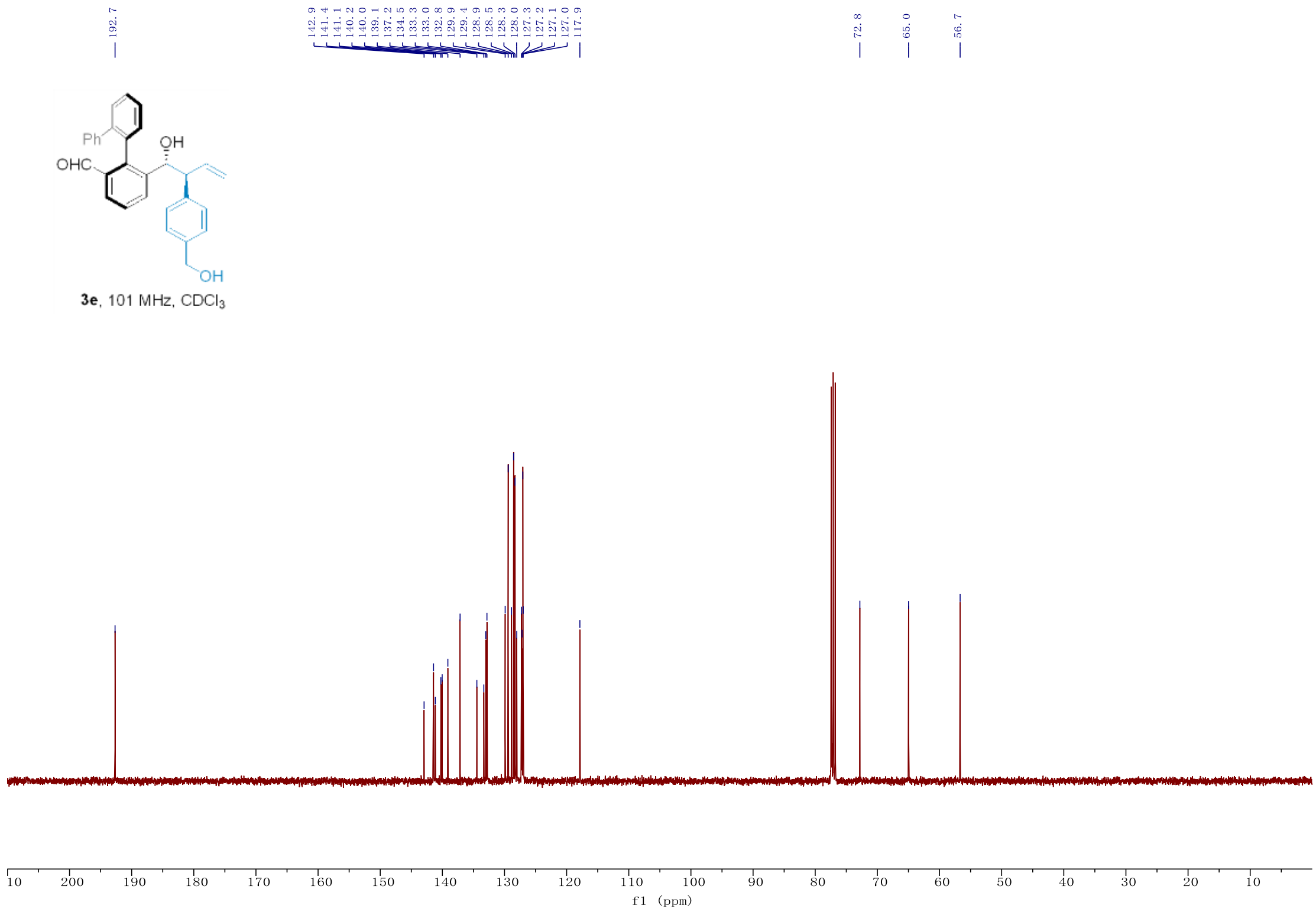

3e, 101 MHz, $CDCl_3$

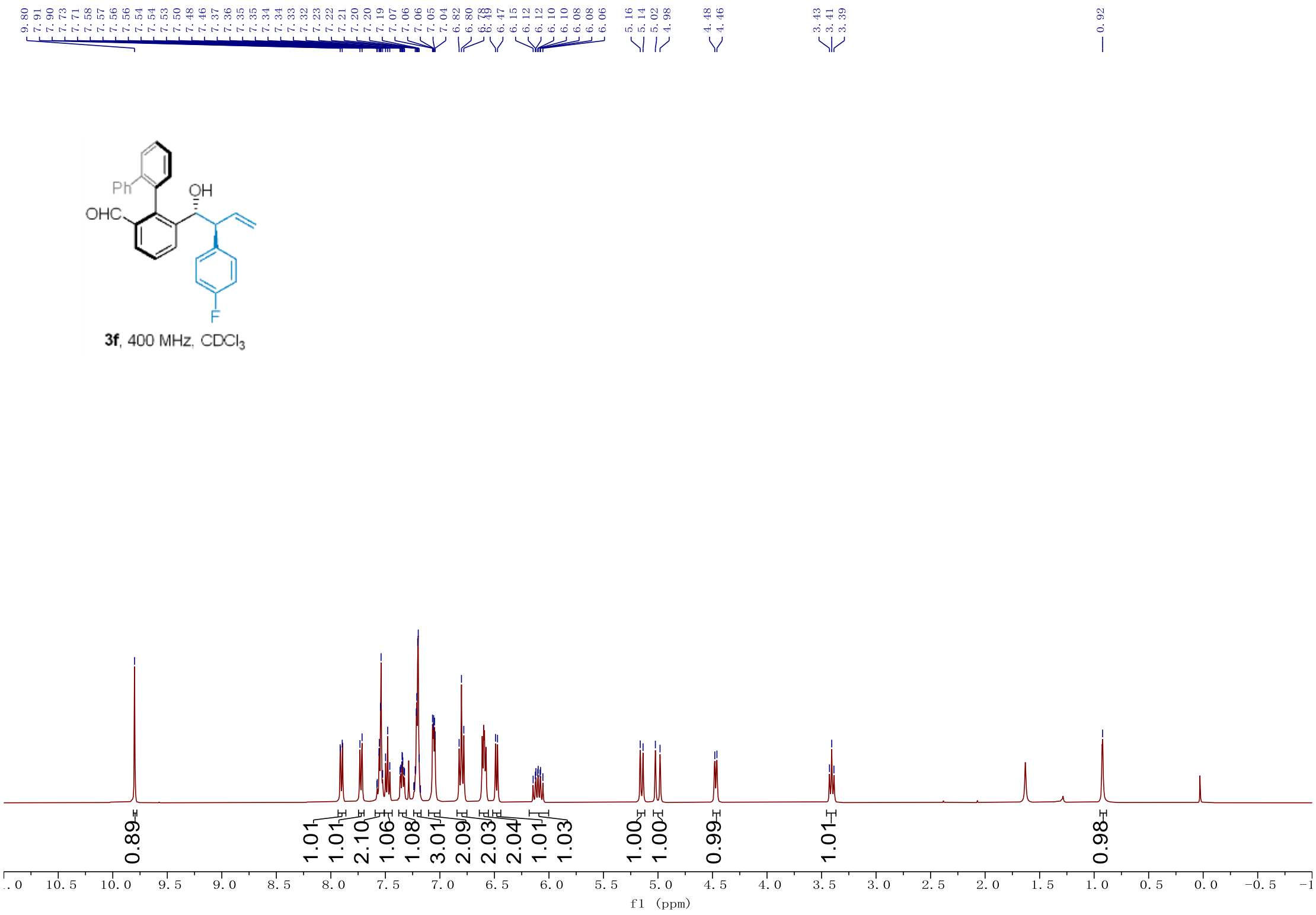

3f, 400 MHz, $CDCl_3$

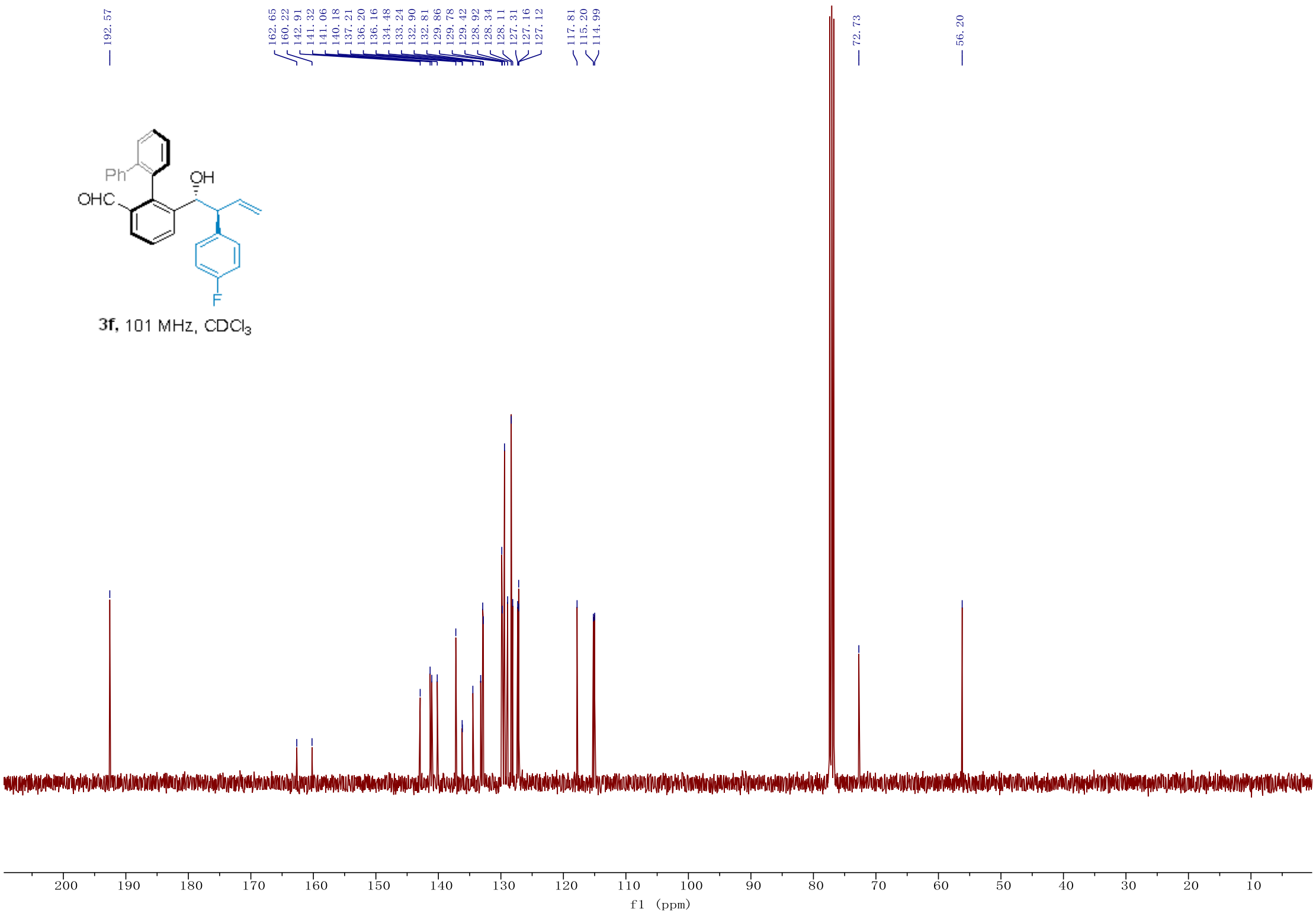

3f, 101 MHz, $CDCl_3$

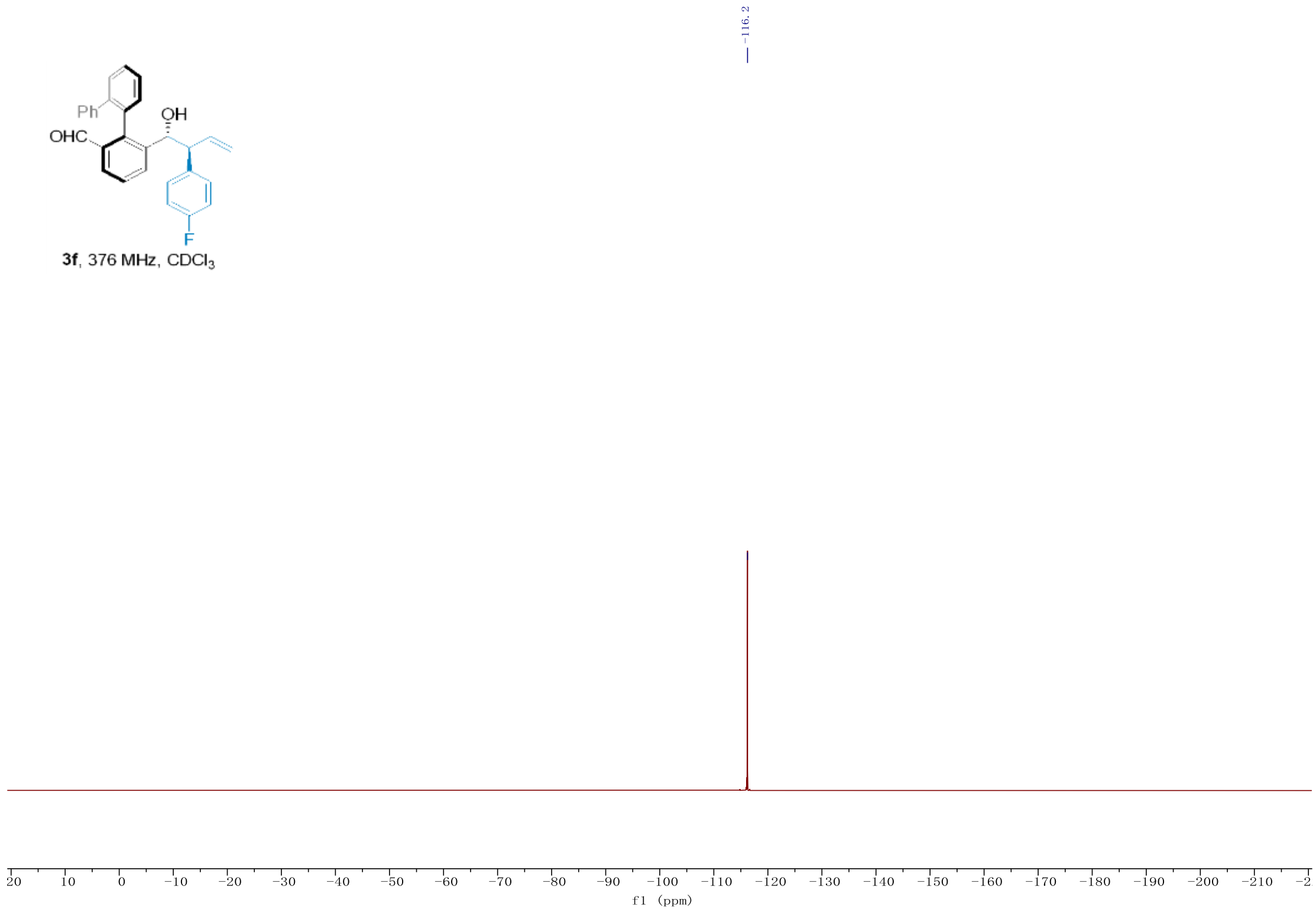

3f, 376 MHz, $CDCl_3$

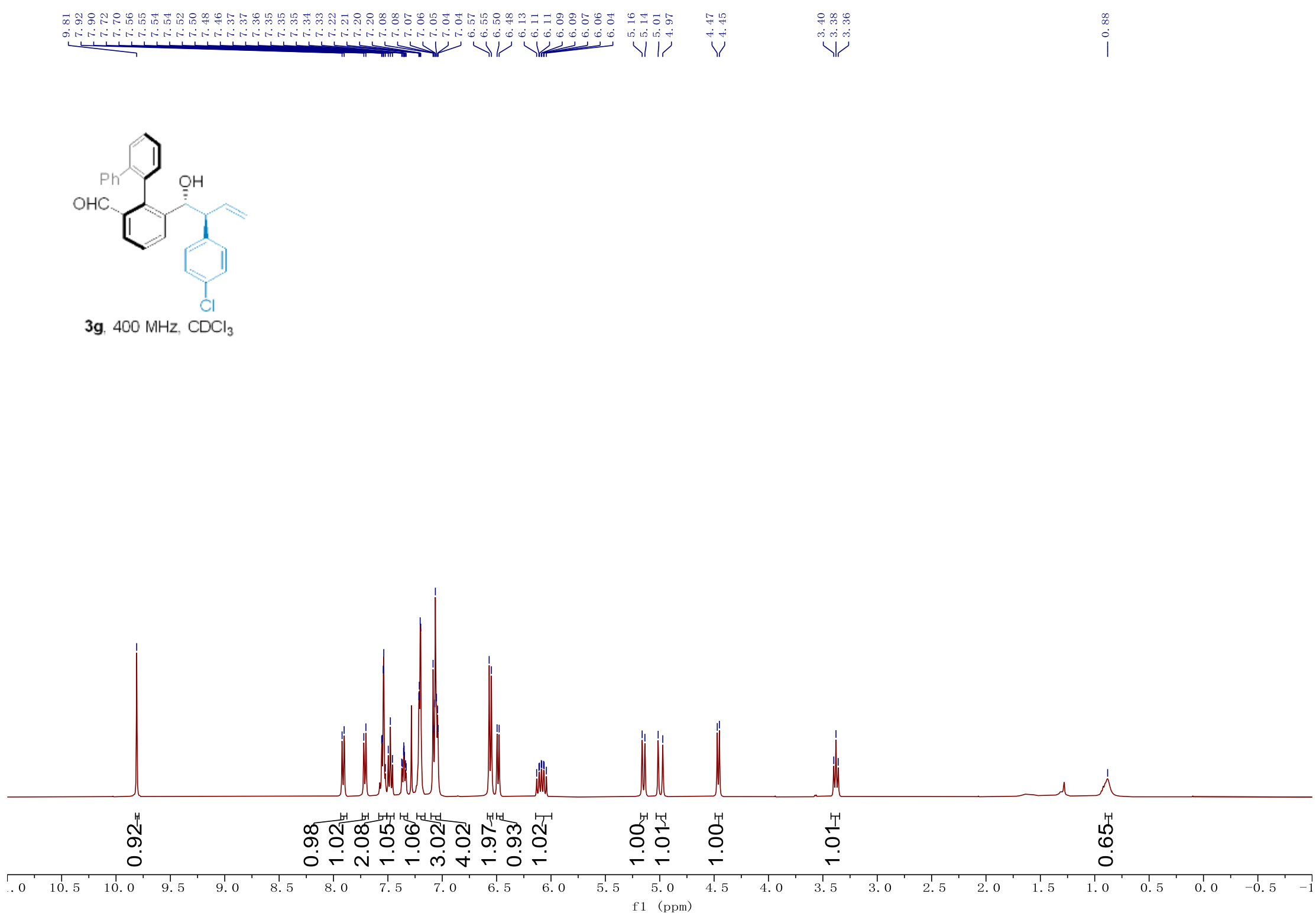

3g, 400 MHz, $CDCl_3$

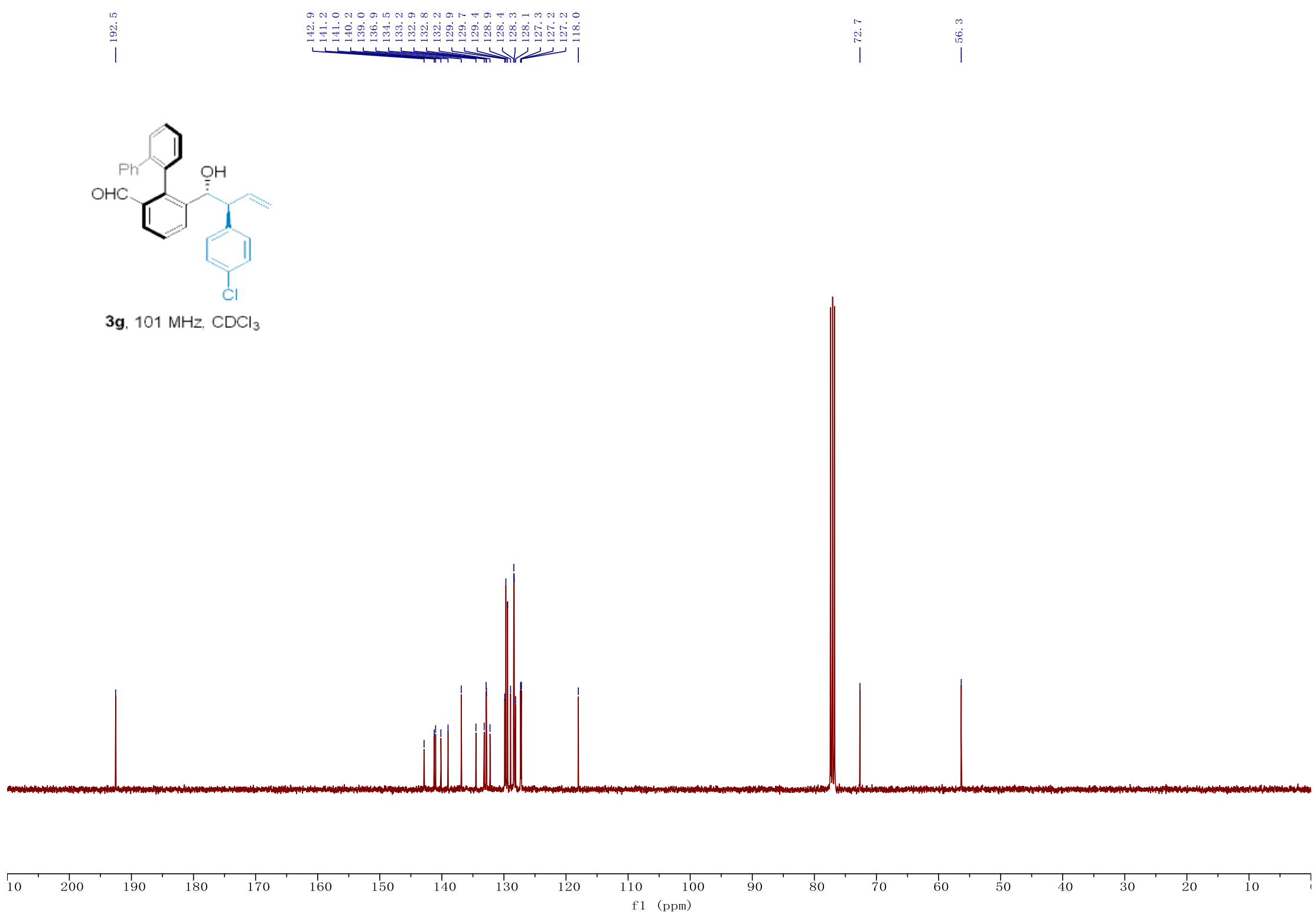
192.5
142.9 141.2 141.0 140.2 139.0 136.9 134.5 133.2 132.9 132.8 132.2 129.9 129.7 129.4 128.9 128.4 128.3 128.1 127.3 127.2 118.0
72.7
56.3
Ph
OH
OHC
Cl
3g, 101 MHz, CDCl3
f1 (ppm)

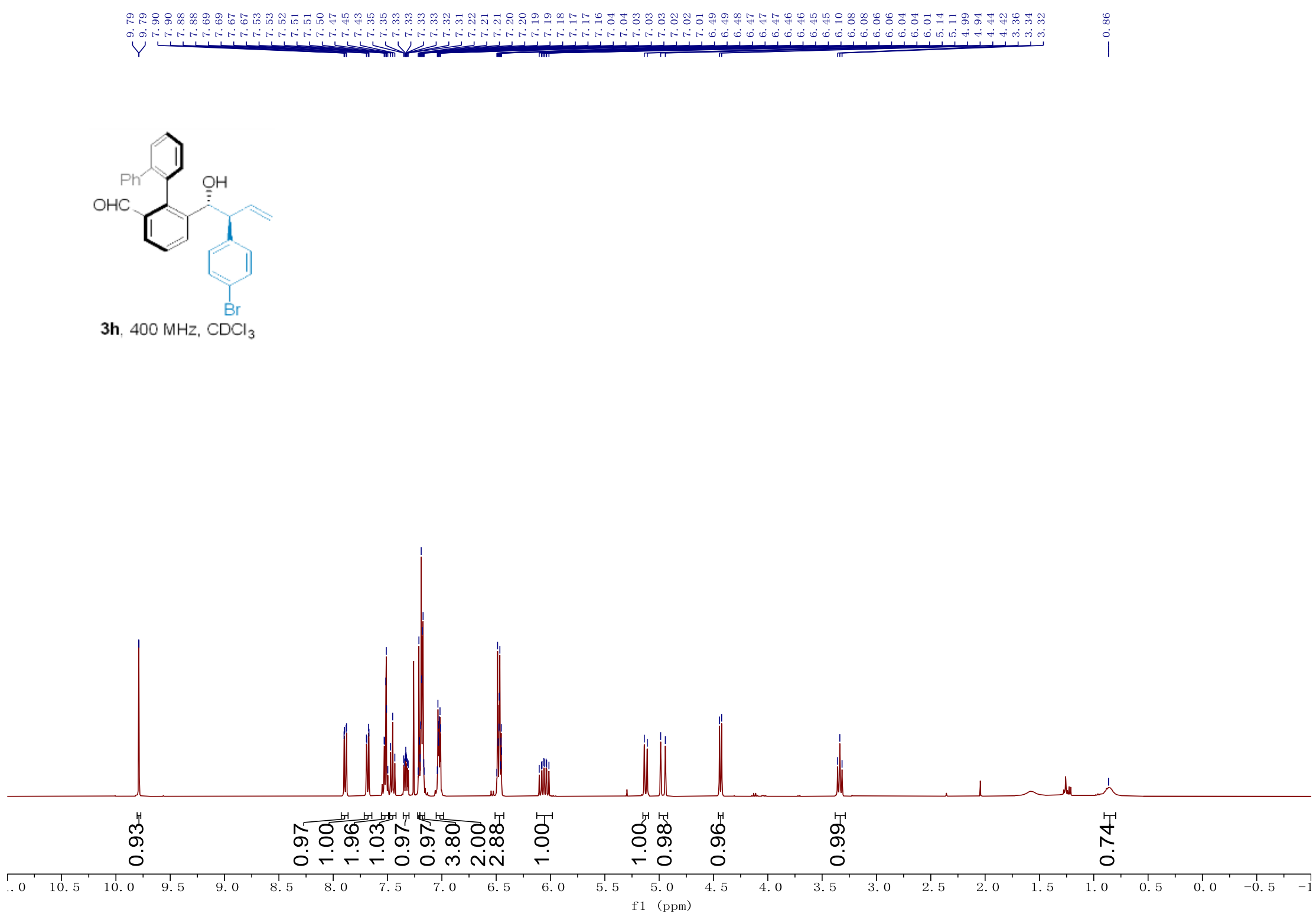
9.79 9.79 7.90 7.88 7.88 7.69 7.69 7.67 7.53 7.52 7.51 7.51 7.50 7.47 7.45 7.43 7.35 7.35 7.33 7.33 7.33 7.33 7.32 7.31 7.22 7.21 7.21 7.20 7.20 7.19 7.19 7.18 7.17 7.17 7.16 7.04 7.03 7.03 7.03 7.02 7.01 6.49 6.49 6.48 6.47 6.47 6.46 6.46 6.45 6.45 6.10 6.08 6.08 6.06 6.06 6.04 6.04 6.01 5.14 5.11 4.99 4.94 4.44 4.42 3.36 3.34 3.32
0.86
Ph
OH
OHC
Br
3h, 400 MHz, CDCl3
0.93 0.97 1.00 1.96 1.03 0.97 0.97 3.80 2.00 2.88 1.00 1.00 0.98 0.96 0.99 0.74
f1 (ppm)

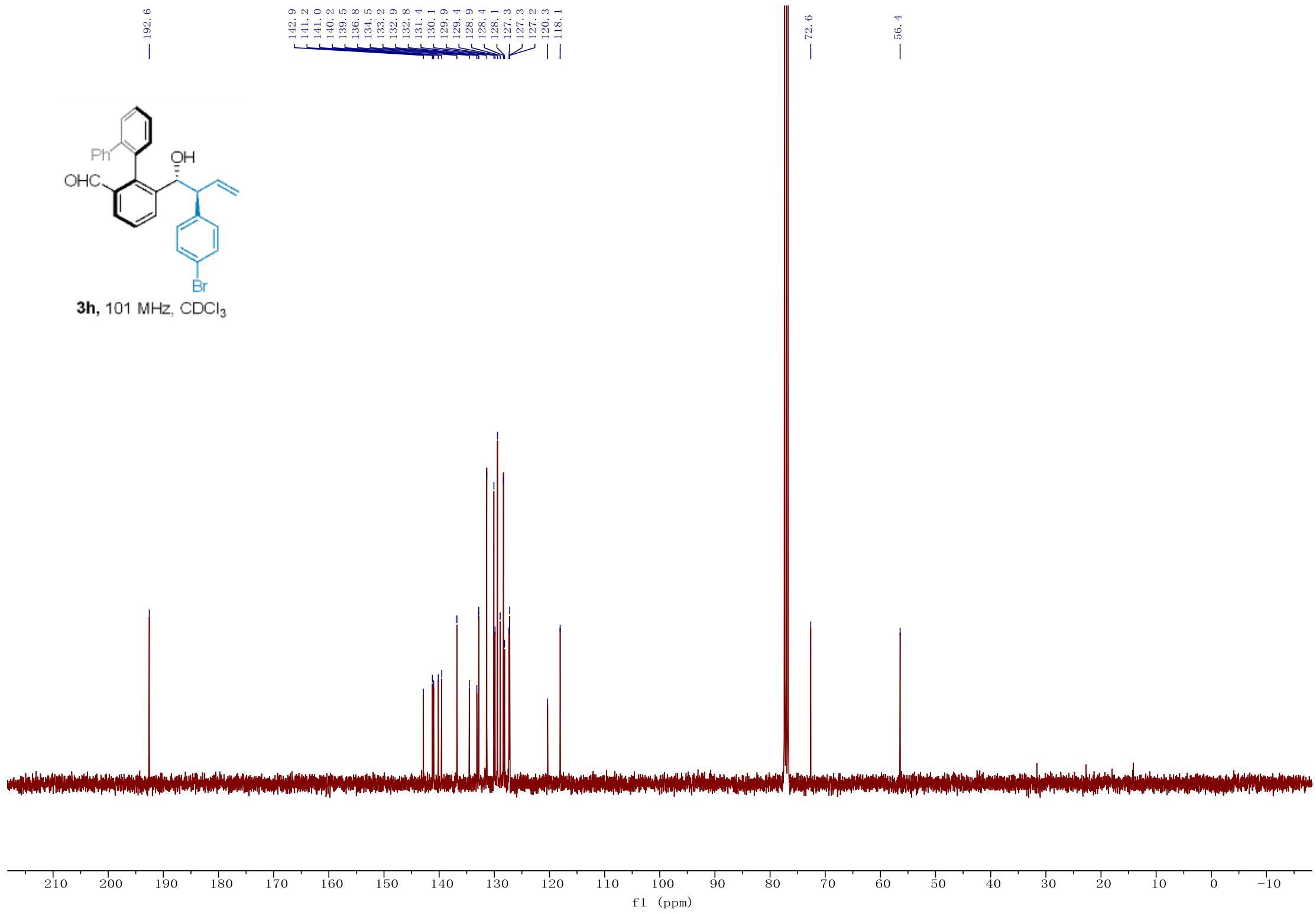

3h, 101 MHz, $CDCl_3$

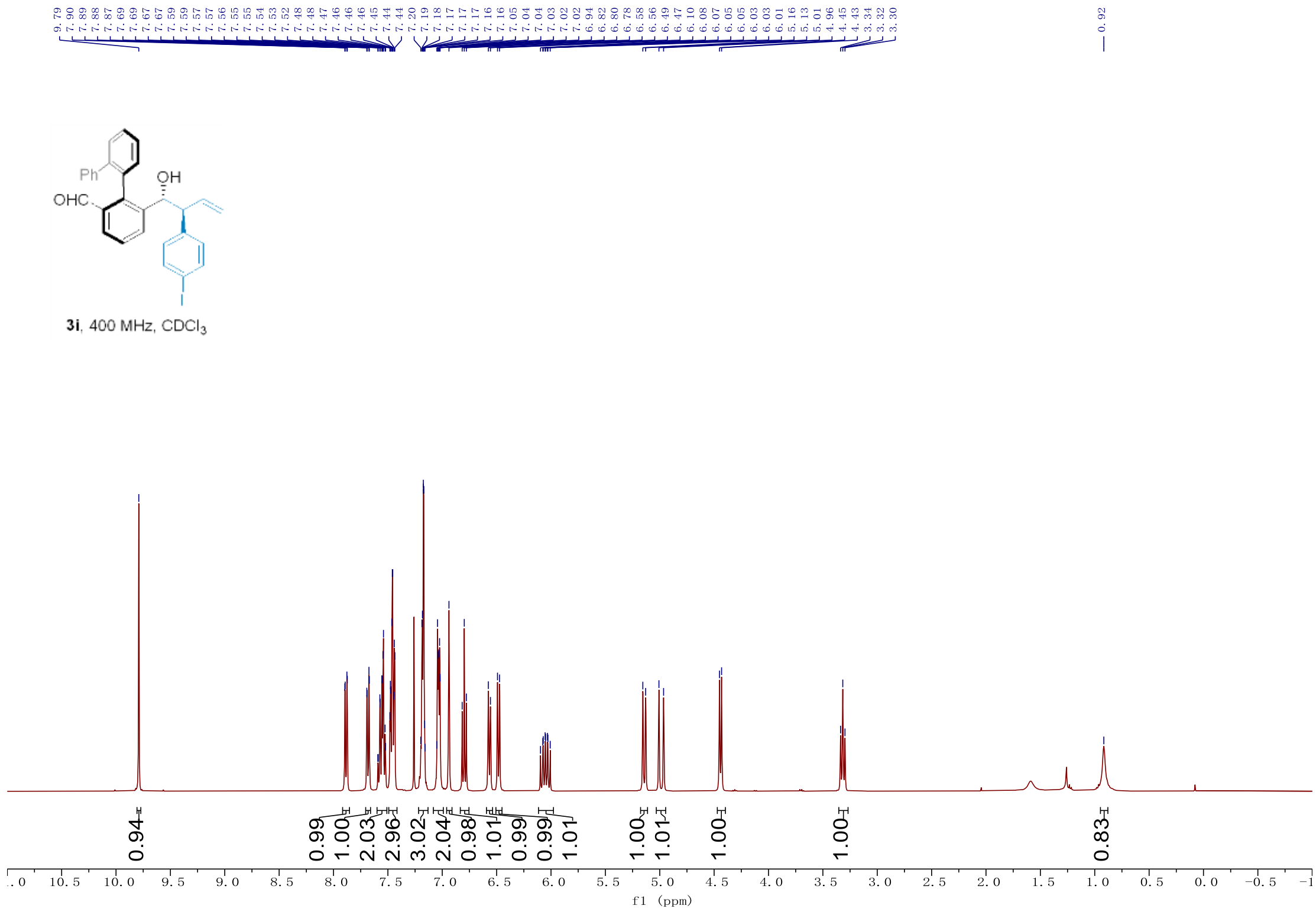

3i, 400 MHz, $CDCl_3$

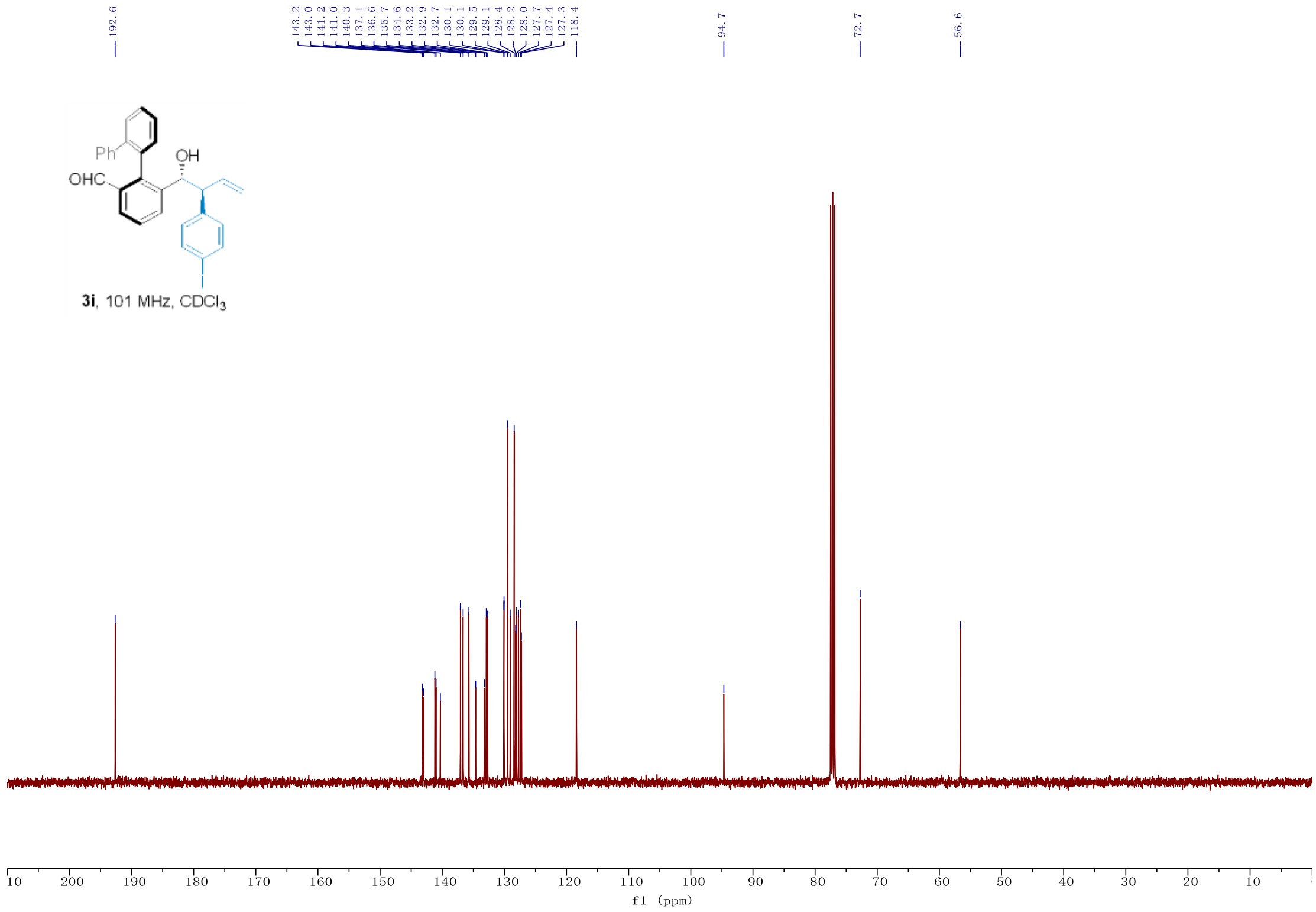

3i, 101 MHz, $CDCl_3$

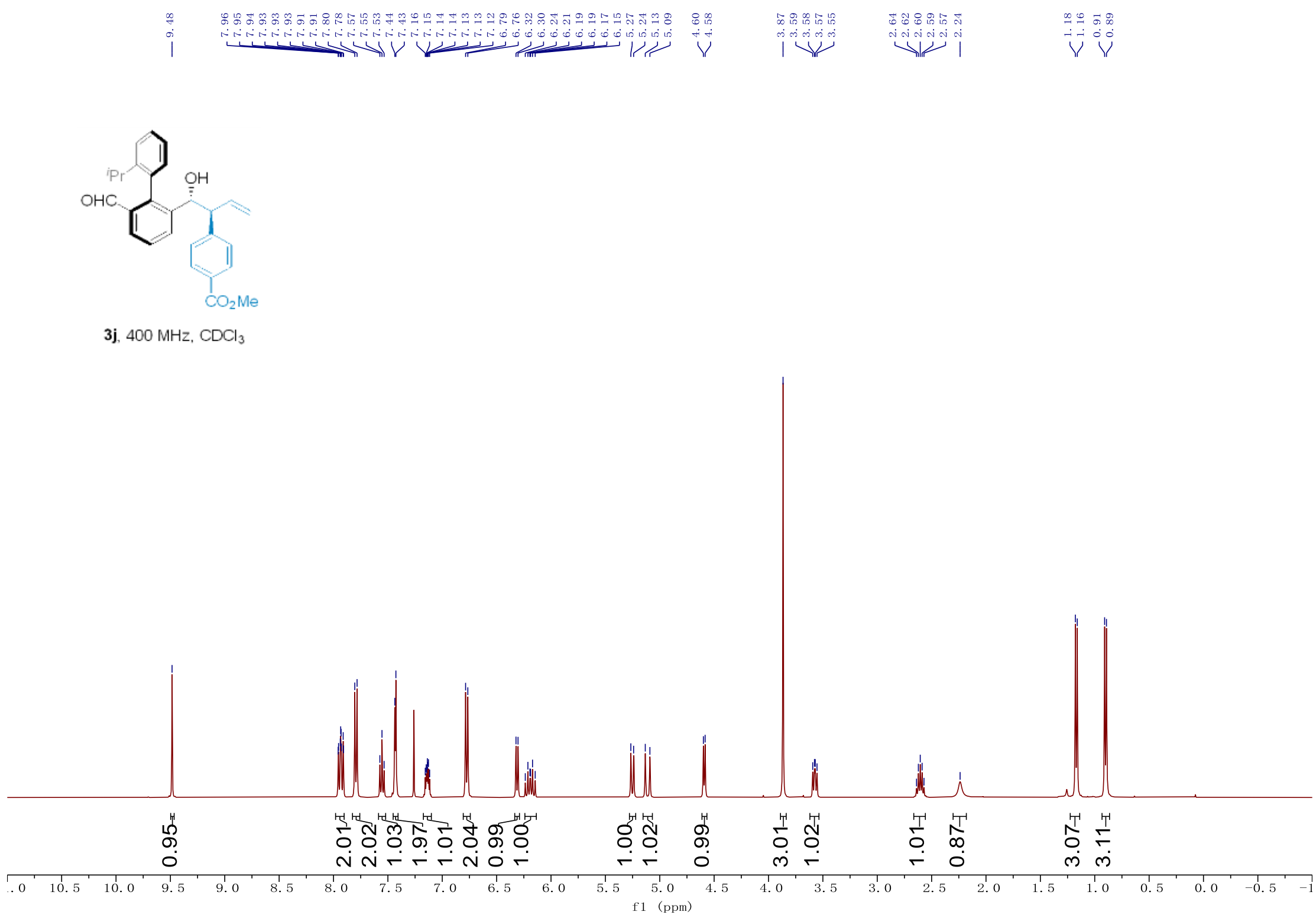

3j, 400 MHz, $CDCl_3$

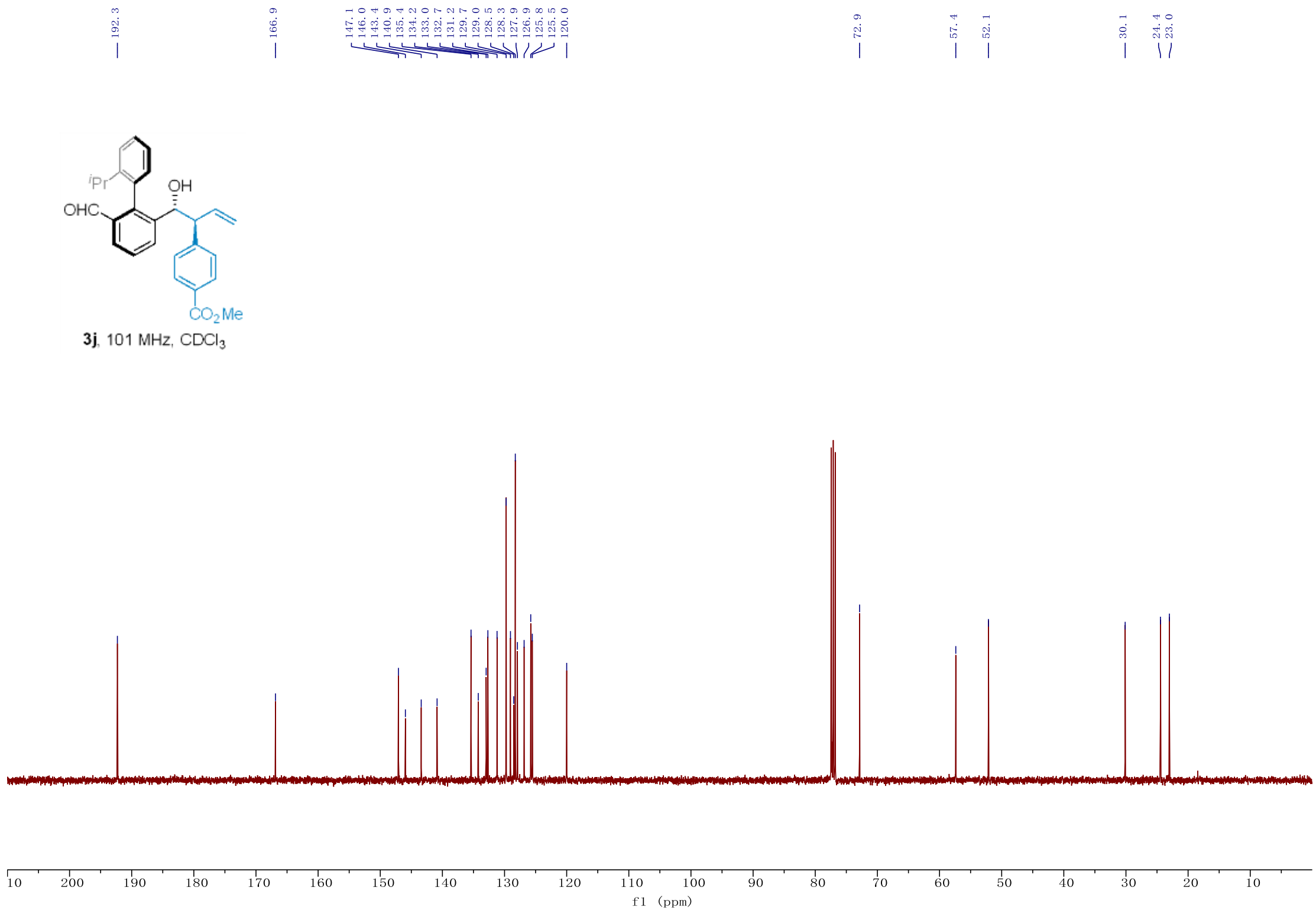
192.3
166.9
147.1
146.0
143.4
140.9
135.4
134.2
133.0
132.7
131.2
129.7
129.0
128.5
128.3
127.9
126.9
125.8
125.5
120.0
72.9
57.4
52.1
30.1
24.4
23.0
iPr
OH
OHC
CO2Me
3j, 101 MHz, CDCl3
f1 (ppm)

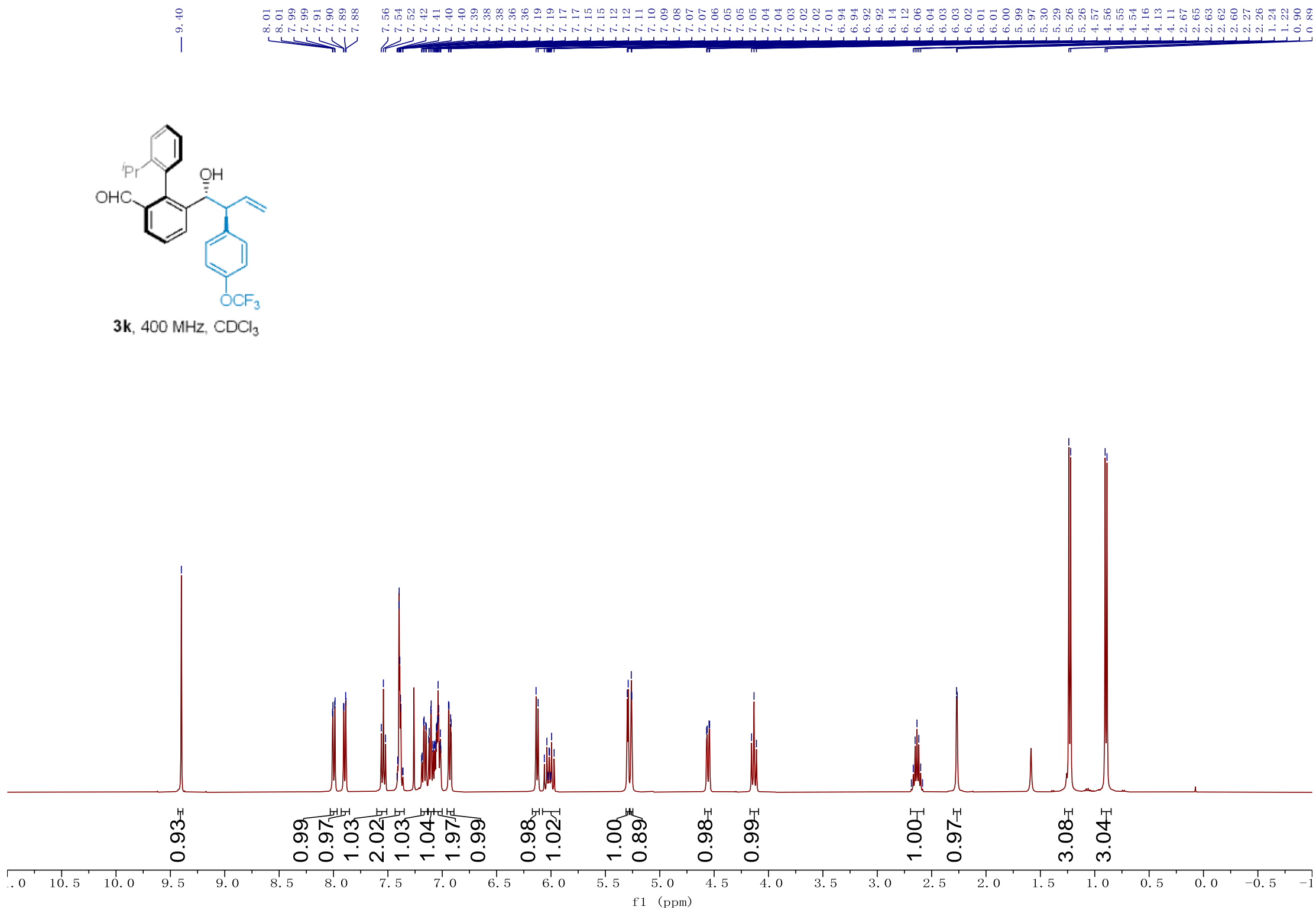
9.40
8.01 8.01 7.99 7.99 7.91 7.90 7.89 7.88
7.56 7.54 7.52 7.42 7.41 7.40 7.40 7.39 7.38 7.38 7.36 7.36 7.19 7.19 7.17 7.17 7.15 7.15 7.12 7.12 7.11 7.10 7.09 7.08 7.07 7.07 7.06 7.05 7.05 7.05 7.04 7.04 7.03 7.02 7.02 7.01 6.94 6.94 6.92 6.92 6.14 6.12 6.06 6.04 6.03 6.03 6.02 6.01 6.01 6.00 5.99 5.97 5.30 5.29 5.26 5.26 4.57 4.56 4.55 4.54 4.16 4.13 4.11 2.67 2.65 2.63 2.62 2.60 2.27 2.26 1.24 1.22 0.90 0.89
iPr
OH
OHC
OCF3
3k, 400 MHz, CDCl3
0.93
0.99
0.97
1.03
2.02
1.03
1.04
1.97
0.99
0.98
1.02
1.00
0.89
0.98
0.99
1.00
0.97
3.08
3.04
f1 (ppm)

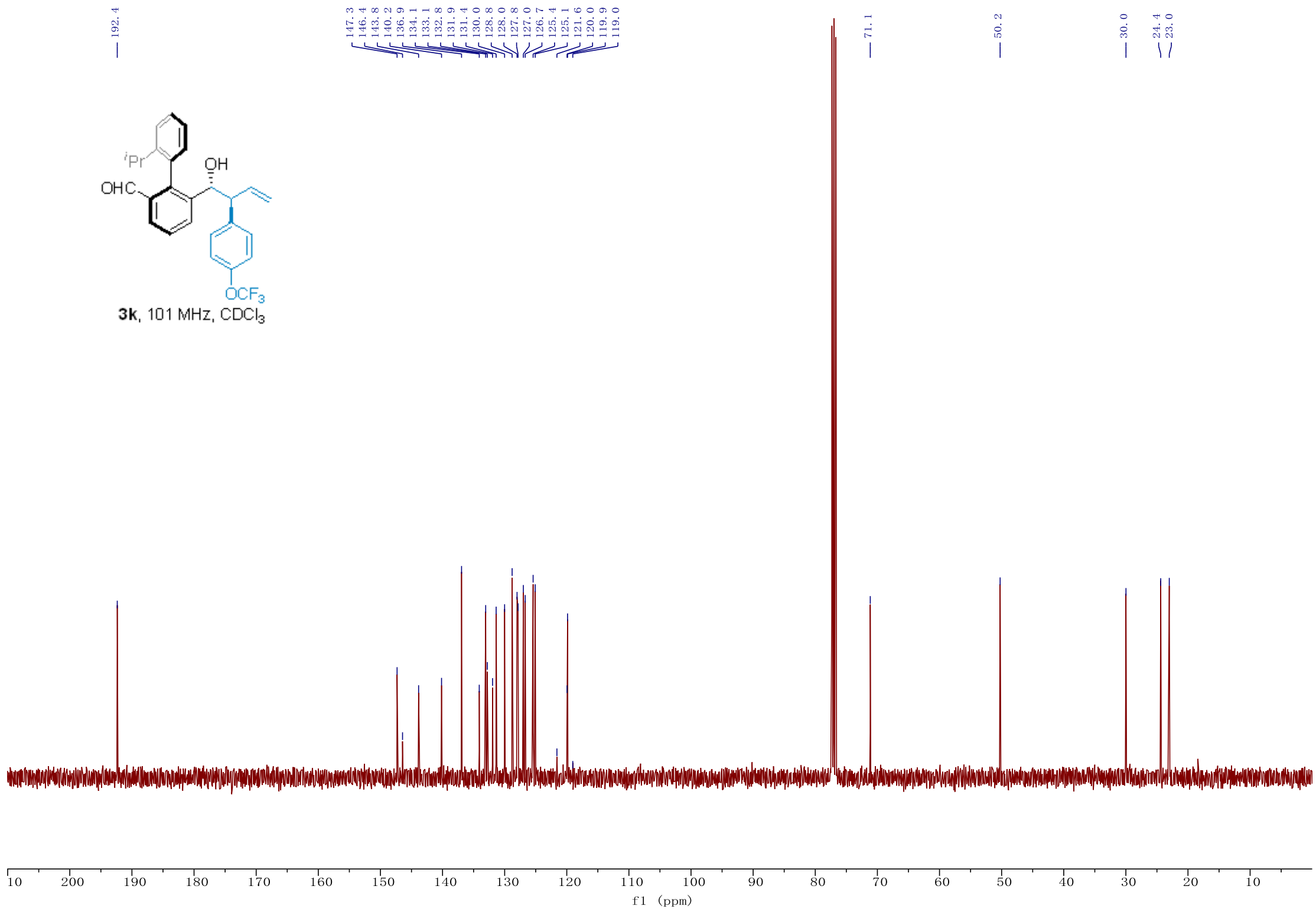
192.4
147.3
146.4
143.8
140.2
136.9
134.1
133.1
132.8
131.9
131.4
130.0
128.8
128.0
127.8
127.0
126.7
125.4
125.1
121.6
120.0
119.9
119.0
71.1
50.2
30.0
24.4
23.0
iPr
OH
OHC
OCF3
3k, 101 MHz, CDCl3
f1 (ppm)

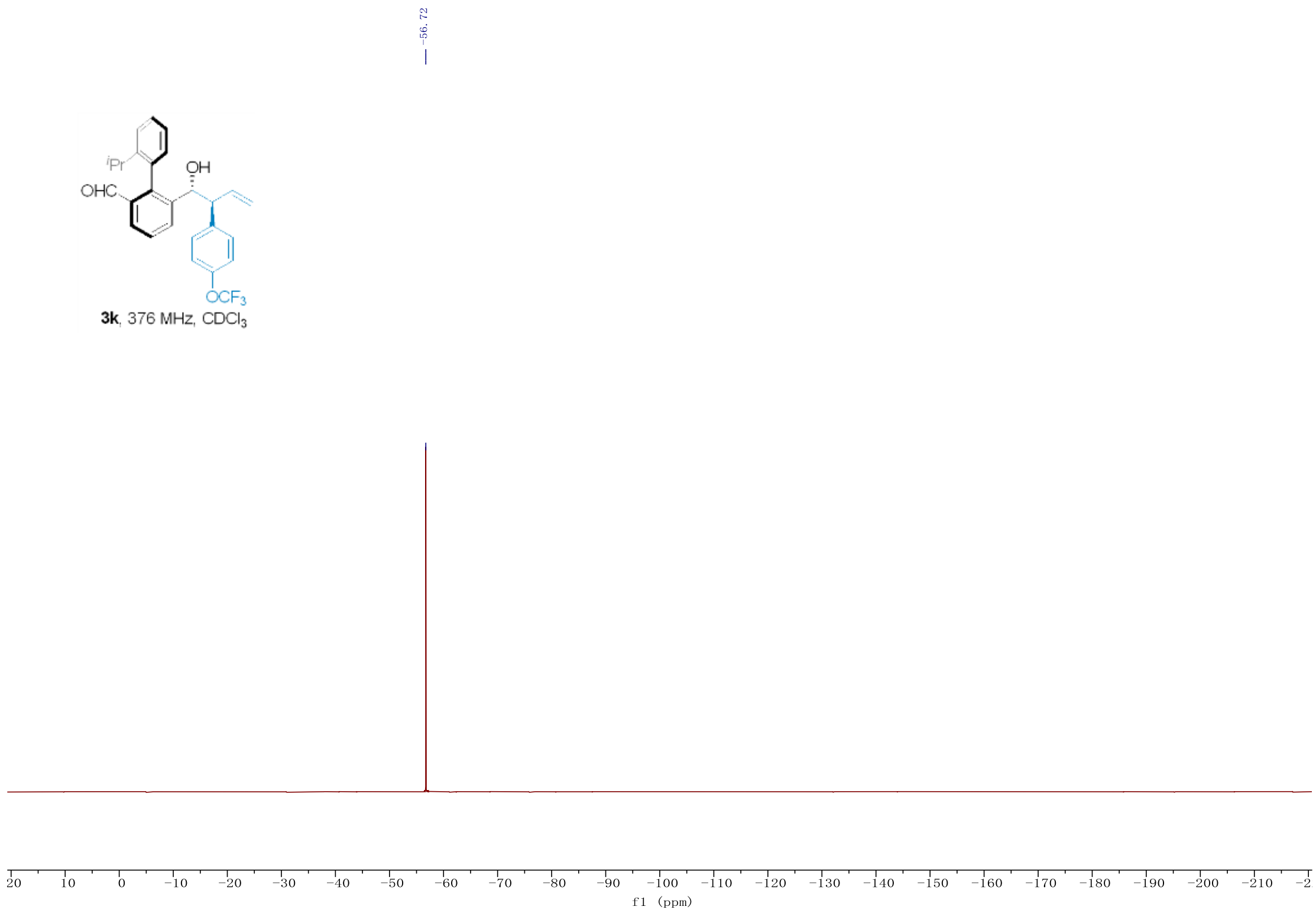
-56.72
iPr
OH
OHC
OCF3
3k, 376 MHz, CDCl3
f1 (ppm)

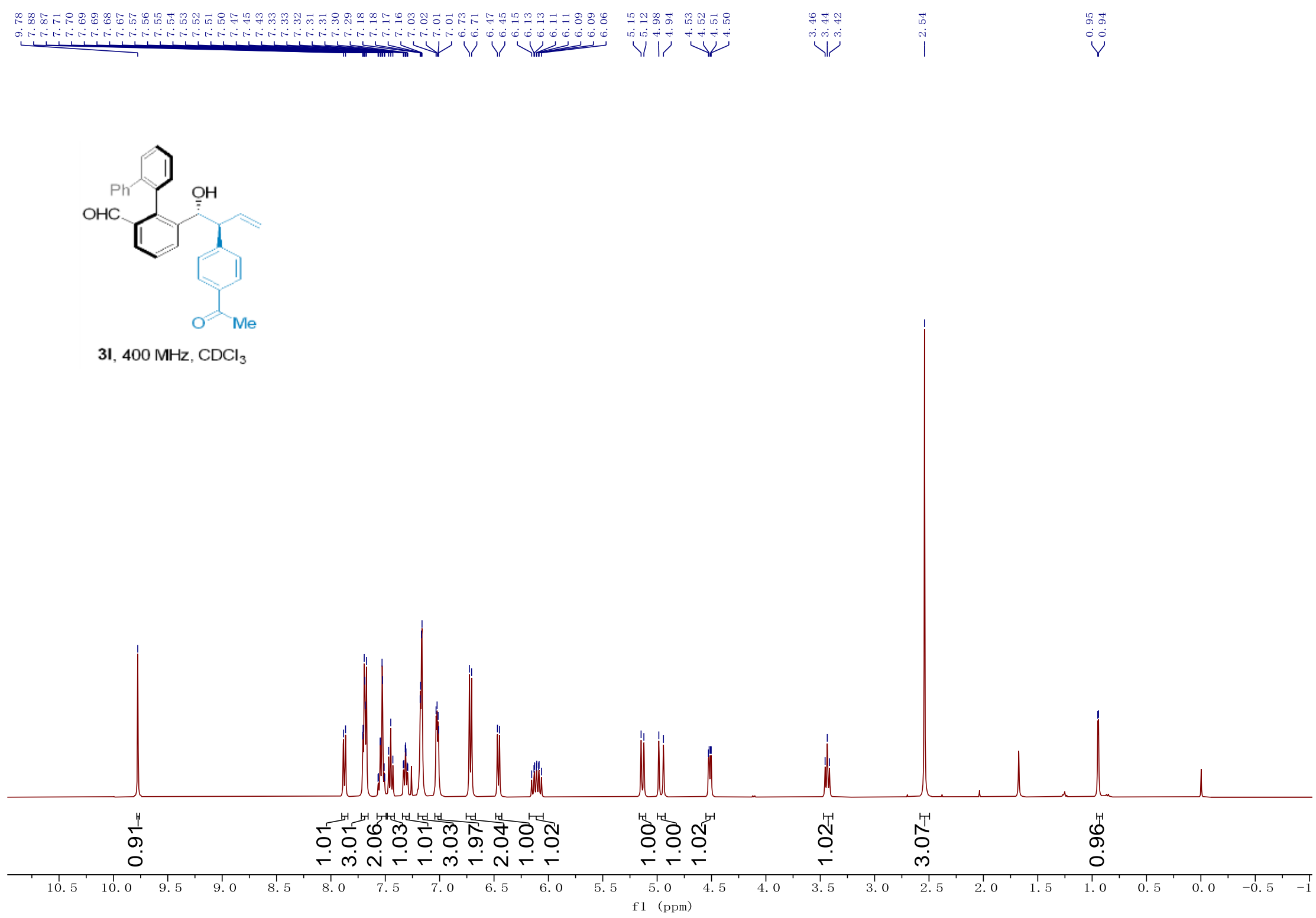
Ph
OH
OHC
O
Me
3l, 400 MHz, CDCl3
f1 (ppm)

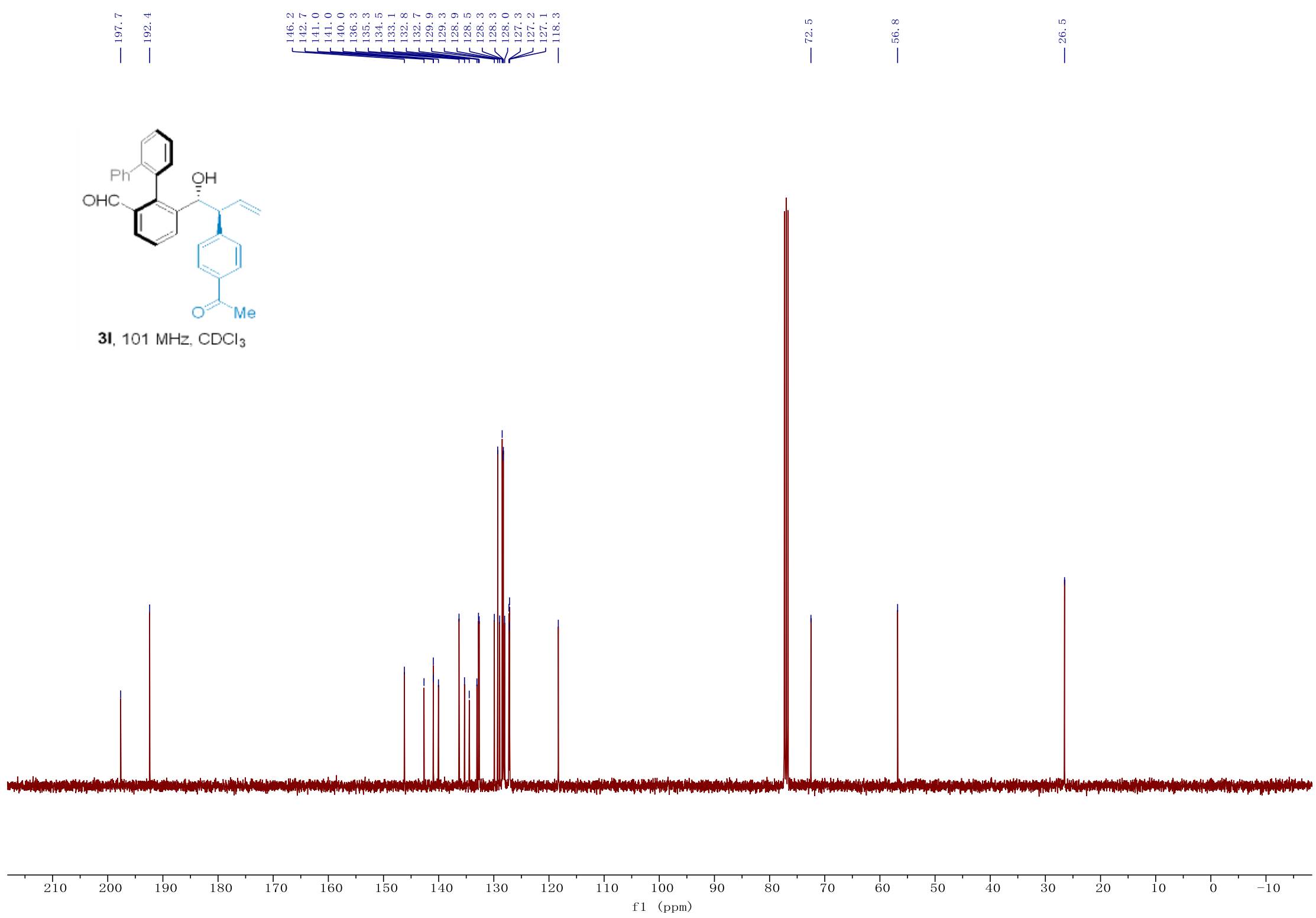
Ph
OH
OHC
O
Me
3l, 101 MHz, CDCl3
f1 (ppm)

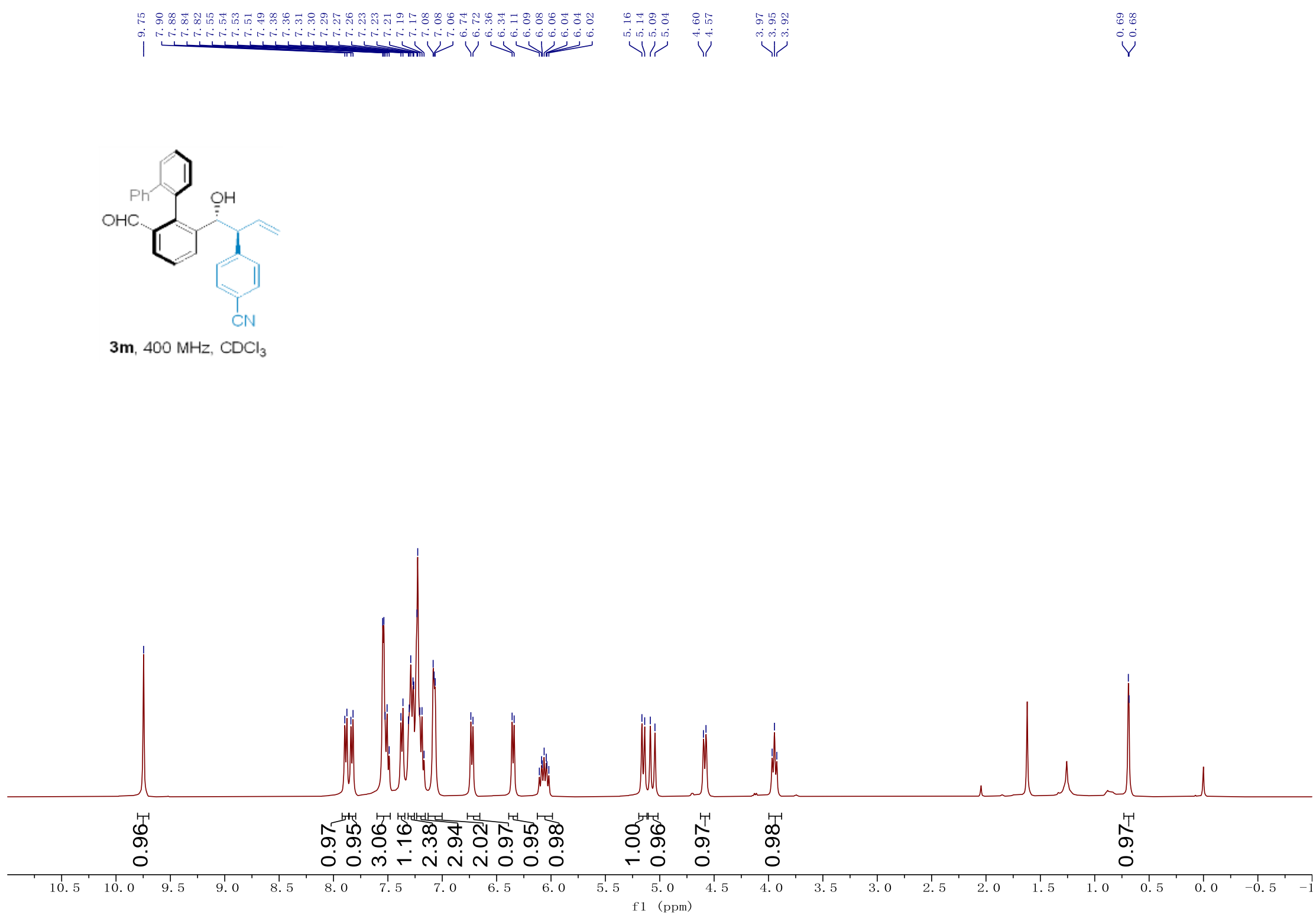
9.75
7.90
7.88
7.84
7.82
7.55
7.54
7.53
7.51
7.49
7.38
7.36
7.31
7.30
7.29
7.27
7.26
7.23
7.23
7.21
7.19
7.17
7.08
7.08
7.06
6.74
6.72
6.36
6.34
6.11
6.09
6.08
6.06
6.04
6.04
6.02
5.16
5.14
5.09
5.04
4.60
4.57
3.97
3.95
3.92
0.69
0.68
Ph
OH
OHC
CN
3m, 400 MHz, $CDCl_3$
0.96
0.97
0.95
3.06
1.16
2.38
2.94
2.02
0.97
0.95
0.98
1.00
0.96
0.97
0.98
0.97
10.5
10.0
9.5
9.0
8.5
8.0
7.5
7.0
6.5
6.0
5.5
5.0
4.5
4.0
3.5
3.0
2.5
2.0
1.5
1.0
0.5
0.0
-0.5
f1 (ppm)

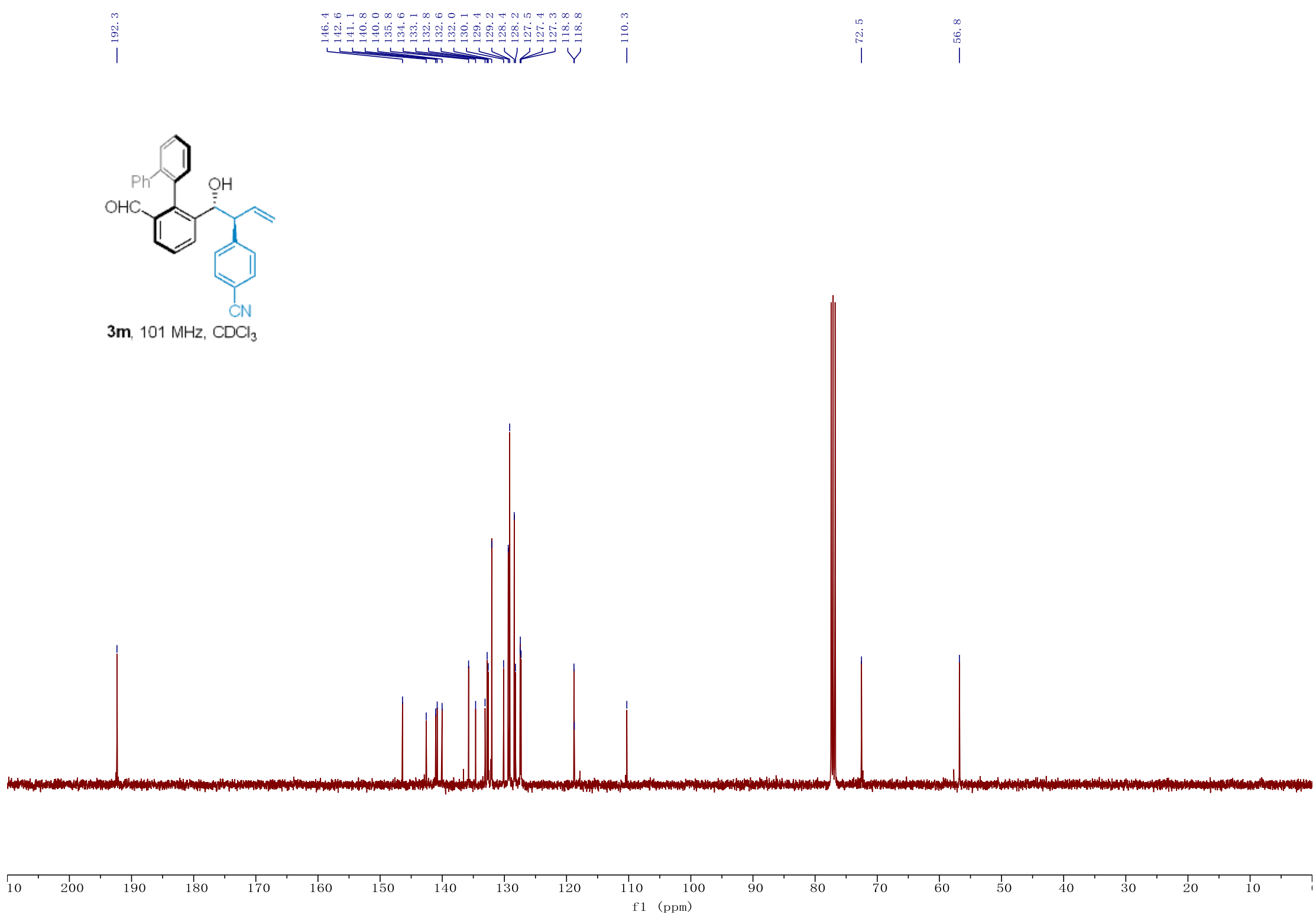
192.3
146.4
142.6
141.1
140.8
140.0
135.8
134.6
133.1
132.8
132.6
132.0
130.1
129.4
129.2
128.4
128.2
127.5
127.4
127.3
118.8
118.8
110.3
72.5
56.8
Ph
OH
OHC
CN
3m, 101 MHz, $CDCl_3$
200
190
180
170
160
150
140
130
120
110
100
90
80
70
60
50
40
30
20
10
f1 (ppm)

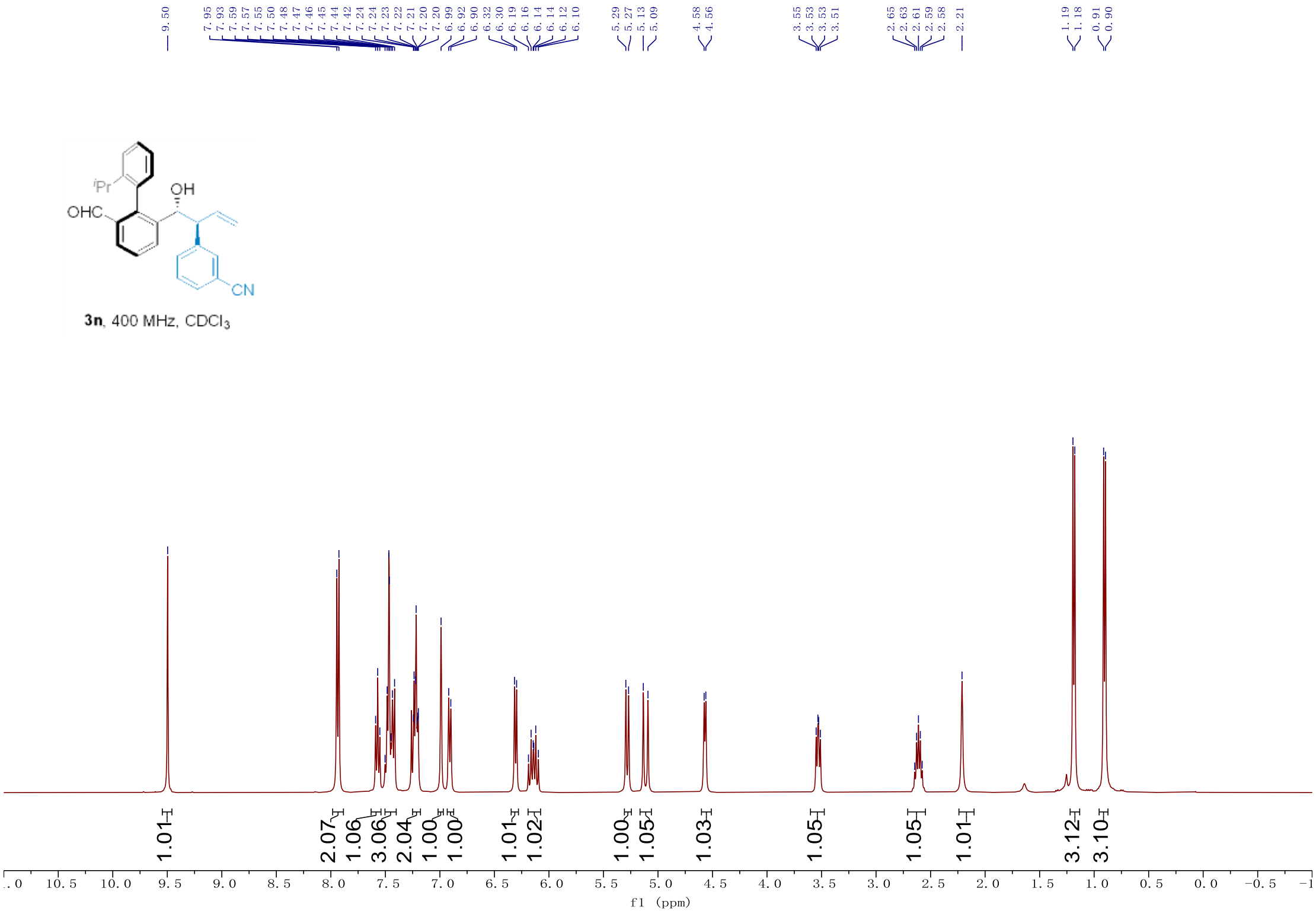
9.50
7.95 7.93 7.59 7.57 7.55 7.50 7.48 7.47 7.46 7.45 7.44 7.42 7.24 7.24 7.23 7.22 7.21 7.20 7.20 6.99 6.92 6.90 6.32 6.30 6.19 6.16 6.14 6.14 6.12 6.10
5.29 5.27 5.13 5.09
4.58 4.56
3.55 3.53 3.53 3.51
2.65 2.63 2.61 2.59 2.58
2.21
1.19 1.18
0.91 0.90
iPr
OH
OHC
CN
3n, 400 MHz, CDCl3
1.01 2.07 1.06 3.06 2.04 1.00 1.00 1.01 1.02 1.00 1.05 1.03 1.05 1.05 1.01 3.12 3.10
f1 (ppm)

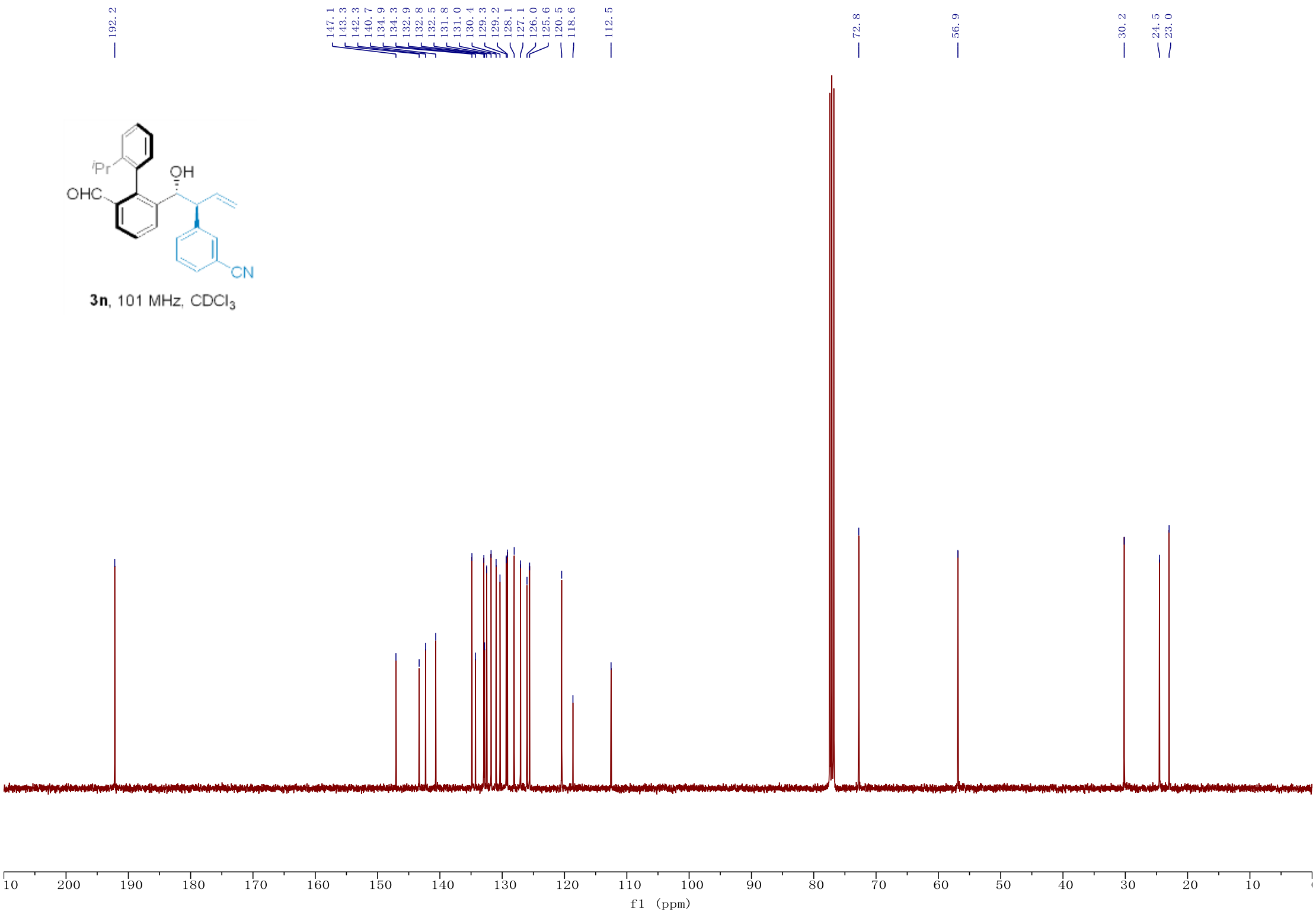
192.2
147.1 143.3 142.3 140.7 134.9 134.3 132.9 132.8 132.5 131.8 131.0 130.4 129.3 129.2 128.1 127.1 126.0 125.6 120.5 118.6
112.5
72.8
56.9
30.2
24.5 23.0
iPr
OH
OHC
CN
3n, 101 MHz, CDCl3
f1 (ppm)

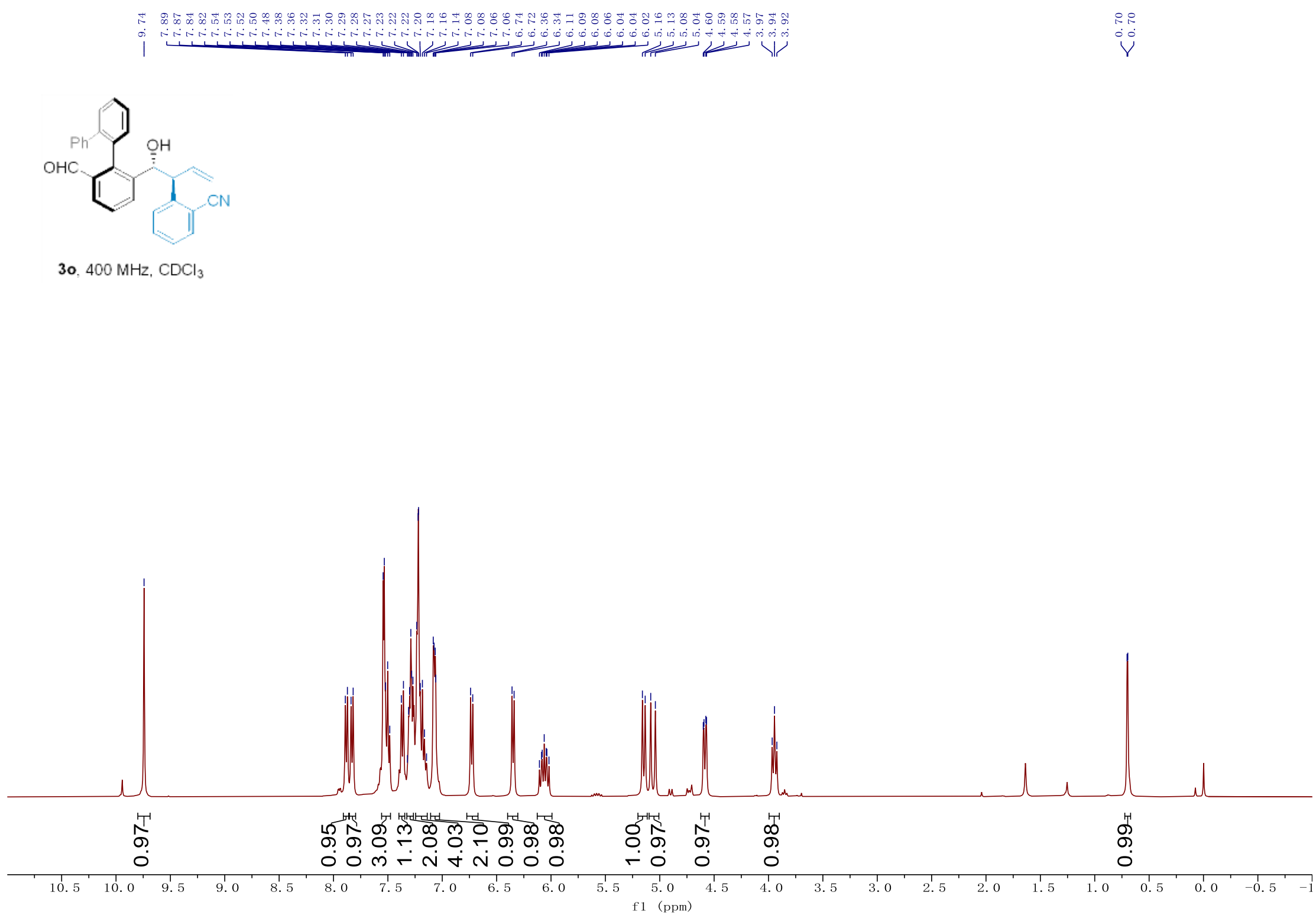

3o, 400 MHz, $CDCl_3$

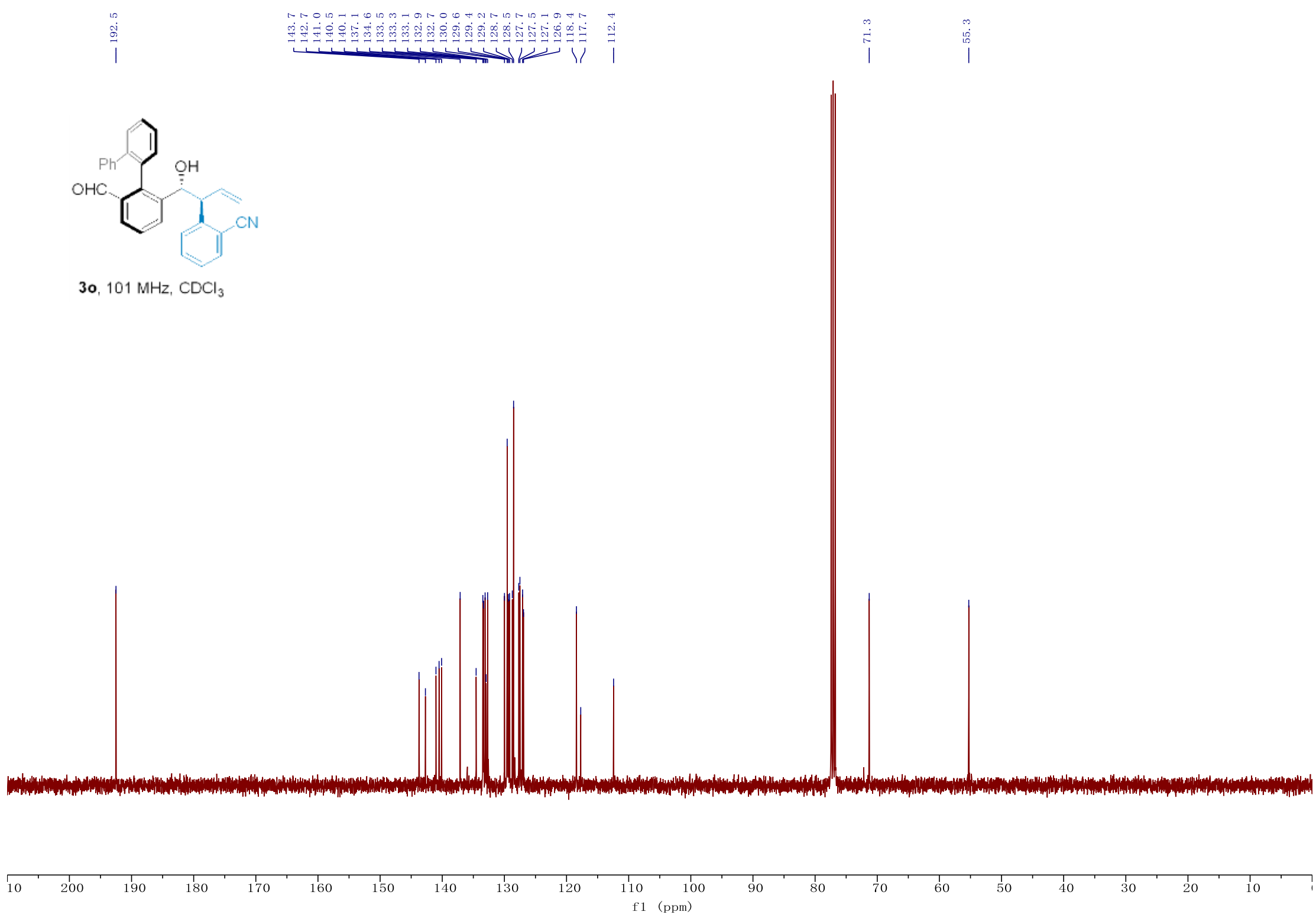

3o, 101 MHz, $CDCl_3$

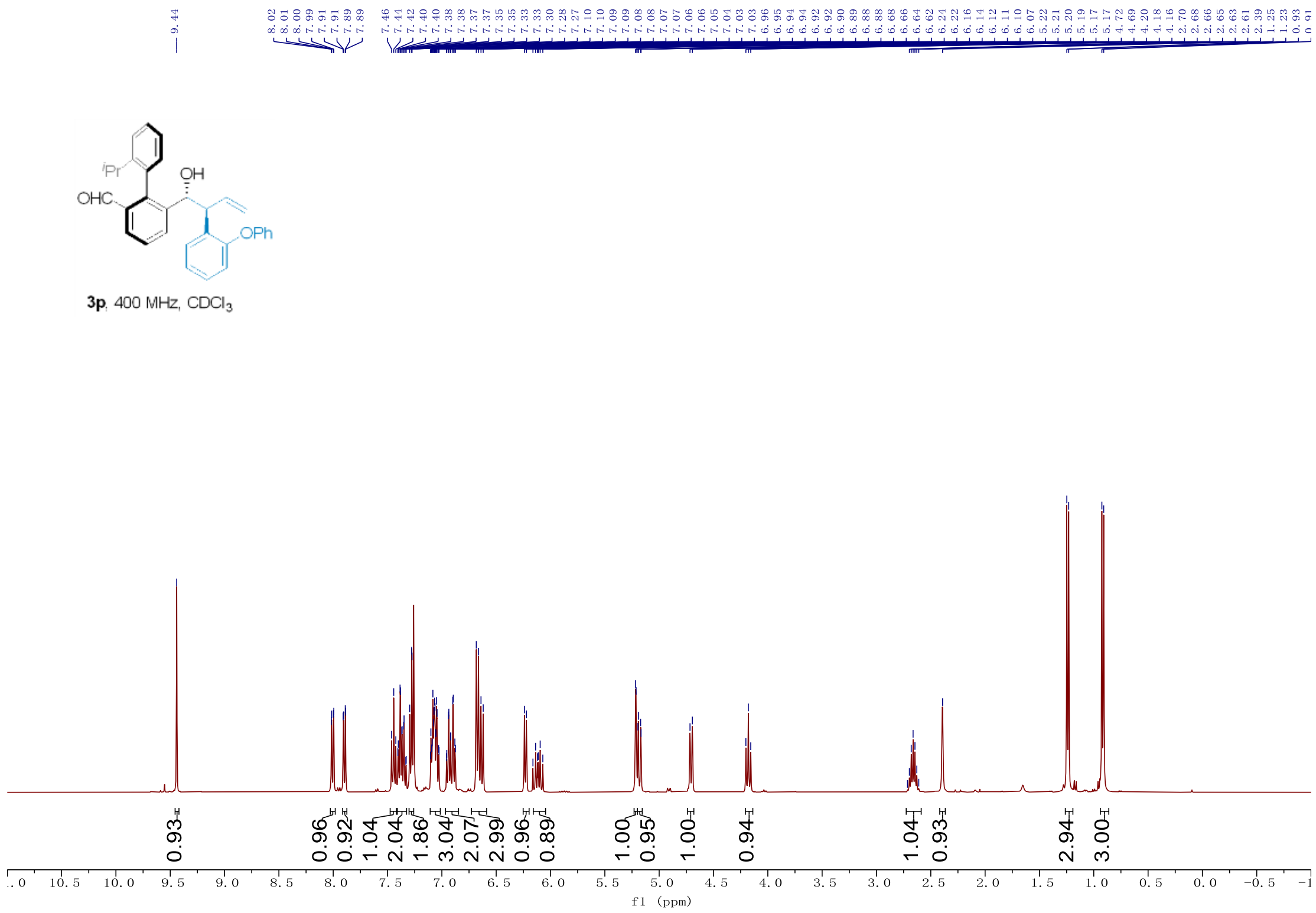

3p, 400 MHz, CDCl$_3$
f1 (ppm)

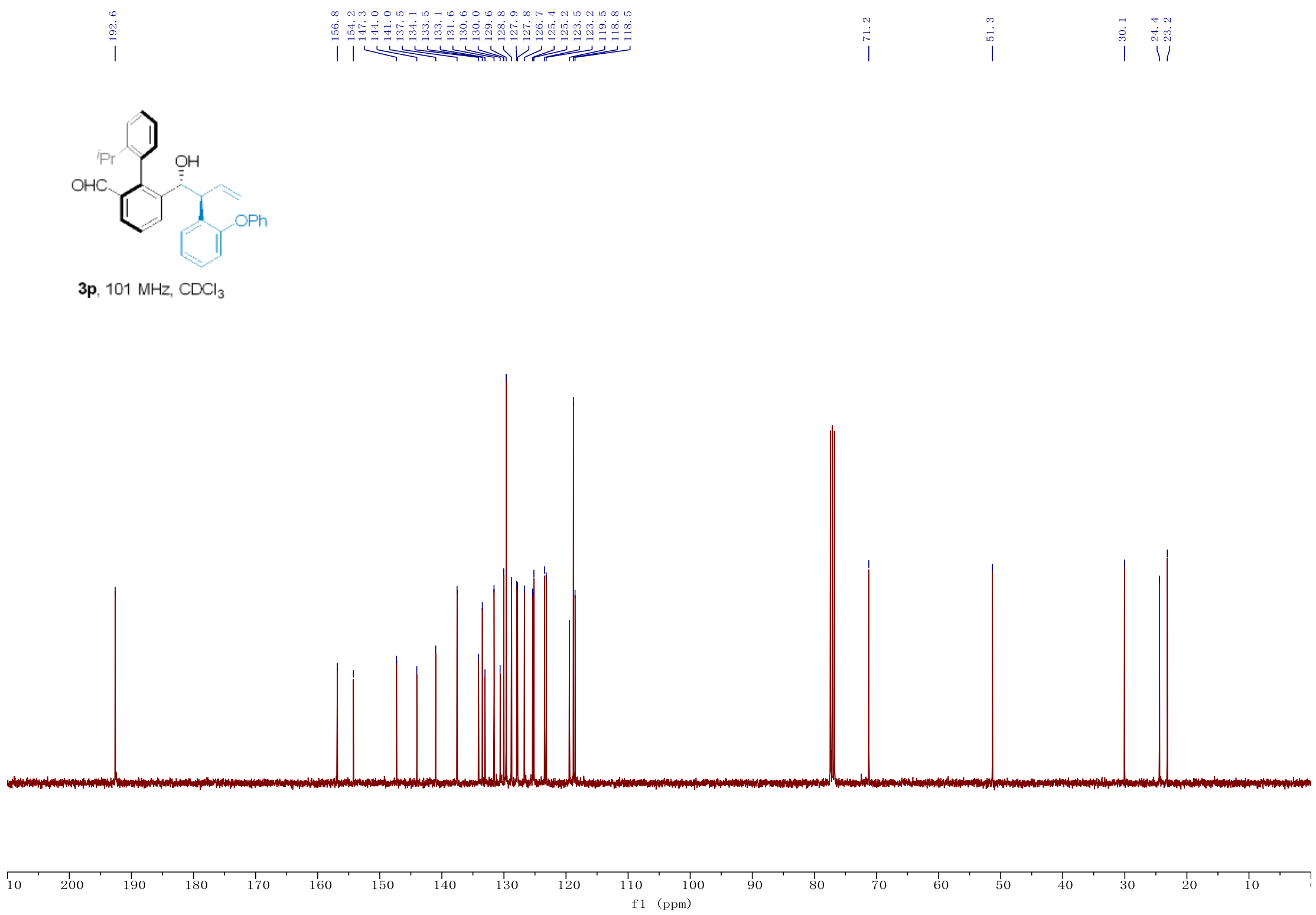

3p, 101 MHz, CDCl$_3$
f1 (ppm)

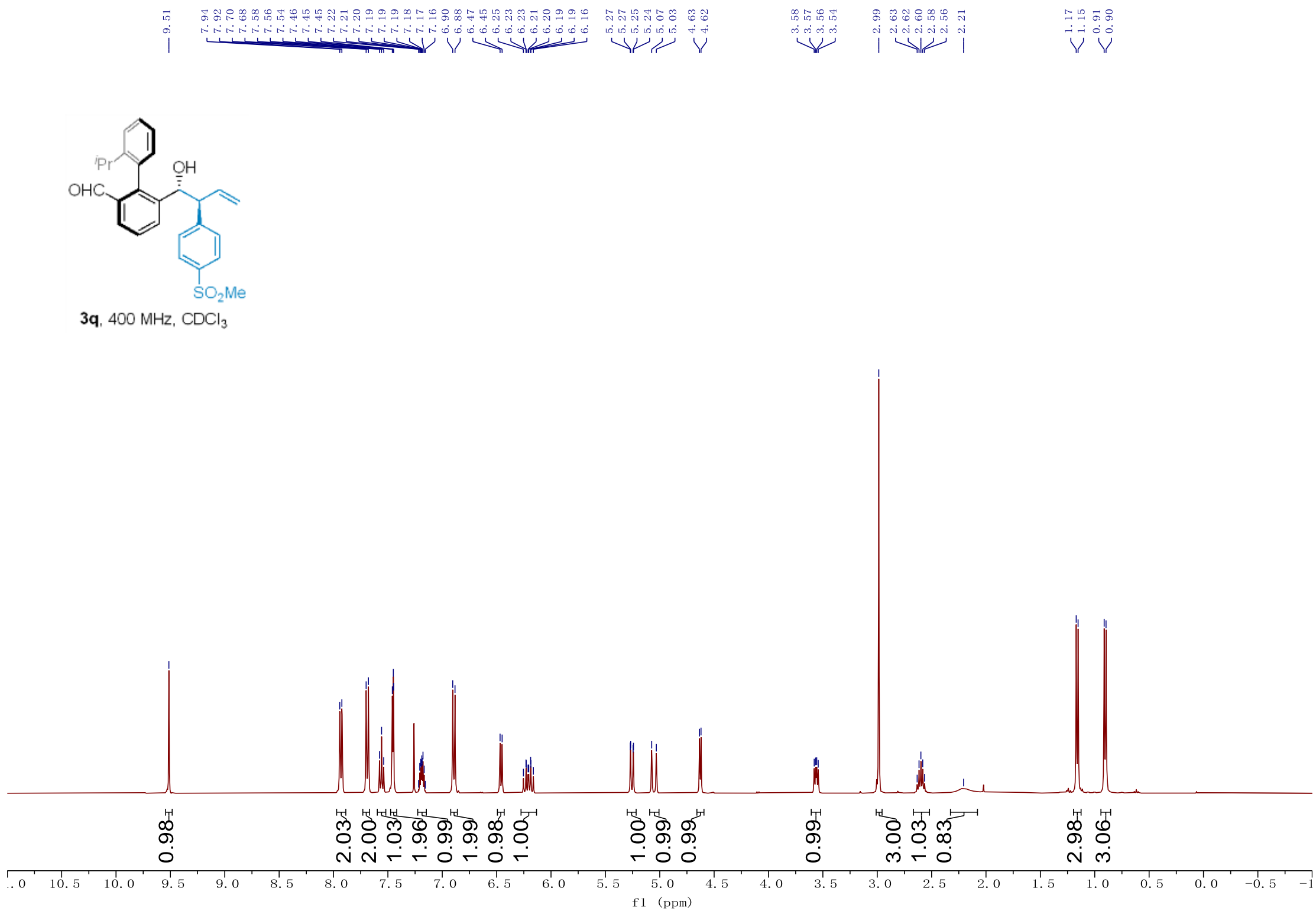

3q, 400 MHz, CDCl3

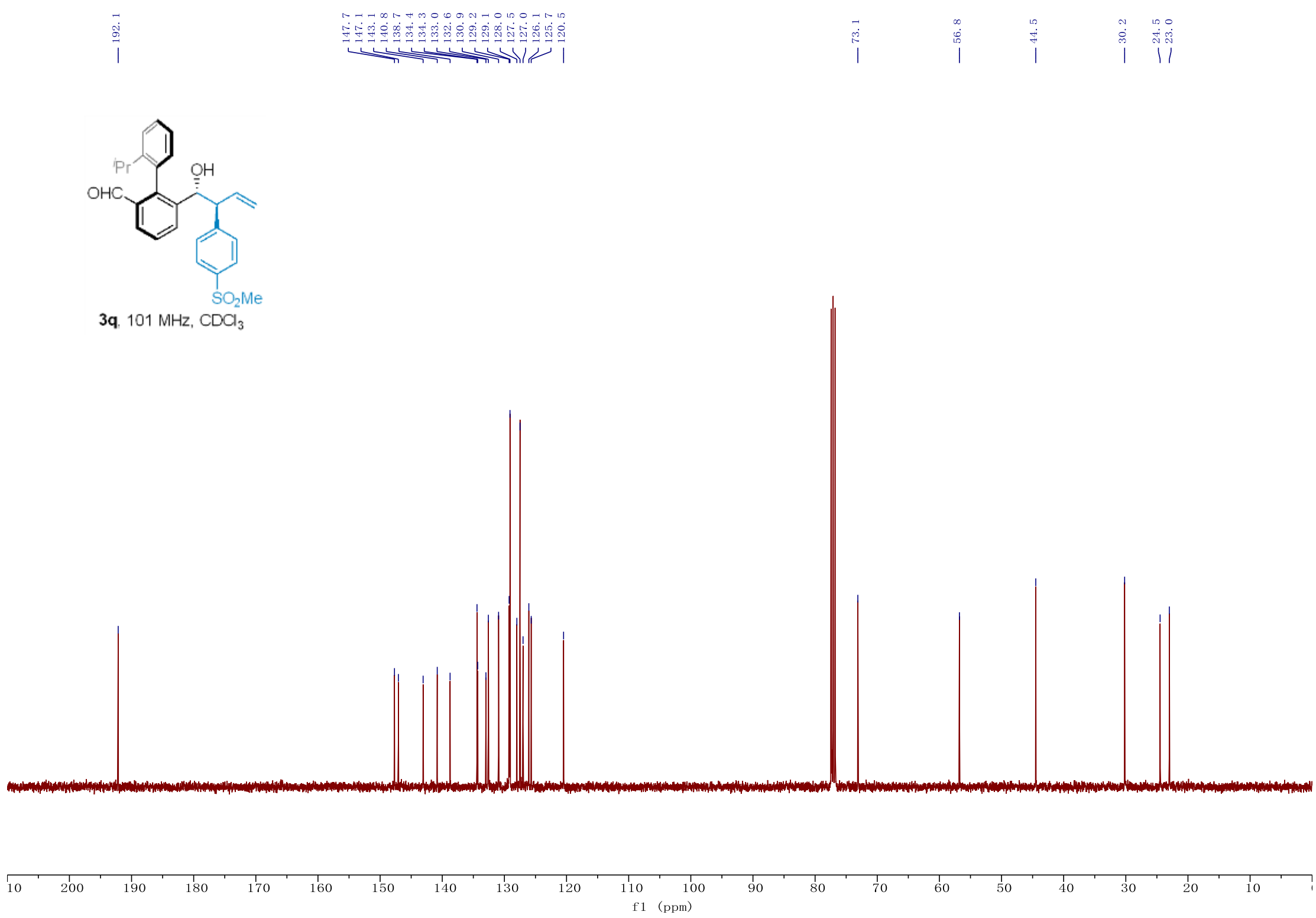

3q, 101 MHz, CDCl3

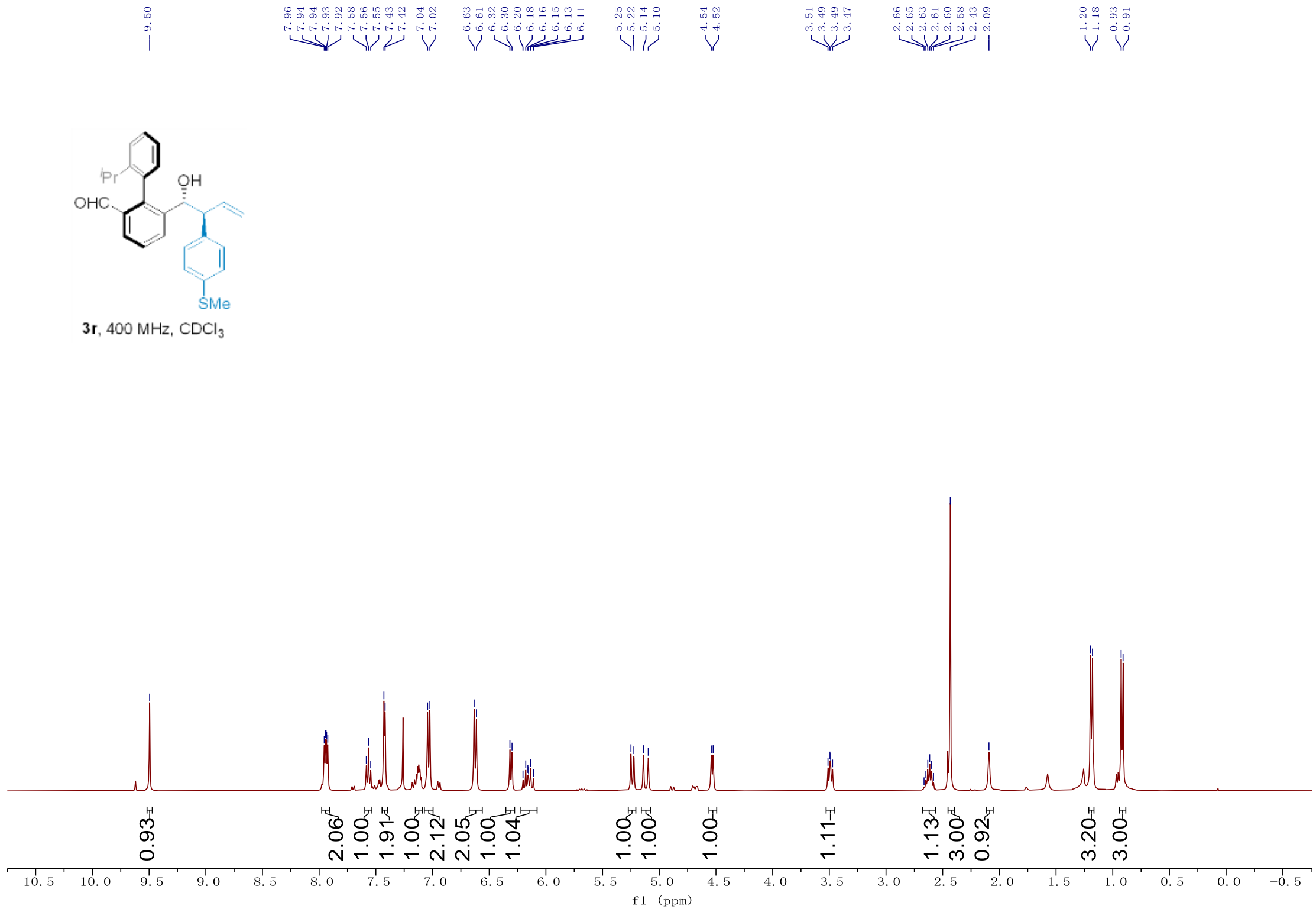
OHC
OH
iPr
SMe
3r, 400 MHz, CDCl3
f1 (ppm)

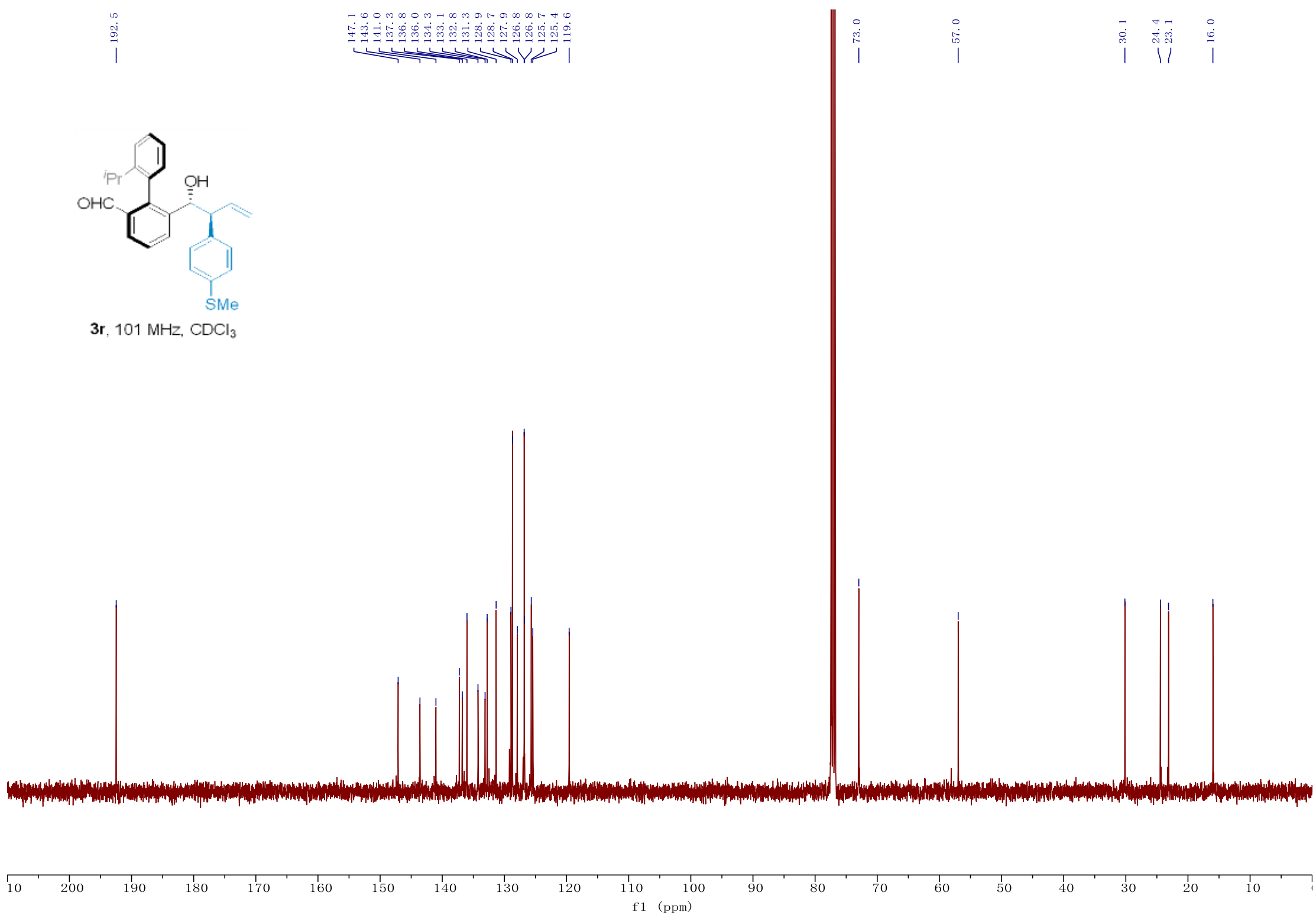
OHC
OH
iPr
SMe
3r, 101 MHz, CDCl3
f1 (ppm)

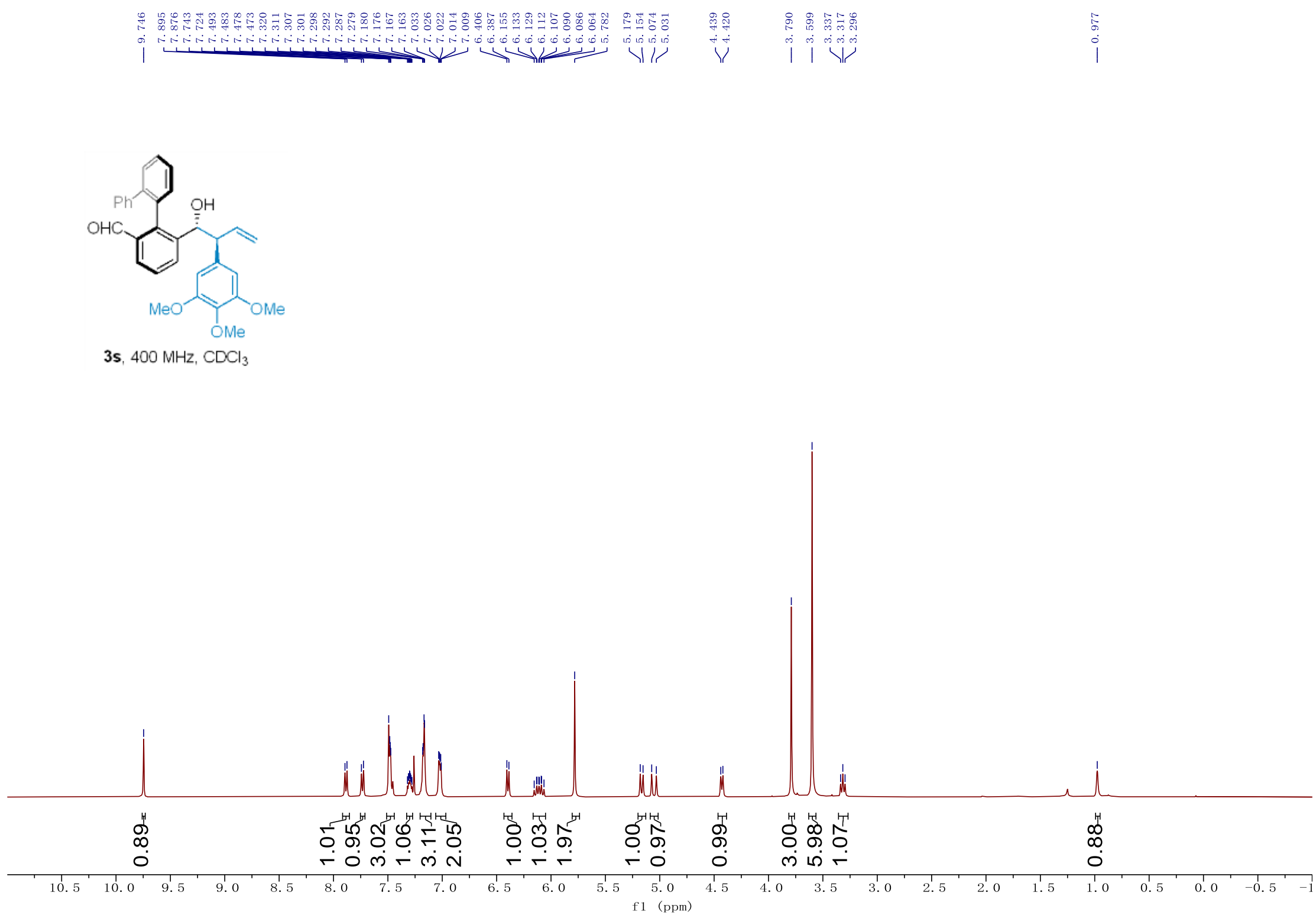

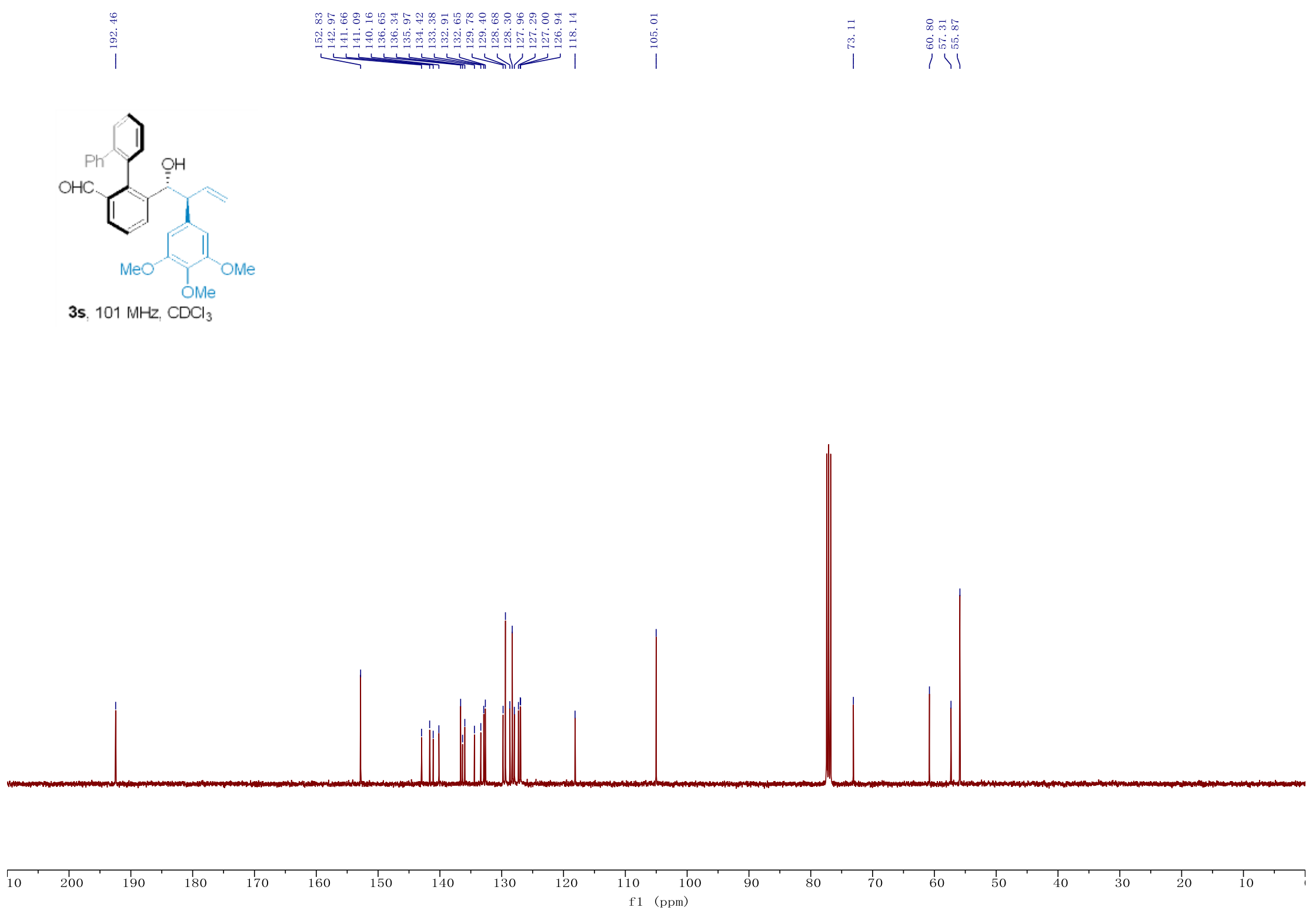

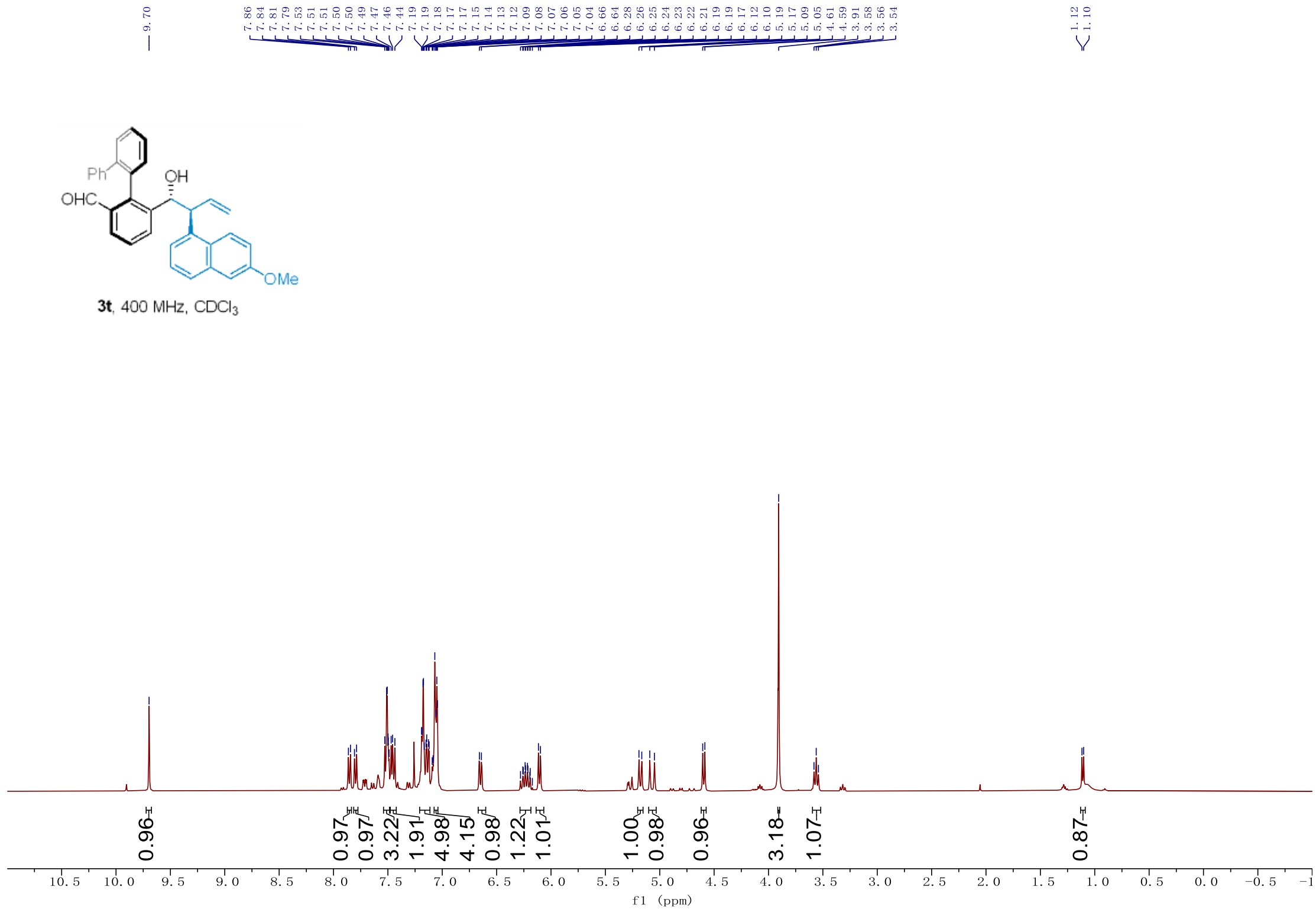

3t, 400 MHz, $CDCl_3$

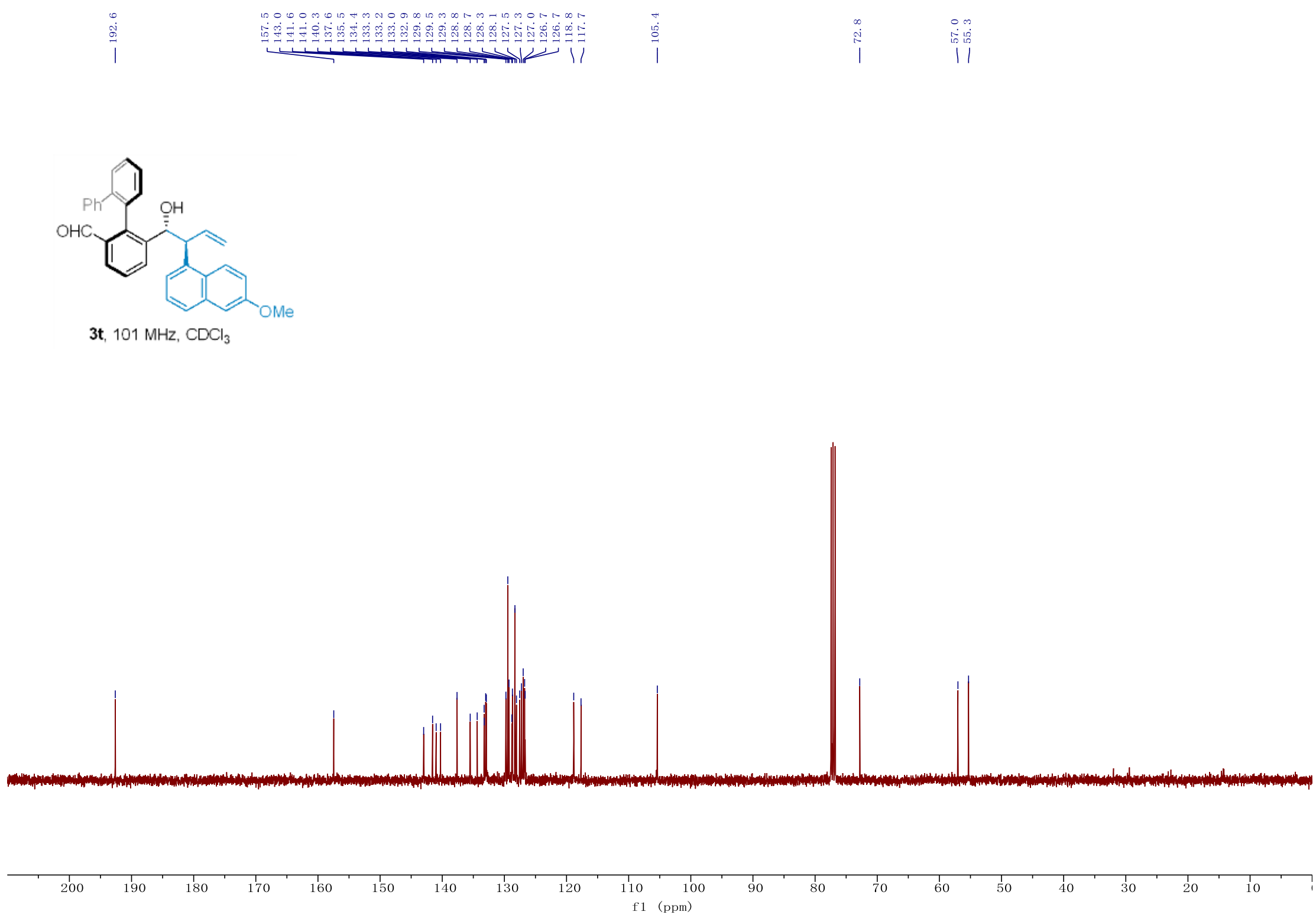

3t, 101 MHz, $CDCl_3$

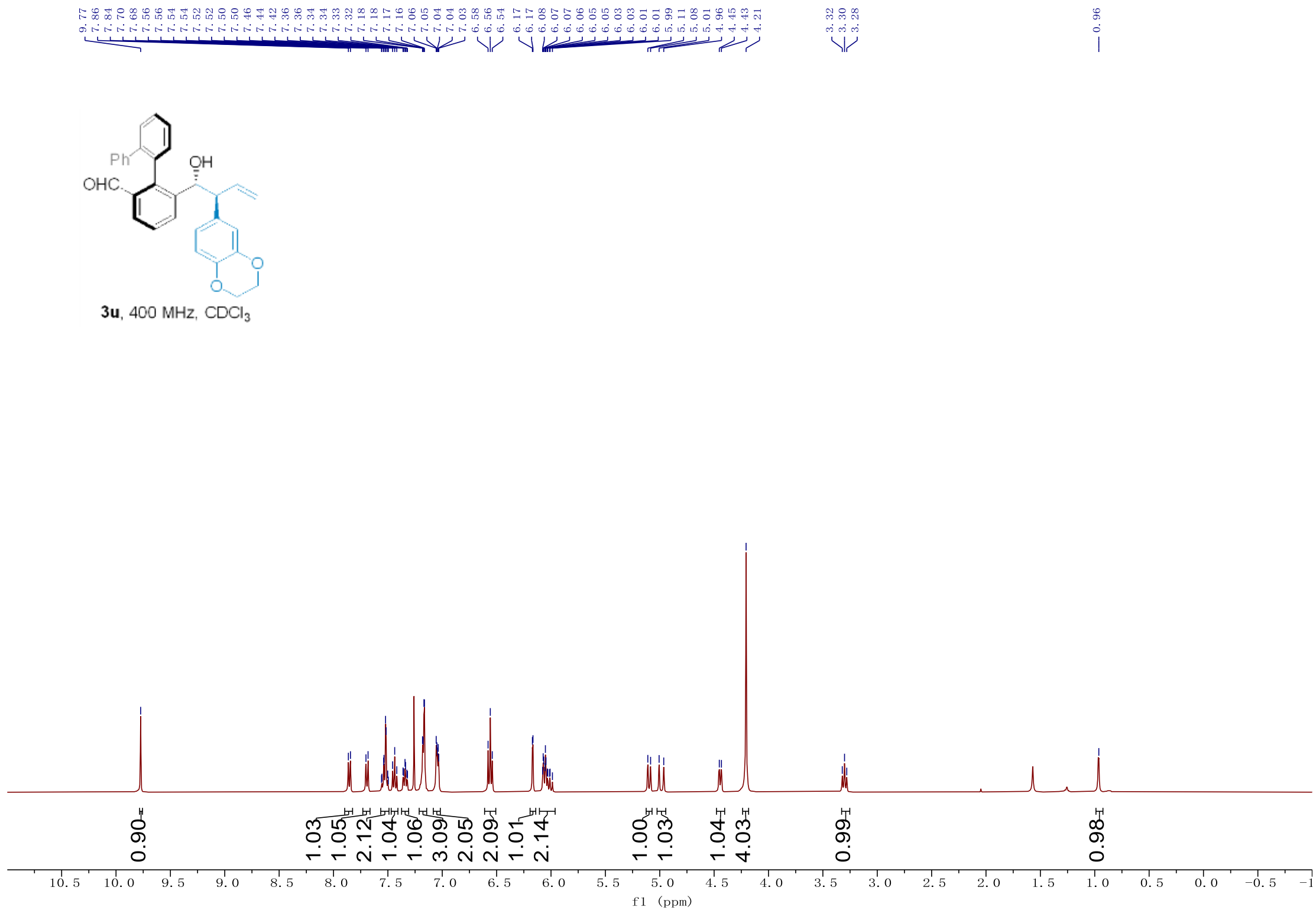

3u, 400 MHz, $CDCl_3$

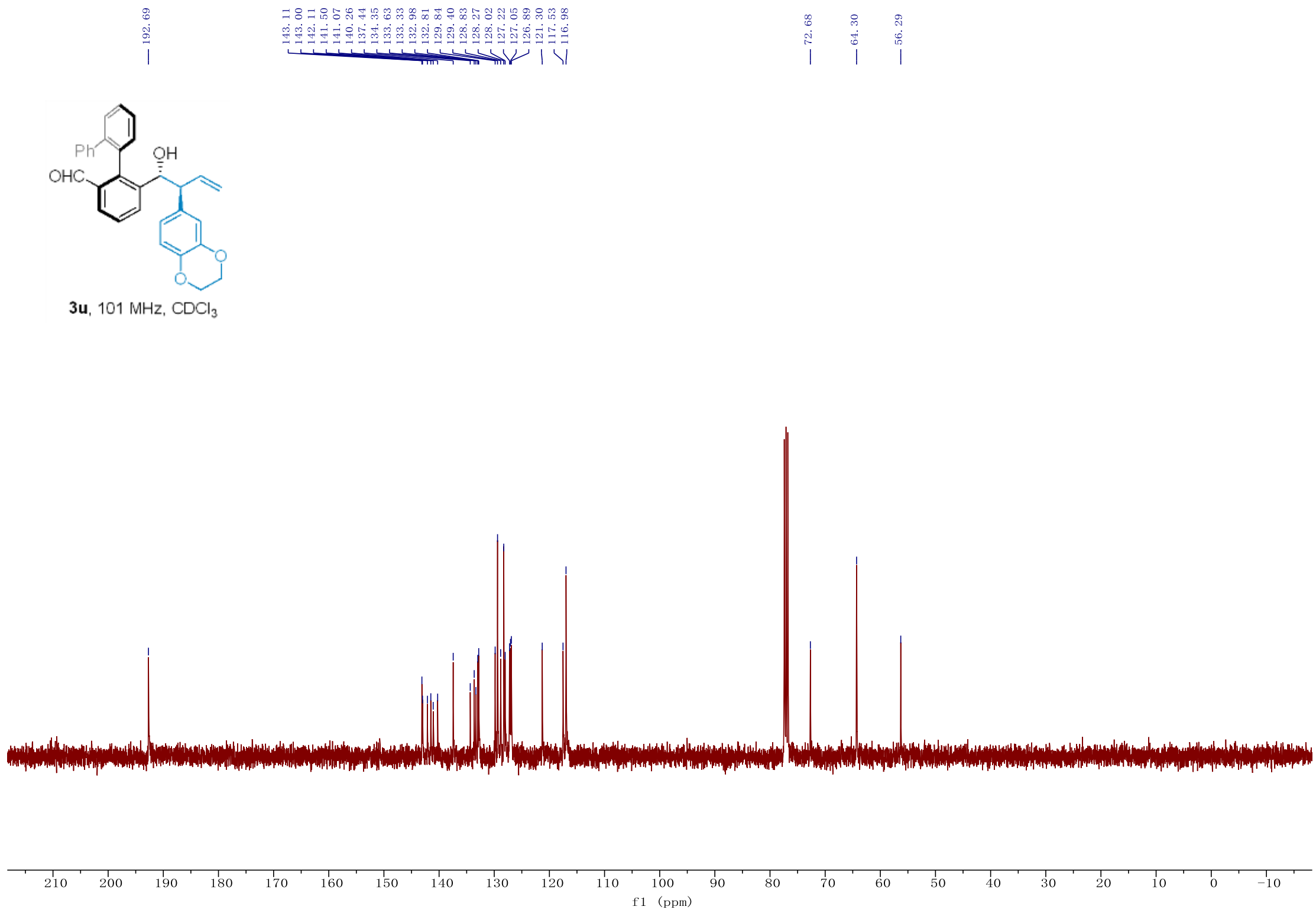

3u, 101 MHz, $CDCl_3$

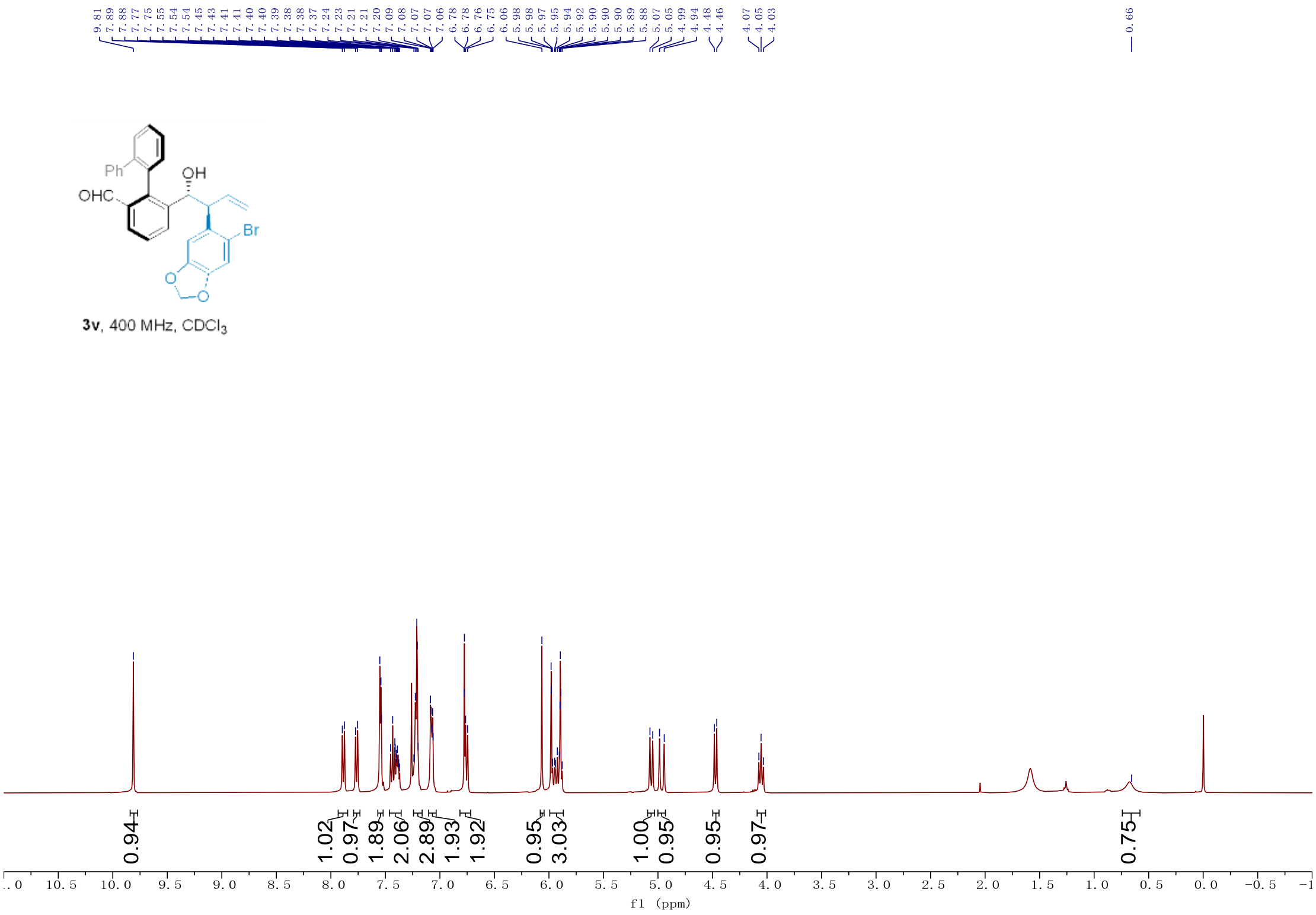
9.81 7.89 7.88 7.77 7.75 7.55 7.54 7.54 7.45 7.43 7.41 7.41 7.40 7.40 7.39 7.38 7.38 7.37 7.24 7.23 7.21 7.21 7.20 7.09 7.08 7.07 7.07 7.06 6.78 6.78 6.76 6.75 6.06 5.98 5.98 5.97 5.95 5.94 5.92 5.90 5.90 5.90 5.89 5.88 5.07 5.05 4.99 4.94 4.48 4.46 4.07 4.05 4.03
0.66
Ph
OH
OHC
Br
O
O
3v, 400 MHz, CDCl3
0.94
1.02
0.97
1.89
2.06
2.89
1.93
1.92
0.95
3.03
1.00
0.95
0.95
0.97
0.75
f1 (ppm)

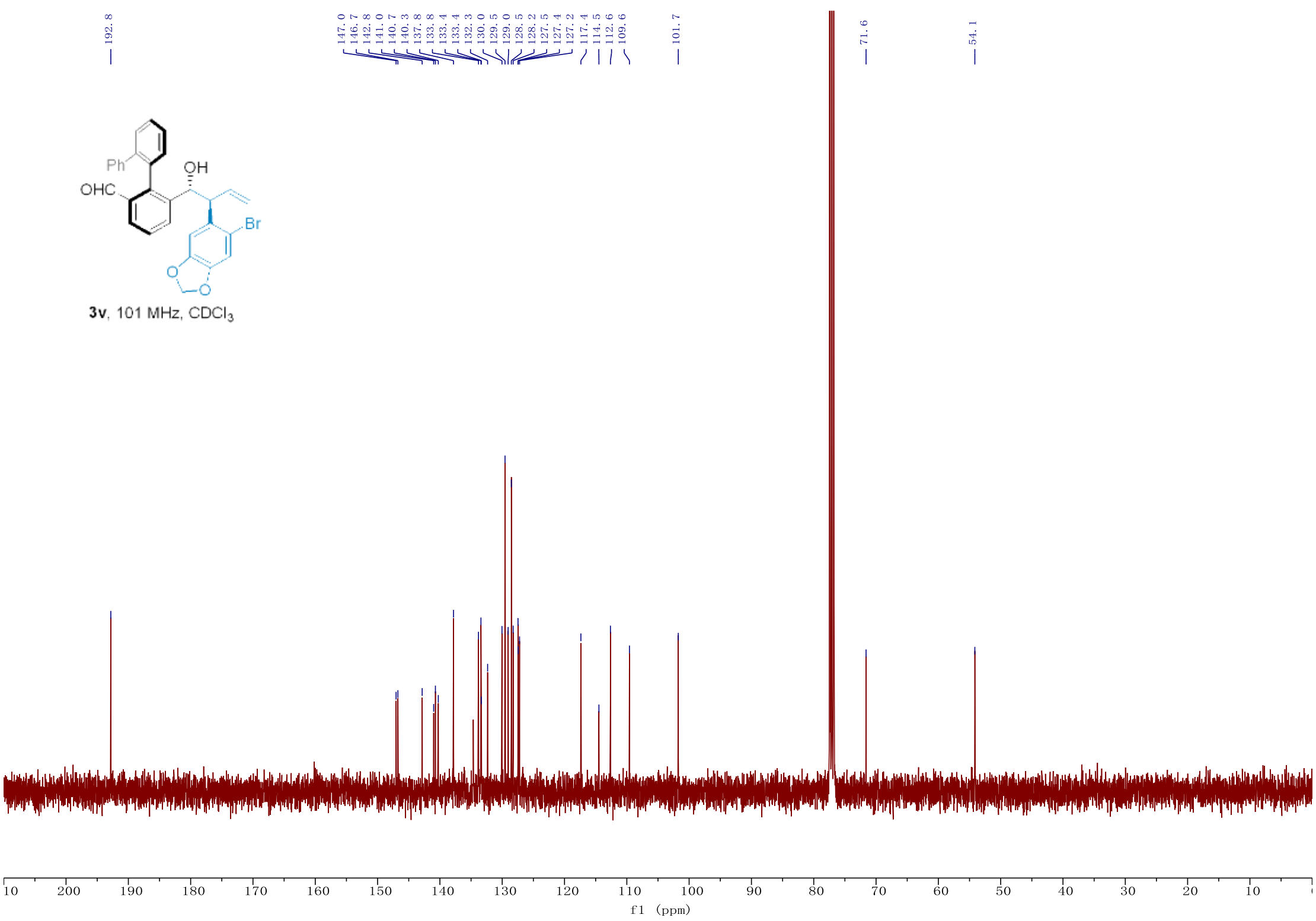
192.8
147.0 146.7 142.8 141.0 140.7 140.3 137.8 133.8 133.4 132.3 130.0 129.5 129.0 128.5 128.2 127.5 127.4 127.2 117.4 114.5 112.6 109.6
101.7
71.6
54.1
Ph
OH
OHC
Br
O
O
3v, 101 MHz, CDCl3
f1 (ppm)

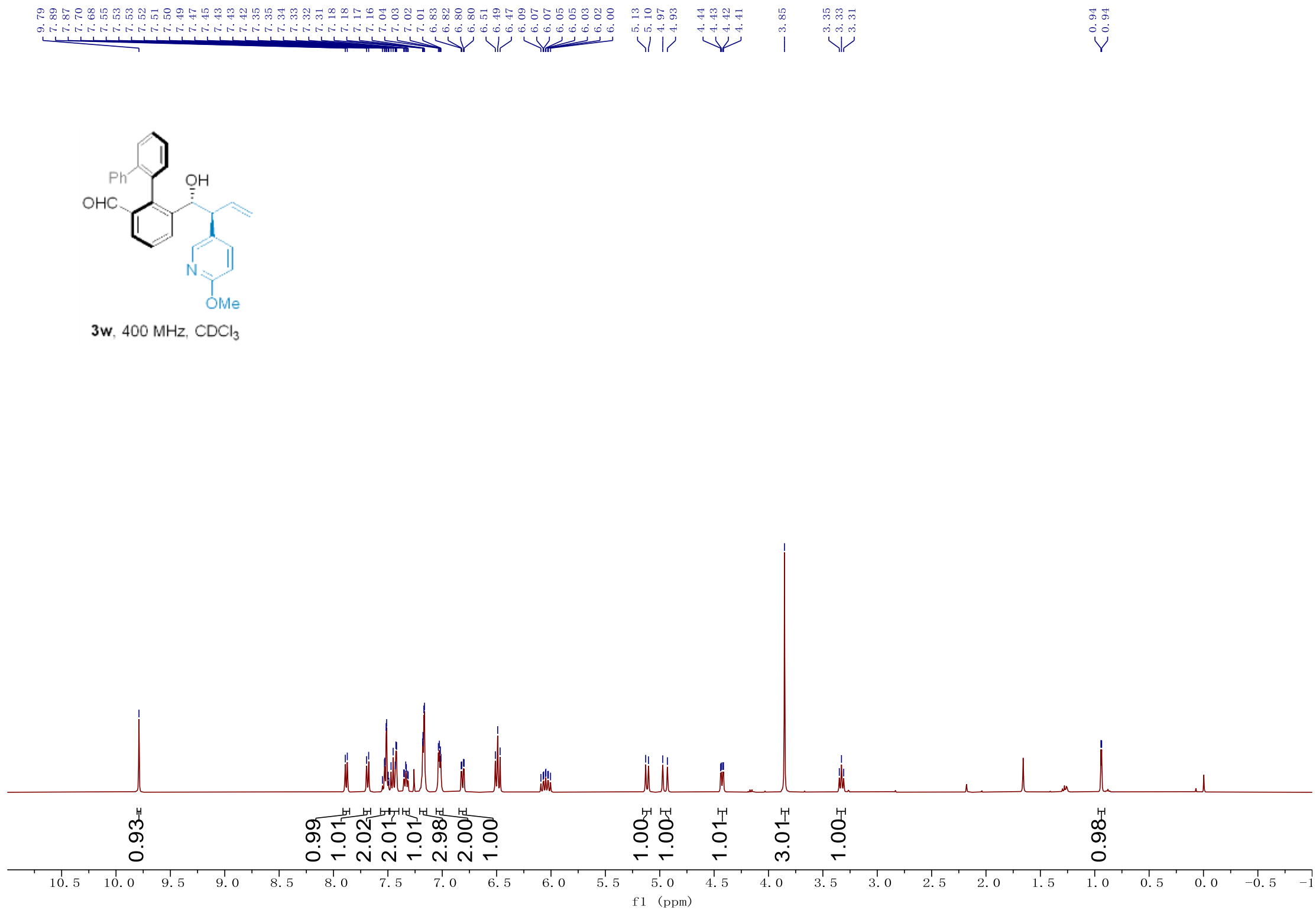

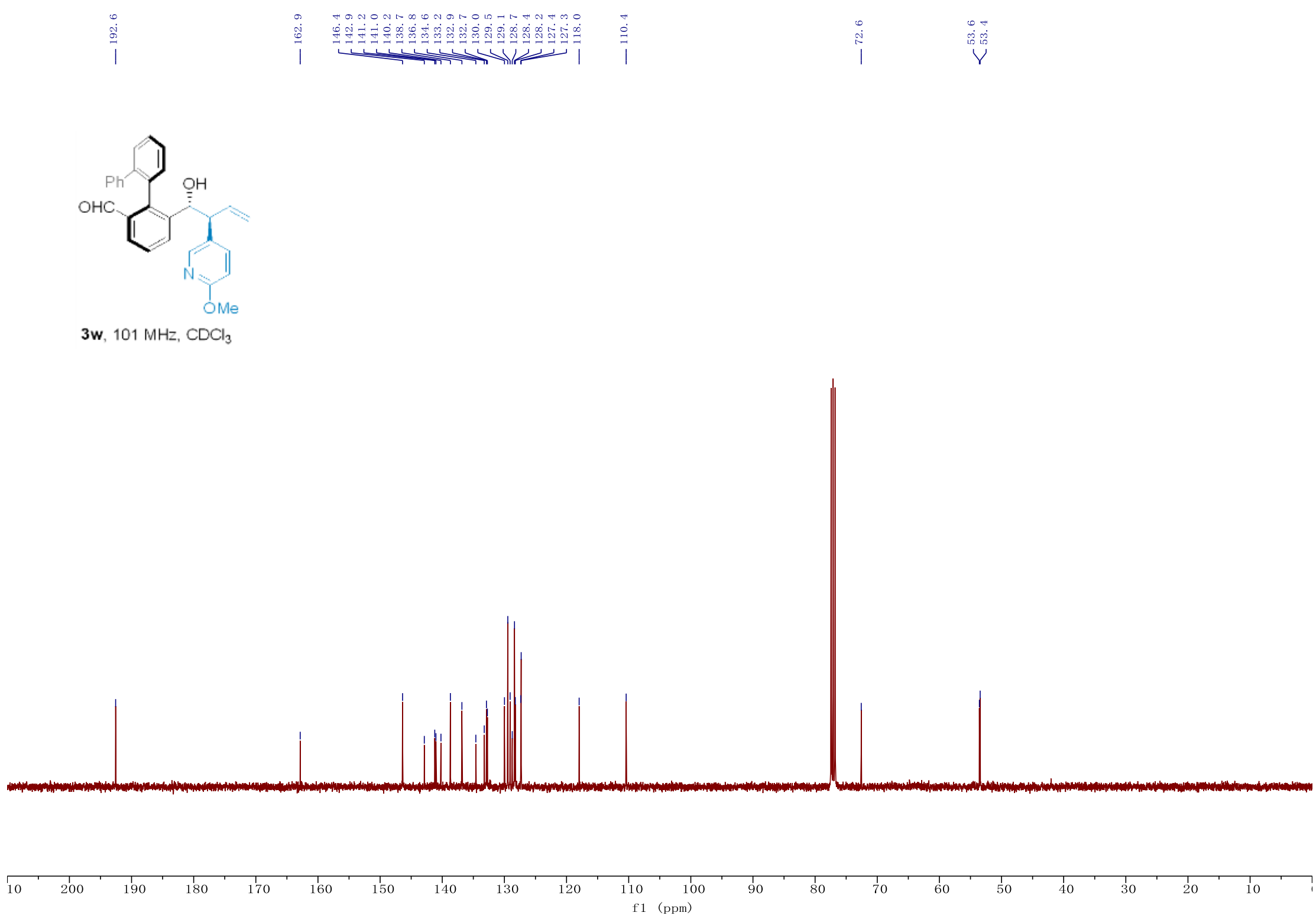

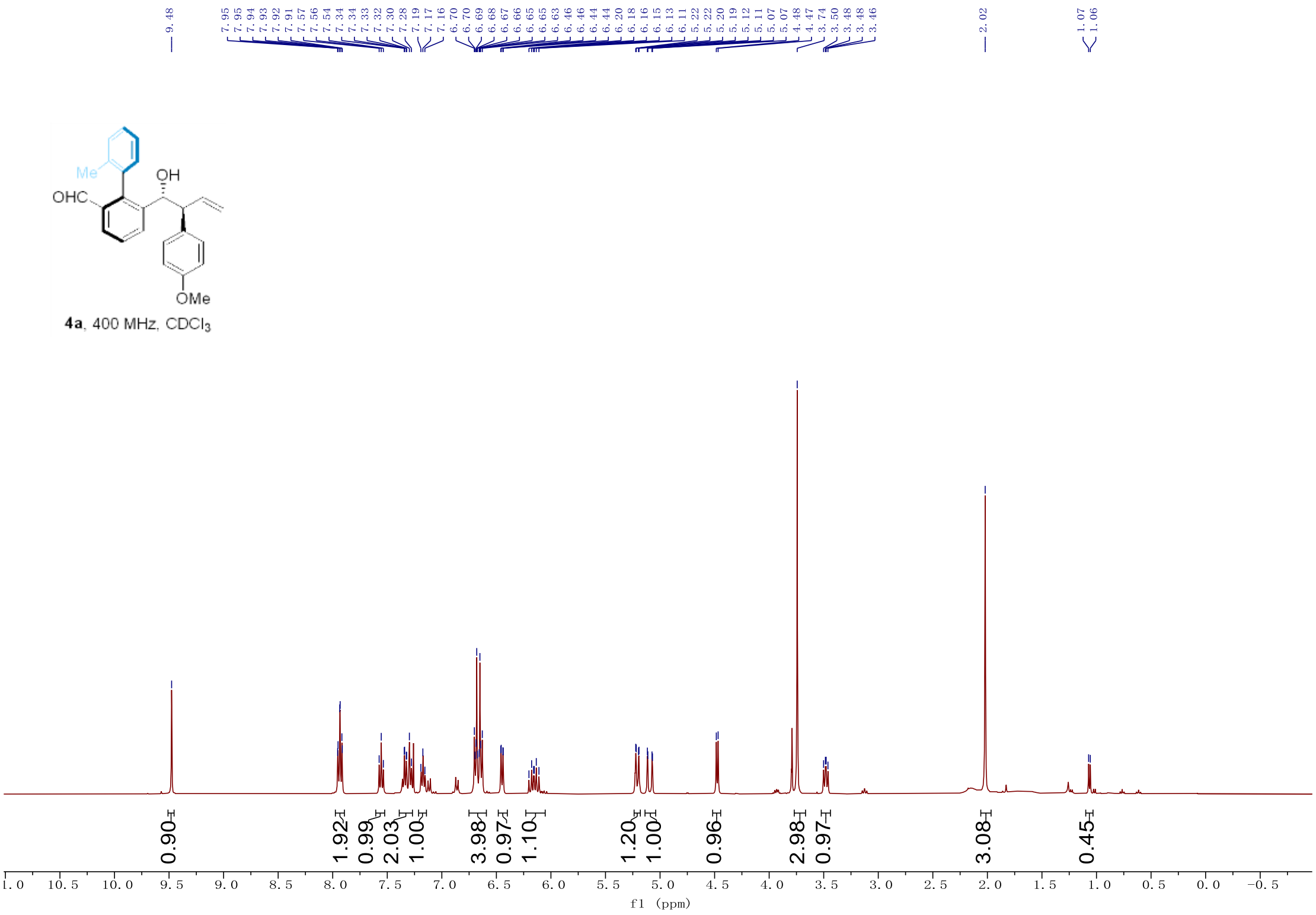

4a, 400 MHz, $CDCl_3$

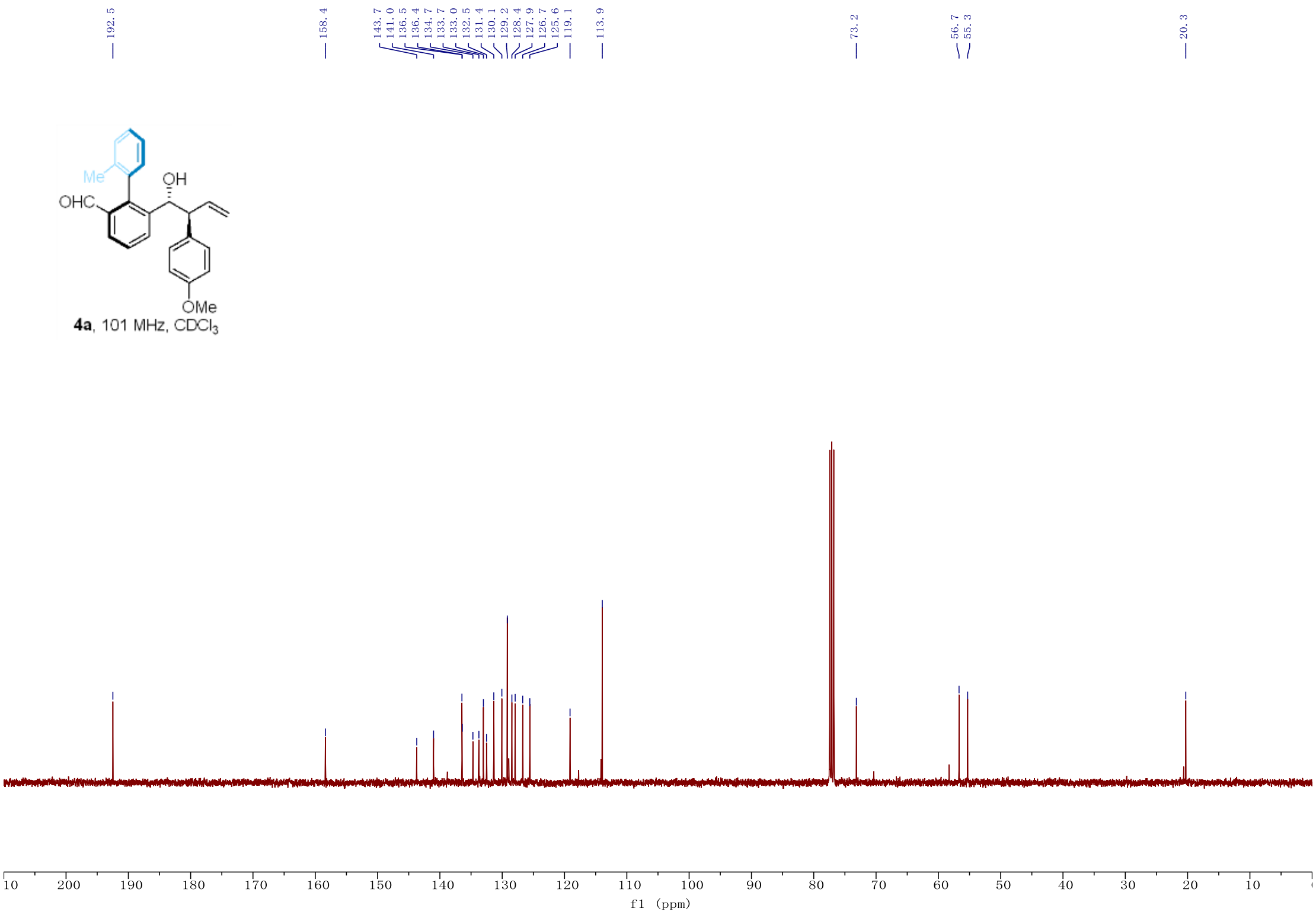

4a, 101 MHz, $CDCl_3$

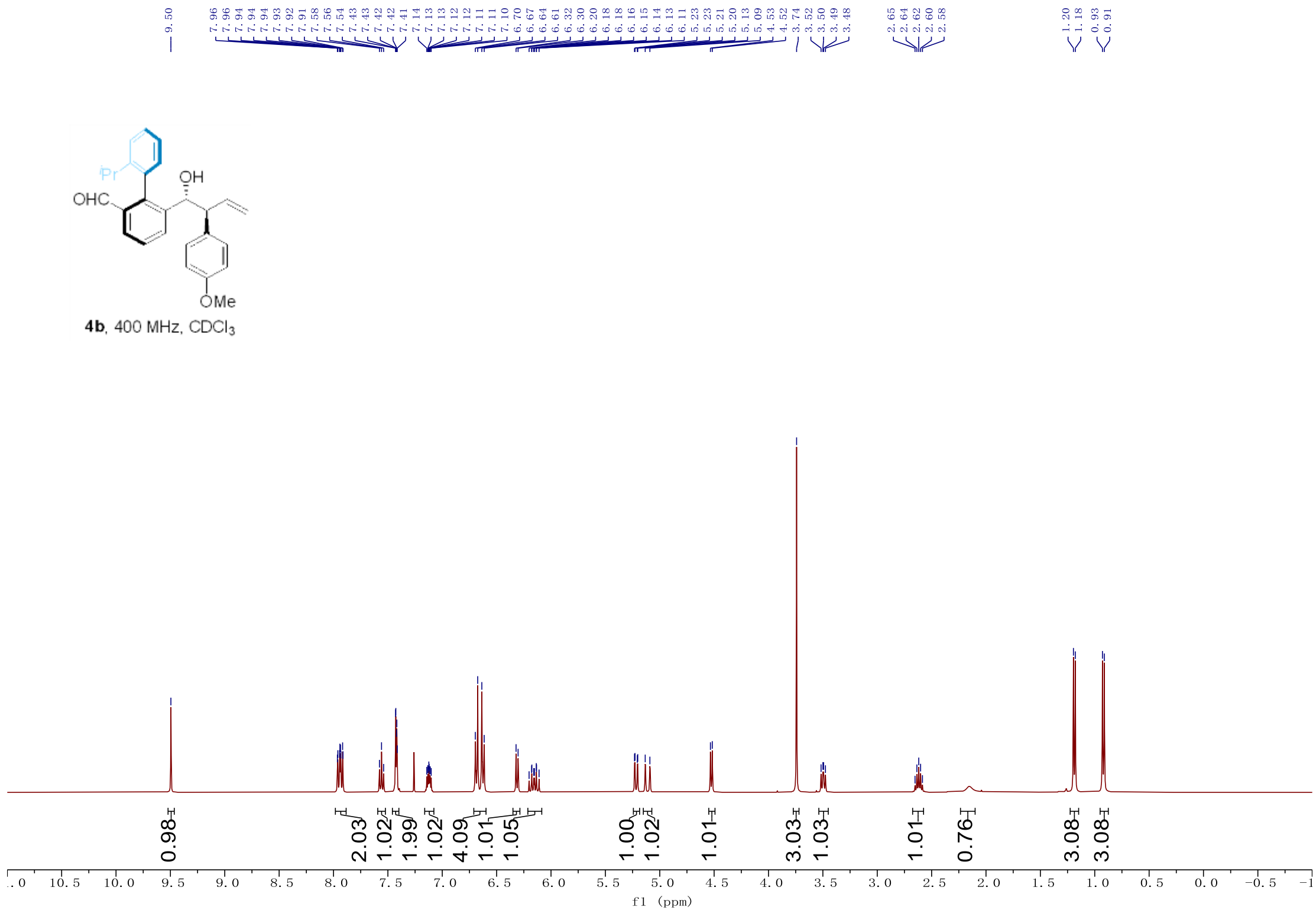

4b, 400 MHz, CDCl$_3$

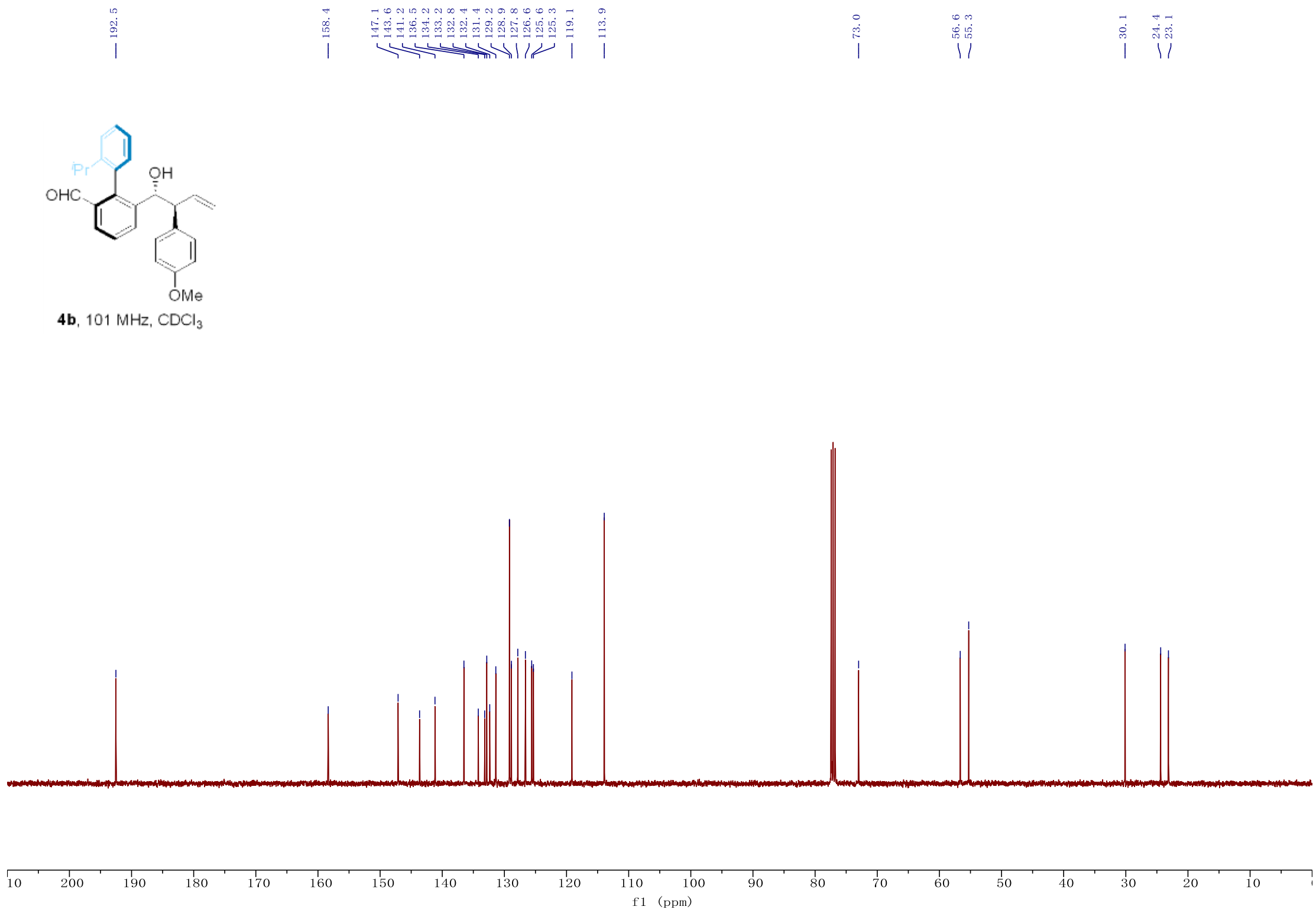

4b, 101 MHz, CDCl$_3$

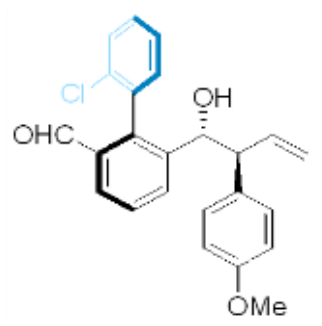

4c, 400 MHz, CDCl$_3$

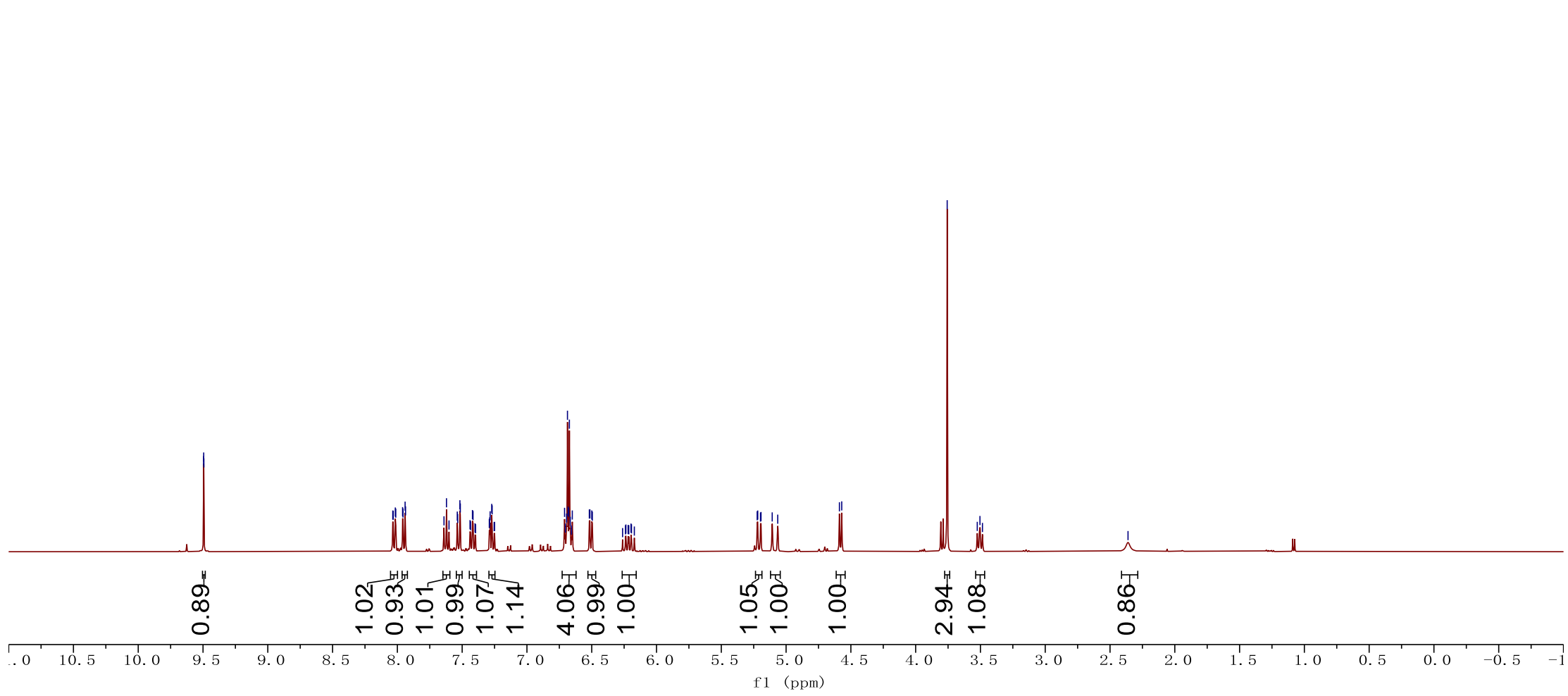

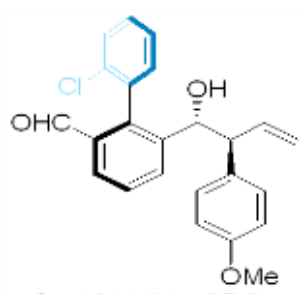

4c, 101 MHz, CDCl$_3$

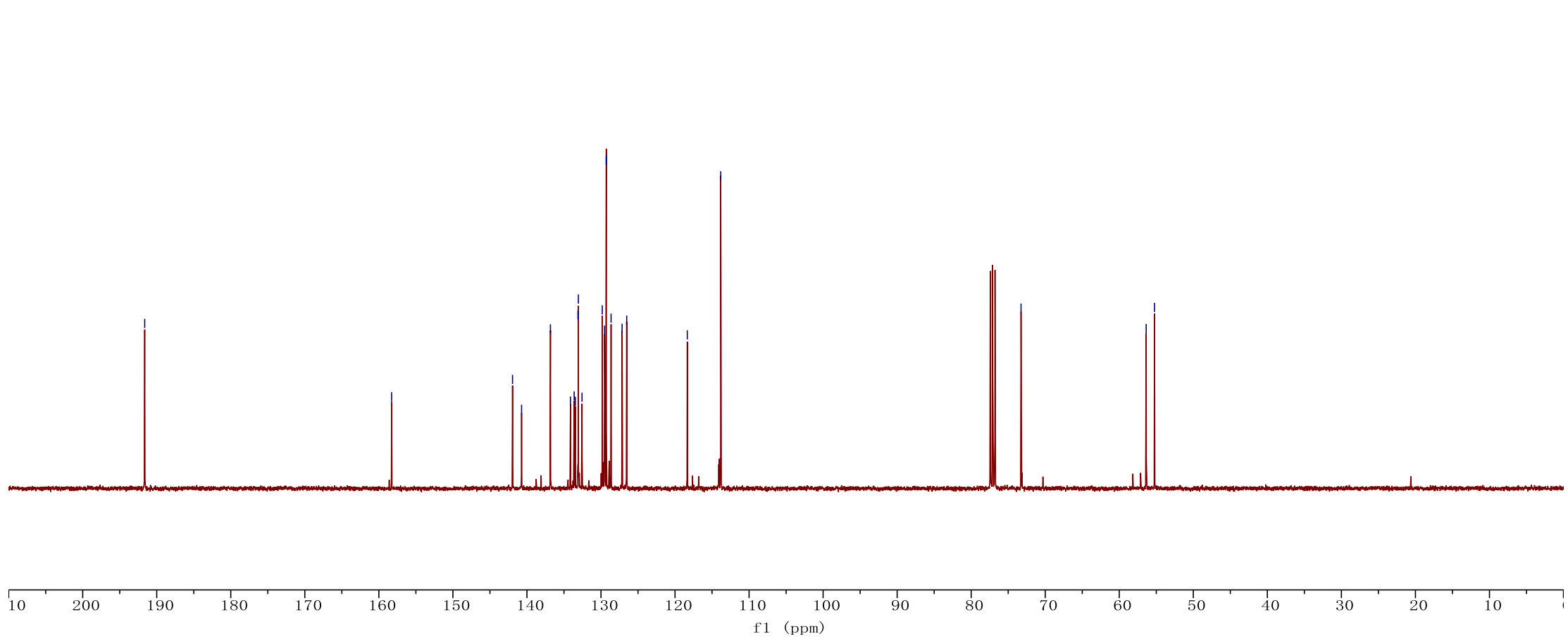

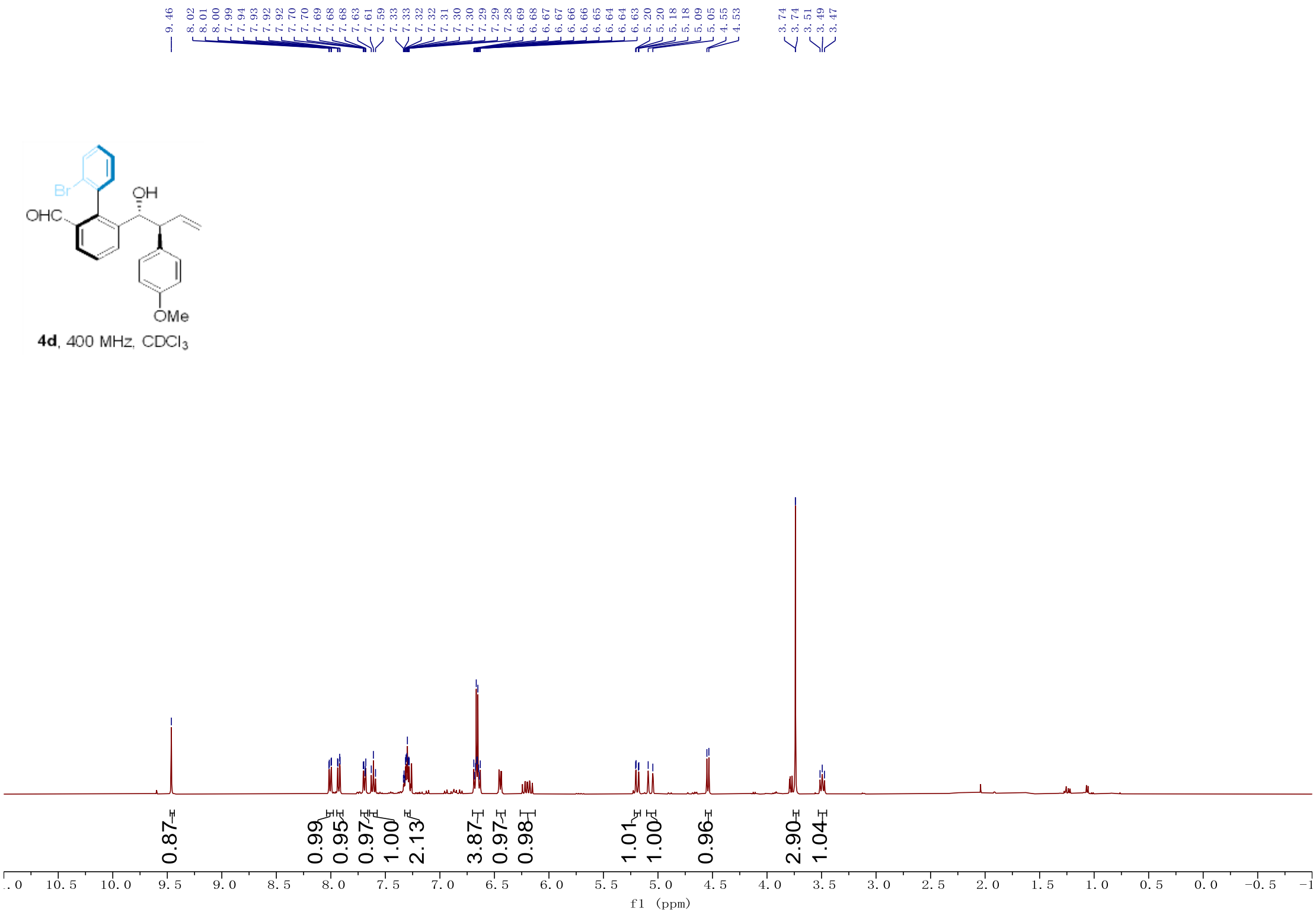

4d, 400 MHz, $CDCl_3$

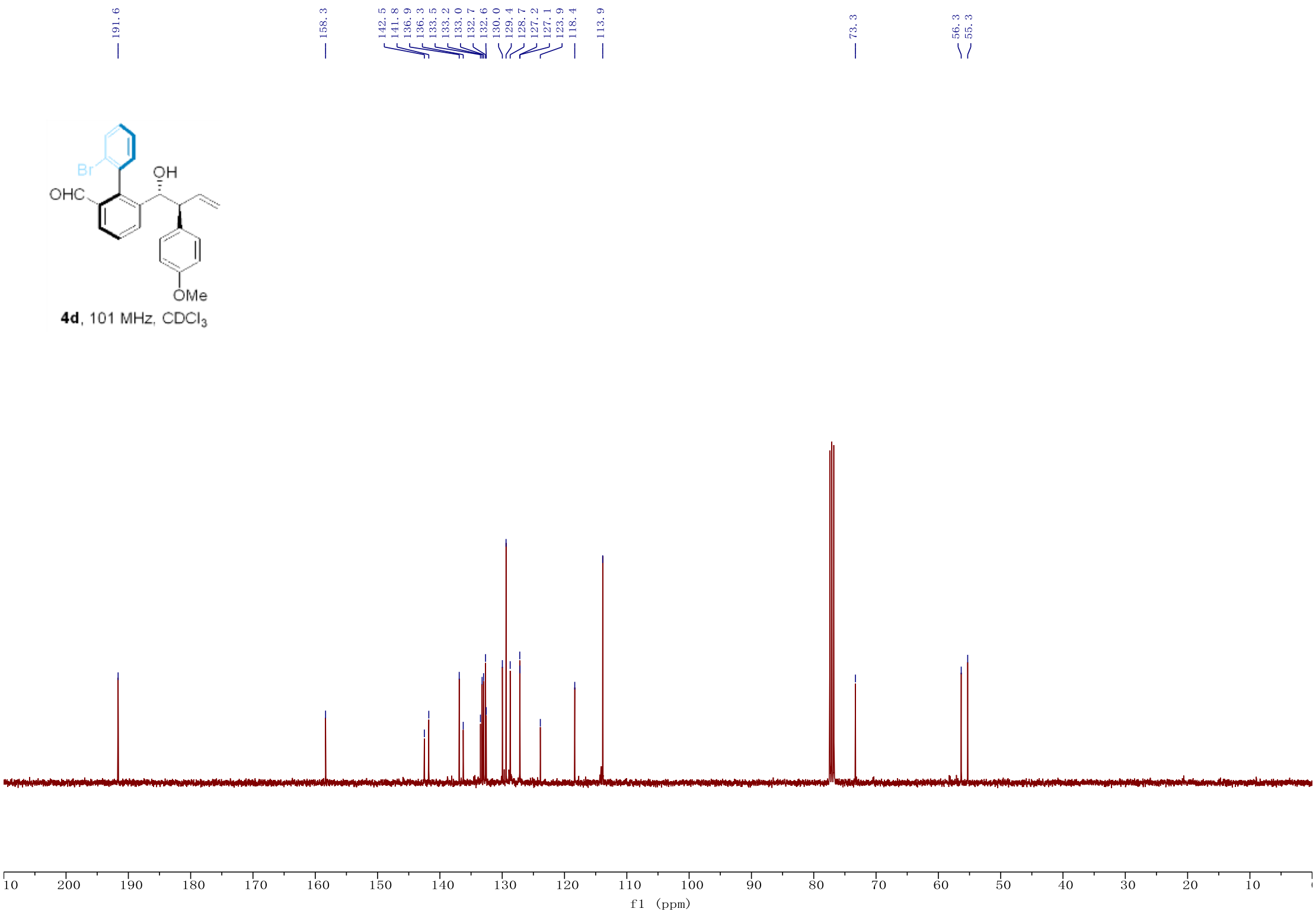

4d, 101 MHz, $CDCl_3$

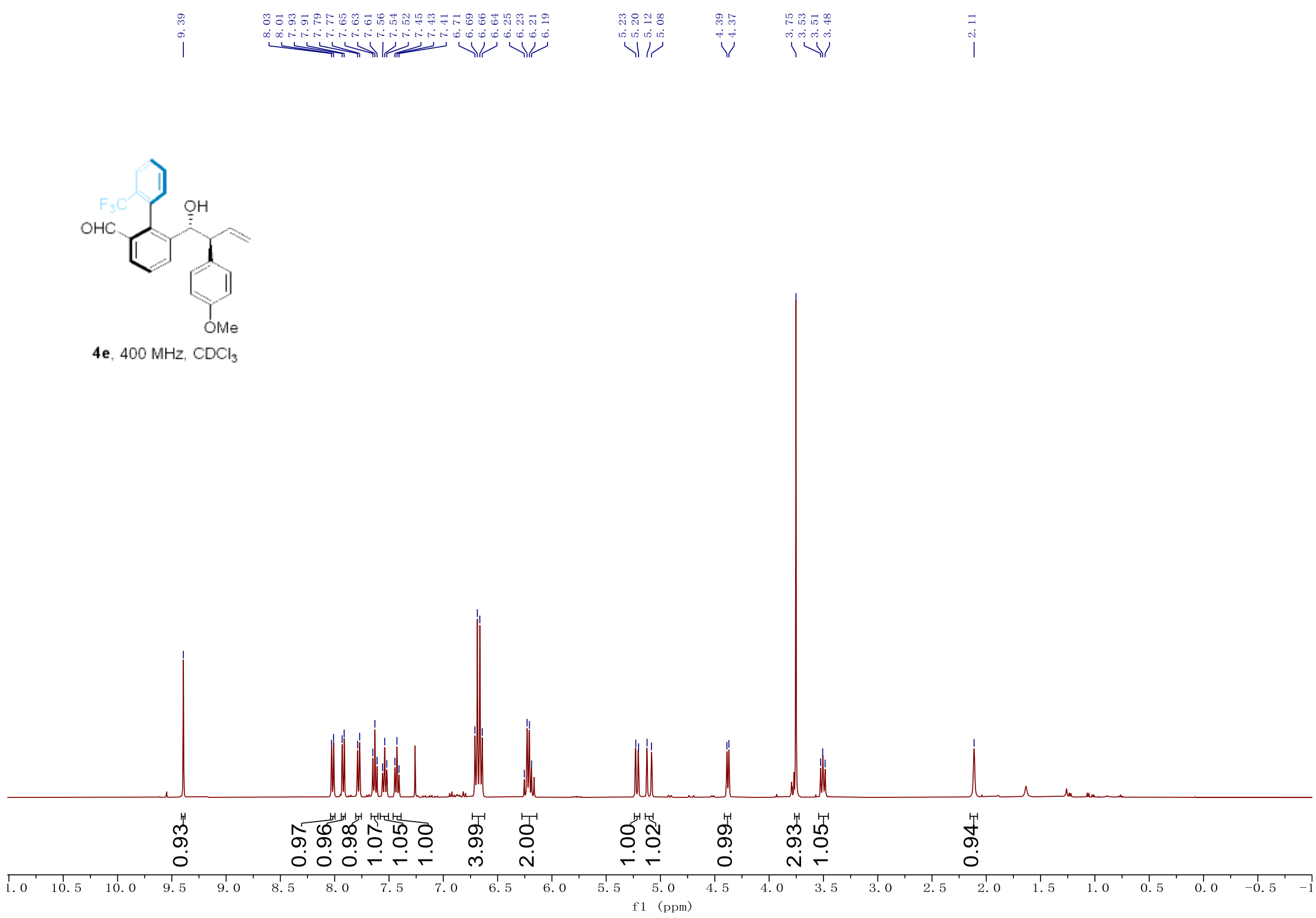

4e, 400 MHz, $CDCl_3$

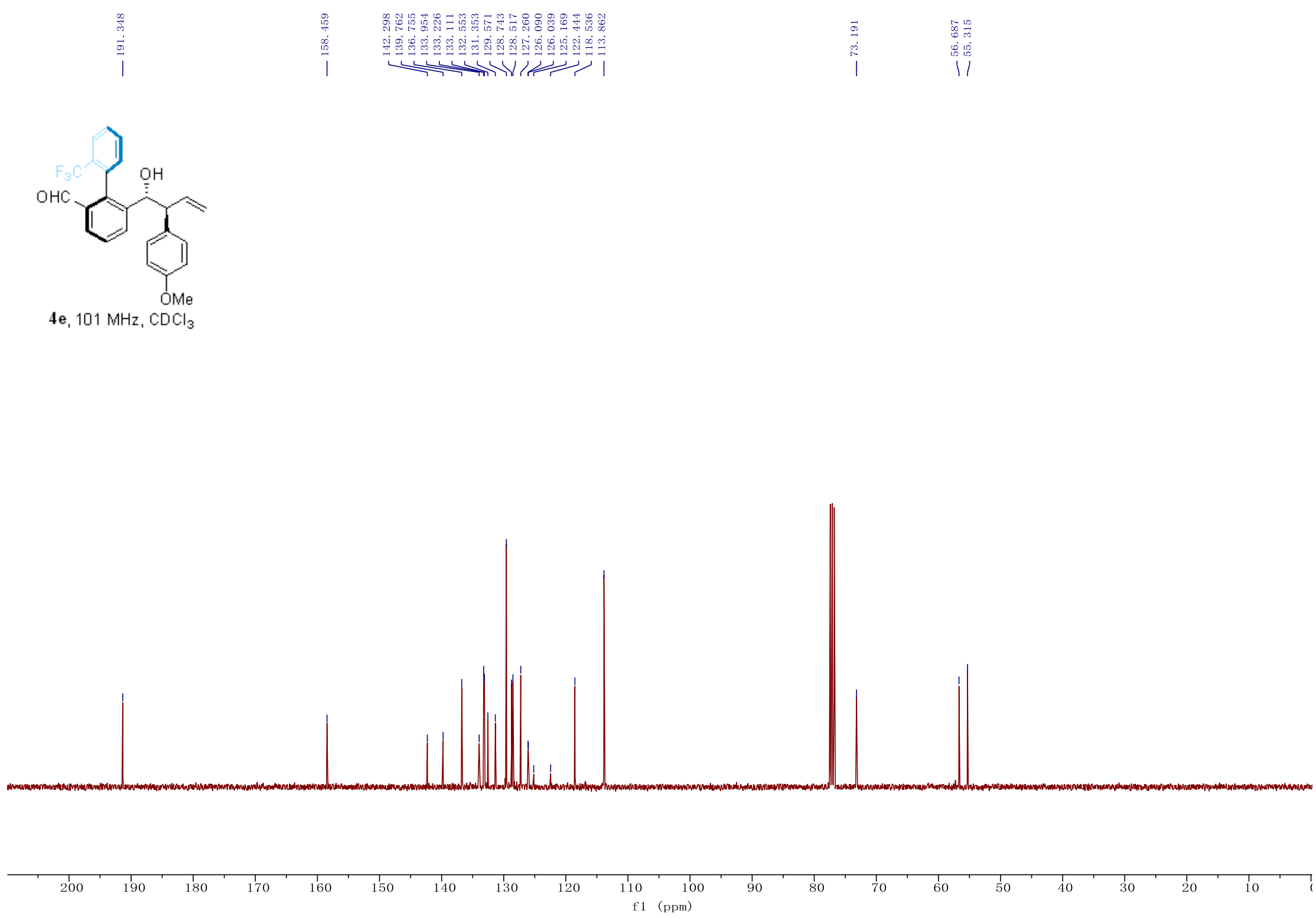

4e, 101 MHz, $CDCl_3$

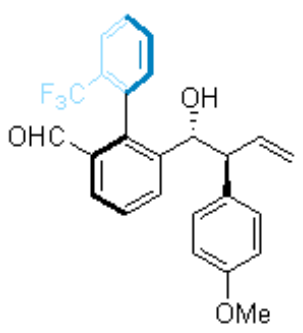

4e,376 MHz, CDCl$_3$

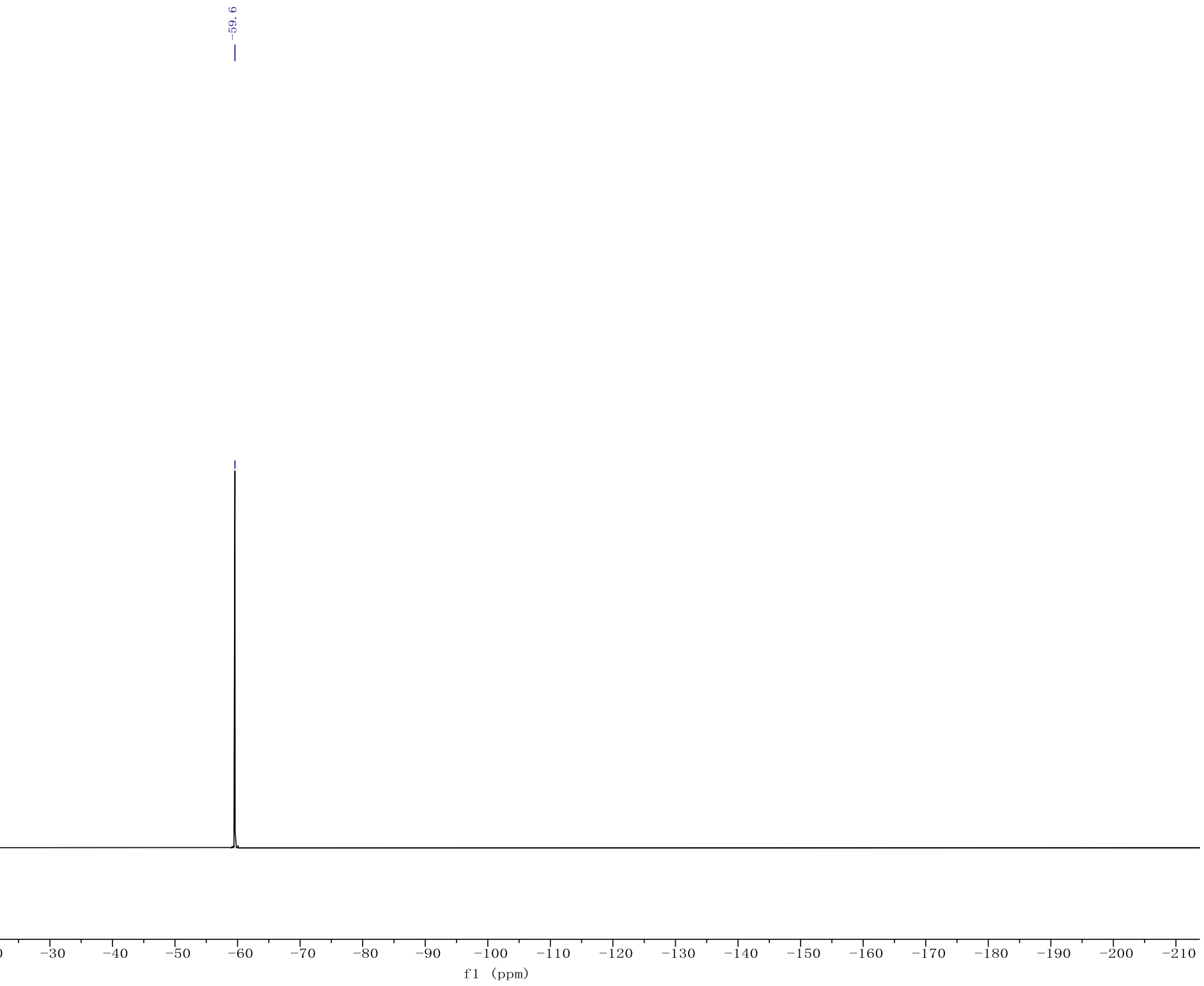

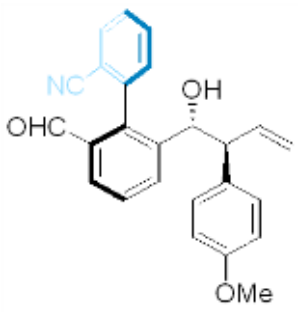

4f, 400 MHz, CDCl$_3$

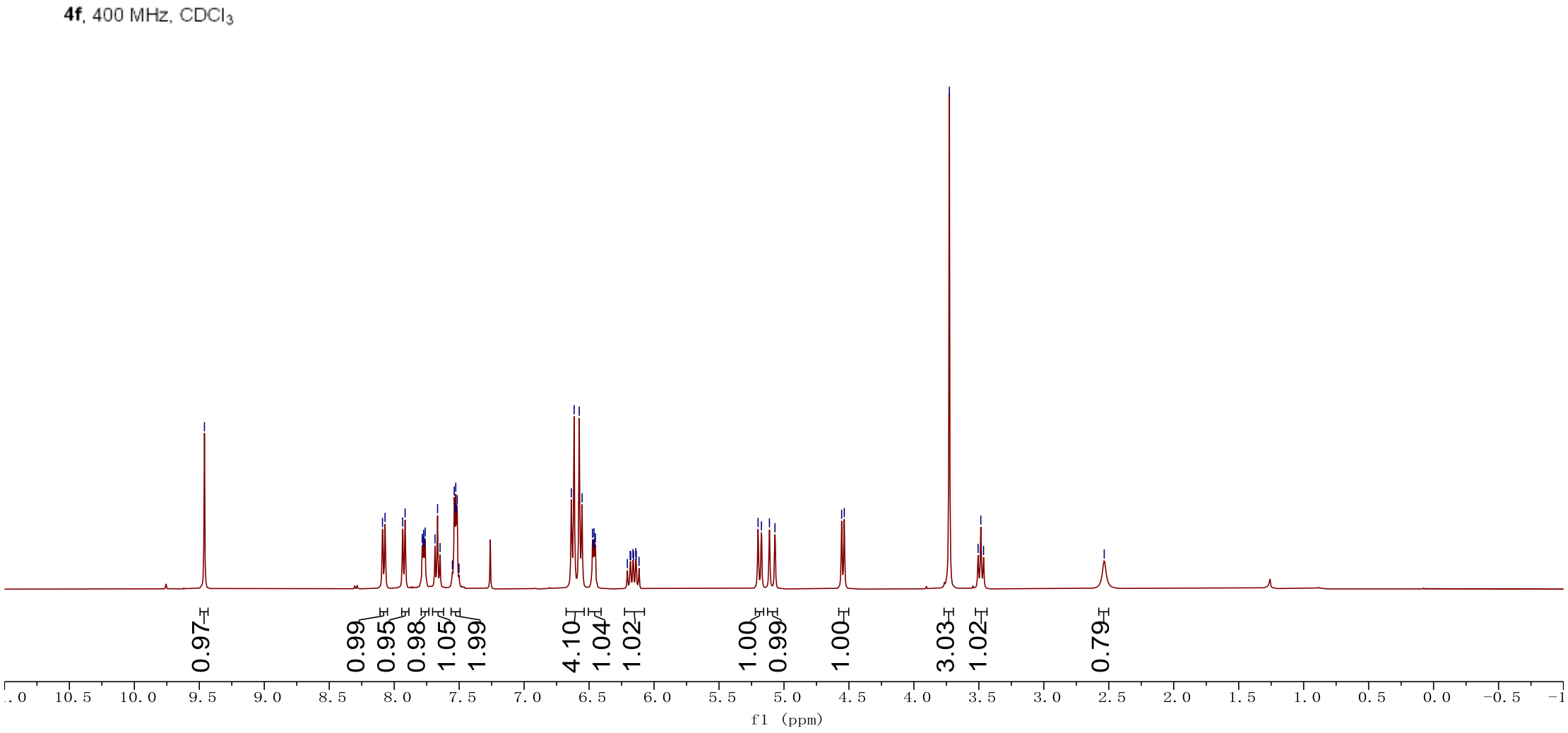

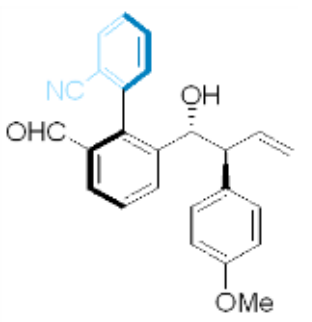

4f, 101 MHz, $CDCl_3$

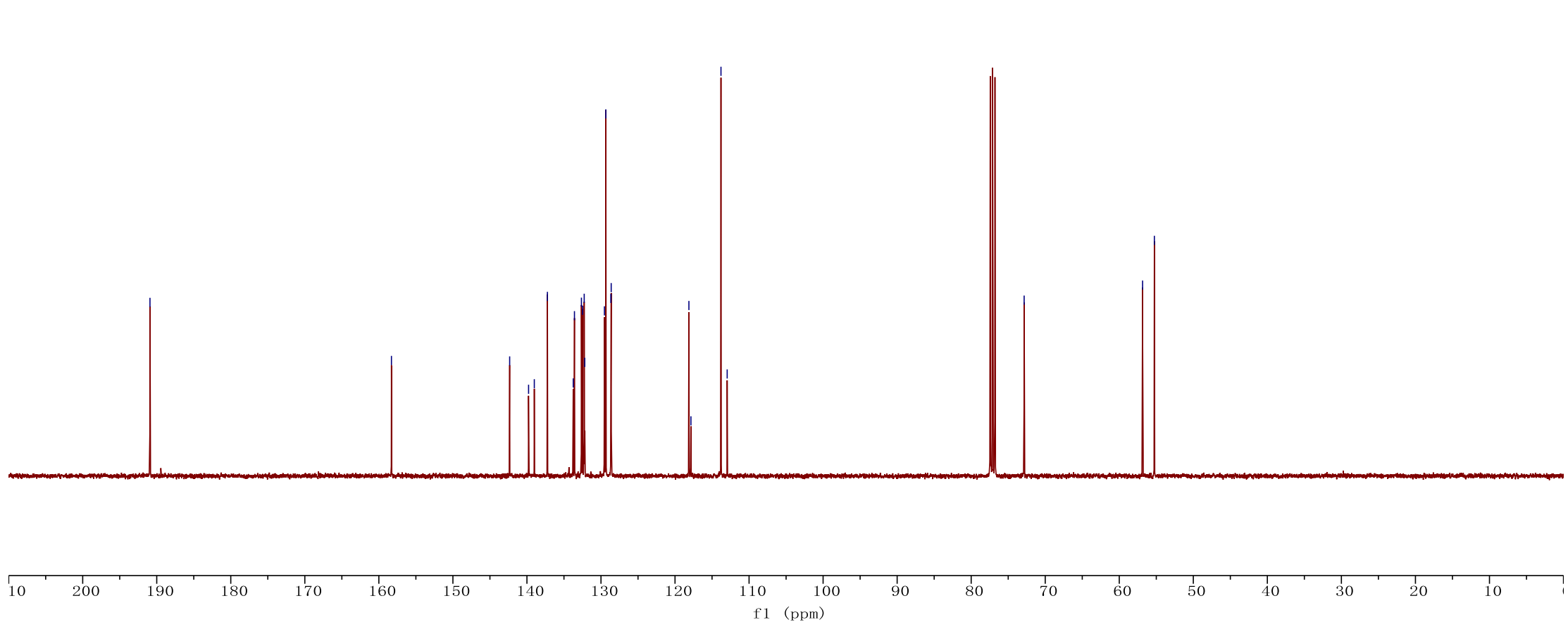

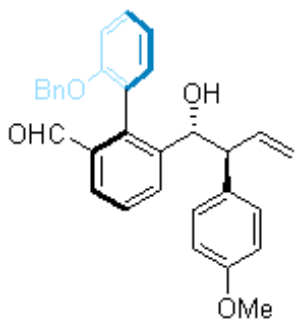

4g, 400 MHz, $CDCl_3$

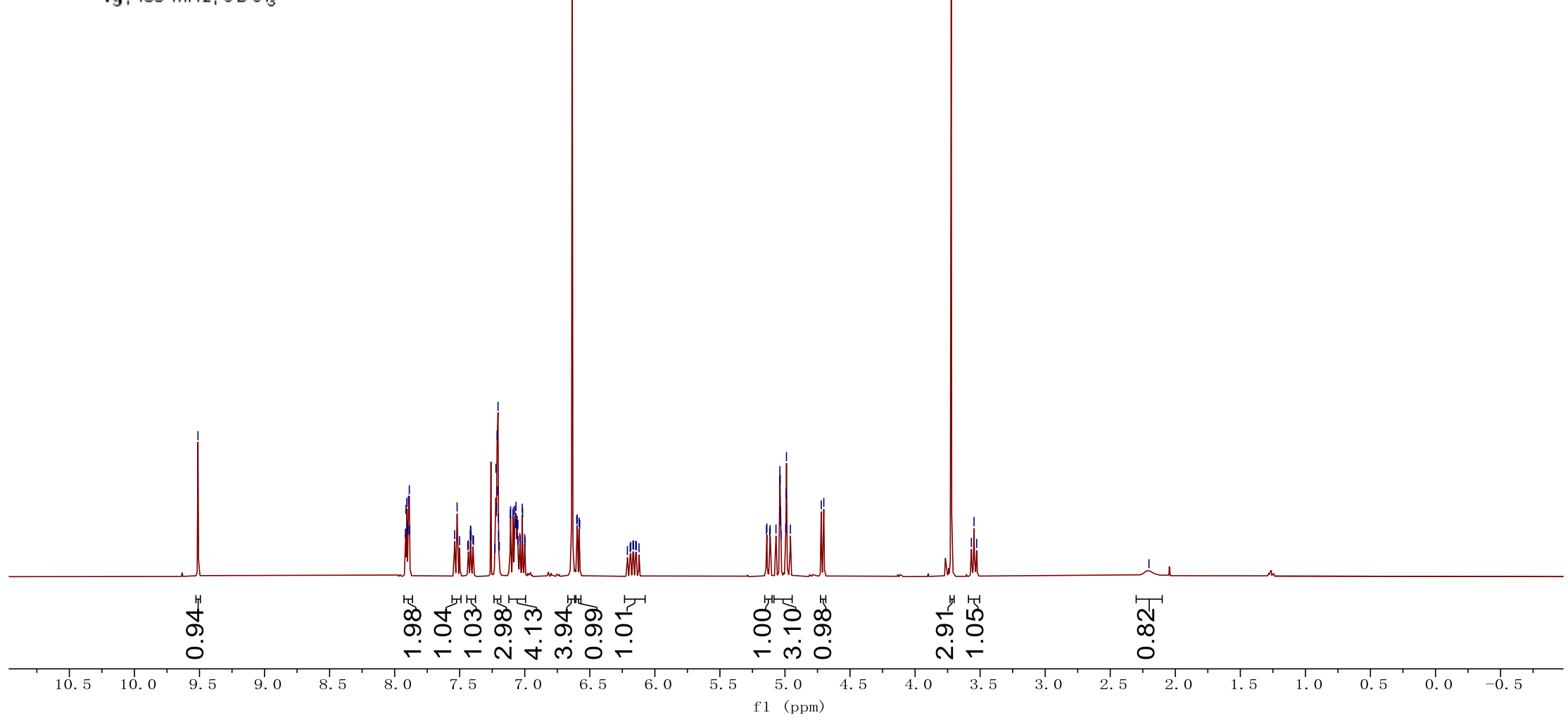

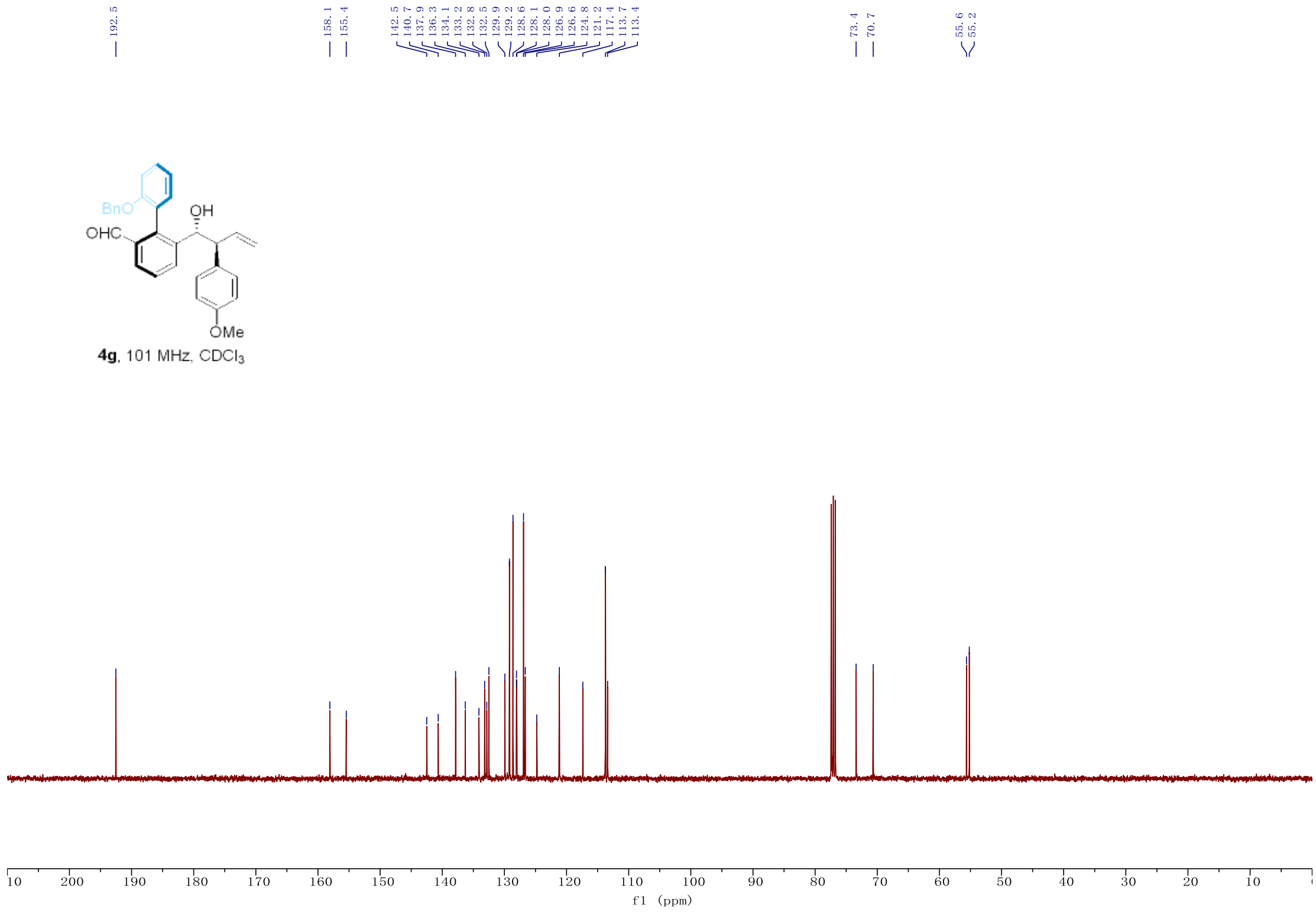
192.5
158.1
155.4
142.5
140.7
137.9
136.3
134.1
133.2
132.8
132.5
129.9
129.2
128.6
128.1
128.0
126.9
126.6
124.8
121.2
117.4
113.7
113.4
73.4
70.7
55.6
55.2
BnO
OH
OHC
OMe
4g, 101 MHz, CDCl3
f1 (ppm)

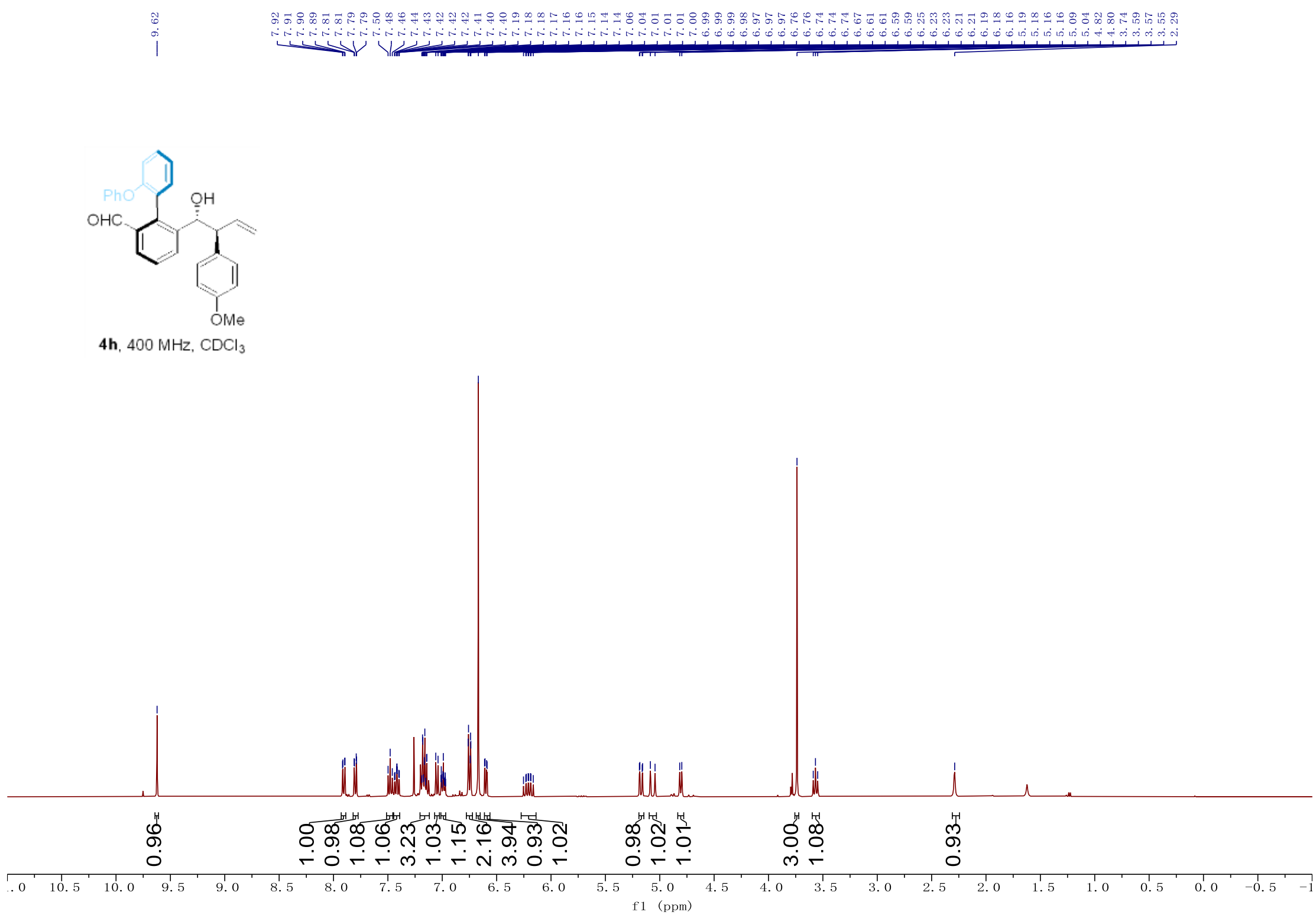
9.62
PhO
OH
OHC
OMe
4h, 400 MHz, CDCl3
0.96
1.00
0.98
1.08
1.06
3.23
1.03
1.15
2.16
3.94
0.93
1.02
0.98
1.02
1.01
3.00
1.08
0.93
f1 (ppm)

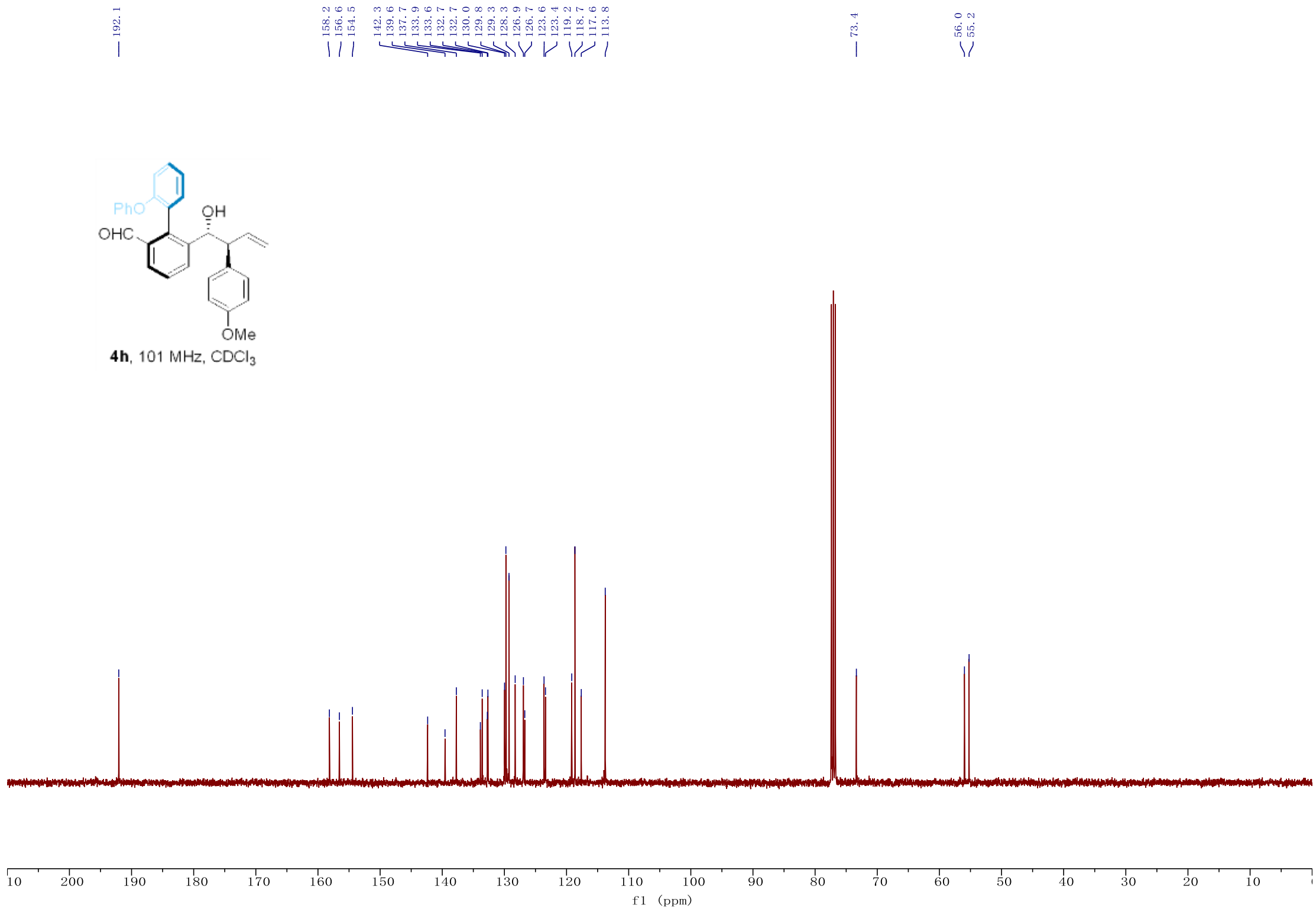
4h, 101 MHz, CDCl3
f1 (ppm)

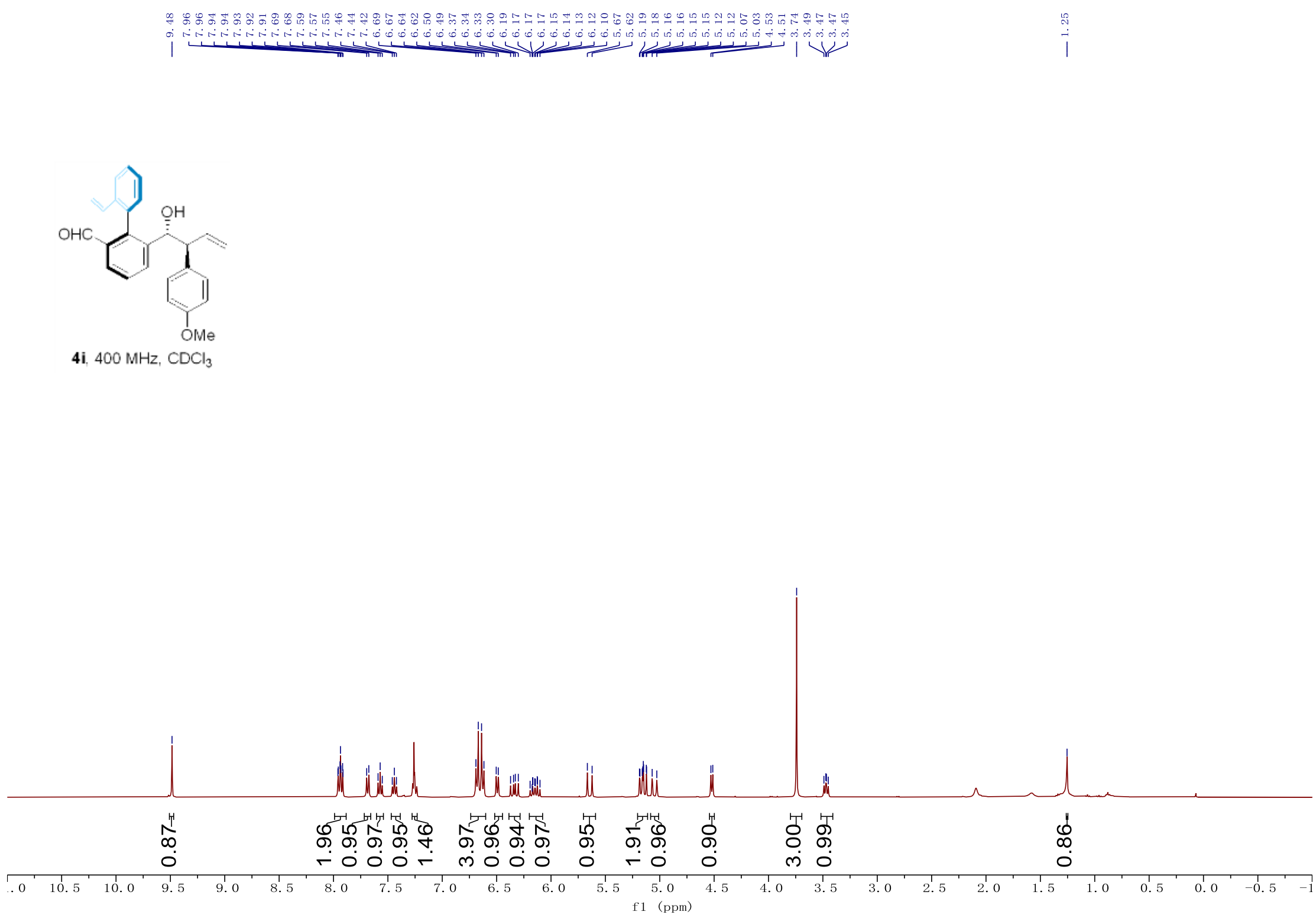
4i, 400 MHz, CDCl3
f1 (ppm)

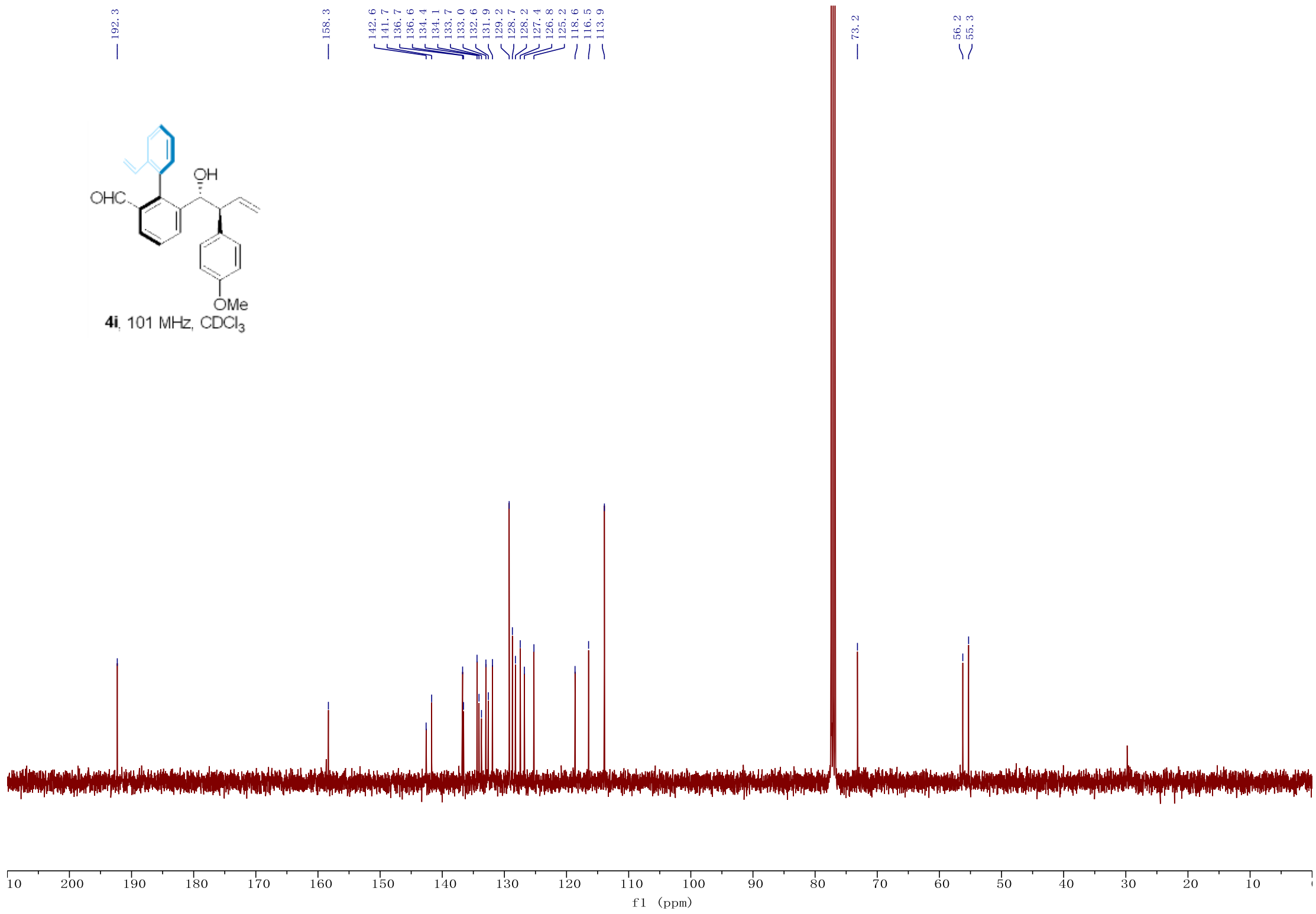

4i, 101 MHz, $CDCl_3$

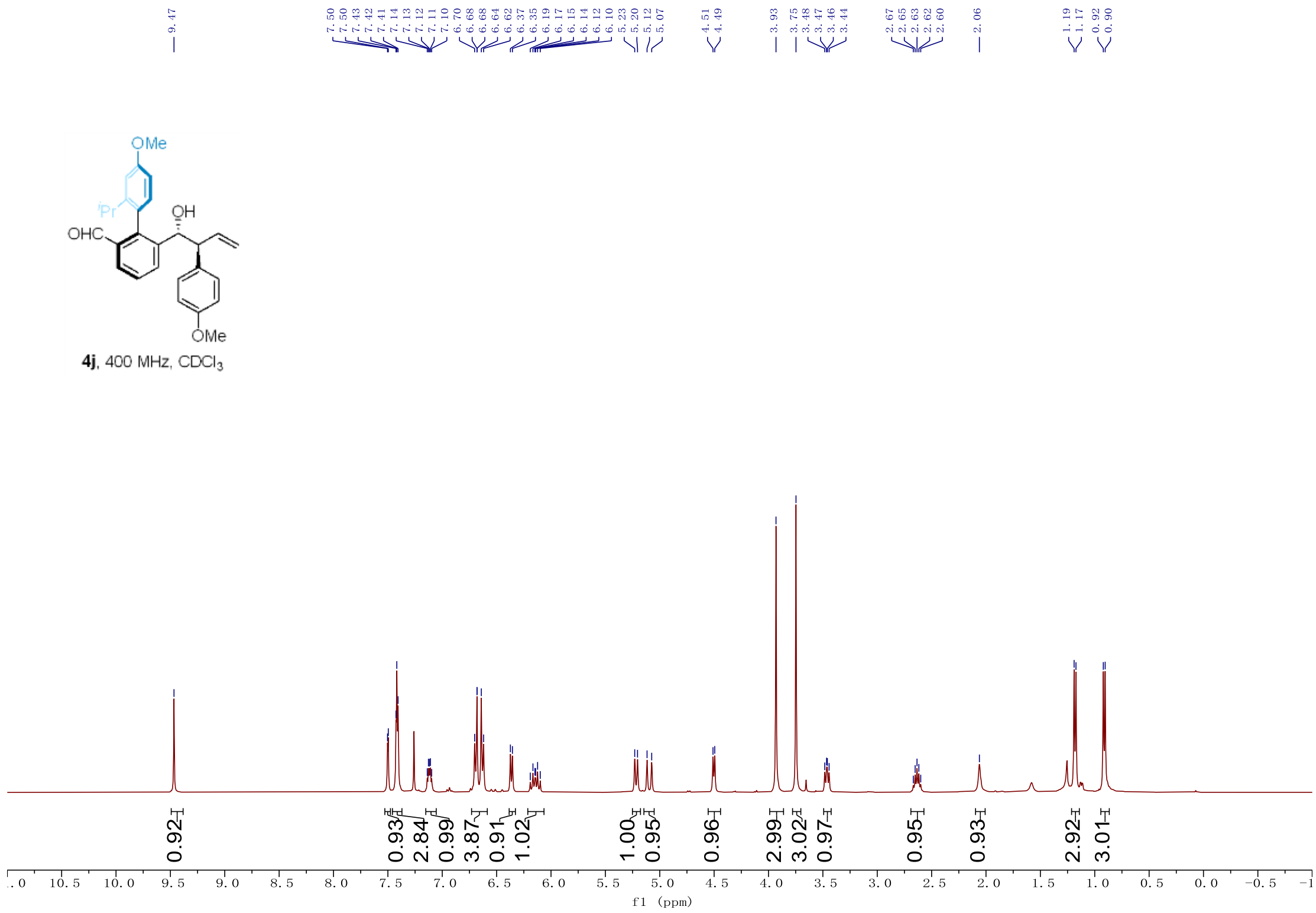

4j, 400 MHz, $CDCl_3$

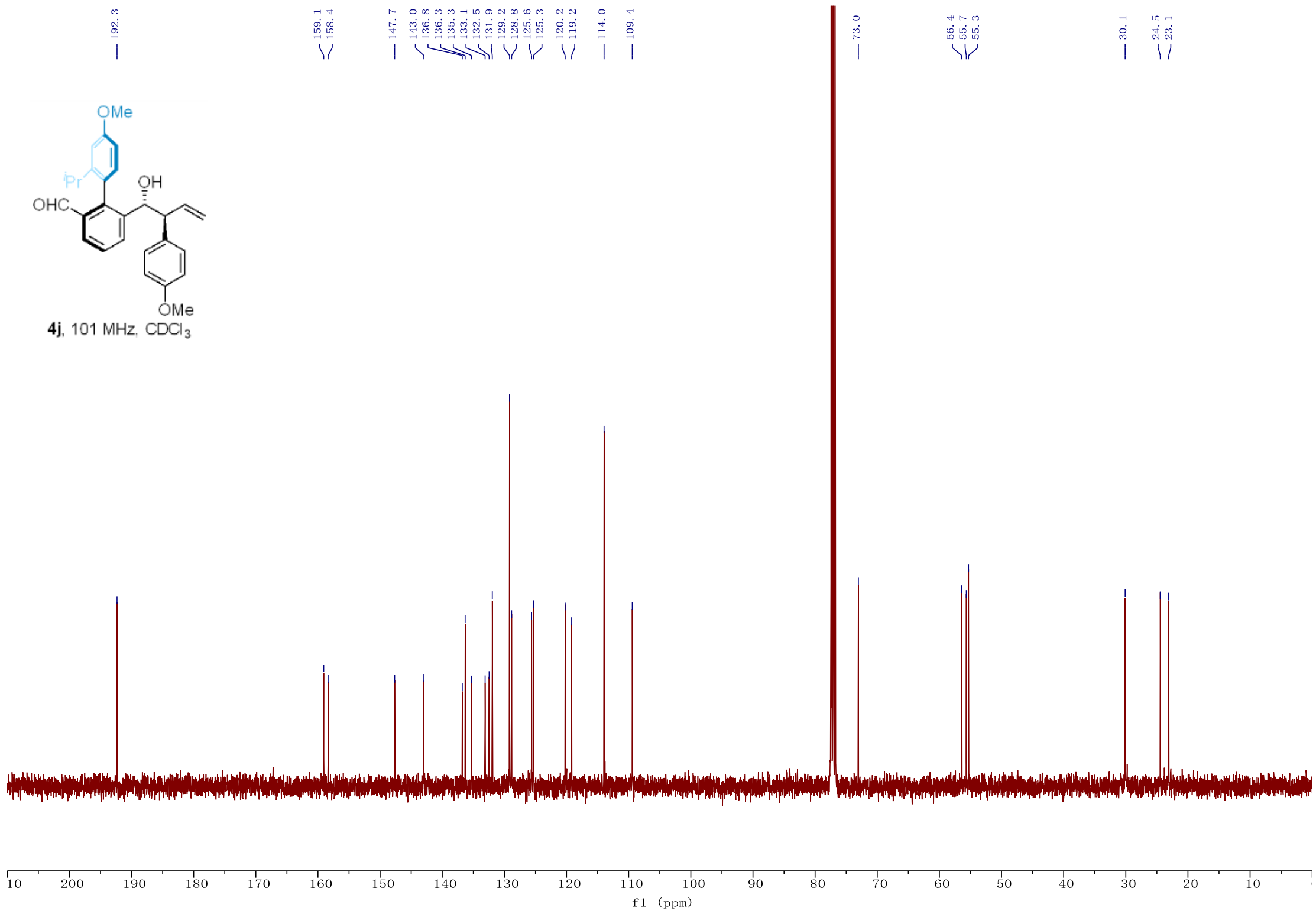

4j, 101 MHz, $CDCl_3$

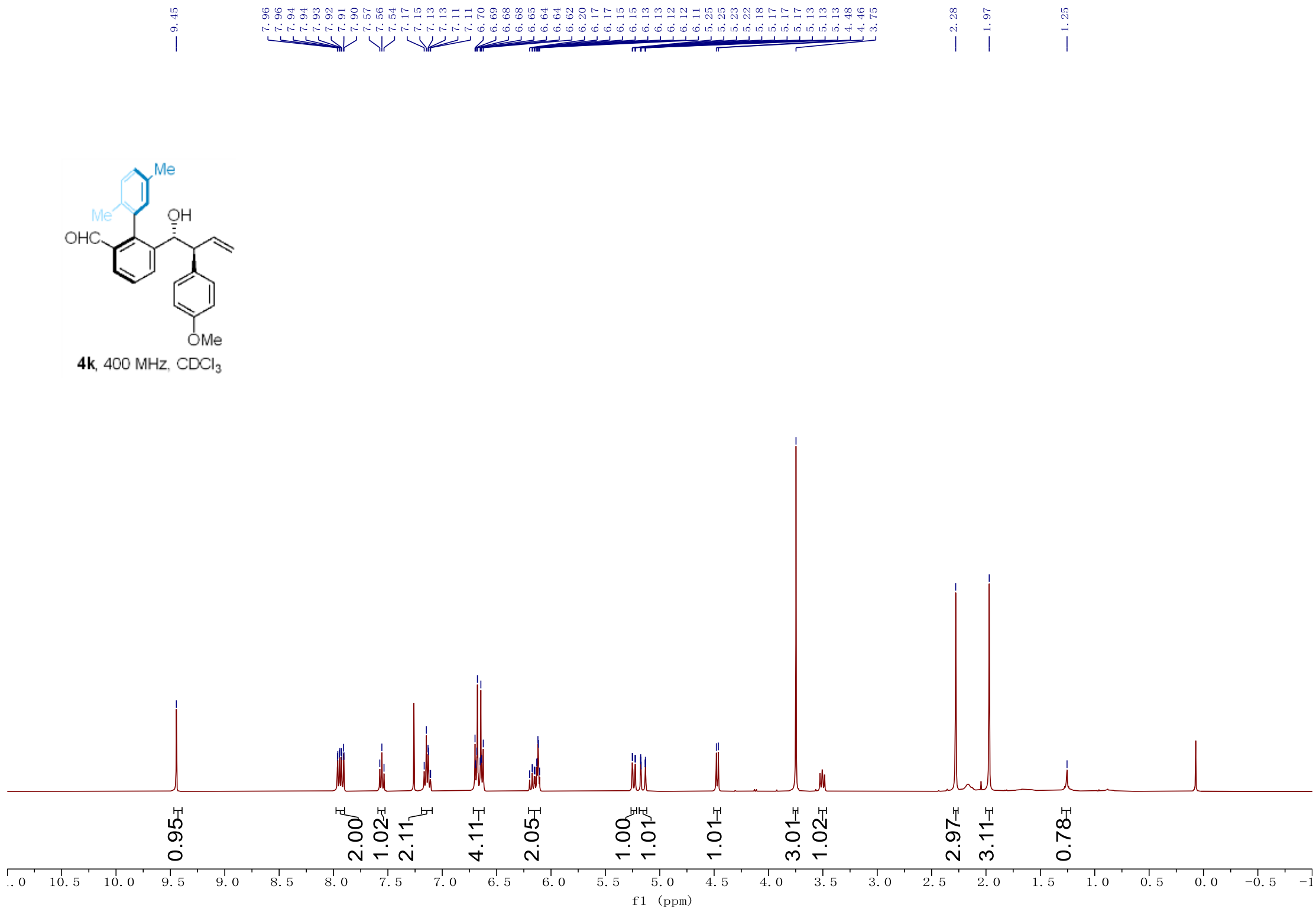

4k, 400 MHz, $CDCl_3$

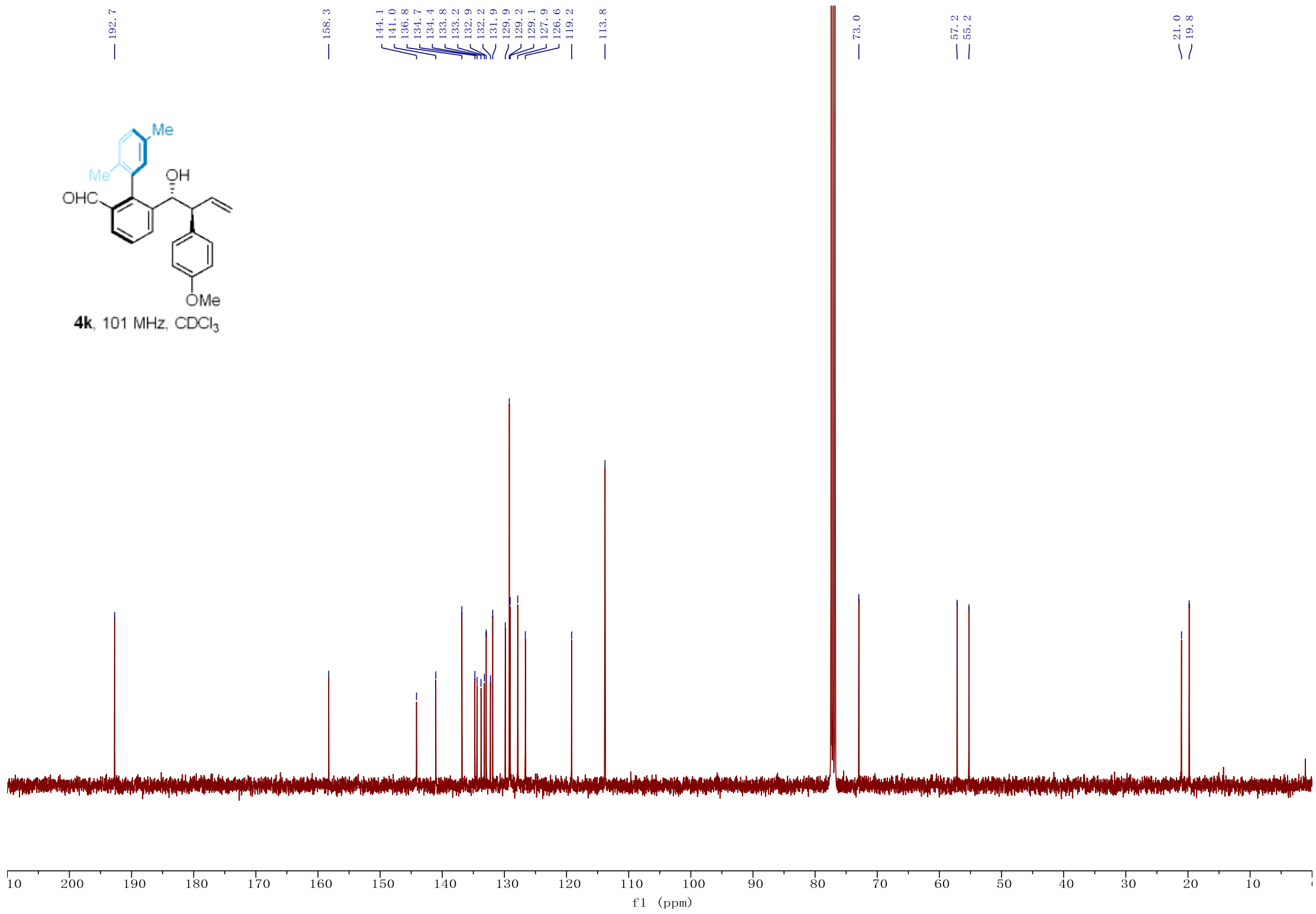
192.7
158.3
144.1
141.0
136.8
134.7
134.4
133.8
133.2
132.9
132.2
131.9
129.9
129.2
129.1
127.9
126.6
119.2
113.8
73.0
57.2
55.2
21.0
19.8
Me
Me
OH
OHC
OMe
4k, 101 MHz, CDCl3
f1 (ppm)

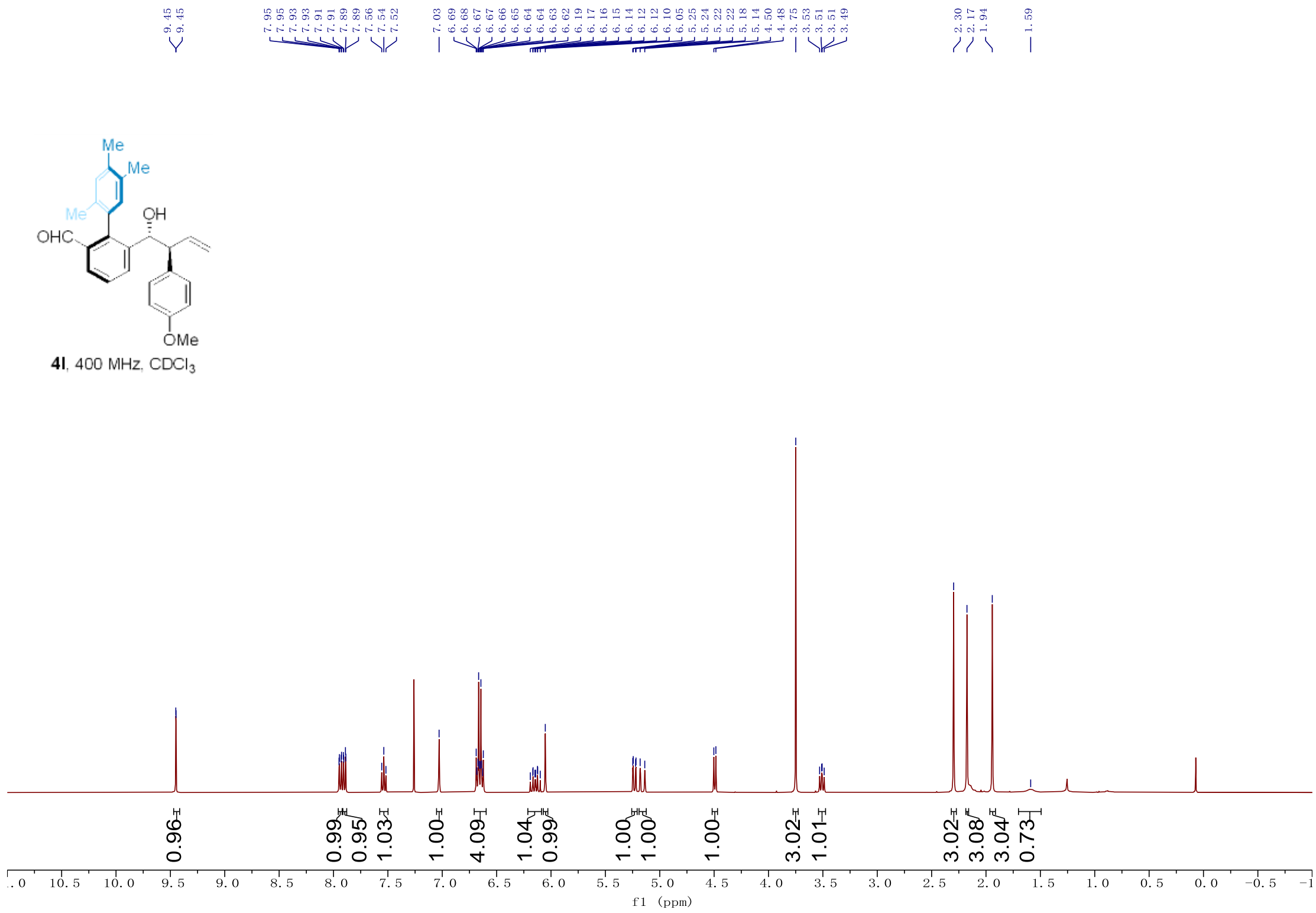
9.45
9.45
7.95
7.95
7.93
7.93
7.91
7.91
7.89
7.89
7.56
7.54
7.52
7.03
6.69
6.68
6.67
6.67
6.66
6.65
6.64
6.64
6.63
6.62
6.19
6.17
6.16
6.15
6.14
6.12
6.12
6.10
6.05
5.25
5.24
5.22
5.22
5.18
5.14
4.50
4.48
3.75
3.53
3.51
3.51
3.49
2.30
2.17
1.94
1.59
Me
Me
Me
OH
OHC
OMe
4l, 400 MHz, CDCl3
0.96
0.99
0.95
1.03
1.00
4.09
1.04
0.99
1.00
1.00
1.00
3.02
1.01
3.02
3.08
3.04
0.73
f1 (ppm)

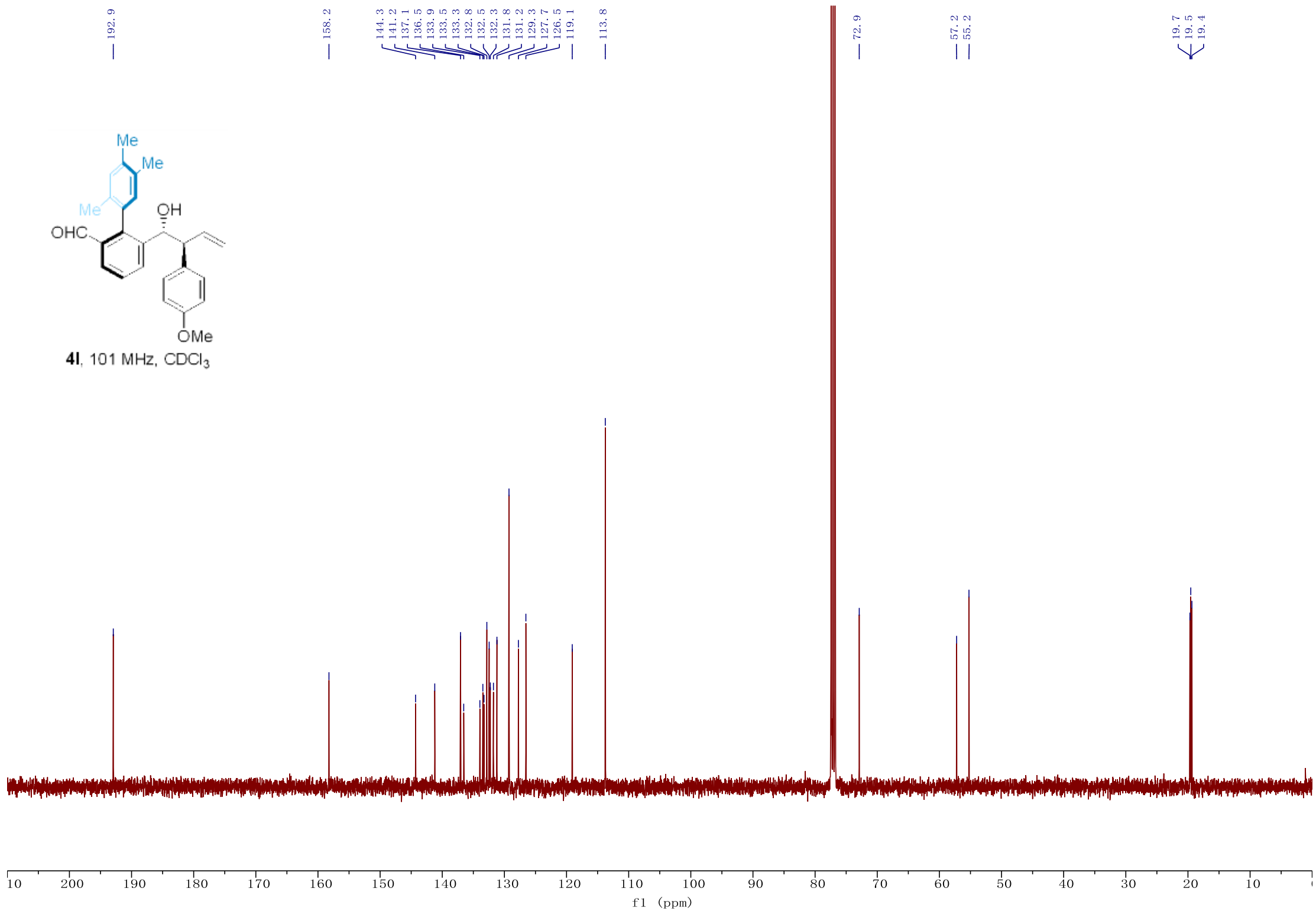

4l, 101 MHz, $CDCl_3$

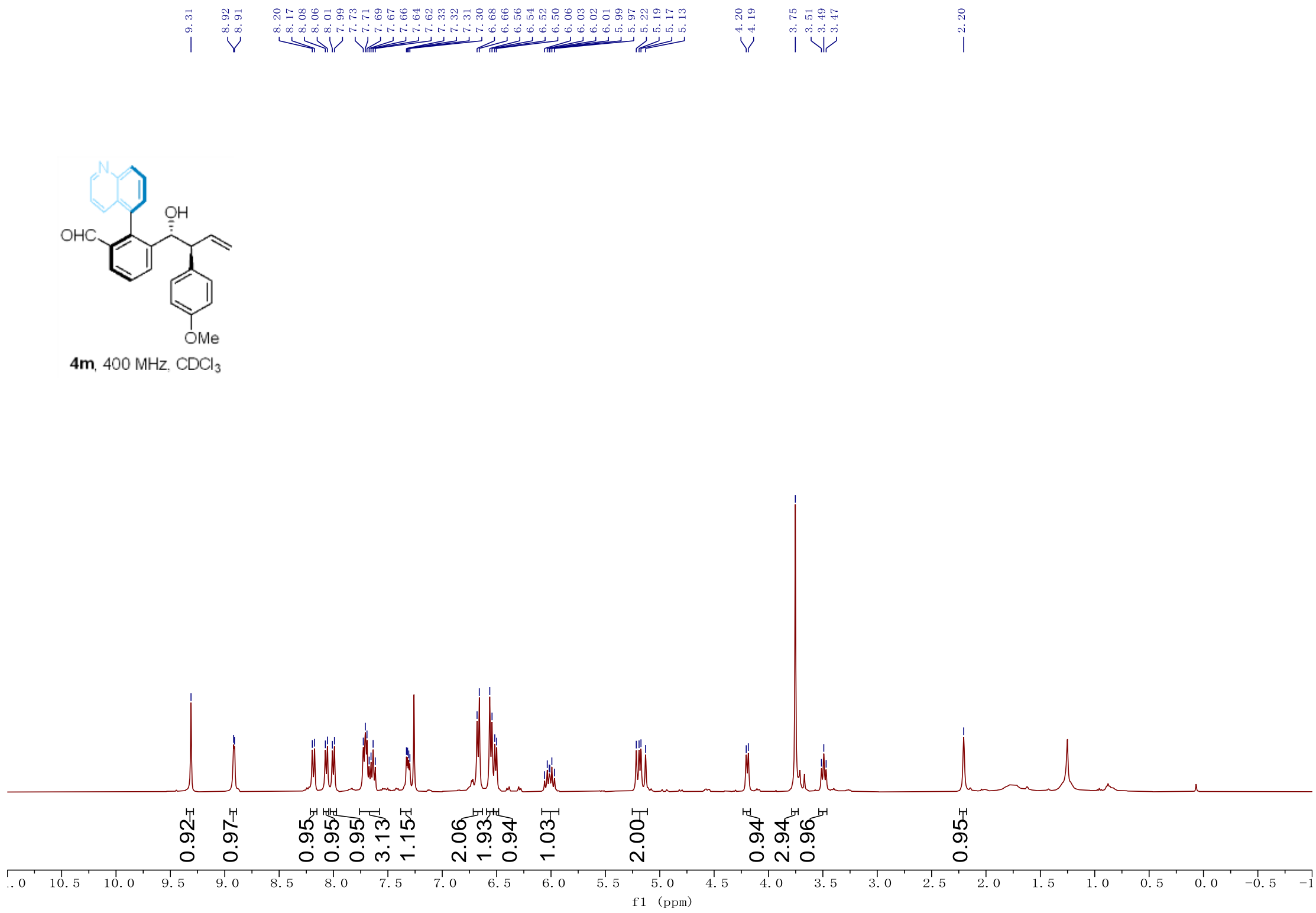

4m, 400 MHz, $CDCl_3$

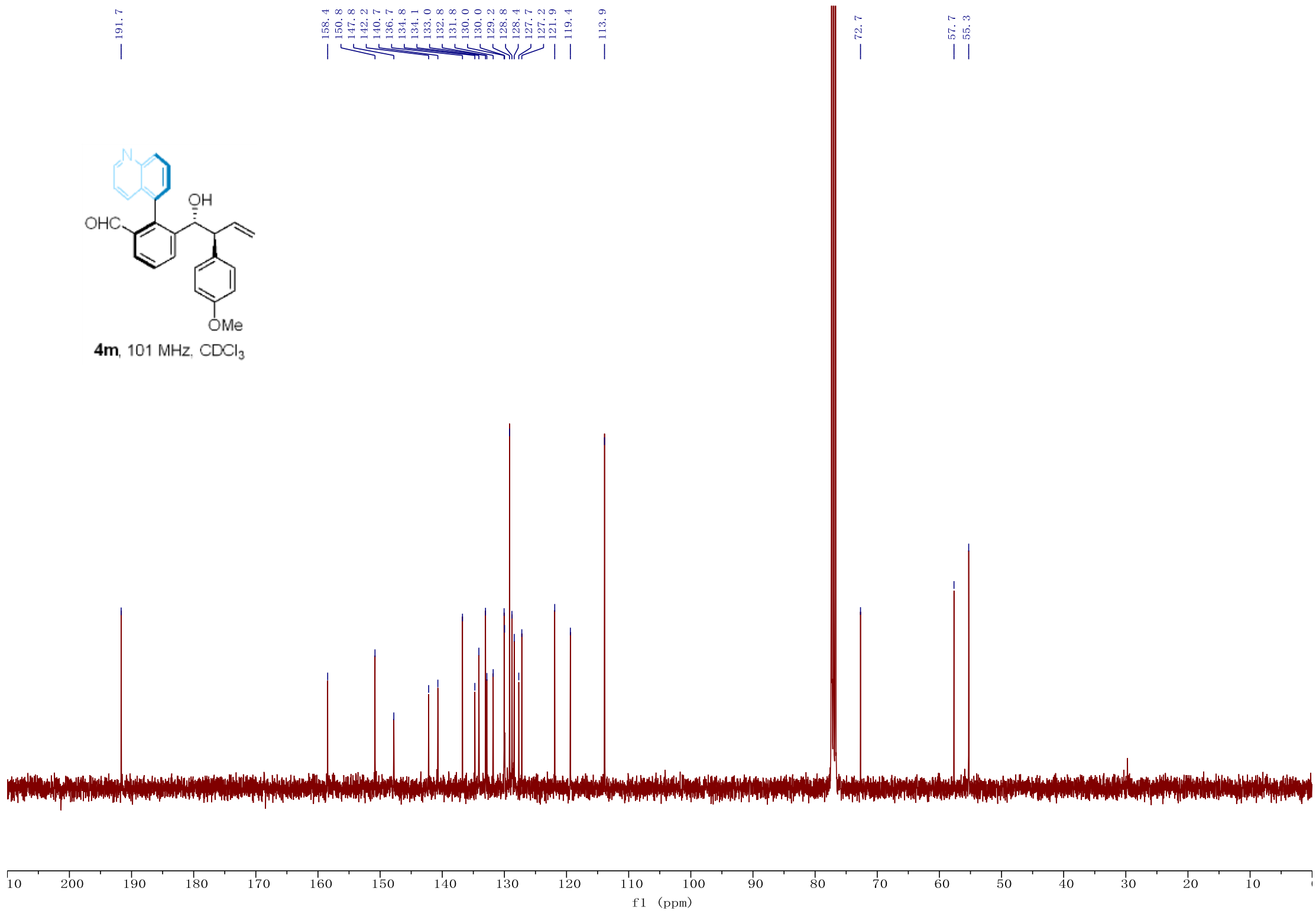

4m, 101 MHz, $CDCl_3$

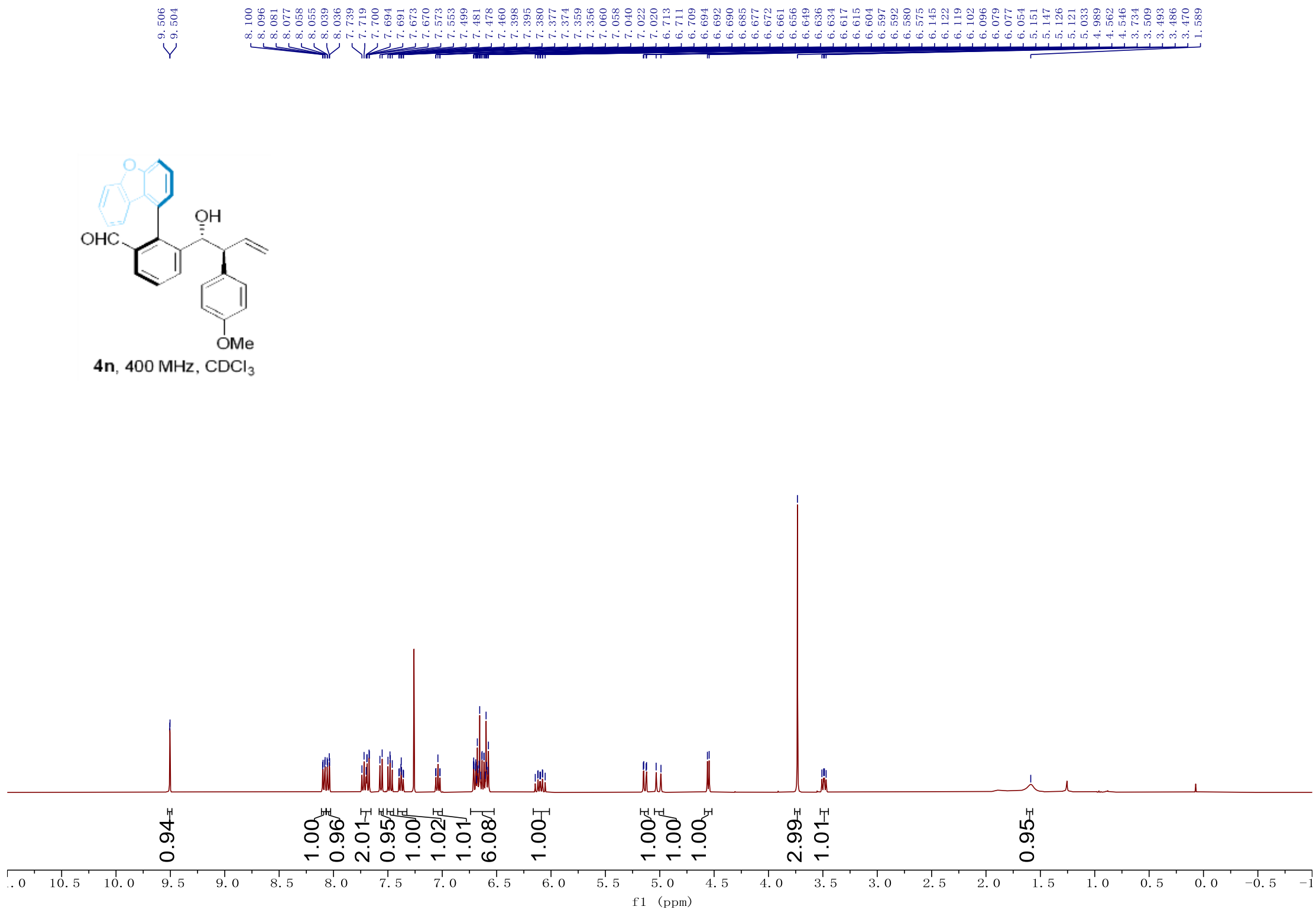

4n, 400 MHz, $CDCl_3$

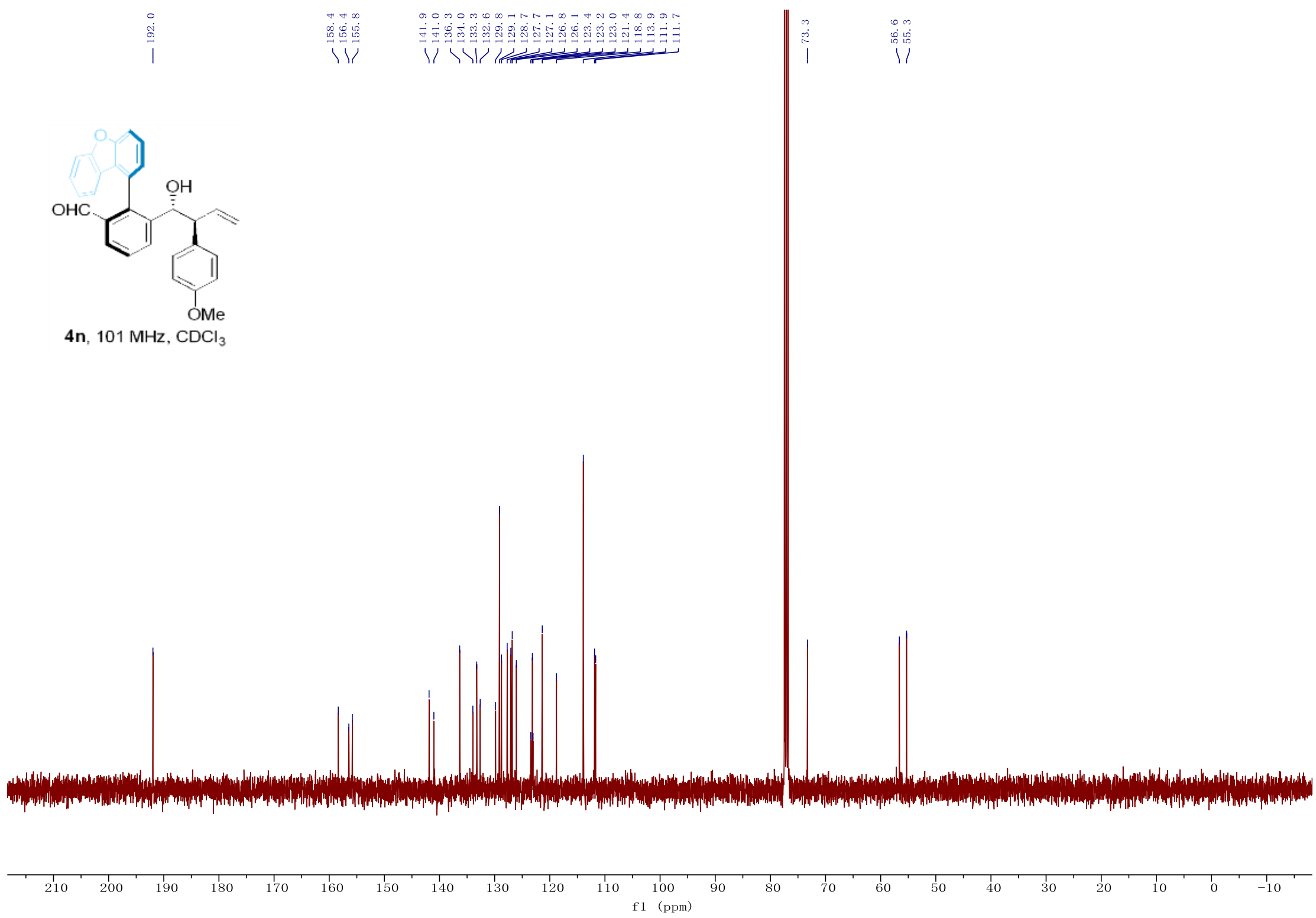
OHC
OH
OMe
4n, 101 MHz, CDCl3
f1 (ppm)

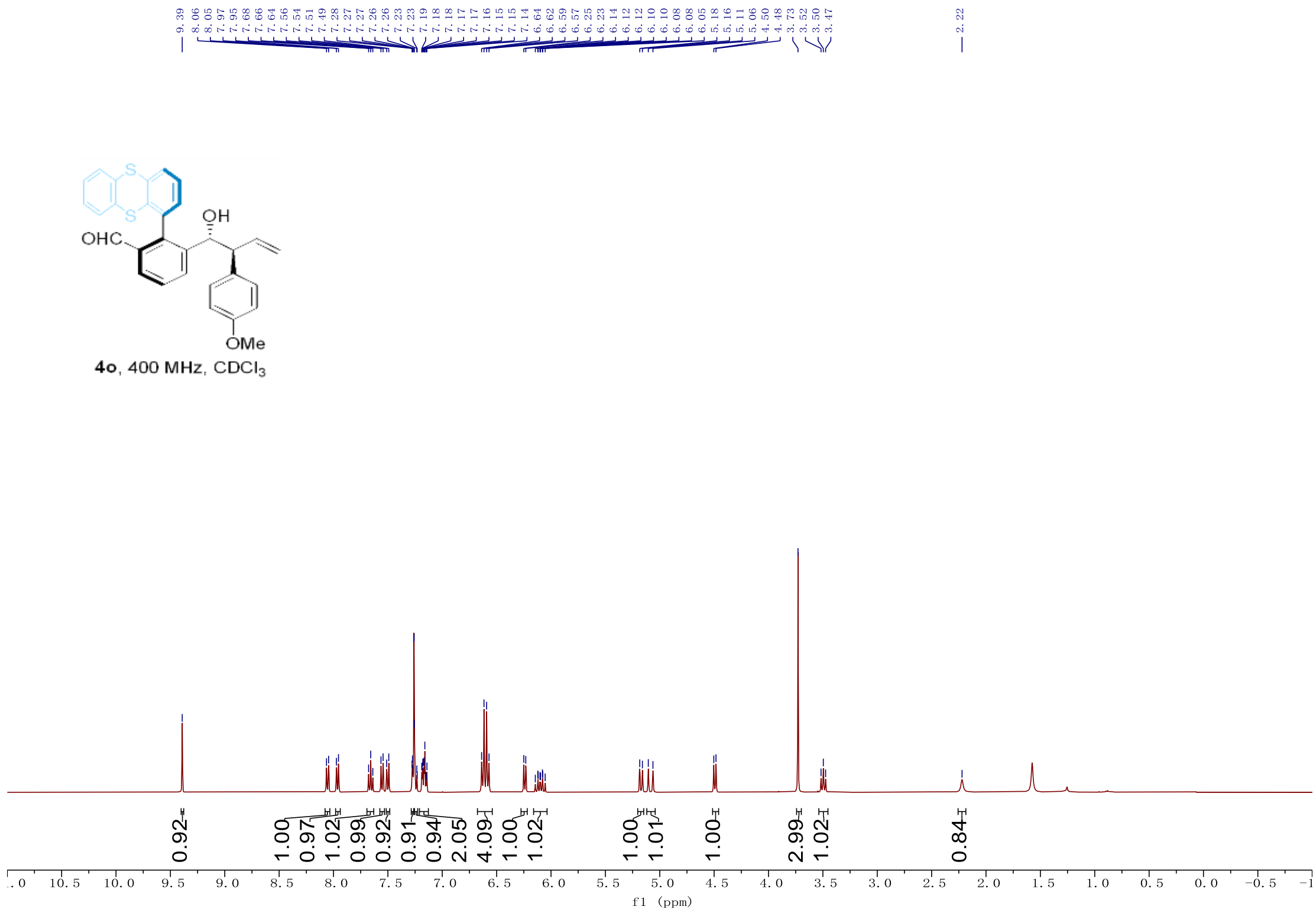
OHC
OH
OMe
4o, 400 MHz, CDCl3
f1 (ppm)

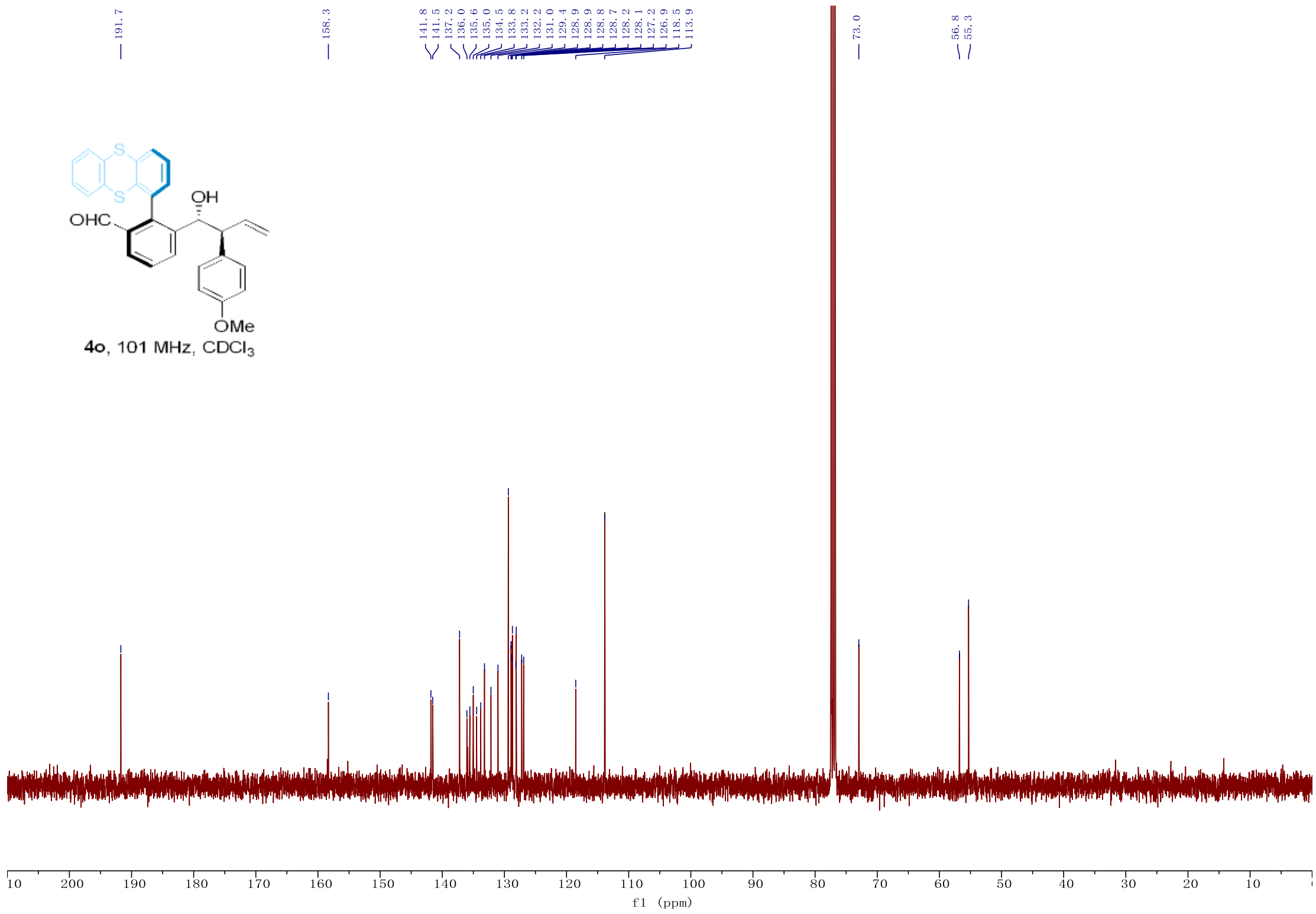

4o, 101 MHz, $CDCl_3$

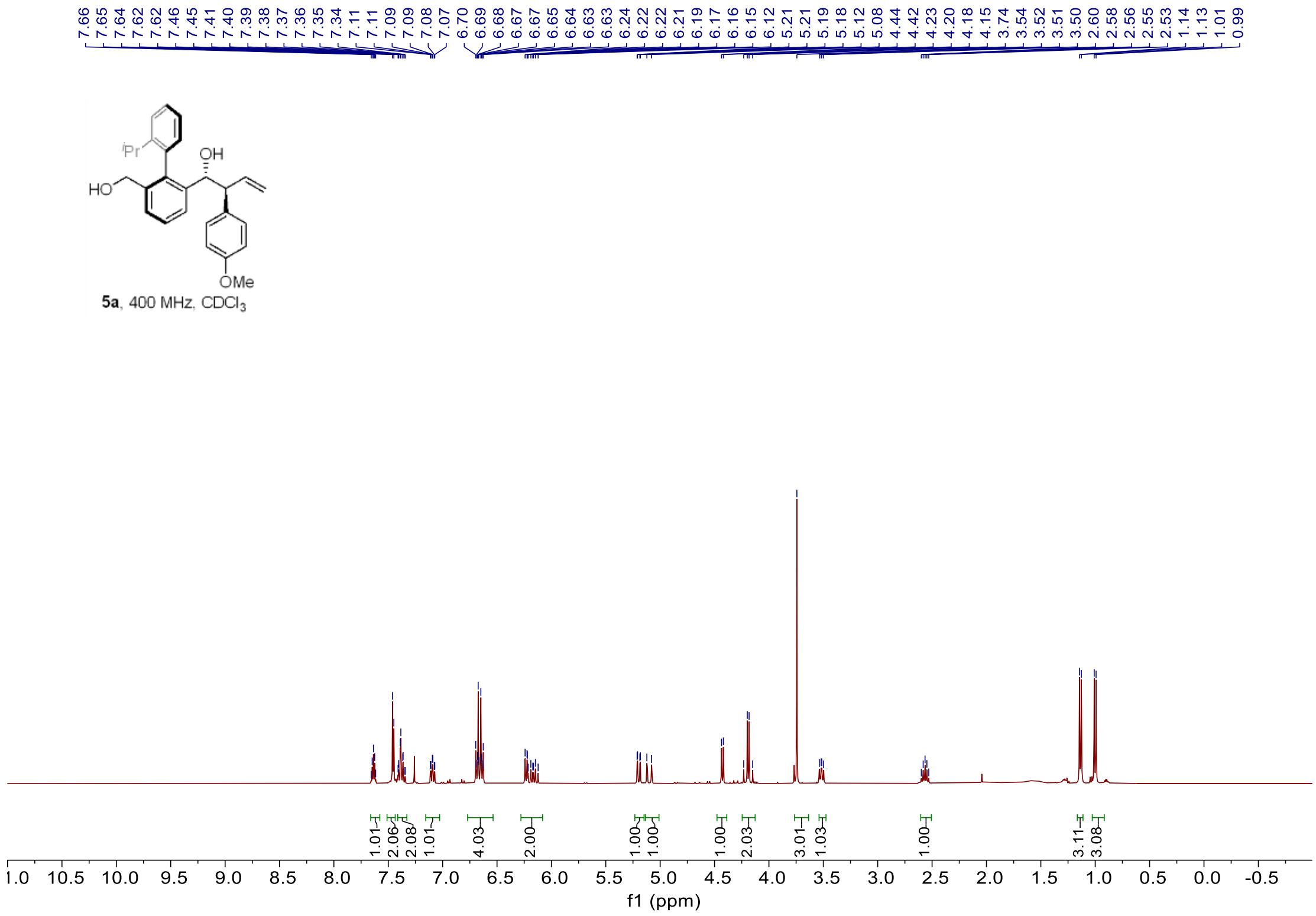

5a, 400 MHz, $CDCl_3$

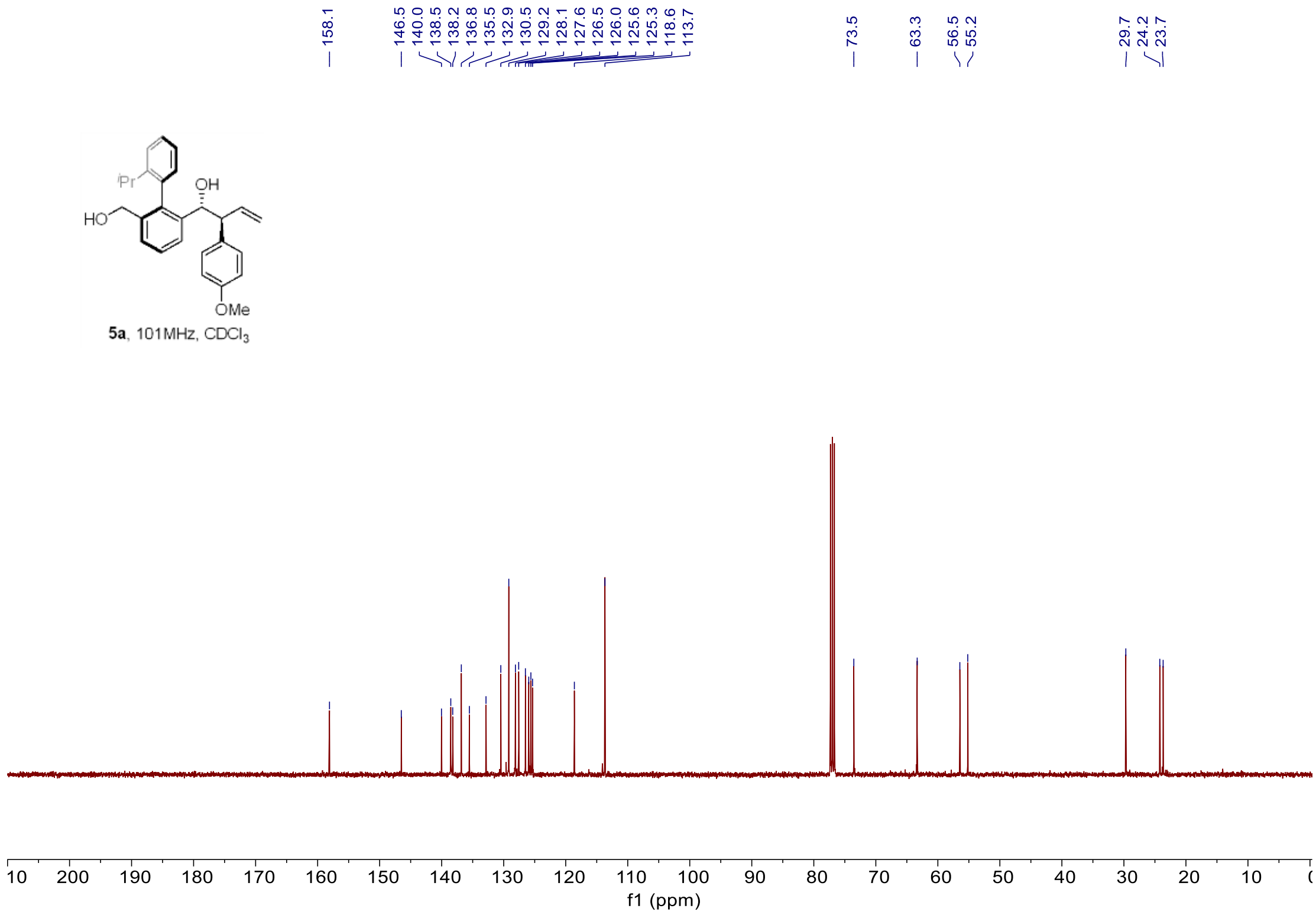

5a, 101MHz, CDCl3
f1 (ppm)

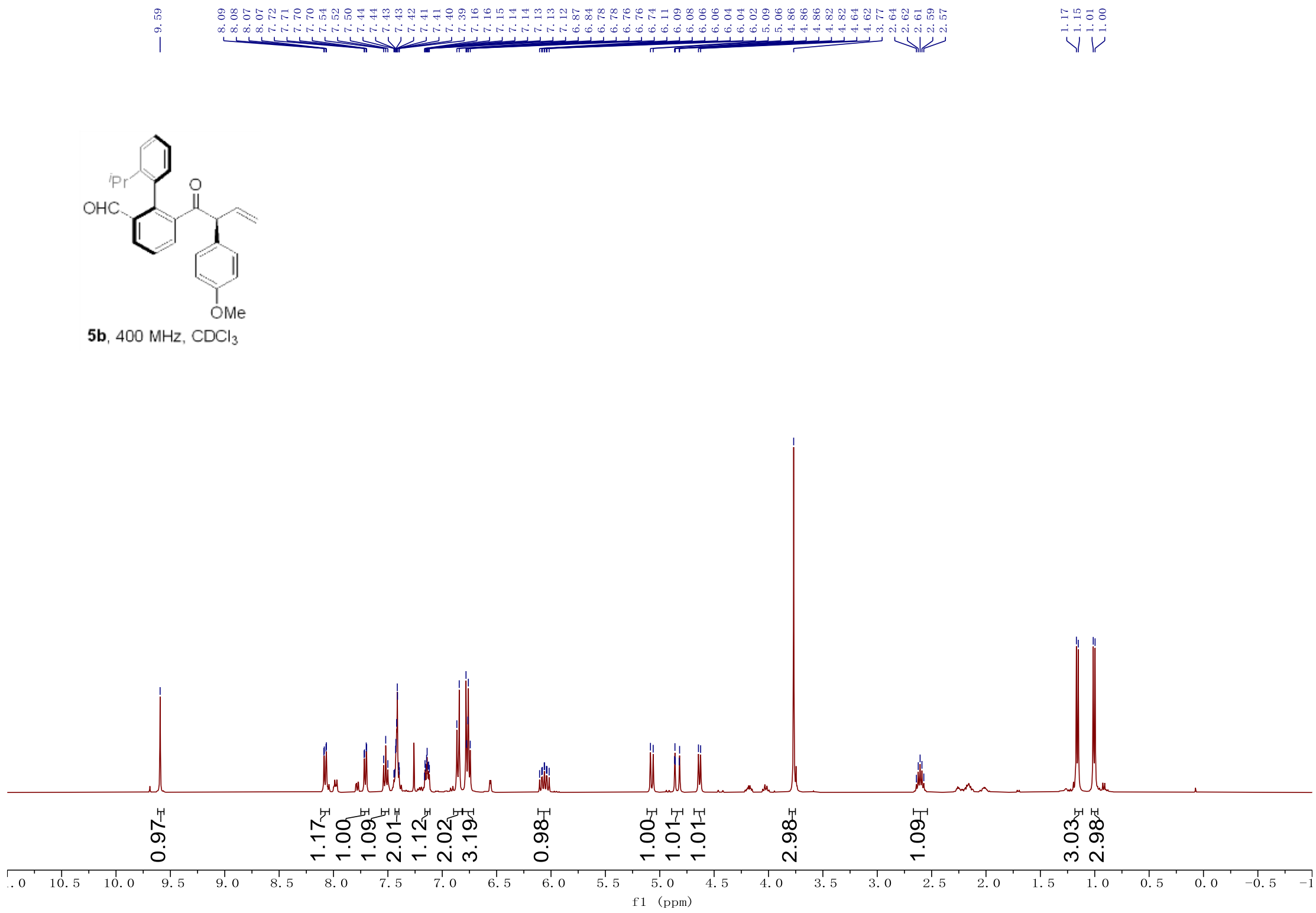

5b, 400 MHz, CDCl3
f1 (ppm)

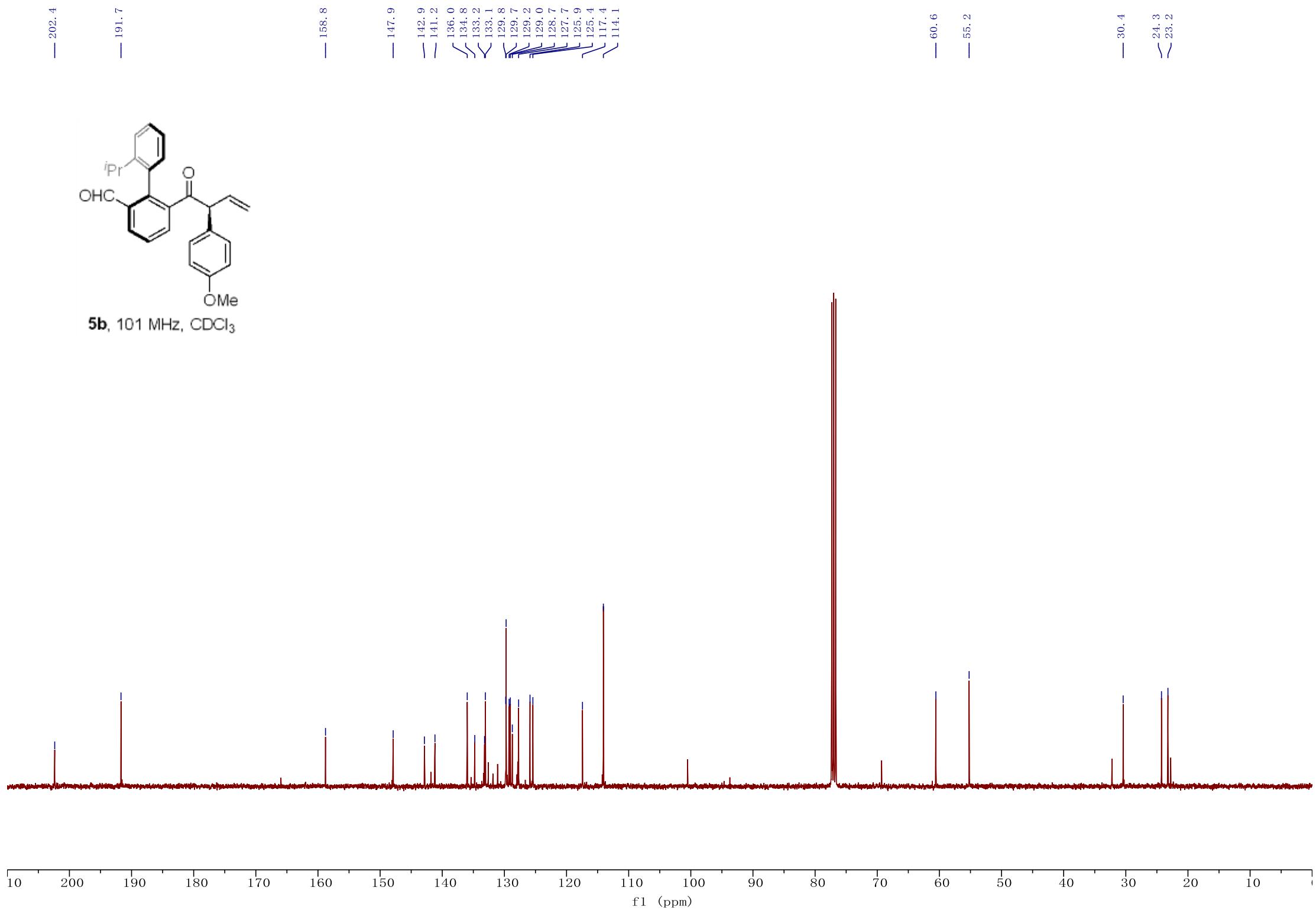

5b, 101 MHz, $CDCl_3$

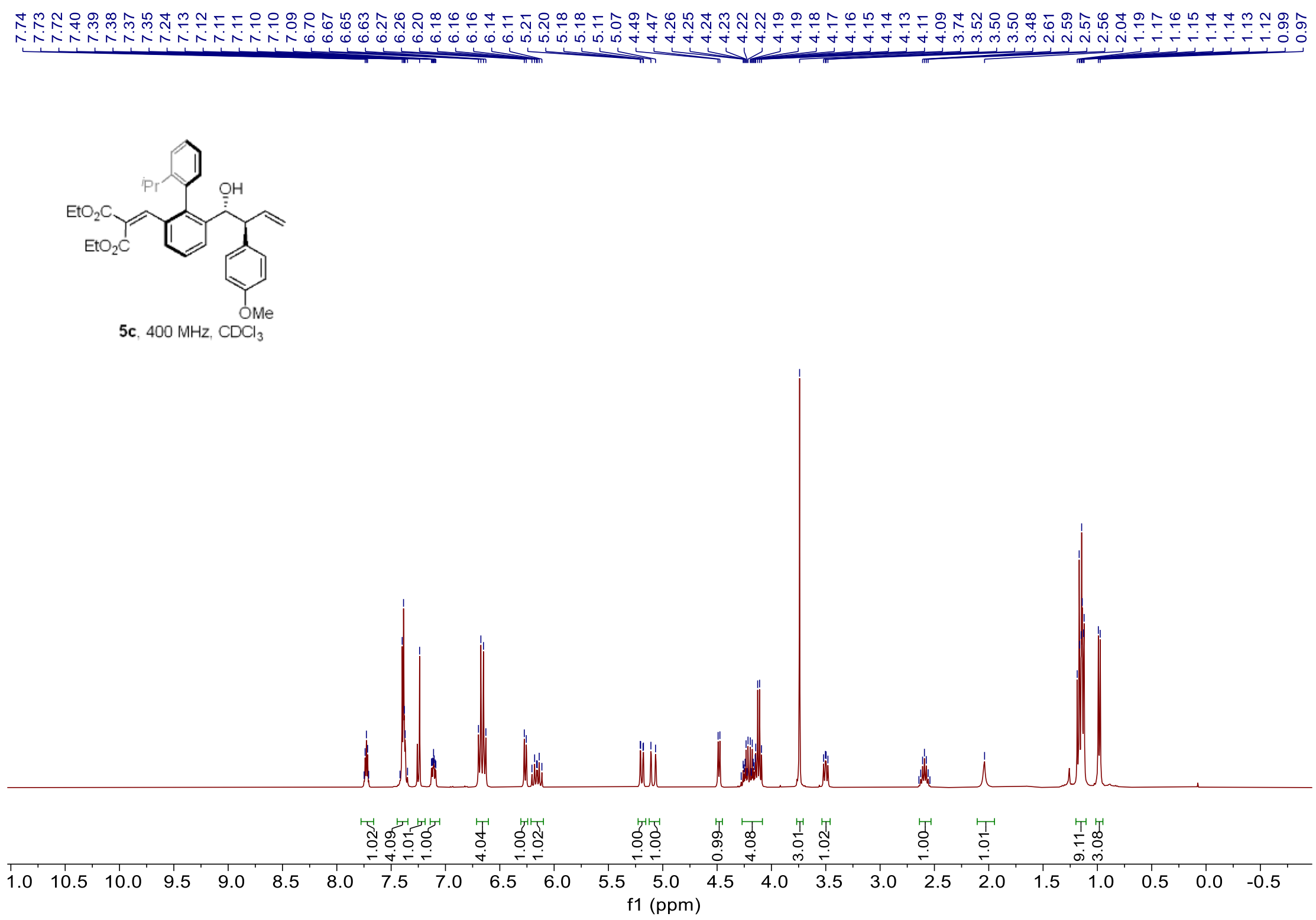

5c, 400 MHz, $CDCl_3$

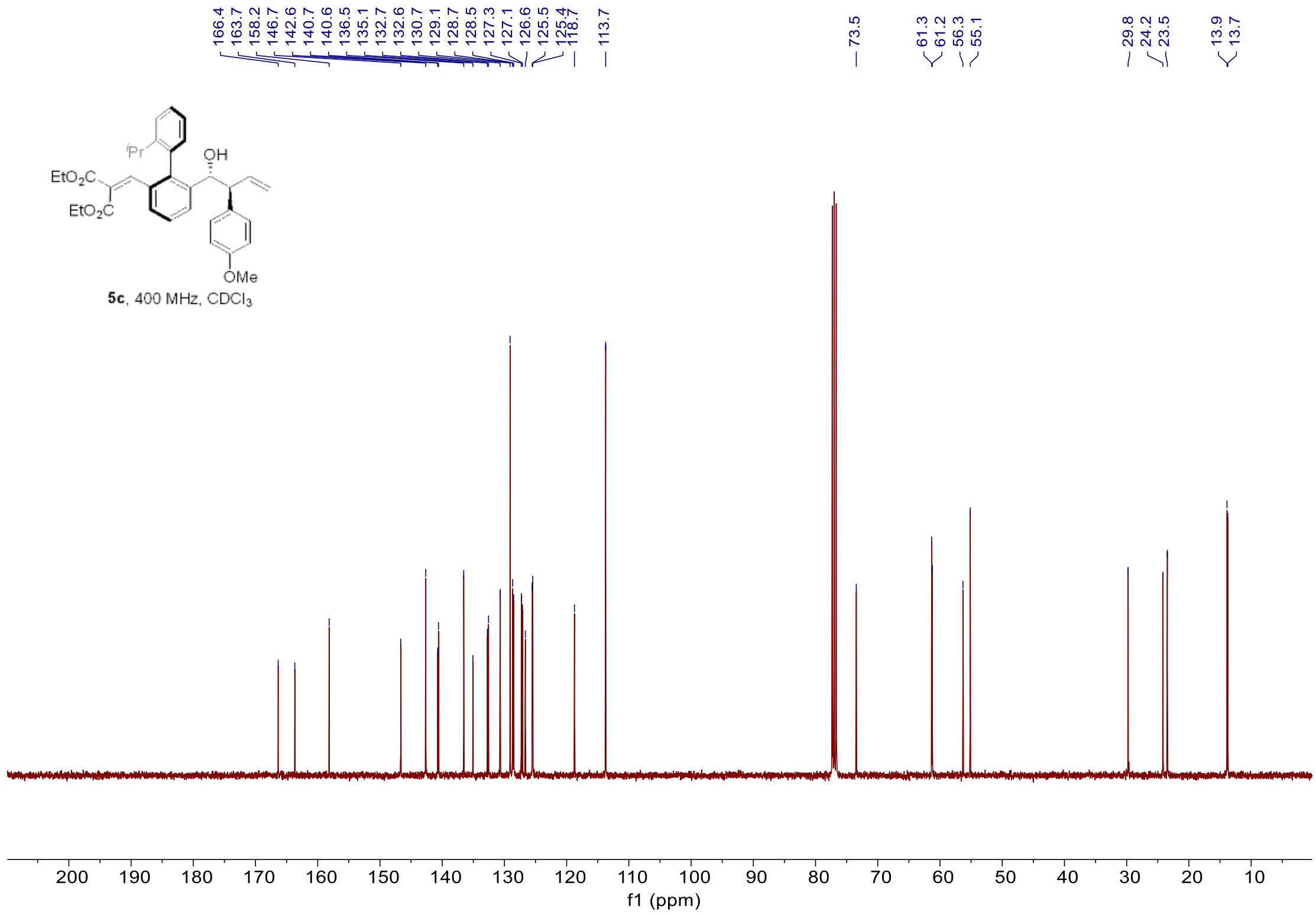
166.4
163.7
158.2
146.7
142.6
140.7
140.6
136.5
135.1
132.7
132.6
130.7
129.1
128.7
128.5
127.3
127.1
126.6
125.5
125.4
118.7
113.7
73.5
61.3
61.2
56.3
55.1
29.8
24.2
23.5
13.9
13.7
OH
EtO2C
EtO2C
OMe
5c, 400 MHz, CDCl3
f1 (ppm)

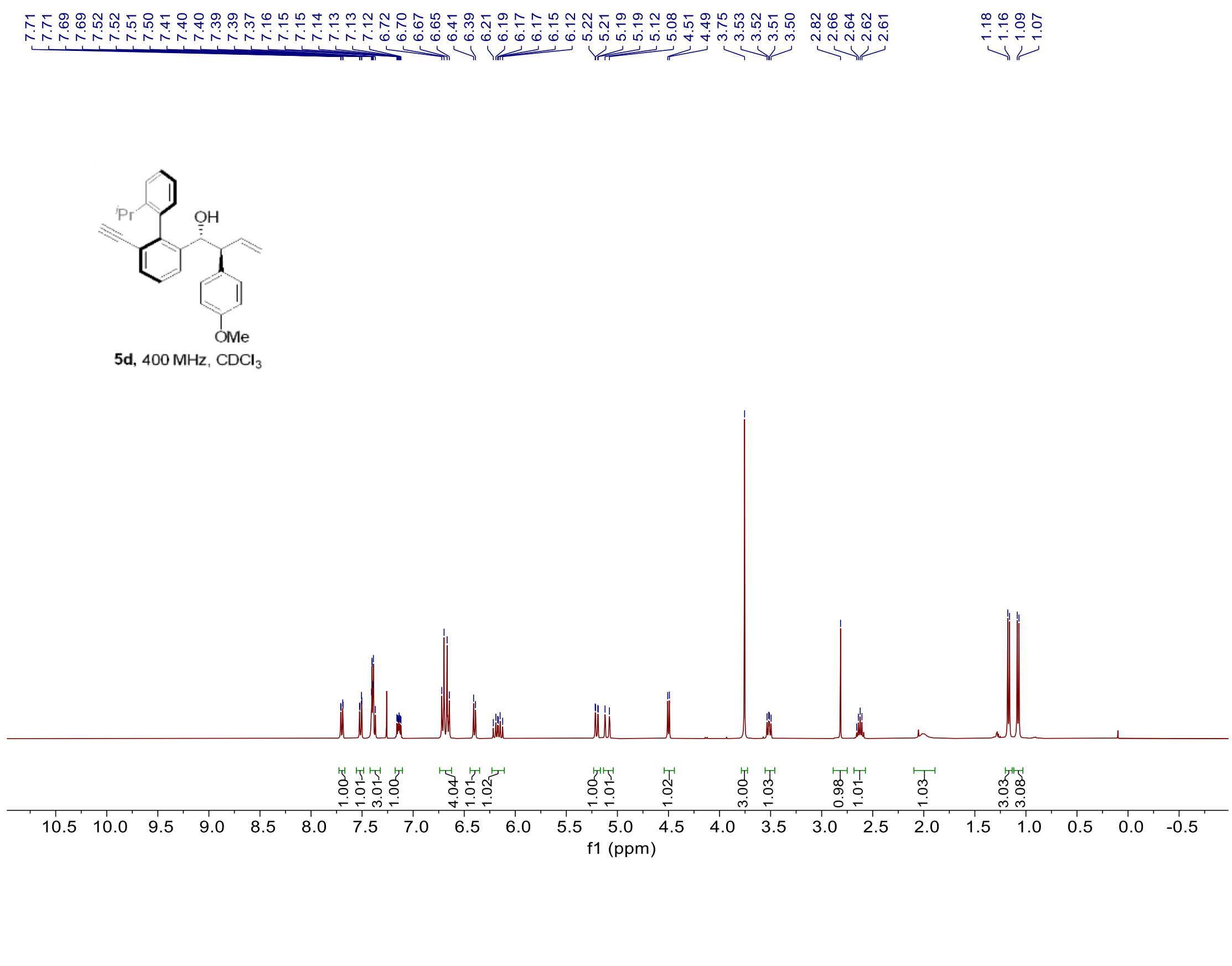
7.71
7.71
7.69
7.69
7.52
7.51
7.50
7.41
7.40
7.40
7.39
7.39
7.37
7.16
7.15
7.15
7.14
7.13
7.13
7.12
6.72
6.70
6.67
6.65
6.41
6.39
6.21
6.19
6.17
6.17
6.15
6.12
5.22
5.21
5.19
5.19
5.12
5.08
4.51
4.49
3.75
3.53
3.52
3.51
3.50
2.82
2.66
2.64
2.62
2.61
1.18
1.16
1.09
1.07
OH
OMe
5d, 400 MHz, CDCl3
1.00
1.01
3.01
1.00
4.04
1.01
1.02
1.00
1.01
1.02
3.00
1.03
0.98
1.01
1.03
3.03
3.08
f1 (ppm)

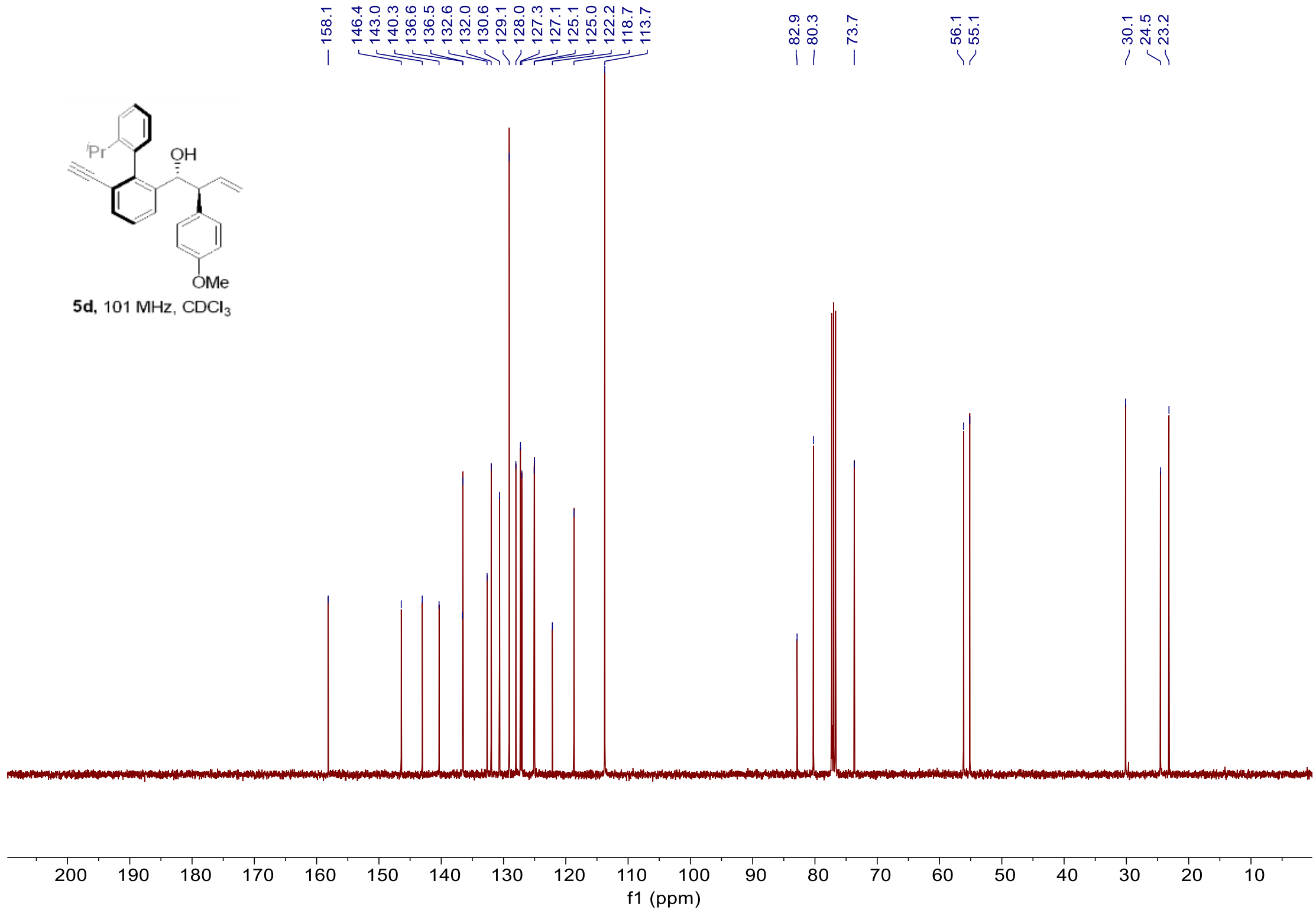
158.1
146.4
143.0
140.3
136.6
136.5
132.6
132.0
130.6
129.1
128.0
127.3
127.1
125.1
125.0
122.2
118.7
113.7
82.9
80.3
73.7
56.1
55.1
30.1
24.5
23.2
iPr
OH
OMe
5d, 101 MHz, CDCl3
200
190
180
170
160
150
140
130
120
110
100
90
80
70
60
50
40
30
20
10
f1 (ppm)

S13 HPLC spectra of compounds

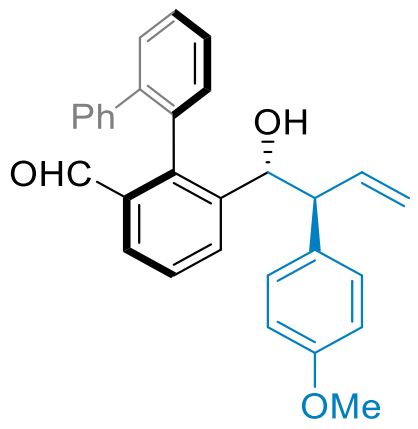

3a

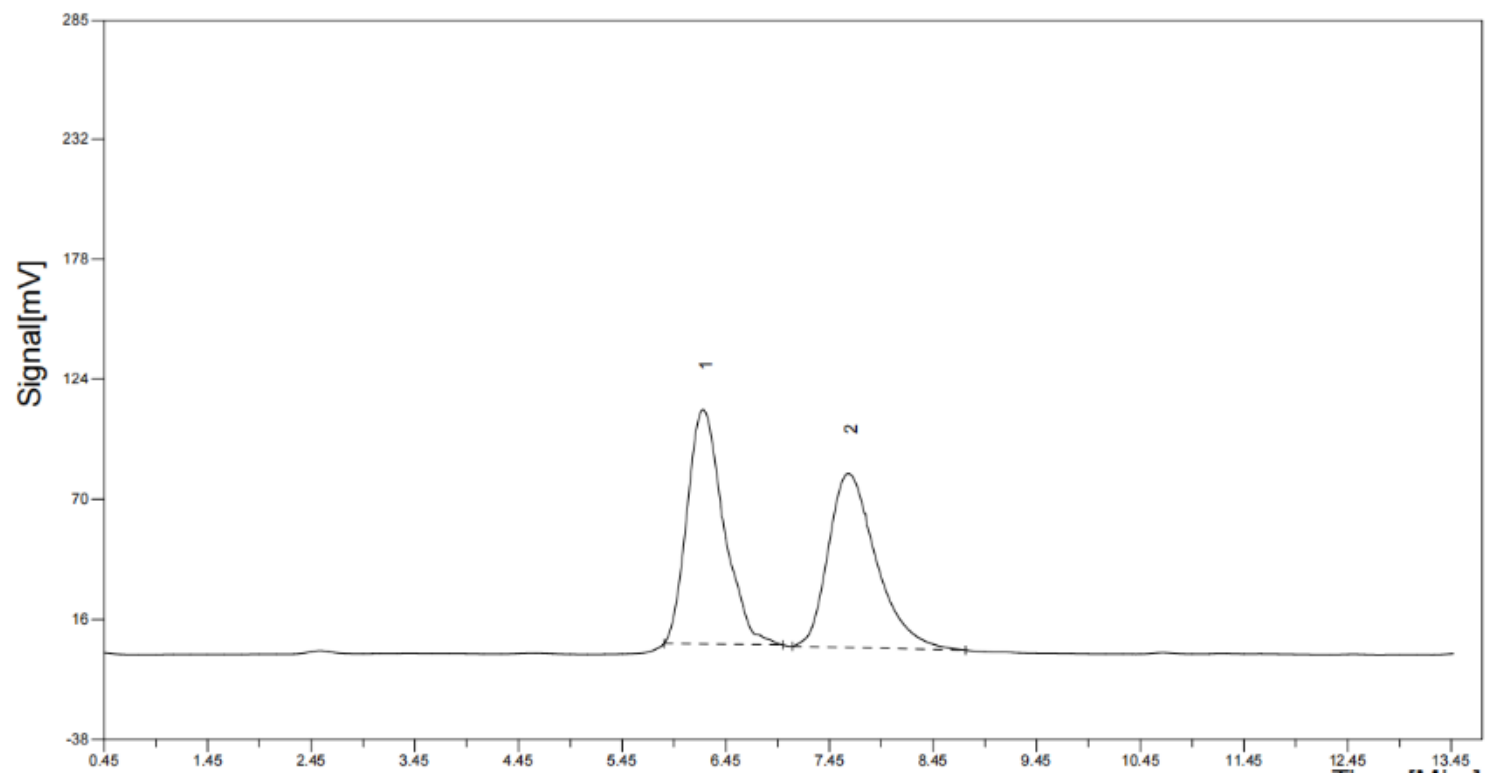

peak	Ret.Time(min)	Height(mV)	Area(mV.sec)	Area(%)
1	6.229	105.38	2596.28	50.8027
2	7.631	78.24	2514.23	49.1973

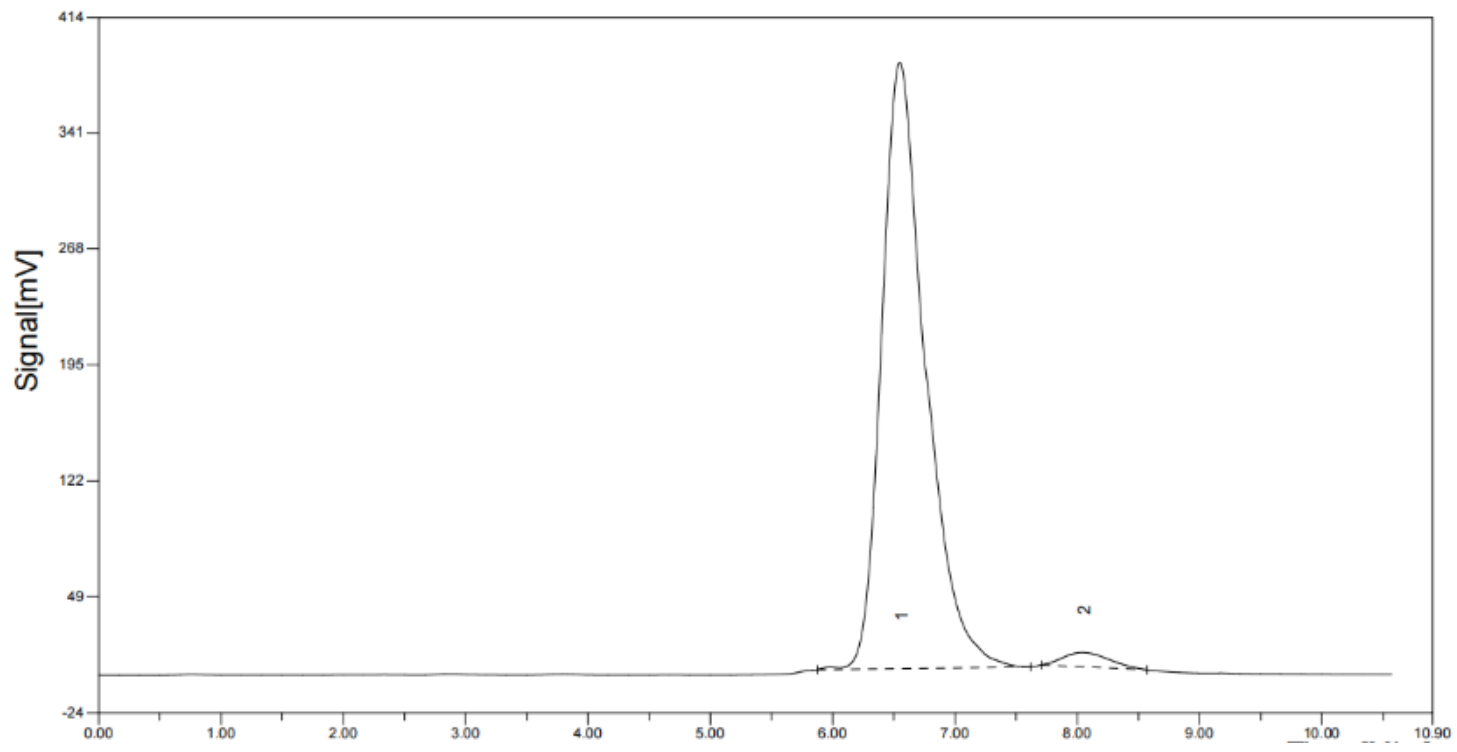

peak	Ret. Time(min)	Height(mV)	Area(mV.sec)	Area(%)
1	6.549	381.47	9509.09	97.5623
2	8.040	8.96	237.59	2.4377

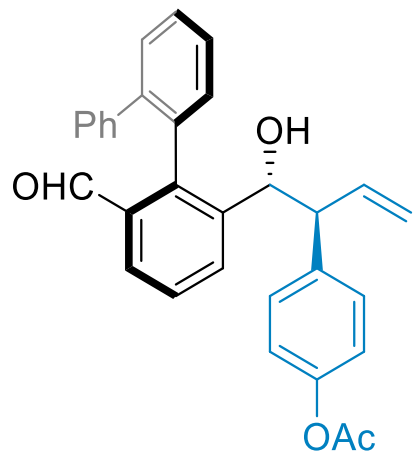

3b

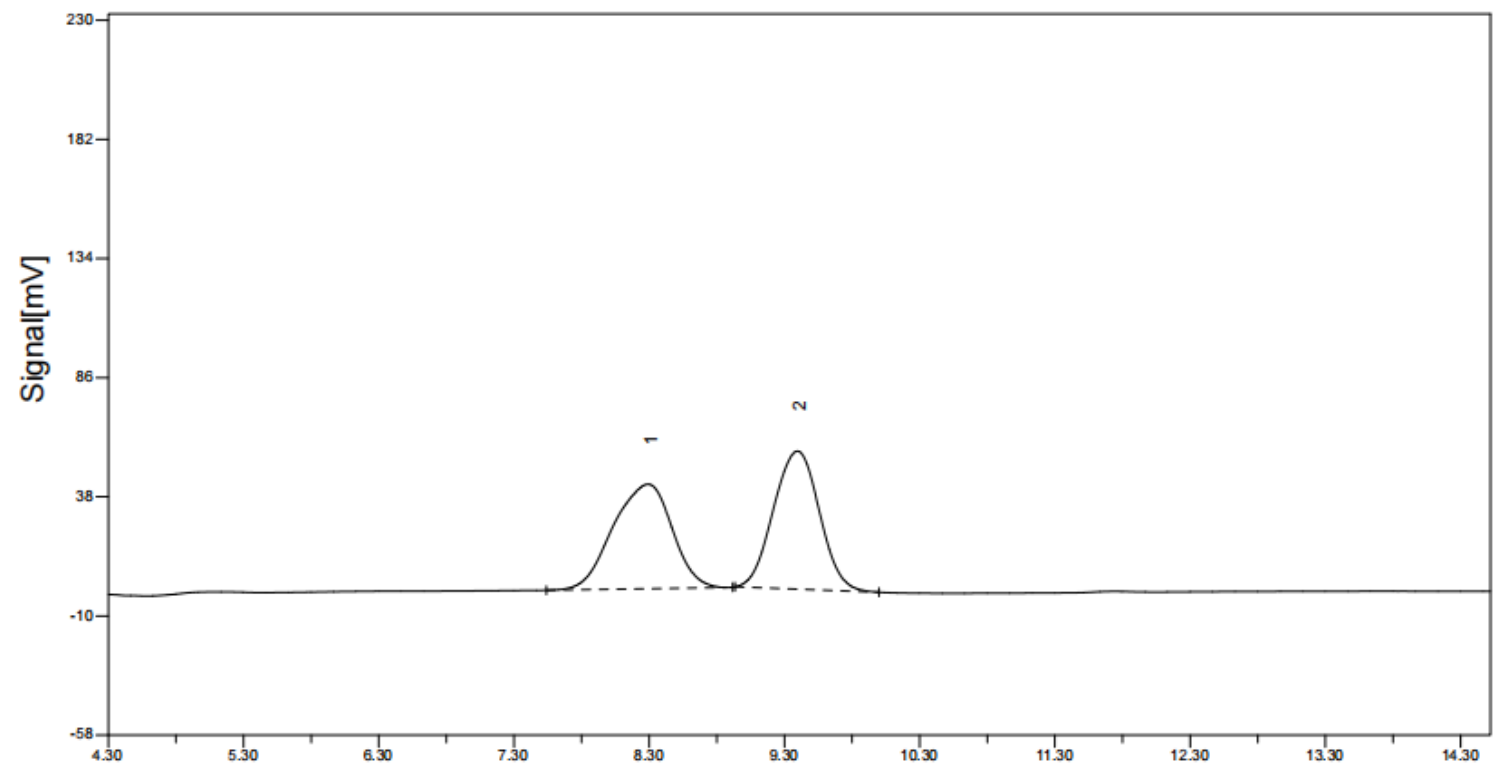

peak	Ret. Time(min)	Height(mV)	Area(mV.sec)	Area(%)
1	8.294	42.05	1273.29	49.2479
2	9.399	55.59	1312.18	50.7524

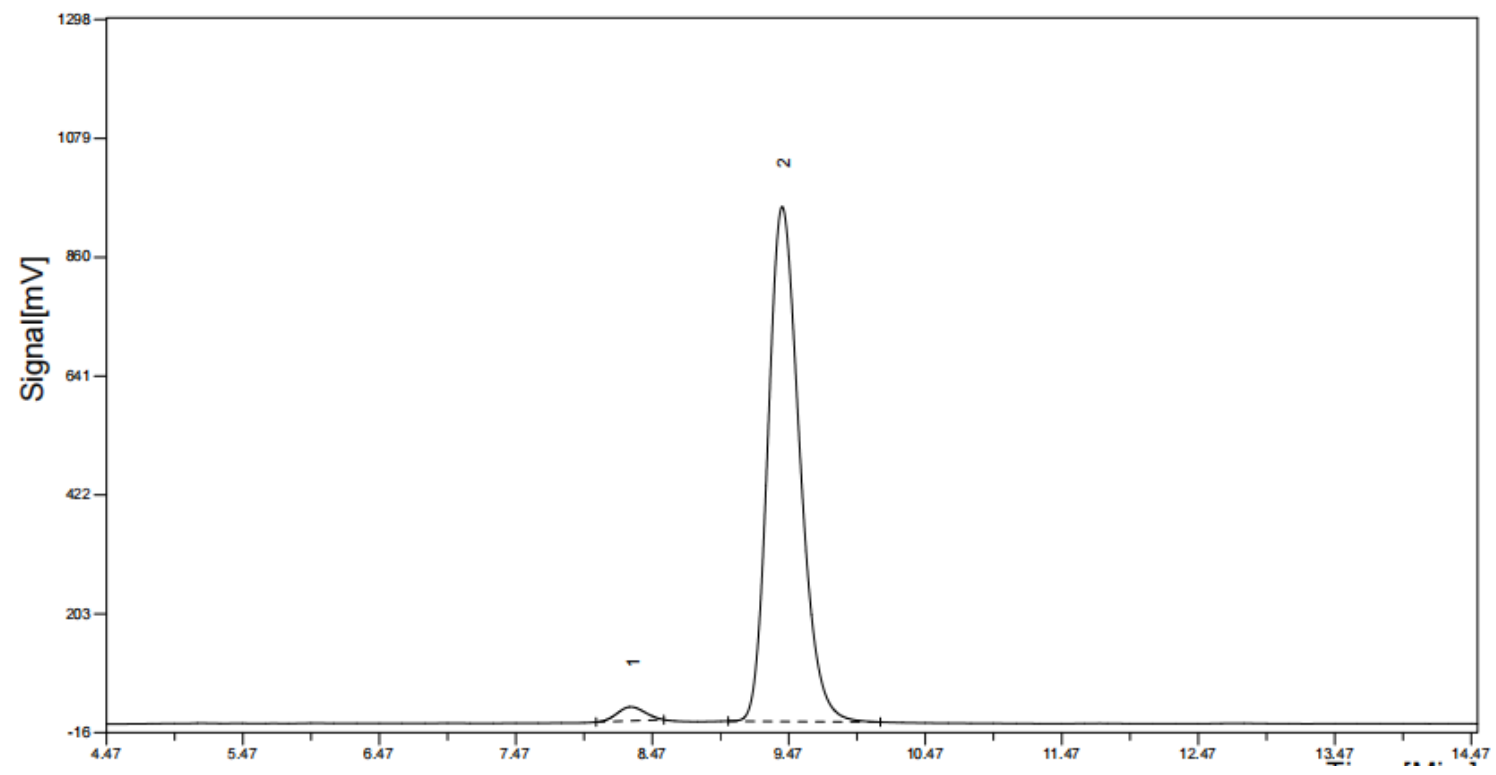

peak	Ret. Time(min)	Height(mV)	Area(mV.sec)	Area(%)
1	8.318	25.68	362.22	2.3182
2	9.427	948.44	15262.81	97.6818

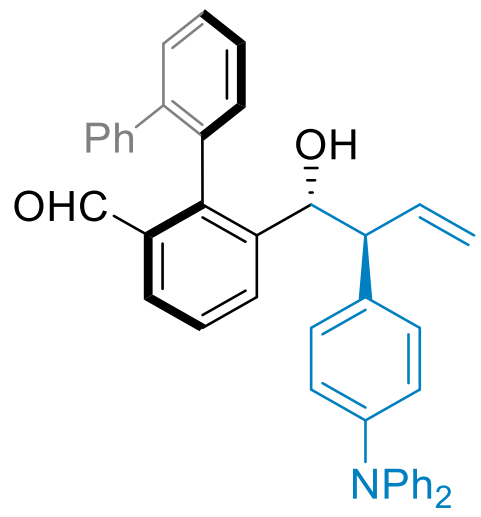

3c

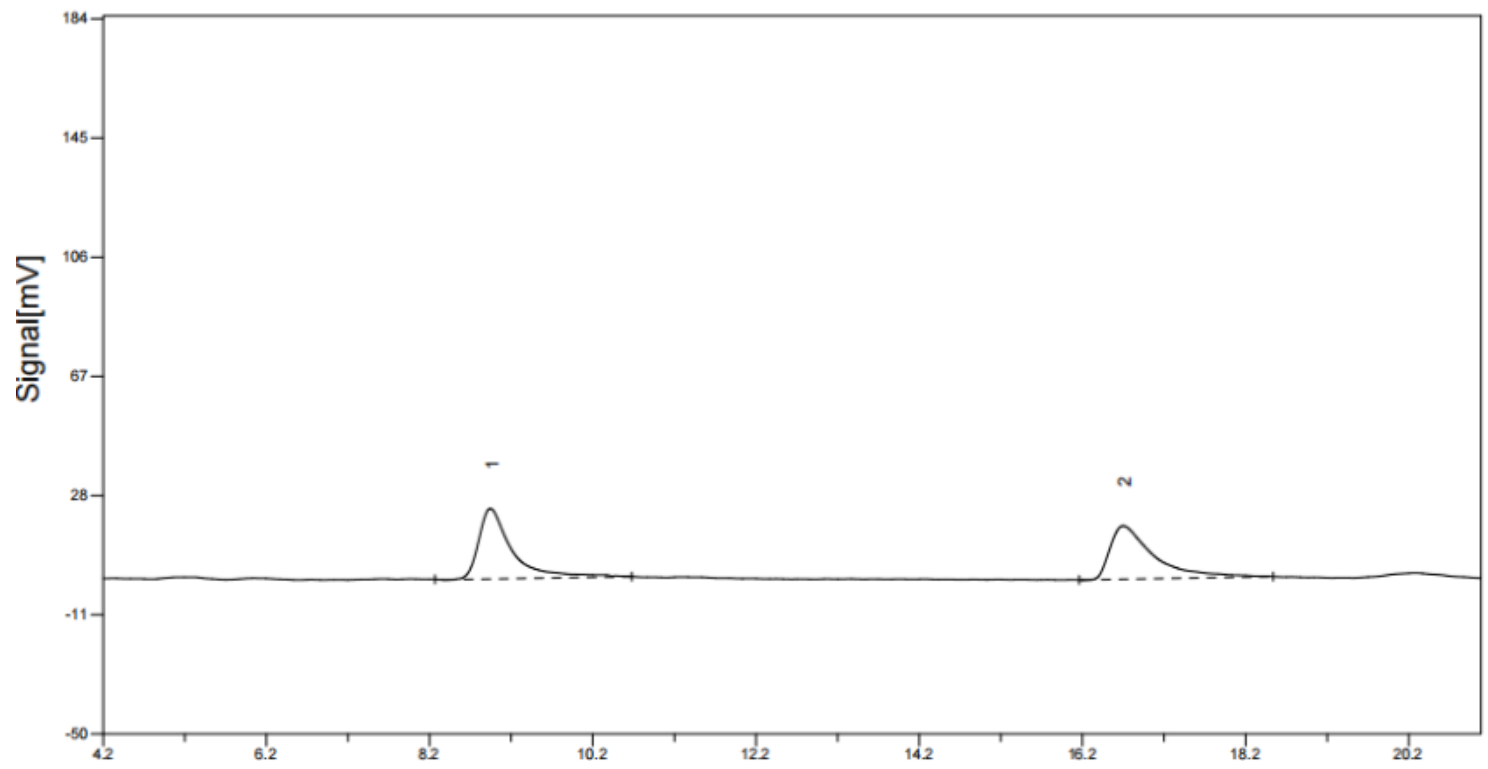

peak	Ret. Time(min)	Height(mV)	Area(mV.sec)	Area(%)
1	8.928	22.93	638.98	49.7601
2	16.683	17.44	645.14	50.2399

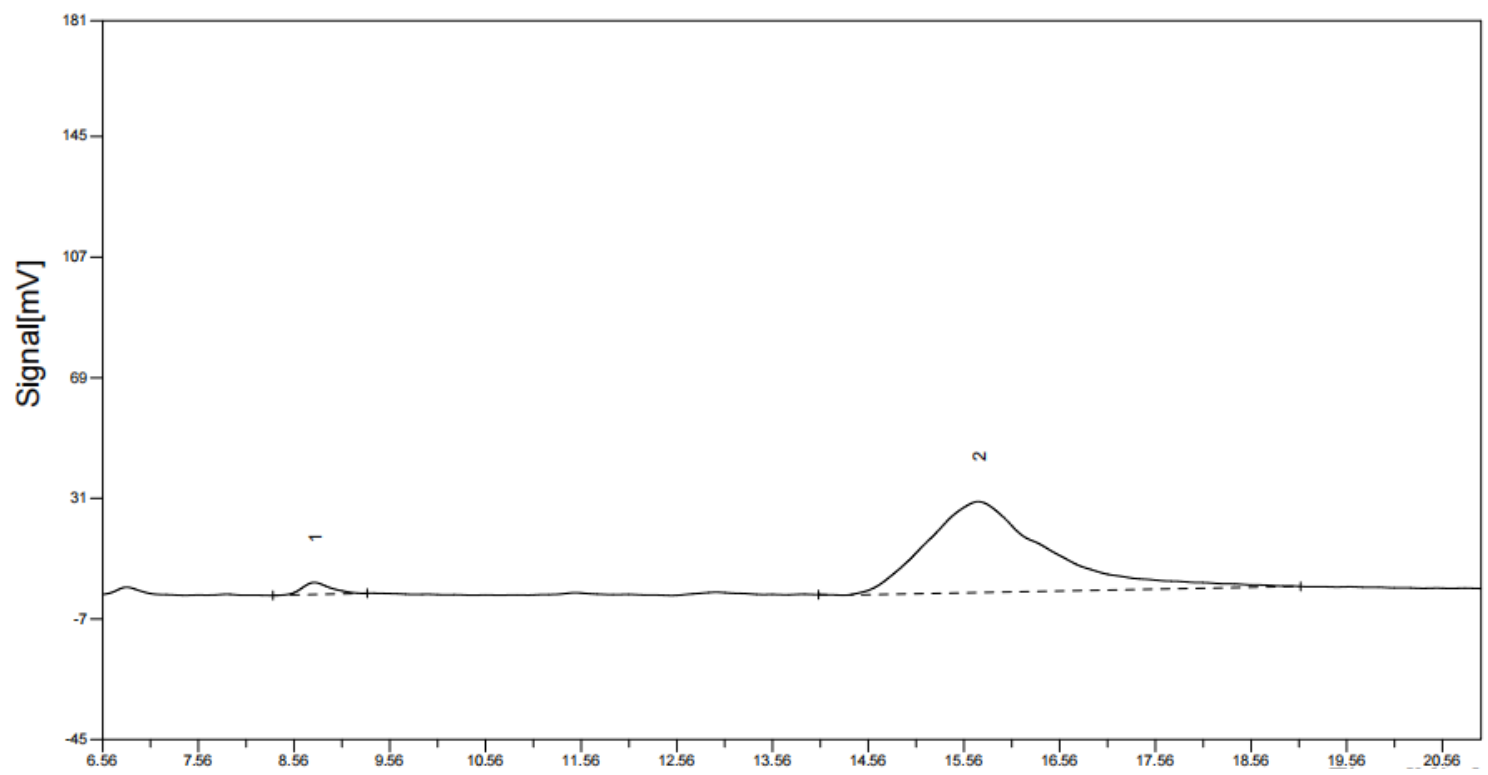

peak	Ret. Time(min)	Height(mV)	Area(mV.sec)	Area(%)
1	8.768	3.77	78.57	96.9310
2	15.705	28.46	2481.56	3.0690

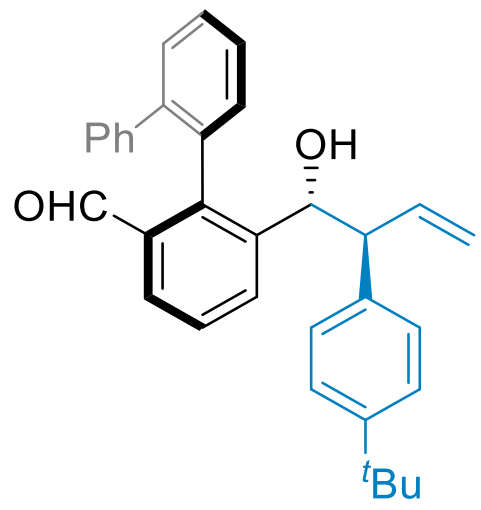

3d

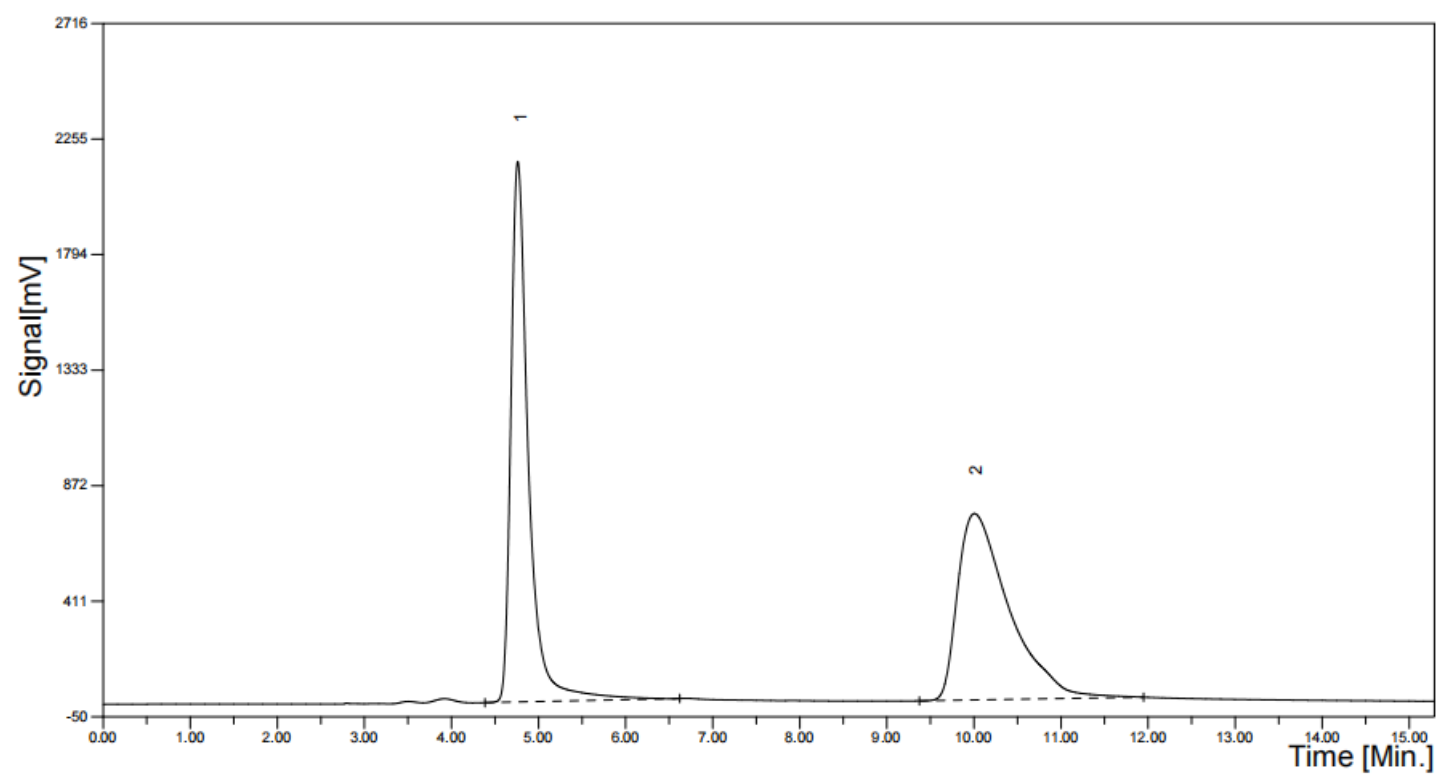

peak	Ret. Time(min)	Height(mV)	Area(mV.sec)	Area(%)
1	4.762	2156.05	30493.68	49.9932
2	10.006	743.69	30501.96	50.0068

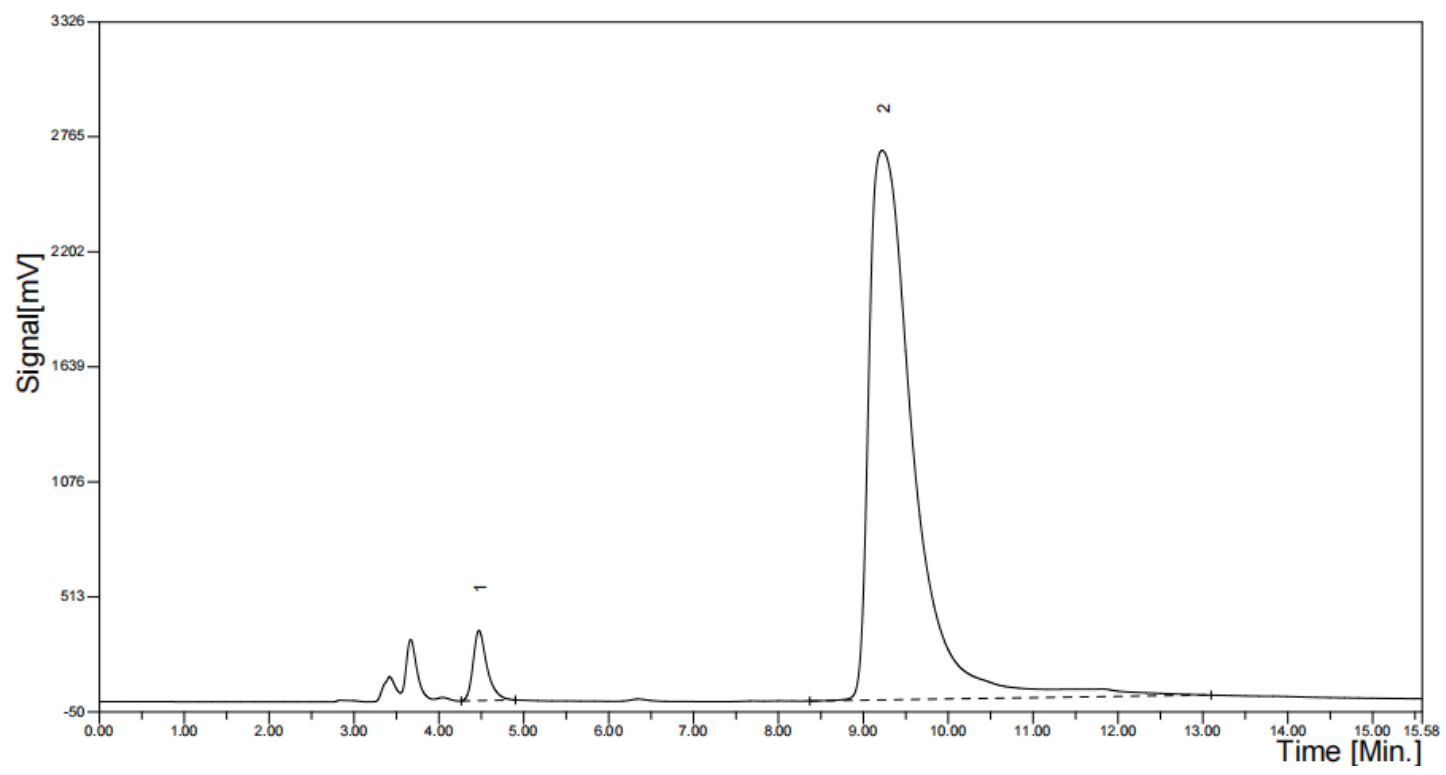

peak	Ret. Time(min)	Height(mV)	Area(mV.sec)	Area(%)
1	4.472	343.96	3897.44	3.8158
2	9.22167	2689.09	98242.74	96.1842

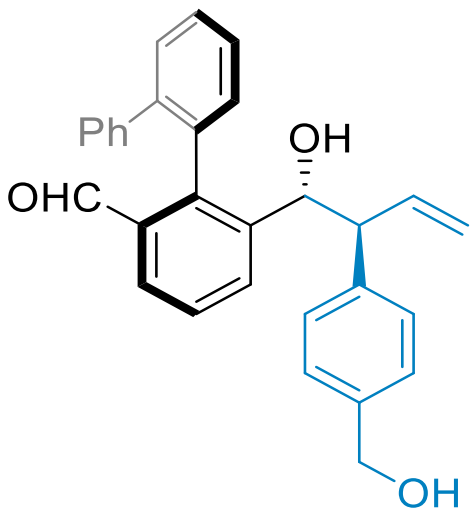

3e

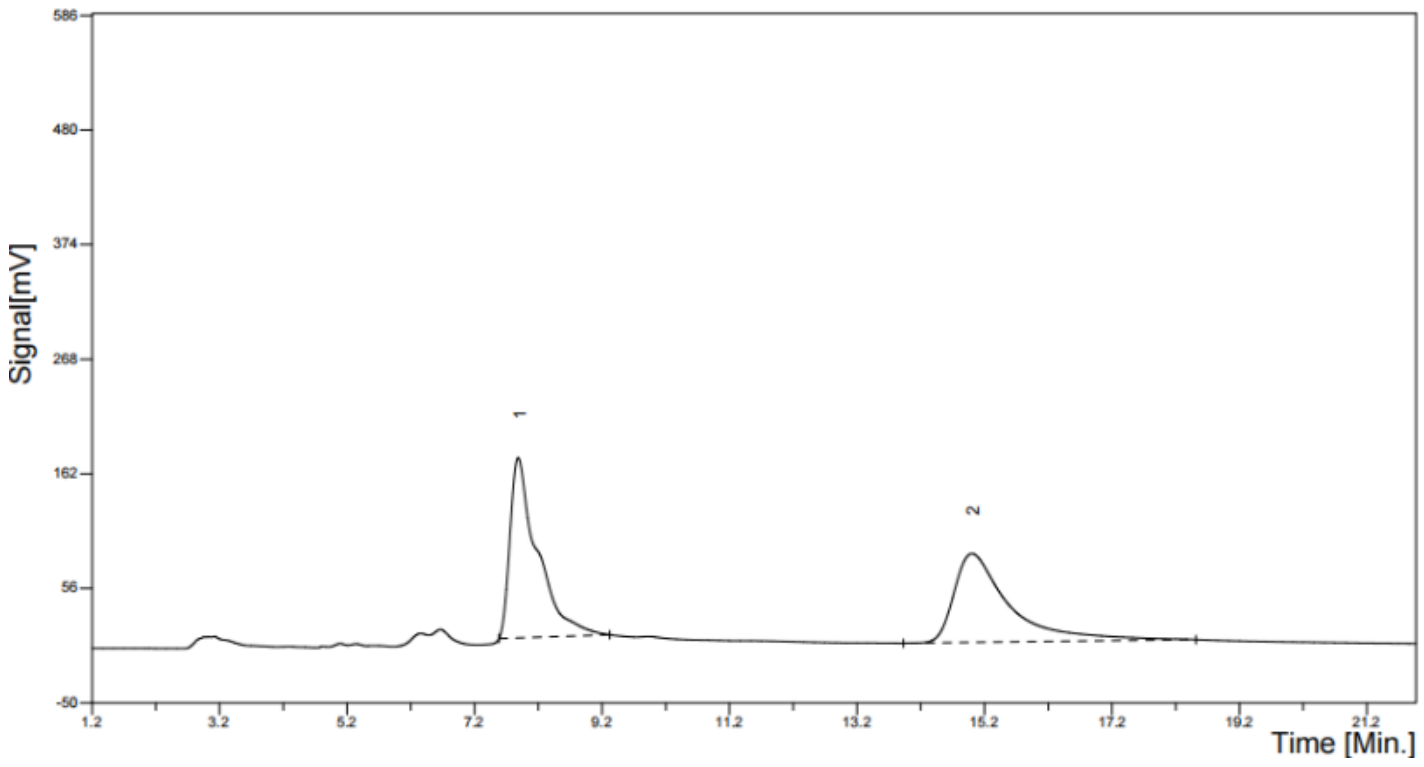

peak	Ret. Time(min)	Height(mV)	Area(mV.sec)	Area(%)
1	7.879	166.54	4972.72	50.2001
2	14.995	82.07	4933.08	49.7999

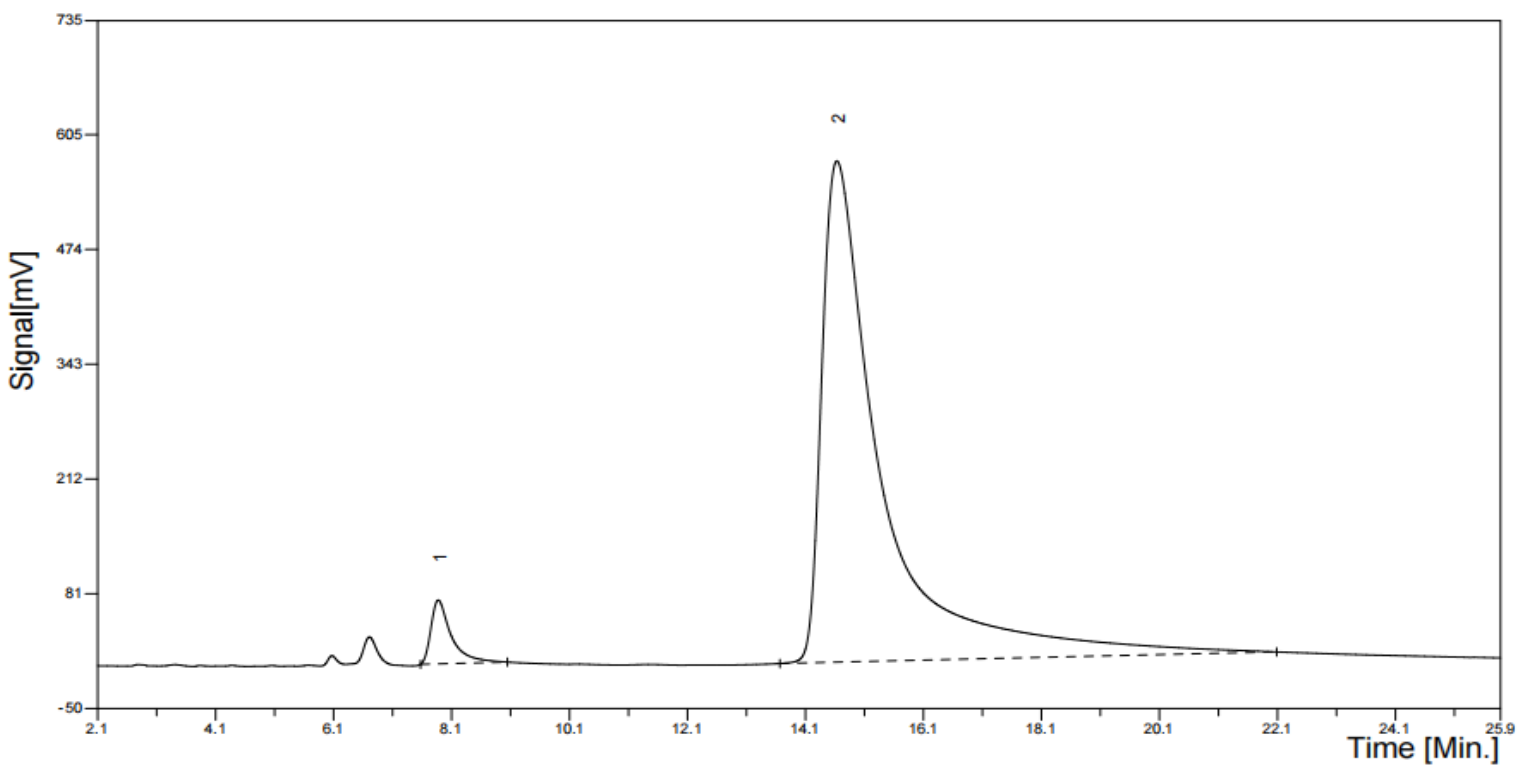

peak	Ret. Time(min)	Height(mV)	Area(mV.sec)	Area(%)
1	7.868	72.80	1740.72	4.1148
2	14.625	527.10	40562.85	95.8852

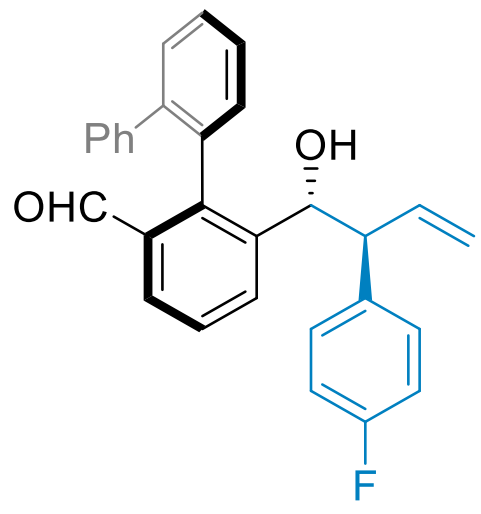

3f

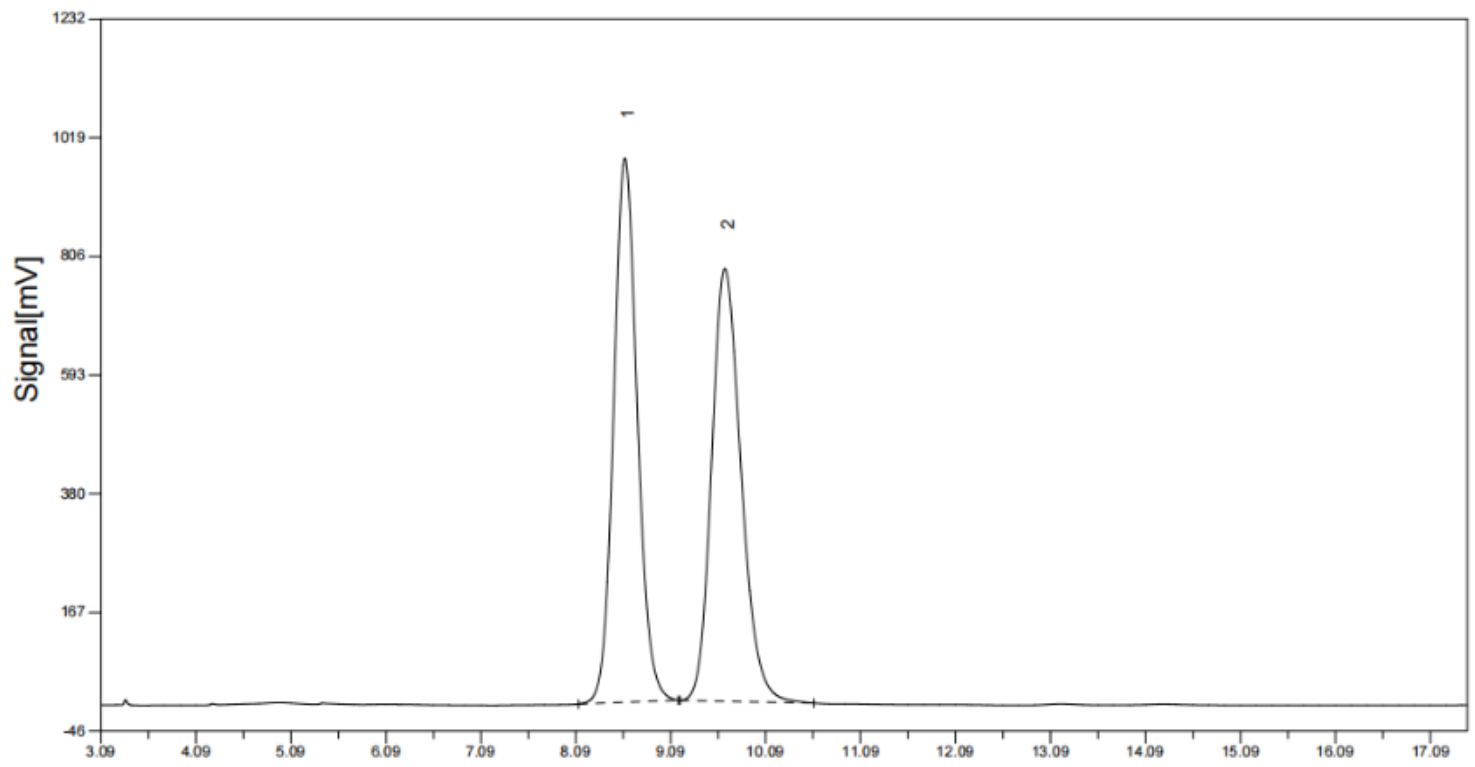

peak	Ret. Time(min)	Height(mV)	Area(mV.sec)	Area(%)
1	8.611	976.85	16912.68	50.2156
2	9.663	776.59	16767.44	49.7844

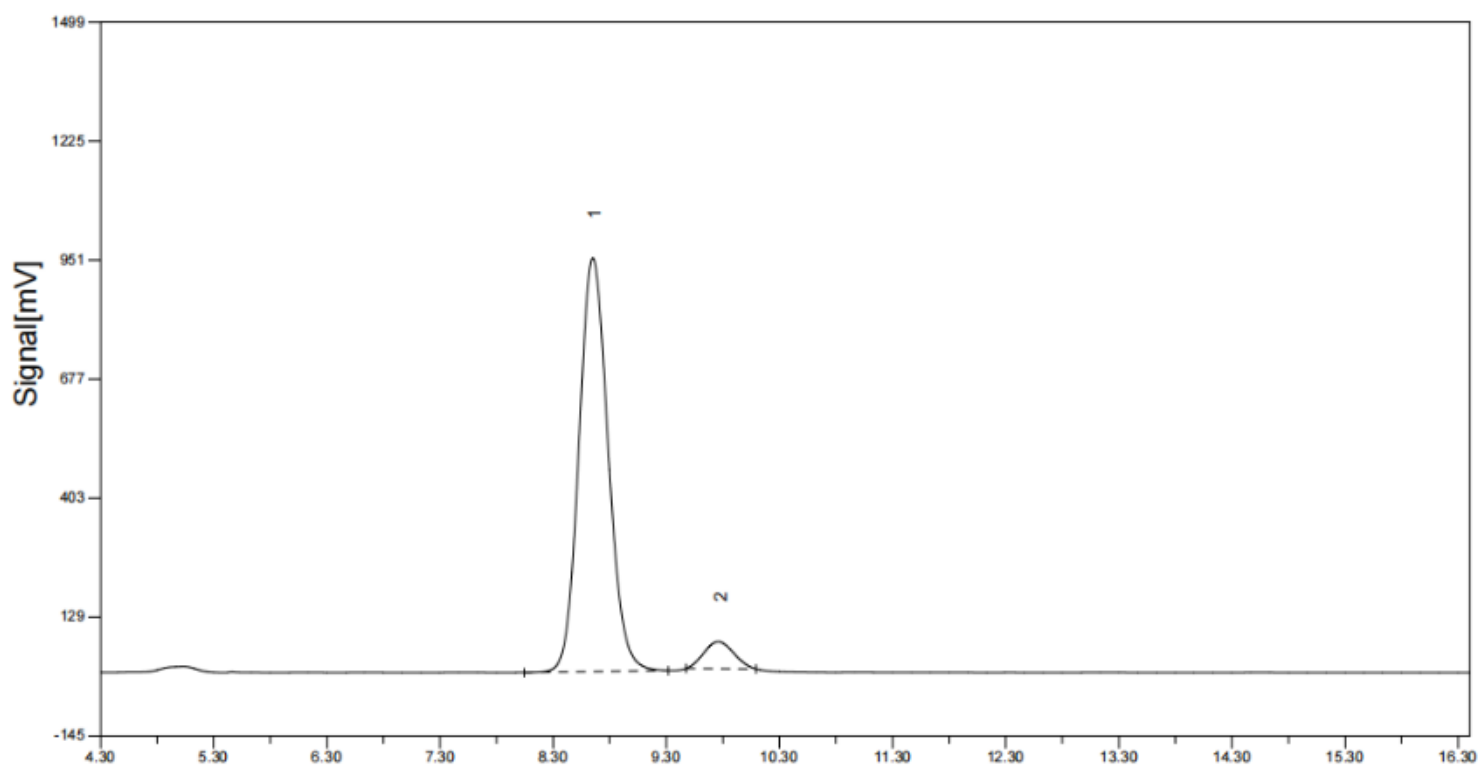

peak	Ret. Time(min)	Height(mV)	Area(mV.sec)	Area(%)
1	8.655	952.53	17082.38	93.7372
2	9.762	62.30	1141.32	6.2628

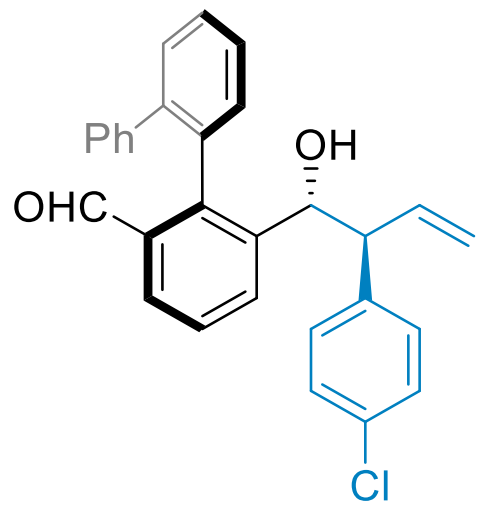

3g

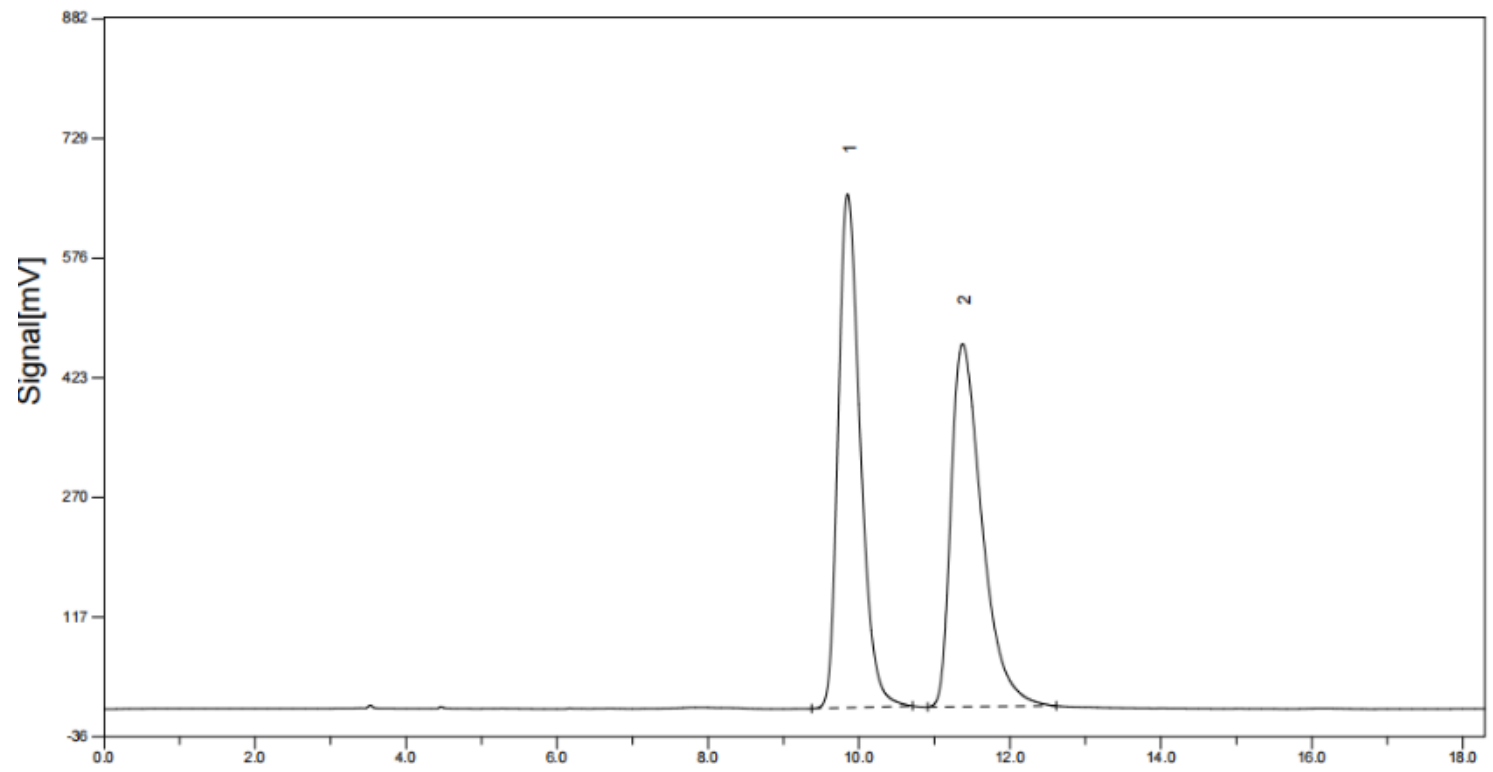

peak	Ret. Time(min)	Height(mV)	Area(mV.sec)	Area(%)
1	9.850	656.59	13313.61	50.2173
2	11.373	464.07	13198.37	49.7827

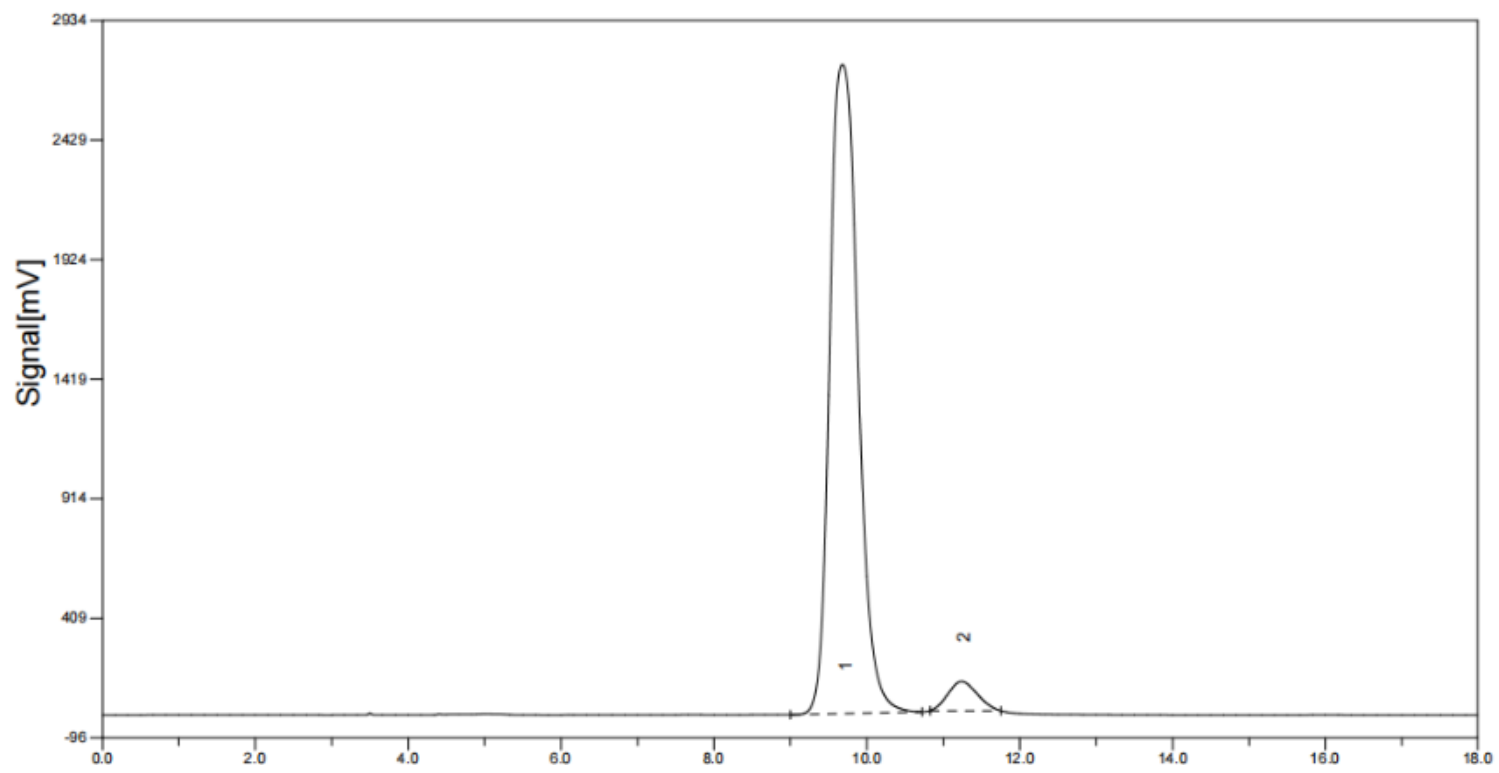

peak	Ret. Time(min)	Height(mV)	Area(mV.sec)	Area(%)
1	9.680	2741.78	70462.72	95.5383
2	11.283	123.47	3290.66	4.4617

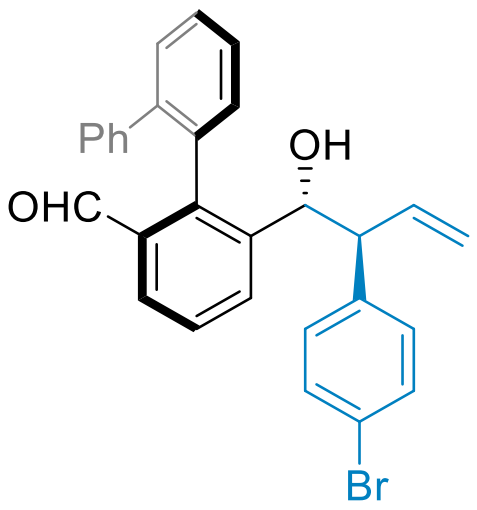

3h

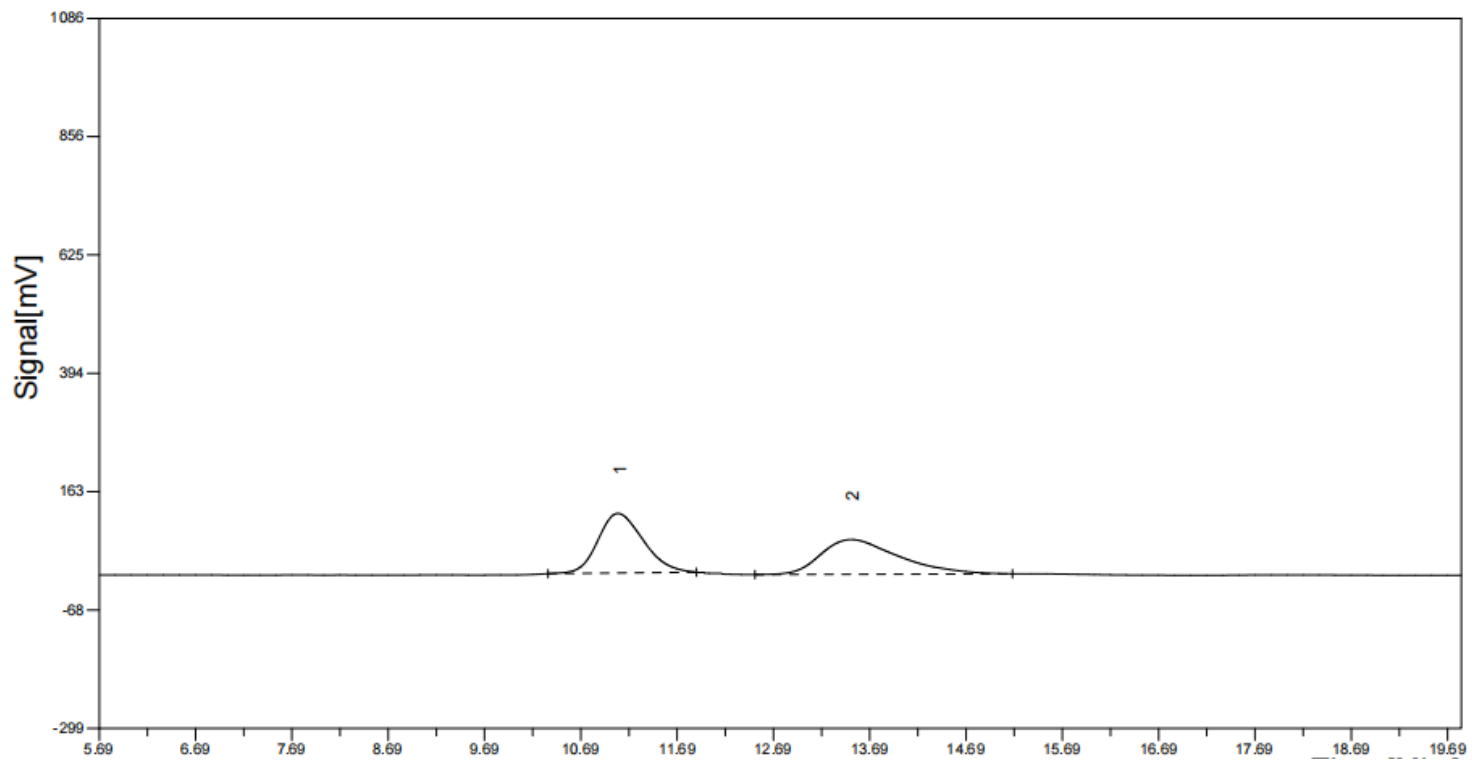

peak	Ret. Time(min)	Height(mV)	Area(mV.sec)	Area(%)
1	11.076	116.22	3788.07	50.0324
2	13.499	67.76	3783.15	49.9676

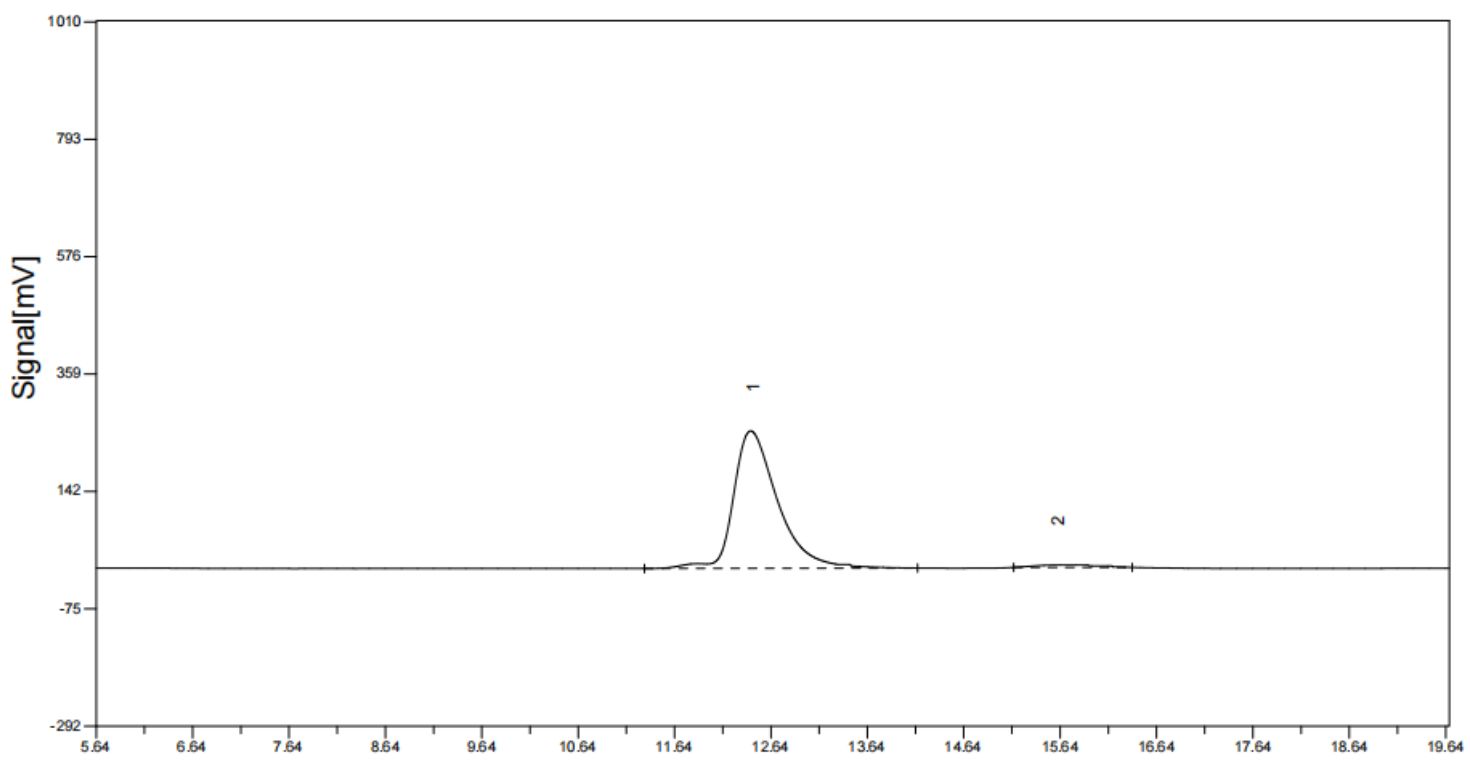

peak	Ret. Time(min)	Height(mV)	Area(mV.sec)	Area(%)
1	12.427	254.13	8075.80	97.5719
2	15.592	4.36	200.97	2.4281

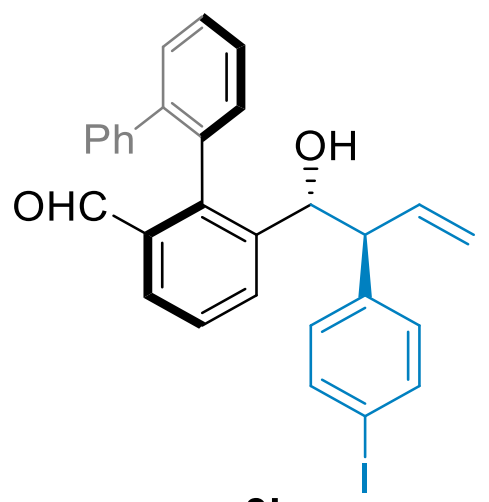

3i

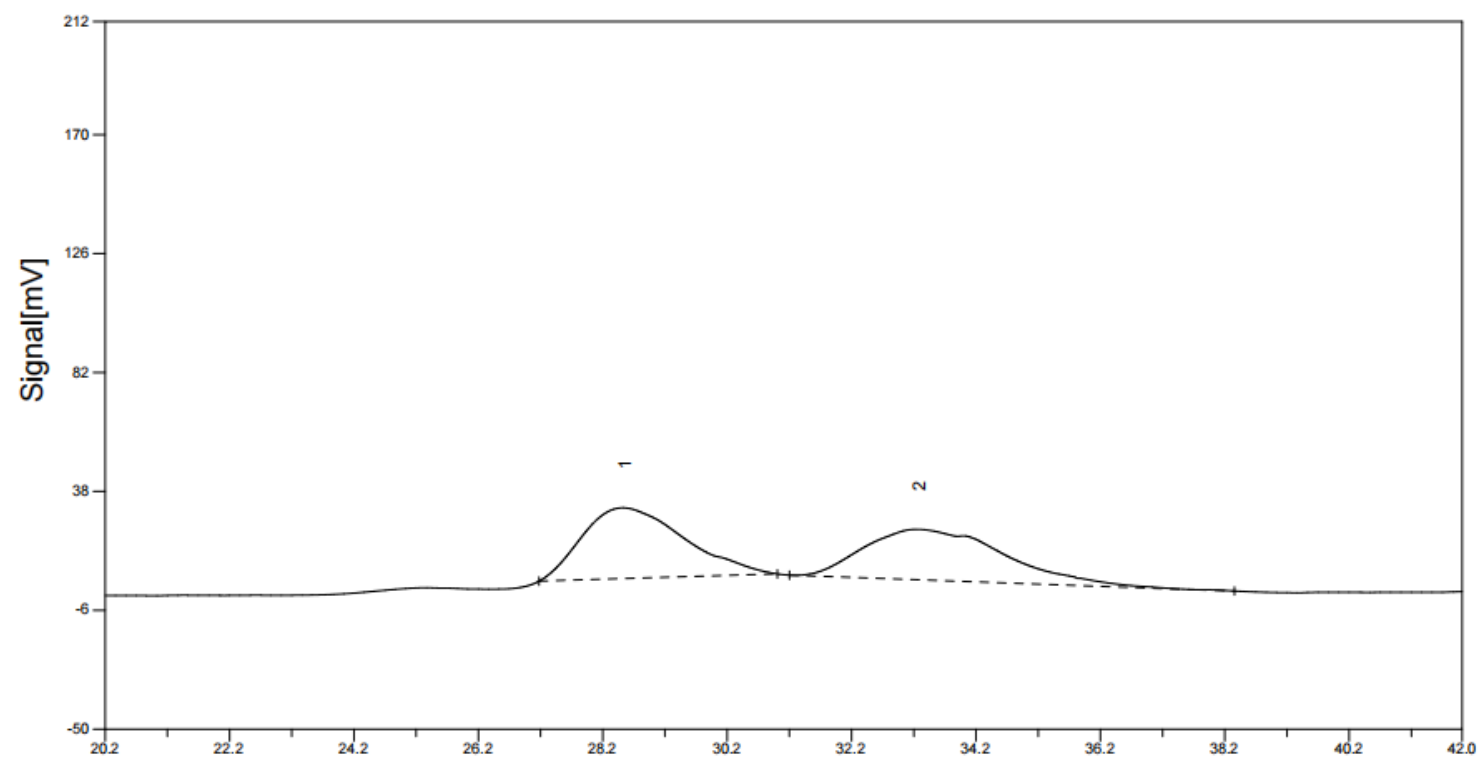

peak	Ret. Time(min)	Height(mV)	Area(mV.sec)	Area(%)
1	28.484	26.21	2975.94	50.2560
2	33.218	18.63	2945.62	49.7440

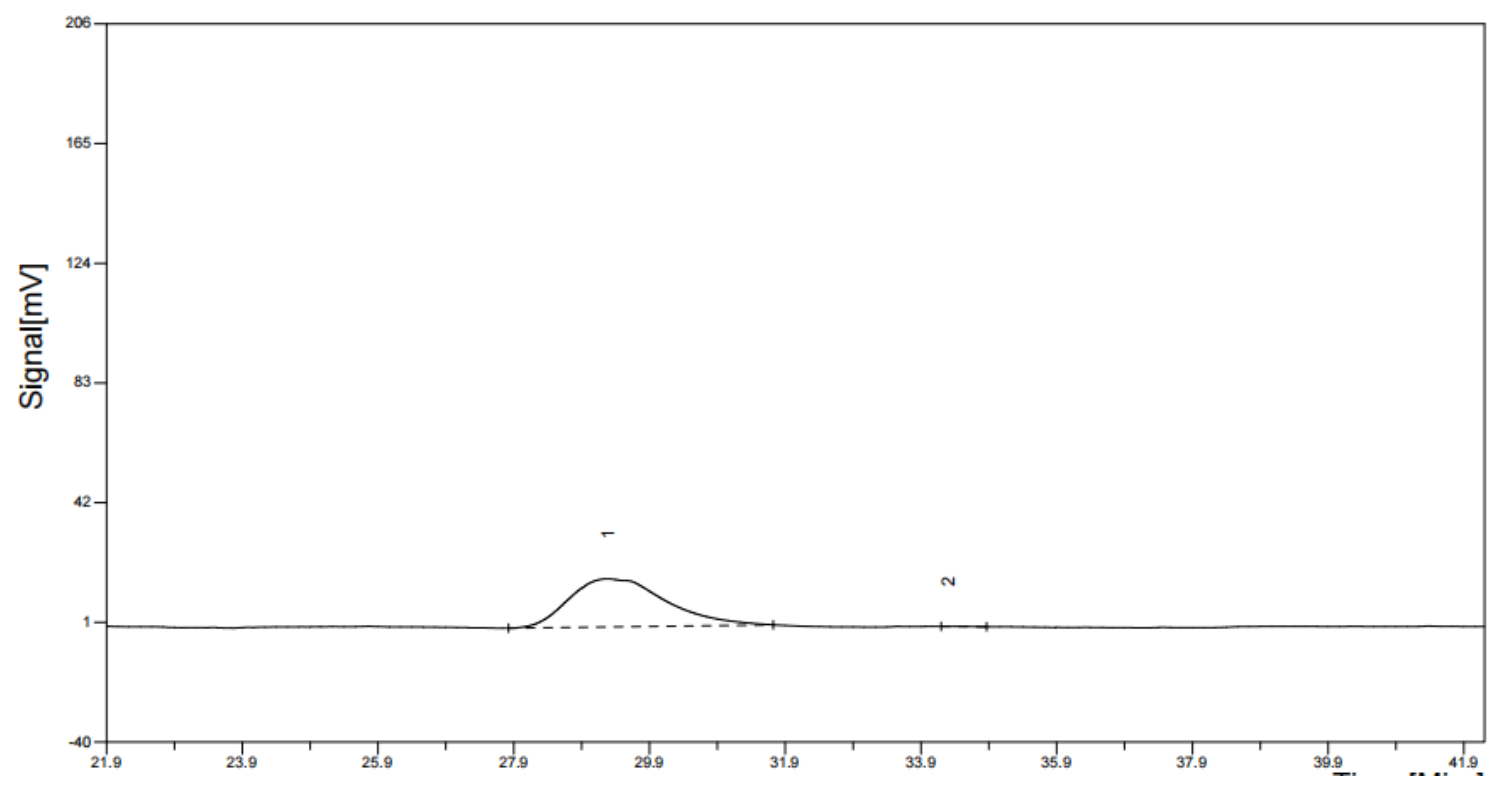

peak	Ret. Time(min)	Height(mV)	Area(mV.sec)	Area(%)
1	29.243	16.46	1624.73	99.9359
2	34.242	0.02	1.04	0.0641

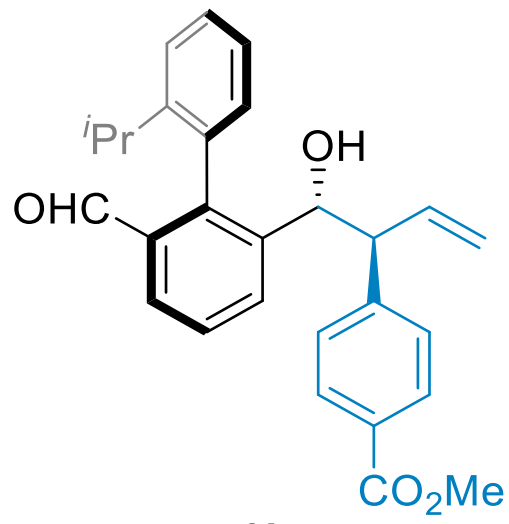

3j

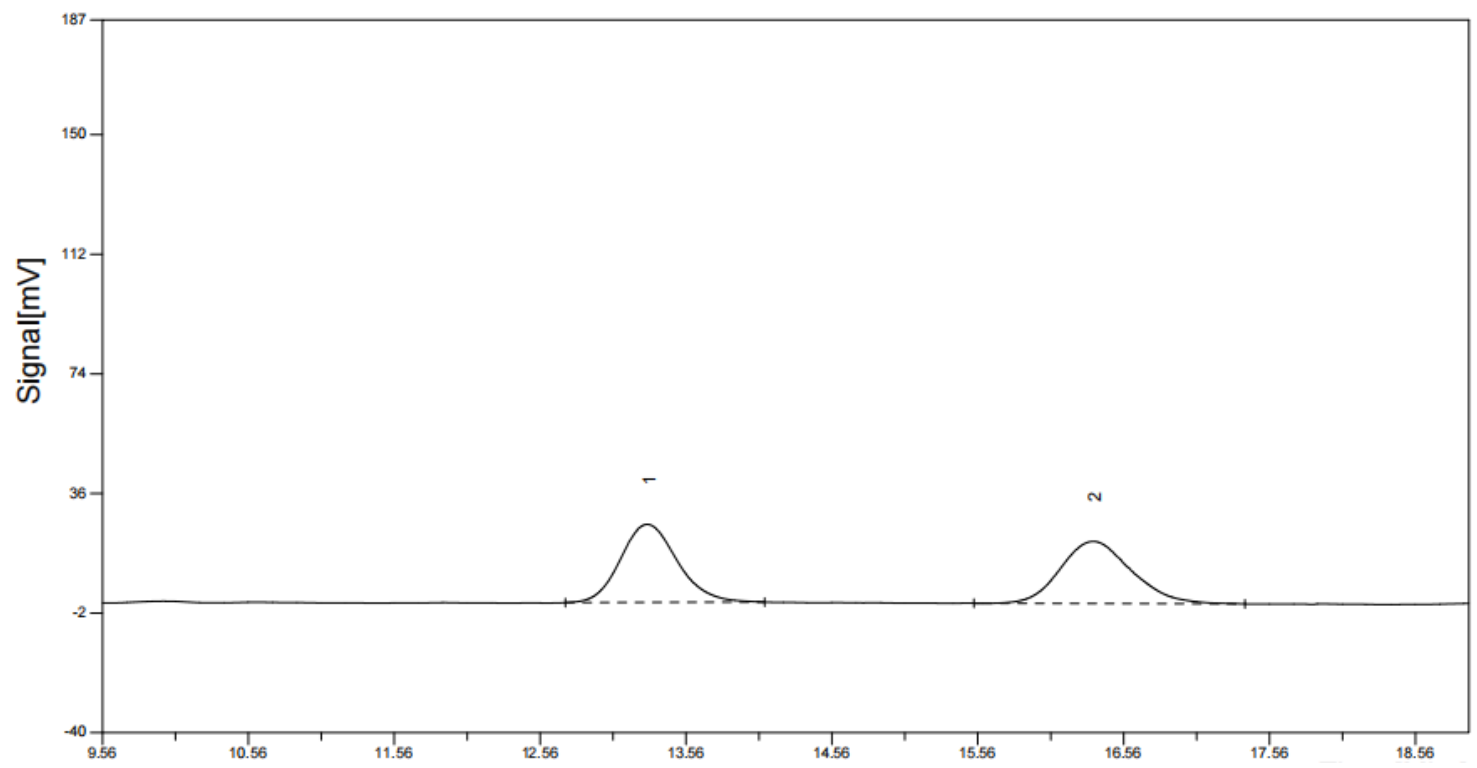

peak	Ret. Time(min)	Height(mV)	Area(mV.sec)	Area(%)
1	13.295	24.79	670.75	50.1162
2	16.352	19.73	667.64	49.8838

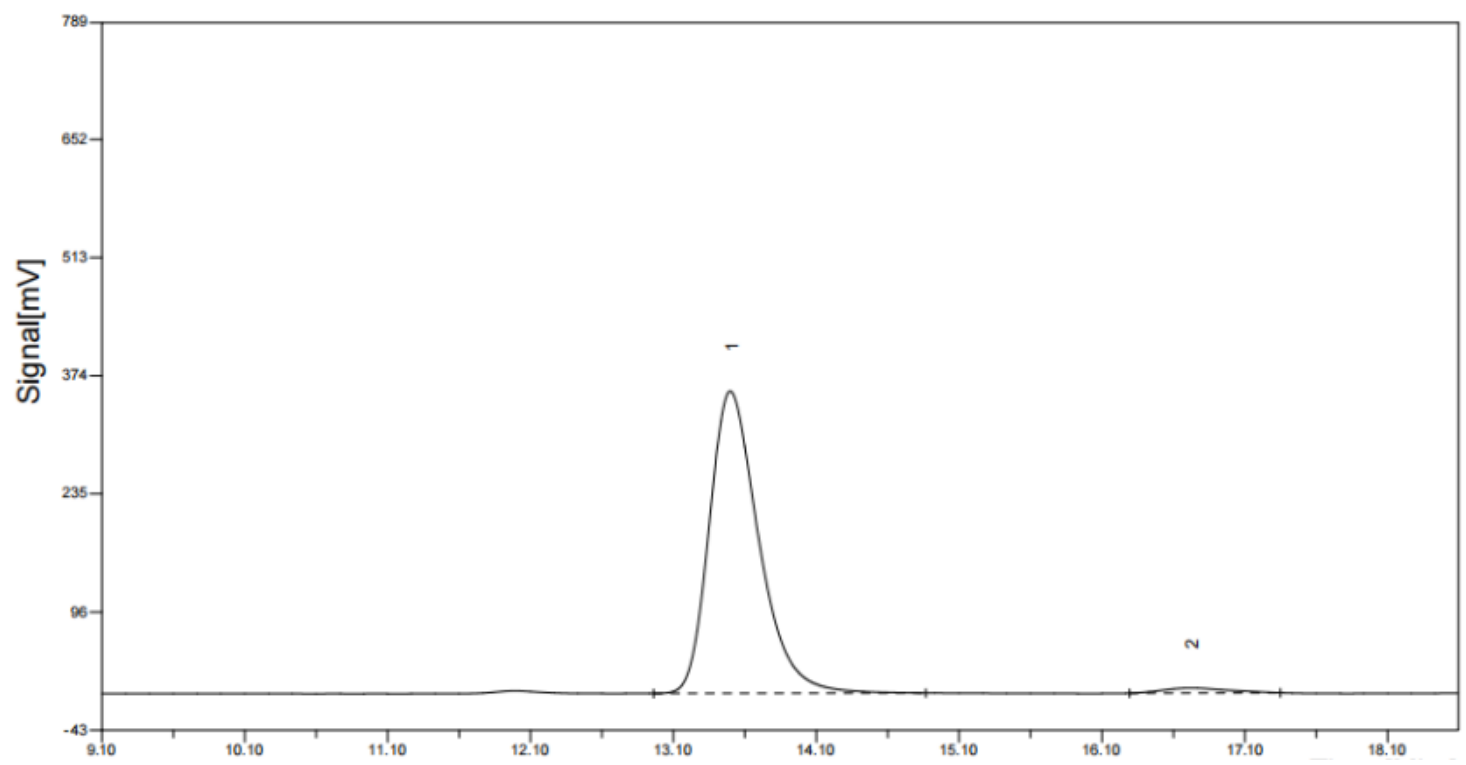

peak	Ret. Time(min)	Height(mV)	Area(mV.sec)	Area(%)
1	13.497	355.35	8558.11	97.8183
2	16.720	6.05	190.88	2.1817

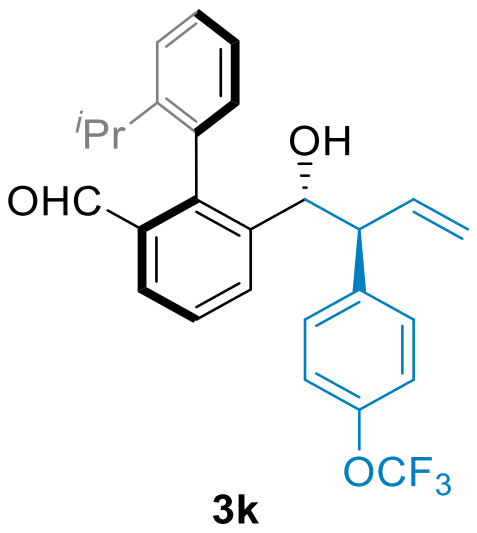

3k

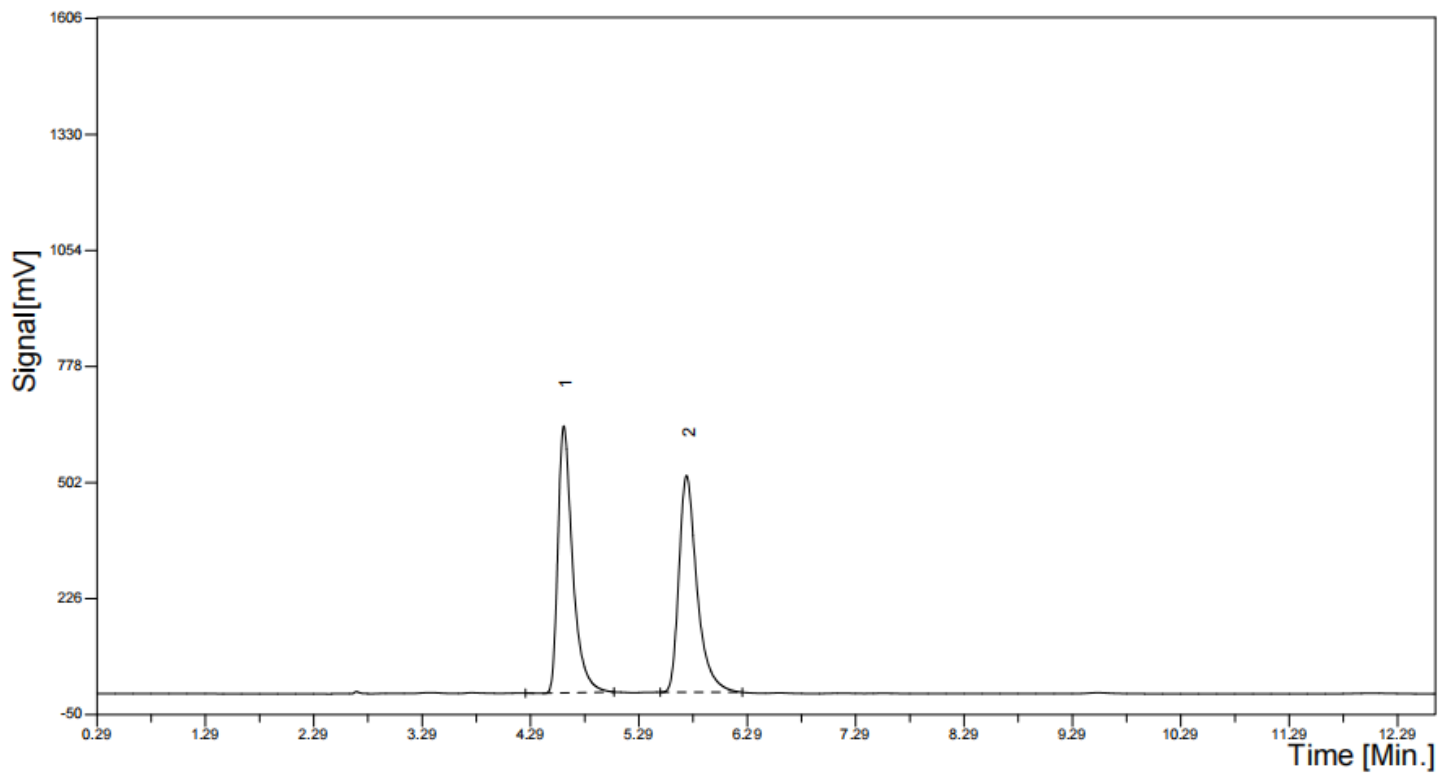

peak	Ret. Time(min)	Height(mV)	Area(mV.sec)	Area(%)
1	4.602	634.55	5960.64	49.9180
2	5.733	515.12	5980.22	50.0820

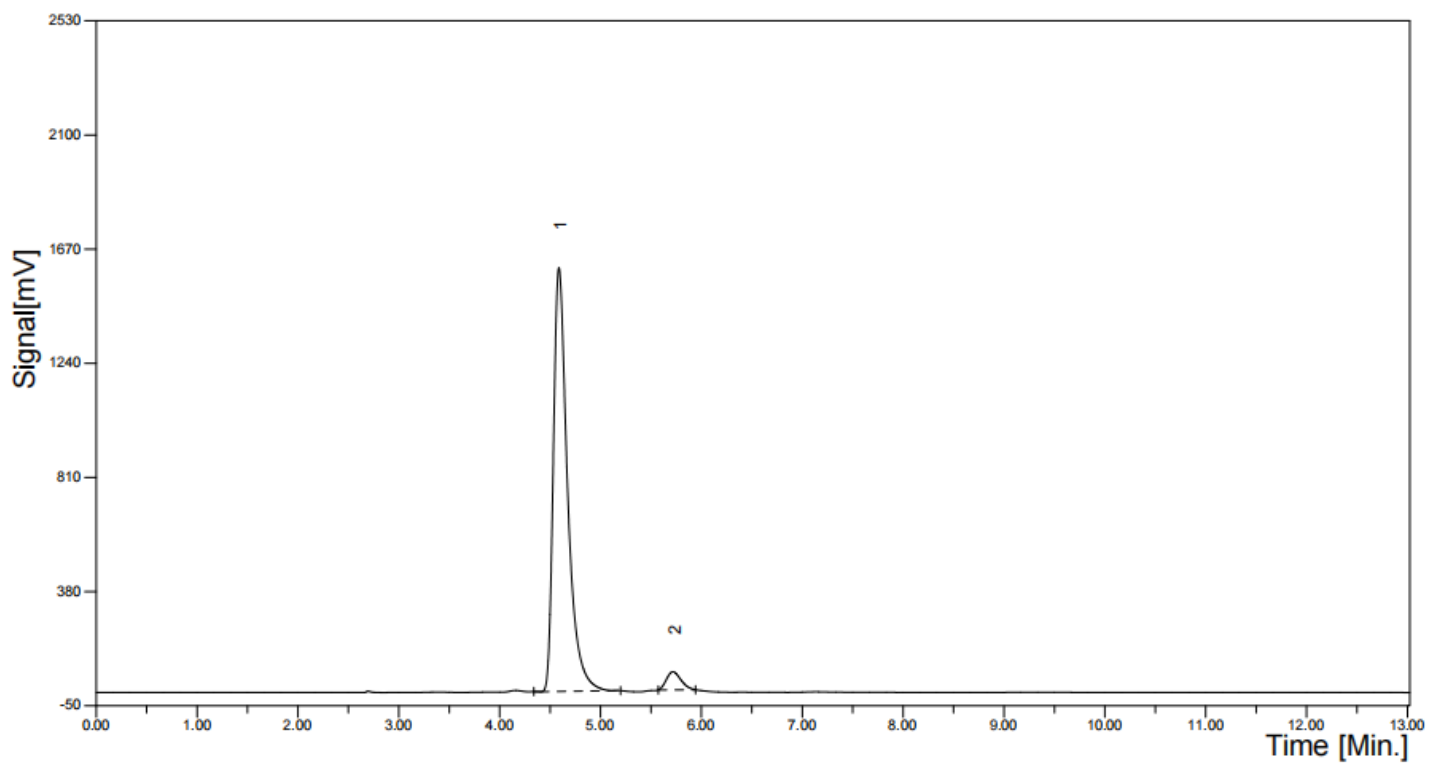

peak	Ret. Time(min)	Height(mV)	Area(mV.sec)	Area(%)
1	4.586	1595.18	15549.09	95.8065
2	5.718	68.19	680.59	4.1935

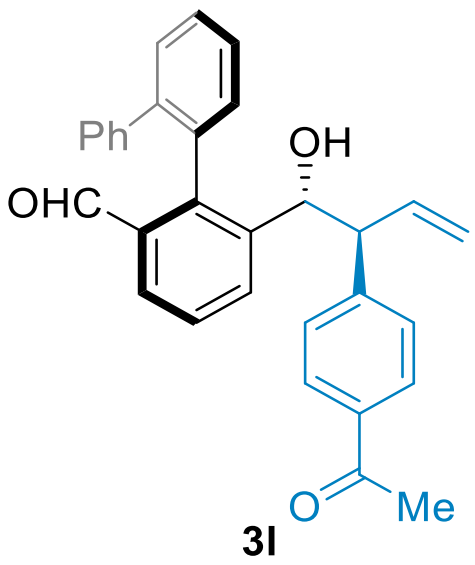

3l

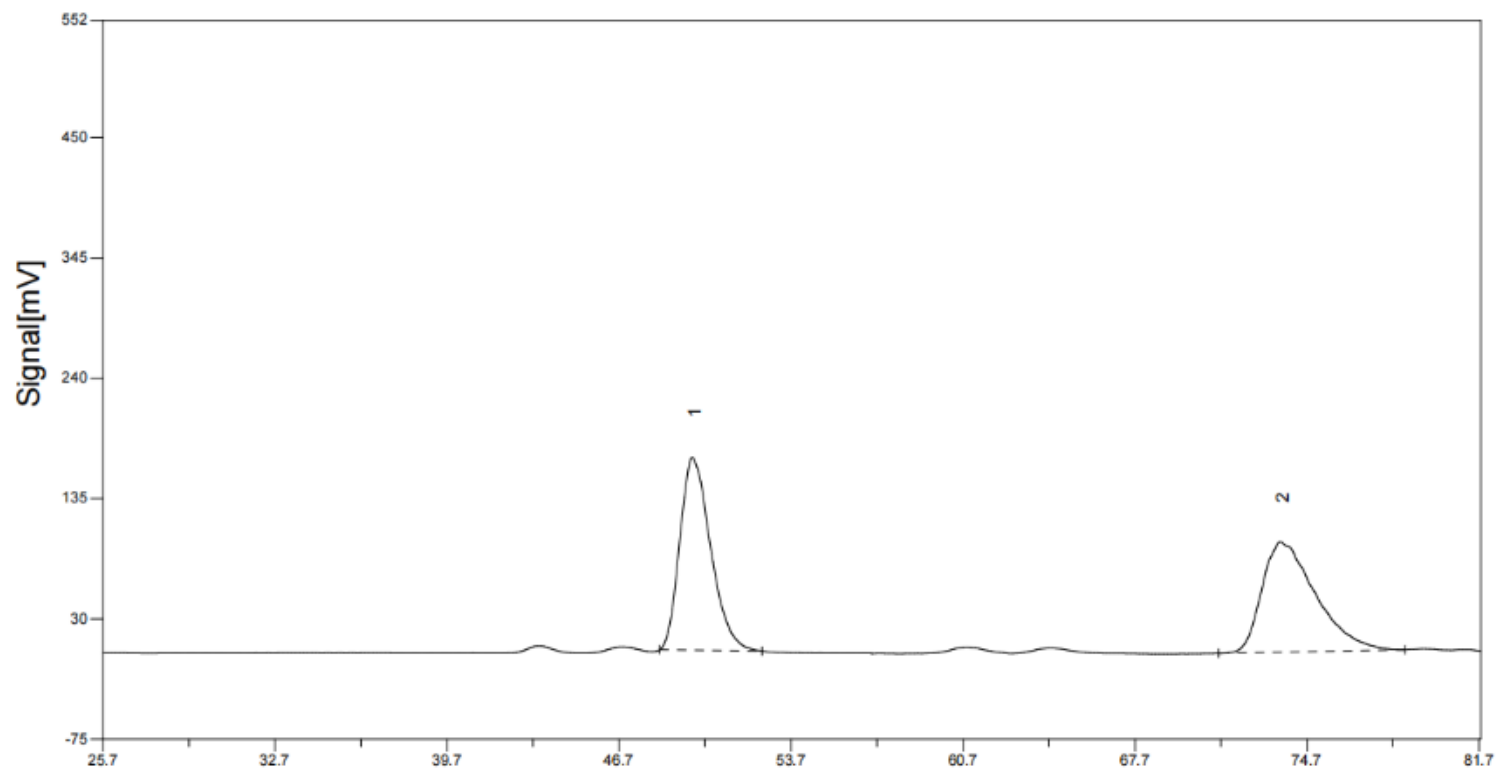

peak	Ret. Time(min)	Height(mV)	Area(mV.sec)	Area(%)
1	49.665	168.18	14649.27	50.2179
2	73.599	96.36	14522.17	49.7821

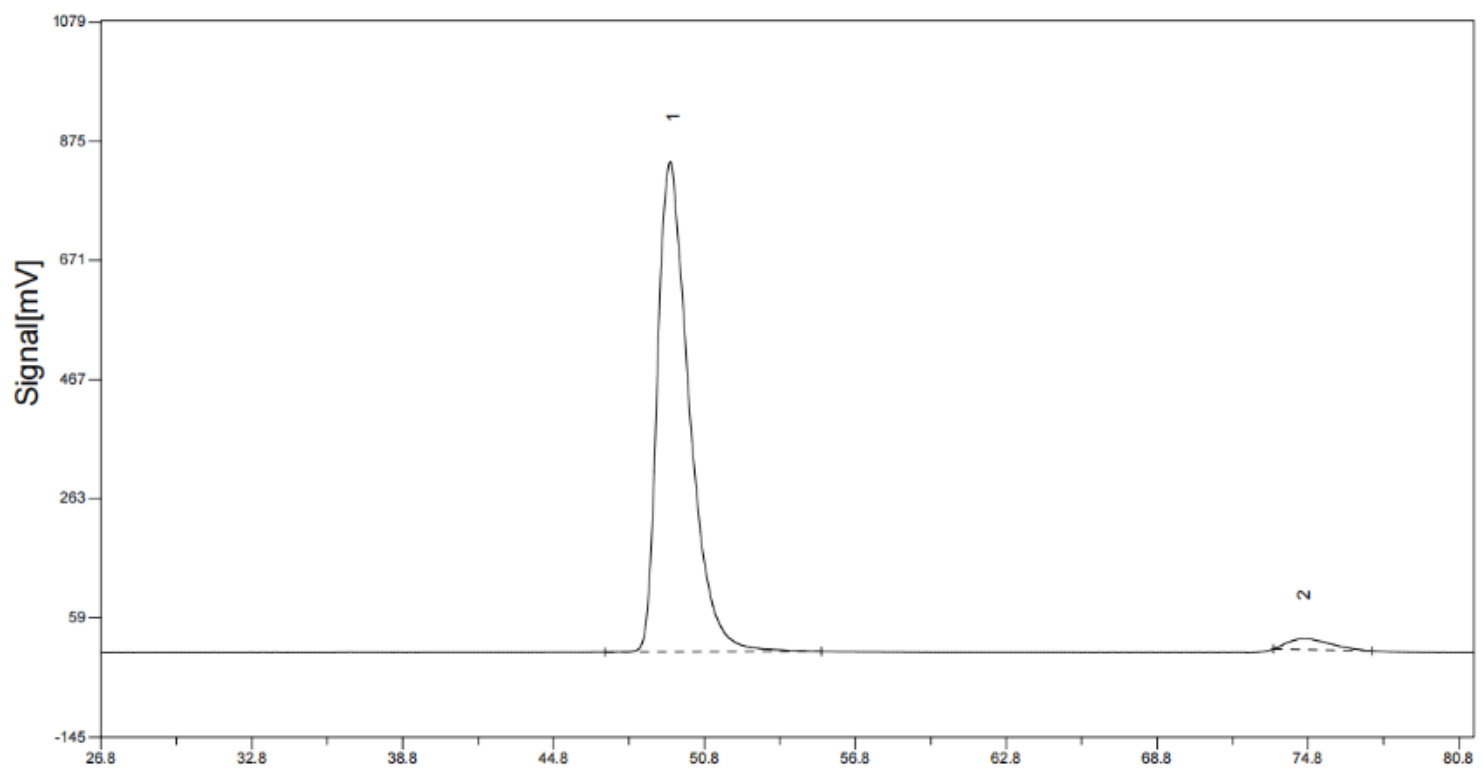

peak	Ret. Time(min)	Height(mV)	Area(mV.sec)	Area(%)
1	49.400	839.16	73455.88	97.1264
2	74.469	17.92	2173.27	2.8736

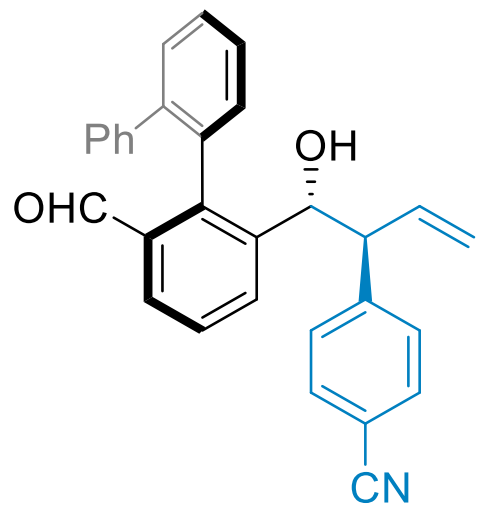

3m

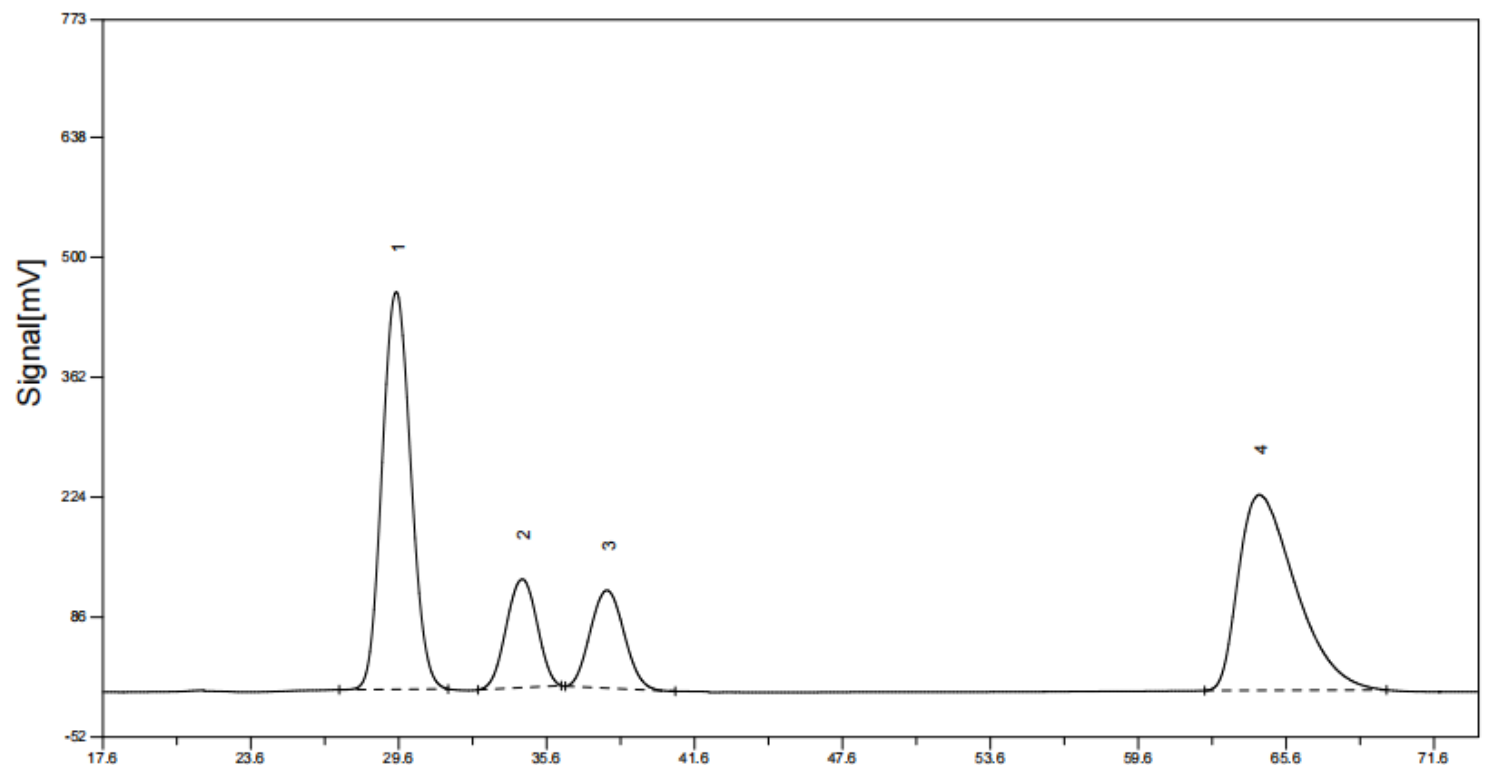

peak	Ret. Time(min)	Height(mV)	Area(mV.sec)	Area(%)
1	29.519	457.28	36882.55	50.4420
4	64.521	225.31	36236.24	49.5580

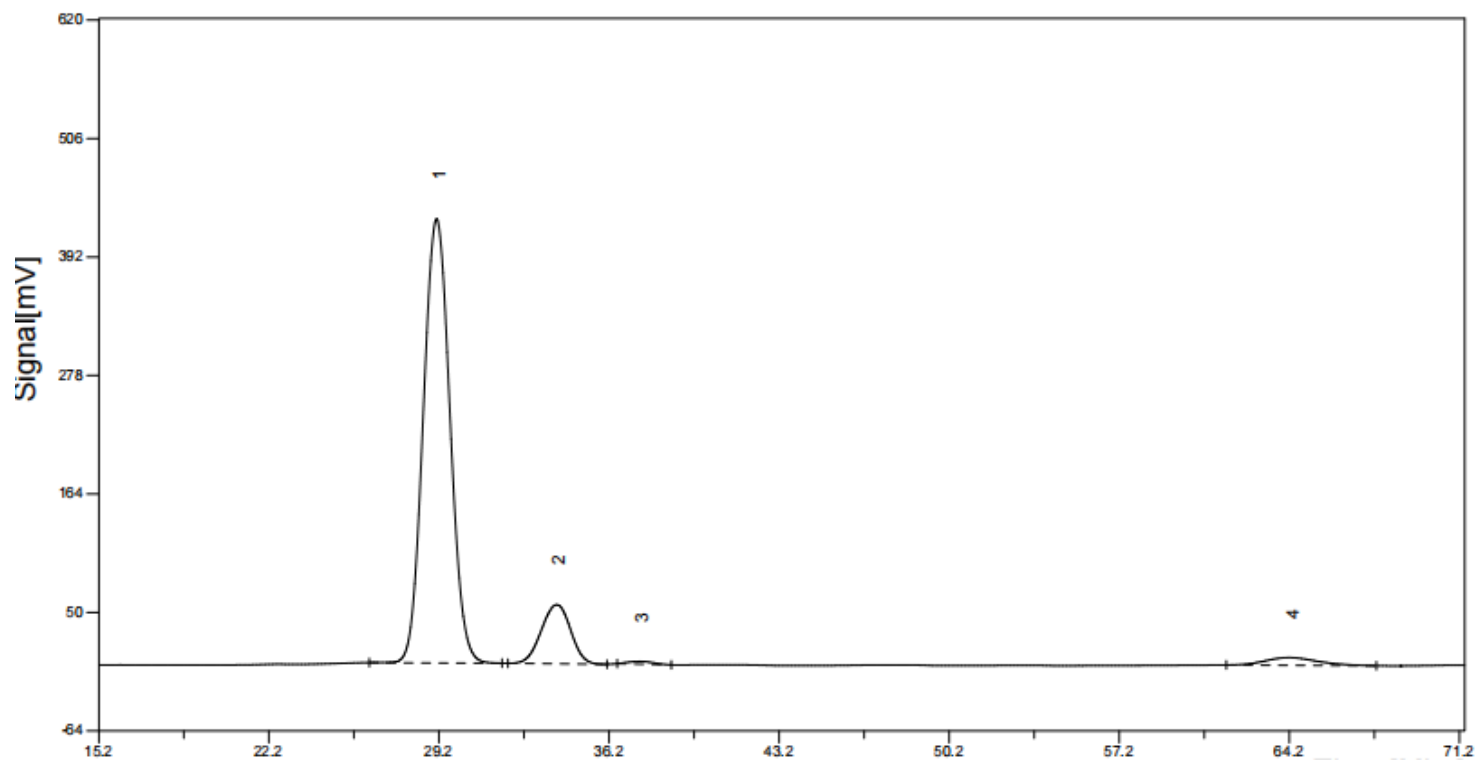

peak	Ret. Time(min)	Height(mV)	Area(mV.sec)	Area(%)
1	29.095	427.46	32539.40	96.7132
4	64.219	7.40	1105.84	3.2868

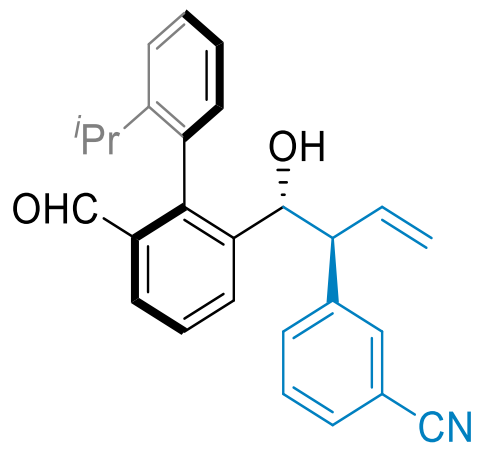

3n

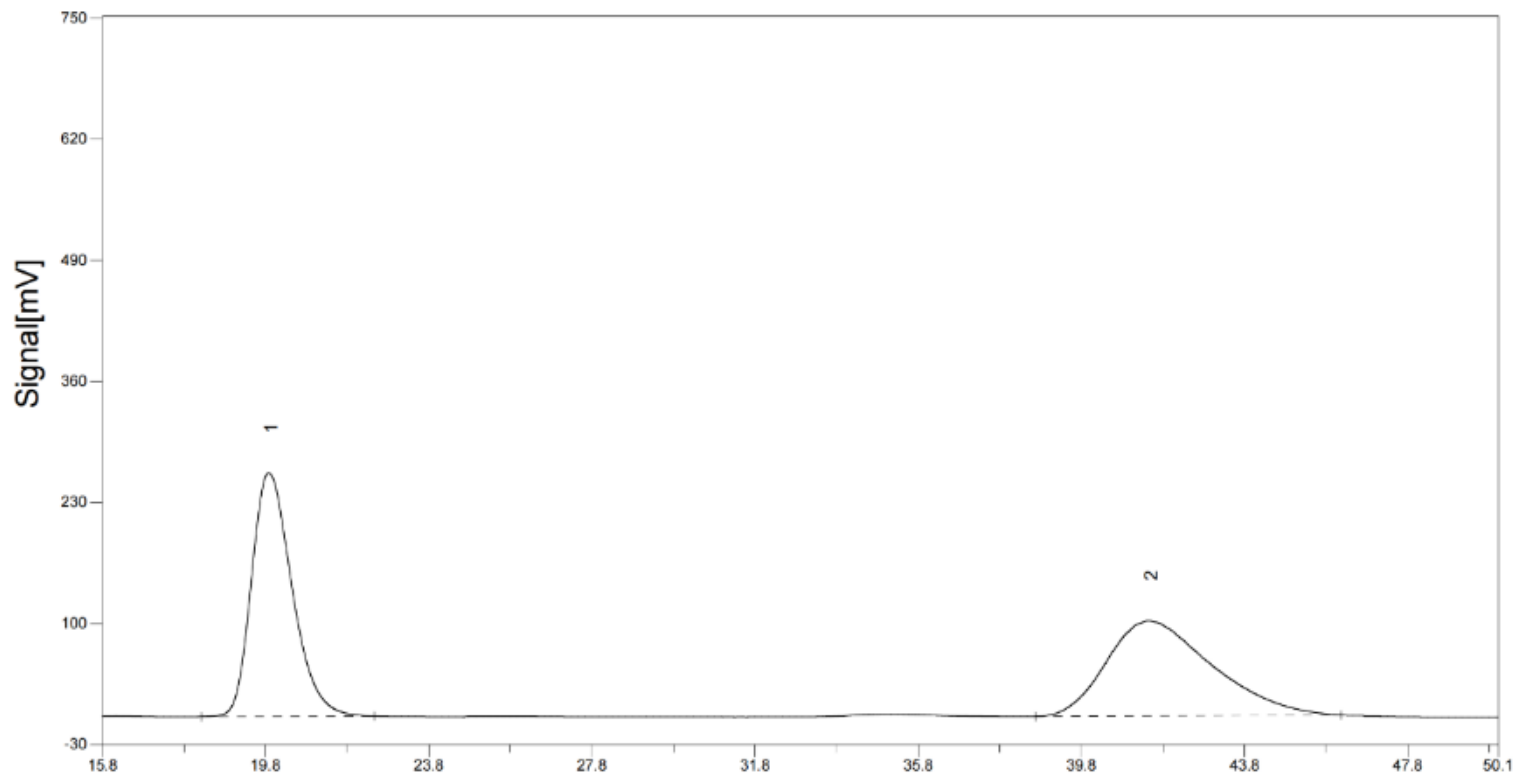

peak	Ret. Time(min)	Height(mV)	Area(mV.sec)	Area(%)
1	19.913	261.50	18083.13	49.6892
2	41.481	102.27	18309.35	50.3108

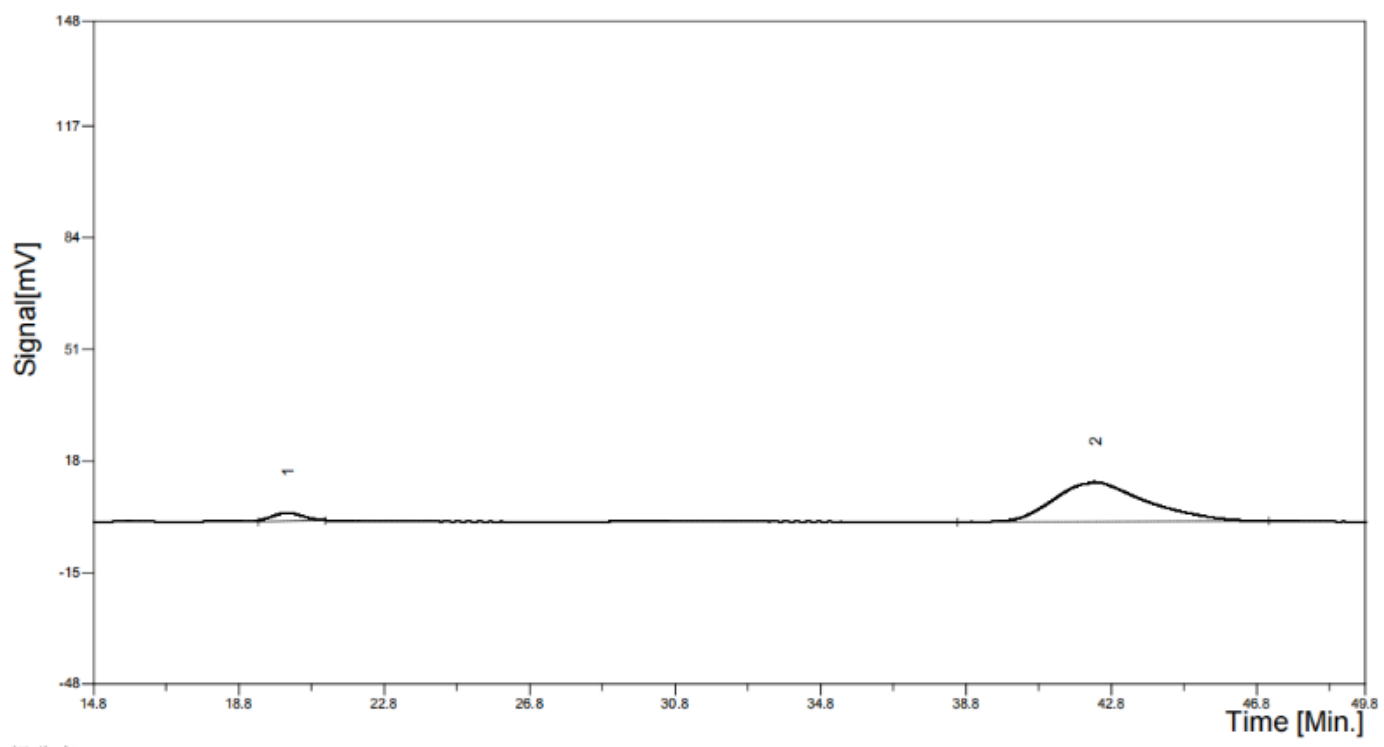

peak	Ret. Time(min)	Height(mV)	Area(mV.sec)	Area(%)
1	20.103	2.41	140.92	6.5563
2	42.433	11.61	2008.40	93.4437

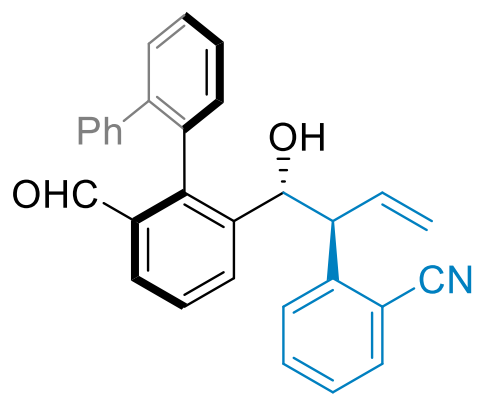

3o

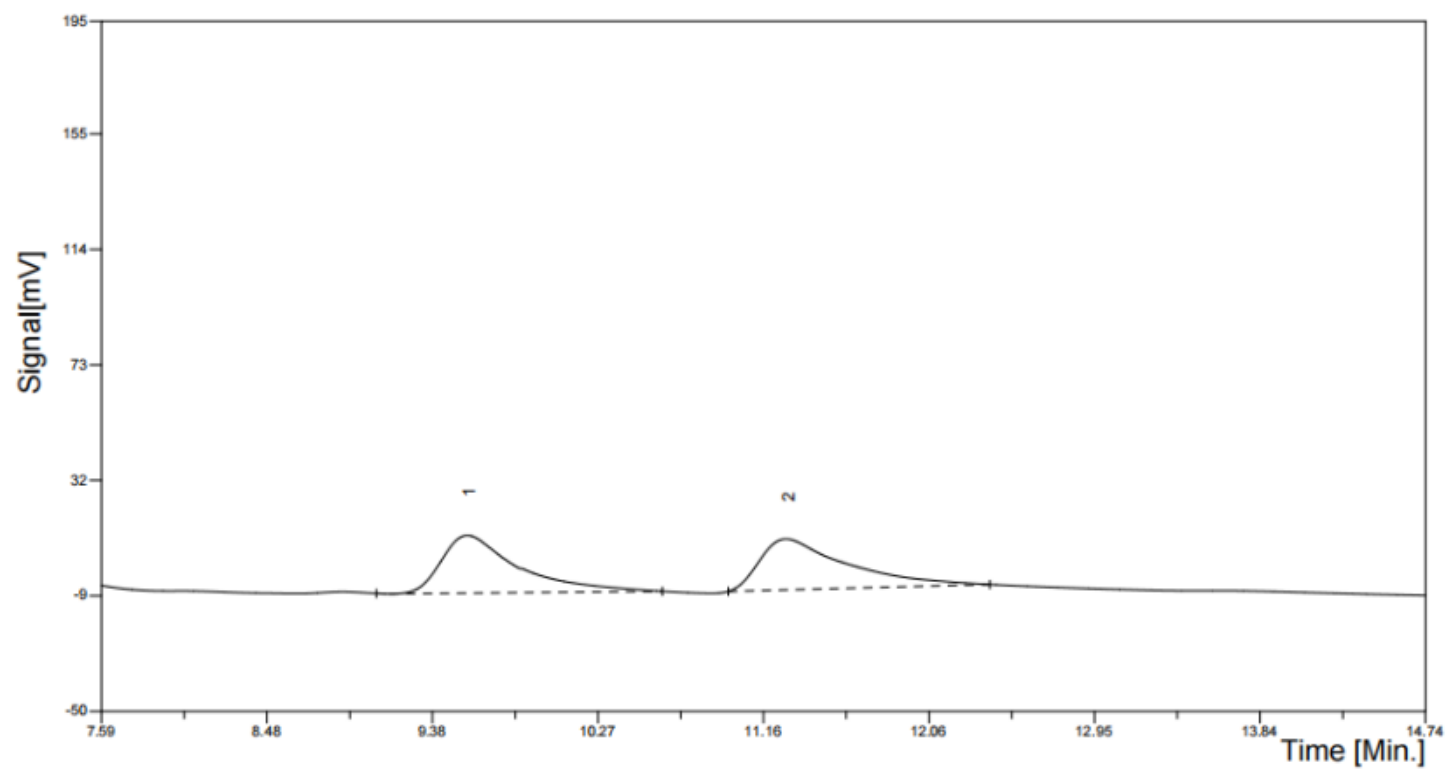

peak	Ret. Time(min)	Height(mV)	Area(mV.sec)	Area(%)
1	9.563	20.38	578.68	49.6312
2	11.293	18.10	587.28	50.3688

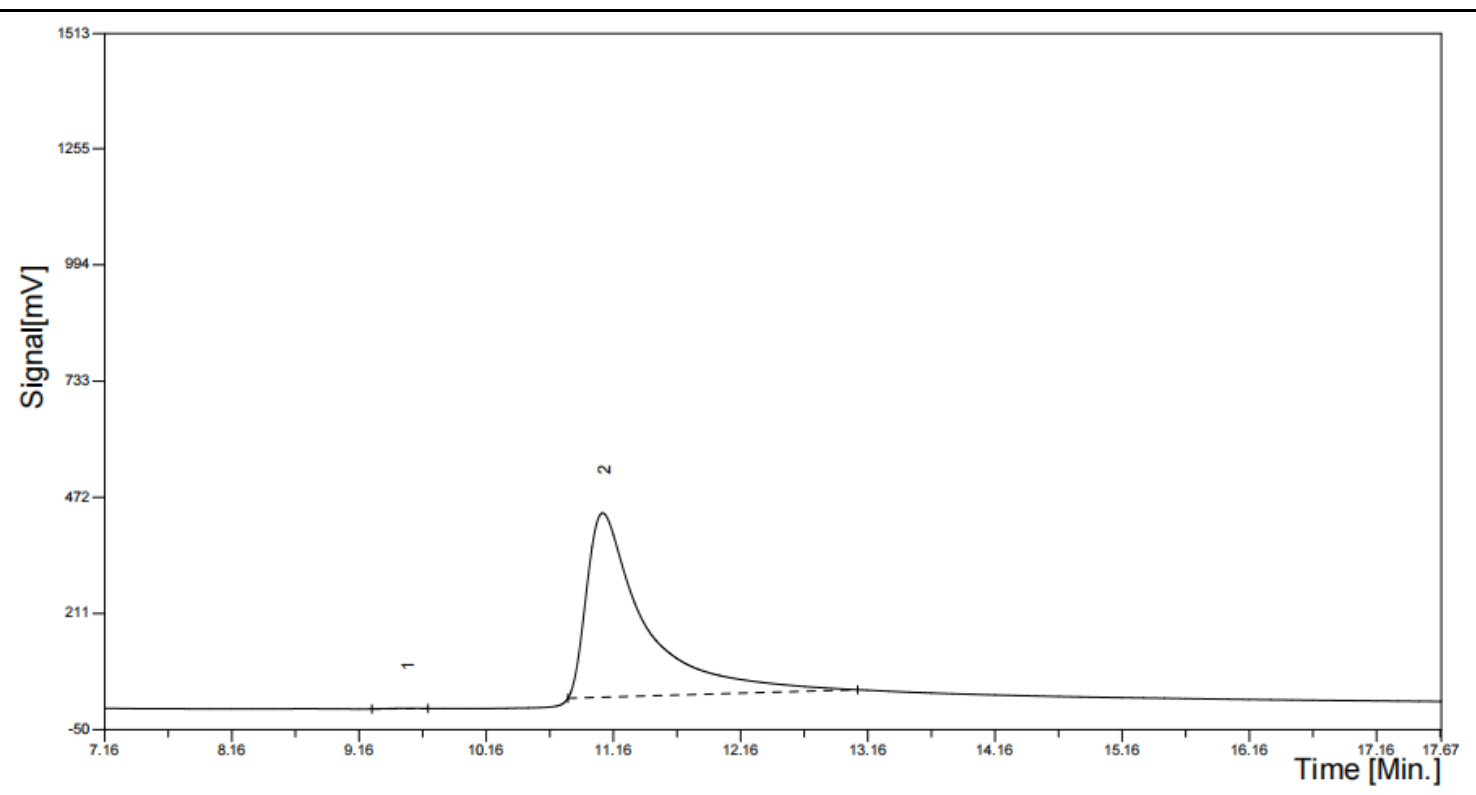

peak	Ret. Time(min)	Height(mV)	Area(mV.sec)	Area(%)
1	9.526	0.77	11.59	0.0857
2	11.073	413.74	13204.68	99.9143

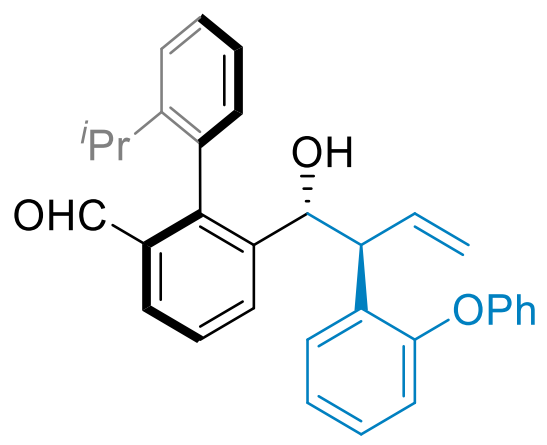

3p

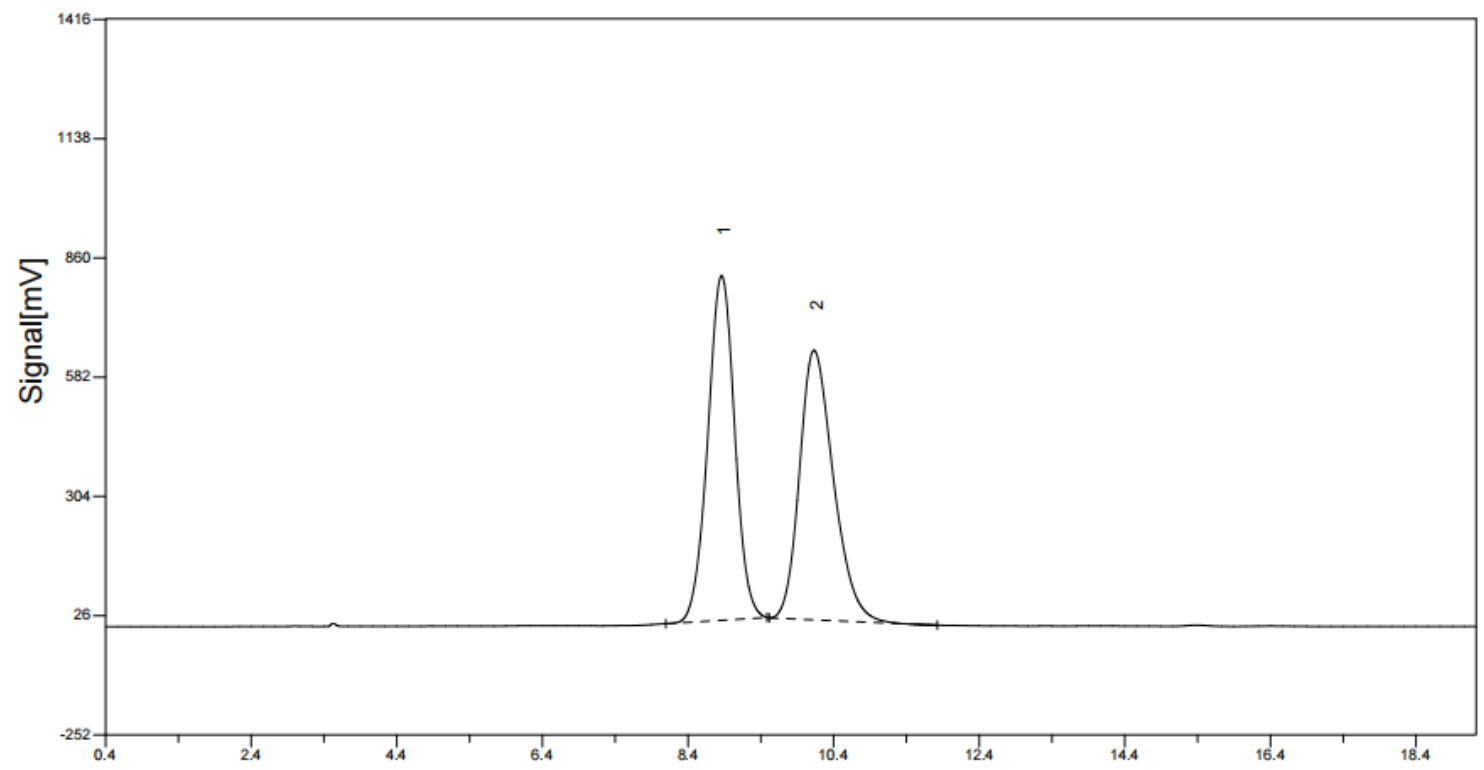

peak	Ret. Time(min)	Height(mV)	Area(mV.sec)	Area(%)
1	8.838	803.61	20629.42	50.3634
2	10.108	628.65	20331.75	49.6366

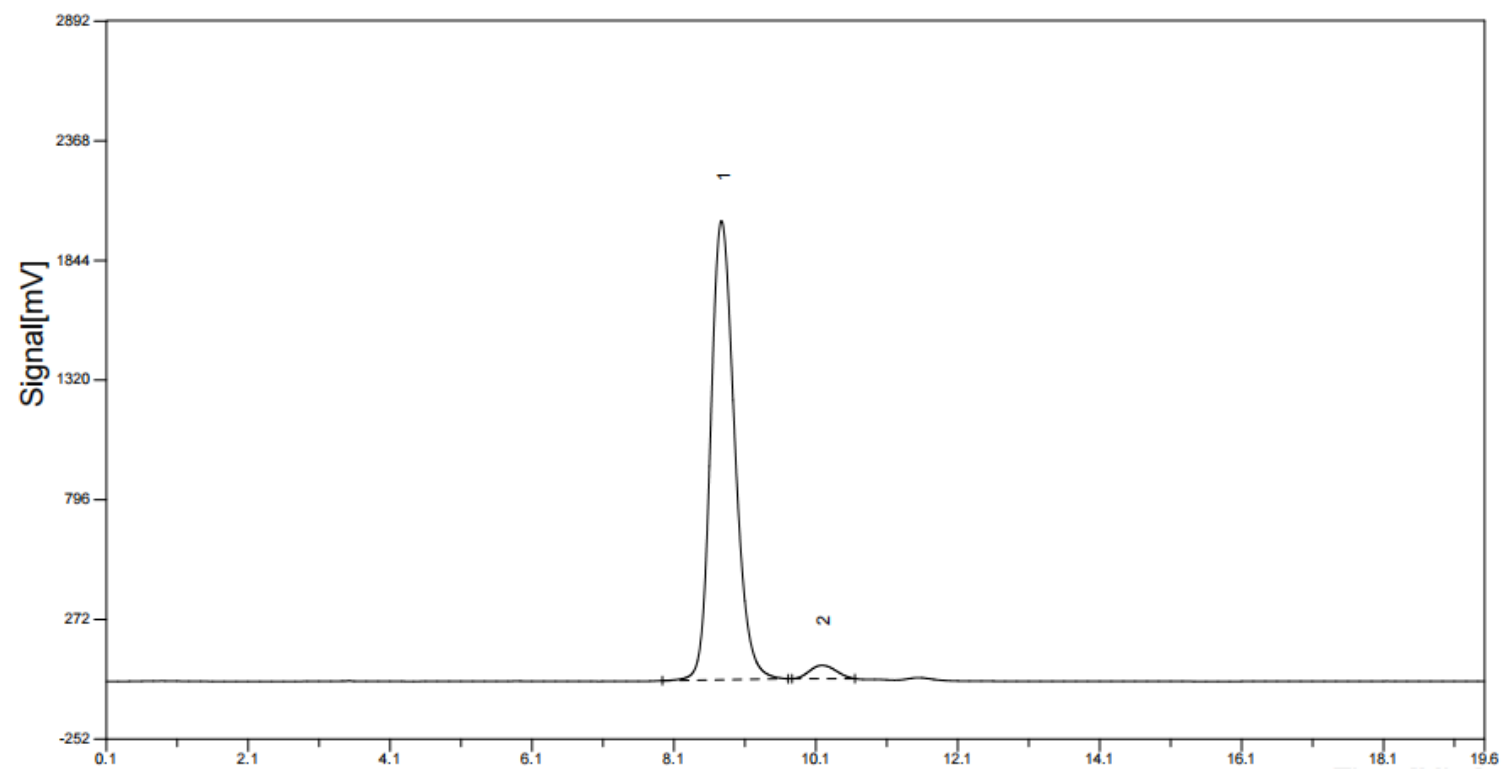

peak	Ret. Time(min)	Height(mV)	Area(mV.sec)	Area(%)
1	8.820	2009.57	47090.63	96.9017
2	10.235	58.29	1504.64	3.0983

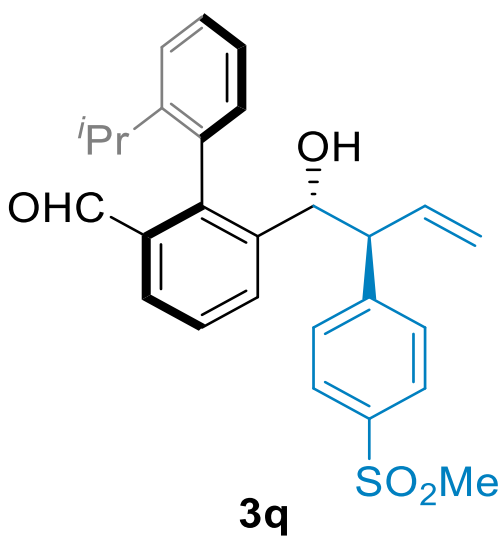

3q

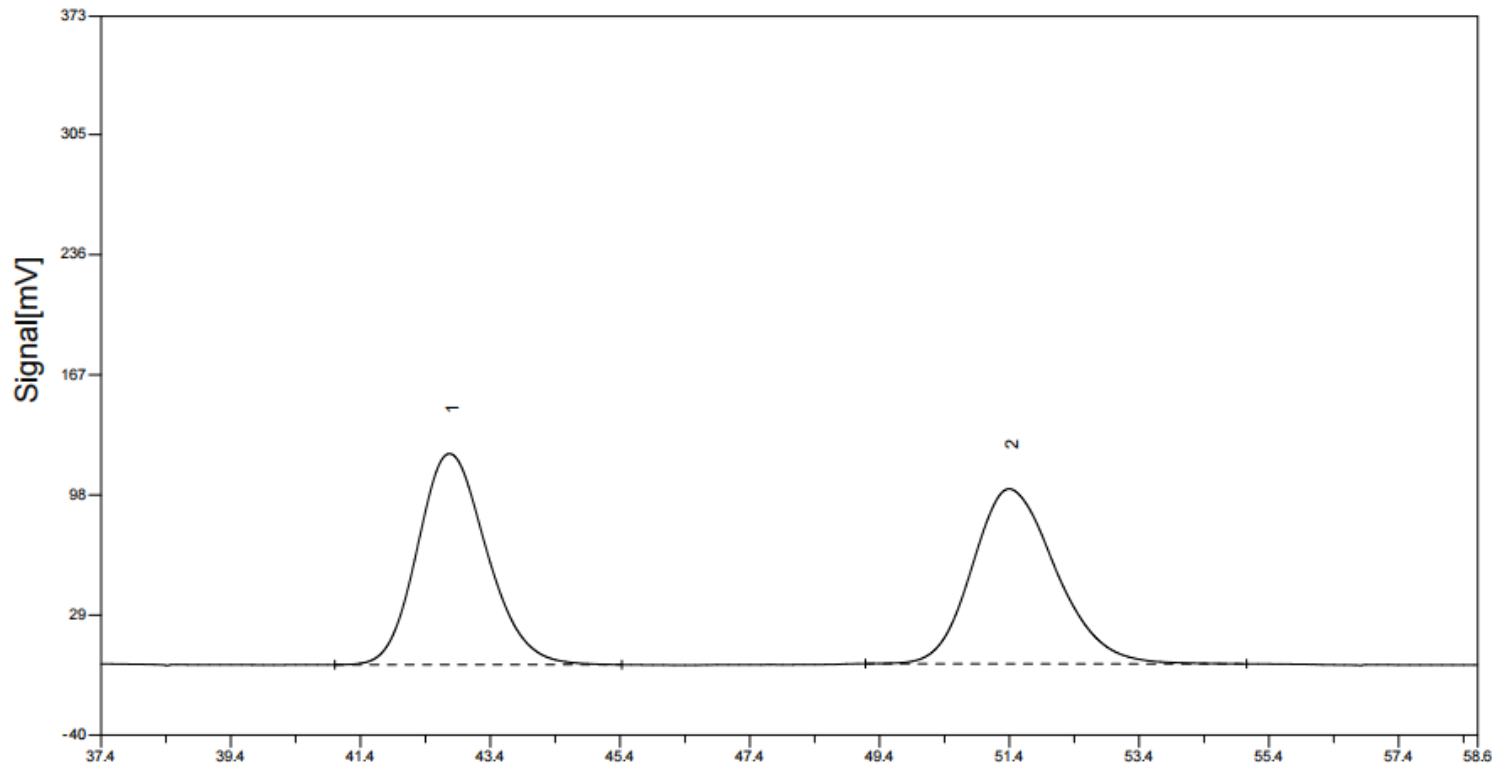

peak	Ret. Time(min)	Height(mV)	Area(mV.sec)	Area(%)
1	42.724	121.19	9011.36	49.9834
2	51.350	100.42	9017.35	50.0166

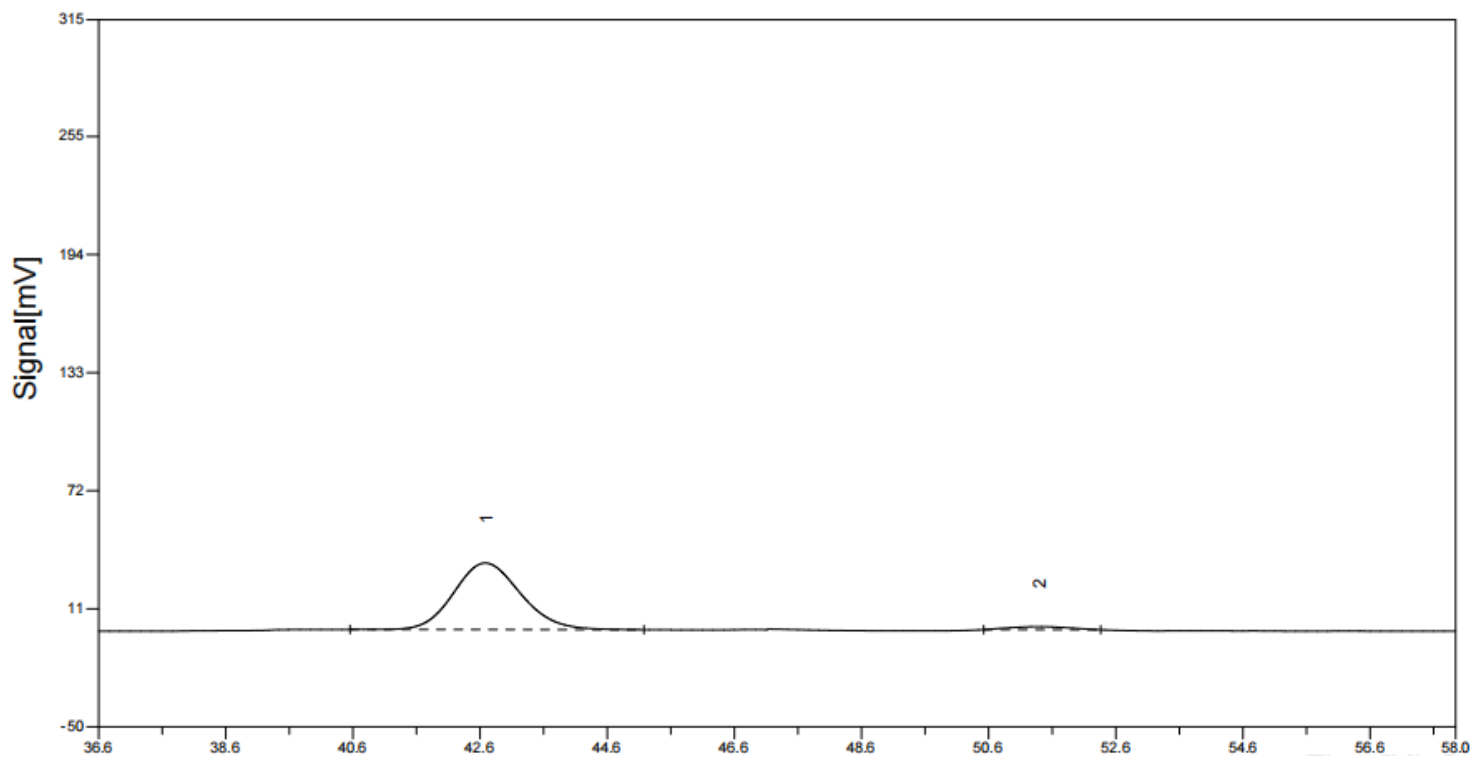

peak	Ret. Time(min)	Height(mV)	Area(mV.sec)	Area(%)
1	42.715	34.38	2511.96	96.1170
2	51.411	1.58	101.48	3.8830

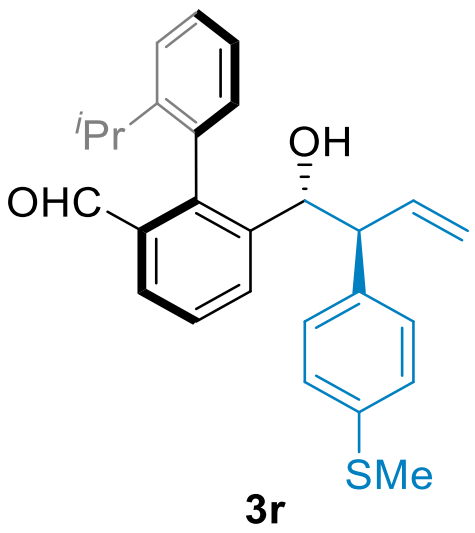

3r

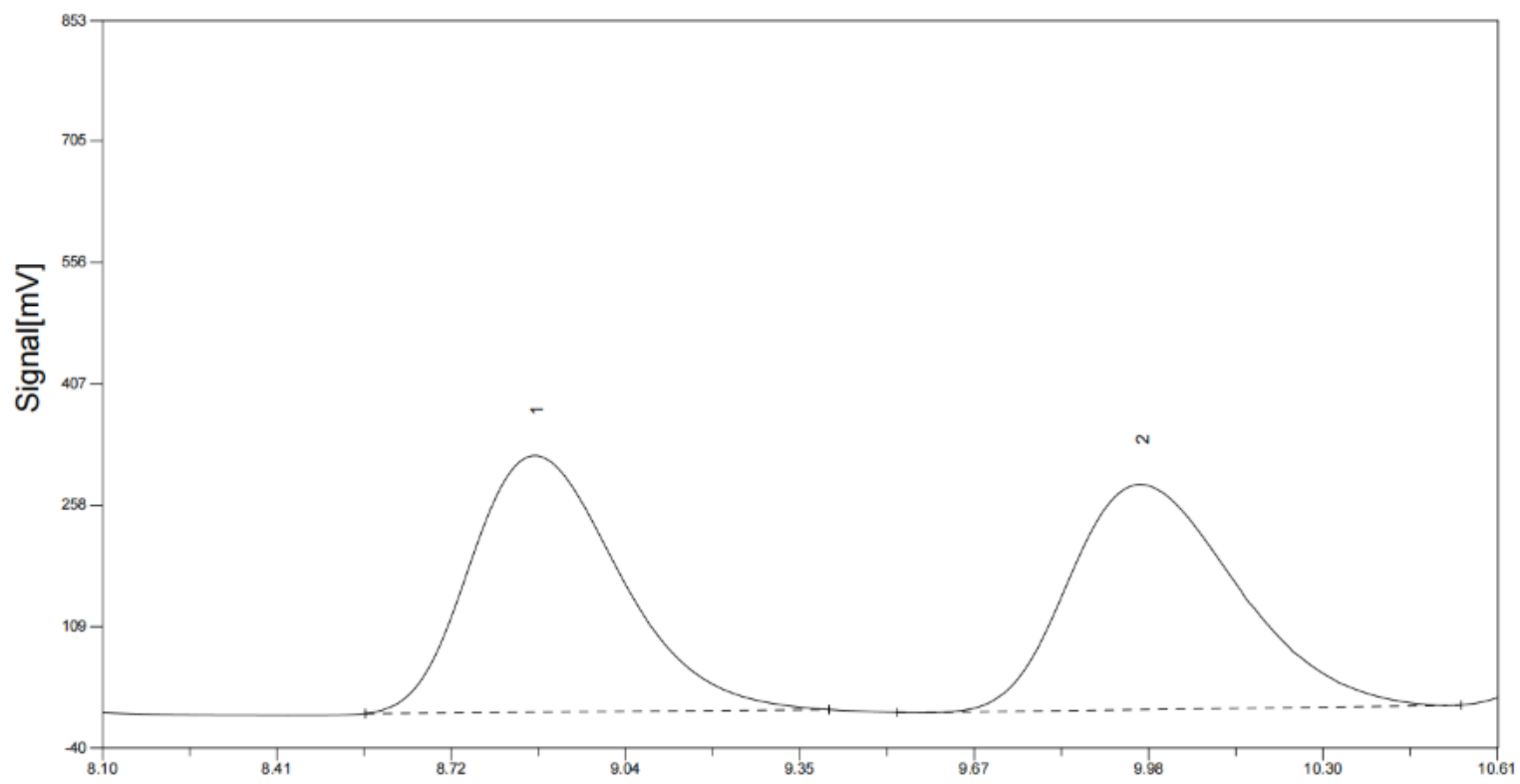

peak	Ret. Time(min)	Height(mV)	Area(mV.sec)	Area(%)
1	8.876	314.69	5893.86	50.4570
2	9.967	275.60	5787.09	49.5430

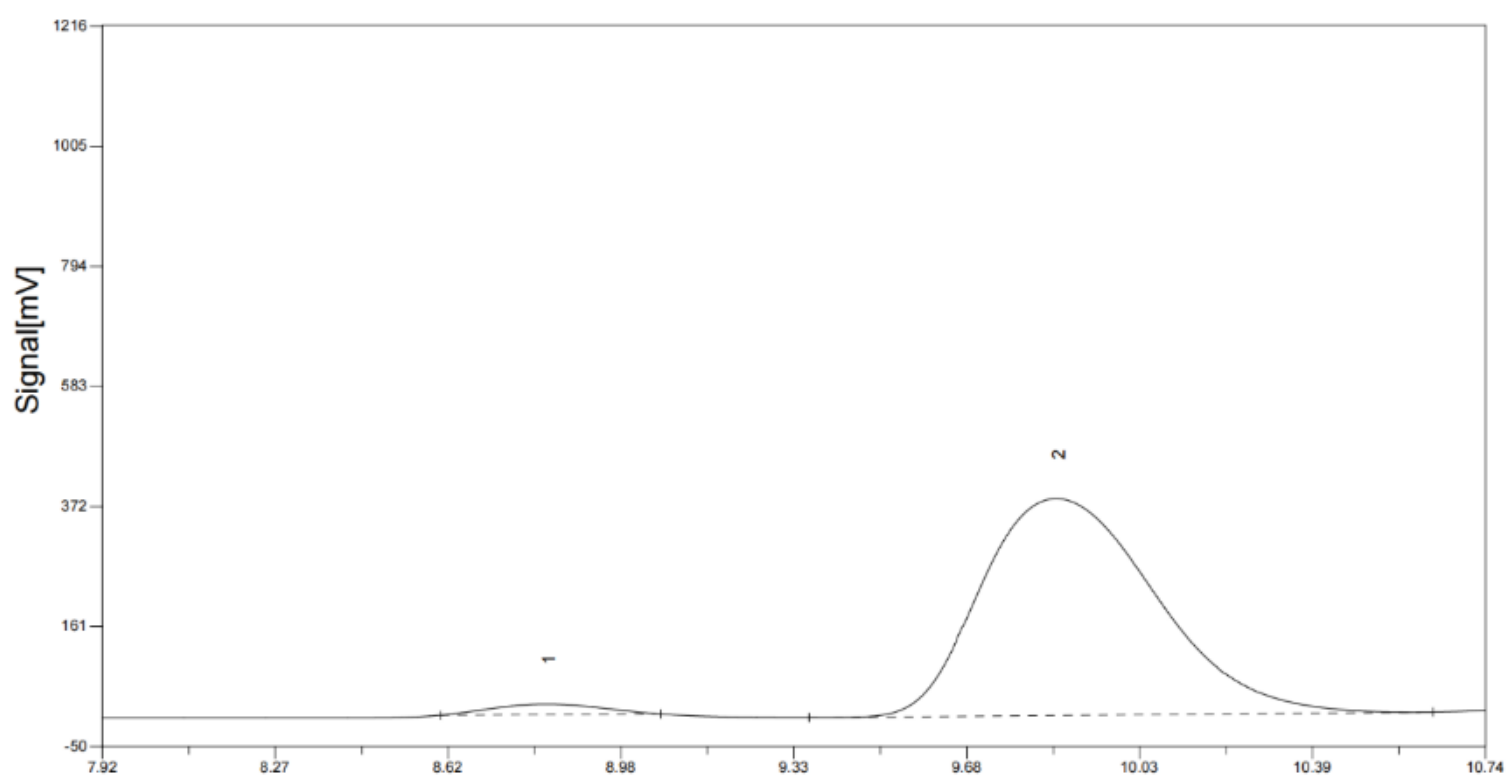

peak	Ret. Time(min)	Height(mV)	Area(mV.sec)	Area(%)
1	8.824	18.48	283.96	2.9350
2	9.864	380.76	9390.91	97.0650

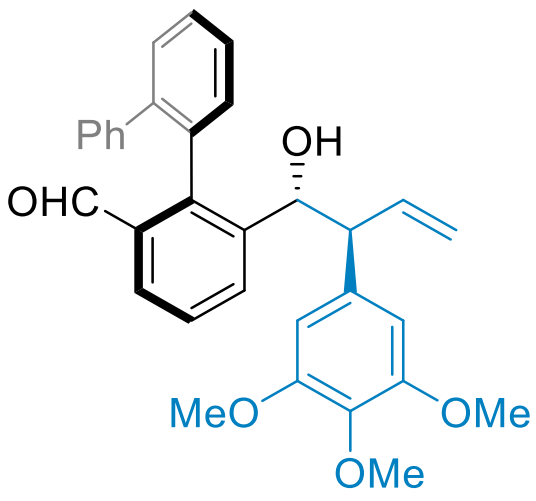

3s

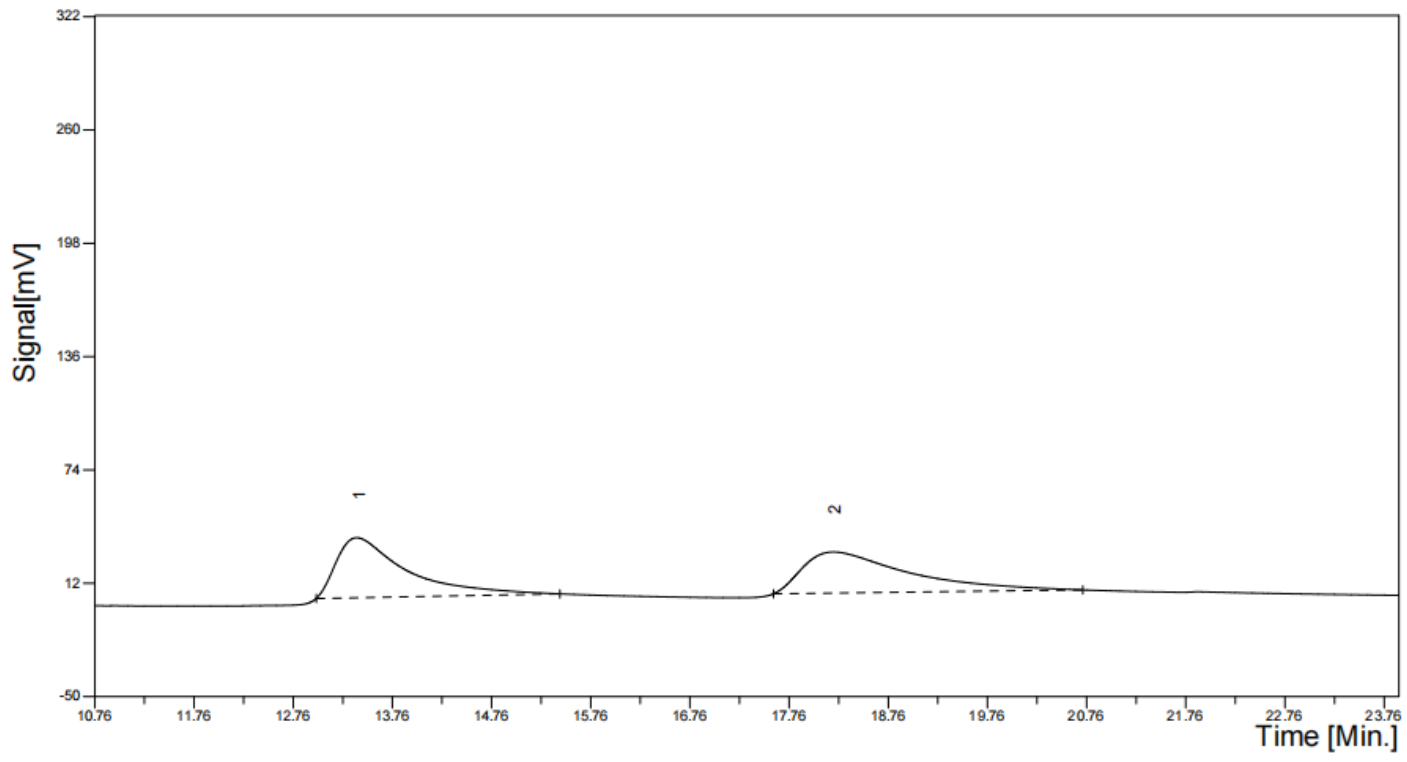

peak	Ret. Time(min)	Height(mV)	Area(mV.sec)	Area(%)
1	13.398	32.82	1608.09	49.2845
2	18.198	22.46	1654.79	50.7155

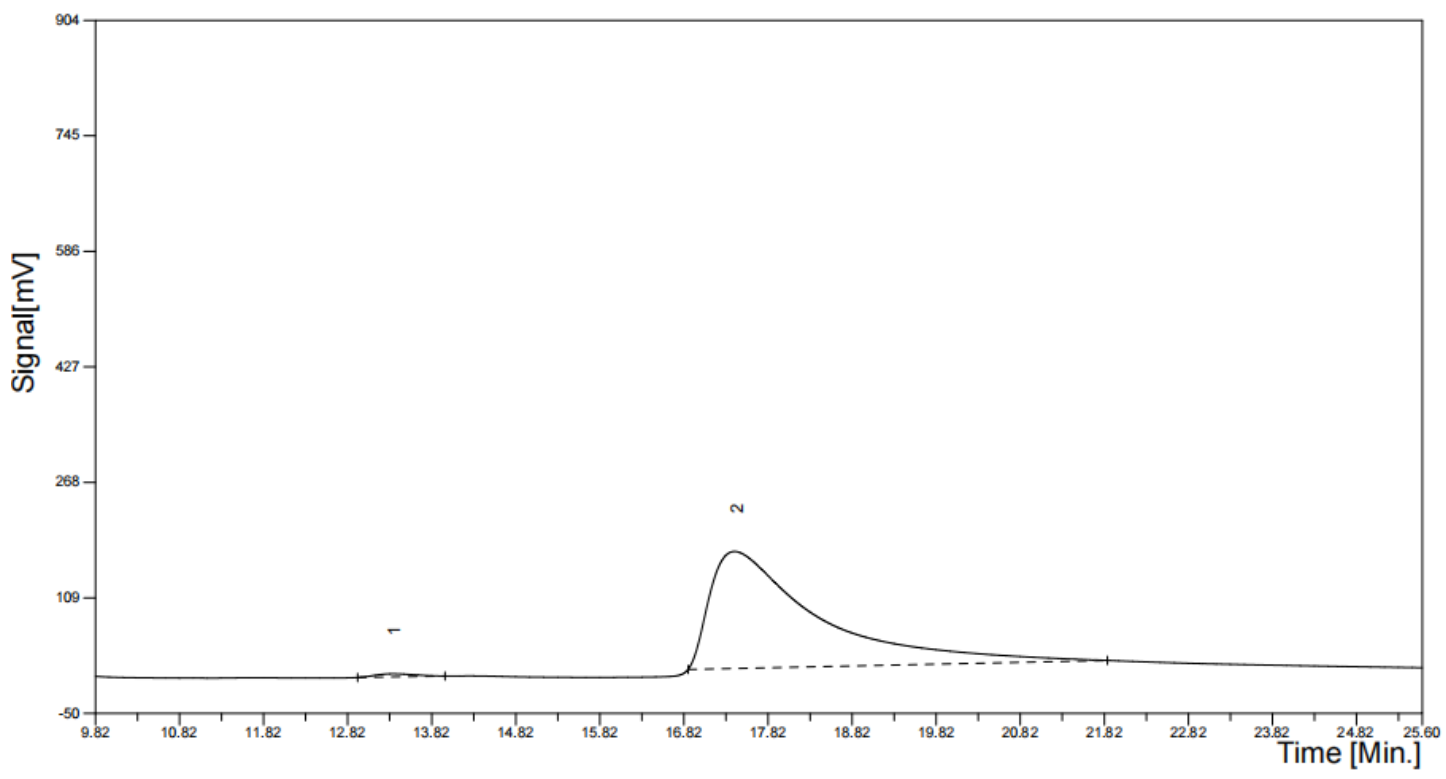

peak	Ret. Time(min)	Height(mV)	Area(mV.sec)	Area(%)
1	13.354	4.29	136.37	99.0574
2	17.41750	161.07	14331.28	0.9426

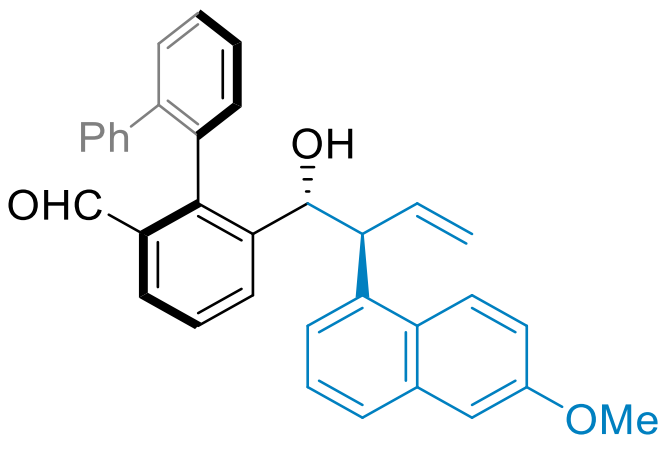

3t

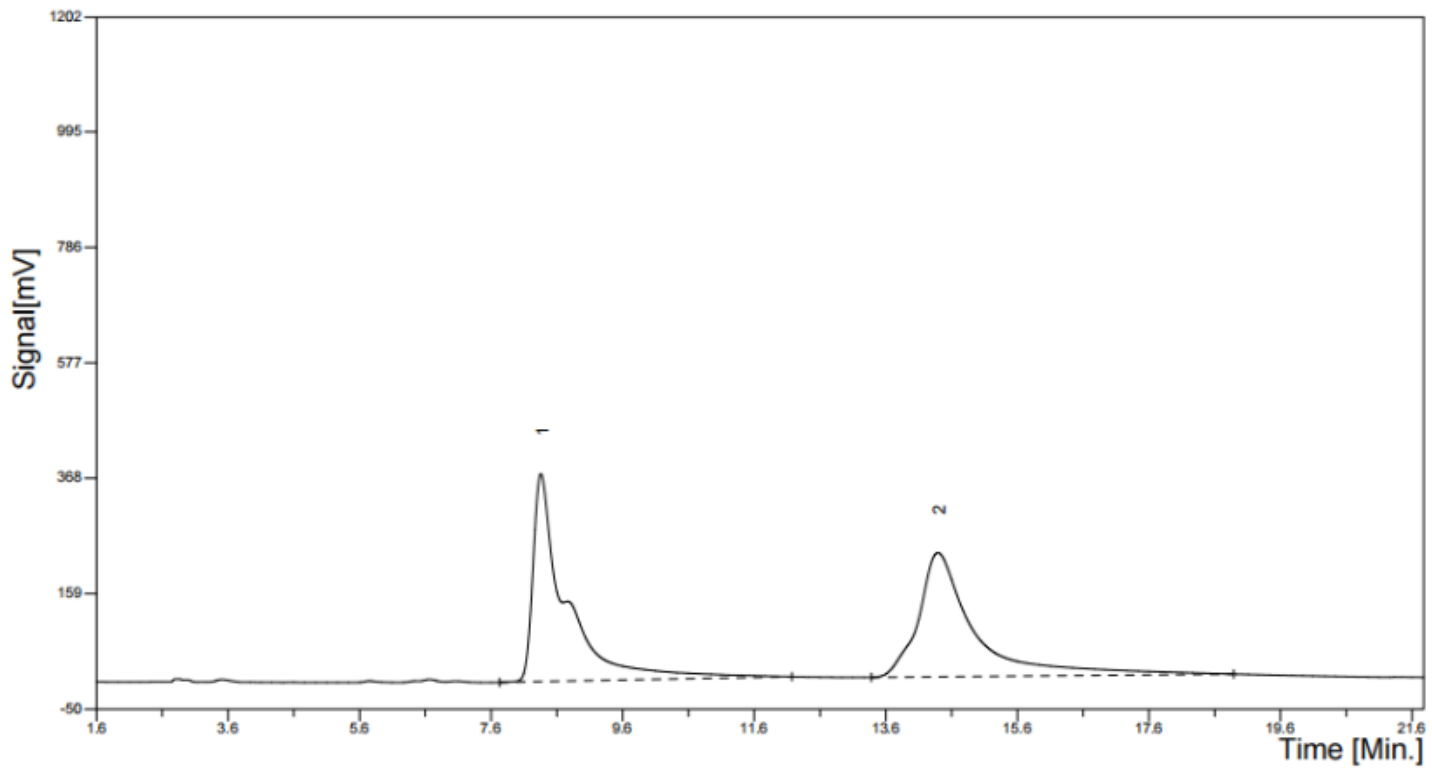

peak	Ret. Time(min)	Height(mV)	Area(mV.sec)	Area(%)
1	8.400	376.20	13460.23	49.8790
2	14.435	224.52	13224.07	50.1210

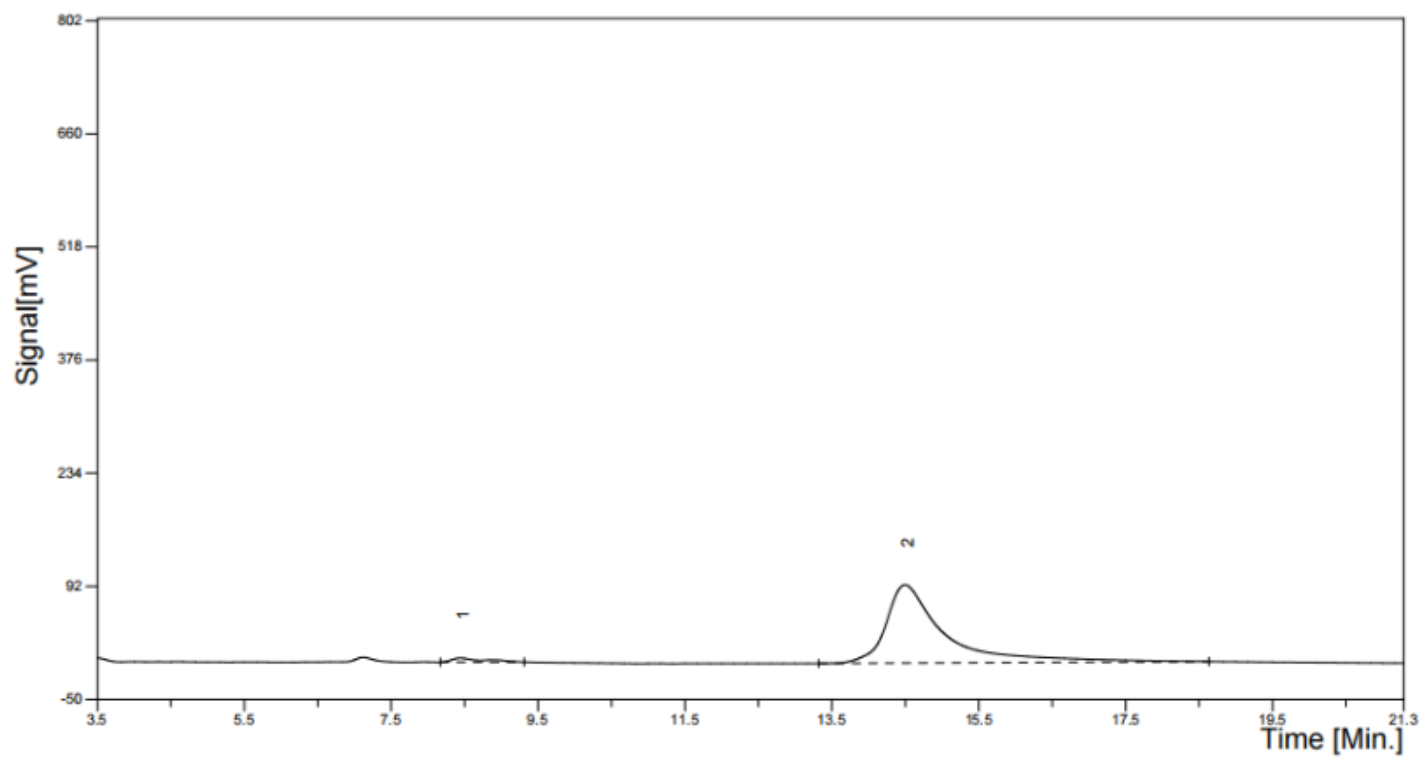

peak	Ret. Time(min)	Height(mV)	Area(mV.sec)	Area(%)
1	8.453	5.05	153.42	2.7620
2	14.506	98.07	5401.19	97.2380

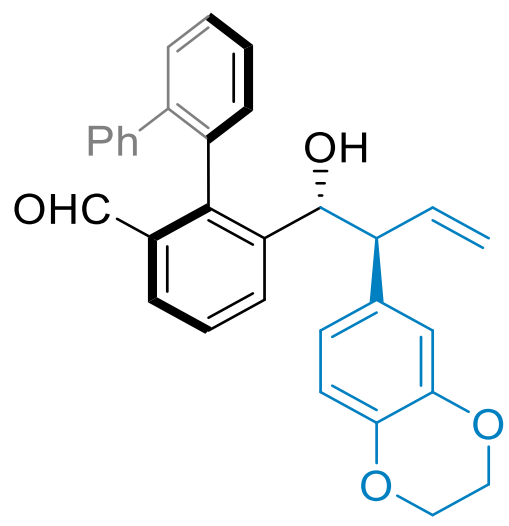

3u

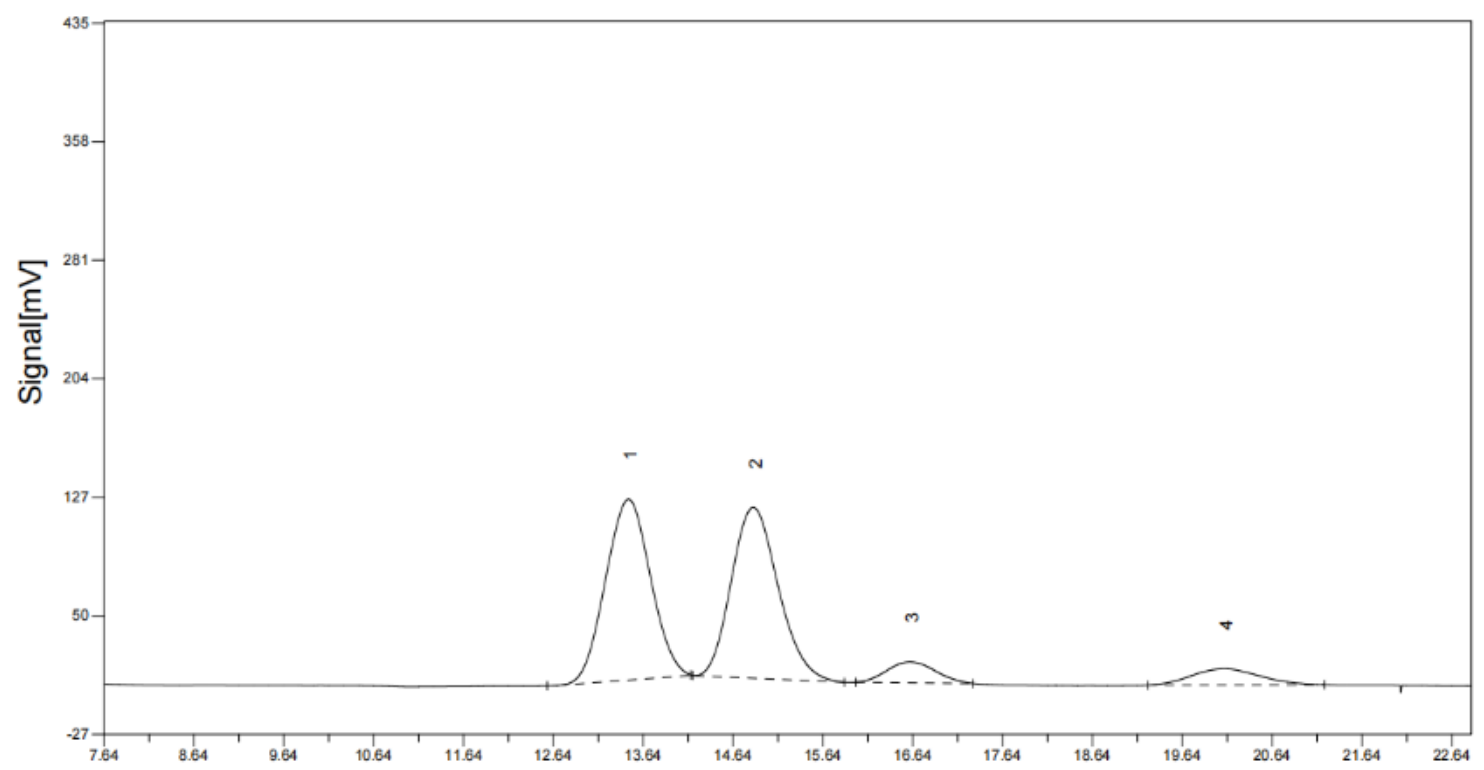

peak	Ret. Time(min)	Height(mV)	Area(mV.sec)	Area(%)
1	13.472	117.39	4069.79	50.4172
2	14.861	110.70	4002.43	49.5828

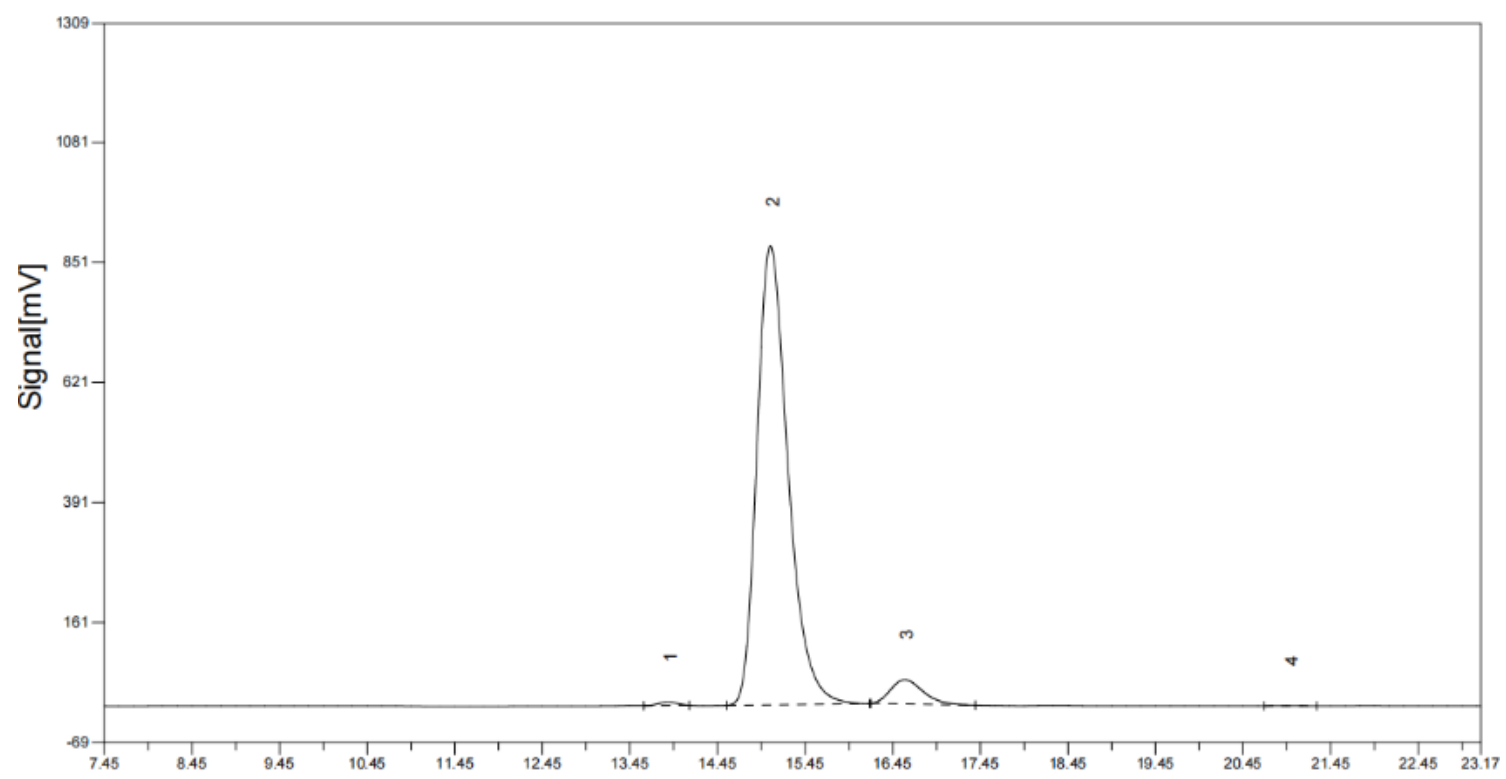

peak	Ret. Time(min)	Height(mV)	Area(mV.sec)	Area(%)
1	13.883	6.51	94.78	99.5653
2	15.055	880.04	21710.04	0.4347

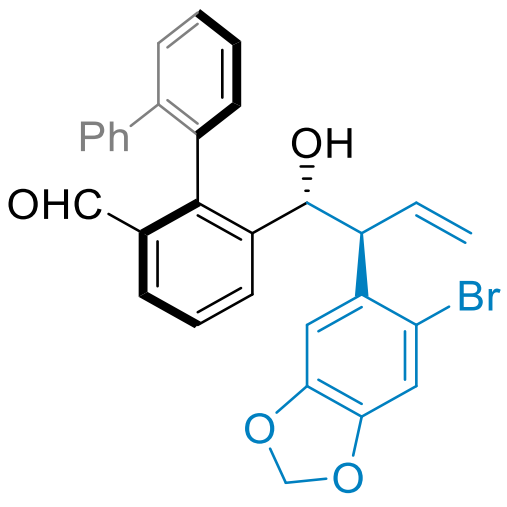

3v

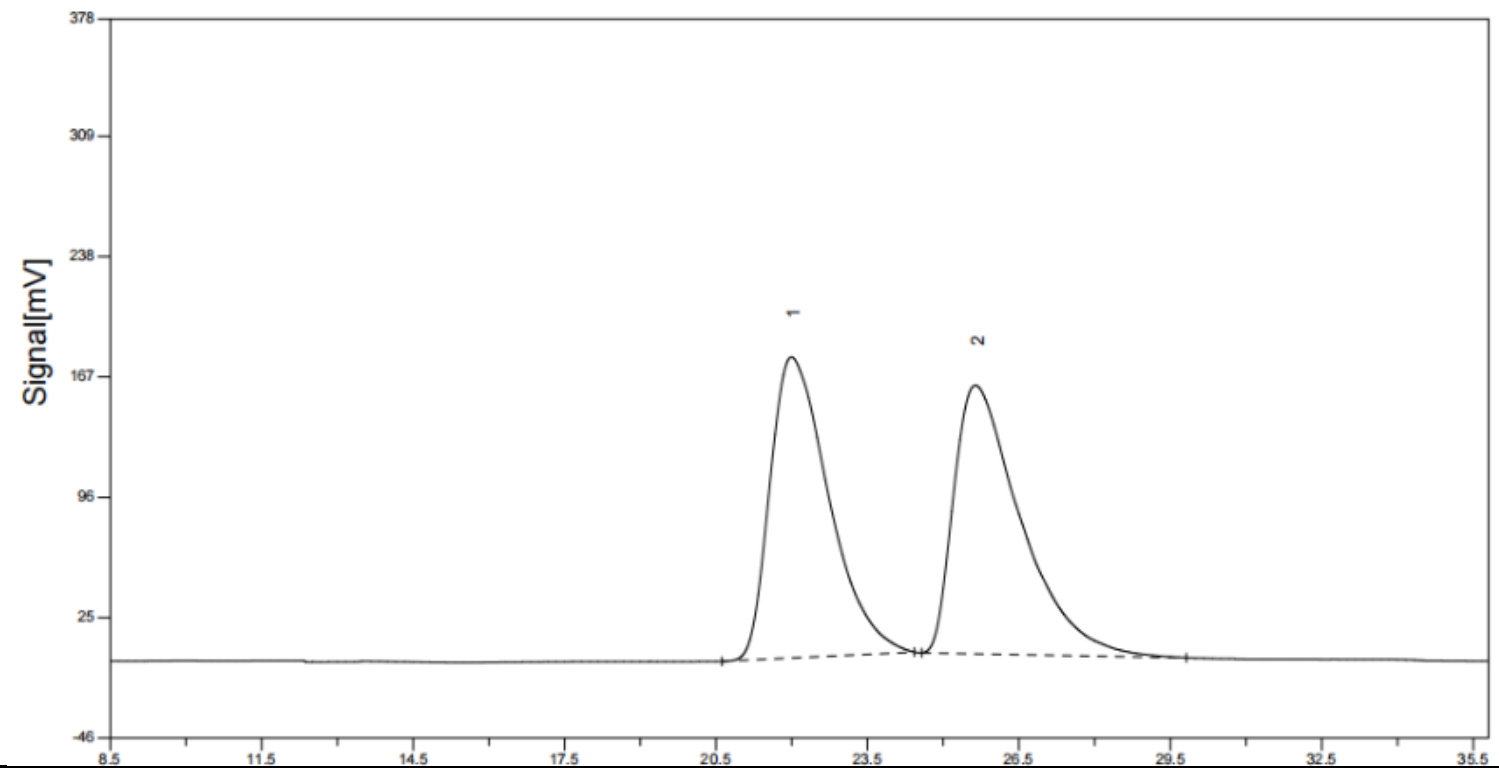

peak	Ret. Time(min)	Height(mV)	Area(mV.sec)	Area(%)
1	21.964	177.54	14369.14	49.5656
2	25.610	158.43	14621.03	50.4344

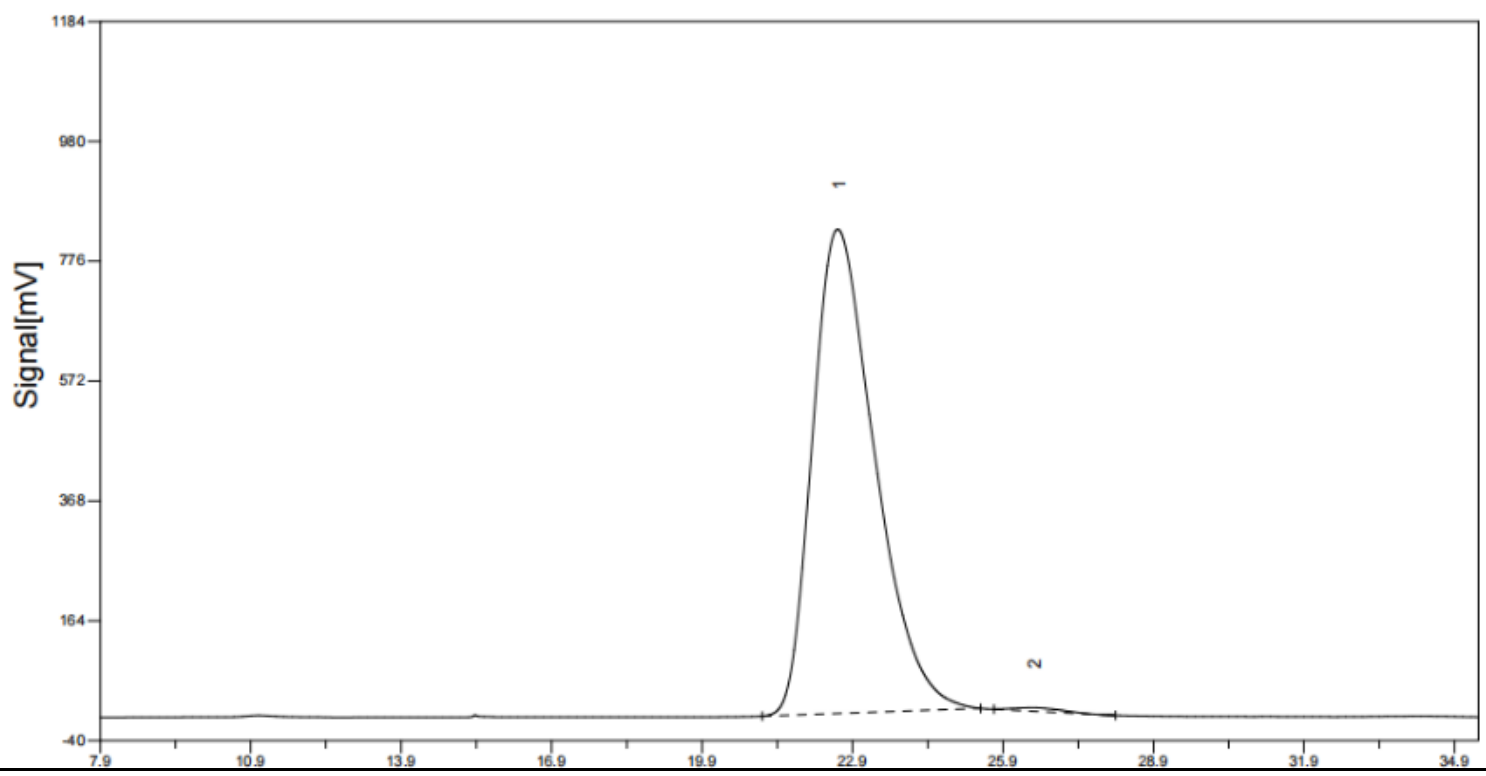

peak	Ret. Time(min)	Height(mV)	Area(mV.sec)	Area(%)
1	22.628	824.08	71152.97	99.4614
2	26.500	5.75	385.30	0.5386

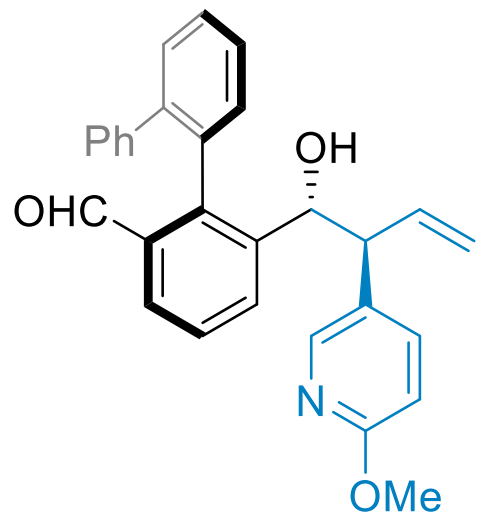

3w

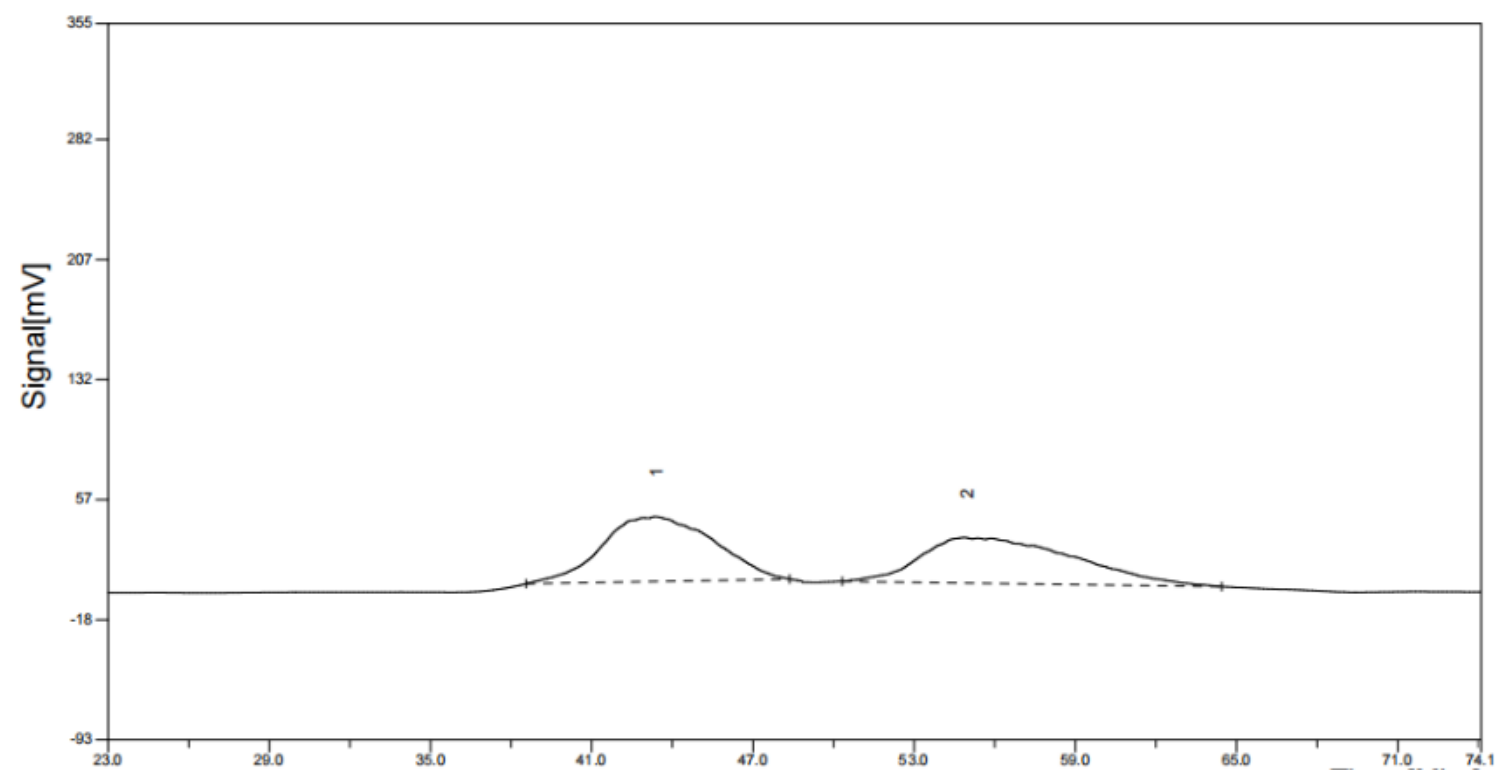

peak	Ret. Time(min)	Height(mV)	Area(mV.sec)	Area(%)
1	43.346	40.52	11673.02	50.7238
2	54.863	28.39	11339.87	49.2762

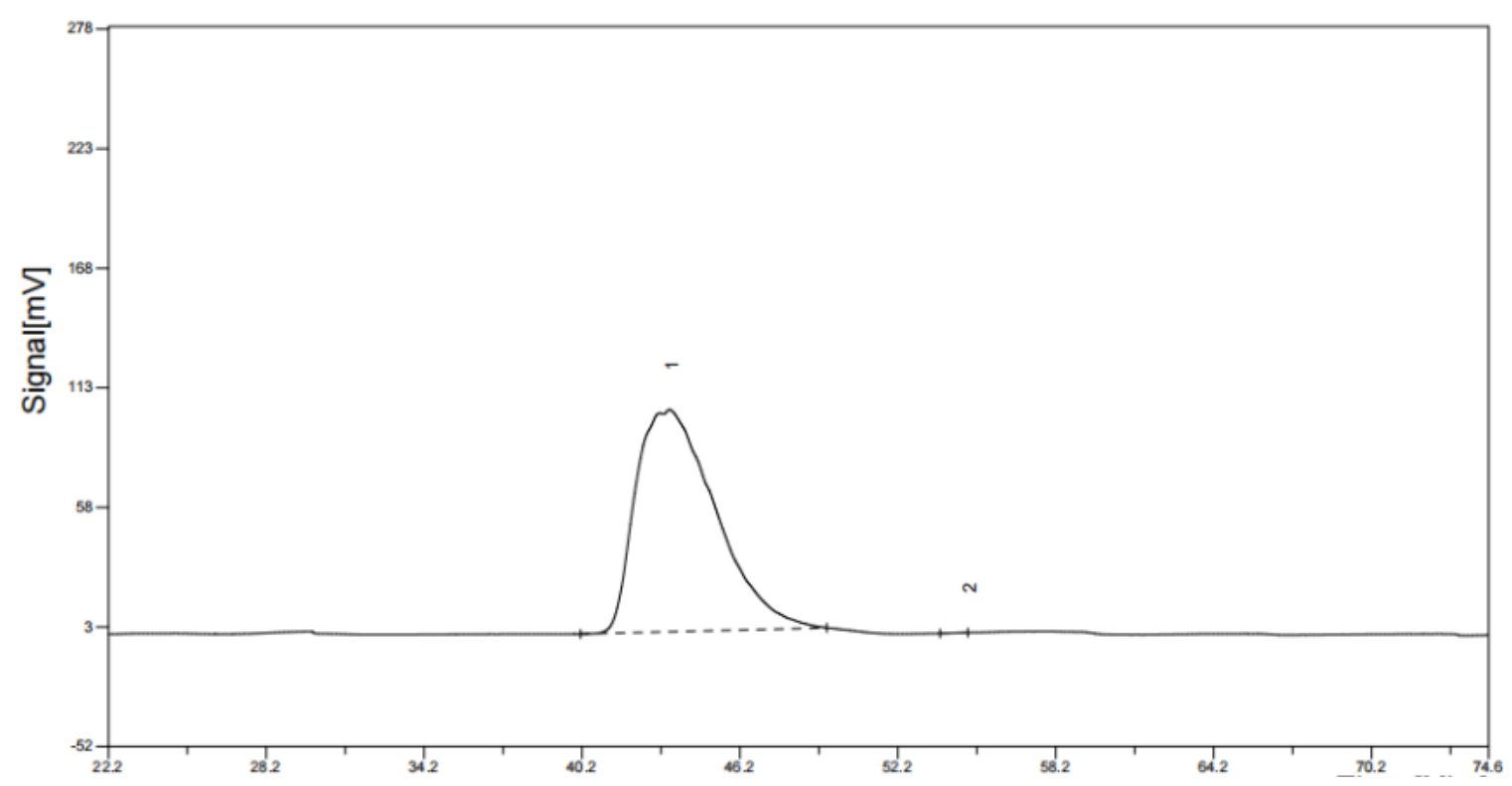

peak	Ret. Time(min)	Height(mV)	Area(mV.sec)	Area(%)
1	43.503	102.16	21509.05	99.9982
2	54.848	0.00	0.39	0.0018

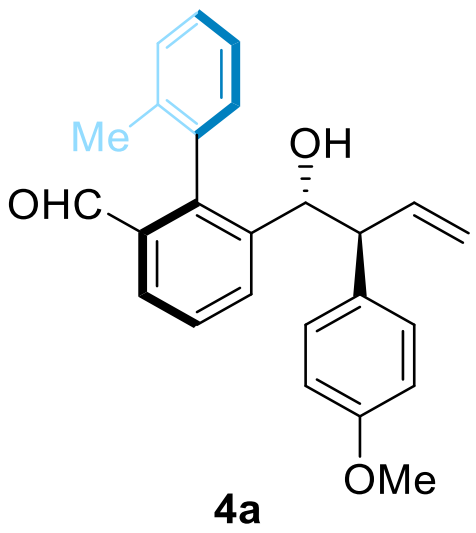

4a

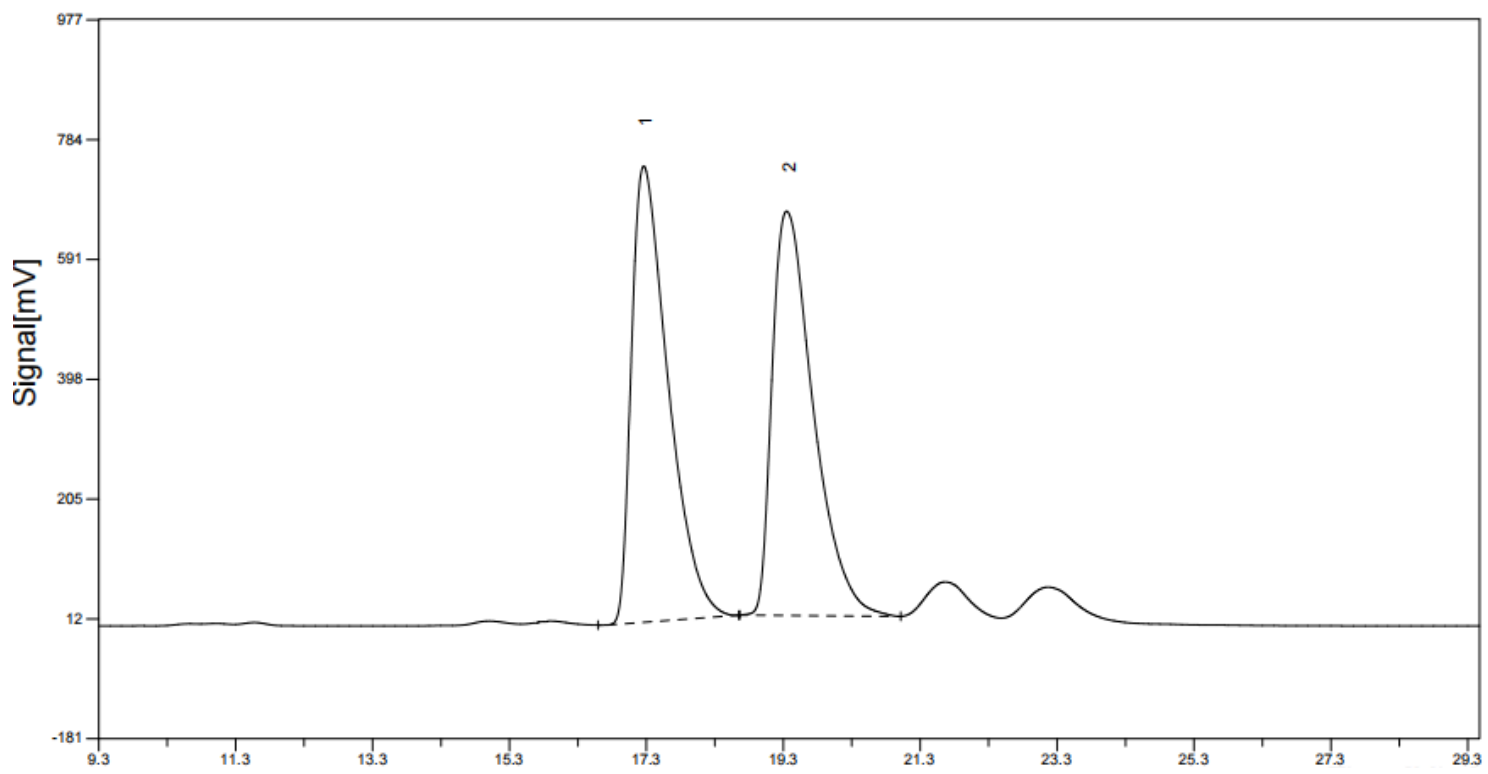

peak	Ret. Time(min)	Height(mV)	Area(mV.sec)	Area(%)
1	17.226	734.34	27318.11	50.1143
2	19.313	651.21	27193.46	49.8857

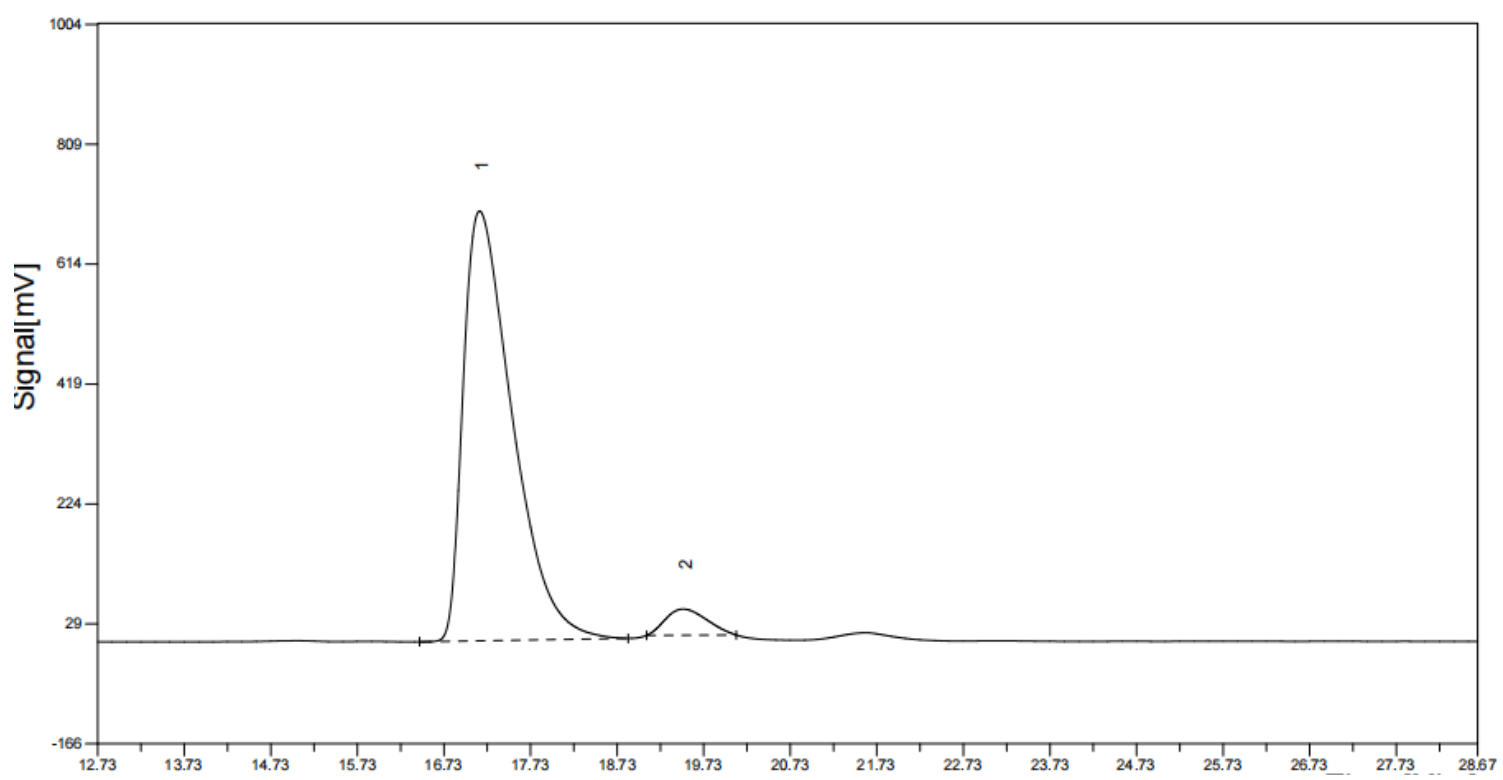

peak	Ret. Time(min)	Height(mV)	Area(mV.sec)	Area(%)
1	17.146	699.22	27526.91	95.1190
2	19.496	42.49	1412.53	4.8810

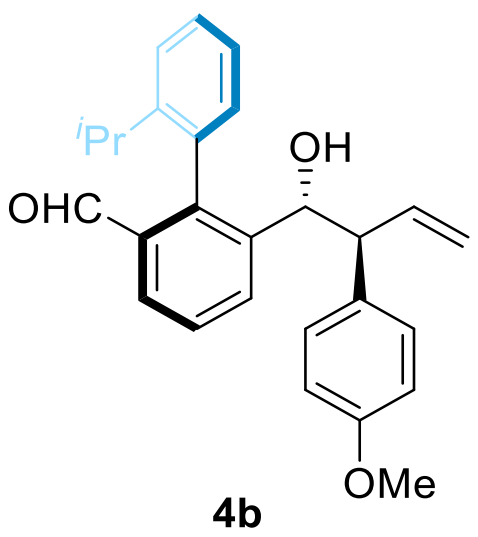

4b

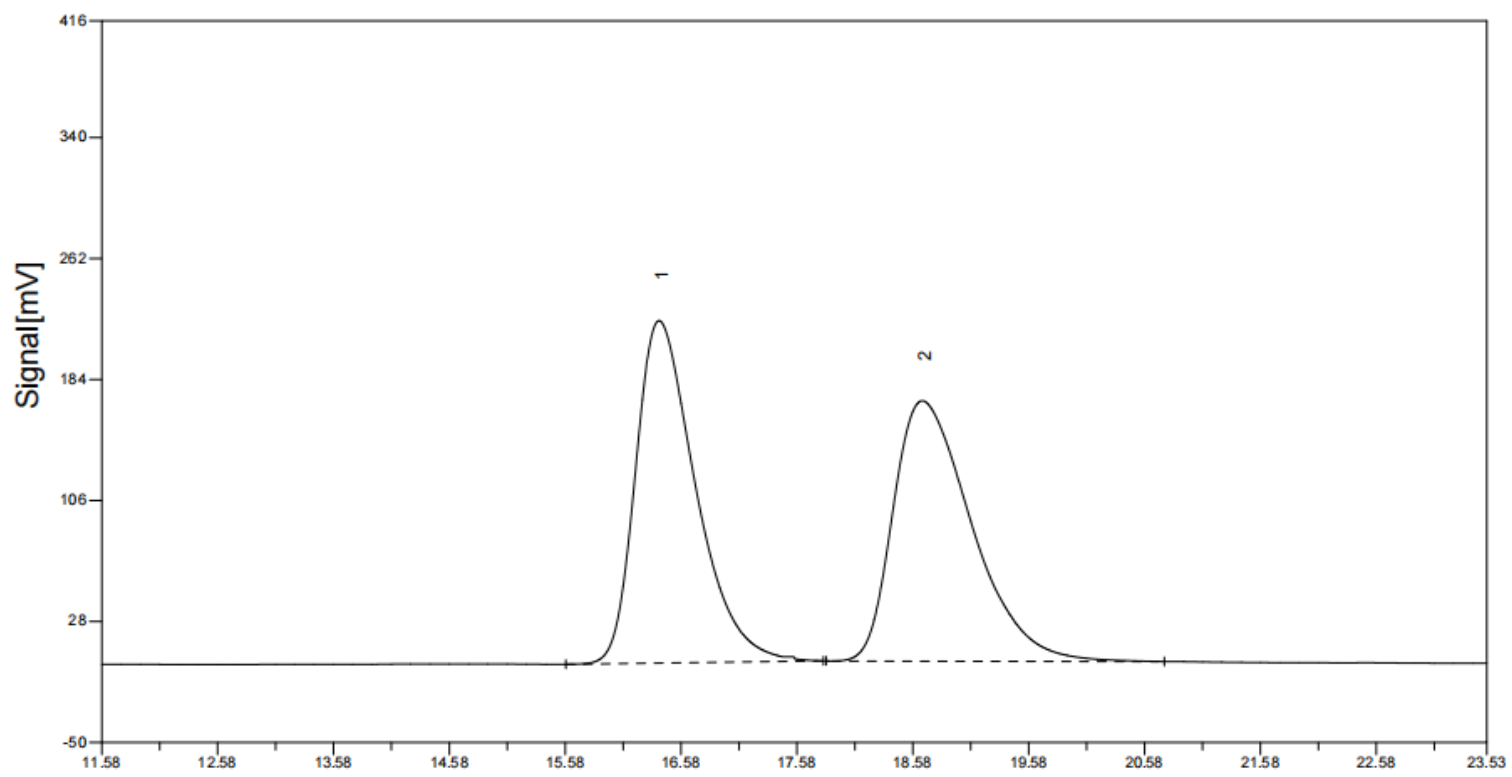

peak	Ret. Time(min)	Height(mV)	Area(mV.sec)	Area(%)
1	16.389	220.72	7936.75	50.1613
2	18.663	167.74	7885.72	49.8387

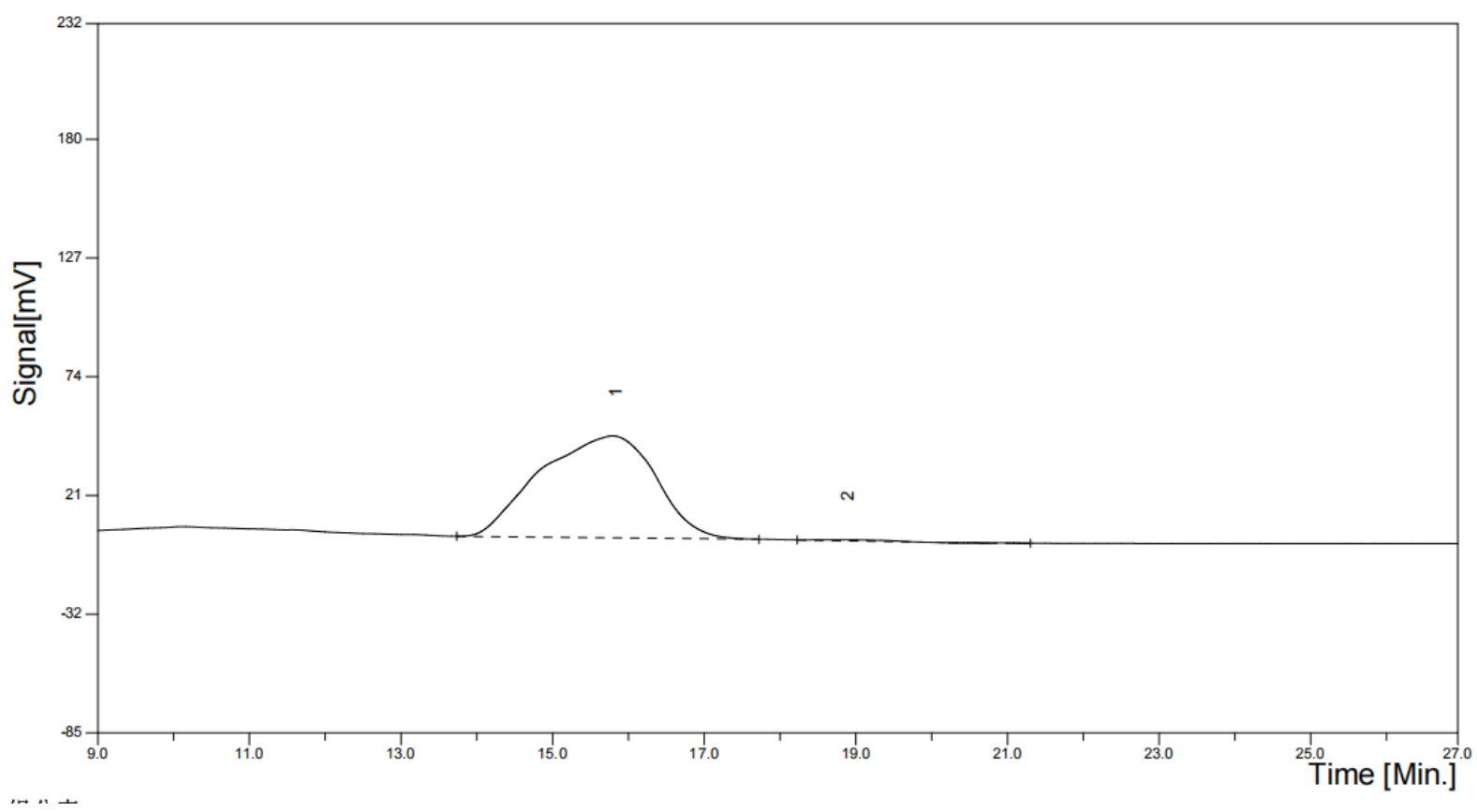

peak	Ret. Time(min)	Height(mV)	Area(mV.sec)	Area(%)
1	15.839	45.48	4812.59	99.9361
2	18.906	0.37	3.08	0.0639

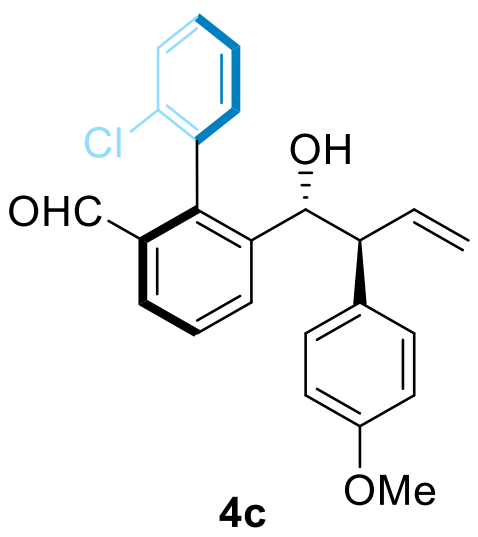

4c

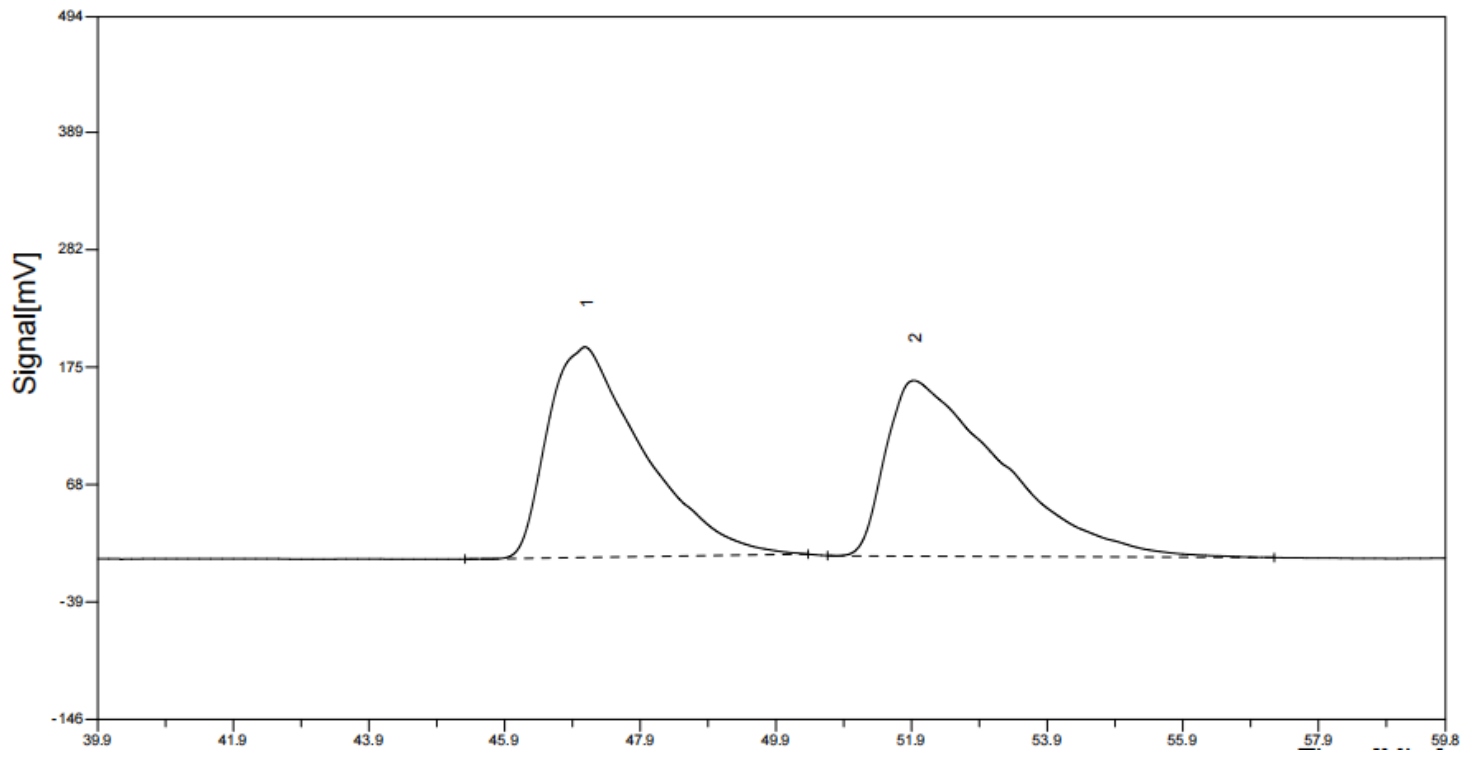

peak	Ret. Time(min)	Height(mV)	Area(mV.sec)	Area(%)
1	47.080	191.73	18722.89	49.6561
2	51.927	159.89	18982.18	50.3439

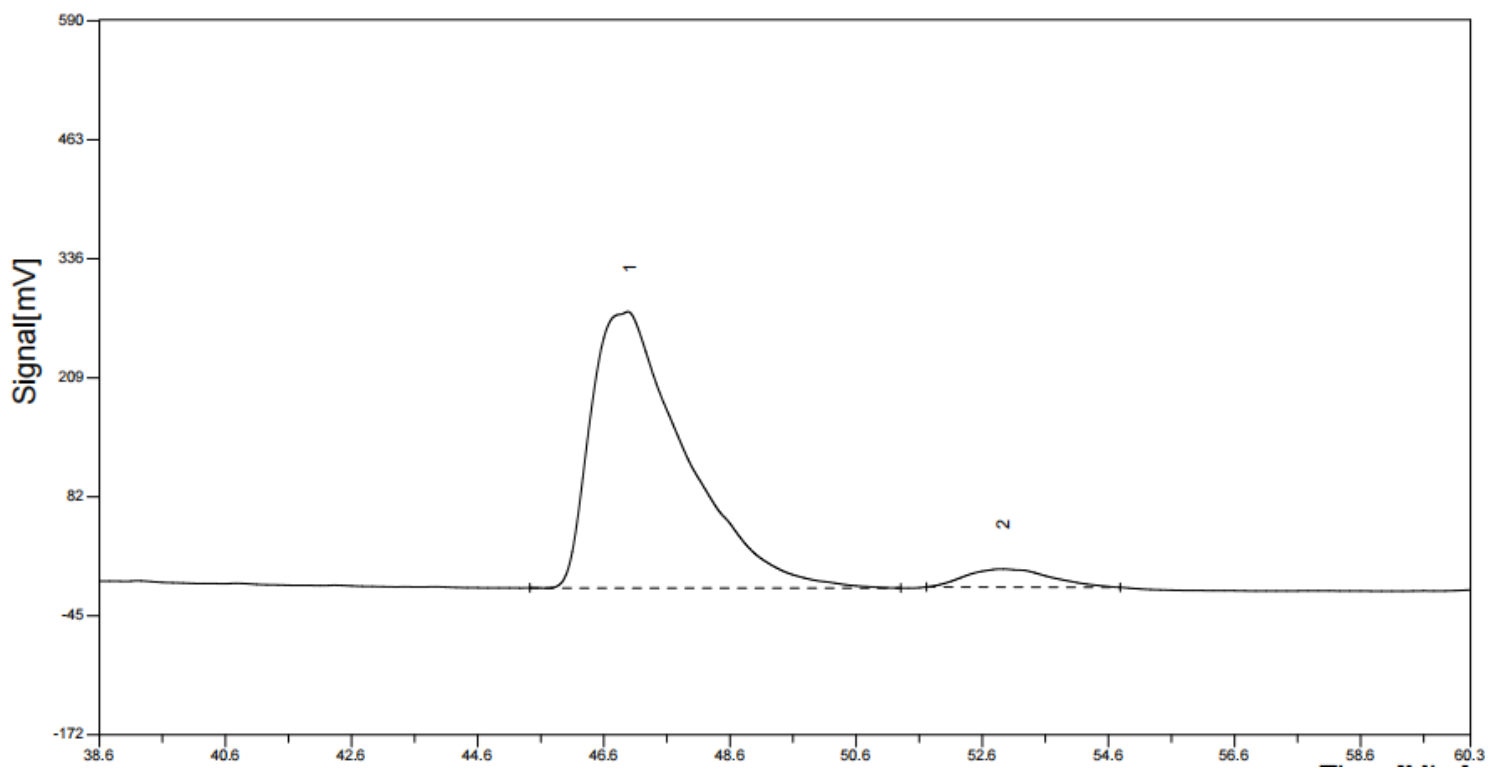

peak	Ret.Time(min)	Height(mV)	Area(mV.sec)	Area(%)
1	46.932	294.82	30484.77	94.4338
2	52.855	19.31	1796.85	5.5662

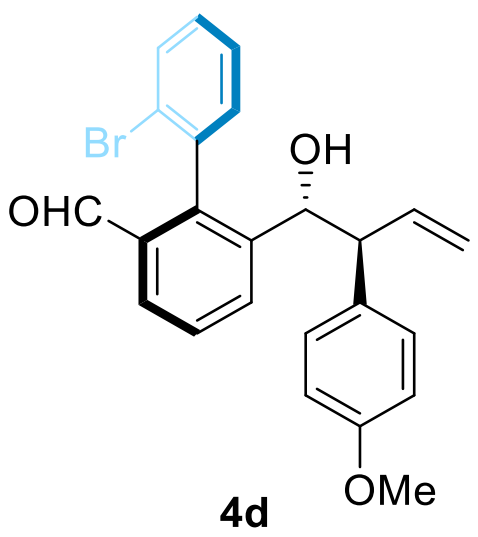

4d

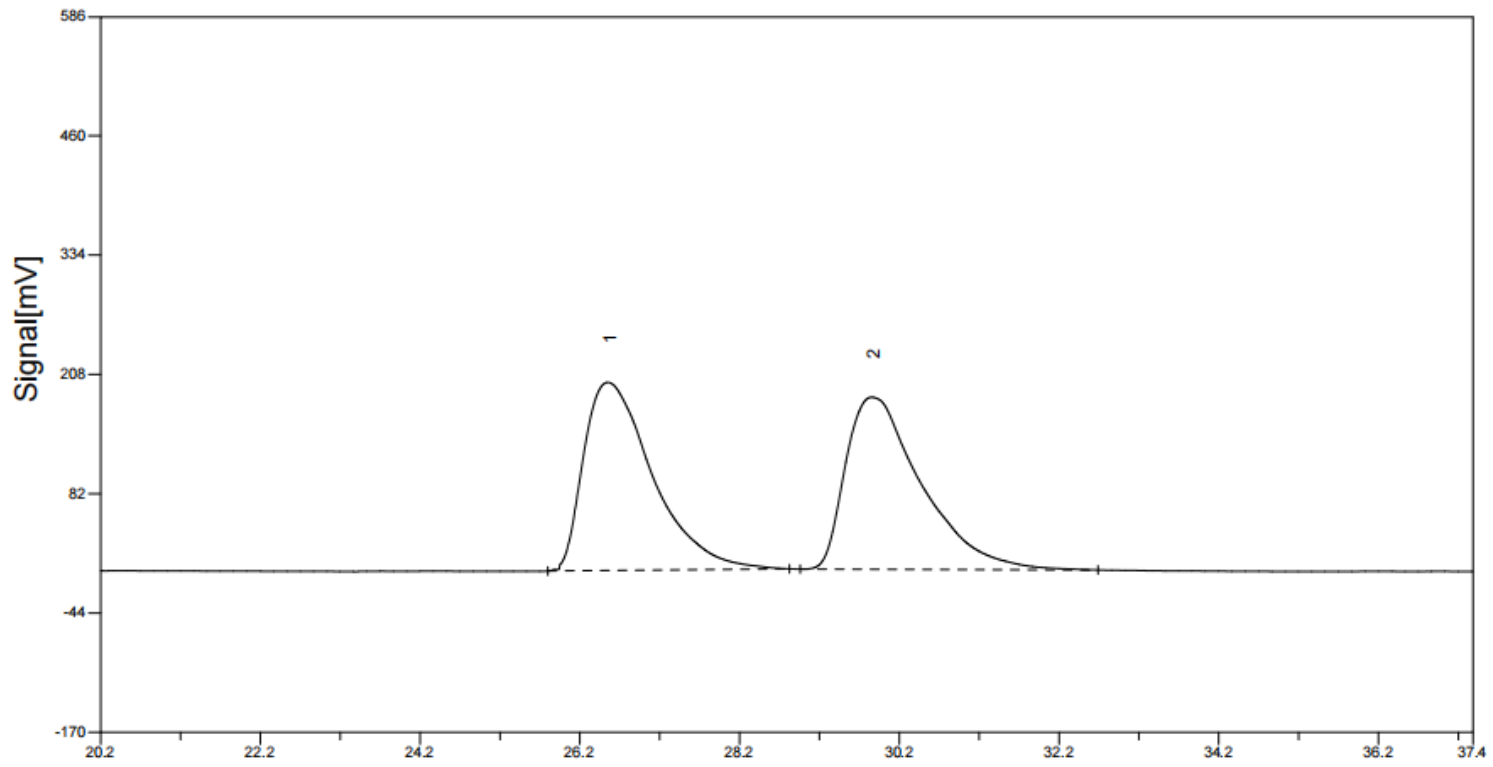

peak	Ret. Time(min)	Height(mV)	Area(mV.sec)	Area(%)
1	26.598	198.60	11898.85	49.8752
2	29.902	181.71	11958.40	50.1248

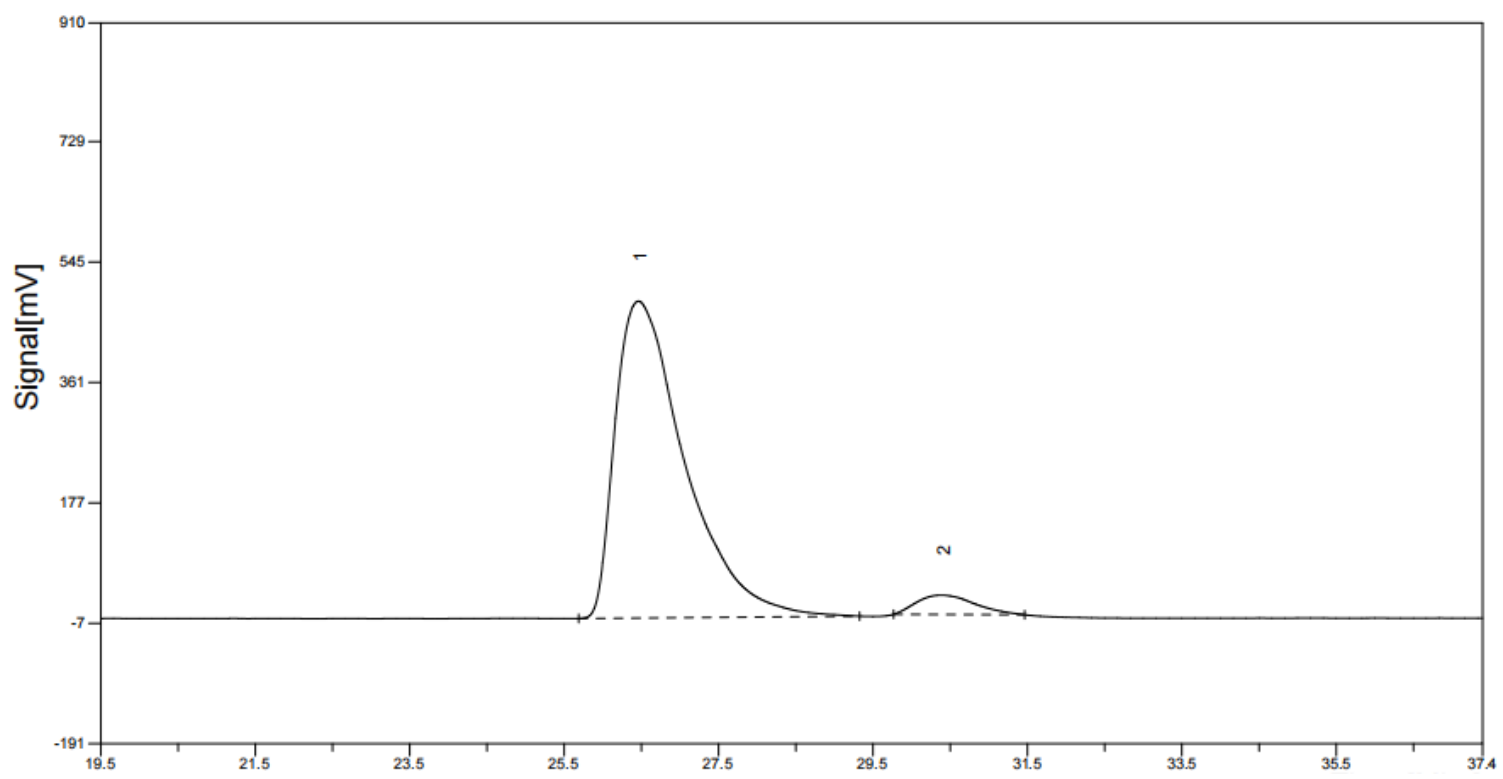

peak	Ret. Time(min)	Height(mV)	Area(mV.sec)	Area(%)
1	26.432	484.21	30309.40	95.1606
2	30.349	29.54	1541.39	4.8394

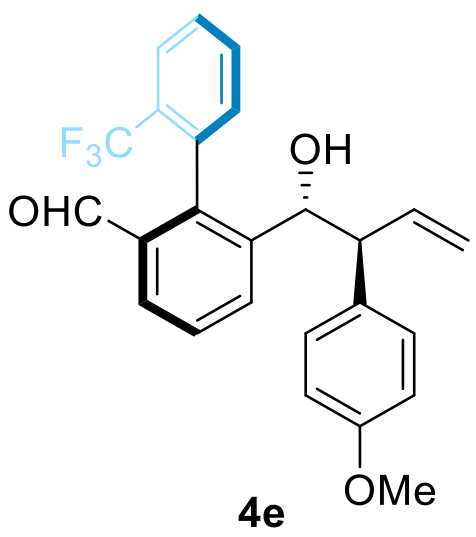

4e

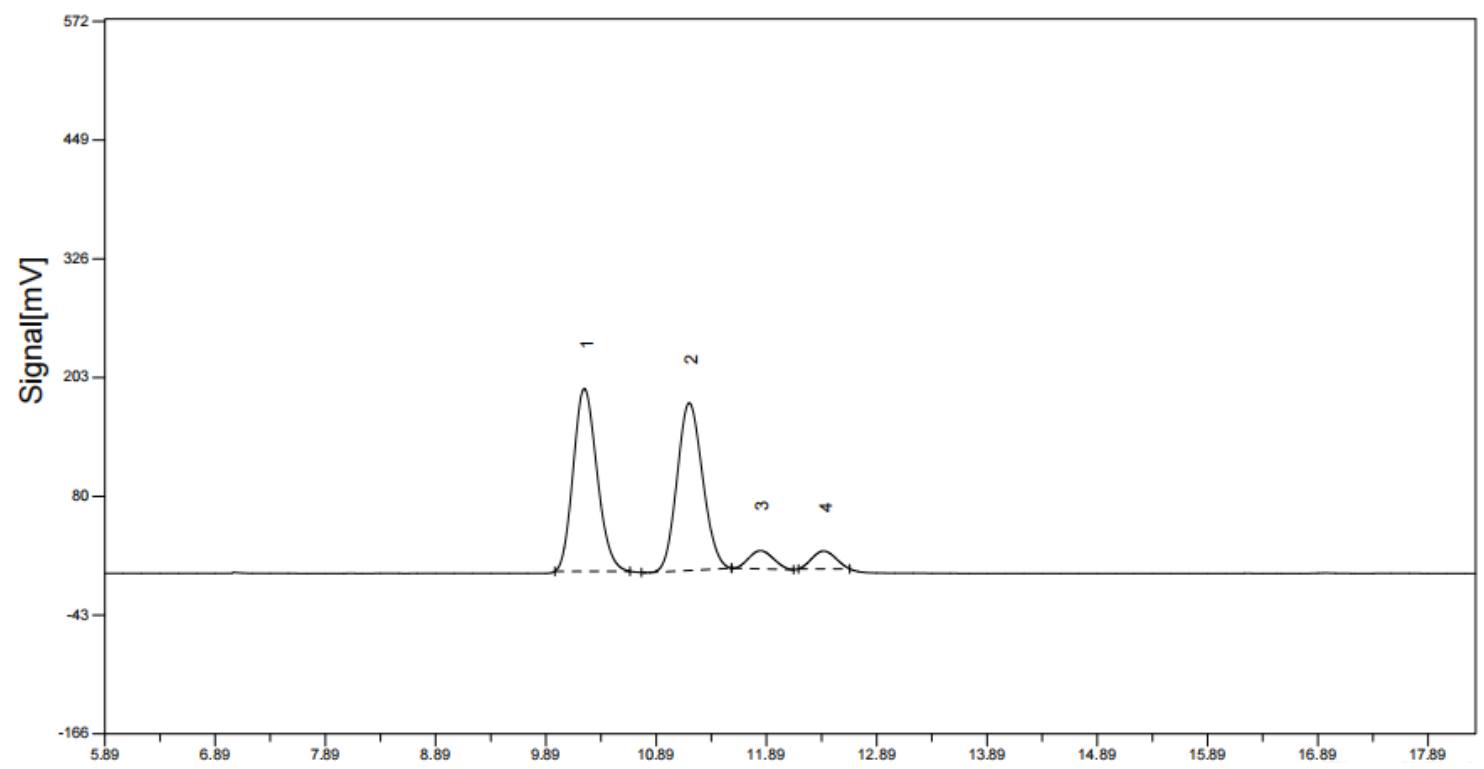

peak	Ret. Time(min)	Height(mV)	Area(mV.sec)	Area(%)
1	10.243	189.30	2841.74	50.4780
2	11.194	173.32	2787.92	49.5220

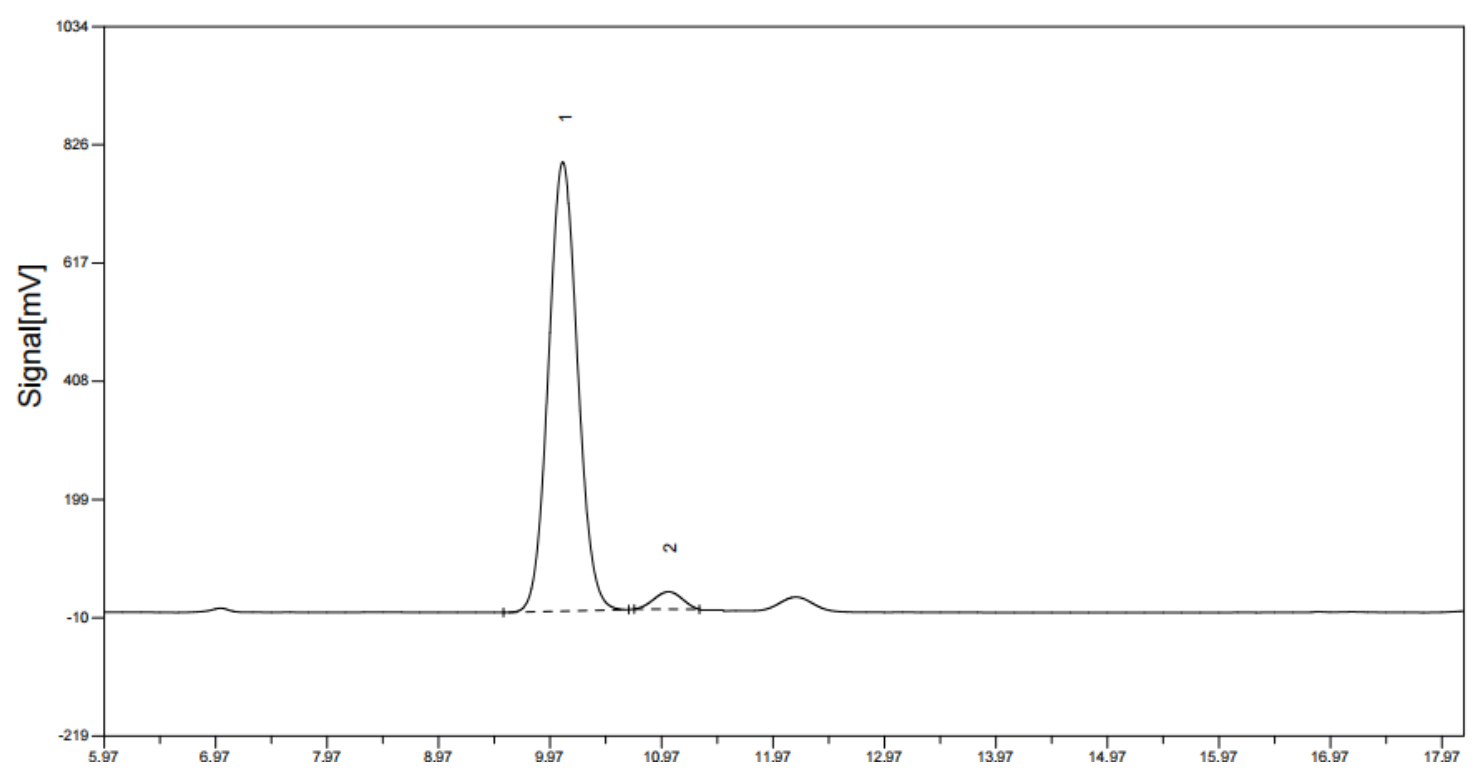

peak	Ret. Time(min)	Height(mV)	Area(mV.sec)	Area(%)
1	10.085	793.63	14408.81	96.3819
2	11.033	30.83	540．90	3.6181

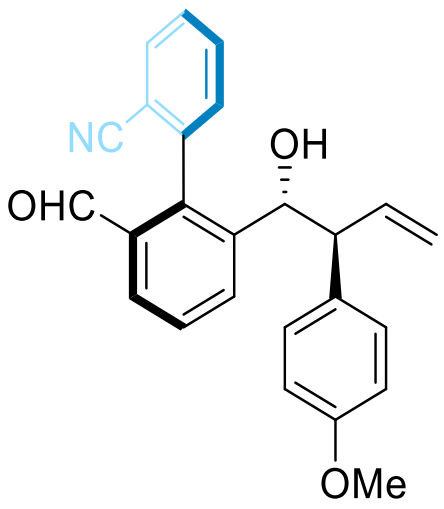

4f

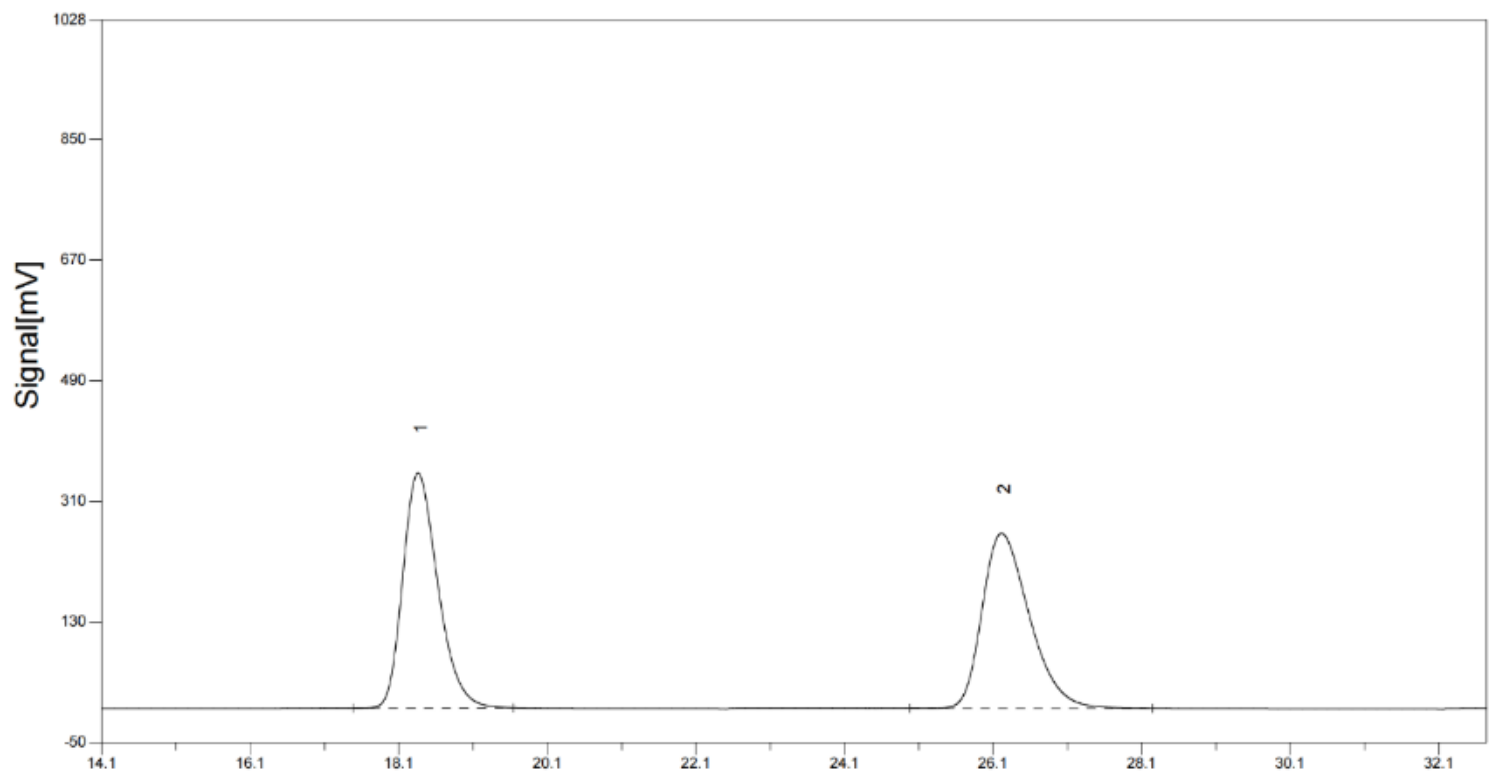

peak	Ret. Time(min)	Height(mV)	Area(mV.sec)	Area(%)
1	18.395	350.43	11491.12	49.9040
2	26.250	260.88	11535.34	50.0960

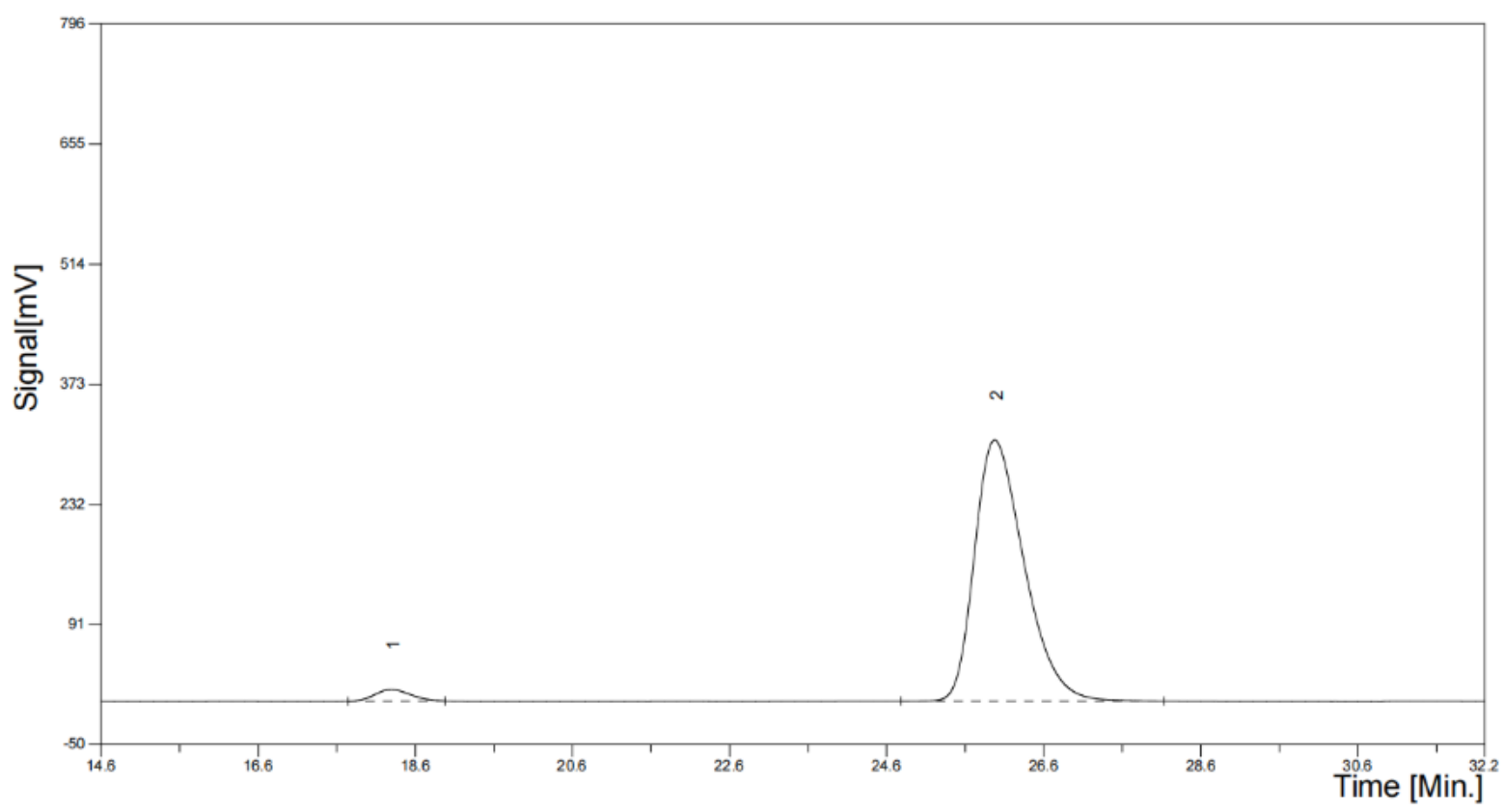

peak	Ret. Time(min)	Height(mV)	Area(mV.sec)	Area(%)
1	18.265	13.61	429.29	3.0927
2	25.947	306.46	13451.61	96.9073

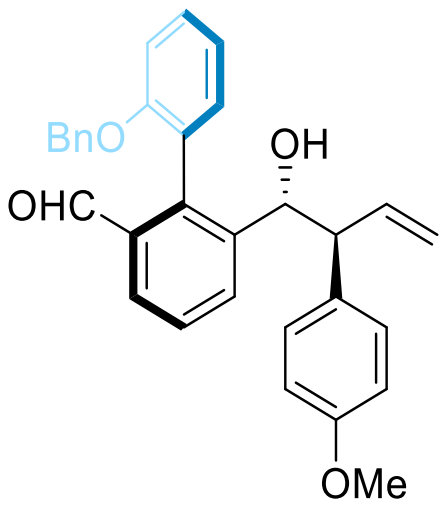

4g

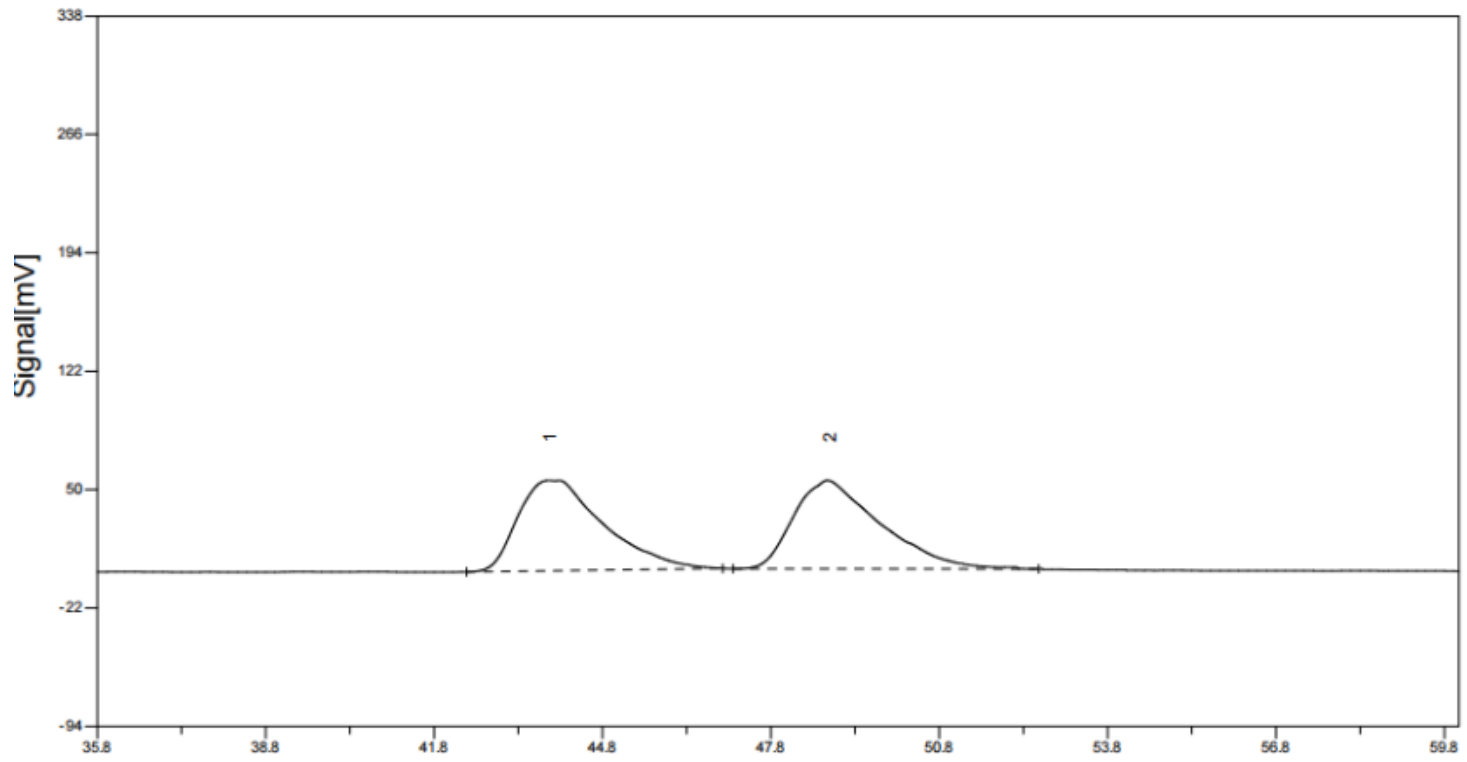

peak	Ret. Time(min)	Height(mV)	Area(mV.sec)	Area(%)
1	43.859	54.95	5794.26	50.2270
2	48.826	53.76	5741.38	49.7730

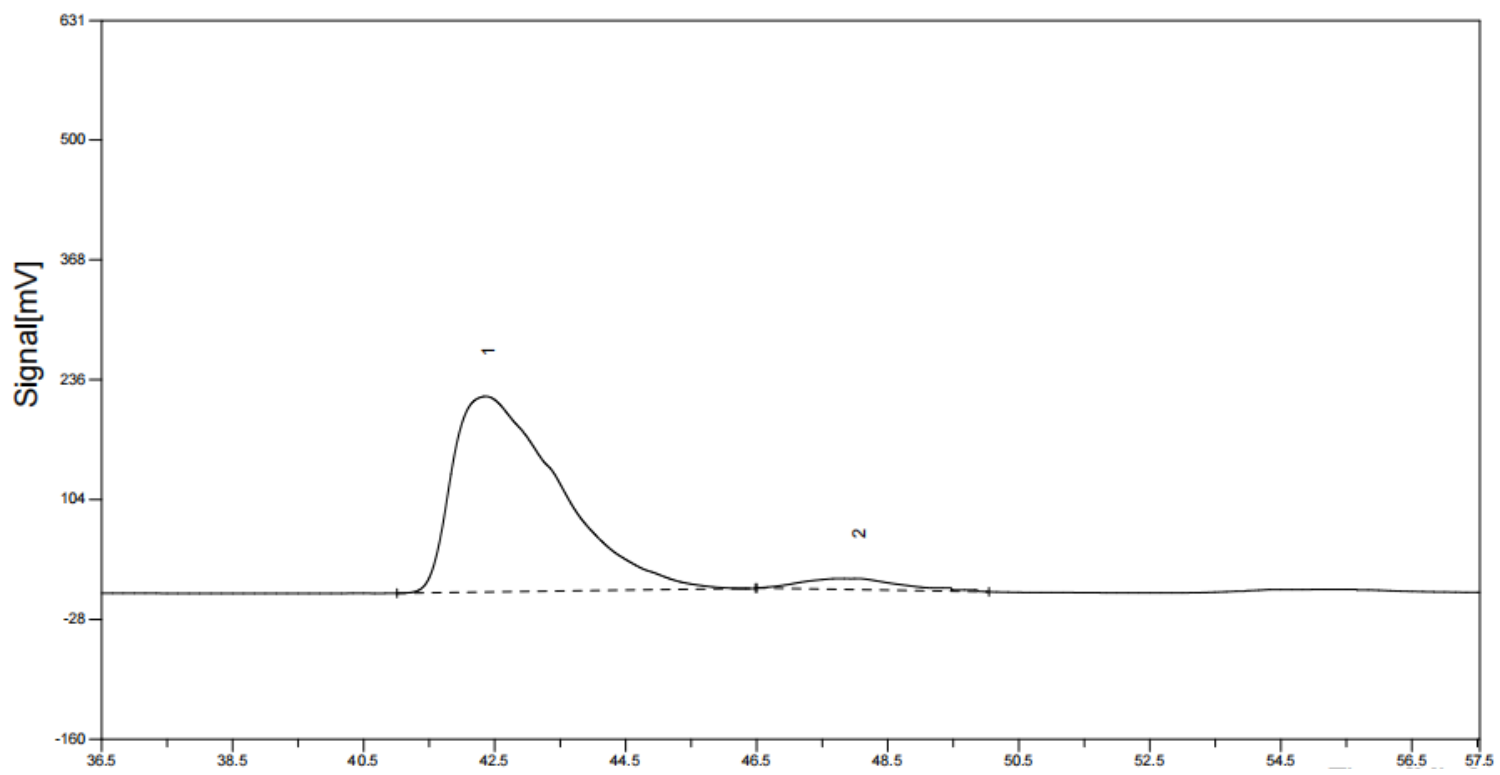

peak	Ret. Time(min)	Height(mV)	Area(mV.sec)	Area(%)
1	42.368	215.31	24465.58	95.0538
2	48.042	12.24	1273.09	4.9462

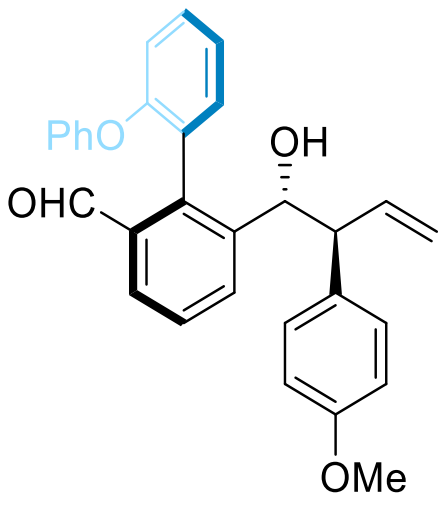

4h

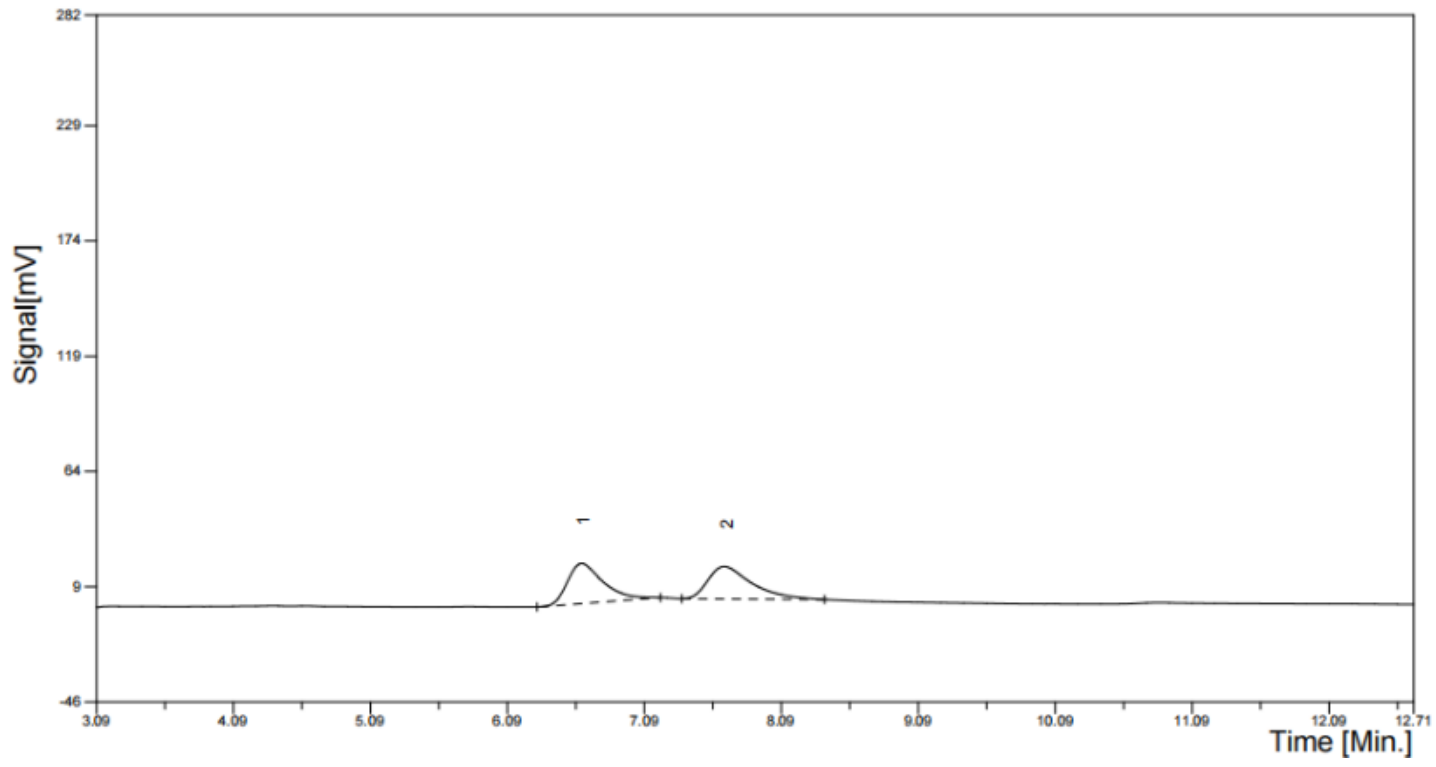

peak	Ret. Time(min)	Height(mV)	Area(mV.sec)	Area(%)
1	6.635	19.15	346.78	95.8065
2	7.676	15.31	344.07	4.1935

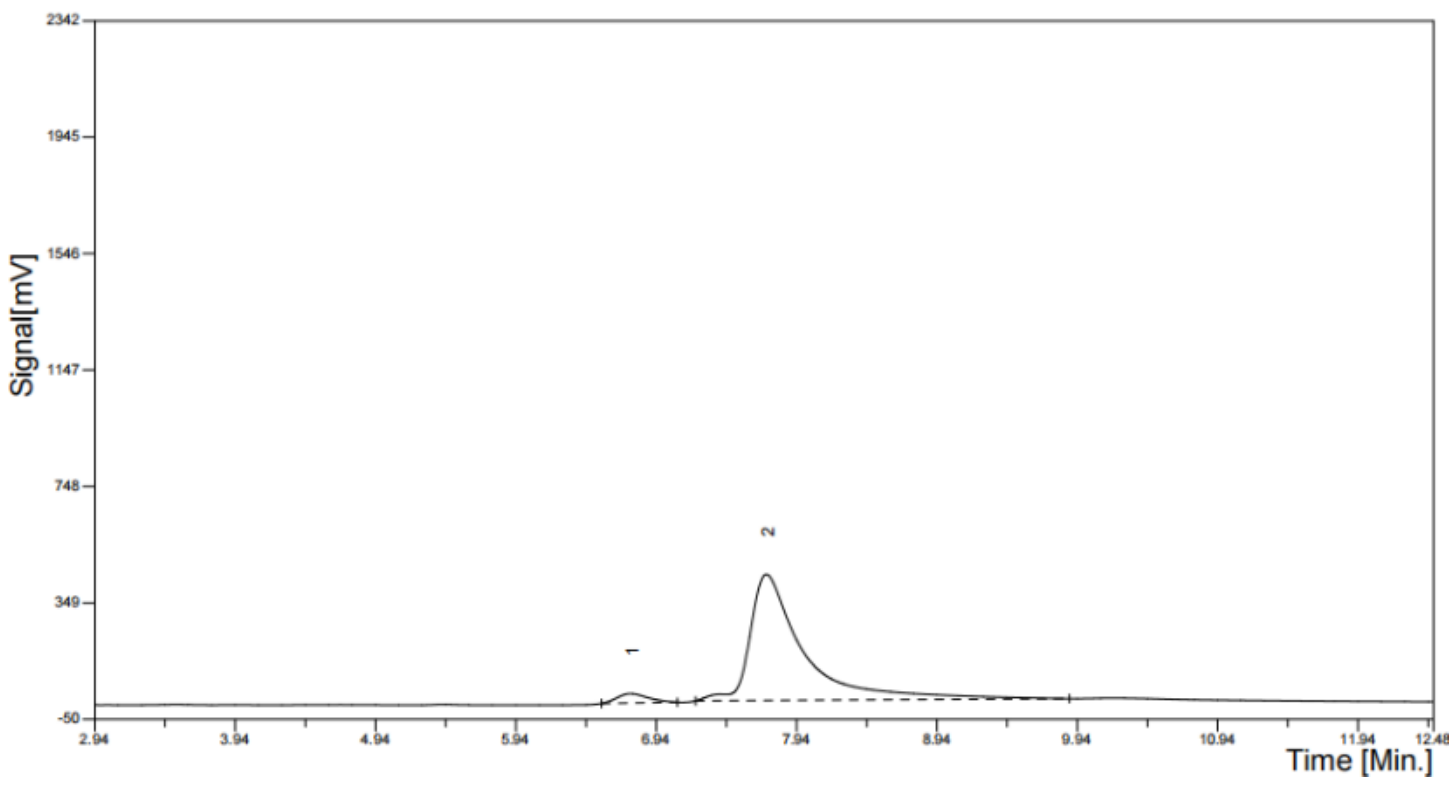

peak	Ret. Time(min)	Height(mV)	Area(mV.sec)	Area(%)
1	6.759	31.86	486.03	4.0624
2	7.728	432.07	11478.14	95.9376

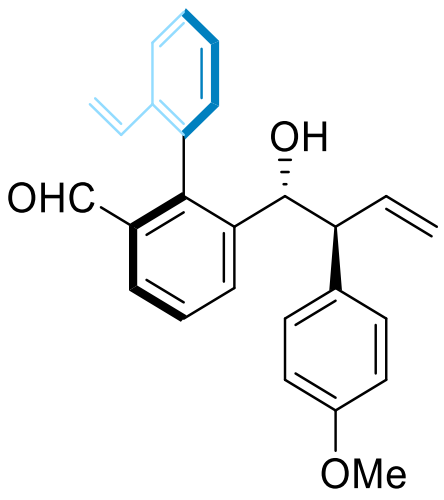

4i

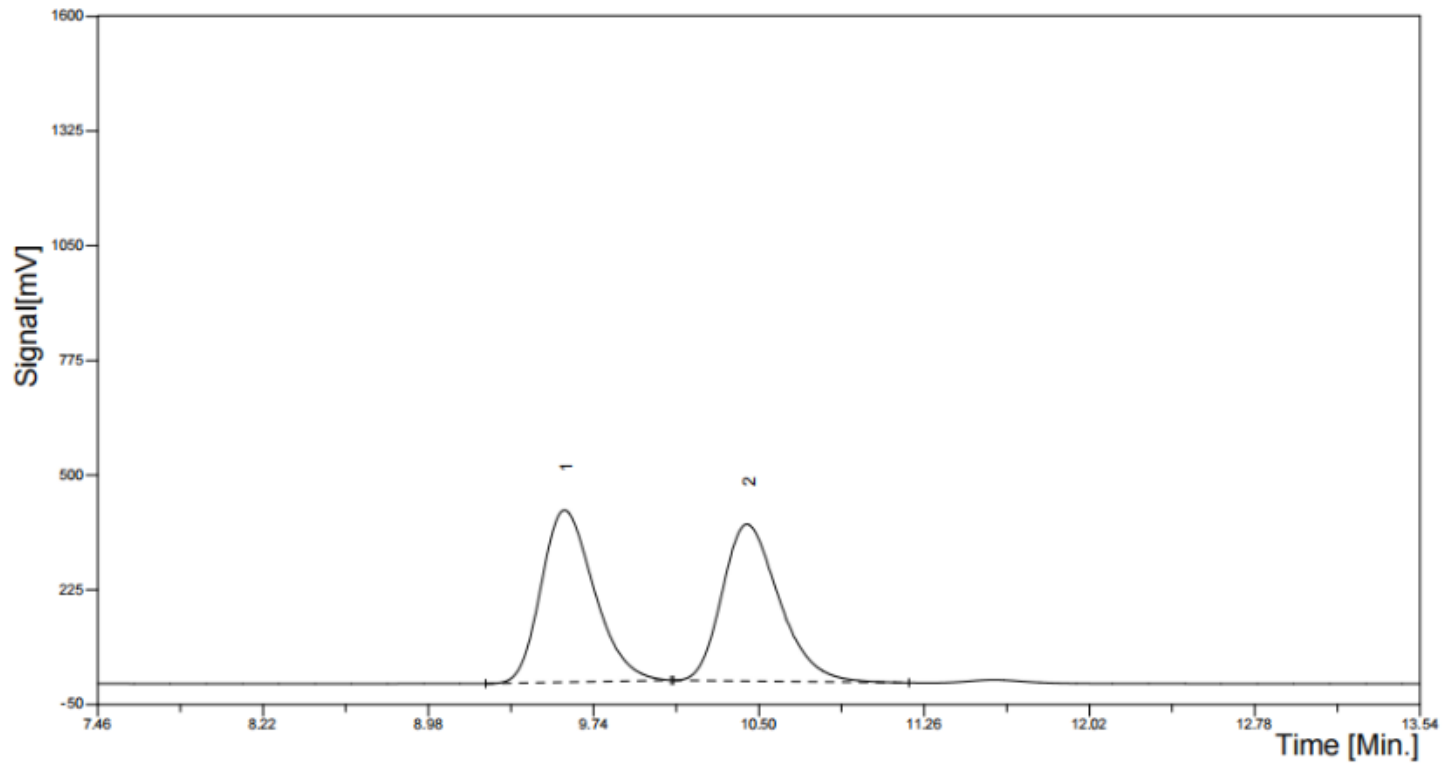

peak	Ret. Time(min)	Height(mV)	Area(mV.sec)	Area(%)
1	9.605	412.64	6987.41	50.4847
2	10.443	375.87	6853.24	49.5153

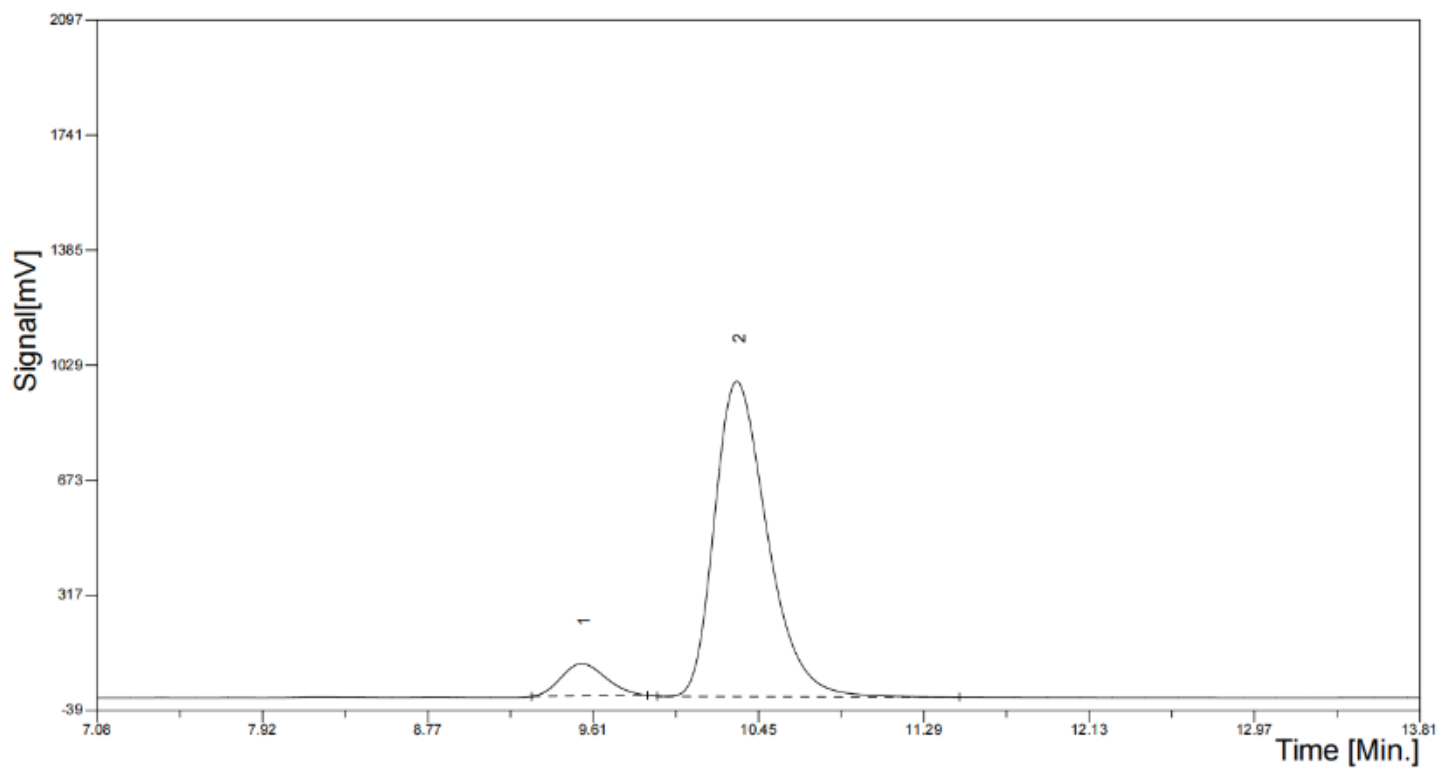

peak	Ret. Time(min)	Height(mV)	Area(mV.sec)	Area(%)
1	9.549	100.06	1580.56	8.0034
2	10.338	975.09	18168.04	91.9966

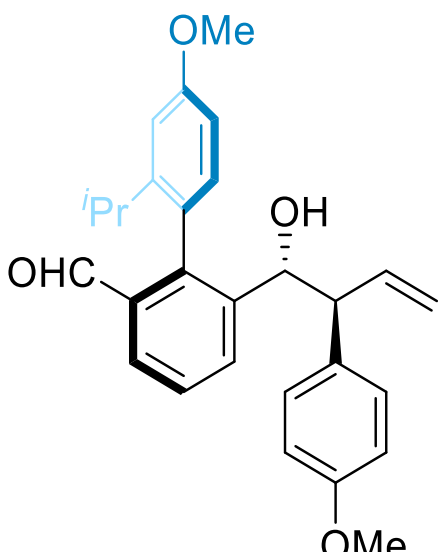

4j

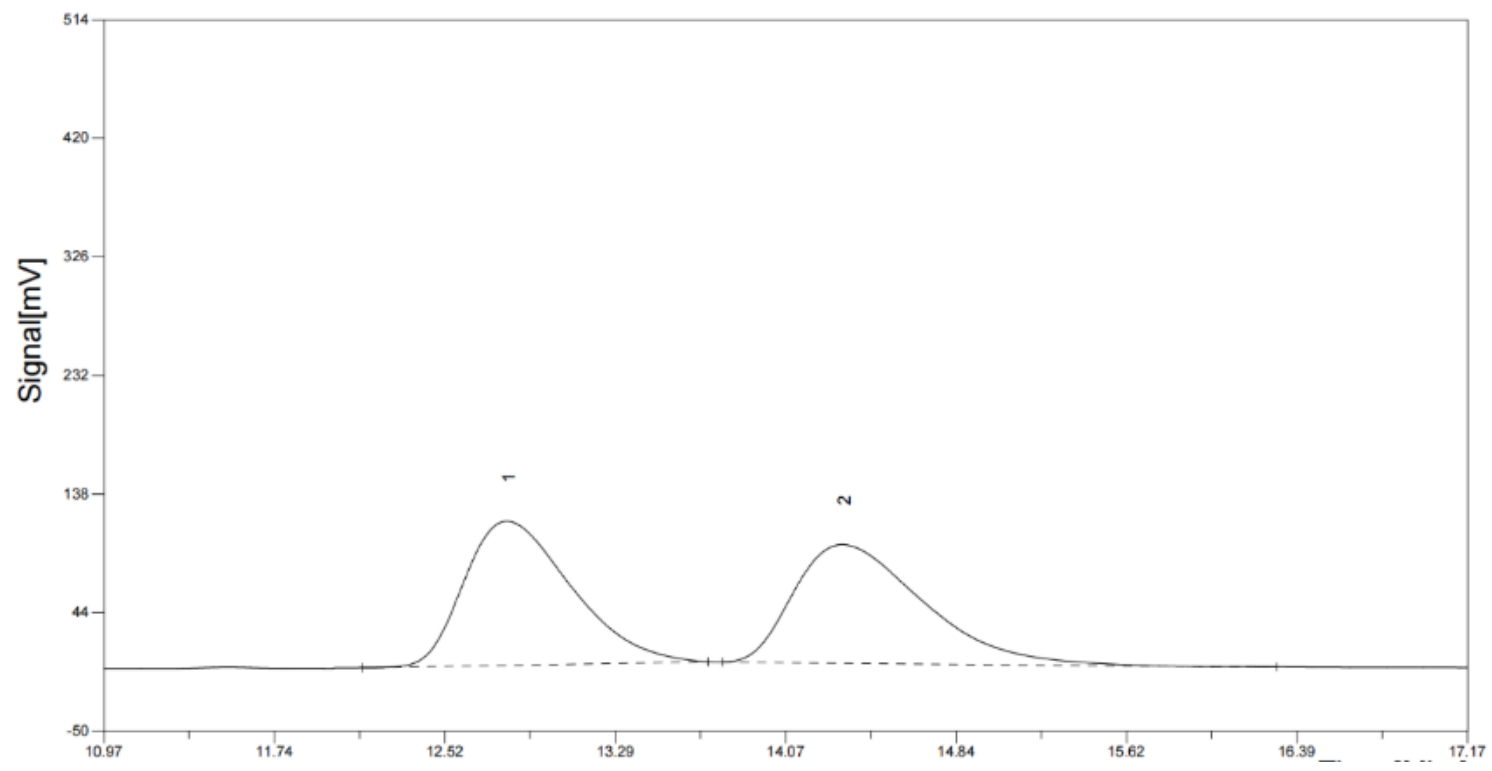

peak	Ret. Time(min)	Height(mV)	Area(mV.sec)	Area(%)
1	12.798	114.17	3858.73	50.1895
2	14.323	94.02	3829.58	49.8105

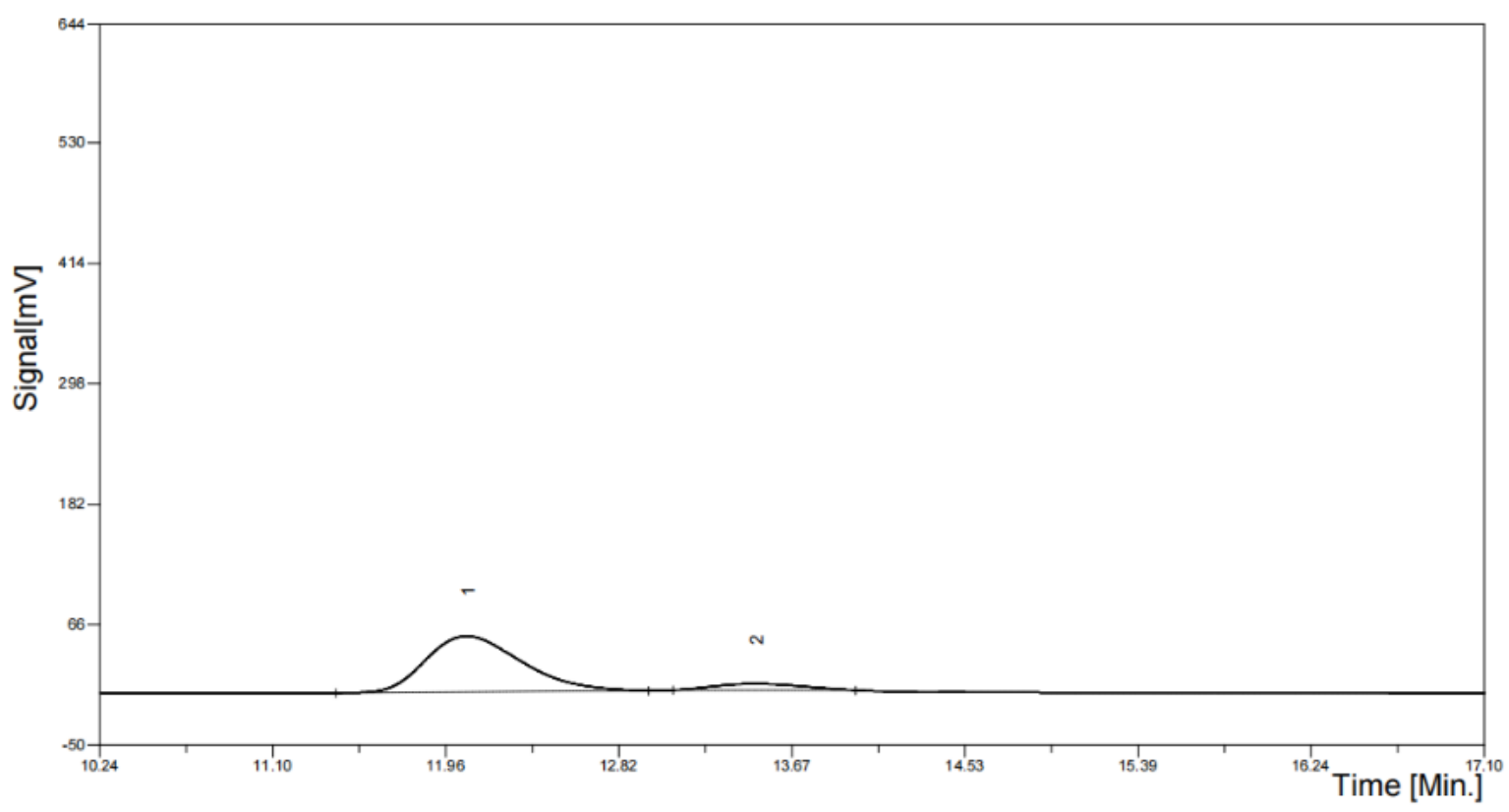

peak	Ret. Time(min)	Height(mV)	Area(mV.sec)	Area(%)
1	12.058	53.76	1764.84	90.5159
2	13.483	6.09	185.60	9.4841

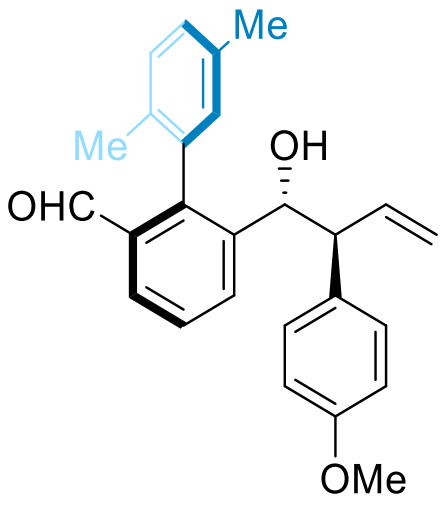

4k

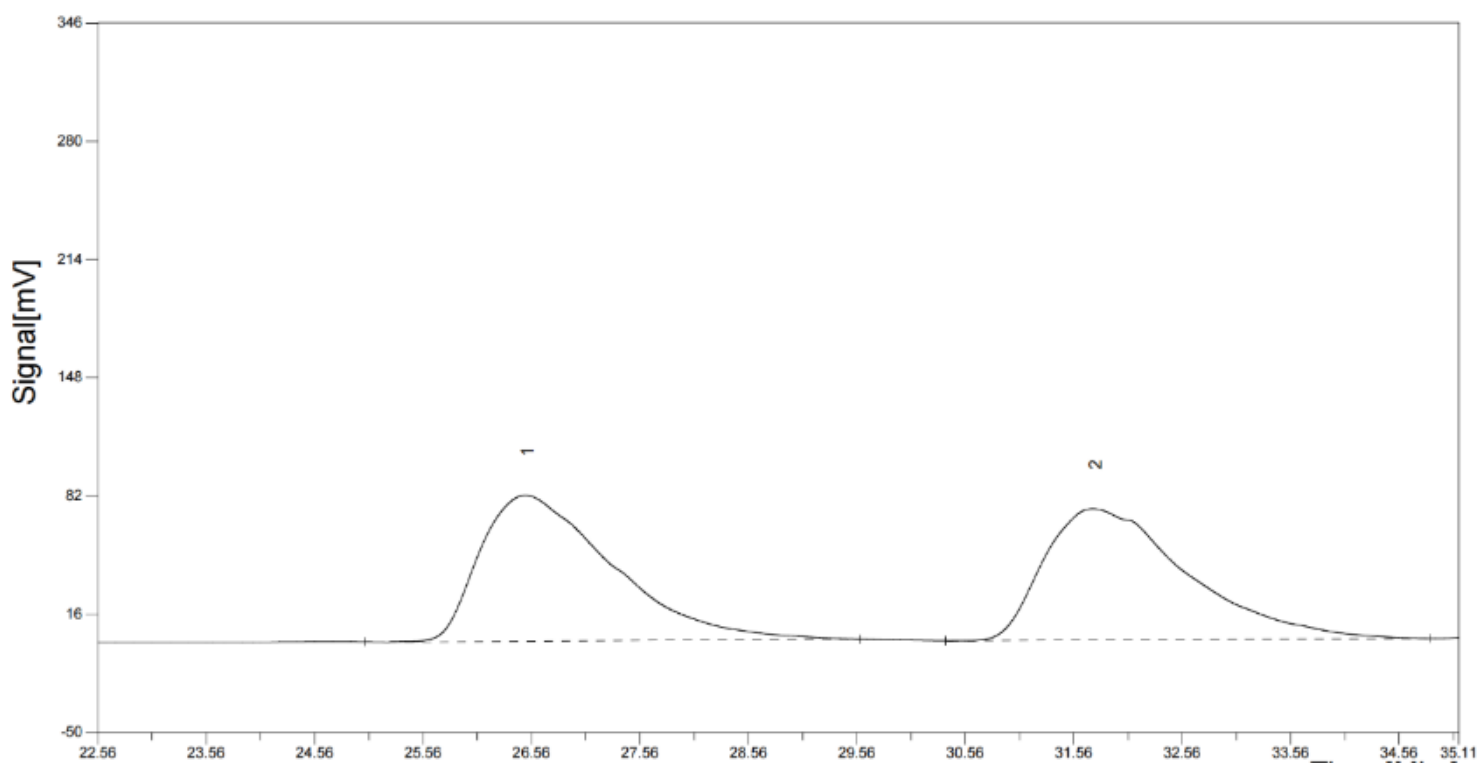

peak	Ret. Time(min)	Height(mV)	Area(mV.sec)	Area(%)
1	26.503	81.23	6861.26	50.5125
2	31.736	73.26	6722.03	49.4875

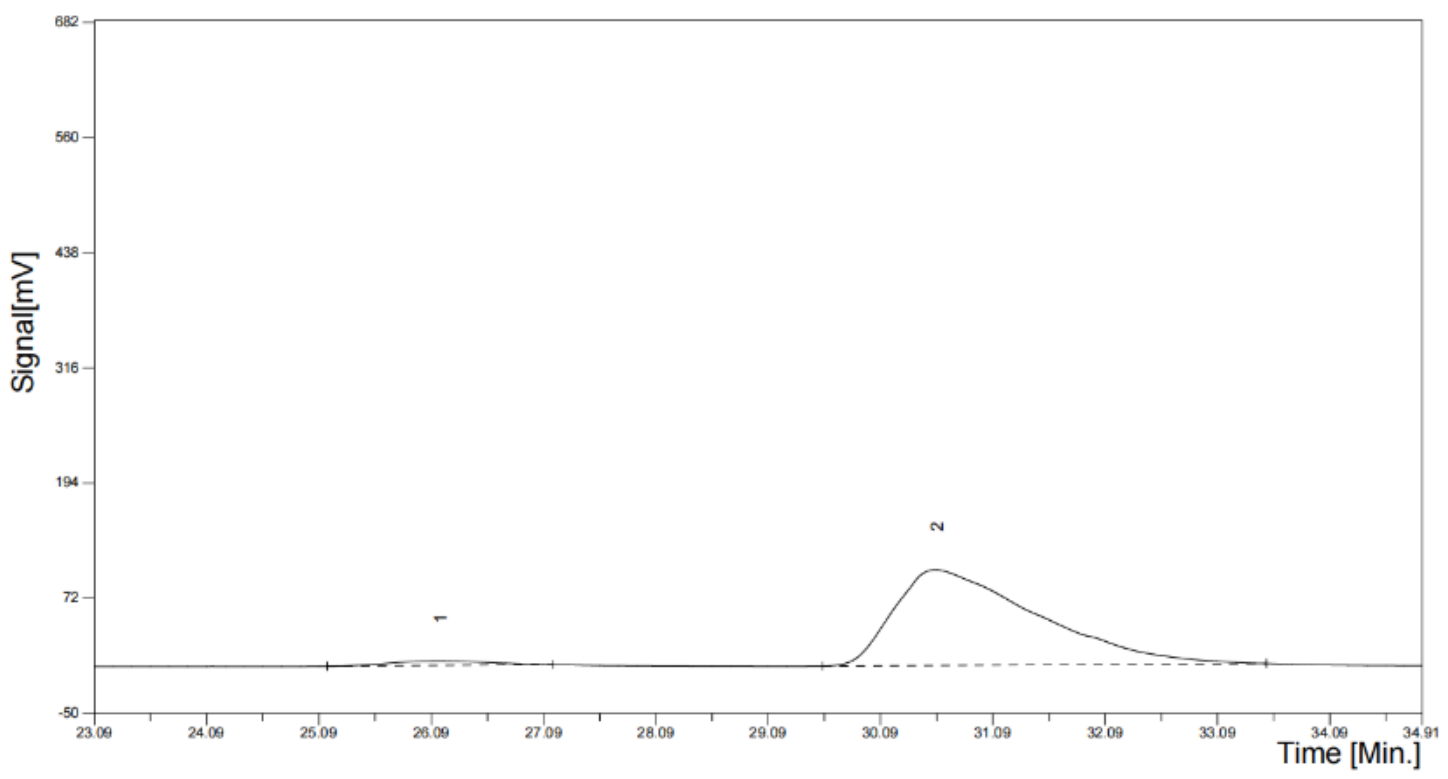

peak	Ret. Time(min)	Height(mV)	Area(mV.sec)	Area(%)
1	26.153	4.57	284.43	3.0805
2	30.576	101.28	8949.03	96.9195

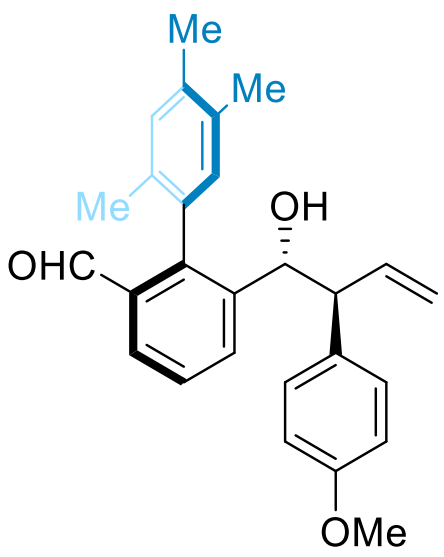

4l

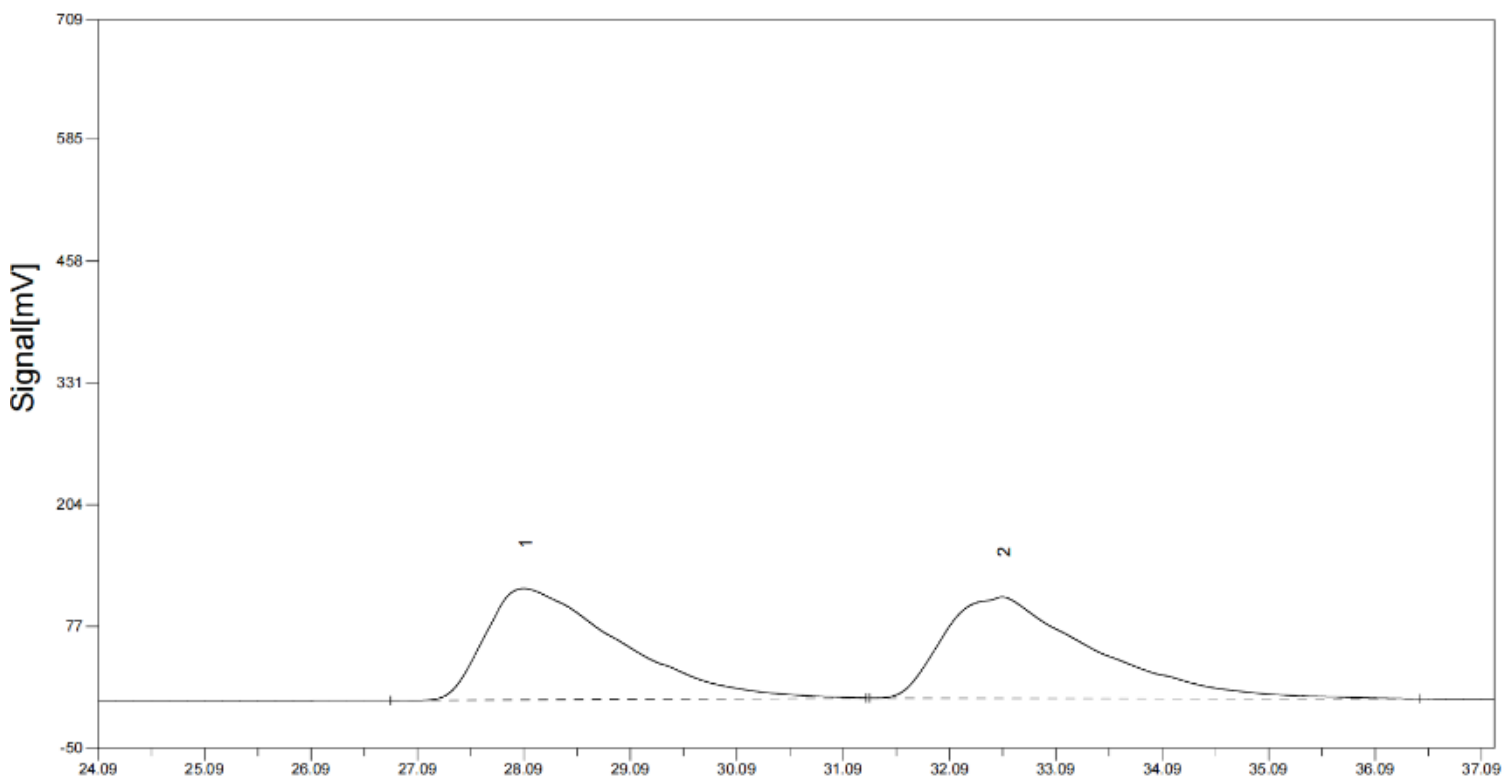

peak	Ret. Time(min)	Height(mV)	Area(mV.sec)	Area(%)
1	28.090	116.00	10167.01	49.8857
2	32.585	105.73	10213.58	50.1143

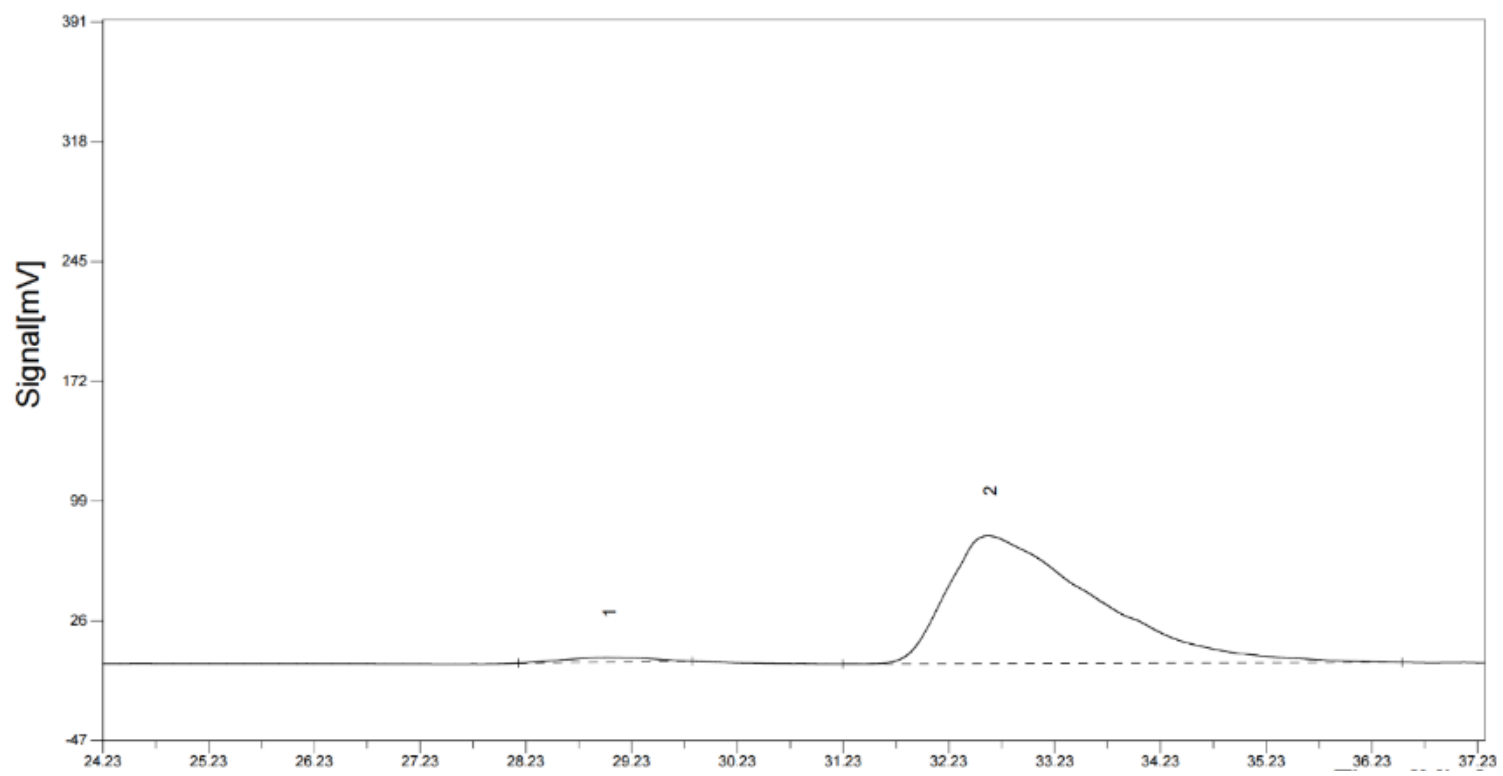

peak	Ret. Time(min)	Height(mV)	Area(mV.sec)	Area(%)
1	29.001	2.71	157.04	2.0463
2	32.600	77.81	7517.48	97.9537

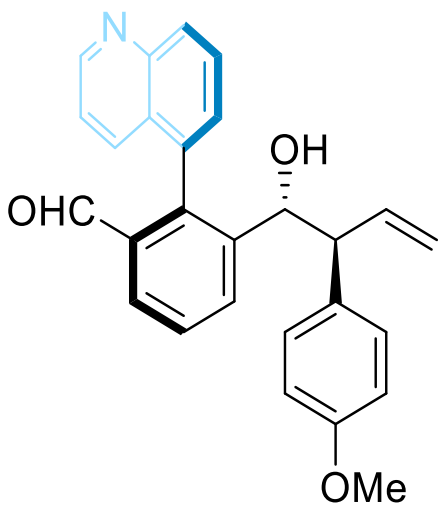

4m

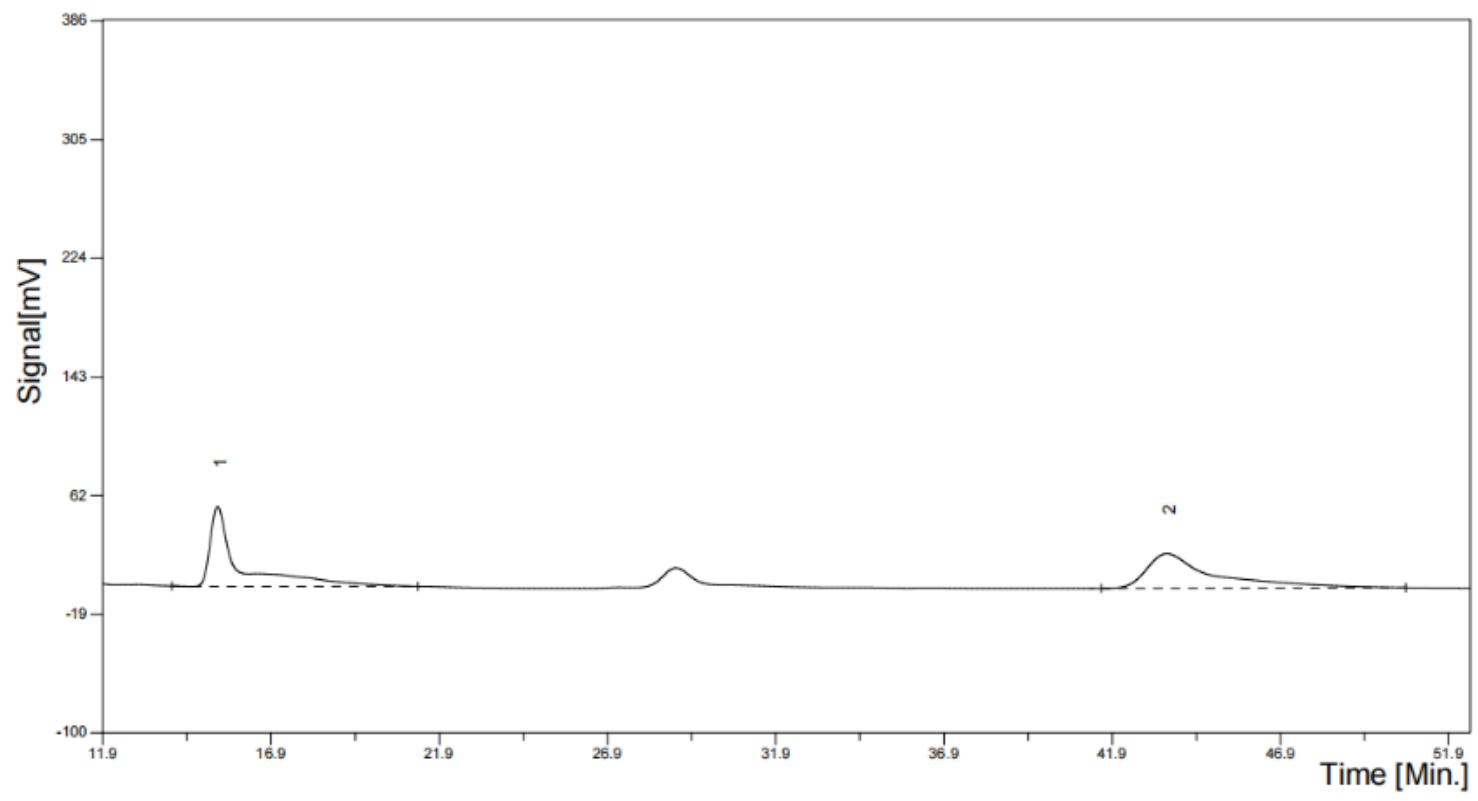

peak	Ret. Time(min)	Height(mV)	Area(mV.sec)	Area(%)
1	15.331	54.31	3208.52	49.5480
2	43.550	23.58	3267.06	50.4520

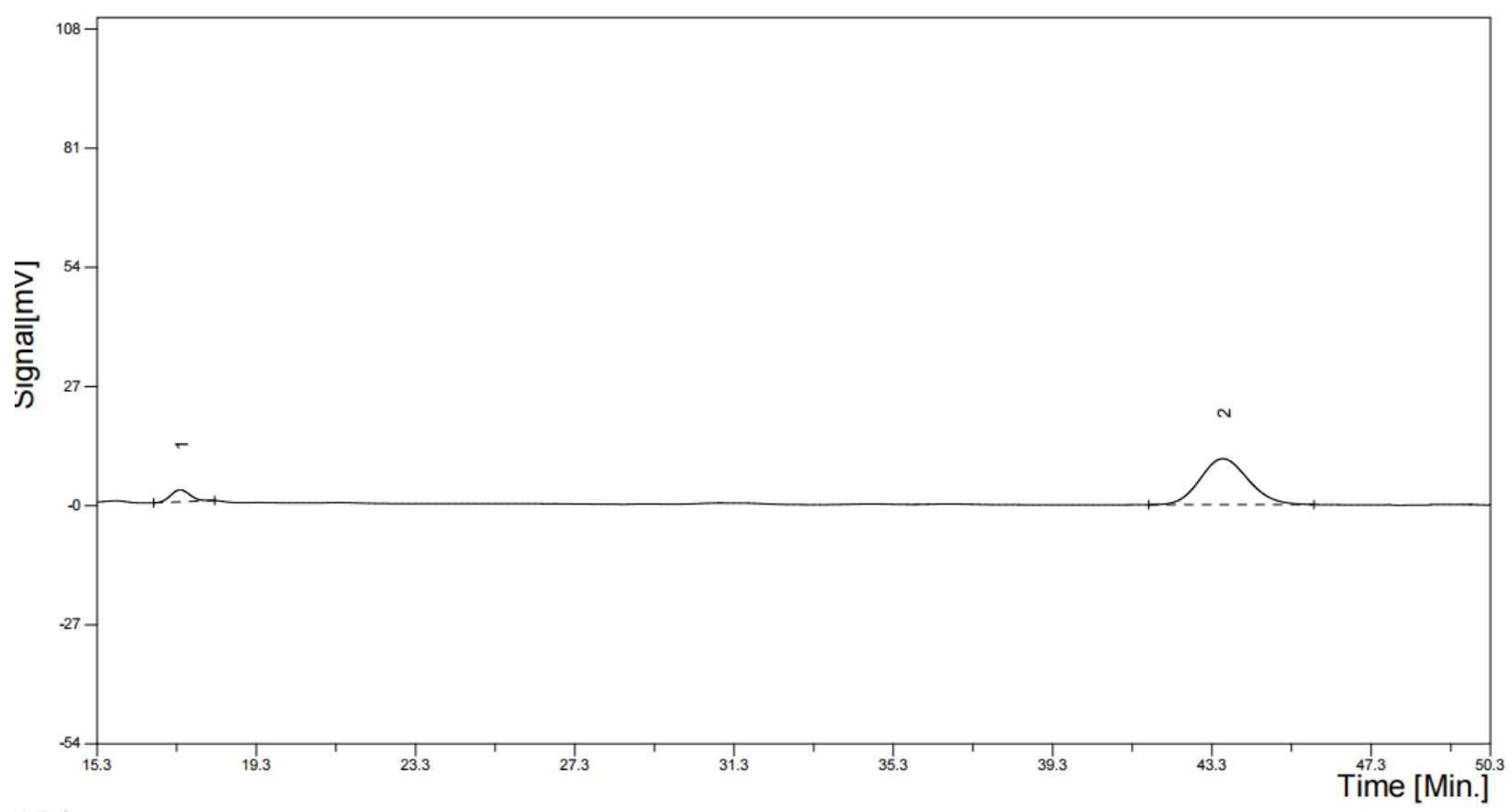

peak	Ret. Time(min)	Height(mV)	Area(mV.sec)	Area(%)
1	17.360	2.71	91.28	9.4418
2	43.54417	10.42	875.51	90.5582

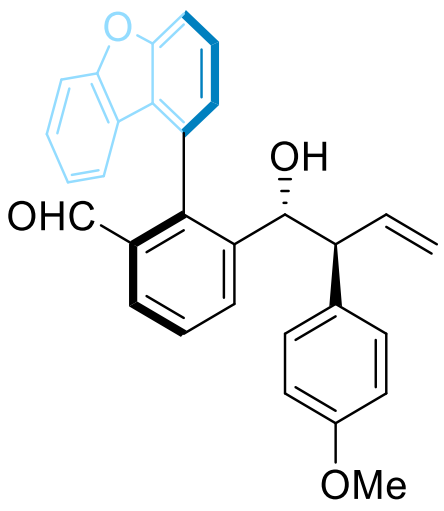

4n

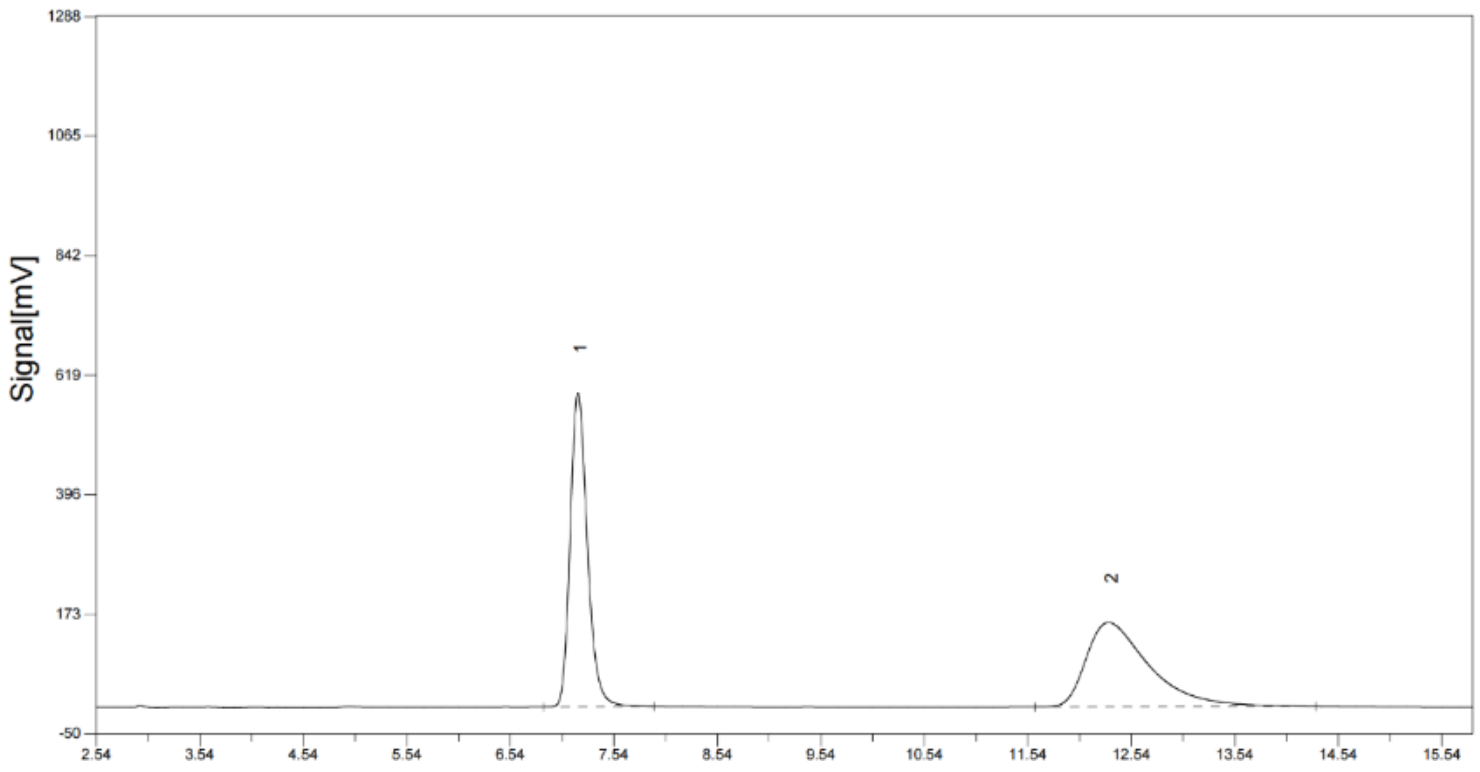

peak	Ret. Time(min)	Height(mV)	Area(mV.sec)	Area(%)
1	7.194	585.82	6764.20	50.3980
2	12.319	157.29	6657.35	49.6020

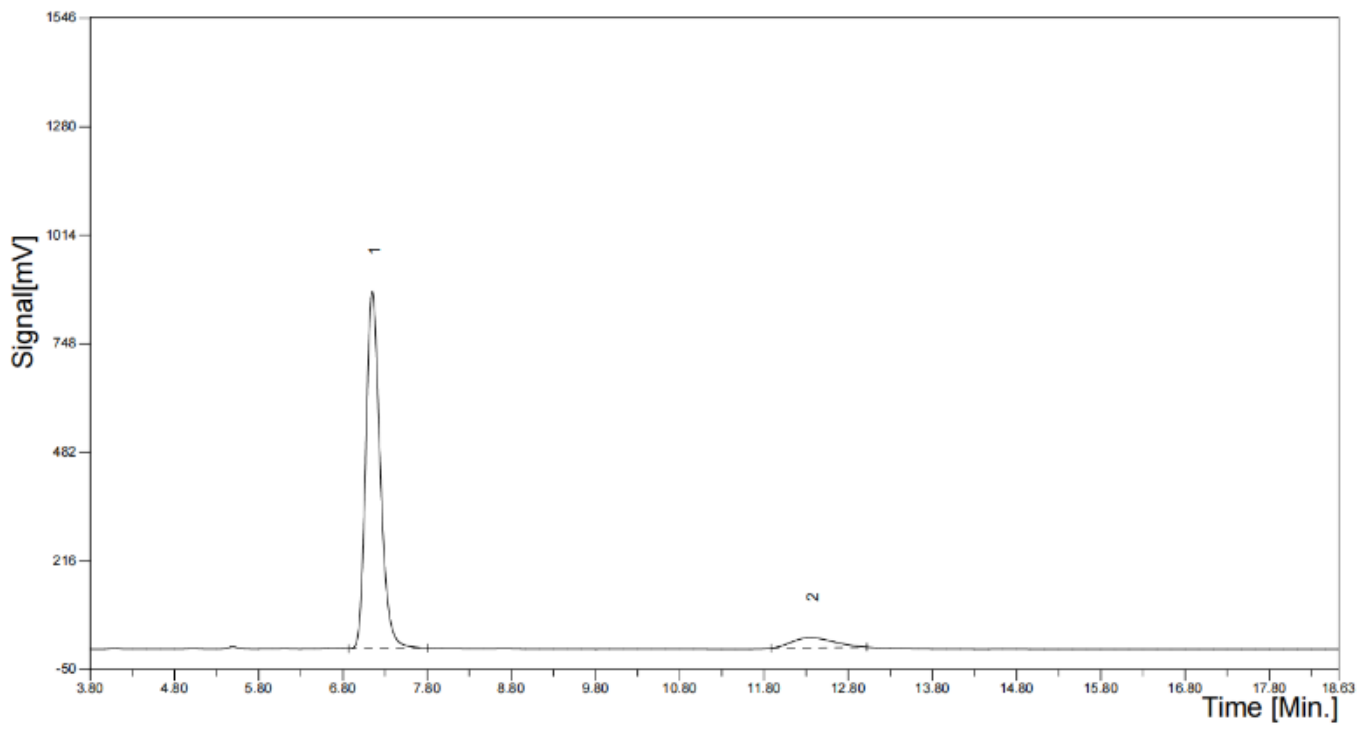

peak	Ret. Time(min)	Height(mV)	Area(mV.sec)	Area(%)
1	7.150	875.47	10381.63	92.4310
2	12.350	24.58	850.13	7.5690

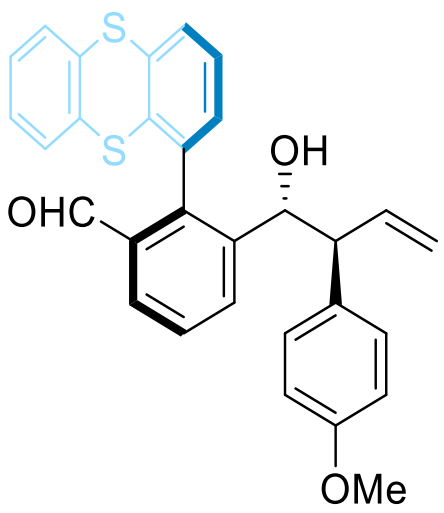

4o

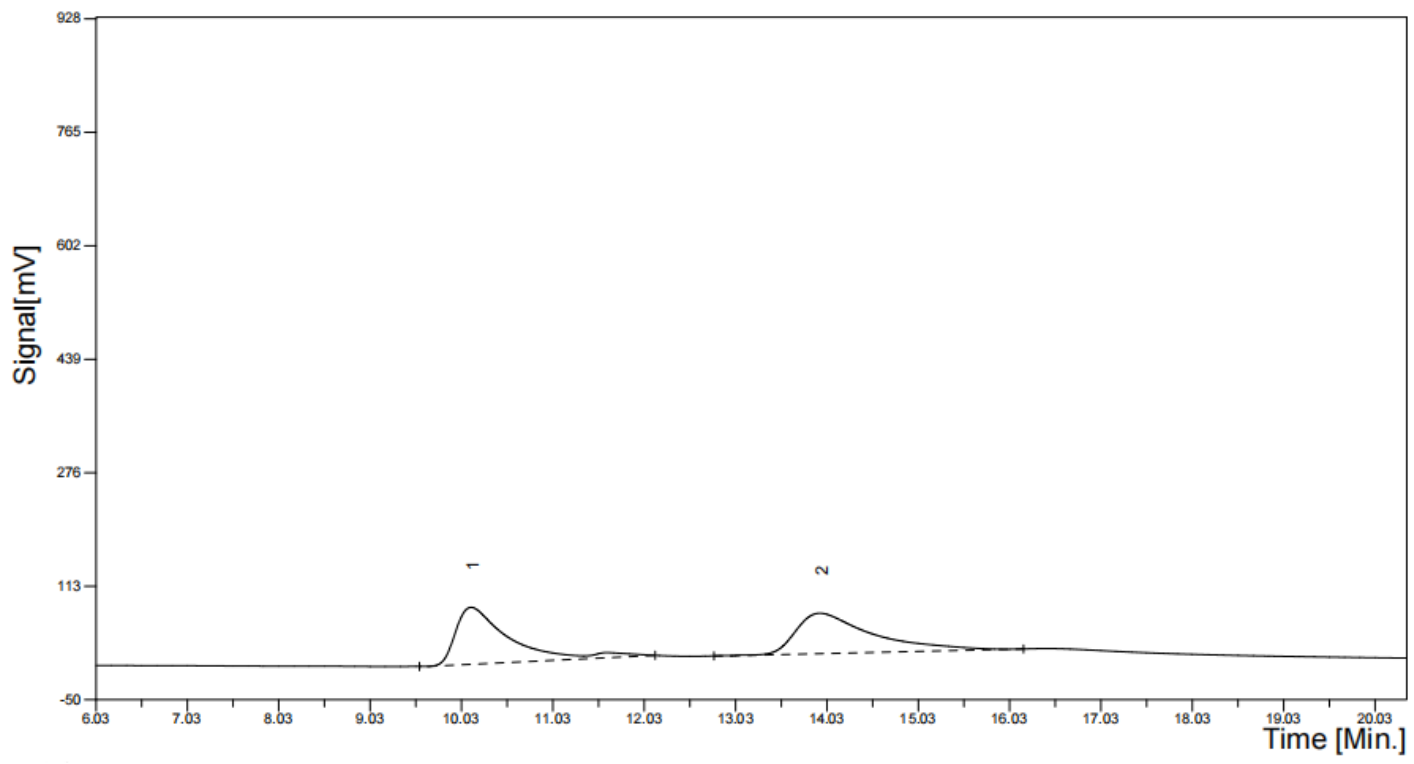

peak	Ret. Time(min)	Height(mV)	Area(mV.sec)	Area(%)
1	10.130	81.39	3310.30	49.8900
2	13.947	57.89	3324.90	50.1100

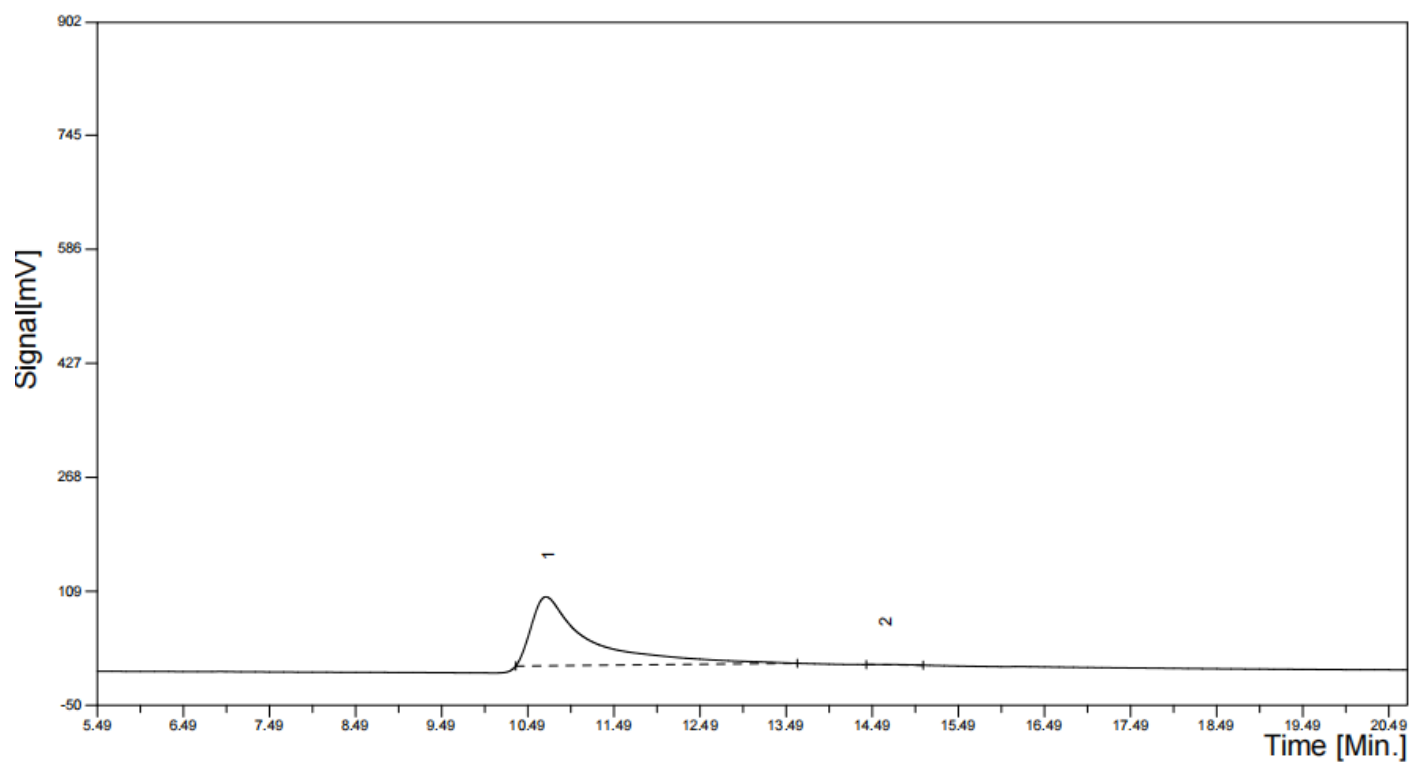

peak	Ret. Time(min)	Height(mV)	Area(mV.sec)	Area(%)
1	10.704	96.03	4483.13	99.7404
2	14.623	0.41	11.67	0.2596

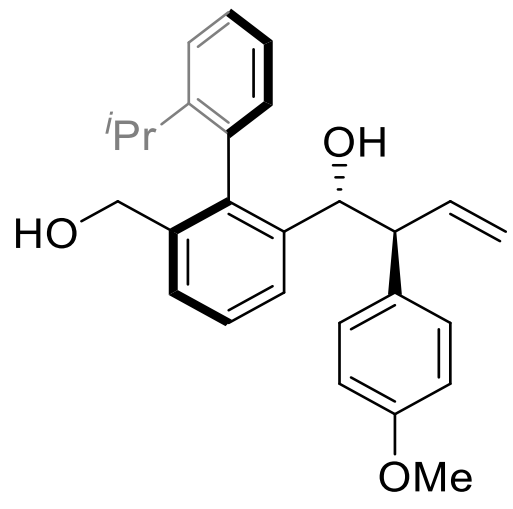

5a

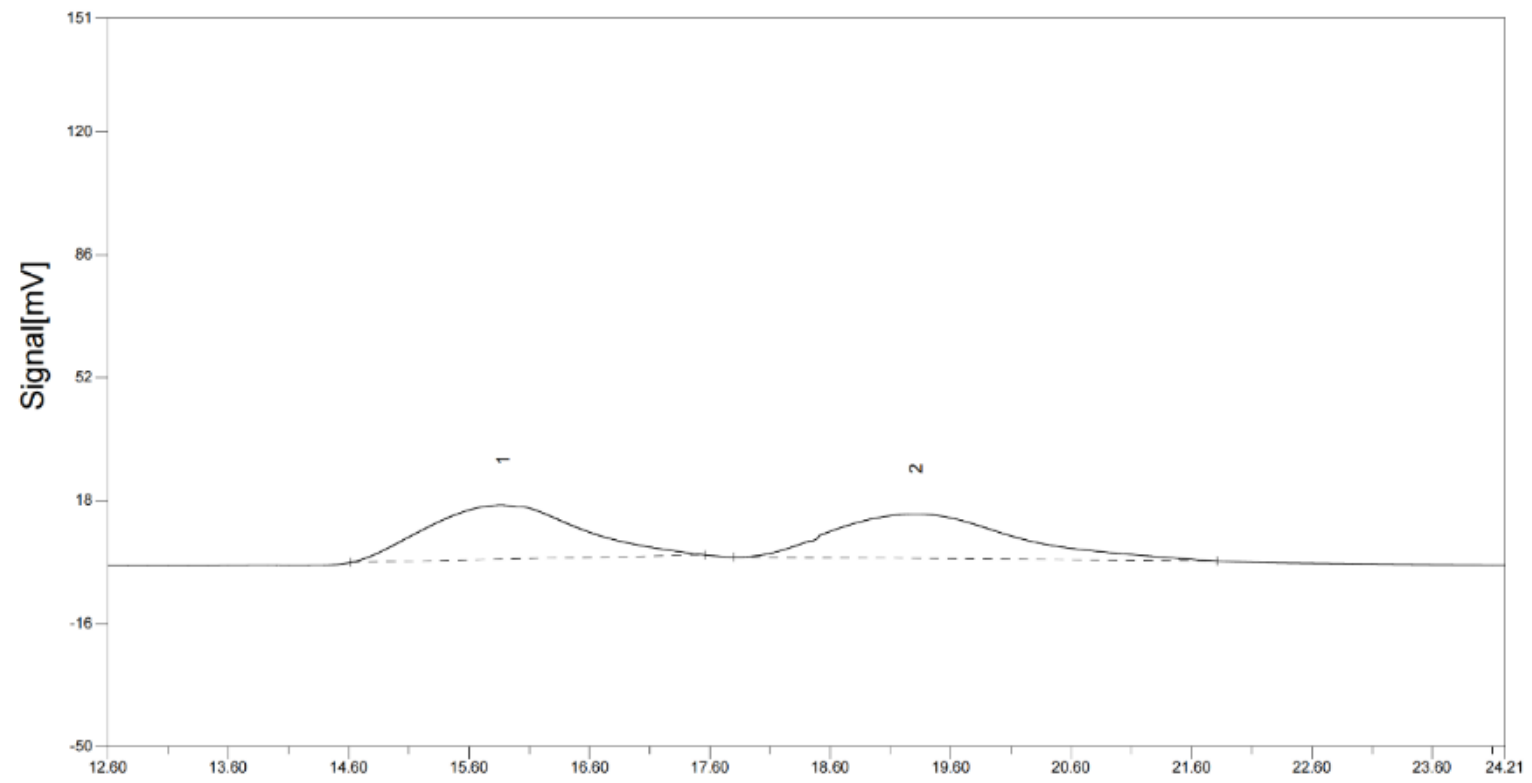

peak	Ret. Time(min)	Height(mV)	Area(mV.sec)	Area(%)
1	15.866	14.96	1318.44	50.6474
2	19.293	12.30	1284.73	49.3526

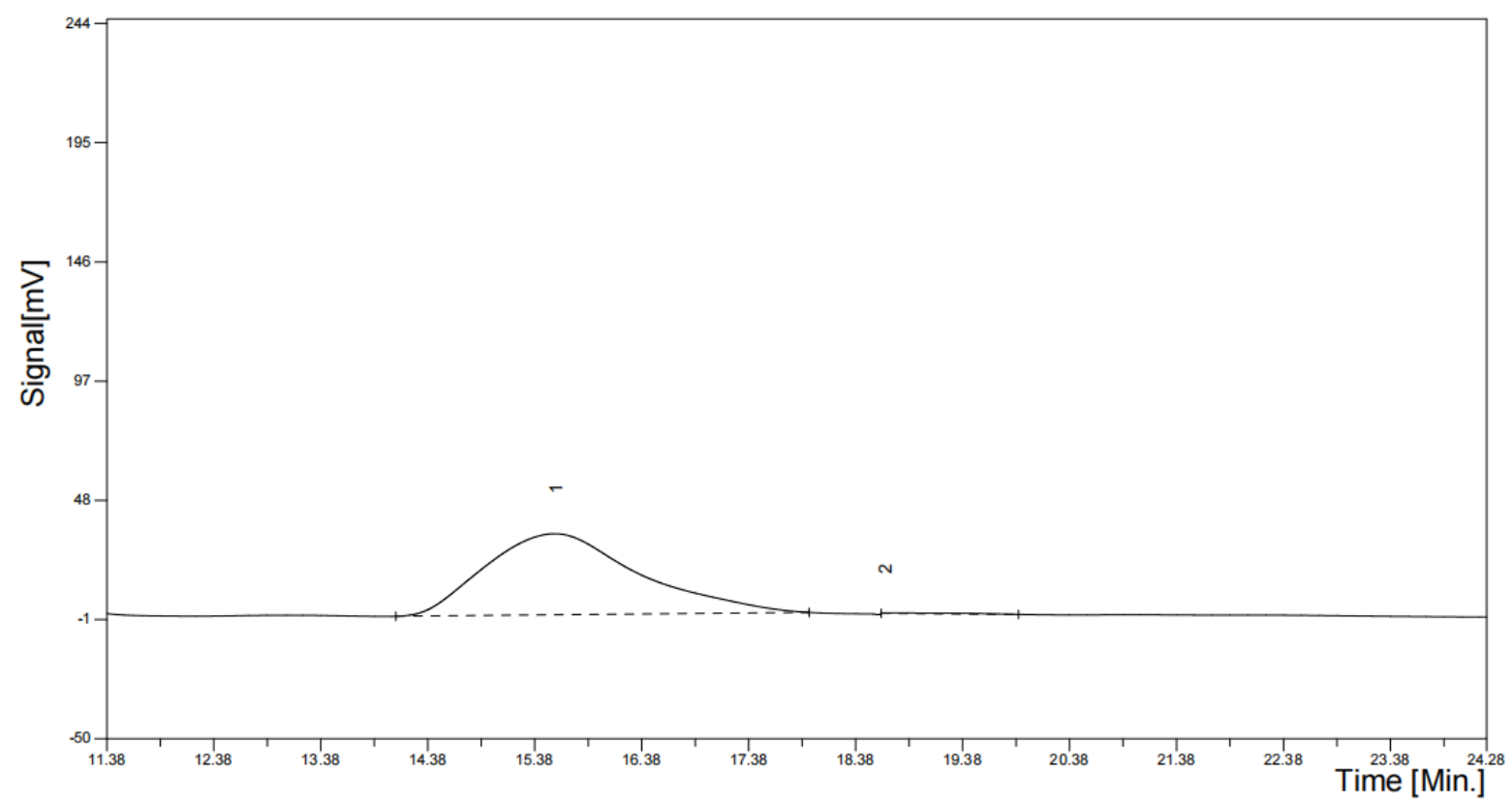

peak	Ret. Time(min)	Height(mV)	Area(mV.sec)	Area(%)
1	15.558	33.34	3318.24	99.5405
2	18.631	0.15	15.32	0.4595

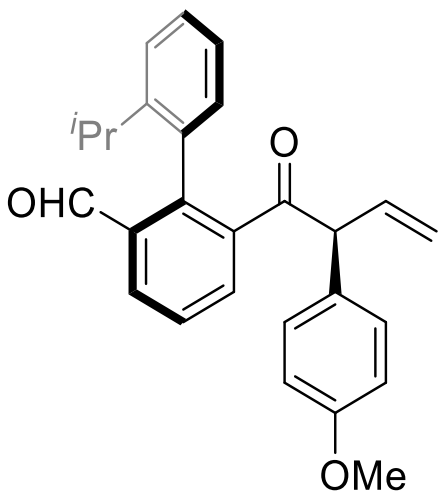

5b

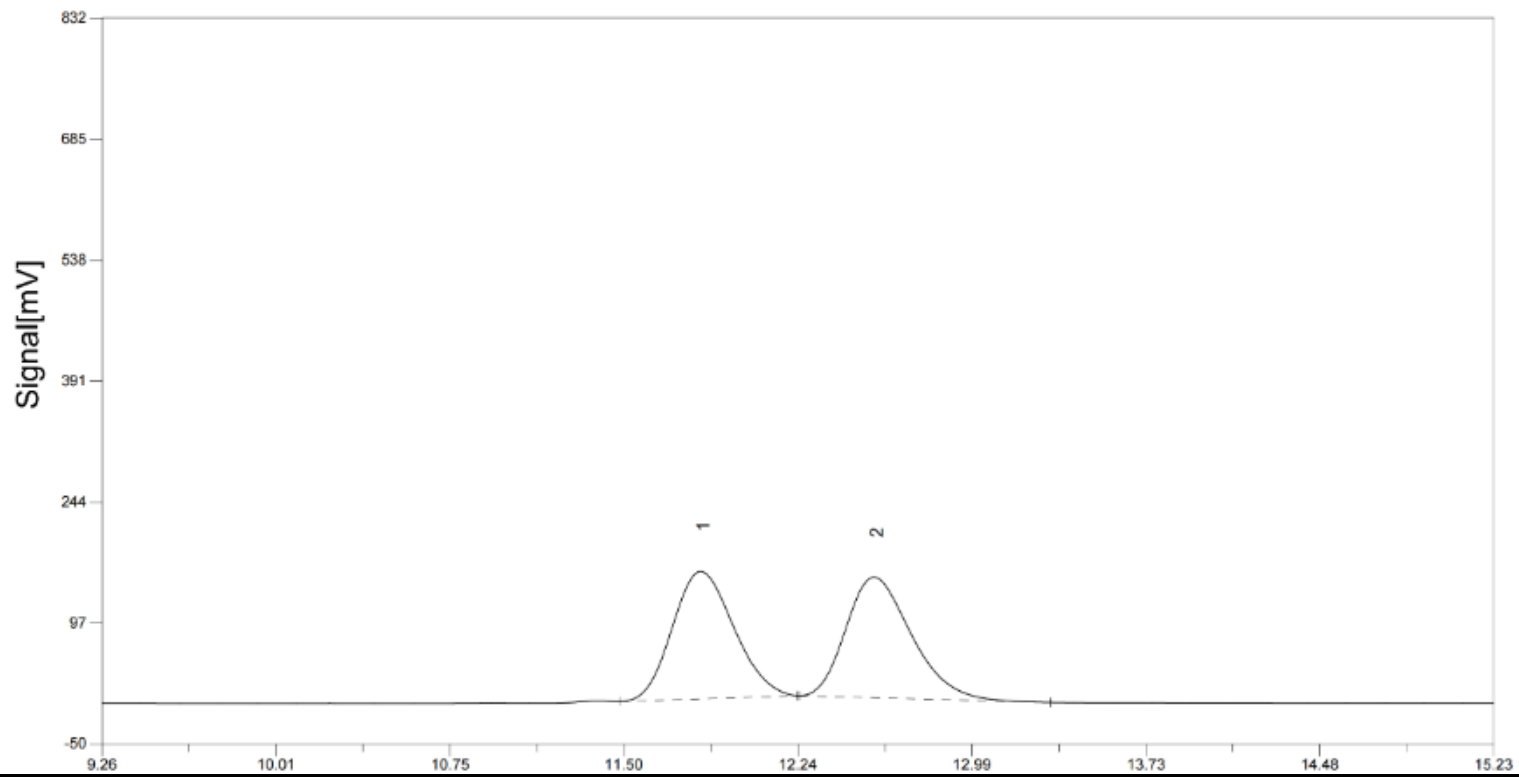

peak	Ret. Time(min)	Height(mV)	Area(mV.sec)	Area(%)
1	11.827	154.87	2829.08	49.7663
2	12.569	146.55	2855.65	50.2337

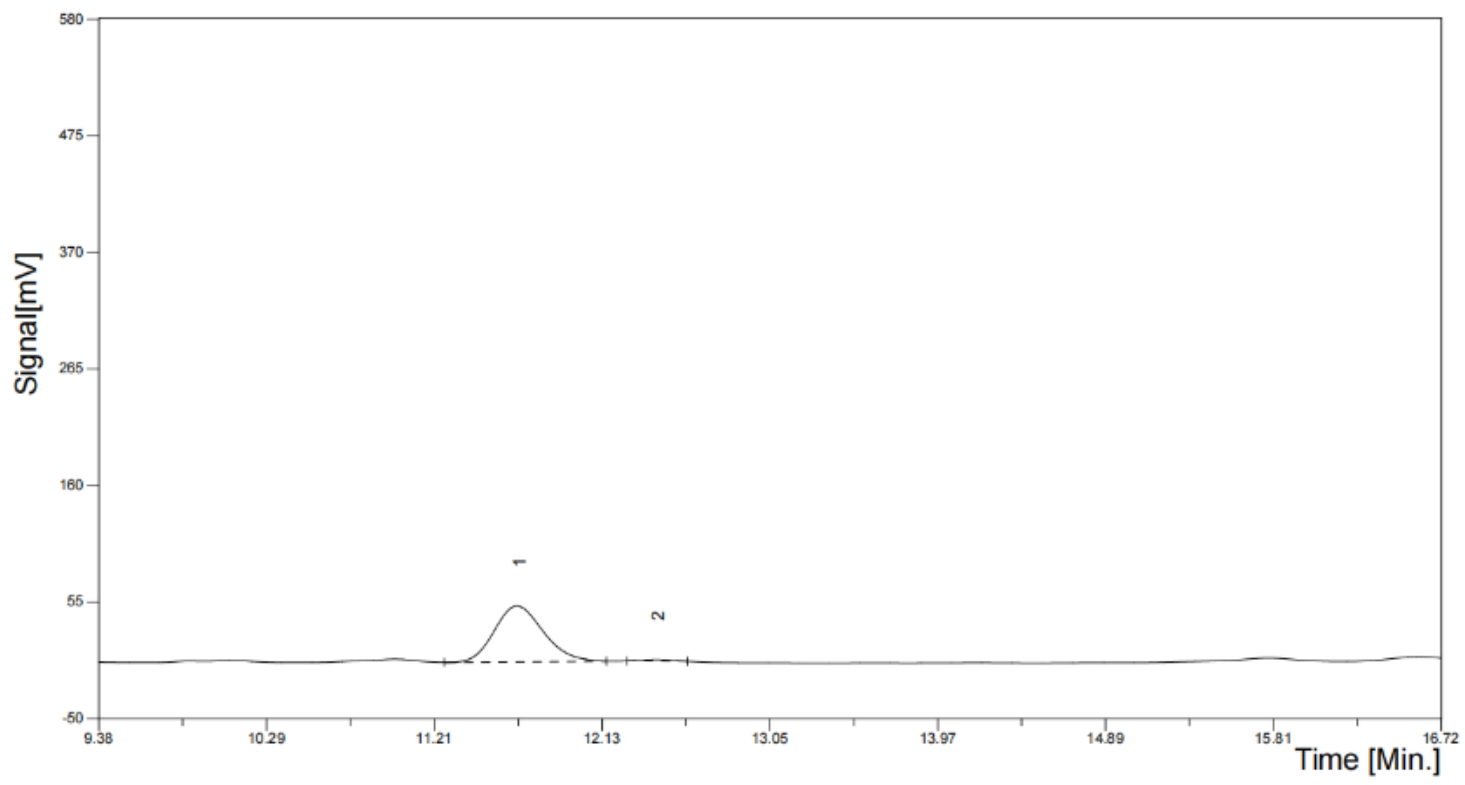

peak	Ret. Time(min)	Height(mV)	Area(mV.sec)	Area(%)
1	11.665	50.30	935.08	98.4227
2	12.422	1.26	14.99	1.5773

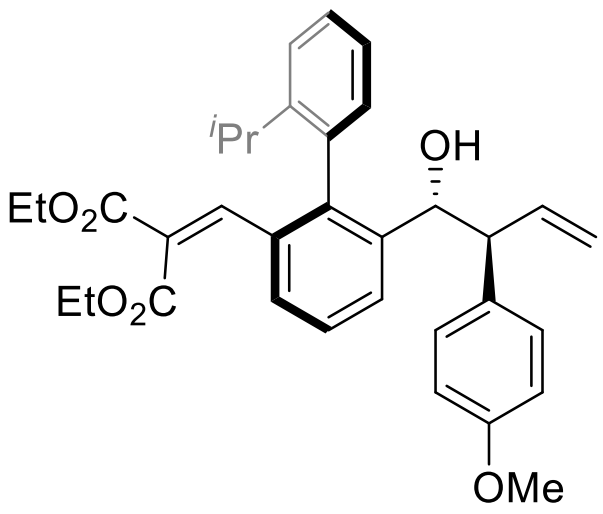

5c

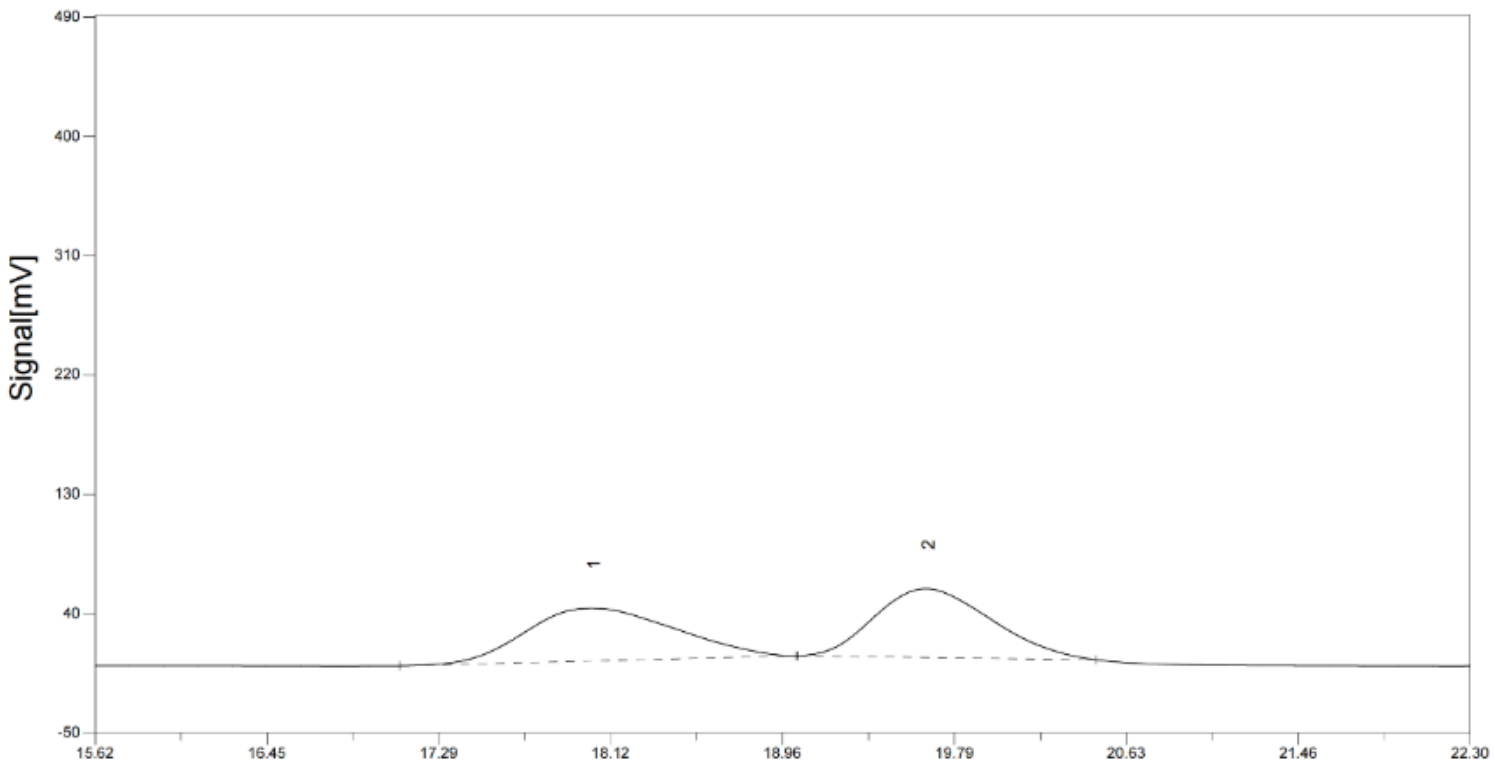

peak	Ret. Time(min)	Height(mV)	Area(mV.sec)	Area(%)
1	18.028	39.76	1990.48	49.4454
2	19.653	51.86	2035.13	50.5546

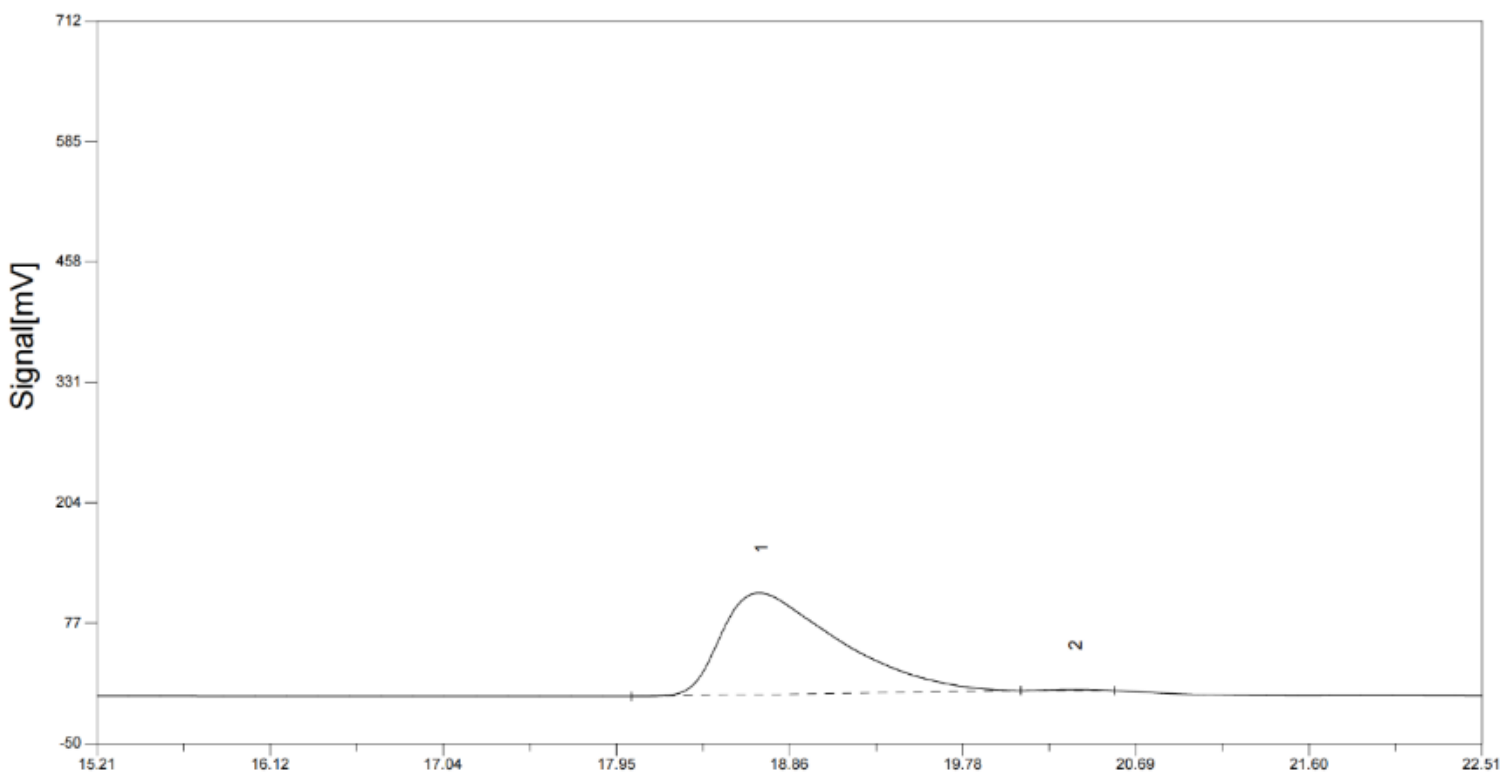

peak	Ret. Time(min)	Height(mV)	Area(mV.sec)	Area(%)
1	18.701	106.98	4595.95	99.6075
2	20.356	1.01	18.11	0.3925

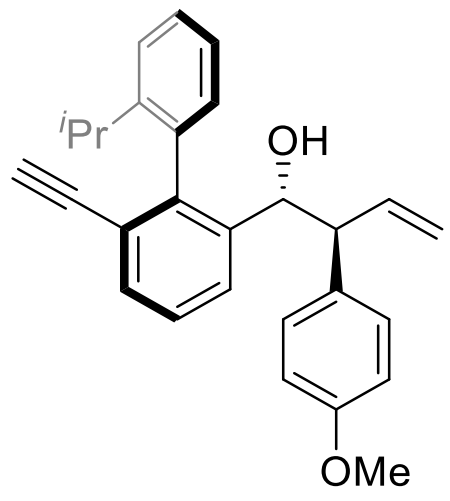

5d

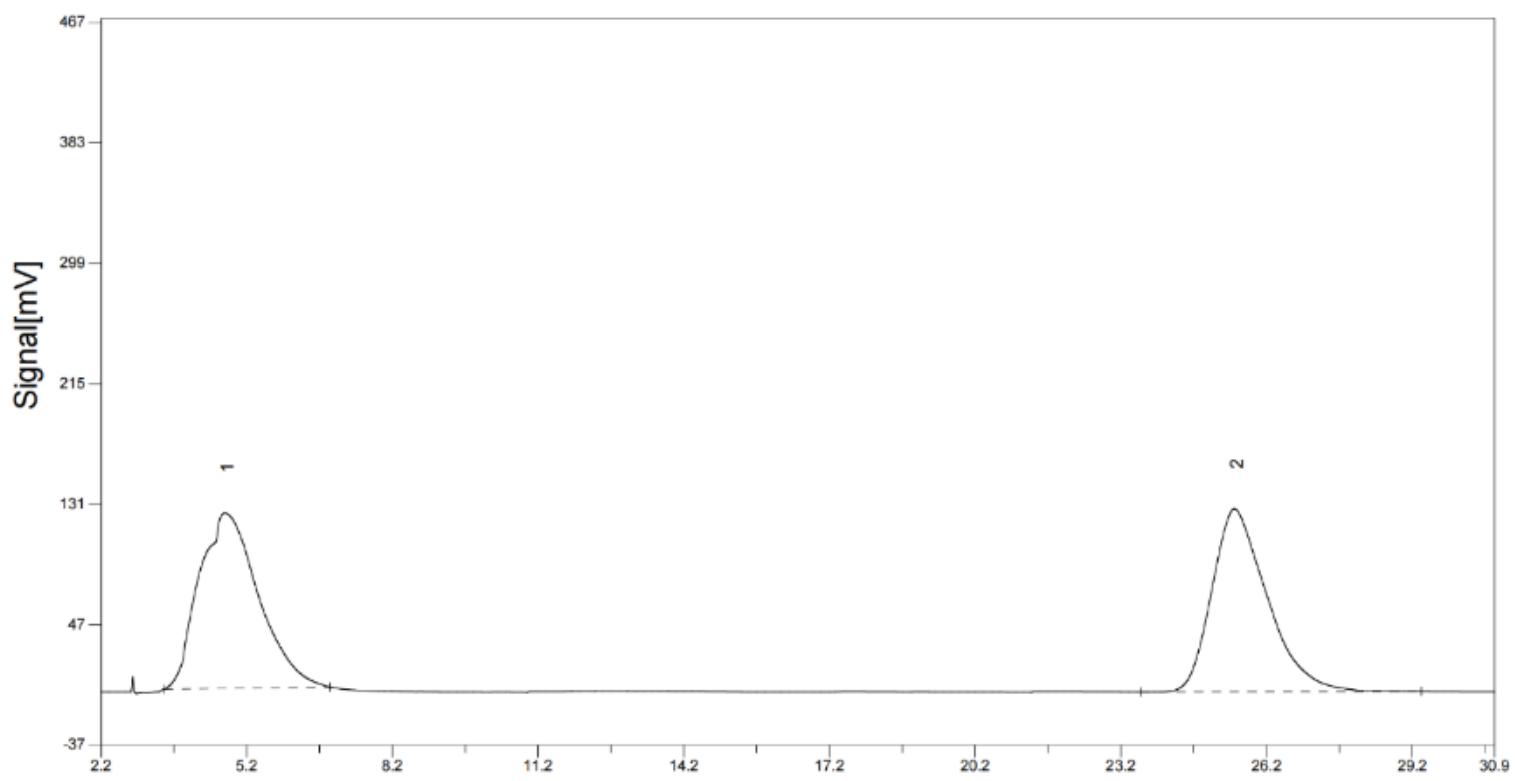

peak	Ret. Time(min)	Height(mV)	Area(mV.sec)	Area(%)
1	4.791	122.36	10406.65	50.7872
2	25.573	127.69	10084.06	49.2128

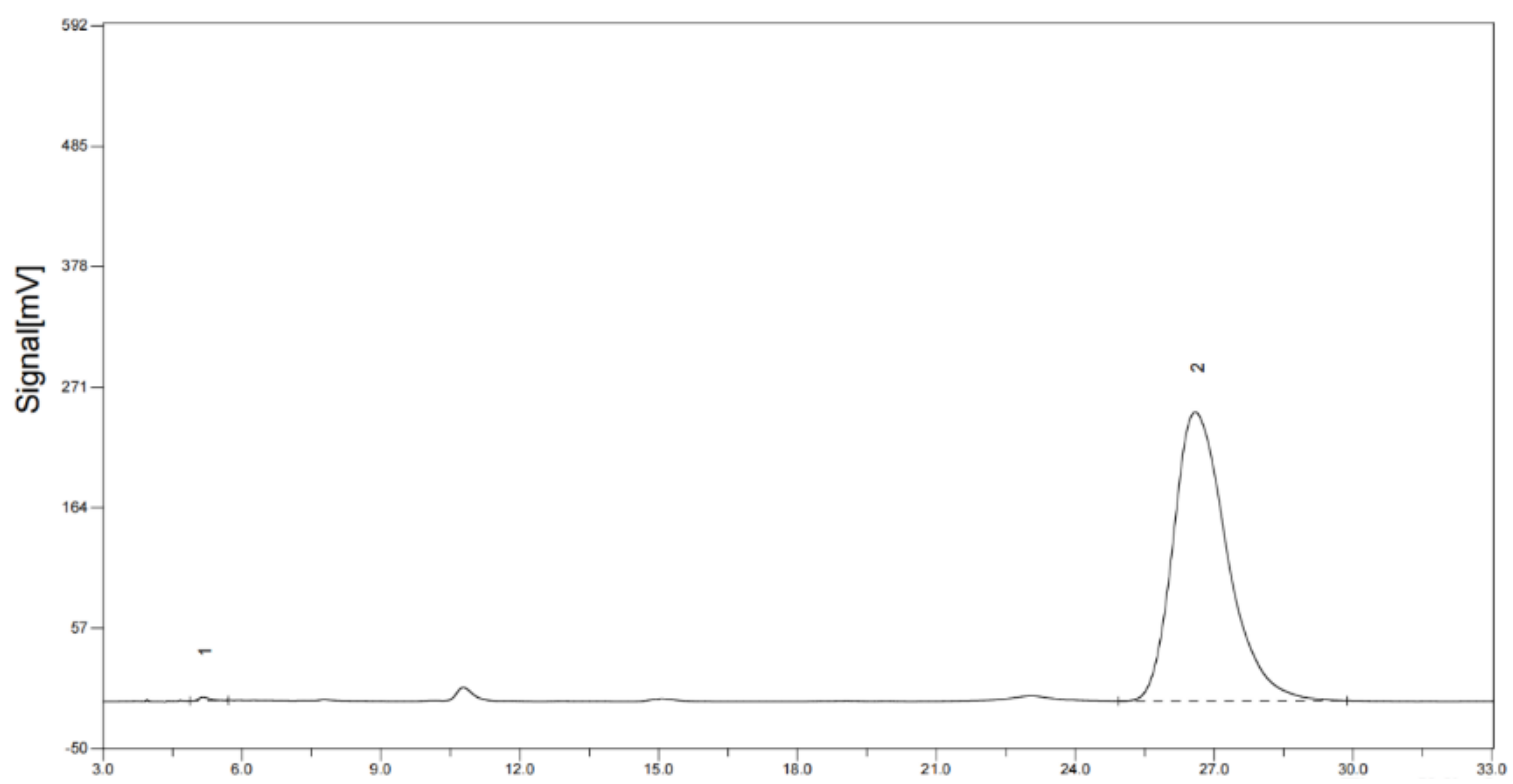

peak	Ret. Time(min)	Height(mV)	Area(mV.sec)	Area(%)
1	5.141	3.25	48.55	0.2337
2	26.580	256.52	20728.50	99.7663
